# Proteomic Analysis of Exudates from Chronic Ulcer of Diabetic Foot Treated with Scorpion Antimicrobial Peptide

**DOI:** 10.1155/2022/5852786

**Published:** 2022-10-03

**Authors:** Zhixiang Tan, Zhiguo Yu, Xiaobo Xu, Lanxia Meng, Mosheng Yu, Rui Tao, Yingliang Wu, Zhanyong Zhu

**Affiliations:** ^1^Department of Plastic Surgery, Renmin Hospital of Wuhan University, Wuhan, Hubei, China; ^2^Department of Emergency, Central People's Liberation Army, Wuhan, Hubei 430060, China; ^3^State Key Laboratory of Virology, College of Life Sciences, Wuhan University, Wuhan, Hubei 430060, China; ^4^Department of Neurology, Renmin Hospital of Wuhan University, Wuhan, Hubei 430060, China

## Abstract

Scorpion peptides have good therapeutic effect on chronic ulcer of diabetic foot, but the related pharmacological mechanism has remained unclear. The different proteins and bacteria present in ulcer exudates from chronic diabetic foot patients, treated with scorpion antimicrobial peptide at different stages, were analyzed using isobaric tags for quantification-labeled proteomics and bacteriological methods. According to the mass spectrometry data, a total of 1865 proteins were identified qualitatively, and the number of the different proteins was 130 (mid/early), 401 (late/early), and 310 (mid, late/early). In addition, functional annotation, cluster analysis of effects and the analysis of signal pathway, transcription regulation, and protein-protein interaction network were carried out. The results showed that the biochemical changes of wound microenvironment during the treatment involved activated biological functions such as protein synthesis, cell proliferation, differentiation, migration, movement, and survival. Inhibited biological functions such as cell death, inflammatory response, immune diseases, and bacterial growth were also involved. Bacteriological analysis showed that *Burkholderia cepacia* was the main bacteria in the early and middle stage of ulcer exudate and *Staphylococcus epidermidis* in the late stage. This study provides basic data for further elucidation of the molecular mechanism of diabetic foot.

## 1. Introduction

Diabetic foot is an ulcer and/or deep tissue destruction and infection which caused by neuropathy of lower extremity and various degrees of peripheral vascular lesions [1]. It is one of the most serious chronic complications of diabetes mellitus, which seriously threatens living quality and is an important cause of death and disability in diabetic patients [2]. Although the specific pathogenesis is still unclear, it is related to many factors such as neuropathy, peripheral vascular disease, artery insufficiency, trauma and infection, history of ulceration or amputation, foot pressure, peripheral edema, and plantar callus formation [3–7].

Wound healing is a complex cascade process involving repair, regeneration, and remodeling of damaged tissue, related to many growth factors and their receptors, extracellular matrix molecules as well as different proteases and their inhibitors. Many studies have identified the important roles of these molecules in the response [8–10]. Diabetic foot ulcer wound healing is associated with protein glycosylation, in which glycated proteins play an important regulatory role [11]. Previous studies have found that Kanglexin significantly alleviated the production of advanced glycosylation end products, which in turn activated FGFR1/ERK signaling, thereby promoting angiogenesis and accelerating diabetic wound healing [12]. lnc-URIDS knockdown promoted the migration of dermal fibroblasts under advanced glycation end products treatment *in vitro* and accelerated diabetic wound healing *in vivo* [13]. In addition, it has also been found that the transcription factor FOXM1 has the function of activating and promoting immune cell survival, and the inhibition of FOXM1 in diabetic mouse models leads to delayed wound healing and reduced recruitment of neutrophils and macrophages in diabetic wounds [14]. Although wound healing has attracted widespread attention, its mechanism needs to be further studied [15].

As a liquid biopsy, extravasate reflects the metabolism of wounds and can effectively identify various factors involved in the restoration of injured skin or those associated with unrestored skin injury [15–17]. In recent years, quantitative proteomics have gradually become one of the hotspots in proteomics research [18–20], of which wound extravasate can elucidate the complex pathological mechanism of chronic ulcer. A large amount of information can identify and quantify the proteome changes related to disease progression or its status [21, 22]. Proteomics, based on mass spectrometry, protein separation, and purification technology, have been applied to the study of chronic ulcer in a small scale [23–25]. Its studies related to wound repair have reported that protein expression in badly healed chronic wounds differs from that in normally healed wounds [23]. Krisp et al. used a multidimensional protein identification technology to investigate and compare the differences in the protein composition of exudates of chronic diabetic foot with wounds in burn patients in 2013. Spectral counting revealed that 188 proteins differentially expressed in chronic wounds, most of which are associated with biological processes, including inflammation, angiogenesis, and cell death [26].

Scorpion venom is a complex mixture containing many bioactive substances with potential medicinal value. Scorpion antibacterial peptide in the venom of *Mesobuthus martensii* can specifically inhibit Gram-positive bacteria, and it can also inhibit the growth of methicillin-resistant *Staphylococcus aureus* and other methicillin-resistant bacteria [27]. In this study, we chemically synthesized this scorpion antimicrobial peptide *in vitro* and developed it into gel preparation. Three patients with diabetic foot treated with this scorpion antibacterial peptide gel were observed. The exudates from chronic foot wounds were collected at different stages of treatment (early, middle, and late). Bacteriological and isobaric tags for relative and absolute quantitation (iTRAQ)-labeled quantitative proteomics analyses were performed on these exudates.

## 2. Materials and Methods

### 2.1. Collection of Samples

**Figure 1:**
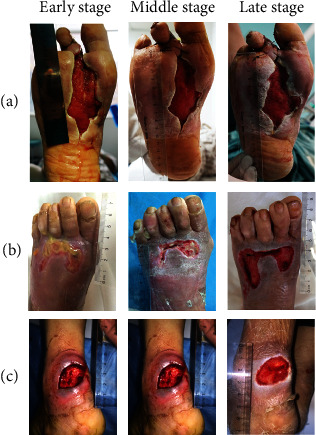
Changes of ulcer wounds in diabetic foot patients during different treatment periods. (a) is the ulcer wound changes in the lesion site of patients with No. 1 diabetic foot after antimicrobial peptide treatment in the early, middle, and late stage; (b) is the ulcer wound changes in the lesion site of patients with No. 2 diabetic foot after antimicrobial peptide treatment in the early, middle, and late stage; (c) is the ulcer wound changes in the lesion site of patients with No. 3 diabetic foot after antimicrobial peptide treatment in the early, middle, and late stages. The initial ulcer areas of the three patients were about 40 cm^2^, 10 cm^2^, and 6 cm^2^, respectively, and the amount of wound exudate was between 100 *μ*L and 2 mL.

Diabetic foot patients came from the department of plastic surgery and endocrinology. The collection and experiment of patients' samples were approved by the Ethics Committee of the Hospital, and the patients signed the informed consent. Inclusion criteria for diabetic foot patients were [1] diabetes mellitus; [2] the average age of the three patients (numbered 1, 2, and 3) was about 60; [3] had type 2 diabetes mellitus for as long as 5-10 years; [4] the foot ulcer and nonunion had been present for 10-40 days; (5) the ulcer areas were about 40 cm^2^, 10 cm^2^, and 6 cm^2^ (Figure 1); [6] the amount of wound exudate was between 100 *μ*L and 2 mL. Exclusion criteria for diabetic foot patients was [1] active cellulitis; [2] osteomyelitis; [3] gangrene; [4] vascular insufficiency (defined as an ankle-brachial index (ABI) < 0.7 and for those with an ABI > 1.3; [5] revascularization to the ipsilateral lower extremity in the last 6 weeks; [6] any experimental drugs taken or applied topically to the wound for 4 weeks preceding the study.

### 2.2. Preparation of Scorpion Antibacterial Peptide Gel

The peptide used in this study is antimicrobial from the venom of *Mesobuthus martensii*. Scorpion antibacterial peptide was chemically synthesized *in vitro* and made into gel preparation. The relevant information is as follows:

#### 2.2.1. Sequence Information

Phe-Ile-Gly-Ala-Ile-Ala-Arg-Leu-Leu-Ser-Lys-Ile-Phe-NH2, xCH3COOH.

Molecular formula: C71H118N18O14 × C2H4O2.

Molecular weight: 1447.83 × 60.02.

#### 2.2.2. Preparation Information

Polypeptide purity > 99%; the concentration of active ingredient was 0.05% (ABP-W1 mass/total mass).

### 2.3. Processing of Samples

According to the ulcer condition of foot wound and the progress of treatment, patients were sampled in three stages: early, middle, and late. The treatment schedule was after the first debridement for each patient at admission, and as the starting point, debridement medication was given to the patient every 5-6 days, lasting about 15 days, and a total of 3-4 treatments were carried out. The wound was washed with water and the residual liquid was dried with sterile gauze. The exudate was drained; a PVA sponge was used to cover the wound, and the outer layer was covered with sterile gauze. After 30 min, the sponge was taken out and wetted with 2-3 ml enzyme inhibitor (1 pill enzyme inhibitor dissolved in 25 ml saline). The sponge was transferred into a 50 ml centrifugal tube and centrifuged (10 min × 4000 g, 4°C) to collect the exudates. The leachate was transferred into a new 1.5 ml EP tube and centrifuged again (10 min × 14,000 g, 4°C) to get the supernatant. The supernatant was filtered (0.45 *μ*m membrane), and the filtrate was collected. BCA protein was quantified and packed separately. The exudate samples of three patients in the same sampling period (early, middle, and late) were merged. The final four groups of samples were A group (serum), B group (early exudate), C group (middle exudate), and D group (late exudate). The samples of each group were frozen at -80°C until use.

### 2.4. SDS-PAGE Separation of Exudate Samples

Separation gel (15%, 15 ml) contained 7.5 ml gel storage solution (30% Acr-Bis), 3.95 ml separation gel buffer (0.4% SDS, 1.5 M Tris-HCl and pH 8.8), 0.15 ml 10% AP, 0.006 ml TEMED, and 3.4 ml ddH_2_O. The stacking gel (4.8%, 5 ml) contained 0.8 ml gel storage solution (30% Acr-Bis) concentrated gel, 1.25 ml stacking gel buffer (0.5 M Tris-HCl, 0.4% SDS, pH 6.8) 0.027 ml 10% AP, 0.006 ml TEMED, and 2.95 ml ddH_2_O [28]. The solutions were allowed to polymerize completely before running. The freeze-dried protein sample (30 *μ*g) was diluted with sample buffer and denatured for 10 min. Next, it was centrifuged at 10,000 g at indoor temperature for 10 min. The supernatant was collected and subjected to electrophoresis. The separating and stacking gels were run at constant currents of 25 mA and 45 mA, respectively. After electrophoresis, the gels were stained using Coomassie blue and destained with solution containing 10% acetic acid, 30% methanol, and 60% water [29].

### 2.5. Bacterial Count and Species Identification

The cotton swab dipped in the exudates of chronic ulcer wound of diabetic foot patients was fully stirred in 5 ml LB medium to release bacteria into the liquid medium. The bacterial medium was diluted properly (0, 10, 50, and 100 times). A volume of 100 *μ*l was taken from each group onto an uncoated LB plate and overnight incubation was carried out at 37°C. Next, the colonies were counted, and the spots were picked up in 500 ml of LB liquid medium. After the reculture for 6 to 8 h, 30% glycerol was added to preserve the strains, which were stored at -20°C and part of which were sent to China Center for Type Culture Collection for identification of strains (16S rDNA sequencing).

### 2.6. Removal of High-Abundance Proteins

Four groups (A, B, C, and D group) of freeze-dried samples (50 mg each) were dissolved in 500 ml PBS. ProteoMiner kit was used for separation according to manufacturer's instructions. SDS-PAGE was used to analyze the protein and detect the depletion effect of the kit on serum high-abundance proteins in the exudates.

### 2.7. iTRAQ Labeling

The protein concentrations were quantified using a BCA Protein Assay Kit. Protein samples (200 *μ*g) were reduced and alkylated, and then purified by trichloroacetic acid (TCA)/acetone precipitation method [29]. The samples were labeled 8-plex iTRAQ according to the manufacturer's instructions as follows: the A group, labeled with iTRAQ reagent 113 and 117; the B group, labeled with iTRAQ reagent 114 and 118; the C group, labeled with iTRAQ reagent 115 and 118; the D group, labeled with iTRAQ reagent 116 and 121. The four labeled groups of polypeptide samples were merged and lyophilized in a vacuum freezer for high-pH RPLC separation [30, 31].

### 2.8. Reversed Phase Reversed Phase Liquid Chromatography Approach at High pH for Protein Separation

The polypeptide mixture was dissolved in buffer A (20 mM ammonium formate aqueous solution, adjusted with ammonium hydroxide to pH 10) and then separated by ultrahigh performance liquid chromatography (with an X Bridge C18 reversed-phase column). Linear gradient was used for separation; buffer B (20 mM ammonium formate dissolved in 90% acetonitrile and adjusted by ammonium hydroxide to pH 10) increased from 5% to 35% within 40 min, and then the column was rebalanced for 15 min under the initial conditions. The flow rate of the column was kept at 200 *μ*L/min at room temperature. A total of 15 elution peaks were collected, and all the components were lyophilized for reserve [32, 33].

### 2.9. Low pH NanoRPLC-MS/MS Analysis

Each elution peak component was suspended in 30 *μ*l solution C (0.1% Formic acid aqueous solution) and then analyzed by nanoLC-ESI-Q-Exactive MS/MS. The experimental equipment was a Q-Exactive mass spectrometer connected to Nano ultrahigh performance liquid chromatography (nanoRPLC) and equipped with electrospray ionization (ESI) [34]. A 5 *μ*L polypeptide sample was loaded onto a C18 precolumn (Acclaim PepMap C18, 100 *μm* × 2 *cm*) for desalting for 3 min (flow rate 10 *μ*L/min), and it was then separated on an analytical C18 column (Acclaim PepMap C18, 75 *μ*m × 15 cm). The linear gradient was used for elution. The solution D (acetonitrile containing 0.1% formic acid) was increased from 2% to 40% within 100 min, and then the column was rebalanced for 15 min under initial conditions at a flow rate of 300 nL/min and column temperature of 40°C. The elution sample was injected into the mass spectrometer by electrospray ionization (ESI) at a voltage of 1.9 kV [35].

### 2.10. Database Search

Proteowizard (version 3.0.9490) was used to extract, deconvolute, and deisotope the Tandem Mass Spectrometry data files. The extracted MS/MS spectra were searched with Mascot (Matrix Science, London, UK; version 2.5.1) [36]. The first searched database was the contaminated protein database provided by MaxPlanc Institute (July 2012 version, 247 sequences) to check if the samples were contaminated. Next, the Uniprot-Swissprot human proteome database (February 2016 version, a total of 20199 sequences) was selected for analysis. To search the Mascot database, the parameters were set as follows: Digestion enzyme type, Trypsin; The number of maximal missed cleavages, 2; MS1 fault-tolerant, 7 PPM; MS2 fault-tolerant, 0.02 Da and cysteine reductive alkylation C +57.03 Da. Lysine and peptide N-terminal ITRAQ8-PLEX modified K/n − term + 304.2 Da residues were selected as fixed modification and methionine oxidation M +16 Da, and tyrosine ITRAQ8-PLEX modified Y +304.2 Da as differential modification.

### 2.11. Qualitative Quality Control of Protein

We used Scaffold (version Scaffold_4.5.1, Proteome Software Inc., Portland, OR) to control and integrate the MS/MS matched peptide segments and protein identification results. The standard for quality control of peptide segments was that the false positive rate was less than 1% based on Scafold Local FDR algorithm, which was acceptable for identification of peptide segments. The standard of protein identification was that each protein matched at least two specific peptide segments, and the false positive rate was less than 1%. Protein information which contained the same peptide segments and cannot be distinguished by MS/MS analysis alone was assembled by Occam's razor rule.

### 2.12. Protein Quantitative Quality Control

Scaffold Q+ (version Scaffold_4.5.1, Proteome Software Inc., Portland, OR) software was used to analyze the quantitative data of peptide segments and proteins. The iTRAQ quantitative intensity of multiple peptide segments of the same protein was normalized and used for the corresponding protein. The intensity of the two reference channels was normalized and the average value was taken as the basis for comparing all groups. We chose 1.3 times of different quantitative value as difference threshold. Using ANOVA test, *P* < 0.05 was significantly statistical. In this study, the total number of quantitative spectrograms was 52 660, accounting for 90% of identified spectrograms (58194).

### 2.13. Bioinformatics Data Analysis

Ingenuity Pathway Analysis (Spring 2016, Qiagen Inc., CA) and Genecards Gene Analytics (2016 LifeMap Sciences Inc., CA) were used to analyze the function, pathway, interaction annotation, and enrichment of differentially expressed proteins.

## 3. Results

### 3.1. Changes in Diabetic Foot Ulcer Wounds at Different Treatment Periods of Antibacterial Active Peptides

We focused on the clinical treatment of three patients with diabetic foot and sampled them at different stages of treatment. After treatment, the physiological condition of ulcer wound was significantly improved; the inflammation reaction of the wound was alleviated, and the suppuration and redness subsided. At the same time, the granulation tissue grew inside the wound; the edge skin contracted inward; the wound area was reduced, and the infection was alleviated. After about 20-30 days of treatment, all the three patients had successful skin transplantation.

### 3.2. Bacteriological Identification of Diabetic Foot Ulcer Wounds

**Figure 2:**
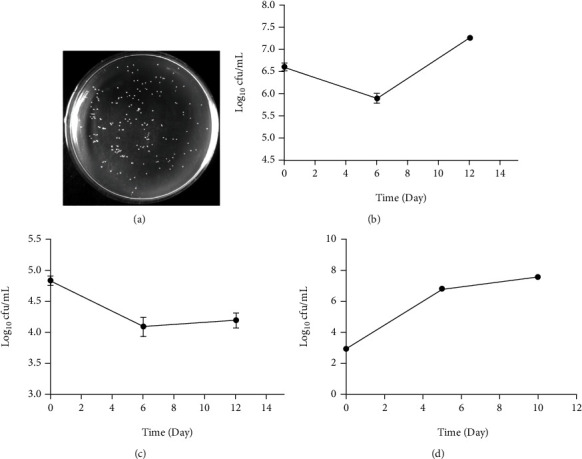
Bacteriological analysis of ulcer wound exudate in diabetic foot patients. (a) The growth morphology of colonies in exudate samples on LB plates, with smooth edges and milky color colonies (about 0.5 mm in diameter). (b) The number of colony-forming units in the exudate of patient No. 1 ranged from 10^4-7^ CFU/mL, and the changes in the early, middle, and late stages were first decreased and then increased. (c) The number of colony-forming units in the exudate of patient No. 2 ranged from 10^4-5^ CFU/mL, and the change trend also decreased first and then increased slightly. (d) The number of colony-forming units in the exudate of patient No. 3 was between 10^3-8^ CFU/mL, with a large range of change and a gradual increase, with a large range in the early and middle stages and a small range in the middle and late stages.

We collected samples of nondisinfected exudates for bacteriological analysis. The colony forming unit (CFU) was counted to reflect the change in the number of living bacteria in the exudates. As shown in Figure 2(a), after incubation for about 20 h at 37°C, a milky white colony (about 0.5 mm in diameter) was formed on the LB plate. The CFU in the exudates of the three patients differed across the different treatment periods. The number of CFU in patients No. 1 was between 10^4-7^ CFU/ml, and the changes in early, middle, and late stages initially decreased and then increased (Figure 2(b)); in patients No. 2, the number of CFU was between 10^4-5^ CFU/ml, and the trend of change first decreased and then slightly increased (Figure 2(c)); in patients No. 3, the number of CFU was between 10^3-8^ CFU/ml. The trend gradually increased with a larger range in the early and middle stages and a smaller range in the middle and late stages (Figure 2(d)). Subsequently, we selected colonies from different periods and sent them to China Center for Type Culture Collection for identification (Supplementary Table S1–3). 16S rDNA sequencing results showed that *Burkholderia cepacia* was the main bacteria in the early and middle exudate samples with number AXBO01000009 and 99.3% similarity, whereas *Staphylococcus epidermidis* was the main bacteria in the late exudate samples with number L37605 and 99.6% similarity.

### 3.3. SDS-PAGE Separation of Exudate from Diabetic Foot Ulcer

**Figure 3:**
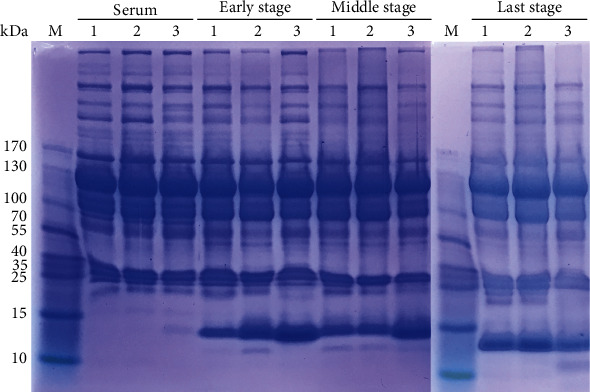
SDS-PAGE separation of serum and exudate protein at different treatment stages.

In order to visualize the changes in exudate proteins at different treatment periods, SDS-PAGE electrophoresis was performed on all the samples. As shown in Figure 3, there were some band differences between exudate protein and serum protein of the three patients at different treatment periods. The most obvious band was below 15 kDa. The protein content of this molecular weight range in serum was low, whereas deep bands appeared in exudate samples. The width and the color depth of the bands were different in different patients at the same period and at different periods for the same patient, indicating that there was a high abundance of differential proteins at this location. In addition, at 20 kDa, 50 kDa, or even higher molecular weight (more than 200 kDa), there were also varying degrees of differences.

### 3.4. Loss of High-Abundance Protein in Exudate of Diabetic Foot Ulcer

Exudates contained many serum high-abundance proteins, such as apolipoprotein APO family, complement protein CO family, fibronectin FIB family, and albumin. Therefore, in order to improve the identification of low-abundance proteins by mass spectrometry, we first carried out a reduction of serum high-abundance protein before quantitative proteomic analysis to reduce the content of these proteins (not all removed). After treatment, the high abundant proteins in the four groups of samples were effectively removed, and the low abundant proteins were effectively concentrated (with clear bands and deeper concentration). Concentrated eluent proteins were used for subsequent isotope labeling, chromatographic separation, mass spectrometry identification, and analysis.

### 3.5. Quantitative Proteomic Analysis of Exudate from Diabetic Foot Ulcer

#### 3.5.1. Statistics of Mass Spectrometry Identification Results

Four groups of iTRAQ labeled samples (A (normal serum), B (early exudate), C (middle exudate), and D (late exudate)) were detected by mass spectrometry. Mascot, Scaffold, and IPA software were used to analyze the mass spectrometry data. According to the results of mass spectrometry of all the sample groups, 444494 MS/MS spectra were obtained. Qualitative identification criteria used each identified protein to obtain at least less than 1% of the false positive rate (FDR), two specific matched peptide fragments, less than 1% of the false positive rate of protein. According to the standard, we identified a total of 58194 spectra corresponding to 1865 proteins (Supplementary Table 4), accounting for about 13% of the total spectrum (58194/444494), which belonged to the normal level in the serum proteome.

In the quantitative identification, the isotope labeling efficiency of the samples was more than 99%, and the consistency of labeling signals among the samples was good. The average coefficient of variation of protein quantification was 20%. The total number of quantitative spectrograms was 52660, accounting for 90% of qualitative identification spectrograms (58194). Quantitative data were analyzed using the criteria of 1.3-fold change and nonparametric test *P* < 0.05 to screen differentially expressed proteins. Four groups were compared and analyzed: BCD group/A group, C group/B group, D group/B group, and CD group/B group. The number of differential proteins was shown in Table 1. According to the screening criteria of 1.3-fold change, 29 proteins were downregulated and 101 proteins were upregulated in C group compared with B group; 82 proteins were downregulated, and 319 proteins were upregulated in D group compared with B group; 56 proteins were downregulated, and 254 proteins were upregulated in CD group compared with B group; and 1153 differential proteins were identified in BCD group compared with A group (*P* < 0.03067) (Supplementary Table 5).

The differential proteins in the different groups were mainly upregulated. Specific to the corresponding molecule, the upregulation of S100-A7 (immune response and angiogenesis), S100-A2 (endothelial cell migration related), calmodulin-like protein 3, protocadherin-18 (cell movement, migration, etc.), 14-3-3 protein Sigma (cell migration, cytokeratinization, etc.), tropomyosin alpha-4 chain (migration of skeleton and muscle cells), and 60S ribosomal protein L19 (protein synthesis) were clearer. The downregulation of collectin-10 (glycometabolic metabolites), complement C5, complement component C6, Mmannose-binding protein C (complement activation), grancalcin (inflammatory response), beta-Ala-His dipeptidase (carnosine hydrolysis and cell death), tumor necrosis factor-inducible gene 6 protein (cell death), and serum amyloid A-1 protein (immune response) was more evident.

### 3.6. Statistics and Analysis of Differential Proteins

#### 3.6.1. Function Annotation

We used Ingenuity Pathway Analysis (IPA) software to annotate the function, signal pathway, and interaction analysis of differential proteins. Firstly, the annotations were made on 130 differential proteins in intermediate exudate (C group) compared with early exudate (B group) and 401 differential proteins in late exudate (D group) compared with early exudate (B group) in order to investigate their biological functions, toxicological functions, and regulatory functions. A total of 500 classifications (subcategories) in C/B group and 500 classifications (subcategories) in D/B group were annotated, but their reliability and the degree of activation/inhibition varied (Supplementary Table 6).

As shown in Table 1, biological functions were identified with high probability (|*Z* *value*| > 2 or ≈ 2) from the differential proteins of C group (intermediate exudate) and B group (early exudate). The main activated physiological effects were cell movement (involving proteins such as GBP1, ACP1, PPIA, and TYMP) and migration (ACP1, PPIA, IGHG1, S100A7, etc.), cell differentiation (PPIA, IGHG1, GLO1, LTF, etc.), body injury and regeneration system (HIST1H1C, PRRC1, C9, TYMP, etc.), tissue survival (KRT14, MIF, HP, APOA1, etc.), protein synthesis (RPL27A, KRT17, MAPK3, RPS9, etc.), cell invasion (MIF, GBP1, KRT17, IGHG1, etc.), and skin formation (KRT14, TGM3, KRT16, CALML5, etc.). Inhibited functions included gangrene-induced cell death (HIST1H1C, C9, ACP1, PPIA, etc.), leukocyte recruitment and inflammatory response (APOA1, MBL2, LTF, APOH, LBP, C5, etc.), nerve cells (MIF, P4HB, TF, C9, etc.), tissue cell death (RPL27A, RPS19, ENO1, MIF, etc.), and bacterial growth (APOA1, LTF, TF, GSN, etc.). In D group (late exudate) compared with B group (early exudate), most of the physiological effects of activation were similar to those of C/B group, such as protein synthesis, cell proliferation, differentiation, migration, movement, survival, invasion, and tissue survival. The inhibited physiological effects were similar to those of C/B group, such as cell death (including natural apoptosis, gangrene, body injury, loss of regulation of connective tissue, bone, muscle disorders, etc.), inflammatory diseases, inflammatory reactions, immune disease, etc.

In summary, these biological functions, which were clearly activated or inhibited, reflected the changes of microenvironment in the healing process of diabetic foot ulcer wounds (mid/early and late/early). In other words, the enhancement of protein synthesis, cell proliferation, differentiation, migration, and movement facilitates cell growth and survival as muscles, skin, blood vessels, and other tissues continued to form and survive. At the same time, cell and tissue death, inflammatory reaction, inflammatory disease-related molecules, and bacterial growth were reduced in the injured area. The comprehensive effects of all the biological processes mentioned above were conducive to the repair and healing of the injured area.

#### 3.6.2. Statistics of Functional Annotations

**Figure 4:**
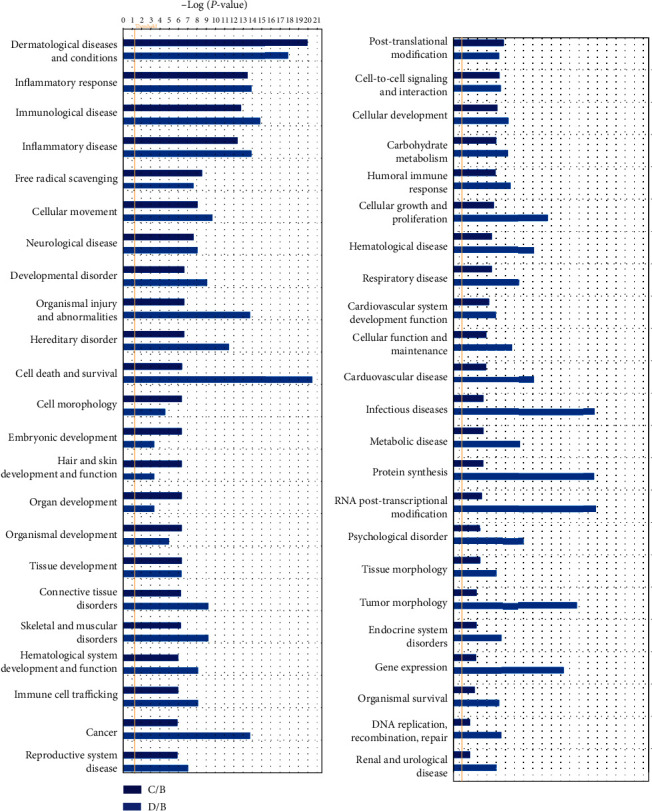
Classification statistics of differential proteins about exudates in different periods. The dark blue column gram showed the classification of differential proteins in C group (medium exudate) compared with B group (early exudate). The light blue column gram showed the classification of differential proteins in D group (late effusion) than that of the B group (early effusion).

The function classifications of Supplementary Table 6 were analyzed by histogram, and the maximum value of -log (*P* value) of each class was displayed. Each class contained multiple subclasses, and the extreme value was taken (Figure 4). From Figure 4, it was shown that in the classification of differential proteins in C group (middle exudate) and B group (early exudate), the most involved functions were skin diseases and health (−log (*P* value) ≈ 20), followed by inflammatory reactions, inflammatory diseases, and immune diseases (−log (*P* value) between 12 and 14), and other functions involved in free radical scavenging, cell movement, nervous system diseases, developmental disorders, body damage and abnormalities, genetic abnormalities, cell death and survival, cell morphology, formation and function of hair and skin, organ and tissue development, blood system development and function, immune cell recruitment, regenerative system diseases, etc. In the classification of differential proteins in D group (late exudate) and B group (early exudate), the number and reliability related to cell death and survival as well as skin diseases and health were the highest, followed by those related to posttranscriptional modification of RNA, infectious diseases, protein synthesis, and immune diseases. Inflammatory diseases, inflammatory reactions, body injury, gene expression, cell growth and differentiation, cell migration, connective tissue, skeletal tissue, muscle tissue disorders, developmental disorders, blood system development and function, immune cell recruitment, neurological diseases, and other related differential protein classification were also highly reliable. In summary, the middle and late-stage differential proteins were more involved in the functions of skin diseases and health, inflammatory reaction, immune diseases, and inflammatory diseases. Comparisons of other functions suggested that the reliability was approximately the same. Except in the late stage (D/B group), cell death and survival, cell growth and differentiation, gene expression, posttranscriptional modification of RNA, protein synthesis, body injury, and infectious diseases were higher than those in the middle stage (C/B group), which can be attributed to a higher number of differential proteins identified.

In summary, it was evident that the causes of chronic ulcer wound and nonunion in diabetic foot may be connected to abnormal cell death, abnormal development and regeneration of nerve, blood vessel, blood system, connective tissue, muscle tissue, excessive inflammation, inflammatory disease, and immune system disease. Bacterial and viral infections were also associated with chronic ulcer wound in diabetic foot. Therapeutic processes were the recovery and regeneration (the growth and regeneration of nerves, blood vessels, muscles, connective tissue, blood system, etc.) and the decline (cell death, immune diseases, inflammatory diseases, viruses and bacterial infections) of physiological or pathological processes.

**Figure 5:**
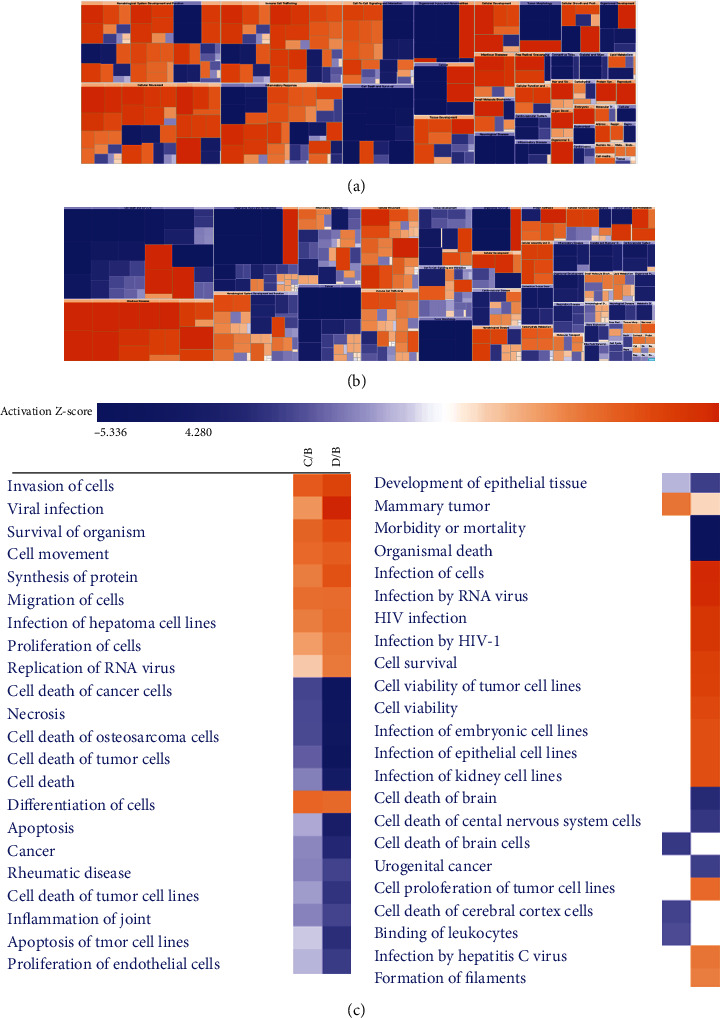
Heatmap of clustering analysis of differential proteins in exudates at different stages. (a) The clustering analysis of differential protein effect in C group (middle exudate) compared with that in B group (early exudate). (b) The clustering analysis of differential protein effect in D group (late exudate) compared with that in B group (early exudate). (c) Analysis of trends in effects of differential proteins in CD/B group.

Further, we analyzed the class and the descendants of annotations of differential proteins and their subclasses by Heatmap of clustering analysis (Figure 5). It was shown that in the effect clustering of differential proteins in C group (middle exudate) compared with B group (early exudate), cell development and movement, cell growth and differentiation, protein synthesis and regeneration system, antimicrobial infection, tissue development, and skin and hair development were almost completely activated (red), reflecting the active metabolism and tissue repair environment in the wound site (Figure 5(a)). The development and function of the blood system, the recruitment of immune cells, cell movement, inflammatory reaction, signal transduction, and interaction between cells were activated and partly inhibited, which was mainly due to the difference in the specific functions of subclasses under each large class, but the general mechanism was the construction of microenvironment to promote the repair of damaged tissues and abnormality. In addition, the inhibition (blue) of cell death, organism injury and abnormalities, inflammatory diseases, nervous system diseases, regeneration system abnormalities, connective tissue, and muscle tissue abnormalities was also consistent with the findings in C/B group. As shown in Figure 5(b), the inhibitory effect (blue) of Heatmap of clustering analysis on differential proteins was more extensive in D group (late exudates) than that in B group (early exudates). It involved cell death and survival, organism injury and abnormalities, inflammation, cancer and tumor, and tissue and organ development. Activation effects included infectious diseases, cell movement, cytoskeleton assembly, protein synthesis, etc. It was showed that the functions associated with the coactivation of different proteins in the middle and late stages included cell invasion, cell survival, cell movement, migration, differentiation, protein synthesis, etc. in Figure 5(c). The functions associated with coinhibition included cell death, apoptosis, gangrene, inflammatory reaction, and the specific activation of anti-infective functions in the late stage.

In the wound healing process of diabetic foot ulcer, tissue regeneration and recovery were more extensive in the midterm. In the late stage, cell and tissue death, cell division, and tissue regeneration as well as infection and inflammation were suppressed. At the same time, there were common activation and inhibition effects, and some of the biological effects were different in the middle and late stages. Moreover, significant differences also existed among some of the biological functions in the middle and late stages.

#### 3.6.3. Classical Pathway Analysis

We performed classical signal pathway analysis of differential proteins (Supplementary Table 7) to investigate the regulation of activation and inhibition of the pathways involved, thus confirming the relevant pathway of evident disorders. It can be seen from the table that EIF2 signaling, cardiac hypertrophy signaling, phospholipase C signaling, and dendritic cell maturation were more obvious activation pathways of differential proteins in C group than in B group. Significant disorder pathways (−log (*P* *value*) were larger, but no consistent activation or inhibition information were acute phase response signaling, completion system, glycolysis I, LXR/RXR activation, regulation of eIF4 and p70S6K Signaling, FXR/RXR Activation, gluconeogenesis I, adenine and adenosine salvage I, systemic lupus erythematosus signaling and mTOR signaling, etc. EIF2 signaling was the only pathway that was more significantly activated in D group (late exudate) than in B group (early exudate). There were many other pathway disorders (−log (*P* value) = 2 − 9) with no apparent consistent activation or inhibition information, such as regulation of eIF4 and p70S6K signaling, LXR/RXR activation, acute phase response signaling, completion system, mTOR signaling, FXR/RXR activation, glycogen Degradation II, etc.

We also investigated the biological functions involved in some signaling pathways. EIF2 signaling, as well as regulation of eIF4 and p70S6K signaling, was eukaryotic signaling pathways mainly involved in protein synthesis, cell proliferation and growth; mTOR signaling was also related to cell growth and proliferation. LXR/RXR and FXR/RXR activation participated in lipid metabolism and acute phase response signaling involved inflammation and participated in nonspecific defense against microorganisms, mostly in damaged tissue and wound sites. Complement system was associated with innate and acquired immunity; systemic lupus erythematosus signaling was associated with autoimmune diseases and cardiac hypertrophy signaling was associated with cardiovascular diseases. Dendritic cell maturation was an important initiation factor of cellular immune response and glycolysis I and gluconeogenesis I involved glycolysis and glucose metabolism.

**Figure 6:**
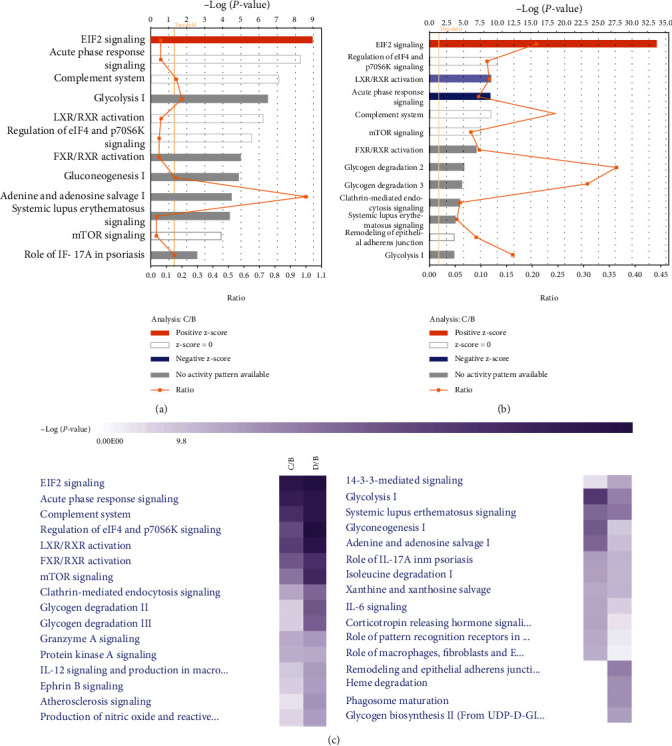
Statistics of signal pathways involved in differential proteins in exudates at different stages. (a) The signaling pathway analysis of differential proteins in C group (middle exudate) compared with that in B group (early exudate). (b) The signaling pathway analysis of differential proteins in D group (late exudate) compared with that in B group (early exudate). (c) Trend analysis of intergroup signaling pathways.

In order to visualize the signal pathways involved, the histogram statistics of Supplementary Table 7 (Figure 6) were carried out. The pathways shown in Figures 6(a) and 6(b) were graphical transformations of Supplementary Table 7, and the results were consistent with those shown in tables. Most of the signal pathways involved in C/B group and D/B group were common, but the number of identified differential proteins was different, which identified for late stage was higher, and hence, more signal pathways were involved in the late stage.  Figure 6(c) showed the trend analysis of the signaling pathways of the two groups. The *Z* value (response activation or inhibition of the signaling pathways) of pathway analysis was low; *P* value (which reflected the signaling pathways disorder or not) was used as the judgment standard. The deeper the purple color, the more significant the effect of disorder of the pathway. As shown in Figure 6(c), EIF2 signaling, acute phase response signaling, complement system, LXR/RXR activation, FXR/RXR activation, regulation of eIF4, p70S6K signaling, and mTOR signaling were the signal pathways with obvious disorders in the middle and late stages. The functions involved were basically consistent with the previous intergroup trend analysis. Most of the pathways were related to protein synthesis, cell proliferation and growth, and metabolic processes as well as inflammation and immune system.

#### 3.6.4. Upstream Transcriptional Regulation Analysis

To understand the transcriptional regulation of differential proteins, we analyzed the upstream regulatory factors of differential proteins (Supplementary Table 8). Three regulators, IgG, RICTOR, and MAPK1, were more inhibited in C group (middle exudate) than in B group (early exudate) (Table 1). Eleven regulators, ROCK2, MYC, MYCN, EGFR, HIF1A, IFNG, ERK1/2, IL5, ARNT2, TP73, and ESR1 were more activated in C group (middle exudate) than in B group (early exudate). Ten regulators of differential proteins, including RICTOR, IgG, CD3, FAAH, FMR1, MAPK1, ACOX1, and AMPK, showed more inhibited activities in D group (late exudate) than in B group (early exudate). In addition, 45 regulators, including MYCN, MYC, TGFB1, ROCK2, KITLG, PPARA, HRAS, EGFR, IFNG, HIF1A, NFE2L2, and FN1, showed more activated activity in D group than in B group.

**Figure 7:**
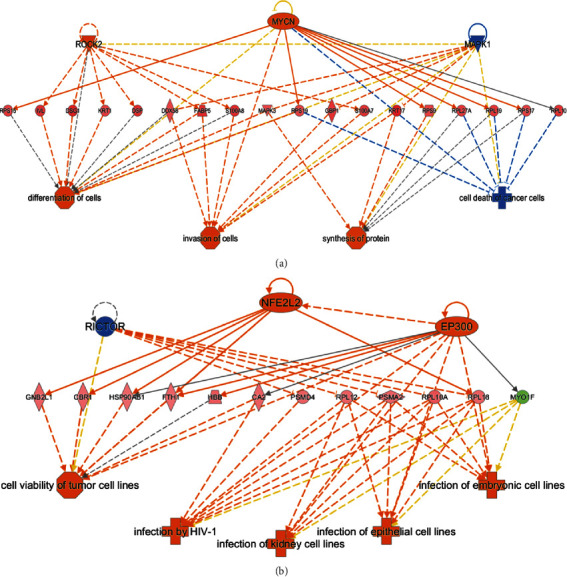
The interaction network and downstream function of upstream regulators. (a) Regulators derived from differential proteins in C/B group. (b) Regulators derived from differential proteins in D/B group.

Furthermore, each subnetwork of possible biological functions of some transcriptional regulators can be clustered to find a common regulator-function network. As shown in Figure 7(a), the three regulators, ROCK2, MYCN, and MAPK1, had interlaced downstream biological functions, however, they can ultimately affect the differentiation, migration, protein synthesis, and death of cells. In Figure 7(b), three regulators, RICTOR, NFE2L2, and EP300 influenced cell survival and infection through interactive downstream regulatory proteins.

#### 3.6.5. Interaction Network

**Figure 8:**
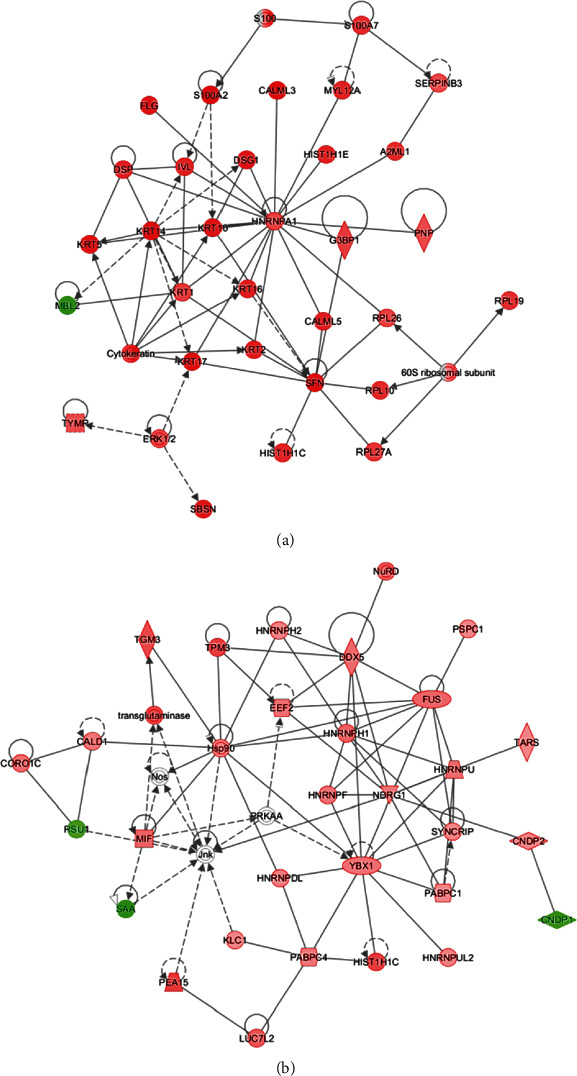
Interaction network of differential proteins. (a) The first interaction network in C/B group. (b) The third interaction network in D/B group.

We analyzed the possible interaction networks in differential proteins (Supplementary Table 9). As shown in the table, the main functions of the three interaction networks with higher reliability in C group (middle exudate) than those in B group (early exudate) were skin diseases, immune diseases, inflammatory diseases, carbohydrate metabolism, posttranslational modification, protein folding, developmental disorders, and others. In D group (late exudate), the main function of interaction network was similar to that in C/B group, but the reliability was slightly higher than that in C/B group, which was related to higher number of differentially identified proteins.  Figure 8(a) showed the first interaction network in C/B group. The differential proteins under this network were mainly related to the functions of skin diseases, immune diseases, and inflammatory diseases. The key proteins that interacted with other proteins were HNRNPA1, KRT14, SFN, and so on.  Figure 8(b) showed the third interaction network in D/B group. The differential proteins involved were related to posttranscriptional modification of RNA, infectious diseases, and protein synthesis. The core proteins were HNRNPH1, NDRG1, Hsp90, FUS, JnK, etc.

## 4. Discussion

There were two main treatments for the patients of diabetic foot, which were oral administration of drugs and surgical treatment. Oral administration or injection of drugs reduced blood sugar, prevented infection, and improved blood circulation. For surgical treatment, the ulcer wound was cleaned up, sterilized, and smeared with scorpion peptide antibacterial gel. Negative pressure suction device was installed to drain the exudate, which promoted blood circulation and oxygen supply to the wound. After a period of treatment, when the wound infection has reduced, the blood circulation was good, and the granulation tissue began to grow; autologous skin flap transplantation was carried out to completely repair the wound. The three patients of diabetic foot enrolled in this study had different ulcer formation time (10-40 days), wound location, and size. After a certain period of clinical treatment, the wound was greatly repaired, and ultimately after autologous skin flap transplantation, the wounds of the three patients were basically healed. During the whole process, wound exudates at different stages reflected the changes in wound microenvironment and the biological and pathological processes involved. Conventional proteomics can only qualitatively identify the protein composition of a sample, but the disease process is often a dynamic change. In this case, quantitative analysis of protein components is particularly necessary. In addition to conventional drug therapy, surgical debridement and negative pressure suction, scorpion peptide antibacterial gel in the treatment process, may have a potential effect against excessive inflammation and purulent secretion, and they may promote granulation tissue growth and accelerate the healing of wounds in addition to killing pathogens (antibacterial peptide effect). However, this is only based on clinical observation and medication experience. The specific mechanism of antibacterial peptide in this process needs to be elucidated.

Bacteriological analysis of exudates showed that the number of pathogenic bacteria in wound site decreased; then, normal flora was observed in the late stage. We can conclude that scorpion antimicrobial peptide played an important role. *Burkholderia cepacia* (also known as *Pseudomonas cepacia*), is a human conditional pathogen in hospitals, which can cause sepsis, pneumonia, abscess, and wound infection, especially in elderly patients, patients with low immunity and being treated for trauma [37]. *Staphylococcus epidermidis*, a Gram-positive bacterium, is a normal flora on the surface of human skin [38]. The main bacterial floras of wound exudates vary at different periods. The number of CFU of *Burkholderia cepacia* decreased from early to middle stage, which may be as a result of a variety of treatments including the scorpion antimicrobial peptide. In the late stage, the main flora was *Staphylococcus epidermidis*, a normal parasitic bacterium on skin surface, and the number of *Burkholderia cepacia* CFU naturally increased.

The SDS-PAGE separation of exudate proteins showed that there were differences in protein bands between the samples at different stages, and the components of exudate and serum were also different. Quantitative mass spectrometry data of exudate samples showed that the number of differential proteins in early, middle, and late stages was within the normal range, but there were 1153 differential proteins compared with the serum control group in the case of 1865 proteins identified. However, 600-800 differential proteins were usually identified in normal human serum, indicating a considerable difference. Therefore, in the follow-up data analysis process, we did not take the serum group into account, but only analyzed the differential proteins of the wound exudates at different periods, which was enough to reflect the dynamic process of the disease.

Quantitative mass spectrometry with iTRAQ labeling can not only qualitatively analyze sample proteins but also analyze differential proteins between samples [39]. Through in-depth analysis of the functional types, predictive activities, signaling pathways, transcriptional regulation, and protein-protein interaction networks involved in these differential proteins, we could clearly observe the biological effects as well as physiological and pathological changes of ulcer wounds at different stages. This information provided valuable reference to facilitate the understanding of the occurrence and treatment of diabetic foot. In terms of quantitative results, filter criteria were set as 1.3 times difference threshold and *P* < 0.05; 130 differential proteins were identified from the middle-term exudates; 401 differential proteins were identified from the late exudates. There were 310 differential proteins identified which emerged concurrently in the middle and late stages. Specific differential proteins, such as S100 calcium binding proteins A2, A8, A7, and A6, were highly expressed in ulcer wounds, which was consistent with previous reports [26]. However, there was no significant difference in matrix metalloproteinases, which were usually highly expressed in chronic ulcer wounds [40], and they can degrade collagen and gel, hindering wound healing. In this study, the reasons for the large differences in enzymes may be related to individual differences, different sensitivity of detection methods or decreased concentration of enzymes during wound healing [41], which was not within the standard of significant quality control. However, we detected more differential molecules, covering a wider range of functions and physiological effects.

The annotations of these differential proteins showed that the main activated effects in the middle and late stages included protein synthesis, cell proliferation, differentiation, migration, movement, invasion, and tissue survival. Cell death, inflammatory diseases, inflammatory reactions, and immune diseases were all inhibited. However, both activation and inhibition in the late stage involved more protein molecules and more specific classifications with higher reliability. This indicated that in the late stage, the wound exhibited a stronger and faster self-repairing effect. Overall, these biological functions, which were either activated or inhibited, reflected the microenvironment changes associated with the healing process of diabetic foot ulcer wounds. Skin diseases and health, inflammatory response, and immune diseases were more involved both in the middle and late stages. Heatmap and clustering analysis of differential proteins in exudates at different stages showed that in the wound healing process of diabetic foot ulcer, the midterm involved tissue regeneration and recovery, and the late stage involved the inhibition of cell and tissue death, slowing down cell division and tissue regeneration as well as the suppression of infection and inflammation. There were common activation and inhibition effects as well as significant biological effects in the middle and late stages. Finally, we conducted transcriptional regulation analysis and protein interaction network analysis, revealing the complex regulatory mechanism and interaction among differential proteins.

In this study, through quantitative proteomic analysis, we confirmed that the causes of chronic ulcer wound and nonunion in diabetic foot may be related to abnormal cell death, abnormal development and regeneration of nerve, blood vessel, blood system, connective tissue, muscle tissue, excessive inflammatory reaction, inflammatory disease, immune system disease, and bacterial and viral infections. Therapeutic processes involved the recovery (the development and regeneration of nerves, blood vessels, muscles, connective tissue, blood system, etc.) and the decline (cell death, immune diseases, inflammatory diseases, inflammatory reactions, and viral and bacterial infections) of these physiological or pathological processes. Although the specific targets and mechanisms of scorpion antimicrobial peptide in the treatment process still need to be verified by more in-depth and more specific experiments. The result of the proteomics analysis provided a differential protein molecular map of diabetic foot ulcer wounds, which provided some basic data and references to further elucidate the molecular mechanism of the development and treatment of the disease and identify diagnostic markers of healing in chronic wound.

## Figures and Tables

**Table 1:** Quantitative differential protein statistics of chronic ulcer exudates from diabetic foot.

Fold change	Group comparative analysis			
	BCD group/A group	C group/B group	D group/B group	CD group/B group
1.3 times downregulation	1153 (*P* < 0.03067)	29	82	56
1.3 times upregulation		101	319	254

A (normal serum); B (early exudate); C (middle exudate); D (late exudate).

## Data Availability

The datasets used and/or analyzed during the current study are available.

## Additional Points

*Key contribution.* Proteomic analysis of exudate from diabetic foot wounds treated with scorpion venom antibacterial peptide provided a differential protein molecular map of diabetic foot wounds, which further clarified the molecular mechanism of disease development and treatment and identified the diagnosis of chronic wound healing landmark.

## Supplementary Materials

Supplementary Materials Bacteriological identification of diabetic foot ulcer wounds is available on Supplementary Table 1–3. Identification results by mass spectrometry is available on Supplementary Table 4; analysis of proteins in diabetic wound exudate by iTRAQ is available on Supplementary Table 5; IPA technology for the annotation of differential proteins is available on Supplementary Table 6; classical signal pathway analysis of differential proteins is available on Supplementary Table 7; analysis of upstream regulatory factors is available on Supplementary Table 8; analysis of possible interaction networks in differential proteins is available on Supplementary Table 9.

## References

[1] Armstrong D. G., Boulton A. J. M., Bus S. A. (2017). Diabetic foot ulcers and their recurrence. *The New England Journal of Medicine*.

[2] Skrepnek G. H., Mills J. L., Lavery L. A., Armstrong D. G. (2017). Health care service and outcomes among an estimated 6.7 million ambulatory care diabetic foot cases in the U.S. *Diabetes Care*.

[3] Abbott C. A., Carrington A. L., Ashe H. (2002). The north-west diabetes foot care study: incidence of, and risk factors for, new diabetic foot ulceration in a community-based patient cohort. *Diabetic Medicine*.

[4] Vinik A. I., Nevoret M. L., Casellini C., Parson H. (2013). Diabetic neuropathy. *Endocrinology and Metabolism Clinics of North America*.

[5] Murray H. J., Young M. J., Hollis S., Boulton A. J. (1996). The association between callus formation, high pressures and neuropathy in diabetic foot ulceration. *Diabetic Medicine*.

[6] Zochodne D. W. (2008). Diabetic polyneuropathy: an update. *Current Opinion in Neurology*.

[7] Wattanakit K., Folsom A. R., Selvin E. (2005). Risk factors for peripheral arterial disease incidence in persons with diabetes: the atherosclerosis risk in communities (ARIC) study. *Atherosclerosis*.

[8] Amendt C., Mann A., Schirmacher P., Blessing M. (2002). Resistance of keratinocytes to TGFbeta-mediated growth restriction and apoptosis induction accelerates re-epithelialization in skin wounds. *Journal of Cell Science*.

[9] Asplin I. R., Wu S. M., Mathew S., Bhattacharjee G., Pizzo S. V. (2001). Differential regulation of the fibroblast growth factor (FGF) family by alpha(2)-macroglobulin: evidence for selective modulation of FGF-2-induced angiogenesis. *Blood*.

[10] Burgoyne R. D., Morgan A. (2003). Secretory granule exocytosis. *Physiological Reviews*.

[11] Yazdanpanah S., Rabiee M., Tahriri M. (2017). Evaluation of glycated albumin (GA) and GA/HbA1c ratio for diagnosis of diabetes and glycemic control: a comprehensive review. *Critical Reviews in Clinical Laboratory Sciences*.

[12] Zhao Y., Wang X., Yang S. (2020). Kanglexin accelerates diabetic wound healing by promoting angiogenesis via FGFR1/ERK signaling. *Biomedicine & Pharmacotherapy*.

[13] Hu M., Wu Y., Yang C. (2020). Novel long noncoding RNA lnc-URIDS delays diabetic wound healing by targeting Plod1. *Diabetes*.

[14] Sawaya A. P., Stone R. C., Brooks S. R. (2020). Deregulated immune cell recruitment orchestrated by FOXM1 impairs human diabetic wound healing. *Nature Communications*.

[15] Fonder M. A., Lazarus G. S., Cowan D. A., Aronson-Cook B., Kohli A. R., Mamelak A. J. (2008). Treating the chronic wound: a practical approach to the care of nonhealing wounds and wound care dressings. *Journal of the American Academy of Dermatology*.

[16] Lauer G., Sollberg S., Cole M. (2000). Expression and proteolysis of vascular endothelial growth factor is increased in chronic wounds. *The Journal of Investigative Dermatology*.

[17] Moor A. N., Vachon D. J., Gould L. J. (2009). Proteolytic activity in wound fluids and tissues derived from chronic venous leg ulcers. *Wound Repair and Regeneration*.

[18] Wilkins M. R., Sanchez J. C., Gooley A. A. (1996). Progress with proteome projects: why all proteins expressed by a genome should be identified and how to do it. *Biotechnology & Genetic Engineering Reviews*.

[19] Rappsilber J., Mann M. (2002). What does it mean to identify a protein in proteomics?. *Trends in Biochemical Sciences*.

[20] Pandey A., Mann M. (2000). Proteomics to study genes and genomes. *Nature*.

[21] Licker V., Kovari E., Hochstrasser D. F., Burkhard P. R. (2009). Proteomics in human Parkinson's disease research. *Journal of Proteomics*.

[22] Kong F., Nicole White C., Xiao X. (2006). Using proteomic approaches to identify new biomarkers for detection and monitoring of ovarian cancer. *Gynecologic Oncology*.

[23] Eming S. A., Koch M., Krieger A. (2010). Differential proteomic analysis distinguishes tissue repair biomarker signatures in wound exudates obtained from normal healing and chronic wounds. *Journal of Proteome Research*.

[24] Fernandez M. L., Broadbent J. A., Shooter G. K., Malda J., Upton Z. (2008). Development of an enhanced proteomic method to detect prognostic and diagnostic markers of healing in chronic wound fluid. *The British Journal of Dermatology*.

[25] Smith E., Hoffman R. (2005). Multiple fragments related to angiostatin and endostatin in fluid from venous leg ulcers. *Wound Repair and Regeneration*.

[26] Krisp C., Jacobsen F., McKay M. J., Molloy M. P., Steinstraesser L., Wolters D. A. (2013). Proteome analysis reveals antiangiogenic environments in chronic wounds of diabetes mellitus type 2 patients. *Proteomics*.

[27] Zhang L., Shi W., Zeng X. C. (2015). Unique diversity of the venom peptides from the scorpion Androctonus bicolor revealed by transcriptomic and proteomic analysis. *Journal of Proteomics*.

[28] Kinoshita-Kikuta E., Kinoshita E., Matsuda A., Koike T. (2014). Tips on improving the efficiency of electrotransfer of target proteins from Phos-tag SDS-PAGE gel. *Proteomics*.

[29] Faca V. M., Song K. S., Wang H. (2008). A mouse to human search for plasma proteome changes associated with pancreatic tumor development. *PLoS Medicine*.

[30] Yu H., Wang X., Xu J. (2017). iTRAQ-based quantitative proteomics analysis of molecular mechanisms associated with Bombyx mori (Lepidoptera) larval midgut response to BmNPV in susceptible and near-isogenic strains. *Journal of Proteomics*.

[31] Wang T., Chen H., Lv K. (2017). iTRAQ-based proteomics analysis of hippocampus in spatial memory deficiency rats induced by simulated microgravity. *Journal of Proteomics*.

[32] Song C., Ye M., Han G. (2010). Reversed-phase-reversed-phase liquid chromatography approach with high orthogonality for multidimensional separation of phosphopeptides. *Analytical Chemistry*.

[33] Dowell J. A., Frost D. C., Zhang J., Li L. (2008). Comparison of two-dimensional fractionation techniques for shotgun proteomics. *Analytical Chemistry*.

[34] Williams T. L., Leopold P., Musser S. (2002). Automated postprocessing of electrospray LC/MS data for profiling protein expression in bacteria. *Analytical Chemistry*.

[35] Wang Y., Balgley B. M., Rudnick P. A., Lee C. S. (2005). Effects of chromatography conditions on intact protein separations for top-down proteomics. *Journal of Chromatography A*.

[36] Perkins D. N., Pappin D. J., Creasy D. M., Cottrell J. S. (1999). Probability-based protein identification by searching sequence databases using mass spectrometry data. *Electrophoresis*.

[37] Song J. E., Kwak Y. G., Um T. H. (2018). Outbreak of Burkholderia cepacia pseudobacteraemia caused by intrinsically contaminated commercial 0.5% chlorhexidine solution in neonatal intensive care units. *The Journal of Hospital Infection*.

[38] Nakamizo S., Egawa G., Honda T., Nakajima S., Belkaid Y., Kabashima K. (2015). Commensal bacteria and cutaneous immunity. *Seminars in Immunopathology*.

[39] Bouchal P., Roumeliotis T., Hrstka R., Nenutil R., Vojtesek B., Garbis S. D. (2009). Biomarker discovery in low-grade breast cancer using isobaric stable isotope tags and two-dimensional liquid chromatography-tandem mass spectrometry (iTRAQ-2DLC-MS/MS) based quantitative proteomic analysis. *Journal of Proteome Research*.

[40] Muller M., Trocme C., Morel F., Halimi S., Benhamou P. Y. (2009). Increased Matrix Metalloproteinase-9 Predicts Poor Wound Healing in Diabetic Foot Ulcers. *Diabetes Care*.

[41] Muller M., Trocme C., Lardy B., Morel F., Halimi S., Benhamou P. Y. (2008). Matrix metalloproteinases and diabetic foot ulcers: the ratio of MMP-1 to TIMP-1 is a predictor of wound healing. *Diabetic Medicine*.

