# Supplementary material for: Proteomic Analysis of Exudates from Chronic Ulcer of Diabetic Foot Treated with Scorpion Antimicrobial Peptide
Source: Mediators Inflamm. 2022 Oct 3;2022:5852786. doi: 10.1155/2022/5852786 (PMC9550419; doi:10.1155/2022/5852786)
Supplement: Supplementary Materials — Bacteriological identification of diabetic foot ulcer wounds is available on Supplementary Table 1–3. Identification results by mass spectrometry is available on Supplementary Table 4; analysis of proteins in diabetic wound exudate by iTRAQ is available on Supplementary Table 5; IPA technology for the annotation of differential proteins is available on Supplementary Table 6; classical signal pathway analysis of differential proteins is available on Supplementary Table 7; analysis of upstream regulatory factors is available on Supplementary Table 8; analysis of possible interaction networks in differential proteins is available on Supplementary Table 9. [file 5852786.f1.zip › Supplementary Table 2.docx]

Supplementary Table 2 Bacteriological identification of diabetic foot ulcer wounds (middle stage)

| Rank | Name | Strain | Authors | Taxonomy | Accession | Pairwise Similarity (%) | Diff/Total nt | Completeness (%) |
| --- | --- | --- | --- | --- | --- | --- | --- | --- |
| 1 | Burkholderia cepacia | ATCC 25416(T) | (Palleroni and Holmes 1981) Yabuuchi et al. 1993 | Bacteria; Proteobacteria; Betaproteobacteria; Burkholderiales; Burkholderiaceae; Burkholderia; Burkholderia cepacia | AXBO01000009 | 99.3 | 10/1425 | 100 |
| 2 | Burkholderia anthina | R-4183(T) | Vandamme et al. 2002 | Bacteria; Proteobacteria; Betaproteobacteria; Burkholderiales; Burkholderiaceae; Burkholderia; Burkholderia anthina | AJ420880 | 99.3 | 10/1425 | 100 |
| 3 | Burkholderia seminalis | R-24196(T) | Vanlaere et al. 2008 | Bacteria; Proteobacteria; Betaproteobacteria; Burkholderiales; Burkholderiaceae; Burkholderia; Burkholderia seminalis | AM747631 | 99.3 | 10/1422 | 100 |
| 4 | Burkholderia pyrrocinia | LMG 14191(T) | (Imanaka et al. 1965) Vandamme et al. 1997 | Bacteria; Proteobacteria; Betaproteobacteria; Burkholderiales; Burkholderiaceae; Burkholderia; Burkholderia pyrrocinia | U96930 | 99.28 | 10/1398 | 97.62 |
| 5 | Burkholderia cenocepacia | J2315(T) | Vandamme et al. 2003 | Bacteria; Proteobacteria; Betaproteobacteria; Burkholderiales; Burkholderiaceae; Burkholderia; Burkholderia cenocepacia | AM747720 | 99.23 | 11/1425 | 100 |
| 6 | Burkholderia ubonensis | CIP 107078(T) | Yabuuchi et al. 2000 | Bacteria; Proteobacteria; Betaproteobacteria; Burkholderiales; Burkholderiaceae; Burkholderia; Burkholderia ubonensis | EU024179 | 99.16 | 11/1313 | 90.31 |
| 7 | Burkholderia metallica | R-16017(T) | Vanlaere et al. 2008 | Bacteria; Proteobacteria; Betaproteobacteria; Burkholderiales; Burkholderiaceae; Burkholderia; Burkholderia metallica | AM747632 | 99.16 | 12/1425 | 100 |
| 8 | Burkholderia arboris | R-24201(T) | Vanlaere et al. 2008 | Bacteria; Proteobacteria; Betaproteobacteria; Burkholderiales; Burkholderiaceae; Burkholderia; Burkholderia arboris | AM747630 | 99.16 | 12/1424 | 100 |
| 9 | Burkholderia latens | R-5630(T) | Vanlaere et al. 2008 | Bacteria; Proteobacteria; Betaproteobacteria; Burkholderiales; Burkholderiaceae; Burkholderia; Burkholderia latens | AM747628 | 99.02 | 14/1425 | 100 |
| 10 | Burkholderia ambifaria | AMMD(T) | Coenye et al. 2001 | Bacteria; Proteobacteria; Betaproteobacteria; Burkholderiales; Burkholderiaceae; Burkholderia; Burkholderia ambifaria | CP000442 | 99.02 | 14/1425 | 100 |
| 11 | Burkholderia diffusa | R-15930(T) | Vanlaere et al. 2008 | Bacteria; Proteobacteria; Betaproteobacteria; Burkholderiales; Burkholderiaceae; Burkholderia; Burkholderia diffusa | AM747629 | 99.02 | 14/1425 | 100 |
| 12 | Burkholderia lata | 383(T) | Vanlaere et al. 2009 | Bacteria; Proteobacteria; Betaproteobacteria; Burkholderiales; Burkholderiaceae; Burkholderia; Burkholderia lata | CP000150 | 98.95 | 15/1425 | 100 |
| 13 | Burkholderia vietnamiensis | LMG 10929(T) | Gillis et al. 1995 | Bacteria; Proteobacteria; Betaproteobacteria; Burkholderiales; Burkholderiaceae; Burkholderia; Burkholderia vietnamiensis | AF097534 | 98.95 | 15/1425 | 100 |
| 14 | Burkholderia stabilis | LMG 14294(T) | Vandamme et al. 2000 | Bacteria; Proteobacteria; Betaproteobacteria; Burkholderiales; Burkholderiaceae; Burkholderia; Burkholderia stabilis | AF148554 | 98.95 | 15/1423 | 100 |
| 15 | Burkholderia multivorans | ATCC BAA-247(T) | Vandamme et al. 1997 | Bacteria; Proteobacteria; Betaproteobacteria; Burkholderiales; Burkholderiaceae; Burkholderia; Burkholderia multivorans | ALIW01000278 | 98.74 | 18/1425 | 100 |
| 16 | Burkholderia dolosa | LMG 18943(T) | Vermis et al. 2004 | Bacteria; Proteobacteria; Betaproteobacteria; Burkholderiales; Burkholderiaceae; Burkholderia; Burkholderia dolosa | JX986970 | 98.66 | 19/1423 | 100 |
| 17 | Burkholderia glumae | LMG 2196(T) | (Kurita and Tabei 1967) Urakami et al. 1994 | Bacteria; Proteobacteria; Betaproteobacteria; Burkholderiales; Burkholderiaceae; Burkholderia; Burkholderia glumae | AMRF01000003 | 98.18 | 26/1425 | 100 |
| 18 | Burkholderia pseudomultivorans | LMG 26883(T) | Peeters et al. 2014 | Bacteria; Proteobacteria; Betaproteobacteria; Burkholderiales; Burkholderiaceae; Burkholderia; Burkholderia pseudomultivorans | HE962386 | 98.17 | 26/1423 | 100 |
| 19 | Burkholderia plantarii | LMG 9035(T) | (Azegami et al. 1987) Urakami et al. 1994 | Bacteria; Proteobacteria; Betaproteobacteria; Burkholderiales; Burkholderiaceae; Burkholderia; Burkholderia plantarii | U96933 | 98.09 | 27/1411 | 98.44 |
| 20 | Burkholderia oklahomensis | C6786(T) | Glass et al. 2006 | Bacteria; Proteobacteria; Betaproteobacteria; Burkholderiales; Burkholderiaceae; Burkholderia; Burkholderia oklahomensis | ABBG01000575 | 98.04 | 28/1425 | 100 |
| 21 | Burkholderia gladioli | CIP 105410(T) | (Severini 1913) Yabuuchi et al. 1993 | Bacteria; Proteobacteria; Betaproteobacteria; Burkholderiales; Burkholderiaceae; Burkholderia; Burkholderia gladioli | EU024168 | 97.94 | 27/1313 | 90.31 |
| 22 | Burkholderia thailandensis | E264(T) | Brett et al. 1998 | Bacteria; Proteobacteria; Betaproteobacteria; Burkholderiales; Burkholderiaceae; Burkholderia; Burkholderia thailandensis | CP000086 | 97.75 | 32/1423 | 100 |
| 23 | Burkholderia pseudomallei | ATCC 23343(T) | (Whitmore 1913) Yabuuchi et al. 1993 | Bacteria; Proteobacteria; Betaproteobacteria; Burkholderiales; Burkholderiaceae; Burkholderia; Burkholderia pseudomallei | DQ108392 | 97.75 | 32/1422 | 100 |
| 24 | Burkholderia rinojensis | A396(T) | Cordova-Kreylos et al. 2013 | Bacteria; Proteobacteria; Betaproteobacteria; Burkholderiales; Burkholderiaceae; Burkholderia; Burkholderia rinojensis | KF650996 | 97.68 | 33/1425 | 100 |
| 25 | Burkholderia mallei | ATCC 23344(T) | (Zopf 1885) Yabuuchi et al. 1993 | Bacteria; Proteobacteria; Betaproteobacteria; Burkholderiales; Burkholderiaceae; Burkholderia; Burkholderia mallei | CP000011 | 97.61 | 34/1423 | 100 |
| 26 | Burkholderia caryophylli | ATCC 25418(T) | (Burkholder 1942) Yabuuchi et al. 1993 | Bacteria; Proteobacteria; Betaproteobacteria; Burkholderiales; Burkholderiaceae; Burkholderia; Burkholderia caryophylli | AB021423 | 96.67 | 47/1411 | 98.57 |
| 27 | Burkholderia dabaoshanensis | GIMN1.004(T) | Zhu et al. 2012 | Bacteria; Proteobacteria; Betaproteobacteria; Burkholderiales; Burkholderiaceae; Burkholderia; Burkholderia dabaoshanensis | FJ210816 | 96.52 | 49/1409 | 98.42 |
| 28 | Burkholderia terrestris | LMG 22937(T) | Vandamme et al. 2013 | Bacteria; Proteobacteria; Betaproteobacteria; Burkholderiales; Burkholderiaceae; Burkholderia; Burkholderia terrestris | HE981726 | 96.42 | 51/1423 | 100 |
| 29 | Burkholderia sordidicola | S5-B(T) | Lim et al. 2003 | Bacteria; Proteobacteria; Betaproteobacteria; Burkholderiales; Burkholderiaceae; Burkholderia; Burkholderia sordidicola | AF512826 | 96.35 | 52/1423 | 99.93 |
| 30 | Burkholderia symbiotica | JPY 345(T) | Sheu et al. 2012 | Bacteria; Proteobacteria; Betaproteobacteria; Burkholderiales; Burkholderiaceae; Burkholderia; Burkholderia symbiotica | HM357233 | 96.08 | 54/1376 | 96.1 |
| 31 | Burkholderia phenazinium | LMG 2247(T) | (Bell and Turner 1973) Viallard et al. 1998 | Bacteria; Proteobacteria; Betaproteobacteria; Burkholderiales; Burkholderiaceae; Burkholderia; Burkholderia phenazinium | U96936 | 96.07 | 55/1401 | 97.82 |
| 32 | Burkholderia soli | GP25-8(T) | Yoo et al. 2007 | Bacteria; Proteobacteria; Betaproteobacteria; Burkholderiales; Burkholderiaceae; Burkholderia; Burkholderia soli | DQ465451 | 96.06 | 56/1423 | 100 |
| 33 | Burkholderia sediminicola | HU2-65W(T) | Lim et al. 2008 | Bacteria; Proteobacteria; Betaproteobacteria; Burkholderiales; Burkholderiaceae; Burkholderia; Burkholderia sediminicola | EU035613 | 96.06 | 56/1423 | 100 |
| 34 | Burkholderia rhizoxinica | HKI 454(T) | Partida-Martinez et al. 2007 | Bacteria; Proteobacteria; Betaproteobacteria; Burkholderiales; Burkholderiaceae; Burkholderia; Burkholderia rhizoxinica | FR687359 | 95.92 | 58/1423 | 100 |
| 35 | Burkholderia unamae | MTl-641(T) | Caballero-Mellado et al. 2004 | Bacteria; Proteobacteria; Betaproteobacteria; Burkholderiales; Burkholderiaceae; Burkholderia; Burkholderia unamae | AY221956 | 95.86 | 59/1425 | 100 |
| 36 | Burkholderia endofungorum | HKI 456(T) | Partida-Martinez et al. 2007 | Bacteria; Proteobacteria; Betaproteobacteria; Burkholderiales; Burkholderiaceae; Burkholderia; Burkholderia endofungorum | AM420302 | 95.64 | 62/1423 | 100 |
| 37 | Pandoraea faecigallinarum | KOx(T) | Sahin et al. 2011 | Bacteria; Proteobacteria; Betaproteobacteria; Burkholderiales; Burkholderiaceae; Pandoraea; Pandoraea faecigallinarum | AB510956 | 95.59 | 60/1362 | 94.32 |
| 38 | Burkholderia tropica | Ppe8(T) | Reis et al. 2004 | Bacteria; Proteobacteria; Betaproteobacteria; Burkholderiales; Burkholderiaceae; Burkholderia; Burkholderia tropica | AJ420332 | 95.57 | 62/1399 | 96.63 |
| 39 | Burkholderia sacchari | IPT101(T) | Brämer et al. 2001 | Bacteria; Proteobacteria; Betaproteobacteria; Burkholderiales; Burkholderiaceae; Burkholderia; Burkholderia sacchari | AF263278 | 95.3 | 67/1425 | 100 |
| 40 | Burkholderia oxyphila | NBRC 105797(T) | Otsuka et al. 2011 | Bacteria; Proteobacteria; Betaproteobacteria; Burkholderiales; Burkholderiaceae; Burkholderia; Burkholderia oxyphila | BAYD01000210 | 95.02 | 71/1425 | 100 |
| 41 | Burkholderia eburnea | RR11(T) | Kang et al. 2014 | Bacteria; Proteobacteria; Betaproteobacteria; Burkholderiales; Burkholderiaceae; Burkholderia; Burkholderia eburnea | JQ692176 | 94.94 | 72/1423 | 99.79 |
