# Supplementary material for: Proteomic Analysis of Exudates from Chronic Ulcer of Diabetic Foot Treated with Scorpion Antimicrobial Peptide
Source: Mediators Inflamm. 2022 Oct 3;2022:5852786. doi: 10.1155/2022/5852786 (PMC9550419; doi:10.1155/2022/5852786)
Supplement: Supplementary Materials — Bacteriological identification of diabetic foot ulcer wounds is available on Supplementary Table 1–3. Identification results by mass spectrometry is available on Supplementary Table 4; analysis of proteins in diabetic wound exudate by iTRAQ is available on Supplementary Table 5; IPA technology for the annotation of differential proteins is available on Supplementary Table 6; classical signal pathway analysis of differential proteins is available on Supplementary Table 7; analysis of upstream regulatory factors is available on Supplementary Table 8; analysis of possible interaction networks in differential proteins is available on Supplementary Table 9. [file 5852786.f1.zip › Supplementary Table 3.docx]

Supplementary Table 3 Bacteriological identification of diabetic foot ulcer wounds (late stage)

| Rank | Name | Strain | Authors | Taxonomy | Accession | Pairwise Similarity (%) | Diff/Total nt | Completeness (%) |
| --- | --- | --- | --- | --- | --- | --- | --- | --- |
| 1 | Staphylococcus epidermidis | ATCC 14990(T) | (Winslow and Winslow 1908) Evans 1916 | Bacteria; Firmicutes; Bacilli; Bacillales; Staphylococcaceae; Staphylococcus; Staphylococcus epidermidis | L37605 | 99.59 | 6/1450 | 99.66 |
| 2 | Staphylococcus caprae | ATCC 35538(T) | Devriese et al. 1983 | Bacteria; Firmicutes; Bacilli; Bacillales; Staphylococcaceae; Staphylococcus; Staphylococcus caprae | AB009935 | 99.17 | 12/1450 | 99.93 |
| 3 | Staphylococcus capitis subsp. capitis | ATCC 27840(T) | Kloos and Schleifer 1975 | Bacteria; Firmicutes; Bacilli; Bacillales; Staphylococcaceae; Staphylococcus; Staphylococcus capitis; Staphylococcus capitis subsp. capitis | L37599 | 99.17 | 12/1449 | 99.73 |
| 4 | Staphylococcus saccharolyticus | ATCC 14953(T) | (Foubert and Douglas 1948) Kilpper-Bälz and Schleifer 1984 | Bacteria; Firmicutes; Bacilli; Bacillales; Staphylococcaceae; Staphylococcus; Staphylococcus saccharolyticus | L37602 | 99.17 | 12/1448 | 100 |
| 5 | Staphylococcus capitis subsp. urealyticus | GTC 727(T) | Bannerman and Kloos 1991 | Bacteria; Firmicutes; Bacilli; Bacillales; Staphylococcaceae; Staphylococcus; Staphylococcus capitis; Staphylococcus capitis subsp. urealyticus | AB233325 | 98.96 | 15/1448 | 98.98 |
| 6 | Staphylococcus pasteuri | ATCC 51129(T) | Chesneau et al. 1993 | Bacteria; Firmicutes; Bacilli; Bacillales; Staphylococcaceae; Staphylococcus; Staphylococcus pasteuri | AF041361 | 98.59 | 20/1417 | 96.2 |
| 7 | Staphylococcus jettensis | SEQ110(T) | De Bel et al. 2013 | Bacteria; Firmicutes; Bacilli; Bacillales; Staphylococcaceae; Staphylococcus; Staphylococcus jettensis | JN092118 | 98.45 | 22/1422 | 97.5 |
| 8 | Staphylococcus petrasii subsp. petrasii | CCM8418(T) | Pantucek et al. 2013 | Bacteria; Firmicutes; Bacilli; Bacillales; Staphylococcaceae; Staphylococcus; Staphylococcus petrasii; Staphylococcus petrasii subsp. petrasii | JX139845 | 98.41 | 23/1450 | 100 |
| 9 | Staphylococcus warneri | ATCC 27836(T) | Kloos and Schleifer 1975 | Bacteria; Firmicutes; Bacilli; Bacillales; Staphylococcaceae; Staphylococcus; Staphylococcus warneri | L37603 | 98.41 | 23/1449 | 99.66 |
| 10 | Staphylococcus simiae | CCM 7213(T) | Pantucek et al. 2005 | Bacteria; Firmicutes; Bacilli; Bacillales; Staphylococcaceae; Staphylococcus; Staphylococcus simiae | AY727530 | 98.28 | 25/1450 | 100 |
| 11 | Staphylococcus haemolyticus | ATCC 29970(T) | Schleifer and Kloos 1975 | Bacteria; Firmicutes; Bacilli; Bacillales; Staphylococcaceae; Staphylococcus; Staphylococcus haemolyticus | L37600 | 98.21 | 26/1450 | 99.66 |
| 12 | Staphylococcus aureus subsp. anaerobius | ATCC 35844 | De La Fuente et al. 1985 | Bacteria; Firmicutes; Bacilli; Bacillales; Staphylococcaceae; Staphylococcus; Staphylococcus aureus; Staphylococcus aureus subsp. anaerobius | D83355 | 98.21 | 26/1450 | 100 |
| 13 | Staphylococcus devriesei | LMG 25332(T) | Supré et al. 2010 | Bacteria; Firmicutes; Bacilli; Bacillales; Staphylococcaceae; Staphylococcus; Staphylococcus devriesei | FJ389206 | 98.21 | 26/1450 | 100 |
| 14 | Staphylococcus hominis subsp. hominis | DSM 20328(T) | Kloos and Schleifer 1975 | Bacteria; Firmicutes; Bacilli; Bacillales; Staphylococcaceae; Staphylococcus; Staphylococcus hominis; Staphylococcus hominis subsp. hominis | X66101 | 98.2 | 26/1448 | 100 |
| 15 | Staphylococcus lugdunensis | ATCC 43809(T) | Freney et al. 1988 | Bacteria; Firmicutes; Bacilli; Bacillales; Staphylococcaceae; Staphylococcus; Staphylococcus lugdunensis | AB009941 | 98.14 | 27/1450 | 99.93 |
| 16 | Staphylococcus aureus subsp. aureus | DSM 20231(T) | Rosenbach 1884 | Bacteria; Firmicutes; Bacilli; Bacillales; Staphylococcaceae; Staphylococcus; Staphylococcus aureus; Staphylococcus aureus subsp. aureus | AMYL01000007 | 98.14 | 27/1450 | 100 |
| 17 | Staphylococcus petrasii subsp. croceilyticus | CCM8421(T) | Pantucek et al. 2013 | Bacteria; Firmicutes; Bacilli; Bacillales; Staphylococcaceae; Staphylococcus; Staphylococcus petrasii; Staphylococcus petrasii subsp. croceilyticus | AY953148 | 98.14 | 27/1449 | 100 |
| 18 | Staphylococcus hominis subsp. novobiosepticus | GTC 1228(T) | Kloos et al. 1998 | Bacteria; Firmicutes; Bacilli; Bacillales; Staphylococcaceae; Staphylococcus; Staphylococcus hominis; Staphylococcus hominis subsp. novobiosepticus | AB233326 | 98.14 | 27/1448 | 98.85 |
| 19 | Staphylococcus muscae | DSM 7068(T) | Hájek et al. 1992 | Bacteria; Firmicutes; Bacilli; Bacillales; Staphylococcaceae; Staphylococcus; Staphylococcus muscae | FR733703 | 97.66 | 34/1450 | 100 |
| 20 | Staphylococcus cohnii subsp. urealyticus | ATCC 49330(T) | Kloos and Wolfshohl 1991 | Bacteria; Firmicutes; Bacilli; Bacillales; Staphylococcaceae; Staphylococcus; Staphylococcus cohnii; Staphylococcus cohnii subsp. urealyticus | AB009936 | 97.52 | 36/1450 | 99.93 |
| 21 | Staphylococcus piscifermentans | ATCC 51136(T) | Tanasupawat et al. 1992 | Bacteria; Firmicutes; Bacilli; Bacillales; Staphylococcaceae; Staphylococcus; Staphylococcus piscifermentans | AB009943 | 97.45 | 37/1450 | 99.93 |
| 22 | Staphylococcus cohnii subsp. cohnii | ATCC 29974(T) | Schleifer and Kloos 1975 | Bacteria; Firmicutes; Bacilli; Bacillales; Staphylococcaceae; Staphylococcus; Staphylococcus cohnii; Staphylococcus cohnii subsp. cohnii | D83361 | 97.45 | 37/1450 | 100 |
| 23 | Staphylococcus saprophyticus subsp. saprophyticus | ATCC 15305(T) | (Fairbrother 1940) Shaw et al. 1951 | Bacteria; Firmicutes; Bacilli; Bacillales; Staphylococcaceae; Staphylococcus; Staphylococcus saprophyticus; Staphylococcus saprophyticus subsp. saprophyticus | AP008934 | 97.45 | 37/1450 | 100 |
| 24 | Staphylococcus pettenkoferi | B3117(T) | Trülzsch et al. 2007 | Bacteria; Firmicutes; Bacilli; Bacillales; Staphylococcaceae; Staphylococcus; Staphylococcus pettenkoferi | AF322002 | 97.38 | 38/1450 | 100 |
| 25 | Staphylococcus xylosus | ATCC 29971(T) | Schleifer and Kloos 1975 | Bacteria; Firmicutes; Bacilli; Bacillales; Staphylococcaceae; Staphylococcus; Staphylococcus xylosus | D83374 | 97.31 | 39/1450 | 100 |
| 26 | Staphylococcus gallinarum | ATCC 35539(T) | Devriese et al. 1983 | Bacteria; Firmicutes; Bacilli; Bacillales; Staphylococcaceae; Staphylococcus; Staphylococcus gallinarum | D83366 | 97.31 | 39/1450 | 100 |
| 27 | Staphylococcus microti | CCM 4903(T) | Nováková et al. 2010 | Bacteria; Firmicutes; Bacilli; Bacillales; Staphylococcaceae; Staphylococcus; Staphylococcus microti | EU888120 | 97.31 | 39/1450 | 100 |
| 28 | Staphylococcus rostri | ARI 262(T) | Riesen and Perreten 2010 | Bacteria; Firmicutes; Bacilli; Bacillales; Staphylococcaceae; Staphylococcus; Staphylococcus rostri | FM242137 | 97.25 | 38/1382 | 94.64 |
| 29 | Staphylococcus carnosus subsp. carnosus | ATCC 51365(T) | Schleifer and Fischer 1982 | Bacteria; Firmicutes; Bacilli; Bacillales; Staphylococcaceae; Staphylococcus; Staphylococcus carnosus; Staphylococcus carnosus subsp. carnosus | AB009934 | 97.24 | 40/1450 | 99.93 |
| 30 | Staphylococcus chromogenes | ATCC 43764(T) | (Devriese et al. 1978) Hajek et al. 1987 | Bacteria; Firmicutes; Bacilli; Bacillales; Staphylococcaceae; Staphylococcus; Staphylococcus chromogenes | D83360 | 97.24 | 40/1450 | 100 |
| 31 | Staphylococcus condimenti | DSM 11674(T) | Probst et al. 1998 | Bacteria; Firmicutes; Bacilli; Bacillales; Staphylococcaceae; Staphylococcus; Staphylococcus condimenti | Y15750 | 97.17 | 41/1450 | 100 |
| 32 | Staphylococcus succinus subsp. succinus | AMG-D1(T) | Lambert et al. 1998 | Bacteria; Firmicutes; Bacilli; Bacillales; Staphylococcaceae; Staphylococcus; Staphylococcus succinus; Staphylococcus succinus subsp. succinus | AF004220 | 97.17 | 41/1450 | 100 |
| 33 | Staphylococcus saprophyticus subsp. bovis | GTC 843(T) | Hájek et al. 1996 | Bacteria; Firmicutes; Bacilli; Bacillales; Staphylococcaceae; Staphylococcus; Staphylococcus saprophyticus; Staphylococcus saprophyticus subsp. bovis | AB233327 | 97.17 | 41/1447 | 98.71 |
| 34 | Staphylococcus hyicus | ATCC 11249(T) | (Sompolinski 1953) Devriese et al. 1978 | Bacteria; Firmicutes; Bacilli; Bacillales; Staphylococcaceae; Staphylococcus; Staphylococcus hyicus | D83368 | 97.1 | 42/1450 | 100 |
| 35 | Staphylococcus felis | ATCC 49168(T) | Igimi et al. 1989 | Bacteria; Firmicutes; Bacilli; Bacillales; Staphylococcaceae; Staphylococcus; Staphylococcus felis | D83364 | 97.1 | 42/1450 | 100 |
| 36 | Staphylococcus kloosii | ATCC 43959(T) | Schleifer et al. 1985 | Bacteria; Firmicutes; Bacilli; Bacillales; Staphylococcaceae; Staphylococcus; Staphylococcus kloosii | AB009940 | 97.1 | 42/1450 | 99.93 |
| 37 | Staphylococcus succinus subsp. casei | SB72(T) | Place et al. 2003 | Bacteria; Firmicutes; Bacilli; Bacillales; Staphylococcaceae; Staphylococcus; Staphylococcus succinus; Staphylococcus succinus subsp. casei | AJ320272 | 97.03 | 43/1450 | 100 |
| 38 | Staphylococcus simulans | ATCC 27848(T) | Kloos and Schleifer 1975 | Bacteria; Firmicutes; Bacilli; Bacillales; Staphylococcaceae; Staphylococcus; Staphylococcus simulans | D83373 | 97.03 | 43/1450 | 100 |
| 39 | Staphylococcus schleiferi subsp. schleiferi | ATCC49545(T) | Freney et al. 1988 | Bacteria; Firmicutes; Bacilli; Bacillales; Staphylococcaceae; Staphylococcus; Staphylococcus schleiferi; Staphylococcus schleiferi subsp. schleiferi | AB009945 | 96.97 | 44/1450 | 99.93 |
| 40 | Staphylococcus auricularis | ATCC 33753(T) | Kloos and Schleifer 1983 | Bacteria; Firmicutes; Bacilli; Bacillales; Staphylococcaceae; Staphylococcus; Staphylococcus auricularis | L37598 | 96.96 | 44/1448 | 99.66 |
| 41 | Staphylococcus pseudintermedius | LMG 22219(T) | Devriese et al. 2005 | Bacteria; Firmicutes; Bacilli; Bacillales; Staphylococcaceae; Staphylococcus; Staphylococcus pseudintermedius | AJ780976 | 96.9 | 45/1450 | 100 |
| 42 | Staphylococcus equorum subsp. equorum | ATCC 43958(T) | Schleifer et al. 1985 | Bacteria; Firmicutes; Bacilli; Bacillales; Staphylococcaceae; Staphylococcus; Staphylococcus equorum; Staphylococcus equorum subsp. equorum | AB009939 | 96.83 | 46/1450 | 99.93 |
| 43 | Staphylococcus delphini | ATCC 49171(T) | Varaldo et al. 1988 | Bacteria; Firmicutes; Bacilli; Bacillales; Staphylococcaceae; Staphylococcus; Staphylococcus delphini | AB009938 | 96.83 | 46/1450 | 99.93 |
