# Supplementary material for: Proteomic Analysis of Exudates from Chronic Ulcer of Diabetic Foot Treated with Scorpion Antimicrobial Peptide
Source: Mediators Inflamm. 2022 Oct 3;2022:5852786. doi: 10.1155/2022/5852786 (PMC9550419; doi:10.1155/2022/5852786)
Supplement: Supplementary Materials — Bacteriological identification of diabetic foot ulcer wounds is available on Supplementary Table 1–3. Identification results by mass spectrometry is available on Supplementary Table 4; analysis of proteins in diabetic wound exudate by iTRAQ is available on Supplementary Table 5; IPA technology for the annotation of differential proteins is available on Supplementary Table 6; classical signal pathway analysis of differential proteins is available on Supplementary Table 7; analysis of upstream regulatory factors is available on Supplementary Table 8; analysis of possible interaction networks in differential proteins is available on Supplementary Table 9. [file 5852786.f1.zip › Supplementary Table 4.docx]

Supplementary Table 4 Statistics of mass spectrometry identification

| No. | Identified Proteins | Accession | Molecular Weight | Protein Grouping Ambiguity | Percent Coverage | Unique Peptide Count | Unique Spectrum Count | Total Spectrum Count |
| --- | --- | --- | --- | --- | --- | --- | --- | --- |
| 1 | Apolipoprotein B-100 OS=Homo sapiens GN=APOB PE=1 SV=2 | APOB_HUMAN | 516 kDa | TRUE | 72% | 299 | 711 | 2243 |
| 2 | Complement C3 OS=Homo sapiens GN=C3 PE=1 SV=2 | CO3_HUMAN | 187 kDa | TRUE | 86% | 141 | 375 | 2258 |
| 3 | Apolipoprotein A-I OS=Homo sapiens GN=APOA1 PE=1 SV=1 | APOA1_HUMAN | 31 kDa | TRUE | 86% | 41 | 107 | 1924 |
| 4 | Fibrinogen beta chain OS=Homo sapiens GN=FGB PE=1 SV=2 | FIBB_HUMAN | 56 kDa | TRUE | 74% | 43 | 134 | 1753 |
| 5 | Complement C4-B OS=Homo sapiens GN=C4B PE=1 SV=2 | CO4B_HUMAN | 193 kDa | TRUE | 77% | 113 | 274 | 1584 |
| 6 | Fibrinogen alpha chain OS=Homo sapiens GN=FGA PE=1 SV=2 | FIBA_HUMAN | 95 kDa | TRUE | 60% | 56 | 146 | 1401 |
| 7 | Fibronectin OS=Homo sapiens GN=FN1 PE=1 SV=4 | FINC_HUMAN | 263 kDa |  | 65% | 102 | 238 | 1098 |
| 8 | Serum albumin OS=Homo sapiens GN=ALB PE=1 SV=2 | ALBU_HUMAN | 69 kDa | TRUE | 81% | 57 | 149 | 896 |
| 9 | Fibrinogen gamma chain OS=Homo sapiens GN=FGG PE=1 SV=3 | FIBG_HUMAN | 52 kDa |  | 77% | 33 | 99 | 831 |
| 10 | Actin, cytoplasmic 1 OS=Homo sapiens GN=ACTB PE=1 SV=1 | ACTB_HUMAN | 42 kDa | TRUE | 89% | 29 | 89 | 841 |
| 11 | Hemoglobin subunit beta OS=Homo sapiens GN=HBB PE=1 SV=2 | HBB_HUMAN | 16 kDa | TRUE | 96% | 20 | 51 | 701 |
| 12 | Alpha-2-macroglobulin OS=Homo sapiens GN=A2M PE=1 SV=3 | A2MG_HUMAN | 163 kDa | TRUE | 71% | 79 | 197 | 600 |
| 13 | Ceruloplasmin OS=Homo sapiens GN=CP PE=1 SV=1 | CERU_HUMAN | 122 kDa | TRUE | 72% | 56 | 133 | 479 |
| 14 | Hemoglobin subunit alpha OS=Homo sapiens GN=HBA1 PE=1 SV=2 | HBA_HUMAN | 15 kDa | TRUE | 99% | 17 | 46 | 519 |
| 15 | Complement factor H OS=Homo sapiens GN=CFH PE=1 SV=4 | CFAH_HUMAN | 139 kDa | TRUE | 54% | 55 | 138 | 478 |
| 16 | Ig gamma-1 chain C region OS=Homo sapiens GN=IGHG1 PE=1 SV=1 | IGHG1_HUMAN | 36 kDa | TRUE | 70% | 19 | 47 | 492 |
| 17 | Apolipoprotein A-IV OS=Homo sapiens GN=APOA4 PE=1 SV=3 | APOA4_HUMAN | 45 kDa | TRUE | 83% | 37 | 89 | 426 |
| 18 | Ig mu chain C region OS=Homo sapiens GN=IGHM PE=1 SV=3 | IGHM_HUMAN | 49 kDa | TRUE | 60% | 27 | 71 | 473 |
| 19 | Lactotransferrin OS=Homo sapiens GN=LTF PE=1 SV=6 | TRFL_HUMAN | 78 kDa | TRUE | 75% | 54 | 116 | 455 |
| 20 | Myosin-9 OS=Homo sapiens GN=MYH9 PE=1 SV=4 | MYH9_HUMAN | 227 kDa | TRUE | 59% | 119 | 252 | 411 |
| 21 | Vitamin D-binding protein OS=Homo sapiens GN=GC PE=1 SV=1 | VTDB_HUMAN | 53 kDa | TRUE | 77% | 38 | 91 | 367 |
| 22 | Antithrombin-III OS=Homo sapiens GN=SERPINC1 PE=1 SV=1 | ANT3_HUMAN | 53 kDa | TRUE | 67% | 33 | 81 | 422 |
| 23 | Glyceraldehyde-3-phosphate dehydrogenase OS=Homo sapiens GN=GAPDH PE=1 SV=3 | G3P_HUMAN | 36 kDa | TRUE | 85% | 25 | 70 | 420 |
| 24 | Clusterin OS=Homo sapiens GN=CLU PE=1 SV=1 | CLUS_HUMAN | 52 kDa | TRUE | 52% | 22 | 55 | 367 |
| 25 | Pyruvate kinase PKM OS=Homo sapiens GN=PKM PE=1 SV=4 | KPYM_HUMAN | 58 kDa | TRUE | 81% | 46 | 111 | 385 |
| 26 | C4b-binding protein alpha chain OS=Homo sapiens GN=C4BPA PE=1 SV=2 | C4BPA_HUMAN | 67 kDa |  | 67% | 31 | 68 | 326 |
| 27 | Alpha-actinin-1 OS=Homo sapiens GN=ACTN1 PE=1 SV=2 | ACTN1_HUMAN | 103 kDa | TRUE | 74% | 60 | 154 | 369 |
| 28 | Gelsolin OS=Homo sapiens GN=GSN PE=1 SV=1 | GELS_HUMAN | 86 kDa | TRUE | 68% | 43 | 99 | 324 |
| 29 | Ig kappa chain C region OS=Homo sapiens GN=IGKC PE=1 SV=1 | IGKC_HUMAN | 12 kDa | TRUE | 91% | 9 | 21 | 359 |
| 30 | Alpha-1-antitrypsin OS=Homo sapiens GN=SERPINA1 PE=1 SV=3 | A1AT_HUMAN | 47 kDa | TRUE | 67% | 28 | 76 | 300 |
| 31 | Prothrombin OS=Homo sapiens GN=F2 PE=1 SV=2 | THRB_HUMAN | 70 kDa | TRUE | 63% | 37 | 94 | 294 |
| 32 | Talin-1 OS=Homo sapiens GN=TLN1 PE=1 SV=3 | TLN1_HUMAN | 270 kDa | TRUE | 62% | 121 | 212 | 314 |
| 33 | Inter-alpha-trypsin inhibitor heavy chain H4 OS=Homo sapiens GN=ITIH4 PE=1 SV=4 | ITIH4_HUMAN | 103 kDa | TRUE | 63% | 46 | 104 | 277 |
| 34 | Plasminogen OS=Homo sapiens GN=PLG PE=1 SV=2 | PLMN_HUMAN | 91 kDa | TRUE | 63% | 46 | 90 | 282 |
| 35 | Myeloperoxidase OS=Homo sapiens GN=MPO PE=1 SV=1 | PERM_HUMAN | 84 kDa | TRUE | 59% | 43 | 101 | 283 |
| 36 | Vitronectin OS=Homo sapiens GN=VTN PE=1 SV=1 | VTNC_HUMAN | 54 kDa | TRUE | 32% | 15 | 35 | 216 |
| 37 | Vimentin OS=Homo sapiens GN=VIM PE=1 SV=4 | VIME_HUMAN | 54 kDa | TRUE | 73% | 40 | 94 | 271 |
| 38 | Apolipoprotein E OS=Homo sapiens GN=APOE PE=1 SV=1 | APOE_HUMAN | 36 kDa |  | 79% | 30 | 61 | 232 |
| 39 | Ig lambda-2 chain C regions OS=Homo sapiens GN=IGLC2 PE=1 SV=1 | LAC2_HUMAN | 11 kDa | TRUE | 89% | 8 | 21 | 244 |
| 40 | Ras GTPase-activating-like protein IQGAP1 OS=Homo sapiens GN=IQGAP1 PE=1 SV=1 | IQGA1_HUMAN | 189 kDa | TRUE | 66% | 85 | 152 | 212 |
| 41 | Filamin-A OS=Homo sapiens GN=FLNA PE=1 SV=4 | FLNA_HUMAN | 281 kDa | TRUE | 51% | 94 | 162 | 213 |
| 42 | Apolipoprotein A-II OS=Homo sapiens GN=APOA2 PE=1 SV=1 | APOA2_HUMAN | 11 kDa |  | 69% | 8 | 24 | 195 |
| 43 | Complement C5 OS=Homo sapiens GN=C5 PE=1 SV=4 | CO5_HUMAN | 188 kDa | TRUE | 55% | 76 | 133 | 182 |
| 44 | Plectin OS=Homo sapiens GN=PLEC PE=1 SV=3 | PLEC_HUMAN | 532 kDa | TRUE | 31% | 128 | 155 | 184 |
| 45 | Spectrin alpha chain, non-erythrocytic 1 OS=Homo sapiens GN=SPTAN1 PE=1 SV=3 | SPTN1_HUMAN | 285 kDa | TRUE | 57% | 110 | 164 | 197 |
| 46 | Haptoglobin OS=Homo sapiens GN=HP PE=1 SV=1 | HPT_HUMAN | 45 kDa | TRUE | 64% | 27 | 54 | 160 |
| 47 | Histidine-rich glycoprotein OS=Homo sapiens GN=HRG PE=1 SV=1 | HRG_HUMAN | 60 kDa | TRUE | 42% | 21 | 47 | 199 |
| 48 | Heat shock protein HSP 90-beta OS=Homo sapiens GN=HSP90AB1 PE=1 SV=4 | HS90B_HUMAN | 83 kDa | TRUE | 68% | 43 | 82 | 183 |
| 49 | Ig alpha-1 chain C region OS=Homo sapiens GN=IGHA1 PE=1 SV=2 | IGHA1_HUMAN | 38 kDa | TRUE | 63% | 18 | 41 | 185 |
| 50 | Periostin OS=Homo sapiens GN=POSTN PE=1 SV=2 | POSTN_HUMAN | 93 kDa | TRUE | 64% | 47 | 96 | 161 |
| 51 | Transitional endoplasmic reticulum ATPase OS=Homo sapiens GN=VCP PE=1 SV=4 | TERA_HUMAN | 89 kDa | TRUE | 66% | 46 | 95 | 179 |
| 52 | Alpha-actinin-4 OS=Homo sapiens GN=ACTN4 PE=1 SV=2 | ACTN4_HUMAN | 105 kDa | TRUE | 74% | 42 | 90 | 299 |
| 53 | Alpha-2-HS-glycoprotein OS=Homo sapiens GN=AHSG PE=1 SV=1 | FETUA_HUMAN | 39 kDa | TRUE | 55% | 13 | 33 | 149 |
| 54 | Coronin-1A OS=Homo sapiens GN=CORO1A PE=1 SV=4 | COR1A_HUMAN | 51 kDa | TRUE | 61% | 28 | 62 | 149 |
| 55 | Protein S100-A8 OS=Homo sapiens GN=S100A8 PE=1 SV=1 | S10A8_HUMAN | 11 kDa | TRUE | 92% | 11 | 25 | 169 |
| 56 | Spectrin beta chain, non-erythrocytic 1 OS=Homo sapiens GN=SPTBN1 PE=1 SV=2 | SPTB2_HUMAN | 275 kDa | TRUE | 52% | 99 | 134 | 155 |
| 57 | Serum amyloid A-4 protein OS=Homo sapiens GN=SAA4 PE=1 SV=2 | SAA4_HUMAN | 15 kDa |  | 58% | 9 | 25 | 118 |
| 58 | Complement C1r subcomponent OS=Homo sapiens GN=C1R PE=1 SV=2 | C1R_HUMAN | 80 kDa | TRUE | 45% | 27 | 66 | 144 |
| 59 | Alpha-1-antichymotrypsin OS=Homo sapiens GN=SERPINA3 PE=1 SV=2 | AACT_HUMAN | 48 kDa |  | 56% | 22 | 53 | 151 |
| 60 | Elongation factor 2 OS=Homo sapiens GN=EEF2 PE=1 SV=4 | EF2_HUMAN | 95 kDa | TRUE | 54% | 41 | 78 | 109 |
| 61 | Histone H2B type 1-C/E/F/G/I OS=Homo sapiens GN=HIST1H2BC PE=1 SV=4 | H2B1C_HUMAN | 14 kDa | TRUE | 63% | 11 | 25 | 84 |
| 62 | Complement C1s subcomponent OS=Homo sapiens GN=C1S PE=1 SV=1 | C1S_HUMAN | 77 kDa | TRUE | 49% | 24 | 54 | 119 |
| 63 | 14-3-3 protein zeta/delta OS=Homo sapiens GN=YWHAZ PE=1 SV=1 | 1433Z_HUMAN | 28 kDa | TRUE | 74% | 15 | 39 | 145 |
| 64 | 6-phosphogluconate dehydrogenase, decarboxylating OS=Homo sapiens GN=PGD PE=1 SV=3 | 6PGD_HUMAN | 53 kDa |  | 60% | 25 | 52 | 123 |
| 65 | Serum amyloid A-1 protein OS=Homo sapiens GN=SAA1 PE=1 SV=1 | SAA1_HUMAN | 14 kDa | TRUE | 72% | 9 | 18 | 111 |
| 66 | Spectrin alpha chain, erythrocytic 1 OS=Homo sapiens GN=SPTA1 PE=1 SV=5 | SPTA1_HUMAN | 280 kDa | TRUE | 41% | 78 | 102 | 118 |
| 67 | Cytoplasmic dynein 1 heavy chain 1 OS=Homo sapiens GN=DYNC1H1 PE=1 SV=5 | DYHC1_HUMAN | 532 kDa | TRUE | 11% | 43 | 49 | 54 |
| 68 | Complement component C9 OS=Homo sapiens GN=C9 PE=1 SV=2 | CO9_HUMAN | 63 kDa | TRUE | 49% | 22 | 46 | 115 |
| 69 | Serotransferrin OS=Homo sapiens GN=TF PE=1 SV=3 | TRFE_HUMAN | 77 kDa | TRUE | 55% | 40 | 80 | 117 |
| 70 | Phosphoglycerate kinase 1 OS=Homo sapiens GN=PGK1 PE=1 SV=3 | PGK1_HUMAN | 45 kDa | TRUE | 74% | 29 | 65 | 100 |
| 71 | WD repeat-containing protein 1 OS=Homo sapiens GN=WDR1 PE=1 SV=4 | WDR1_HUMAN | 66 kDa |  | 62% | 30 | 59 | 119 |
| 72 | Transthyretin OS=Homo sapiens GN=TTR PE=1 SV=1 | TTHY_HUMAN | 16 kDa |  | 69% | 10 | 25 | 117 |
| 73 | Ig gamma-3 chain C region OS=Homo sapiens GN=IGHG3 PE=1 SV=2 | IGHG3_HUMAN | 41 kDa | TRUE | 62% | 9 | 23 | 308 |
| 74 | Apolipoprotein D OS=Homo sapiens GN=APOD PE=1 SV=1 | APOD_HUMAN | 21 kDa |  | 42% | 7 | 20 | 94 |
| 75 | Glycogen phosphorylase, liver form OS=Homo sapiens GN=PYGL PE=1 SV=4 | PYGL_HUMAN | 97 kDa | TRUE | 57% | 46 | 82 | 117 |
| 76 | Heat shock protein HSP 90-alpha OS=Homo sapiens GN=HSP90AA1 PE=1 SV=5 | HS90A_HUMAN | 85 kDa | TRUE | 61% | 26 | 54 | 217 |
| 77 | Cofilin-1 OS=Homo sapiens GN=CFL1 PE=1 SV=3 | COF1_HUMAN | 19 kDa | TRUE | 72% | 17 | 37 | 98 |
| 78 | Serum paraoxonase/arylesterase 1 OS=Homo sapiens GN=PON1 PE=1 SV=3 | PON1_HUMAN | 40 kDa |  | 66% | 14 | 36 | 98 |
| 79 | L-lactate dehydrogenase A chain OS=Homo sapiens GN=LDHA PE=1 SV=2 | LDHA_HUMAN | 37 kDa | TRUE | 71% | 23 | 50 | 94 |
| 80 | Tubulin alpha-1B chain OS=Homo sapiens GN=TUBA1B PE=1 SV=1 | TBA1B_HUMAN | 50 kDa | TRUE | 66% | 25 | 49 | 109 |
| 81 | Tubulin beta chain OS=Homo sapiens GN=TUBB PE=1 SV=2 | TBB5_HUMAN | 50 kDa | TRUE | 65% | 21 | 53 | 126 |
| 82 | Adenylyl cyclase-associated protein 1 OS=Homo sapiens GN=CAP1 PE=1 SV=5 | CAP1_HUMAN | 52 kDa |  | 59% | 25 | 56 | 94 |
| 83 | Glucose-6-phosphate 1-dehydrogenase OS=Homo sapiens GN=G6PD PE=1 SV=4 | G6PD_HUMAN | 59 kDa |  | 74% | 35 | 59 | 87 |
| 84 | Heparin cofactor 2 OS=Homo sapiens GN=SERPIND1 PE=1 SV=3 | HEP2_HUMAN | 57 kDa | TRUE | 57% | 23 | 51 | 85 |
| 85 | Vinculin OS=Homo sapiens GN=VCL PE=1 SV=4 | VINC_HUMAN | 124 kDa |  | 50% | 47 | 80 | 100 |
| 86 | 14-3-3 protein sigma OS=Homo sapiens GN=SFN PE=1 SV=1 | 1433S_HUMAN | 28 kDa | TRUE | 79% | 17 | 36 | 119 |
| 87 | Collagen alpha-3(VI) chain OS=Homo sapiens GN=COL6A3 PE=1 SV=5 | CO6A3_HUMAN | 344 kDa |  | 26% | 60 | 83 | 98 |
| 88 | Inter-alpha-trypsin inhibitor heavy chain H2 OS=Homo sapiens GN=ITIH2 PE=1 SV=2 | ITIH2_HUMAN | 106 kDa |  | 45% | 36 | 57 | 99 |
| 89 | 14-3-3 protein epsilon OS=Homo sapiens GN=YWHAE PE=1 SV=1 | 1433E_HUMAN | 29 kDa | TRUE | 76% | 23 | 42 | 102 |
| 90 | Ubiquitin-like modifier-activating enzyme 1 OS=Homo sapiens GN=UBA1 PE=1 SV=3 | UBA1_HUMAN | 118 kDa | TRUE | 49% | 40 | 63 | 89 |
| 91 | Actin, alpha cardiac muscle 1 OS=Homo sapiens GN=ACTC1 PE=1 SV=1 | ACTC_HUMAN | 42 kDa | TRUE | 64% | 10 | 22 | 519 |
| 92 | Nicotinamide phosphoribosyltransferase OS=Homo sapiens GN=NAMPT PE=1 SV=1 | NAMPT_HUMAN | 56 kDa | TRUE | 59% | 27 | 55 | 89 |
| 93 | Moesin OS=Homo sapiens GN=MSN PE=1 SV=3 | MOES_HUMAN | 68 kDa | TRUE | 60% | 36 | 56 | 75 |
| 94 | Vitamin K-dependent protein S OS=Homo sapiens GN=PROS1 PE=1 SV=1 | PROS_HUMAN | 75 kDa |  | 40% | 23 | 41 | 88 |
| 95 | Complement component C8 beta chain OS=Homo sapiens GN=C8B PE=1 SV=3 | CO8B_HUMAN | 67 kDa |  | 45% | 24 | 51 | 98 |
| 96 | Acylamino-acid-releasing enzyme OS=Homo sapiens GN=APEH PE=1 SV=4 | ACPH_HUMAN | 81 kDa |  | 54% | 29 | 51 | 93 |
| 97 | Protein disulfide-isomerase OS=Homo sapiens GN=P4HB PE=1 SV=3 | PDIA1_HUMAN | 57 kDa |  | 65% | 33 | 57 | 78 |
| 98 | Serum amyloid P-component OS=Homo sapiens GN=APCS PE=1 SV=2 | SAMP_HUMAN | 25 kDa |  | 55% | 10 | 20 | 85 |
| 99 | Apolipoprotein L1 OS=Homo sapiens GN=APOL1 PE=1 SV=5 | APOL1_HUMAN | 44 kDa |  | 51% | 22 | 44 | 91 |
| 100 | T-complex protein 1 subunit epsilon OS=Homo sapiens GN=CCT5 PE=1 SV=1 | TCPE_HUMAN | 60 kDa |  | 67% | 32 | 61 | 82 |
| 101 | Elongation factor 1-alpha 1 OS=Homo sapiens GN=EEF1A1 PE=1 SV=1 | EF1A1_HUMAN | 50 kDa |  | 50% | 18 | 44 | 86 |
| 102 | T-complex protein 1 subunit theta OS=Homo sapiens GN=CCT8 PE=1 SV=4 | TCPQ_HUMAN | 60 kDa |  | 67% | 30 | 61 | 89 |
| 103 | Stress-induced-phosphoprotein 1 OS=Homo sapiens GN=STIP1 PE=1 SV=1 | STIP1_HUMAN | 63 kDa |  | 49% | 29 | 50 | 68 |
| 104 | Transforming growth factor-beta-induced protein ig-h3 OS=Homo sapiens GN=TGFBI PE=1 SV=1 | BGH3_HUMAN | 75 kDa | TRUE | 51% | 25 | 40 | 70 |
| 105 | Plastin-2 OS=Homo sapiens GN=LCP1 PE=1 SV=6 | PLSL_HUMAN | 70 kDa | TRUE | 67% | 35 | 64 | 88 |
| 106 | Delta-aminolevulinic acid dehydratase OS=Homo sapiens GN=ALAD PE=1 SV=1 | HEM2_HUMAN | 36 kDa |  | 79% | 21 | 48 | 100 |
| 107 | Basement membrane-specific heparan sulfate proteoglycan core protein OS=Homo sapiens GN=HSPG2 PE=1 SV=4 | PGBM_HUMAN | 469 kDa | TRUE | 12% | 42 | 57 | 74 |
| 108 | Kininogen-1 OS=Homo sapiens GN=KNG1 PE=1 SV=2 | KNG1_HUMAN | 72 kDa |  | 35% | 23 | 43 | 81 |
| 109 | ATP-citrate synthase OS=Homo sapiens GN=ACLY PE=1 SV=3 | ACLY_HUMAN | 121 kDa |  | 45% | 43 | 64 | 83 |
| 110 | Calmodulin OS=Homo sapiens GN=CALM1 PE=1 SV=2 | CALM_HUMAN | 17 kDa | TRUE | 77% | 9 | 24 | 98 |
| 111 | Peroxiredoxin-2 OS=Homo sapiens GN=PRDX2 PE=1 SV=5 | PRDX2_HUMAN | 22 kDa | TRUE | 87% | 16 | 30 | 74 |
| 112 | Complement component C6 OS=Homo sapiens GN=C6 PE=1 SV=3 | CO6_HUMAN | 105 kDa |  | 33% | 29 | 48 | 72 |
| 113 | Major vault protein OS=Homo sapiens GN=MVP PE=1 SV=4 | MVP_HUMAN | 99 kDa |  | 64% | 39 | 67 | 82 |
| 114 | Endoplasmin OS=Homo sapiens GN=HSP90B1 PE=1 SV=1 | ENPL_HUMAN | 92 kDa | TRUE | 42% | 31 | 46 | 73 |
| 115 | Ficolin-3 OS=Homo sapiens GN=FCN3 PE=1 SV=2 | FCN3_HUMAN | 33 kDa | TRUE | 47% | 11 | 22 | 66 |
| 116 | Titin OS=Homo sapiens GN=TTN PE=1 SV=4 | TITIN_HUMAN | 3816 kDa | TRUE | 0.05% | 2 | 2 | 2 |
| 117 | Apolipoprotein C-III OS=Homo sapiens GN=APOC3 PE=1 SV=1 | APOC3_HUMAN | 11 kDa |  | 59% | 7 | 12 | 91 |
| 118 | Actin-related protein 3 OS=Homo sapiens GN=ACTR3 PE=1 SV=3 | ARP3_HUMAN | 47 kDa | TRUE | 74% | 24 | 44 | 82 |
| 119 | Fibulin-1 OS=Homo sapiens GN=FBLN1 PE=1 SV=4 | FBLN1_HUMAN | 77 kDa | TRUE | 41% | 22 | 37 | 69 |
| 120 | Plasma kallikrein OS=Homo sapiens GN=KLKB1 PE=1 SV=1 | KLKB1_HUMAN | 71 kDa |  | 48% | 29 | 52 | 75 |
| 121 | T-complex protein 1 subunit beta OS=Homo sapiens GN=CCT2 PE=1 SV=4 | TCPB_HUMAN | 57 kDa |  | 72% | 30 | 58 | 77 |
| 122 | Alpha-enolase OS=Homo sapiens GN=ENO1 PE=1 SV=2 | ENOA_HUMAN | 47 kDa | TRUE | 59% | 22 | 46 | 69 |
| 123 | Ferritin heavy chain OS=Homo sapiens GN=FTH1 PE=1 SV=2 | FRIH_HUMAN | 21 kDa |  | 82% | 12 | 29 | 74 |
| 124 | Complement factor B OS=Homo sapiens GN=CFB PE=1 SV=2 | CFAB_HUMAN | 86 kDa |  | 45% | 29 | 47 | 70 |
| 125 | Leukotriene A-4 hydrolase OS=Homo sapiens GN=LTA4H PE=1 SV=2 | LKHA4_HUMAN | 69 kDa | TRUE | 61% | 30 | 54 | 73 |
| 126 | Phosphatidylinositol-glycan-specific phospholipase D OS=Homo sapiens GN=GPLD1 PE=1 SV=3 | PHLD_HUMAN | 92 kDa | TRUE | 39% | 25 | 49 | 84 |
| 127 | Lamin-B1 OS=Homo sapiens GN=LMNB1 PE=1 SV=2 | LMNB1_HUMAN | 66 kDa | TRUE | 57% | 33 | 60 | 81 |
| 128 | Ig gamma-4 chain C region OS=Homo sapiens GN=IGHG4 PE=1 SV=1 | IGHG4_HUMAN | 36 kDa | TRUE | 69% | 7 | 19 | 163 |
| 129 | Prelamin-A/C OS=Homo sapiens GN=LMNA PE=1 SV=1 | LMNA_HUMAN | 74 kDa | TRUE | 44% | 33 | 47 | 61 |
| 130 | Transketolase OS=Homo sapiens GN=TKT PE=1 SV=3 | TKT_HUMAN | 68 kDa |  | 53% | 28 | 45 | 63 |
| 131 | Ig gamma-2 chain C region OS=Homo sapiens GN=IGHG2 PE=1 SV=2 | IGHG2_HUMAN | 36 kDa | TRUE | 61% | 8 | 18 | 315 |
| 132 | Clathrin heavy chain 1 OS=Homo sapiens GN=CLTC PE=1 SV=5 | CLH1_HUMAN | 192 kDa | TRUE | 38% | 47 | 60 | 67 |
| 133 | Coagulation factor V OS=Homo sapiens GN=F5 PE=1 SV=4 | FA5_HUMAN | 252 kDa | TRUE | 22% | 42 | 61 | 67 |
| 134 | von Willebrand factor OS=Homo sapiens GN=VWF PE=1 SV=4 | VWF_HUMAN | 309 kDa | TRUE | 14% | 34 | 47 | 51 |
| 135 | EH domain-containing protein 1 OS=Homo sapiens GN=EHD1 PE=1 SV=2 | EHD1_HUMAN | 61 kDa | TRUE | 59% | 29 | 52 | 69 |
| 136 | Keratin, type II cytoskeletal 1 OS=Homo sapiens GN=KRT1 PE=1 SV=6 | K2C1_HUMAN | 66 kDa | TRUE | 39% | 27 | 45 | 66 |
| 137 | Catalase OS=Homo sapiens GN=CAT PE=1 SV=3 | CATA_HUMAN | 60 kDa |  | 58% | 26 | 46 | 63 |
| 138 | 78 kDa glucose-regulated protein OS=Homo sapiens GN=HSPA5 PE=1 SV=2 | GRP78_HUMAN | 72 kDa | TRUE | 49% | 30 | 47 | 60 |
| 139 | Cathepsin G OS=Homo sapiens GN=CTSG PE=1 SV=2 | CATG_HUMAN | 29 kDa |  | 45% | 13 | 26 | 71 |
| 140 | Coagulation factor XIII A chain OS=Homo sapiens GN=F13A1 PE=1 SV=4 | F13A_HUMAN | 83 kDa |  | 48% | 26 | 49 | 66 |
| 141 | Cullin-associated NEDD8-dissociated protein 1 OS=Homo sapiens GN=CAND1 PE=1 SV=2 | CAND1_HUMAN | 136 kDa |  | 38% | 37 | 62 | 72 |
| 142 | F-actin-capping protein subunit beta OS=Homo sapiens GN=CAPZB PE=1 SV=4 | CAPZB_HUMAN | 31 kDa |  | 59% | 14 | 35 | 64 |
| 143 | 26S proteasome non-ATPase regulatory subunit 2 OS=Homo sapiens GN=PSMD2 PE=1 SV=3 | PSMD2_HUMAN | 100 kDa | TRUE | 44% | 30 | 44 | 57 |
| 144 | Histone H2A type 1-H OS=Homo sapiens GN=HIST1H2AH PE=1 SV=3 | H2A1H_HUMAN | 14 kDa | TRUE | 63% | 8 | 14 | 49 |
| 145 | Protein S100-A9 OS=Homo sapiens GN=S100A9 PE=1 SV=1 | S10A9_HUMAN | 13 kDa |  | 82% | 8 | 27 | 65 |
| 146 | Lumican OS=Homo sapiens GN=LUM PE=1 SV=2 | LUM_HUMAN | 38 kDa |  | 43% | 14 | 32 | 72 |
| 147 | T-complex protein 1 subunit eta OS=Homo sapiens GN=CCT7 PE=1 SV=2 | TCPH_HUMAN | 59 kDa |  | 56% | 24 | 38 | 58 |
| 148 | Beta-actin-like protein 2 OS=Homo sapiens GN=ACTBL2 PE=1 SV=2 | ACTBL_HUMAN | 42 kDa | TRUE | 38% | 4 | 9 | 291 |
| 149 | Complement C1q subcomponent subunit A OS=Homo sapiens GN=C1QA PE=1 SV=2 | C1QA_HUMAN | 26 kDa |  | 41% | 7 | 13 | 59 |
| 150 | Complement C1q subcomponent subunit C OS=Homo sapiens GN=C1QC PE=1 SV=3 | C1QC_HUMAN | 26 kDa |  | 40% | 7 | 16 | 76 |
| 151 | Inter-alpha-trypsin inhibitor heavy chain H3 OS=Homo sapiens GN=ITIH3 PE=1 SV=2 | ITIH3_HUMAN | 100 kDa | TRUE | 31% | 20 | 37 | 45 |
| 152 | Hsc70-interacting protein OS=Homo sapiens GN=ST13 PE=1 SV=2 | F10A1_HUMAN | 41 kDa |  | 30% | 11 | 28 | 51 |
| 153 | Protein diaphanous homolog 1 OS=Homo sapiens GN=DIAPH1 PE=1 SV=2 | DIAP1_HUMAN | 141 kDa |  | 42% | 41 | 53 | 62 |
| 154 | Hemopexin OS=Homo sapiens GN=HPX PE=1 SV=2 | HEMO_HUMAN | 52 kDa | TRUE | 36% | 16 | 29 | 44 |
| 155 | T-complex protein 1 subunit alpha OS=Homo sapiens GN=TCP1 PE=1 SV=1 | TCPA_HUMAN | 60 kDa | TRUE | 62% | 28 | 47 | 65 |
| 156 | Programmed cell death 6-interacting protein OS=Homo sapiens GN=PDCD6IP PE=1 SV=1 | PDC6I_HUMAN | 96 kDa | TRUE | 37% | 31 | 48 | 54 |
| 157 | Proteasome subunit alpha type-1 OS=Homo sapiens GN=PSMA1 PE=1 SV=1 | PSA1_HUMAN | 30 kDa |  | 75% | 19 | 33 | 63 |
| 158 | T-complex protein 1 subunit zeta OS=Homo sapiens GN=CCT6A PE=1 SV=3 | TCPZ_HUMAN | 58 kDa | TRUE | 58% | 28 | 45 | 59 |
| 159 | Actin-related protein 2/3 complex subunit 2 OS=Homo sapiens GN=ARPC2 PE=1 SV=1 | ARPC2_HUMAN | 34 kDa |  | 64% | 18 | 37 | 53 |
| 160 | Dynamin-2 OS=Homo sapiens GN=DNM2 PE=1 SV=2 | DYN2_HUMAN | 98 kDa | TRUE | 34% | 27 | 36 | 41 |
| 161 | Apolipoprotein M OS=Homo sapiens GN=APOM PE=1 SV=2 | APOM_HUMAN | 21 kDa |  | 60% | 10 | 20 | 46 |
| 162 | Immunoglobulin lambda-like polypeptide 5 OS=Homo sapiens GN=IGLL5 PE=2 SV=2 | IGLL5_HUMAN | 23 kDa | TRUE | 48% | 4 | 10 | 174 |
| 163 | Lysozyme C OS=Homo sapiens GN=LYZ PE=1 SV=1 | LYSC_HUMAN | 17 kDa |  | 68% | 10 | 15 | 46 |
| 164 | Afamin OS=Homo sapiens GN=AFM PE=1 SV=1 | AFAM_HUMAN | 69 kDa | TRUE | 42% | 25 | 39 | 56 |
| 165 | X-ray repair cross-complementing protein 5 OS=Homo sapiens GN=XRCC5 PE=1 SV=3 | XRCC5_HUMAN | 83 kDa | TRUE | 45% | 25 | 45 | 61 |
| 166 | Ubiquitin carboxyl-terminal hydrolase 5 OS=Homo sapiens GN=USP5 PE=1 SV=2 | UBP5_HUMAN | 96 kDa |  | 40% | 25 | 40 | 55 |
| 167 | X-ray repair cross-complementing protein 6 OS=Homo sapiens GN=XRCC6 PE=1 SV=2 | XRCC6_HUMAN | 70 kDa | TRUE | 50% | 25 | 43 | 54 |
| 168 | C4b-binding protein beta chain OS=Homo sapiens GN=C4BPB PE=1 SV=1 | C4BPB_HUMAN | 28 kDa |  | 44% | 10 | 22 | 58 |
| 169 | Eukaryotic initiation factor 4A-I OS=Homo sapiens GN=EIF4A1 PE=1 SV=1 | IF4A1_HUMAN | 46 kDa | TRUE | 51% | 18 | 36 | 56 |
| 170 | T-complex protein 1 subunit delta OS=Homo sapiens GN=CCT4 PE=1 SV=4 | TCPD_HUMAN | 58 kDa |  | 66% | 26 | 36 | 44 |
| 171 | Serine/threonine-protein phosphatase 2A 65 kDa regulatory subunit A alpha isoform OS=Homo sapiens GN=PPP2R1A PE=1 SV=4 | 2AAA_HUMAN | 65 kDa | TRUE | 50% | 25 | 33 | 40 |
| 172 | Complement component C7 OS=Homo sapiens GN=C7 PE=1 SV=2 | CO7_HUMAN | 94 kDa | TRUE | 41% | 28 | 41 | 54 |
| 173 | Hemoglobin subunit delta OS=Homo sapiens GN=HBD PE=1 SV=2 | HBD_HUMAN | 16 kDa | TRUE | 93% | 8 | 21 | 355 |
| 174 | 26S protease regulatory subunit 4 OS=Homo sapiens GN=PSMC1 PE=1 SV=1 | PRS4_HUMAN | 49 kDa | TRUE | 58% | 23 | 41 | 57 |
| 175 | T-complex protein 1 subunit gamma OS=Homo sapiens GN=CCT3 PE=1 SV=4 | TCPG_HUMAN | 61 kDa |  | 61% | 28 | 41 | 53 |
| 176 | V-type proton ATPase catalytic subunit A OS=Homo sapiens GN=ATP6V1A PE=1 SV=2 | VATA_HUMAN | 68 kDa |  | 46% | 22 | 40 | 49 |
| 177 | 14-3-3 protein gamma OS=Homo sapiens GN=YWHAG PE=1 SV=2 | 1433G_HUMAN | 28 kDa | TRUE | 80% | 13 | 27 | 98 |
| 178 | Apolipoprotein C-II OS=Homo sapiens GN=APOC2 PE=1 SV=1 | APOC2_HUMAN | 11 kDa |  | 52% | 5 | 16 | 60 |
| 179 | F-actin-capping protein subunit alpha-1 OS=Homo sapiens GN=CAPZA1 PE=1 SV=3 | CAZA1_HUMAN | 33 kDa | TRUE | 64% | 13 | 27 | 57 |
| 180 | CD5 antigen-like OS=Homo sapiens GN=CD5L PE=1 SV=1 | CD5L_HUMAN | 38 kDa |  | 52% | 16 | 30 | 48 |
| 181 | Histone H3.2 OS=Homo sapiens GN=HIST2H3A PE=1 SV=3 | H32_HUMAN | 15 kDa | TRUE | 47% | 5 | 12 | 29 |
| 182 | Hexokinase-3 OS=Homo sapiens GN=HK3 PE=1 SV=2 | HXK3_HUMAN | 99 kDa | TRUE | 37% | 25 | 39 | 55 |
| 183 | 26S protease regulatory subunit 6A OS=Homo sapiens GN=PSMC3 PE=1 SV=3 | PRS6A_HUMAN | 49 kDa | TRUE | 72% | 27 | 42 | 55 |
| 184 | Calreticulin OS=Homo sapiens GN=CALR PE=1 SV=1 | CALR_HUMAN | 48 kDa |  | 54% | 18 | 32 | 49 |
| 185 | Keratin, type II cytoskeletal 6B OS=Homo sapiens GN=KRT6B PE=1 SV=5 | K2C6B_HUMAN | 60 kDa | TRUE | 35% | 22 | 35 | 61 |
| 186 | Mannan-binding lectin serine protease 1 OS=Homo sapiens GN=MASP1 PE=1 SV=3 | MASP1_HUMAN | 79 kDa | TRUE | 38% | 23 | 38 | 56 |
| 187 | Actin-related protein 2 OS=Homo sapiens GN=ACTR2 PE=1 SV=1 | ARP2_HUMAN | 45 kDa | TRUE | 44% | 13 | 32 | 41 |
| 188 | Complement C1q subcomponent subunit B OS=Homo sapiens GN=C1QB PE=1 SV=3 | C1QB_HUMAN | 27 kDa |  | 47% | 10 | 24 | 47 |
| 189 | Neutral alpha-glucosidase AB OS=Homo sapiens GN=GANAB PE=1 SV=3 | GANAB_HUMAN | 107 kDa |  | 34% | 27 | 31 | 43 |
| 190 | Pyruvate kinase PKLR OS=Homo sapiens GN=PKLR PE=1 SV=2 | KPYR_HUMAN | 62 kDa | TRUE | 66% | 30 | 49 | 78 |
| 191 | Phospholipid transfer protein OS=Homo sapiens GN=PLTP PE=1 SV=1 | PLTP_HUMAN | 55 kDa | TRUE | 31% | 13 | 33 | 51 |
| 192 | Proteasome subunit alpha type-7 OS=Homo sapiens GN=PSMA7 PE=1 SV=1 | PSA7_HUMAN | 28 kDa | TRUE | 59% | 14 | 23 | 43 |
| 193 | C-1-tetrahydrofolate synthase, cytoplasmic OS=Homo sapiens GN=MTHFD1 PE=1 SV=3 | C1TC_HUMAN | 102 kDa |  | 31% | 27 | 43 | 51 |
| 194 | Heterogeneous nuclear ribonucleoprotein K OS=Homo sapiens GN=HNRNPK PE=1 SV=1 | HNRPK_HUMAN | 51 kDa |  | 51% | 21 | 38 | 57 |
| 195 | Prolow-density lipoprotein receptor-related protein 1 OS=Homo sapiens GN=LRP1 PE=1 SV=2 | LRP1_HUMAN | 505 kDa | TRUE | 9.30% | 35 | 42 | 45 |
| 196 | Ubiquitin carboxyl-terminal hydrolase 14 OS=Homo sapiens GN=USP14 PE=1 SV=3 | UBP14_HUMAN | 56 kDa | TRUE | 43% | 20 | 37 | 47 |
| 197 | ATP-dependent 6-phosphofructokinase, liver type OS=Homo sapiens GN=PFKL PE=1 SV=6 | PFKAL_HUMAN | 85 kDa | TRUE | 37% | 26 | 35 | 44 |
| 198 | Adenosylhomocysteinase OS=Homo sapiens GN=AHCY PE=1 SV=4 | SAHH_HUMAN | 48 kDa |  | 37% | 16 | 30 | 53 |
| 199 | Tropomyosin alpha-4 chain OS=Homo sapiens GN=TPM4 PE=1 SV=3 | TPM4_HUMAN | 29 kDa | TRUE | 44% | 15 | 27 | 47 |
| 200 | Spliceosome RNA helicase DDX39B OS=Homo sapiens GN=DDX39B PE=1 SV=1 | DX39B_HUMAN | 49 kDa | TRUE | 36% | 16 | 29 | 45 |
| 201 | Coatomer subunit alpha OS=Homo sapiens GN=COPA PE=1 SV=2 | COPA_HUMAN | 138 kDa |  | 32% | 33 | 44 | 47 |
| 202 | Elongation factor 1-gamma OS=Homo sapiens GN=EEF1G PE=1 SV=3 | EF1G_HUMAN | 50 kDa |  | 48% | 19 | 31 | 43 |
| 203 | Procollagen C-endopeptidase enhancer 1 OS=Homo sapiens GN=PCOLCE PE=1 SV=2 | PCOC1_HUMAN | 48 kDa |  | 51% | 16 | 33 | 52 |
| 204 | Ribosome-binding protein 1 OS=Homo sapiens GN=RRBP1 PE=1 SV=4 | RRBP1_HUMAN | 152 kDa | TRUE | 23% | 30 | 37 | 47 |
| 205 | UV excision repair protein RAD23 homolog A OS=Homo sapiens GN=RAD23A PE=1 SV=1 | RD23A_HUMAN | 40 kDa | TRUE | 51% | 12 | 23 | 57 |
| 206 | Tripeptidyl-peptidase 2 OS=Homo sapiens GN=TPP2 PE=1 SV=4 | TPP2_HUMAN | 138 kDa |  | 26% | 28 | 34 | 39 |
| 207 | Polyubiquitin-C OS=Homo sapiens GN=UBC PE=1 SV=3 | UBC_HUMAN | 77 kDa |  | 6.90% | 5 | 8 | 39 |
| 208 | Galectin-3-binding protein OS=Homo sapiens GN=LGALS3BP PE=1 SV=1 | LG3BP_HUMAN | 65 kDa | TRUE | 38% | 18 | 28 | 44 |
| 209 | Pregnancy zone protein OS=Homo sapiens GN=PZP PE=1 SV=4 | PZP_HUMAN | 164 kDa | TRUE | 34% | 28 | 42 | 117 |
| 210 | Sulfhydryl oxidase 1 OS=Homo sapiens GN=QSOX1 PE=1 SV=3 | QSOX1_HUMAN | 83 kDa |  | 32% | 22 | 29 | 39 |
| 211 | Neuroblast differentiation-associated protein AHNAK OS=Homo sapiens GN=AHNAK PE=1 SV=2 | AHNK_HUMAN | 629 kDa |  | 6.60% | 31 | 32 | 32 |
| 212 | Fructose-bisphosphate aldolase A OS=Homo sapiens GN=ALDOA PE=1 SV=2 | ALDOA_HUMAN | 39 kDa | TRUE | 59% | 19 | 33 | 50 |
| 213 | Dynactin subunit 2 OS=Homo sapiens GN=DCTN2 PE=1 SV=4 | DCTN2_HUMAN | 44 kDa | TRUE | 53% | 19 | 34 | 43 |
| 214 | Heat shock cognate 71 kDa protein OS=Homo sapiens GN=HSPA8 PE=1 SV=1 | HSP7C_HUMAN | 71 kDa | TRUE | 44% | 22 | 39 | 50 |
| 215 | Myeloid cell nuclear differentiation antigen OS=Homo sapiens GN=MNDA PE=1 SV=1 | MNDA_HUMAN | 46 kDa |  | 36% | 13 | 20 | 25 |
| 216 | Protein disulfide-isomerase A4 OS=Homo sapiens GN=PDIA4 PE=1 SV=2 | PDIA4_HUMAN | 73 kDa |  | 39% | 26 | 33 | 38 |
| 217 | 14-3-3 protein beta/alpha OS=Homo sapiens GN=YWHAB PE=1 SV=3 | 1433B_HUMAN | 28 kDa | TRUE | 77% | 8 | 16 | 118 |
| 218 | Lipopolysaccharide-binding protein OS=Homo sapiens GN=LBP PE=1 SV=3 | LBP_HUMAN | 53 kDa | TRUE | 40% | 15 | 25 | 45 |
| 219 | Flavin reductase (NADPH) OS=Homo sapiens GN=BLVRB PE=1 SV=3 | BLVRB_HUMAN | 22 kDa |  | 46% | 7 | 21 | 44 |
| 220 | Keratin, type I cytoskeletal 9 OS=Homo sapiens GN=KRT9 PE=1 SV=3 | K1C9_HUMAN | 62 kDa | TRUE | 51% | 23 | 36 | 48 |
| 221 | 26S proteasome non-ATPase regulatory subunit 3 OS=Homo sapiens GN=PSMD3 PE=1 SV=2 | PSMD3_HUMAN | 61 kDa |  | 43% | 19 | 27 | 36 |
| 222 | Apolipoprotein B receptor OS=Homo sapiens GN=APOBR PE=1 SV=2 | APOBR_HUMAN | 115 kDa | TRUE | 36% | 27 | 37 | 49 |
| 223 | Fatty acid synthase OS=Homo sapiens GN=FASN PE=1 SV=3 | FAS_HUMAN | 273 kDa | TRUE | 17% | 32 | 40 | 40 |
| 224 | 26S protease regulatory subunit 7 OS=Homo sapiens GN=PSMC2 PE=1 SV=3 | PRS7_HUMAN | 49 kDa |  | 49% | 19 | 34 | 46 |
| 225 | Bifunctional purine biosynthesis protein PURH OS=Homo sapiens GN=ATIC PE=1 SV=3 | PUR9_HUMAN | 65 kDa |  | 55% | 24 | 42 | 51 |
| 226 | Complement component C8 alpha chain OS=Homo sapiens GN=C8A PE=1 SV=2 | CO8A_HUMAN | 65 kDa |  | 33% | 15 | 29 | 44 |
| 227 | Dihydropyrimidinase-related protein 2 OS=Homo sapiens GN=DPYSL2 PE=1 SV=1 | DPYL2_HUMAN | 62 kDa | TRUE | 51% | 19 | 31 | 41 |
| 228 | Filamin-B OS=Homo sapiens GN=FLNB PE=1 SV=2 | FLNB_HUMAN | 278 kDa | TRUE | 18% | 29 | 31 | 42 |
| 229 | Leukocyte elastase inhibitor OS=Homo sapiens GN=SERPINB1 PE=1 SV=1 | ILEU_HUMAN | 43 kDa | TRUE | 61% | 20 | 37 | 48 |
| 230 | Ig kappa chain V-III region HAH OS=Homo sapiens PE=2 SV=1 | KV312_HUMAN | 14 kDa | TRUE | 50% | 7 | 11 | 36 |
| 231 | Neutrophil cytosol factor 2 OS=Homo sapiens GN=NCF2 PE=1 SV=2 | NCF2_HUMAN | 60 kDa |  | 49% | 24 | 35 | 40 |
| 232 | Proteasome subunit alpha type-6 OS=Homo sapiens GN=PSMA6 PE=1 SV=1 | PSA6_HUMAN | 27 kDa |  | 50% | 12 | 24 | 52 |
| 233 | Synaptic vesicle membrane protein VAT-1 homolog OS=Homo sapiens GN=VAT1 PE=1 SV=2 | VAT1_HUMAN | 42 kDa |  | 51% | 15 | 28 | 43 |
| 234 | Calpain-1 catalytic subunit OS=Homo sapiens GN=CAPN1 PE=1 SV=1 | CAN1_HUMAN | 82 kDa |  | 42% | 26 | 35 | 43 |
| 235 | DNA damage-binding protein 1 OS=Homo sapiens GN=DDB1 PE=1 SV=1 | DDB1_HUMAN | 127 kDa |  | 23% | 25 | 32 | 37 |
| 236 | Heat shock 70 kDa protein 1A/1B OS=Homo sapiens GN=HSPA1A PE=1 SV=5 | HSP71_HUMAN | ? | TRUE | 0.00% | 16 | 30 | 56 |
| 237 | Heat shock 70 kDa protein 4 OS=Homo sapiens GN=HSPA4 PE=1 SV=4 | HSP74_HUMAN | 94 kDa | TRUE | 39% | 26 | 37 | 43 |
| 238 | Lymphocyte-specific protein 1 OS=Homo sapiens GN=LSP1 PE=1 SV=1 | LSP1_HUMAN | 37 kDa |  | 63% | 16 | 31 | 45 |
| 239 | 40S ribosomal protein S3 OS=Homo sapiens GN=RPS3 PE=1 SV=2 | RS3_HUMAN | 27 kDa |  | 70% | 16 | 25 | 33 |
| 240 | Cytosolic non-specific dipeptidase OS=Homo sapiens GN=CNDP2 PE=1 SV=2 | CNDP2_HUMAN | 53 kDa |  | 60% | 19 | 33 | 43 |
| 241 | 26S proteasome non-ATPase regulatory subunit 1 OS=Homo sapiens GN=PSMD1 PE=1 SV=2 | PSMD1_HUMAN | 106 kDa |  | 41% | 27 | 37 | 46 |
| 242 | Spectrin beta chain, erythrocytic OS=Homo sapiens GN=SPTB PE=1 SV=5 | SPTB1_HUMAN | 246 kDa | TRUE | 22% | 34 | 40 | 46 |
| 243 | Alpha-2-antiplasmin OS=Homo sapiens GN=SERPINF2 PE=1 SV=3 | A2AP_HUMAN | 55 kDa |  | 53% | 18 | 30 | 42 |
| 244 | Dynactin subunit 1 OS=Homo sapiens GN=DCTN1 PE=1 SV=3 | DCTN1_HUMAN | 142 kDa | TRUE | 25% | 25 | 31 | 40 |
| 245 | Glutathione peroxidase 3 OS=Homo sapiens GN=GPX3 PE=1 SV=2 | GPX3_HUMAN | 26 kDa |  | 35% | 7 | 17 | 30 |
| 246 | High mobility group protein B2 OS=Homo sapiens GN=HMGB2 PE=1 SV=2 | HMGB2_HUMAN | 24 kDa | TRUE | 25% | 7 | 21 | 29 |
| 247 | Myosin light polypeptide 6 OS=Homo sapiens GN=MYL6 PE=1 SV=2 | MYL6_HUMAN | 17 kDa | TRUE | 52% | 8 | 21 | 41 |
| 248 | Protein S100-P OS=Homo sapiens GN=S100P PE=1 SV=2 | S100P_HUMAN | 10 kDa | TRUE | 79% | 6 | 12 | 32 |
| 249 | Transaldolase OS=Homo sapiens GN=TALDO1 PE=1 SV=2 | TALDO_HUMAN | 38 kDa |  | 46% | 18 | 29 | 36 |
| 250 | Histone H1.5 OS=Homo sapiens GN=HIST1H1B PE=1 SV=3 | H15_HUMAN | 23 kDa | TRUE | 32% | 9 | 14 | 33 |
| 251 | Inter-alpha-trypsin inhibitor heavy chain H1 OS=Homo sapiens GN=ITIH1 PE=1 SV=3 | ITIH1_HUMAN | 101 kDa | TRUE | 36% | 23 | 31 | 39 |
| 252 | Keratin, type I cytoskeletal 14 OS=Homo sapiens GN=KRT14 PE=1 SV=4 | K1C14_HUMAN | 52 kDa | TRUE | 43% | 20 | 29 | 41 |
| 253 | Protein-arginine deiminase type-4 OS=Homo sapiens GN=PADI4 PE=1 SV=2 | PADI4_HUMAN | 74 kDa | TRUE | 34% | 17 | 33 | 42 |
| 254 | GTP-binding nuclear protein Ran OS=Homo sapiens GN=RAN PE=1 SV=3 | RAN_HUMAN | 24 kDa |  | 67% | 11 | 20 | 37 |
| 255 | UTP--glucose-1-phosphate uridylyltransferase OS=Homo sapiens GN=UGP2 PE=1 SV=5 | UGPA_HUMAN | 57 kDa | TRUE | 46% | 19 | 29 | 34 |
| 256 | Retinal dehydrogenase 1 OS=Homo sapiens GN=ALDH1A1 PE=1 SV=2 | AL1A1_HUMAN | 55 kDa | TRUE | 37% | 16 | 29 | 38 |
| 257 | Ankyrin-1 OS=Homo sapiens GN=ANK1 PE=1 SV=3 | ANK1_HUMAN | 206 kDa |  | 17% | 24 | 33 | 42 |
| 258 | Collagen alpha-1(VI) chain OS=Homo sapiens GN=COL6A1 PE=1 SV=3 | CO6A1_HUMAN | 109 kDa | TRUE | 18% | 16 | 21 | 28 |
| 259 | Ferritin light chain OS=Homo sapiens GN=FTL PE=1 SV=2 | FRIL_HUMAN | 20 kDa |  | 55% | 10 | 21 | 30 |
| 260 | Heterogeneous nuclear ribonucleoprotein Q OS=Homo sapiens GN=SYNCRIP PE=1 SV=2 | HNRPQ_HUMAN | 70 kDa | TRUE | 37% | 19 | 30 | 40 |
| 261 | Laminin subunit beta-1 OS=Homo sapiens GN=LAMB1 PE=1 SV=2 | LAMB1_HUMAN | 198 kDa | TRUE | 15% | 21 | 29 | 32 |
| 262 | Vesicle-fusing ATPase OS=Homo sapiens GN=NSF PE=1 SV=3 | NSF_HUMAN | 83 kDa | TRUE | 40% | 26 | 36 | 42 |
| 263 | Adenylosuccinate lyase OS=Homo sapiens GN=ADSL PE=1 SV=2 | PUR8_HUMAN | 55 kDa |  | 54% | 19 | 29 | 38 |
| 264 | Retinol-binding protein 4 OS=Homo sapiens GN=RBP4 PE=1 SV=3 | RET4_HUMAN | 23 kDa | TRUE | 41% | 6 | 10 | 43 |
| 265 | C-reactive protein OS=Homo sapiens GN=CRP PE=1 SV=1 | CRP_HUMAN | 25 kDa |  | 25% | 7 | 15 | 39 |
| 266 | Importin subunit beta-1 OS=Homo sapiens GN=KPNB1 PE=1 SV=2 | IMB1_HUMAN | 97 kDa |  | 31% | 20 | 28 | 34 |
| 267 | Perilipin-3 OS=Homo sapiens GN=PLIN3 PE=1 SV=3 | PLIN3_HUMAN | 47 kDa |  | 55% | 15 | 26 | 34 |
| 268 | Fermitin family homolog 3 OS=Homo sapiens GN=FERMT3 PE=1 SV=1 | URP2_HUMAN | 76 kDa |  | 43% | 21 | 32 | 34 |
| 269 | V-type proton ATPase subunit B, brain isoform OS=Homo sapiens GN=ATP6V1B2 PE=1 SV=3 | VATB2_HUMAN | 57 kDa |  | 54% | 19 | 27 | 39 |
| 270 | Carboxypeptidase N catalytic chain OS=Homo sapiens GN=CPN1 PE=1 SV=1 | CBPN_HUMAN | 52 kDa |  | 33% | 11 | 22 | 32 |
| 271 | Extracellular matrix protein 1 OS=Homo sapiens GN=ECM1 PE=1 SV=2 | ECM1_HUMAN | 61 kDa |  | 35% | 17 | 27 | 33 |
| 272 | Keratin, type I cytoskeletal 10 OS=Homo sapiens GN=KRT10 PE=1 SV=6 | K1C10_HUMAN | 59 kDa | TRUE | 39% | 19 | 31 | 48 |
| 273 | Kinesin-1 heavy chain OS=Homo sapiens GN=KIF5B PE=1 SV=1 | KINH_HUMAN | 110 kDa | TRUE | 35% | 28 | 34 | 38 |
| 274 | Proteasome activator complex subunit 1 OS=Homo sapiens GN=PSME1 PE=1 SV=1 | PSME1_HUMAN | 29 kDa | TRUE | 50% | 14 | 24 | 37 |
| 275 | AP-2 complex subunit alpha-1 OS=Homo sapiens GN=AP2A1 PE=1 SV=3 | AP2A1_HUMAN | 108 kDa | TRUE | 30% | 24 | 32 | 37 |
| 276 | Complement factor H-related protein 5 OS=Homo sapiens GN=CFHR5 PE=1 SV=1 | FHR5_HUMAN | 64 kDa | TRUE | 24% | 14 | 23 | 32 |
| 277 | Histone H4 OS=Homo sapiens GN=HIST1H4A PE=1 SV=2 | H4_HUMAN | 11 kDa |  | 60% | 9 | 20 | 43 |
| 278 | Tyrosine-protein kinase SYK OS=Homo sapiens GN=SYK PE=1 SV=1 | KSYK_HUMAN | 72 kDa |  | 29% | 14 | 17 | 17 |
| 279 | Puromycin-sensitive aminopeptidase OS=Homo sapiens GN=NPEPPS PE=1 SV=2 | PSA_HUMAN | 103 kDa |  | 29% | 24 | 31 | 33 |
| 280 | Serine/threonine-protein kinase 10 OS=Homo sapiens GN=STK10 PE=1 SV=1 | STK10_HUMAN | 112 kDa | TRUE | 22% | 18 | 29 | 30 |
| 281 | Protein-glutamine gamma-glutamyltransferase 2 OS=Homo sapiens GN=TGM2 PE=1 SV=2 | TGM2_HUMAN | 77 kDa |  | 30% | 17 | 25 | 33 |
| 282 | Beta-Ala-His dipeptidase OS=Homo sapiens GN=CNDP1 PE=1 SV=4 | CNDP1_HUMAN | 57 kDa |  | 39% | 15 | 27 | 34 |
| 283 | Protein DDI1 homolog 2 OS=Homo sapiens GN=DDI2 PE=1 SV=1 | DDI2_HUMAN | 45 kDa |  | 53% | 14 | 24 | 34 |
| 284 | 1,4-alpha-glucan-branching enzyme OS=Homo sapiens GN=GBE1 PE=1 SV=3 | GLGB_HUMAN | 80 kDa |  | 34% | 19 | 27 | 32 |
| 285 | Drebrin-like protein OS=Homo sapiens GN=DBNL PE=1 SV=1 | DBNL_HUMAN | 48 kDa | TRUE | 44% | 14 | 22 | 33 |
| 286 | Eukaryotic translation initiation factor 3 subunit A OS=Homo sapiens GN=EIF3A PE=1 SV=1 | EIF3A_HUMAN | 167 kDa | TRUE | 16% | 21 | 25 | 27 |
| 287 | Ficolin-2 OS=Homo sapiens GN=FCN2 PE=1 SV=2 | FCN2_HUMAN | 34 kDa | TRUE | 34% | 9 | 16 | 26 |
| 288 | Protein flightless-1 homolog OS=Homo sapiens GN=FLII PE=1 SV=2 | FLII_HUMAN | 145 kDa | TRUE | 26% | 25 | 30 | 32 |
| 289 | Peptidyl-prolyl cis-trans isomerase A OS=Homo sapiens GN=PPIA PE=1 SV=2 | PPIA_HUMAN | 18 kDa | TRUE | 59% | 12 | 23 | 32 |
| 290 | UV excision repair protein RAD23 homolog B OS=Homo sapiens GN=RAD23B PE=1 SV=1 | RD23B_HUMAN | 43 kDa | TRUE | 45% | 14 | 22 | 40 |
| 291 | E3 ubiquitin-protein ligase RNF123 OS=Homo sapiens GN=RNF123 PE=1 SV=1 | RN123_HUMAN | 149 kDa | TRUE | 17% | 17 | 22 | 26 |
| 292 | Deoxynucleoside triphosphate triphosphohydrolase SAMHD1 OS=Homo sapiens GN=SAMHD1 PE=1 SV=2 | SAMH1_HUMAN | 72 kDa |  | 45% | 22 | 27 | 34 |
| 293 | Zyxin OS=Homo sapiens GN=ZYX PE=1 SV=1 | ZYX_HUMAN | 61 kDa | TRUE | 28% | 11 | 19 | 36 |
| 294 | Actin-related protein 2/3 complex subunit 1B OS=Homo sapiens GN=ARPC1B PE=1 SV=3 | ARC1B_HUMAN | 41 kDa | TRUE | 45% | 15 | 22 | 30 |
| 295 | Cathelicidin antimicrobial peptide OS=Homo sapiens GN=CAMP PE=1 SV=1 | CAMP_HUMAN | 19 kDa | TRUE | 36% | 9 | 15 | 29 |
| 296 | Heterogeneous nuclear ribonucleoprotein U OS=Homo sapiens GN=HNRNPU PE=1 SV=6 | HNRPU_HUMAN | 91 kDa | TRUE | 18% | 14 | 21 | 30 |
| 297 | DNA-dependent protein kinase catalytic subunit OS=Homo sapiens GN=PRKDC PE=1 SV=3 | PRKDC_HUMAN | 469 kDa | TRUE | 6.00% | 19 | 20 | 21 |
| 298 | 26S protease regulatory subunit 10B OS=Homo sapiens GN=PSMC6 PE=1 SV=1 | PRS10_HUMAN | 44 kDa | TRUE | 58% | 19 | 28 | 35 |
| 299 | Proteasome subunit alpha type-5 OS=Homo sapiens GN=PSMA5 PE=1 SV=3 | PSA5_HUMAN | 26 kDa |  | 55% | 10 | 23 | 38 |
| 300 | Heterogeneous nuclear ribonucleoproteins A2/B1 OS=Homo sapiens GN=HNRNPA2B1 PE=1 SV=2 | ROA2_HUMAN | 37 kDa | TRUE | 34% | 11 | 17 | 31 |
| 301 | Alpha-1B-glycoprotein OS=Homo sapiens GN=A1BG PE=1 SV=4 | A1BG_HUMAN | 54 kDa |  | 35% | 12 | 19 | 33 |
| 302 | Glucose-6-phosphate isomerase OS=Homo sapiens GN=GPI PE=1 SV=4 | G6PI_HUMAN | 63 kDa | TRUE | 32% | 14 | 21 | 26 |
| 303 | Eukaryotic translation initiation factor 5A-1 OS=Homo sapiens GN=EIF5A PE=1 SV=2 | IF5A1_HUMAN | 17 kDa |  | 68% | 10 | 22 | 33 |
| 304 | 26S protease regulatory subunit 8 OS=Homo sapiens GN=PSMC5 PE=1 SV=1 | PRS8_HUMAN | 46 kDa | TRUE | 56% | 19 | 29 | 45 |
| 305 | Phosphoribosylformylglycinamidine synthase OS=Homo sapiens GN=PFAS PE=1 SV=4 | PUR4_HUMAN | 145 kDa | TRUE | 21% | 21 | 26 | 32 |
| 306 | Aldehyde dehydrogenase family 16 member A1 OS=Homo sapiens GN=ALDH16A1 PE=1 SV=2 | A16A1_HUMAN | 85 kDa |  | 21% | 14 | 20 | 29 |
| 307 | AMP deaminase 3 OS=Homo sapiens GN=AMPD3 PE=1 SV=1 | AMPD3_HUMAN | 89 kDa | TRUE | 29% | 20 | 25 | 27 |
| 308 | Bactericidal permeability-increasing protein OS=Homo sapiens GN=BPI PE=1 SV=4 | BPI_HUMAN | 54 kDa |  | 54% | 15 | 24 | 33 |
| 309 | Azurocidin OS=Homo sapiens GN=AZU1 PE=1 SV=3 | CAP7_HUMAN | 27 kDa |  | 45% | 7 | 13 | 32 |
| 310 | Complement factor I OS=Homo sapiens GN=CFI PE=1 SV=2 | CFAI_HUMAN | 66 kDa | TRUE | 23% | 12 | 18 | 22 |
| 311 | Heterogeneous nuclear ribonucleoprotein L OS=Homo sapiens GN=HNRNPL PE=1 SV=2 | HNRPL_HUMAN | 64 kDa |  | 43% | 15 | 25 | 29 |
| 312 | Interleukin enhancer-binding factor 3 OS=Homo sapiens GN=ILF3 PE=1 SV=3 | ILF3_HUMAN | 95 kDa | TRUE | 27% | 18 | 25 | 34 |
| 313 | Ig kappa chain V-IV region Len OS=Homo sapiens PE=1 SV=2 | KV402_HUMAN | 13 kDa | TRUE | 43% | 4 | 10 | 37 |
| 314 | Nucleophosmin OS=Homo sapiens GN=NPM1 PE=1 SV=2 | NPM_HUMAN | 33 kDa |  | 39% | 11 | 22 | 33 |
| 315 | Rho GTPase-activating protein 1 OS=Homo sapiens GN=ARHGAP1 PE=1 SV=1 | RHG01_HUMAN | 50 kDa |  | 53% | 17 | 23 | 30 |
| 316 | Exportin-1 OS=Homo sapiens GN=XPO1 PE=1 SV=1 | XPO1_HUMAN | 123 kDa | TRUE | 26% | 20 | 24 | 26 |
| 317 | 14-3-3 protein theta OS=Homo sapiens GN=YWHAQ PE=1 SV=1 | 1433T_HUMAN | 28 kDa | TRUE | 57% | 9 | 20 | 85 |
| 318 | Protein AMBP OS=Homo sapiens GN=AMBP PE=1 SV=1 | AMBP_HUMAN | 39 kDa |  | 35% | 9 | 17 | 37 |
| 319 | AP-1 complex subunit beta-1 OS=Homo sapiens GN=AP1B1 PE=1 SV=2 | AP1B1_HUMAN | 105 kDa | TRUE | 30% | 23 | 28 | 29 |
| 320 | ATP-dependent RNA helicase A OS=Homo sapiens GN=DHX9 PE=1 SV=4 | DHX9_HUMAN | 141 kDa |  | 18% | 16 | 18 | 24 |
| 321 | NSFL1 cofactor p47 OS=Homo sapiens GN=NSFL1C PE=1 SV=2 | NSF1C_HUMAN | 41 kDa |  | 54% | 15 | 23 | 33 |
| 322 | Proteasome subunit alpha type-3 OS=Homo sapiens GN=PSMA3 PE=1 SV=2 | PSA3_HUMAN | 28 kDa | TRUE | 37% | 12 | 20 | 29 |
| 323 | 26S proteasome non-ATPase regulatory subunit 12 OS=Homo sapiens GN=PSMD12 PE=1 SV=3 | PSD12_HUMAN | 53 kDa |  | 45% | 19 | 29 | 30 |
| 324 | Splicing factor, proline- and glutamine-rich OS=Homo sapiens GN=SFPQ PE=1 SV=2 | SFPQ_HUMAN | 76 kDa | TRUE | 30% | 19 | 26 | 32 |
| 325 | Ig heavy chain V-III region BUT OS=Homo sapiens PE=1 SV=1 | HV306_HUMAN | 12 kDa | TRUE | 36% | 4 | 10 | 34 |
| 326 | 14-3-3 protein eta OS=Homo sapiens GN=YWHAH PE=1 SV=4 | 1433F_HUMAN | 28 kDa | TRUE | 68% | 13 | 23 | 77 |
| 327 | 4-trimethylaminobutyraldehyde dehydrogenase OS=Homo sapiens GN=ALDH9A1 PE=1 SV=3 | AL9A1_HUMAN | 54 kDa | TRUE | 35% | 15 | 22 | 28 |
| 328 | Cadherin-5 OS=Homo sapiens GN=CDH5 PE=1 SV=5 | CADH5_HUMAN | 88 kDa |  | 20% | 13 | 21 | 30 |
| 329 | Polyadenylate-binding protein 1 OS=Homo sapiens GN=PABPC1 PE=1 SV=2 | PABP1_HUMAN | 71 kDa | TRUE | 28% | 16 | 24 | 28 |
| 330 | Peroxiredoxin-6 OS=Homo sapiens GN=PRDX6 PE=1 SV=3 | PRDX6_HUMAN | 25 kDa | TRUE | 70% | 17 | 24 | 29 |
| 331 | Carboxypeptidase N subunit 2 OS=Homo sapiens GN=CPN2 PE=1 SV=3 | CPN2_HUMAN | 61 kDa |  | 40% | 14 | 21 | 32 |
| 332 | Glucosidase 2 subunit beta OS=Homo sapiens GN=PRKCSH PE=1 SV=2 | GLU2B_HUMAN | 59 kDa | TRUE | 29% | 13 | 20 | 24 |
| 333 | Hemoglobin subunit gamma-2 OS=Homo sapiens GN=HBG2 PE=1 SV=2 | HBG2_HUMAN | 16 kDa | TRUE | 79% | 12 | 25 | 76 |
| 334 | Isocitrate dehydrogenase [NADP] cytoplasmic OS=Homo sapiens GN=IDH1 PE=1 SV=2 | IDHC_HUMAN | 47 kDa | TRUE | 58% | 19 | 26 | 32 |
| 335 | Unconventional myosin-If OS=Homo sapiens GN=MYO1F PE=1 SV=3 | MYO1F_HUMAN | 125 kDa | TRUE | 26% | 19 | 22 | 25 |
| 336 | Serine/threonine-protein phosphatase 2A catalytic subunit alpha isoform OS=Homo sapiens GN=PPP2CA PE=1 SV=1 | PP2AA_HUMAN | 36 kDa | TRUE | 67% | 14 | 20 | 27 |
| 337 | Proteasome subunit alpha type-2 OS=Homo sapiens GN=PSMA2 PE=1 SV=2 | PSA2_HUMAN | 26 kDa | TRUE | 48% | 10 | 20 | 29 |
| 338 | Adenylosuccinate synthetase isozyme 2 OS=Homo sapiens GN=ADSS PE=1 SV=3 | PURA2_HUMAN | 50 kDa |  | 43% | 16 | 22 | 31 |
| 339 | Ras-related C3 botulinum toxin substrate 2 OS=Homo sapiens GN=RAC2 PE=1 SV=1 | RAC2_HUMAN | 21 kDa | TRUE | 35% | 6 | 14 | 27 |
| 340 | Transforming protein RhoA OS=Homo sapiens GN=RHOA PE=1 SV=1 | RHOA_HUMAN | 22 kDa | TRUE | 51% | 9 | 20 | 32 |
| 341 | Bifunctional glutamate/proline--tRNA ligase OS=Homo sapiens GN=EPRS PE=1 SV=5 | SYEP_HUMAN | 171 kDa | TRUE | 13% | 17 | 20 | 22 |
| 342 | Thrombospondin-1 OS=Homo sapiens GN=THBS1 PE=1 SV=2 | TSP1_HUMAN | 129 kDa | TRUE | 19% | 19 | 23 | 29 |
| 343 | Protein Z-dependent protease inhibitor OS=Homo sapiens GN=SERPINA10 PE=1 SV=1 | ZPI_HUMAN | 51 kDa |  | 30% | 12 | 17 | 26 |
| 344 | Alpha-1-acid glycoprotein 2 OS=Homo sapiens GN=ORM2 PE=1 SV=2 | A1AG2_HUMAN | 24 kDa | TRUE | 47% | 10 | 19 | 28 |
| 345 | Alpha-centractin OS=Homo sapiens GN=ACTR1A PE=1 SV=1 | ACTZ_HUMAN | 43 kDa | TRUE | 44% | 12 | 20 | 25 |
| 346 | Coatomer subunit beta' OS=Homo sapiens GN=COPB2 PE=1 SV=2 | COPB2_HUMAN | 102 kDa |  | 24% | 18 | 24 | 28 |
| 347 | Cullin-3 OS=Homo sapiens GN=CUL3 PE=1 SV=2 | CUL3_HUMAN | 89 kDa | TRUE | 26% | 20 | 25 | 30 |
| 348 | Importin-5 OS=Homo sapiens GN=IPO5 PE=1 SV=4 | IPO5_HUMAN | 124 kDa | TRUE | 23% | 19 | 27 | 29 |
| 349 | Laminin subunit gamma-1 OS=Homo sapiens GN=LAMC1 PE=1 SV=3 | LAMC1_HUMAN | 178 kDa |  | 15% | 21 | 27 | 29 |
| 350 | Myosin regulatory light chain 12A OS=Homo sapiens GN=MYL12A PE=1 SV=2 | ML12A_HUMAN | 20 kDa | TRUE | 73% | 12 | 22 | 31 |
| 351 | Profilin-1 OS=Homo sapiens GN=PFN1 PE=1 SV=2 | PROF1_HUMAN | 15 kDa |  | 70% | 9 | 21 | 36 |
| 352 | 26S protease regulatory subunit 6B OS=Homo sapiens GN=PSMC4 PE=1 SV=2 | PRS6B_HUMAN | 47 kDa | TRUE | 52% | 13 | 20 | 29 |
| 353 | Tyrosine-protein phosphatase non-receptor type 6 OS=Homo sapiens GN=PTPN6 PE=1 SV=1 | PTN6_HUMAN | 68 kDa | TRUE | 36% | 18 | 21 | 23 |
| 354 | 40S ribosomal protein S19 OS=Homo sapiens GN=RPS19 PE=1 SV=2 | RS19_HUMAN | 16 kDa |  | 50% | 10 | 17 | 26 |
| 355 | Selenium-binding protein 1 OS=Homo sapiens GN=SELENBP1 PE=1 SV=2 | SBP1_HUMAN | 52 kDa |  | 46% | 18 | 26 | 35 |
| 356 | Signal transducer and activator of transcription 1-alpha/beta OS=Homo sapiens GN=STAT1 PE=1 SV=2 | STAT1_HUMAN | 87 kDa | TRUE | 27% | 17 | 22 | 23 |
| 357 | Nipped-B-like protein OS=Homo sapiens GN=NIPBL PE=1 SV=2 | NIPBL_HUMAN | 316 kDa | TRUE | 1.90% | 2 | 2 | 2 |
| 358 | Protein 4.1 OS=Homo sapiens GN=EPB41 PE=1 SV=4 | 41_HUMAN | 97 kDa | TRUE | 24% | 15 | 21 | 23 |
| 359 | Elongation factor 1-delta OS=Homo sapiens GN=EEF1D PE=1 SV=5 | EF1D_HUMAN | 31 kDa | TRUE | 50% | 11 | 18 | 30 |
| 360 | FMNL_HUMAN | FMNL_HUMAN | ? | TRUE | 0.00% | 19 | 21 | 21 |
| 361 | Histone H1.4 OS=Homo sapiens GN=HIST1H1E PE=1 SV=2 | H14_HUMAN | 22 kDa | TRUE | 31% | 7 | 13 | 45 |
| 362 | Mitogen-activated protein kinase 1 OS=Homo sapiens GN=MAPK1 PE=1 SV=3 | MK01_HUMAN | 41 kDa | TRUE | 43% | 14 | 23 | 32 |
| 363 | STE20-like serine/threonine-protein kinase OS=Homo sapiens GN=SLK PE=1 SV=1 | SLK_HUMAN | 143 kDa | TRUE | 22% | 20 | 23 | 30 |
| 364 | Ubiquitin carboxyl-terminal hydrolase 15 OS=Homo sapiens GN=USP15 PE=1 SV=3 | UBP15_HUMAN | 112 kDa | TRUE | 24% | 22 | 29 | 32 |
| 365 | Insulin-like growth factor-binding protein complex acid labile subunit OS=Homo sapiens GN=IGFALS PE=1 SV=1 | ALS_HUMAN | 66 kDa |  | 34% | 16 | 23 | 31 |
| 366 | Chloride intracellular channel protein 1 OS=Homo sapiens GN=CLIC1 PE=1 SV=4 | CLIC1_HUMAN | 27 kDa | TRUE | 66% | 13 | 20 | 26 |
| 367 | Coagulation factor XIII B chain OS=Homo sapiens GN=F13B PE=1 SV=3 | F13B_HUMAN | 76 kDa |  | 24% | 12 | 16 | 23 |
| 368 | Hematopoietic lineage cell-specific protein OS=Homo sapiens GN=HCLS1 PE=1 SV=3 | HCLS1_HUMAN | 54 kDa | TRUE | 33% | 15 | 25 | 29 |
| 369 | L-lactate dehydrogenase B chain OS=Homo sapiens GN=LDHB PE=1 SV=2 | LDHB_HUMAN | 37 kDa | TRUE | 54% | 15 | 24 | 35 |
| 370 | Protein disulfide-isomerase A6 OS=Homo sapiens GN=PDIA6 PE=1 SV=1 | PDIA6_HUMAN | 48 kDa |  | 41% | 14 | 20 | 26 |
| 371 | Ras-related protein Rab-1B OS=Homo sapiens GN=RAB1B PE=1 SV=1 | RAB1B_HUMAN | 22 kDa | TRUE | 60% | 11 | 21 | 26 |
| 372 | 60S ribosomal protein L5 OS=Homo sapiens GN=RPL5 PE=1 SV=3 | RL5_HUMAN | 34 kDa |  | 35% | 10 | 14 | 14 |
| 373 | Sorting nexin-6 OS=Homo sapiens GN=SNX6 PE=1 SV=1 | SNX6_HUMAN | 47 kDa | TRUE | 25% | 10 | 15 | 19 |
| 374 | Aspartate--tRNA ligase, cytoplasmic OS=Homo sapiens GN=DARS PE=1 SV=2 | SYDC_HUMAN | 57 kDa | TRUE | 47% | 22 | 26 | 26 |
| 375 | Thymidine phosphorylase OS=Homo sapiens GN=TYMP PE=1 SV=2 | TYPH_HUMAN | 50 kDa |  | 53% | 19 | 27 | 32 |
| 376 | Ubiquitin-associated domain-containing protein 1 OS=Homo sapiens GN=UBAC1 PE=1 SV=1 | UBAC1_HUMAN | 45 kDa | TRUE | 38% | 13 | 19 | 26 |
| 377 | Vigilin OS=Homo sapiens GN=HDLBP PE=1 SV=2 | VIGLN_HUMAN | 141 kDa | TRUE | 18% | 17 | 20 | 24 |
| 378 | Vacuolar protein sorting-associated protein 35 OS=Homo sapiens GN=VPS35 PE=1 SV=2 | VPS35_HUMAN | 92 kDa | TRUE | 29% | 18 | 21 | 25 |
| 379 | Coatomer subunit beta OS=Homo sapiens GN=COPB1 PE=1 SV=3 | COPB_HUMAN | 107 kDa | TRUE | 25% | 19 | 25 | 29 |
| 380 | 55 kDa erythrocyte membrane protein OS=Homo sapiens GN=MPP1 PE=1 SV=2 | EM55_HUMAN | 52 kDa |  | 27% | 12 | 18 | 20 |
| 381 | EGF-containing fibulin-like extracellular matrix protein 1 OS=Homo sapiens GN=EFEMP1 PE=1 SV=2 | FBLN3_HUMAN | 55 kDa |  | 26% | 11 | 17 | 25 |
| 382 | Heat shock protein beta-1 OS=Homo sapiens GN=HSPB1 PE=1 SV=2 | HSPB1_HUMAN | 23 kDa |  | 69% | 13 | 22 | 31 |
| 383 | Leucine-rich repeat flightless-interacting protein 1 OS=Homo sapiens GN=LRRFIP1 PE=1 SV=2 | LRRF1_HUMAN | 89 kDa | TRUE | 24% | 16 | 25 | 32 |
| 384 | Mannan-binding lectin serine protease 2 OS=Homo sapiens GN=MASP2 PE=1 SV=4 | MASP2_HUMAN | 76 kDa |  | 23% | 12 | 16 | 28 |
| 385 | Phosphatidylinositol 5-phosphate 4-kinase type-2 alpha OS=Homo sapiens GN=PIP4K2A PE=1 SV=2 | PI42A_HUMAN | 46 kDa | TRUE | 39% | 12 | 16 | 20 |
| 386 | 1-phosphatidylinositol 4,5-bisphosphate phosphodiesterase gamma-2 OS=Homo sapiens GN=PLCG2 PE=1 SV=4 | PLCG2_HUMAN | 148 kDa | TRUE | 17% | 19 | 21 | 22 |
| 387 | Ribose-phosphate pyrophosphokinase 1 OS=Homo sapiens GN=PRPS1 PE=1 SV=2 | PRPS1_HUMAN | 35 kDa | TRUE | 49% | 14 | 26 | 31 |
| 388 | Multifunctional protein ADE2 OS=Homo sapiens GN=PAICS PE=1 SV=3 | PUR6_HUMAN | 47 kDa | TRUE | 31% | 12 | 15 | 17 |
| 389 | Ribonuclease inhibitor OS=Homo sapiens GN=RNH1 PE=1 SV=2 | RINI_HUMAN | 50 kDa |  | 49% | 17 | 23 | 29 |
| 390 | Carboxypeptidase B2 OS=Homo sapiens GN=CPB2 PE=1 SV=2 | CBPB2_HUMAN | 48 kDa |  | 33% | 13 | 20 | 27 |
| 391 | Dedicator of cytokinesis protein 2 OS=Homo sapiens GN=DOCK2 PE=1 SV=2 | DOCK2_HUMAN | 212 kDa | TRUE | 10% | 17 | 18 | 19 |
| 392 | Fascin OS=Homo sapiens GN=FSCN1 PE=1 SV=3 | FSCN1_HUMAN | 55 kDa | TRUE | 47% | 17 | 23 | 29 |
| 393 | Ras GTPase-activating-like protein IQGAP2 OS=Homo sapiens GN=IQGAP2 PE=1 SV=4 | IQGA2_HUMAN | 181 kDa | TRUE | 20% | 23 | 25 | 32 |
| 394 | Phosphatidylcholine-sterol acyltransferase OS=Homo sapiens GN=LCAT PE=1 SV=1 | LCAT_HUMAN | 50 kDa |  | 23% | 7 | 14 | 25 |
| 395 | Nucleobindin-1 OS=Homo sapiens GN=NUCB1 PE=1 SV=4 | NUCB1_HUMAN | 54 kDa |  | 46% | 16 | 21 | 27 |
| 396 | Proliferation-associated protein 2G4 OS=Homo sapiens GN=PA2G4 PE=1 SV=3 | PA2G4_HUMAN | 44 kDa |  | 38% | 13 | 18 | 22 |
| 397 | Proteasome inhibitor PI31 subunit OS=Homo sapiens GN=PSMF1 PE=1 SV=2 | PSMF1_HUMAN | 30 kDa |  | 40% | 9 | 16 | 26 |
| 398 | 40S ribosomal protein S2 OS=Homo sapiens GN=RPS2 PE=1 SV=2 | RS2_HUMAN | 31 kDa |  | 42% | 12 | 17 | 20 |
| 399 | RuvB-like 2 OS=Homo sapiens GN=RUVBL2 PE=1 SV=3 | RUVB2_HUMAN | 51 kDa | TRUE | 49% | 20 | 24 | 29 |
| 400 | Syntaxin-binding protein 2 OS=Homo sapiens GN=STXBP2 PE=1 SV=2 | STXB2_HUMAN | 66 kDa | TRUE | 28% | 16 | 20 | 21 |
| 401 | Tropomyosin alpha-3 chain OS=Homo sapiens GN=TPM3 PE=1 SV=2 | TPM3_HUMAN | 33 kDa | TRUE | 31% | 6 | 11 | 43 |
| 402 | Ubiquitin-like modifier-activating enzyme 6 OS=Homo sapiens GN=UBA6 PE=1 SV=1 | UBA6_HUMAN | 118 kDa |  | 17% | 16 | 20 | 25 |
| 403 | Protein argonaute-2 OS=Homo sapiens GN=AGO2 PE=1 SV=3 | AGO2_HUMAN | 97 kDa | TRUE | 19% | 13 | 17 | 20 |
| 404 | Cytosol aminopeptidase OS=Homo sapiens GN=LAP3 PE=1 SV=3 | AMPL_HUMAN | 56 kDa |  | 43% | 19 | 23 | 29 |
| 405 | Complement component C8 gamma chain OS=Homo sapiens GN=C8G PE=1 SV=3 | CO8G_HUMAN | 22 kDa |  | 53% | 8 | 18 | 28 |
| 406 | Coronin-1C OS=Homo sapiens GN=CORO1C PE=1 SV=1 | COR1C_HUMAN | 53 kDa | TRUE | 35% | 15 | 23 | 35 |
| 407 | IgGFc-binding protein OS=Homo sapiens GN=FCGBP PE=1 SV=3 | FCGBP_HUMAN | 572 kDa | TRUE | 4.20% | 20 | 25 | 30 |
| 408 | Grancalcin OS=Homo sapiens GN=GCA PE=1 SV=2 | GRAN_HUMAN | 24 kDa |  | 43% | 8 | 15 | 26 |
| 409 | Glutathione S-transferase P OS=Homo sapiens GN=GSTP1 PE=1 SV=2 | GSTP1_HUMAN | 23 kDa |  | 59% | 11 | 21 | 25 |
| 410 | Microtubule-associated protein RP/EB family member 1 OS=Homo sapiens GN=MAPRE1 PE=1 SV=3 | MARE1_HUMAN | 30 kDa | TRUE | 52% | 12 | 17 | 20 |
| 411 | Putative neutrophil cytosol factor 1B OS=Homo sapiens GN=NCF1B PE=5 SV=2 | NCF1B_HUMAN | 45 kDa | TRUE | 35% | 11 | 24 | 26 |
| 412 | Nucleoside diphosphate kinase B OS=Homo sapiens GN=NME2 PE=1 SV=1 | NDKB_HUMAN | 17 kDa | TRUE | 74% | 10 | 18 | 27 |
| 413 | Protein Niban OS=Homo sapiens GN=FAM129A PE=1 SV=1 | NIBAN_HUMAN | 103 kDa |  | 24% | 17 | 22 | 25 |
| 414 | Pigment epithelium-derived factor OS=Homo sapiens GN=SERPINF1 PE=1 SV=4 | PEDF_HUMAN | 46 kDa | TRUE | 42% | 15 | 20 | 23 |
| 415 | Nicotinate phosphoribosyltransferase OS=Homo sapiens GN=NAPRT PE=1 SV=2 | PNCB_HUMAN | 58 kDa |  | 34% | 13 | 20 | 25 |
| 416 | Proteasome subunit beta type-1 OS=Homo sapiens GN=PSMB1 PE=1 SV=2 | PSB1_HUMAN | 26 kDa |  | 64% | 10 | 18 | 28 |
| 417 | Proteasome subunit beta type-4 OS=Homo sapiens GN=PSMB4 PE=1 SV=4 | PSB4_HUMAN | 29 kDa | TRUE | 48% | 8 | 17 | 27 |
| 418 | Staphylococcal nuclease domain-containing protein 1 OS=Homo sapiens GN=SND1 PE=1 SV=1 | SND1_HUMAN | 102 kDa |  | 28% | 20 | 23 | 27 |
| 419 | Alanine--tRNA ligase, cytoplasmic OS=Homo sapiens GN=AARS PE=1 SV=2 | SYAC_HUMAN | 107 kDa |  | 30% | 22 | 26 | 27 |
| 420 | Ig kappa chain V-I region DEE OS=Homo sapiens PE=1 SV=1 | KV105_HUMAN | 12 kDa | TRUE | 27% | 3 | 6 | 32 |
| 421 | Ig heavy chain V-III region BRO OS=Homo sapiens PE=1 SV=1 | HV305_HUMAN | 13 kDa | TRUE | 34% | 2 | 4 | 35 |
| 422 | Bridging integrator 2 OS=Homo sapiens GN=BIN2 PE=1 SV=3 | BIN2_HUMAN | 62 kDa |  | 41% | 18 | 22 | 25 |
| 423 | Hsp90 co-chaperone Cdc37 OS=Homo sapiens GN=CDC37 PE=1 SV=1 | CDC37_HUMAN | 44 kDa | TRUE | 33% | 10 | 20 | 26 |
| 424 | Coatomer subunit delta OS=Homo sapiens GN=ARCN1 PE=1 SV=1 | COPD_HUMAN | 57 kDa |  | 33% | 17 | 26 | 28 |
| 425 | Rab GDP dissociation inhibitor beta OS=Homo sapiens GN=GDI2 PE=1 SV=2 | GDIB_HUMAN | 51 kDa | TRUE | 45% | 15 | 20 | 28 |
| 426 | Haptoglobin-related protein OS=Homo sapiens GN=HPR PE=2 SV=2 | HPTR_HUMAN | 39 kDa | TRUE | 62% | 9 | 15 | 92 |
| 427 | Lamin-B2 OS=Homo sapiens GN=LMNB2 PE=1 SV=4 | LMNB2_HUMAN | 70 kDa | TRUE | 32% | 17 | 21 | 30 |
| 428 | N-acetyl-D-glucosamine kinase OS=Homo sapiens GN=NAGK PE=1 SV=4 | NAGK_HUMAN | 37 kDa |  | 45% | 14 | 20 | 27 |
| 429 | Peroxiredoxin-1 OS=Homo sapiens GN=PRDX1 PE=1 SV=1 | PRDX1_HUMAN | 22 kDa | TRUE | 42% | 8 | 15 | 25 |
| 430 | Proteasome subunit beta type-7 OS=Homo sapiens GN=PSMB7 PE=1 SV=1 | PSB7_HUMAN | 30 kDa |  | 21% | 5 | 15 | 26 |
| 431 | 26S proteasome non-ATPase regulatory subunit 11 OS=Homo sapiens GN=PSMD11 PE=1 SV=3 | PSD11_HUMAN | 47 kDa |  | 41% | 15 | 19 | 24 |
| 432 | 26S proteasome non-ATPase regulatory subunit 6 OS=Homo sapiens GN=PSMD6 PE=1 SV=1 | PSMD6_HUMAN | 46 kDa |  | 32% | 12 | 15 | 21 |
| 433 | Peroxidasin homolog OS=Homo sapiens GN=PXDN PE=1 SV=2 | PXDN_HUMAN | 165 kDa |  | 13% | 17 | 21 | 23 |
| 434 | Selenoprotein P OS=Homo sapiens GN=SEPP1 PE=1 SV=3 | SEPP1_HUMAN | 43 kDa | TRUE | 23% | 8 | 13 | 16 |
| 435 | Septin-7 OS=Homo sapiens GN=SEPT7 PE=1 SV=2 | SEPT7_HUMAN | 51 kDa | TRUE | 29% | 13 | 14 | 20 |
| 436 | Adipocyte plasma membrane-associated protein OS=Homo sapiens GN=APMAP PE=1 SV=2 | APMAP_HUMAN | 46 kDa |  | 34% | 13 | 23 | 26 |
| 437 | Apolipoprotein C-I OS=Homo sapiens GN=APOC1 PE=1 SV=1 | APOC1_HUMAN | 9 kDa | TRUE | 35% | 5 | 10 | 26 |
| 438 | Cell division control protein 42 homolog OS=Homo sapiens GN=CDC42 PE=1 SV=2 | CDC42_HUMAN | 21 kDa | TRUE | 48% | 7 | 16 | 26 |
| 439 | Probable ATP-dependent RNA helicase DDX17 OS=Homo sapiens GN=DDX17 PE=1 SV=2 | DDX17_HUMAN | 80 kDa | TRUE | 21% | 14 | 21 | 25 |
| 440 | Serine/threonine-protein kinase OSR1 OS=Homo sapiens GN=OXSR1 PE=1 SV=1 | OXSR1_HUMAN | 58 kDa | TRUE | 32% | 13 | 18 | 21 |
| 441 | Poly(rC)-binding protein 1 OS=Homo sapiens GN=PCBP1 PE=1 SV=2 | PCBP1_HUMAN | 37 kDa | TRUE | 42% | 11 | 18 | 23 |
| 442 | 26S proteasome non-ATPase regulatory subunit 13 OS=Homo sapiens GN=PSMD13 PE=1 SV=2 | PSD13_HUMAN | 43 kDa |  | 46% | 15 | 19 | 25 |
| 443 | Polypyrimidine tract-binding protein 1 OS=Homo sapiens GN=PTBP1 PE=1 SV=1 | PTBP1_HUMAN | 57 kDa | TRUE | 22% | 10 | 17 | 22 |
| 444 | Tropomodulin-3 OS=Homo sapiens GN=TMOD3 PE=1 SV=1 | TMOD3_HUMAN | 40 kDa | TRUE | 52% | 13 | 20 | 24 |
| 445 | COP9 signalosome complex subunit 5 OS=Homo sapiens GN=COPS5 PE=1 SV=4 | CSN5_HUMAN | 38 kDa |  | 51% | 12 | 18 | 19 |
| 446 | Eukaryotic translation initiation factor 3 subunit B OS=Homo sapiens GN=EIF3B PE=1 SV=3 | EIF3B_HUMAN | 92 kDa | TRUE | 25% | 15 | 16 | 19 |
| 447 | Glia maturation factor gamma OS=Homo sapiens GN=GMFG PE=1 SV=1 | GMFG_HUMAN | 17 kDa | TRUE | 44% | 6 | 15 | 20 |
| 448 | Heterogeneous nuclear ribonucleoprotein M OS=Homo sapiens GN=HNRNPM PE=1 SV=3 | HNRPM_HUMAN | 78 kDa |  | 25% | 16 | 19 | 22 |
| 449 | Integrin-linked protein kinase OS=Homo sapiens GN=ILK PE=1 SV=2 | ILK_HUMAN | 51 kDa |  | 32% | 13 | 22 | 23 |
| 450 | Protein NDRG1 OS=Homo sapiens GN=NDRG1 PE=1 SV=1 | NDRG1_HUMAN | 43 kDa | TRUE | 32% | 8 | 18 | 27 |
| 451 | Nidogen-1 OS=Homo sapiens GN=NID1 PE=1 SV=3 | NID1_HUMAN | 136 kDa | TRUE | 17% | 18 | 27 | 29 |
| 452 | Nucleolin OS=Homo sapiens GN=NCL PE=1 SV=3 | NUCL_HUMAN | 77 kDa | TRUE | 21% | 14 | 19 | 19 |
| 453 | 26S proteasome non-ATPase regulatory subunit 5 OS=Homo sapiens GN=PSMD5 PE=1 SV=3 | PSMD5_HUMAN | 56 kDa |  | 46% | 17 | 22 | 26 |
| 454 | 60S acidic ribosomal protein P0 OS=Homo sapiens GN=RPLP0 PE=1 SV=1 | RLA0_HUMAN | 34 kDa |  | 35% | 9 | 19 | 21 |
| 455 | Rho-associated protein kinase 1 OS=Homo sapiens GN=ROCK1 PE=1 SV=1 | ROCK1_HUMAN | 158 kDa | TRUE | 16% | 17 | 18 | 21 |
| 456 | Protein S100-A4 OS=Homo sapiens GN=S100A4 PE=1 SV=1 | S10A4_HUMAN | 12 kDa | TRUE | 37% | 5 | 14 | 26 |
| 457 | V-type proton ATPase subunit E 1 OS=Homo sapiens GN=ATP6V1E1 PE=1 SV=1 | VATE1_HUMAN | 26 kDa |  | 48% | 12 | 20 | 27 |
| 458 | Ig kappa chain V-I region Roy OS=Homo sapiens PE=1 SV=1 | KV116_HUMAN | 12 kDa |  | 24% | 2 | 5 | 27 |
| 459 | Arf-GAP with coiled-coil, ANK repeat and PH domain-containing protein 2 OS=Homo sapiens GN=ACAP2 PE=1 SV=3 | ACAP2_HUMAN | 88 kDa | TRUE | 25% | 13 | 18 | 22 |
| 460 | ADP-ribosylation factor 3 OS=Homo sapiens GN=ARF3 PE=1 SV=2 | ARF3_HUMAN | 21 kDa | TRUE | 47% | 9 | 14 | 18 |
| 461 | Argininosuccinate lyase OS=Homo sapiens GN=ASL PE=1 SV=4 | ARLY_HUMAN | 52 kDa | TRUE | 23% | 10 | 14 | 18 |
| 462 | Calpain small subunit 1 OS=Homo sapiens GN=CAPNS1 PE=1 SV=1 | CPNS1_HUMAN | 28 kDa |  | 37% | 8 | 14 | 23 |
| 463 | COP9 signalosome complex subunit 4 OS=Homo sapiens GN=COPS4 PE=1 SV=1 | CSN4_HUMAN | 46 kDa | TRUE | 43% | 12 | 17 | 18 |
| 464 | Complement factor H-related protein 1 OS=Homo sapiens GN=CFHR1 PE=1 SV=2 | FHR1_HUMAN | 38 kDa | TRUE | 38% | 5 | 10 | 56 |
| 465 | Guanine nucleotide-binding protein subunit beta-2-like 1 OS=Homo sapiens GN=GNB2L1 PE=1 SV=3 | GBLP_HUMAN | 35 kDa |  | 33% | 11 | 19 | 21 |
| 466 | Heterogeneous nuclear ribonucleoprotein D0 OS=Homo sapiens GN=HNRNPD PE=1 SV=1 | HNRPD_HUMAN | 38 kDa | TRUE | 26% | 9 | 18 | 21 |
| 467 | Keratin, type II cytoskeletal 5 OS=Homo sapiens GN=KRT5 PE=1 SV=3 | K2C5_HUMAN | 62 kDa | TRUE | 31% | 8 | 16 | 49 |
| 468 | Peptidoglycan recognition protein 1 OS=Homo sapiens GN=PGLYRP1 PE=1 SV=1 | PGRP1_HUMAN | 22 kDa |  | 41% | 5 | 13 | 20 |
| 469 | Protein phosphatase 1 regulatory subunit 7 OS=Homo sapiens GN=PPP1R7 PE=1 SV=1 | PP1R7_HUMAN | 42 kDa |  | 54% | 15 | 19 | 23 |
| 470 | Proteasome subunit alpha type-4 OS=Homo sapiens GN=PSMA4 PE=1 SV=1 | PSA4_HUMAN | 29 kDa |  | 54% | 10 | 15 | 21 |
| 471 | Proteasome subunit beta type-8 OS=Homo sapiens GN=PSMB8 PE=1 SV=3 | PSB8_HUMAN | 30 kDa |  | 29% | 8 | 16 | 26 |
| 472 | 60S ribosomal protein L10a OS=Homo sapiens GN=RPL10A PE=1 SV=2 | RL10A_HUMAN | 25 kDa |  | 46% | 10 | 14 | 19 |
| 473 | Tubulin--tyrosine ligase-like protein 12 OS=Homo sapiens GN=TTLL12 PE=1 SV=2 | TTL12_HUMAN | 74 kDa |  | 35% | 15 | 19 | 23 |
| 474 | General vesicular transport factor p115 OS=Homo sapiens GN=USO1 PE=1 SV=2 | USO1_HUMAN | 108 kDa | TRUE | 16% | 13 | 18 | 22 |
| 475 | Ig lambda chain V-I region HA OS=Homo sapiens PE=1 SV=1 | LV102_HUMAN | 12 kDa | TRUE | 19% | 2 | 4 | 13 |
| 476 | Actin-related protein 2/3 complex subunit 5 OS=Homo sapiens GN=ARPC5 PE=1 SV=3 | ARPC5_HUMAN | 16 kDa | TRUE | 61% | 7 | 15 | 16 |
| 477 | Acid ceramidase OS=Homo sapiens GN=ASAH1 PE=1 SV=5 | ASAH1_HUMAN | 45 kDa |  | 28% | 12 | 16 | 20 |
| 478 | Calponin-2 OS=Homo sapiens GN=CNN2 PE=1 SV=4 | CNN2_HUMAN | 34 kDa |  | 47% | 10 | 16 | 16 |
| 479 | Cytokine receptor-like factor 3 OS=Homo sapiens GN=CRLF3 PE=1 SV=2 | CRLF3_HUMAN | 50 kDa | TRUE | 29% | 10 | 14 | 22 |
| 480 | Sorbitol dehydrogenase OS=Homo sapiens GN=SORD PE=1 SV=4 | DHSO_HUMAN | 38 kDa | TRUE | 25% | 8 | 15 | 18 |
| 481 | Ezrin OS=Homo sapiens GN=EZR PE=1 SV=4 | EZRI_HUMAN | 69 kDa | TRUE | 32% | 12 | 18 | 36 |
| 482 | Coagulation factor XI OS=Homo sapiens GN=F11 PE=1 SV=1 | FA11_HUMAN | 70 kDa |  | 21% | 12 | 19 | 22 |
| 483 | Growth factor receptor-bound protein 2 OS=Homo sapiens GN=GRB2 PE=1 SV=1 | GRB2_HUMAN | 25 kDa |  | 60% | 11 | 15 | 17 |
| 484 | Heterogeneous nuclear ribonucleoprotein F OS=Homo sapiens GN=HNRNPF PE=1 SV=3 | HNRPF_HUMAN | 46 kDa | TRUE | 32% | 9 | 16 | 21 |
| 485 | Ig heavy chain V-III region GAL OS=Homo sapiens PE=1 SV=1 | HV320_HUMAN | 13 kDa | TRUE | 48% | 5 | 9 | 13 |
| 486 | Interstitial collagenase OS=Homo sapiens GN=MMP1 PE=1 SV=3 | MMP1_HUMAN | 54 kDa |  | 34% | 15 | 22 | 24 |
| 487 | Ig mu heavy chain disease protein OS=Homo sapiens PE=1 SV=1 | MUCB_HUMAN | 43 kDa | TRUE | 67% | 4 | 6 | 328 |
| 488 | Neutrophil cytosol factor 4 OS=Homo sapiens GN=NCF4 PE=1 SV=2 | NCF4_HUMAN | 39 kDa | TRUE | 47% | 13 | 17 | 21 |
| 489 | Protein-arginine deiminase type-2 OS=Homo sapiens GN=PADI2 PE=1 SV=2 | PADI2_HUMAN | 76 kDa |  | 28% | 16 | 23 | 26 |
| 490 | Protein-L-isoaspartate(D-aspartate) O-methyltransferase OS=Homo sapiens GN=PCMT1 PE=1 SV=4 | PIMT_HUMAN | 25 kDa |  | 65% | 13 | 16 | 22 |
| 491 | Pleckstrin OS=Homo sapiens GN=PLEK PE=1 SV=3 | PLEK_HUMAN | 40 kDa | TRUE | 35% | 7 | 11 | 16 |
| 492 | Proteasome subunit beta type-5 OS=Homo sapiens GN=PSMB5 PE=1 SV=3 | PSB5_HUMAN | 28 kDa | TRUE | 35% | 9 | 15 | 21 |
| 493 | 40S ribosomal protein S3a OS=Homo sapiens GN=RPS3A PE=1 SV=2 | RS3A_HUMAN | 30 kDa |  | 38% | 9 | 12 | 14 |
| 494 | Protein transport protein Sec23A OS=Homo sapiens GN=SEC23A PE=1 SV=2 | SC23A_HUMAN | 86 kDa | TRUE | 18% | 12 | 18 | 19 |
| 495 | Vasodilator-stimulated phosphoprotein OS=Homo sapiens GN=VASP PE=1 SV=3 | VASP_HUMAN | 40 kDa |  | 38% | 13 | 19 | 22 |
| 496 | Ig kappa chain V-I region EU OS=Homo sapiens PE=1 SV=1 | KV106_HUMAN | 12 kDa | TRUE | 27% | 2 | 6 | 21 |
| 497 | Coatomer subunit gamma-1 OS=Homo sapiens GN=COPG1 PE=1 SV=1 | COPG1_HUMAN | 98 kDa | TRUE | 28% | 20 | 21 | 23 |
| 498 | Glycogen [starch] synthase, muscle OS=Homo sapiens GN=GYS1 PE=1 SV=2 | GYS1_HUMAN | 84 kDa | TRUE | 21% | 12 | 16 | 21 |
| 499 | Heterogeneous nuclear ribonucleoprotein U-like protein 2 OS=Homo sapiens GN=HNRNPUL2 PE=1 SV=1 | HNRL2_HUMAN | 85 kDa | TRUE | 18% | 11 | 19 | 19 |
| 500 | Immunoglobulin J chain OS=Homo sapiens GN=JCHAIN PE=1 SV=4 | IGJ_HUMAN | 18 kDa |  | 43% | 7 | 11 | 22 |
| 501 | Kallistatin OS=Homo sapiens GN=SERPINA4 PE=1 SV=3 | KAIN_HUMAN | 49 kDa |  | 37% | 12 | 16 | 21 |
| 502 | Galectin-1 OS=Homo sapiens GN=LGALS1 PE=1 SV=2 | LEG1_HUMAN | 15 kDa |  | 53% | 7 | 13 | 22 |
| 503 | Non-POU domain-containing octamer-binding protein OS=Homo sapiens GN=NONO PE=1 SV=4 | NONO_HUMAN | 54 kDa | TRUE | 24% | 10 | 14 | 17 |
| 504 | Protein kinase C and casein kinase substrate in neurons protein 2 OS=Homo sapiens GN=PACSIN2 PE=1 SV=2 | PACN2_HUMAN | 56 kDa |  | 29% | 15 | 20 | 24 |
| 505 | 26S proteasome non-ATPase regulatory subunit 14 OS=Homo sapiens GN=PSMD14 PE=1 SV=1 | PSDE_HUMAN | 35 kDa |  | 28% | 8 | 15 | 20 |
| 506 | tRNA-splicing ligase RtcB homolog OS=Homo sapiens GN=RTCB PE=1 SV=1 | RTCB_HUMAN | 55 kDa | TRUE | 27% | 12 | 17 | 18 |
| 507 | Protein transport protein Sec31A OS=Homo sapiens GN=SEC31A PE=1 SV=3 | SC31A_HUMAN | 133 kDa |  | 14% | 13 | 18 | 20 |
| 508 | Structural maintenance of chromosomes protein 3 OS=Homo sapiens GN=SMC3 PE=1 SV=2 | SMC3_HUMAN | 142 kDa | TRUE | 17% | 16 | 16 | 17 |
| 509 | Thrombospondin-2 OS=Homo sapiens GN=THBS2 PE=1 SV=2 | TSP2_HUMAN | 130 kDa | TRUE | 14% | 10 | 13 | 20 |
| 510 | Alpha-1-acid glycoprotein 1 OS=Homo sapiens GN=ORM1 PE=1 SV=1 | A1AG1_HUMAN | 24 kDa | TRUE | 48% | 5 | 13 | 31 |
| 511 | Protein arginine N-methyltransferase 5 OS=Homo sapiens GN=PRMT5 PE=1 SV=4 | ANM5_HUMAN | 73 kDa |  | 32% | 13 | 17 | 18 |
| 512 | AP-2 complex subunit mu OS=Homo sapiens GN=AP2M1 PE=1 SV=2 | AP2M1_HUMAN | 50 kDa |  | 27% | 12 | 19 | 20 |
| 513 | Actin-related protein 2/3 complex subunit 4 OS=Homo sapiens GN=ARPC4 PE=1 SV=3 | ARPC4_HUMAN | 20 kDa |  | 64% | 8 | 15 | 22 |
| 514 | Carbonic anhydrase 1 OS=Homo sapiens GN=CA1 PE=1 SV=2 | CAH1_HUMAN | 29 kDa |  | 52% | 10 | 16 | 22 |
| 515 | Collagen alpha-1(V) chain OS=Homo sapiens GN=COL5A1 PE=1 SV=3 | CO5A1_HUMAN | 184 kDa |  | 8.50% | 12 | 17 | 20 |
| 516 | Eukaryotic peptide chain release factor GTP-binding subunit ERF3A OS=Homo sapiens GN=GSPT1 PE=1 SV=1 | ERF3A_HUMAN | 56 kDa |  | 20% | 10 | 13 | 13 |
| 517 | Hypoxanthine-guanine phosphoribosyltransferase OS=Homo sapiens GN=HPRT1 PE=1 SV=2 | HPRT_HUMAN | 25 kDa |  | 65% | 10 | 15 | 24 |
| 518 | Eukaryotic translation initiation factor 2 subunit 3 OS=Homo sapiens GN=EIF2S3 PE=1 SV=3 | IF2G_HUMAN | 51 kDa | TRUE | 32% | 11 | 14 | 14 |
| 519 | Ribosomal protein S6 kinase alpha-3 OS=Homo sapiens GN=RPS6KA3 PE=1 SV=1 | KS6A3_HUMAN | 84 kDa | TRUE | 8.80% | 7 | 12 | 13 |
| 520 | Laminin subunit alpha-4 OS=Homo sapiens GN=LAMA4 PE=1 SV=4 | LAMA4_HUMAN | 203 kDa |  | 7.40% | 11 | 13 | 14 |
| 521 | Ig lambda chain V-III region LOI OS=Homo sapiens PE=1 SV=1 | LV302_HUMAN | 12 kDa |  | 46% | 4 | 7 | 26 |
| 522 | Phosphoglucomutase-1 OS=Homo sapiens GN=PGM1 PE=1 SV=3 | PGM1_HUMAN | 61 kDa |  | 25% | 12 | 14 | 17 |
| 523 | Ras-related protein Rab-7a OS=Homo sapiens GN=RAB7A PE=1 SV=1 | RAB7A_HUMAN | 23 kDa |  | 61% | 12 | 17 | 19 |
| 524 | Ras-related protein Rab-11B OS=Homo sapiens GN=RAB11B PE=1 SV=4 | RB11B_HUMAN | 24 kDa |  | 50% | 11 | 18 | 23 |
| 525 | 60S ribosomal protein L3 OS=Homo sapiens GN=RPL3 PE=1 SV=2 | RL3_HUMAN | 46 kDa | TRUE | 28% | 12 | 14 | 16 |
| 526 | RuvB-like 1 OS=Homo sapiens GN=RUVBL1 PE=1 SV=1 | RUVB1_HUMAN | 50 kDa |  | 40% | 14 | 21 | 26 |
| 527 | Protein S100-A12 OS=Homo sapiens GN=S100A12 PE=1 SV=2 | S10AC_HUMAN | 11 kDa |  | 86% | 8 | 13 | 17 |
| 528 | Structural maintenance of chromosomes protein 1A OS=Homo sapiens GN=SMC1A PE=1 SV=2 | SMC1A_HUMAN | 143 kDa | TRUE | 9.30% | 10 | 10 | 10 |
| 529 | Serine/arginine-rich splicing factor 1 OS=Homo sapiens GN=SRSF1 PE=1 SV=2 | SRSF1_HUMAN | 28 kDa | TRUE | 39% | 9 | 10 | 12 |
| 530 | Ig kappa chain V-I region WEA OS=Homo sapiens PE=1 SV=1 | KV118_HUMAN | 12 kDa | TRUE | 31% | 3 | 6 | 27 |
| 531 | Rho guanine nucleotide exchange factor 1 OS=Homo sapiens GN=ARHGEF1 PE=1 SV=2 | ARHG1_HUMAN | 102 kDa | TRUE | 17% | 14 | 19 | 20 |
| 532 | Cathepsin D OS=Homo sapiens GN=CTSD PE=1 SV=1 | CATD_HUMAN | 45 kDa | TRUE | 30% | 10 | 13 | 17 |
| 533 | Complement C4-A OS=Homo sapiens GN=C4A PE=1 SV=2 | CO4A_HUMAN | 193 kDa | TRUE | 76% | 4 | 8 | 1580 |
| 534 | Cartilage oligomeric matrix protein OS=Homo sapiens GN=COMP PE=1 SV=2 | COMP_HUMAN | 83 kDa | TRUE | 16% | 9 | 12 | 17 |
| 535 | Coagulation factor X OS=Homo sapiens GN=F10 PE=1 SV=2 | FA10_HUMAN | 55 kDa | TRUE | 17% | 8 | 15 | 17 |
| 536 | Complement factor H-related protein 3 OS=Homo sapiens GN=CFHR3 PE=1 SV=2 | FHR3_HUMAN | 37 kDa | TRUE | 30% | 7 | 13 | 25 |
| 537 | Far upstream element-binding protein 2 OS=Homo sapiens GN=KHSRP PE=1 SV=4 | FUBP2_HUMAN | 73 kDa | TRUE | 23% | 14 | 17 | 19 |
| 538 | Guanylate-binding protein 1 OS=Homo sapiens GN=GBP1 PE=1 SV=2 | GBP1_HUMAN | 68 kDa | TRUE | 21% | 12 | 15 | 15 |
| 539 | Glutamine synthetase OS=Homo sapiens GN=GLUL PE=1 SV=4 | GLNA_HUMAN | 42 kDa | TRUE | 25% | 8 | 10 | 15 |
| 540 | Glutathione peroxidase 1 OS=Homo sapiens GN=GPX1 PE=1 SV=4 | GPX1_HUMAN | 22 kDa |  | 61% | 10 | 19 | 23 |
| 541 | Calpastatin OS=Homo sapiens GN=CAST PE=1 SV=4 | ICAL_HUMAN | 77 kDa |  | 20% | 9 | 13 | 14 |
| 542 | Eukaryotic translation initiation factor 2 subunit 1 OS=Homo sapiens GN=EIF2S1 PE=1 SV=3 | IF2A_HUMAN | 36 kDa |  | 46% | 13 | 17 | 21 |
| 543 | Protein kinase C delta type OS=Homo sapiens GN=PRKCD PE=1 SV=2 | KPCD_HUMAN | 78 kDa |  | 16% | 11 | 14 | 15 |
| 544 | Galectin-3 OS=Homo sapiens GN=LGALS3 PE=1 SV=5 | LEG3_HUMAN | 26 kDa |  | 32% | 8 | 13 | 18 |
| 545 | Protein deglycase DJ-1 OS=Homo sapiens GN=PARK7 PE=1 SV=2 | PARK7_HUMAN | 20 kDa |  | 54% | 11 | 19 | 25 |
| 546 | Prenylcysteine oxidase 1 OS=Homo sapiens GN=PCYOX1 PE=1 SV=3 | PCYOX_HUMAN | 57 kDa |  | 22% | 8 | 8 | 8 |
| 547 | Peroxiredoxin-5, mitochondrial OS=Homo sapiens GN=PRDX5 PE=1 SV=4 | PRDX5_HUMAN | 22 kDa |  | 56% | 10 | 18 | 25 |
| 548 | Proteasome subunit beta type-3 OS=Homo sapiens GN=PSMB3 PE=1 SV=2 | PSB3_HUMAN | 23 kDa |  | 43% | 9 | 14 | 16 |
| 549 | Rho-related GTP-binding protein RhoG OS=Homo sapiens GN=RHOG PE=1 SV=1 | RHOG_HUMAN | 21 kDa | TRUE | 51% | 9 | 15 | 20 |
| 550 | 60S ribosomal protein L4 OS=Homo sapiens GN=RPL4 PE=1 SV=5 | RL4_HUMAN | 48 kDa | TRUE | 29% | 11 | 14 | 16 |
| 551 | Serine/threonine-protein kinase 4 OS=Homo sapiens GN=STK4 PE=1 SV=2 | STK4_HUMAN | 56 kDa |  | 35% | 14 | 20 | 23 |
| 552 | Cullin-4B OS=Homo sapiens GN=CUL4B PE=1 SV=4 | CUL4B_HUMAN | 104 kDa | TRUE | 8.90% | 2 | 2 | 8 |
| 553 | Ig kappa chain V-I region AU OS=Homo sapiens PE=1 SV=1 | KV102_HUMAN | 12 kDa |  | 31% | 2 | 5 | 26 |
| 554 | SH3 domain-binding protein 1 OS=Homo sapiens GN=SH3BP1 PE=1 SV=3 | 3BP1_HUMAN | 76 kDa | TRUE | 20% | 12 | 16 | 18 |
| 555 | Monocyte differentiation antigen CD14 OS=Homo sapiens GN=CD14 PE=1 SV=2 | CD14_HUMAN | 40 kDa |  | 37% | 12 | 17 | 23 |
| 556 | COP9 signalosome complex subunit 1 OS=Homo sapiens GN=GPS1 PE=1 SV=4 | CSN1_HUMAN | 56 kDa | TRUE | 26% | 8 | 10 | 11 |
| 557 | Peptidyl-prolyl cis-trans isomerase FKBP4 OS=Homo sapiens GN=FKBP4 PE=1 SV=3 | FKBP4_HUMAN | 52 kDa | TRUE | 29% | 9 | 13 | 13 |
| 558 | Guanine nucleotide-binding protein G(I)/G(S)/G(T) subunit beta-2 OS=Homo sapiens GN=GNB2 PE=1 SV=3 | GBB2_HUMAN | 37 kDa | TRUE | 28% | 7 | 12 | 19 |
| 559 | Beta-hexosaminidase subunit beta OS=Homo sapiens GN=HEXB PE=1 SV=3 | HEXB_HUMAN | 63 kDa |  | 22% | 10 | 14 | 15 |
| 560 | Interleukin enhancer-binding factor 2 OS=Homo sapiens GN=ILF2 PE=1 SV=2 | ILF2_HUMAN | 43 kDa |  | 50% | 13 | 16 | 19 |
| 561 | Plasma serine protease inhibitor OS=Homo sapiens GN=SERPINA5 PE=1 SV=3 | IPSP_HUMAN | 46 kDa |  | 37% | 12 | 16 | 21 |
| 562 | Niban-like protein 1 OS=Homo sapiens GN=FAM129B PE=1 SV=3 | NIBL1_HUMAN | 84 kDa |  | 16% | 10 | 13 | 14 |
| 563 | ADP-sugar pyrophosphatase OS=Homo sapiens GN=NUDT5 PE=1 SV=1 | NUDT5_HUMAN | 24 kDa |  | 37% | 8 | 16 | 23 |
| 564 | Proliferating cell nuclear antigen OS=Homo sapiens GN=PCNA PE=1 SV=1 | PCNA_HUMAN | 29 kDa |  | 50% | 11 | 17 | 19 |
| 565 | Low molecular weight phosphotyrosine protein phosphatase OS=Homo sapiens GN=ACP1 PE=1 SV=3 | PPAC_HUMAN | 18 kDa |  | 65% | 8 | 14 | 23 |
| 566 | Proteasome activator complex subunit 2 OS=Homo sapiens GN=PSME2 PE=1 SV=4 | PSME2_HUMAN | 27 kDa | TRUE | 43% | 8 | 14 | 16 |
| 567 | Lysine--tRNA ligase OS=Homo sapiens GN=KARS PE=1 SV=3 | SYK_HUMAN | 68 kDa |  | 21% | 12 | 15 | 16 |
| 568 | Exportin-2 OS=Homo sapiens GN=CSE1L PE=1 SV=3 | XPO2_HUMAN | 110 kDa |  | 14% | 10 | 10 | 11 |
| 569 | Ig kappa chain V-I region Scw OS=Homo sapiens PE=1 SV=1 | KV117_HUMAN | 12 kDa |  | 28% | 2 | 5 | 26 |
| 570 | Amyloid beta A4 precursor protein-binding family B member 1-interacting protein OS=Homo sapiens GN=APBB1IP PE=1 SV=1 | AB1IP_HUMAN | 73 kDa | TRUE | 16% | 9 | 12 | 12 |
| 571 | Actin-related protein 2/3 complex subunit 3 OS=Homo sapiens GN=ARPC3 PE=1 SV=3 | ARPC3_HUMAN | 21 kDa |  | 33% | 7 | 11 | 13 |
| 572 | Macrophage-capping protein OS=Homo sapiens GN=CAPG PE=1 SV=2 | CAPG_HUMAN | 38 kDa |  | 41% | 11 | 18 | 22 |
| 573 | Complement C2 OS=Homo sapiens GN=C2 PE=1 SV=2 | CO2_HUMAN | 83 kDa |  | 15% | 11 | 16 | 18 |
| 574 | COP9 signalosome complex subunit 3 OS=Homo sapiens GN=COPS3 PE=1 SV=3 | CSN3_HUMAN | 48 kDa |  | 29% | 10 | 14 | 16 |
| 575 | Deoxyribose-phosphate aldolase OS=Homo sapiens GN=DERA PE=1 SV=2 | DEOC_HUMAN | 35 kDa | TRUE | 51% | 12 | 17 | 20 |
| 576 | KH domain-containing, RNA-binding, signal transduction-associated protein 1 OS=Homo sapiens GN=KHDRBS1 PE=1 SV=1 | KHDR1_HUMAN | 48 kDa |  | 12% | 4 | 8 | 10 |
| 577 | LIM and SH3 domain protein 1 OS=Homo sapiens GN=LASP1 PE=1 SV=2 | LASP1_HUMAN | 30 kDa | TRUE | 41% | 10 | 17 | 20 |
| 578 | Protein phosphatase 1B OS=Homo sapiens GN=PPM1B PE=1 SV=1 | PPM1B_HUMAN | 53 kDa | TRUE | 18% | 8 | 13 | 16 |
| 579 | Myeloblastin OS=Homo sapiens GN=PRTN3 PE=1 SV=3 | PRTN3_HUMAN | 28 kDa |  | 33% | 6 | 9 | 24 |
| 580 | Rho GTPase-activating protein 4 OS=Homo sapiens GN=ARHGAP4 PE=1 SV=2 | RHG04_HUMAN | 105 kDa |  | 16% | 13 | 16 | 17 |
| 581 | Heterogeneous nuclear ribonucleoprotein A1 OS=Homo sapiens GN=HNRNPA1 PE=1 SV=5 | ROA1_HUMAN | 39 kDa | TRUE | 25% | 7 | 12 | 25 |
| 582 | Serpin H1 OS=Homo sapiens GN=SERPINH1 PE=1 SV=2 | SERPH_HUMAN | 46 kDa |  | 38% | 13 | 18 | 18 |
| 583 | Sorting nexin-1 OS=Homo sapiens GN=SNX1 PE=1 SV=3 | SNX1_HUMAN | 59 kDa | TRUE | 28% | 12 | 18 | 19 |
| 584 | Asparagine--tRNA ligase, cytoplasmic OS=Homo sapiens GN=NARS PE=1 SV=1 | SYNC_HUMAN | 63 kDa | TRUE | 19% | 9 | 15 | 16 |
| 585 | Carbonic anhydrase 2 OS=Homo sapiens GN=CA2 PE=1 SV=2 | CAH2_HUMAN | 29 kDa |  | 39% | 9 | 13 | 16 |
| 586 | Cholesteryl ester transfer protein OS=Homo sapiens GN=CETP PE=1 SV=2 | CETP_HUMAN | 55 kDa |  | 21% | 9 | 12 | 17 |
| 587 | Collagen alpha-2(VI) chain OS=Homo sapiens GN=COL6A2 PE=1 SV=4 | CO6A2_HUMAN | 109 kDa |  | 12% | 10 | 11 | 13 |
| 588 | Coronin-1B OS=Homo sapiens GN=CORO1B PE=1 SV=1 | COR1B_HUMAN | 54 kDa | TRUE | 31% | 12 | 13 | 14 |
| 589 | Protein FAM49B OS=Homo sapiens GN=FAM49B PE=1 SV=1 | FA49B_HUMAN | 37 kDa |  | 48% | 12 | 16 | 21 |
| 590 | Formin-binding protein 1 OS=Homo sapiens GN=FNBP1 PE=1 SV=2 | FNBP1_HUMAN | 71 kDa |  | 20% | 12 | 15 | 16 |
| 591 | Far upstream element-binding protein 1 OS=Homo sapiens GN=FUBP1 PE=1 SV=3 | FUBP1_HUMAN | 68 kDa | TRUE | 19% | 9 | 12 | 20 |
| 592 | Glycogenin-1 OS=Homo sapiens GN=GYG1 PE=1 SV=4 | GLYG_HUMAN | 39 kDa |  | 19% | 8 | 14 | 19 |
| 593 | Glutathione S-transferase omega-1 OS=Homo sapiens GN=GSTO1 PE=1 SV=2 | GSTO1_HUMAN | 28 kDa |  | 37% | 9 | 14 | 20 |
| 594 | Hepatoma-derived growth factor OS=Homo sapiens GN=HDGF PE=1 SV=1 | HDGF_HUMAN | 27 kDa | TRUE | 40% | 9 | 15 | 15 |
| 595 | Minor histocompatibility protein HA-1 OS=Homo sapiens GN=HMHA1 PE=1 SV=2 | HMHA1_HUMAN | 125 kDa |  | 12% | 12 | 13 | 14 |
| 596 | Hypoxia up-regulated protein 1 OS=Homo sapiens GN=HYOU1 PE=1 SV=1 | HYOU1_HUMAN | 111 kDa | TRUE | 21% | 13 | 15 | 16 |
| 597 | Keratin, type I cytoskeletal 16 OS=Homo sapiens GN=KRT16 PE=1 SV=4 | K1C16_HUMAN | 51 kDa | TRUE | 42% | 12 | 15 | 42 |
| 598 | cAMP-dependent protein kinase type I-alpha regulatory subunit OS=Homo sapiens GN=PRKAR1A PE=1 SV=1 | KAP0_HUMAN | 43 kDa | TRUE | 29% | 10 | 15 | 19 |
| 599 | Voltage-gated potassium channel subunit beta-2 OS=Homo sapiens GN=KCNAB2 PE=1 SV=2 | KCAB2_HUMAN | 41 kDa |  | 39% | 10 | 14 | 15 |
| 600 | Serine/threonine-protein kinase PAK 2 OS=Homo sapiens GN=PAK2 PE=1 SV=3 | PAK2_HUMAN | 58 kDa | TRUE | 30% | 11 | 18 | 18 |
| 601 | Proprotein convertase subtilisin/kexin type 9 OS=Homo sapiens GN=PCSK9 PE=1 SV=3 | PCSK9_HUMAN | 74 kDa |  | 21% | 11 | 15 | 18 |
| 602 | Proteasome subunit beta type-6 OS=Homo sapiens GN=PSMB6 PE=1 SV=4 | PSB6_HUMAN | 25 kDa |  | 49% | 9 | 11 | 19 |
| 603 | 60S ribosomal protein L6 OS=Homo sapiens GN=RPL6 PE=1 SV=3 | RL6_HUMAN | 33 kDa | TRUE | 30% | 10 | 14 | 17 |
| 604 | U1 small nuclear ribonucleoprotein 70 kDa OS=Homo sapiens GN=SNRNP70 PE=1 SV=2 | RU17_HUMAN | 52 kDa |  | 19% | 10 | 13 | 15 |
| 605 | Septin-6 OS=Homo sapiens GN=SEPT6 PE=1 SV=4 | SEPT6_HUMAN | 50 kDa | TRUE | 22% | 8 | 14 | 17 |
| 606 | Splicing factor 3B subunit 3 OS=Homo sapiens GN=SF3B3 PE=1 SV=4 | SF3B3_HUMAN | 136 kDa |  | 13% | 13 | 13 | 15 |
| 607 | Glycine--tRNA ligase OS=Homo sapiens GN=GARS PE=1 SV=3 | SYG_HUMAN | 83 kDa | TRUE | 16% | 10 | 13 | 15 |
| 608 | Nesprin-1 OS=Homo sapiens GN=SYNE1 PE=1 SV=4 | SYNE1_HUMAN | 1011 kDa | TRUE | 0.35% | 2 | 2 | 2 |
| 609 | Threonine--tRNA ligase, cytoplasmic OS=Homo sapiens GN=TARS PE=1 SV=3 | SYTC_HUMAN | 83 kDa |  | 18% | 13 | 15 | 16 |
| 610 | Tyrosine--tRNA ligase, cytoplasmic OS=Homo sapiens GN=YARS PE=1 SV=4 | SYYC_HUMAN | 59 kDa |  | 29% | 15 | 21 | 22 |
| 611 | Thioredoxin-like protein 1 OS=Homo sapiens GN=TXNL1 PE=1 SV=3 | TXNL1_HUMAN | 32 kDa |  | 50% | 10 | 13 | 17 |
| 612 | HLA class I histocompatibility antigen, A-24 alpha chain OS=Homo sapiens GN=HLA-A PE=1 SV=2 | 1A24_HUMAN | 41 kDa | TRUE | 27% | 8 | 14 | 17 |
| 613 | Cytosolic purine 5'-nucleotidase OS=Homo sapiens GN=NT5C2 PE=1 SV=1 | 5NTC_HUMAN | 65 kDa |  | 17% | 7 | 10 | 13 |
| 614 | Alpha-adducin OS=Homo sapiens GN=ADD1 PE=1 SV=2 | ADDA_HUMAN | 81 kDa | TRUE | 19% | 11 | 15 | 15 |
| 615 | Adiponectin OS=Homo sapiens GN=ADIPOQ PE=1 SV=1 | ADIPO_HUMAN | 26 kDa |  | 19% | 4 | 9 | 18 |
| 616 | Adipocyte enhancer-binding protein 1 OS=Homo sapiens GN=AEBP1 PE=1 SV=1 | AEBP1_HUMAN | 131 kDa | TRUE | 15% | 14 | 16 | 17 |
| 617 | Arf-GAP with Rho-GAP domain, ANK repeat and PH domain-containing protein 1 OS=Homo sapiens GN=ARAP1 PE=1 SV=3 | ARAP1_HUMAN | 162 kDa |  | 4.50% | 4 | 4 | 4 |
| 618 | 60 kDa heat shock protein, mitochondrial OS=Homo sapiens GN=HSPD1 PE=1 SV=2 | CH60_HUMAN | 61 kDa |  | 30% | 14 | 17 | 19 |
| 619 | Coronin-7 OS=Homo sapiens GN=CORO7 PE=1 SV=2 | CORO7_HUMAN | 101 kDa |  | 15% | 10 | 14 | 16 |
| 620 | Cullin-4A OS=Homo sapiens GN=CUL4A PE=1 SV=3 | CUL4A_HUMAN | 88 kDa | TRUE | 16% | 11 | 11 | 13 |
| 621 | Cullin-5 OS=Homo sapiens GN=CUL5 PE=1 SV=4 | CUL5_HUMAN | 91 kDa |  | 13% | 9 | 12 | 14 |
| 622 | Eukaryotic translation initiation factor 3 subunit J OS=Homo sapiens GN=EIF3J PE=1 SV=2 | EIF3J_HUMAN | 29 kDa |  | 28% | 9 | 13 | 16 |
| 623 | S-formylglutathione hydrolase OS=Homo sapiens GN=ESD PE=1 SV=2 | ESTD_HUMAN | 31 kDa |  | 47% | 8 | 11 | 15 |
| 624 | Rho GDP-dissociation inhibitor 2 OS=Homo sapiens GN=ARHGDIB PE=1 SV=3 | GDIR2_HUMAN | 23 kDa | TRUE | 47% | 7 | 12 | 15 |
| 625 | C-Jun-amino-terminal kinase-interacting protein 4 OS=Homo sapiens GN=SPAG9 PE=1 SV=4 | JIP4_HUMAN | 146 kDa | TRUE | 8.50% | 8 | 11 | 17 |
| 626 | Ketosamine-3-kinase OS=Homo sapiens GN=FN3KRP PE=1 SV=2 | KT3K_HUMAN | 34 kDa | TRUE | 26% | 7 | 12 | 18 |
| 627 | Dual specificity mitogen-activated protein kinase kinase 3 OS=Homo sapiens GN=MAP2K3 PE=1 SV=2 | MP2K3_HUMAN | 39 kDa |  | 31% | 9 | 16 | 18 |
| 628 | Ubiquitin thioesterase OTU1 OS=Homo sapiens GN=YOD1 PE=1 SV=1 | OTU1_HUMAN | 38 kDa |  | 35% | 9 | 12 | 16 |
| 629 | Vitamin K-dependent protein C OS=Homo sapiens GN=PROC PE=1 SV=1 | PROC_HUMAN | 52 kDa |  | 20% | 10 | 13 | 22 |
| 630 | Glutaminyl-peptide cyclotransferase OS=Homo sapiens GN=QPCT PE=1 SV=1 | QPCT_HUMAN | 41 kDa |  | 40% | 9 | 13 | 18 |
| 631 | 60S ribosomal protein L13a OS=Homo sapiens GN=RPL13A PE=1 SV=2 | RL13A_HUMAN | 24 kDa |  | 22% | 5 | 9 | 11 |
| 632 | 40S ribosomal protein SA OS=Homo sapiens GN=RPSA PE=1 SV=4 | RSSA_HUMAN | 33 kDa | TRUE | 36% | 7 | 12 | 14 |
| 633 | S-phase kinase-associated protein 1 OS=Homo sapiens GN=SKP1 PE=1 SV=2 | SKP1_HUMAN | 19 kDa |  | 59% | 8 | 12 | 17 |
| 634 | Valine--tRNA ligase OS=Homo sapiens GN=VARS PE=1 SV=4 | SYVC_HUMAN | 140 kDa |  | 10% | 10 | 14 | 14 |
| 635 | Tenascin OS=Homo sapiens GN=TNC PE=1 SV=3 | TENA_HUMAN | 241 kDa |  | 12% | 16 | 16 | 16 |
| 636 | Testin OS=Homo sapiens GN=TES PE=1 SV=1 | TES_HUMAN | 48 kDa |  | 19% | 8 | 11 | 15 |
| 637 | Xaa-Pro aminopeptidase 1 OS=Homo sapiens GN=XPNPEP1 PE=1 SV=3 | XPP1_HUMAN | 70 kDa |  | 23% | 12 | 16 | 18 |
| 638 | Polyadenylate-binding protein 2 OS=Homo sapiens GN=PABPN1 PE=1 SV=3 | PABP2_HUMAN | 33 kDa |  | 14% | 3 | 4 | 5 |
| 639 | Adenine phosphoribosyltransferase OS=Homo sapiens GN=APRT PE=1 SV=2 | APT_HUMAN | 20 kDa |  | 61% | 10 | 12 | 17 |
| 640 | Ubiquitin-like-conjugating enzyme ATG3 OS=Homo sapiens GN=ATG3 PE=1 SV=1 | ATG3_HUMAN | 36 kDa |  | 25% | 8 | 11 | 14 |
| 641 | Cytoplasmic FMR1-interacting protein 2 OS=Homo sapiens GN=CYFIP2 PE=1 SV=2 | CYFP2_HUMAN | 148 kDa | TRUE | 8.10% | 9 | 10 | 12 |
| 642 | Proteasome-associated protein ECM29 homolog OS=Homo sapiens GN=ECM29 PE=1 SV=2 | ECM29_HUMAN | 204 kDa | TRUE | 6.20% | 7 | 8 | 8 |
| 643 | Eukaryotic translation initiation factor 3 subunit E OS=Homo sapiens GN=EIF3E PE=1 SV=1 | EIF3E_HUMAN | 52 kDa |  | 35% | 13 | 14 | 16 |
| 644 | Glutaredoxin-3 OS=Homo sapiens GN=GLRX3 PE=1 SV=2 | GLRX3_HUMAN | 37 kDa |  | 37% | 11 | 15 | 18 |
| 645 | Hyaluronan-binding protein 2 OS=Homo sapiens GN=HABP2 PE=1 SV=1 | HABP2_HUMAN | 63 kDa |  | 7.50% | 5 | 7 | 8 |
| 646 | Heterogeneous nuclear ribonucleoprotein H OS=Homo sapiens GN=HNRNPH1 PE=1 SV=4 | HNRH1_HUMAN | 49 kDa | TRUE | 28% | 6 | 11 | 19 |
| 647 | Plasma protease C1 inhibitor OS=Homo sapiens GN=SERPING1 PE=1 SV=2 | IC1_HUMAN | 55 kDa |  | 20% | 9 | 14 | 16 |
| 648 | Involucrin OS=Homo sapiens GN=IVL PE=1 SV=2 | INVO_HUMAN | 68 kDa |  | 22% | 10 | 12 | 15 |
| 649 | Adenylate kinase isoenzyme 1 OS=Homo sapiens GN=AK1 PE=1 SV=3 | KAD1_HUMAN | 22 kDa | TRUE | 49% | 8 | 11 | 18 |
| 650 | cAMP-dependent protein kinase type II-alpha regulatory subunit OS=Homo sapiens GN=PRKAR2A PE=1 SV=2 | KAP2_HUMAN | 46 kDa |  | 42% | 13 | 19 | 19 |
| 651 | NAD kinase OS=Homo sapiens GN=NADK PE=1 SV=1 | NADK_HUMAN | 49 kDa |  | 26% | 10 | 14 | 19 |
| 652 | Protein disulfide-isomerase A3 OS=Homo sapiens GN=PDIA3 PE=1 SV=4 | PDIA3_HUMAN | 57 kDa | TRUE | 25% | 12 | 15 | 17 |
| 653 | Protein phosphatase 1F OS=Homo sapiens GN=PPM1F PE=1 SV=3 | PPM1F_HUMAN | 50 kDa |  | 37% | 11 | 13 | 15 |
| 654 | 26S proteasome non-ATPase regulatory subunit 7 OS=Homo sapiens GN=PSMD7 PE=1 SV=2 | PSMD7_HUMAN | 37 kDa |  | 35% | 8 | 13 | 14 |
| 655 | Histone-binding protein RBBP4 OS=Homo sapiens GN=RBBP4 PE=1 SV=3 | RBBP4_HUMAN | 48 kDa | TRUE | 32% | 10 | 15 | 16 |
| 656 | Switch-associated protein 70 OS=Homo sapiens GN=SWAP70 PE=1 SV=1 | SWP70_HUMAN | 69 kDa | TRUE | 15% | 7 | 8 | 8 |
| 657 | Serine--tRNA ligase, cytoplasmic OS=Homo sapiens GN=SARS PE=1 SV=3 | SYSC_HUMAN | 59 kDa |  | 35% | 14 | 17 | 19 |
| 658 | Transport and Golgi organization protein 2 homolog OS=Homo sapiens GN=TANGO2 PE=2 SV=1 | TNG2_HUMAN | 31 kDa |  | 63% | 11 | 14 | 16 |
| 659 | Probable ubiquitin carboxyl-terminal hydrolase FAF-X OS=Homo sapiens GN=USP9X PE=1 SV=3 | USP9X_HUMAN | 292 kDa |  | 4.50% | 9 | 11 | 11 |
| 660 | Vascular cell adhesion protein 1 OS=Homo sapiens GN=VCAM1 PE=1 SV=1 | VCAM1_HUMAN | 81 kDa | TRUE | 21% | 15 | 15 | 16 |
| 661 | Y-box-binding protein 3 OS=Homo sapiens GN=YBX3 PE=1 SV=4 | YBOX3_HUMAN | 40 kDa | TRUE | 26% | 6 | 9 | 13 |
| 662 | Ig kappa chain V-III region CLL OS=Homo sapiens PE=4 SV=2 | KV308_HUMAN | 14 kDa | TRUE | 25% | 3 | 4 | 8 |
| 663 | ATPase ASNA1 OS=Homo sapiens GN=ASNA1 PE=1 SV=2 | ASNA_HUMAN | 39 kDa |  | 43% | 11 | 14 | 18 |
| 664 | Ubiquitin-like modifier-activating enzyme ATG7 OS=Homo sapiens GN=ATG7 PE=1 SV=1 | ATG7_HUMAN | 78 kDa |  | 15% | 10 | 13 | 13 |
| 665 | Chromobox protein homolog 3 OS=Homo sapiens GN=CBX3 PE=1 SV=4 | CBX3_HUMAN | 21 kDa | TRUE | 44% | 6 | 10 | 13 |
| 666 | Coiled-coil domain-containing protein 88B OS=Homo sapiens GN=CCDC88B PE=1 SV=1 | CC88B_HUMAN | 165 kDa | TRUE | 7.00% | 9 | 10 | 11 |
| 667 | Cullin-2 OS=Homo sapiens GN=CUL2 PE=1 SV=2 | CUL2_HUMAN | 87 kDa |  | 24% | 15 | 15 | 15 |
| 668 | L-xylulose reductase OS=Homo sapiens GN=DCXR PE=1 SV=2 | DCXR_HUMAN | 26 kDa |  | 52% | 10 | 14 | 17 |
| 669 | Desmoplakin OS=Homo sapiens GN=DSP PE=1 SV=3 | DESP_HUMAN | 332 kDa | TRUE | 2.50% | 6 | 6 | 6 |
| 670 | Bifunctional ATP-dependent dihydroxyacetone kinase/FAD-AMP lyase (cyclizing) OS=Homo sapiens GN=DAK PE=1 SV=2 | DHAK_HUMAN | ? |  | 0.00% | 11 | 12 | 17 |
| 671 | Neutrophil elastase OS=Homo sapiens GN=ELANE PE=1 SV=1 | ELNE_HUMAN | 29 kDa |  | 42% | 7 | 11 | 17 |
| 672 | Guanine nucleotide-binding protein G(i) subunit alpha-2 OS=Homo sapiens GN=GNAI2 PE=1 SV=3 | GNAI2_HUMAN | 40 kDa | TRUE | 27% | 8 | 10 | 13 |
| 673 | Keratin, type I cytoskeletal 17 OS=Homo sapiens GN=KRT17 PE=1 SV=2 | K1C17_HUMAN | 48 kDa | TRUE | 49% | 10 | 13 | 34 |
| 674 | Phosphoribosyl pyrophosphate synthase-associated protein 2 OS=Homo sapiens GN=PRPSAP2 PE=1 SV=1 | KPRB_HUMAN | 41 kDa | TRUE | 28% | 7 | 12 | 14 |
| 675 | Kinectin OS=Homo sapiens GN=KTN1 PE=1 SV=1 | KTN1_HUMAN | 156 kDa | TRUE | 11% | 11 | 11 | 12 |
| 676 | Nucleoside diphosphate kinase A OS=Homo sapiens GN=NME1 PE=1 SV=1 | NDKA_HUMAN | 17 kDa | TRUE | 51% | 4 | 9 | 34 |
| 677 | Pyridoxal kinase OS=Homo sapiens GN=PDXK PE=1 SV=1 | PDXK_HUMAN | 35 kDa | TRUE | 36% | 9 | 14 | 19 |
| 678 | Platelet factor 4 OS=Homo sapiens GN=PF4 PE=1 SV=2 | PLF4_HUMAN | 11 kDa |  | 36% | 4 | 9 | 16 |
| 679 | 60S ribosomal protein L13 OS=Homo sapiens GN=RPL13 PE=1 SV=4 | RL13_HUMAN | 24 kDa |  | 33% | 7 | 9 | 11 |
| 680 | 60S ribosomal protein L15 OS=Homo sapiens GN=RPL15 PE=1 SV=2 | RL15_HUMAN | 24 kDa |  | 42% | 8 | 10 | 15 |
| 681 | 60S ribosomal protein L18 OS=Homo sapiens GN=RPL18 PE=1 SV=2 | RL18_HUMAN | 22 kDa |  | 33% | 5 | 8 | 11 |
| 682 | 60S ribosomal protein L7a OS=Homo sapiens GN=RPL7A PE=1 SV=2 | RL7A_HUMAN | 30 kDa |  | 32% | 7 | 10 | 10 |
| 683 | 40S ribosomal protein S4, X isoform OS=Homo sapiens GN=RPS4X PE=1 SV=2 | RS4X_HUMAN | 30 kDa |  | 29% | 7 | 10 | 11 |
| 684 | 40S ribosomal protein S5 OS=Homo sapiens GN=RPS5 PE=1 SV=4 | RS5_HUMAN | 23 kDa | TRUE | 59% | 9 | 12 | 16 |
| 685 | Endophilin-B2 OS=Homo sapiens GN=SH3GLB2 PE=1 SV=1 | SHLB2_HUMAN | 44 kDa | TRUE | 31% | 11 | 12 | 12 |
| 686 | Serpin B6 OS=Homo sapiens GN=SERPINB6 PE=1 SV=3 | SPB6_HUMAN | 43 kDa | TRUE | 37% | 10 | 14 | 15 |
| 687 | Sulfotransferase 1A1 OS=Homo sapiens GN=SULT1A1 PE=1 SV=3 | ST1A1_HUMAN | 34 kDa | TRUE | 32% | 9 | 12 | 16 |
| 688 | Serine-threonine kinase receptor-associated protein OS=Homo sapiens GN=STRAP PE=1 SV=1 | STRAP_HUMAN | 38 kDa |  | 32% | 9 | 14 | 15 |
| 689 | Triosephosphate isomerase OS=Homo sapiens GN=TPI1 PE=1 SV=3 | TPIS_HUMAN | 31 kDa |  | 40% | 10 | 12 | 16 |
| 690 | Vacuolar protein sorting-associated protein 26A OS=Homo sapiens GN=VPS26A PE=1 SV=2 | VP26A_HUMAN | 38 kDa | TRUE | 22% | 6 | 10 | 11 |
| 691 | WW domain-binding protein 2 OS=Homo sapiens GN=WBP2 PE=1 SV=1 | WBP2_HUMAN | 28 kDa |  | 16% | 5 | 8 | 11 |
| 692 | AP-2 complex subunit beta OS=Homo sapiens GN=AP2B1 PE=1 SV=1 | AP2B1_HUMAN | 105 kDa | TRUE | 26% | 10 | 14 | 28 |
| 693 | Copper chaperone for superoxide dismutase OS=Homo sapiens GN=CCS PE=1 SV=1 | CCS_HUMAN | 29 kDa |  | 47% | 9 | 13 | 16 |
| 694 | Chondroitin sulfate proteoglycan 4 OS=Homo sapiens GN=CSPG4 PE=1 SV=2 | CSPG4_HUMAN | 251 kDa |  | 7.50% | 11 | 11 | 12 |
| 695 | DnaJ homolog subfamily B member 1 OS=Homo sapiens GN=DNAJB1 PE=1 SV=4 | DNJB1_HUMAN | 38 kDa | TRUE | 36% | 12 | 13 | 13 |
| 696 | Elongation factor 1-beta OS=Homo sapiens GN=EEF1B2 PE=1 SV=3 | EF1B_HUMAN | 25 kDa | TRUE | 43% | 6 | 12 | 18 |
| 697 | Glycogen debranching enzyme OS=Homo sapiens GN=AGL PE=1 SV=3 | GDE_HUMAN | 175 kDa | TRUE | 6.50% | 5 | 6 | 6 |
| 698 | Eukaryotic translation initiation factor 4B OS=Homo sapiens GN=EIF4B PE=1 SV=2 | IF4B_HUMAN | 69 kDa | TRUE | 18% | 8 | 10 | 12 |
| 699 | Inorganic pyrophosphatase OS=Homo sapiens GN=PPA1 PE=1 SV=2 | IPYR_HUMAN | 33 kDa |  | 41% | 11 | 15 | 18 |
| 700 | Mitogen-activated protein kinase 14 OS=Homo sapiens GN=MAPK14 PE=1 SV=3 | MK14_HUMAN | 41 kDa |  | 21% | 7 | 12 | 14 |
| 701 | Dual specificity mitogen-activated protein kinase kinase 1 OS=Homo sapiens GN=MAP2K1 PE=1 SV=2 | MP2K1_HUMAN | 43 kDa | TRUE | 21% | 7 | 8 | 9 |
| 702 | Interferon-induced GTP-binding protein Mx1 OS=Homo sapiens GN=MX1 PE=1 SV=4 | MX1_HUMAN | 76 kDa | TRUE | 22% | 10 | 12 | 15 |
| 703 | ATP-dependent 6-phosphofructokinase, muscle type OS=Homo sapiens GN=PFKM PE=1 SV=2 | PFKAM_HUMAN | 85 kDa | TRUE | 17% | 8 | 12 | 17 |
| 704 | PITH domain-containing protein 1 OS=Homo sapiens GN=PITHD1 PE=1 SV=1 | PITH1_HUMAN | 24 kDa |  | 47% | 7 | 10 | 13 |
| 705 | Pre-mRNA-processing-splicing factor 8 OS=Homo sapiens GN=PRPF8 PE=1 SV=2 | PRP8_HUMAN | 274 kDa |  | 4.60% | 8 | 9 | 10 |
| 706 | Ras-related protein Rab-5C OS=Homo sapiens GN=RAB5C PE=1 SV=2 | RAB5C_HUMAN | 23 kDa | TRUE | 52% | 8 | 12 | 17 |
| 707 | Reticulocalbin-3 OS=Homo sapiens GN=RCN3 PE=1 SV=1 | RCN3_HUMAN | 37 kDa |  | 31% | 9 | 13 | 18 |
| 708 | Rho GTPase-activating protein 25 OS=Homo sapiens GN=ARHGAP25 PE=1 SV=2 | RHG25_HUMAN | 73 kDa |  | 16% | 9 | 12 | 13 |
| 709 | 60S ribosomal protein L12 OS=Homo sapiens GN=RPL12 PE=1 SV=1 | RL12_HUMAN | 18 kDa |  | 55% | 7 | 11 | 11 |
| 710 | 60S ribosomal protein L14 OS=Homo sapiens GN=RPL14 PE=1 SV=4 | RL14_HUMAN | 23 kDa | TRUE | 28% | 6 | 9 | 11 |
| 711 | 60S ribosomal protein L26 OS=Homo sapiens GN=RPL26 PE=1 SV=1 | RL26_HUMAN | 17 kDa |  | 25% | 6 | 10 | 13 |
| 712 | 60S ribosomal protein L8 OS=Homo sapiens GN=RPL8 PE=1 SV=2 | RL8_HUMAN | 28 kDa |  | 16% | 4 | 5 | 6 |
| 713 | Heterogeneous nuclear ribonucleoprotein A/B OS=Homo sapiens GN=HNRNPAB PE=1 SV=2 | ROAA_HUMAN | 36 kDa | TRUE | 21% | 5 | 9 | 14 |
| 714 | Septin-9 OS=Homo sapiens GN=SEPT9 PE=1 SV=2 | SEPT9_HUMAN | 65 kDa |  | 12% | 6 | 7 | 11 |
| 715 | Endophilin-A2 OS=Homo sapiens GN=SH3GL1 PE=1 SV=1 | SH3G1_HUMAN | 41 kDa |  | 28% | 7 | 10 | 13 |
| 716 | Acetyl-CoA acetyltransferase, cytosolic OS=Homo sapiens GN=ACAT2 PE=1 SV=2 | THIC_HUMAN | 41 kDa | TRUE | 17% | 6 | 8 | 10 |
| 717 | Transcription intermediary factor 1-beta OS=Homo sapiens GN=TRIM28 PE=1 SV=5 | TIF1B_HUMAN | 89 kDa |  | 17% | 12 | 15 | 16 |
| 718 | E3 ubiquitin-protein ligase UBR4 OS=Homo sapiens GN=UBR4 PE=1 SV=1 | UBR4_HUMAN | 574 kDa | TRUE | 1.20% | 5 | 5 | 5 |
| 719 | Ig kappa chain V-II region TEW OS=Homo sapiens PE=1 SV=1 | KV204_HUMAN | 12 kDa | TRUE | 39% | 3 | 6 | 18 |
| 720 | Calmodulin-like protein 3 OS=Homo sapiens GN=CALML3 PE=1 SV=2 | CALL3_HUMAN | 17 kDa | TRUE | 44% | 4 | 9 | 20 |
| 721 | Caspase-1 OS=Homo sapiens GN=CASP1 PE=1 SV=1 | CASP1_HUMAN | 45 kDa | TRUE | 28% | 9 | 11 | 12 |
| 722 | Coatomer subunit epsilon OS=Homo sapiens GN=COPE PE=1 SV=3 | COPE_HUMAN | 34 kDa | TRUE | 34% | 9 | 9 | 10 |
| 723 | Eosinophil cationic protein OS=Homo sapiens GN=RNASE3 PE=1 SV=2 | ECP_HUMAN | 18 kDa |  | 39% | 6 | 8 | 10 |
| 724 | Fatty acid-binding protein, epidermal OS=Homo sapiens GN=FABP5 PE=1 SV=3 | FABP5_HUMAN | 15 kDa |  | 43% | 6 | 9 | 11 |
| 725 | Fetuin-B OS=Homo sapiens GN=FETUB PE=1 SV=2 | FETUB_HUMAN | 42 kDa |  | 27% | 8 | 9 | 12 |
| 726 | GTPase IMAP family member 4 OS=Homo sapiens GN=GIMAP4 PE=1 SV=1 | GIMA4_HUMAN | 38 kDa |  | 26% | 9 | 13 | 13 |
| 727 | GMP reductase 1 OS=Homo sapiens GN=GMPR PE=1 SV=1 | GMPR1_HUMAN | 37 kDa |  | 18% | 6 | 8 | 12 |
| 728 | Glutamate--cysteine ligase catalytic subunit OS=Homo sapiens GN=GCLC PE=1 SV=2 | GSH1_HUMAN | 73 kDa | TRUE | 19% | 10 | 11 | 13 |
| 729 | Huntingtin-interacting protein 1 OS=Homo sapiens GN=HIP1 PE=1 SV=5 | HIP1_HUMAN | 116 kDa |  | 10% | 9 | 9 | 10 |
| 730 | Inosine-5'-monophosphate dehydrogenase 2 OS=Homo sapiens GN=IMPDH2 PE=1 SV=2 | IMDH2_HUMAN | 56 kDa | TRUE | 25% | 9 | 12 | 12 |
| 731 | MAP kinase-activated protein kinase 3 OS=Homo sapiens GN=MAPKAPK3 PE=1 SV=1 | MAPK3_HUMAN | 43 kDa | TRUE | 22% | 8 | 14 | 18 |
| 732 | Matrix metalloproteinase-9 OS=Homo sapiens GN=MMP9 PE=1 SV=3 | MMP9_HUMAN | 78 kDa |  | 20% | 11 | 16 | 16 |
| 733 | Metastasis-associated protein MTA2 OS=Homo sapiens GN=MTA2 PE=1 SV=1 | MTA2_HUMAN | 75 kDa |  | 9.90% | 5 | 5 | 5 |
| 734 | Nuclear transport factor 2 OS=Homo sapiens GN=NUTF2 PE=1 SV=1 | NTF2_HUMAN | 14 kDa |  | 75% | 6 | 10 | 16 |
| 735 | Platelet-activating factor acetylhydrolase IB subunit gamma OS=Homo sapiens GN=PAFAH1B3 PE=1 SV=1 | PA1B3_HUMAN | 26 kDa |  | 26% | 6 | 7 | 10 |
| 736 | Phosphoglucomutase-2 OS=Homo sapiens GN=PGM2 PE=1 SV=4 | PGM2_HUMAN | 68 kDa | TRUE | 14% | 8 | 10 | 11 |
| 737 | Proteasome activator complex subunit 3 OS=Homo sapiens GN=PSME3 PE=1 SV=1 | PSME3_HUMAN | 30 kDa | TRUE | 29% | 6 | 8 | 11 |
| 738 | Glycogen phosphorylase, brain form OS=Homo sapiens GN=PYGB PE=1 SV=5 | PYGB_HUMAN | 97 kDa | TRUE | 28% | 11 | 13 | 29 |
| 739 | Regulator of nonsense transcripts 1 OS=Homo sapiens GN=UPF1 PE=1 SV=2 | RENT1_HUMAN | 124 kDa |  | 5.50% | 6 | 7 | 7 |
| 740 | 40S ribosomal protein S11 OS=Homo sapiens GN=RPS11 PE=1 SV=3 | RS11_HUMAN | 18 kDa |  | 26% | 6 | 8 | 11 |
| 741 | Splicing factor 3A subunit 1 OS=Homo sapiens GN=SF3A1 PE=1 SV=1 | SF3A1_HUMAN | 89 kDa | TRUE | 15% | 10 | 11 | 13 |
| 742 | Glutamine--tRNA ligase OS=Homo sapiens GN=QARS PE=1 SV=1 | SYQ_HUMAN | 88 kDa |  | 9.70% | 6 | 6 | 6 |
| 743 | UDP-glucose:glycoprotein glucosyltransferase 1 OS=Homo sapiens GN=UGGT1 PE=1 SV=3 | UGGG1_HUMAN | 177 kDa | TRUE | 11% | 13 | 14 | 15 |
| 744 | Vacuolar protein sorting-associated protein 29 OS=Homo sapiens GN=VPS29 PE=1 SV=1 | VPS29_HUMAN | 21 kDa |  | 53% | 8 | 11 | 13 |
| 745 | Zinc-alpha-2-glycoprotein OS=Homo sapiens GN=AZGP1 PE=1 SV=2 | ZA2G_HUMAN | 34 kDa |  | 30% | 8 | 11 | 15 |
| 746 | Histidine--tRNA ligase, cytoplasmic OS=Homo sapiens GN=HARS PE=1 SV=2 | SYHC_HUMAN | 57 kDa |  | 3.50% | 2 | 2 | 2 |
| 747 | AP-1 complex subunit mu-1 OS=Homo sapiens GN=AP1M1 PE=1 SV=3 | AP1M1_HUMAN | 49 kDa |  | 24% | 8 | 9 | 9 |
| 748 | Apolipoprotein C-IV OS=Homo sapiens GN=APOC4 PE=1 SV=1 | APOC4_HUMAN | 15 kDa |  | 51% | 7 | 10 | 13 |
| 749 | Arf-GAP with SH3 domain, ANK repeat and PH domain-containing protein 1 OS=Homo sapiens GN=ASAP1 PE=1 SV=4 | ASAP1_HUMAN | 126 kDa | TRUE | 12% | 10 | 11 | 11 |
| 750 | Basic leucine zipper and W2 domain-containing protein 1 OS=Homo sapiens GN=BZW1 PE=1 SV=1 | BZW1_HUMAN | 48 kDa |  | 24% | 10 | 11 | 11 |
| 751 | Cullin-1 OS=Homo sapiens GN=CUL1 PE=1 SV=2 | CUL1_HUMAN | 90 kDa |  | 16% | 10 | 11 | 12 |
| 752 | ATP-dependent RNA helicase DDX1 OS=Homo sapiens GN=DDX1 PE=1 SV=2 | DDX1_HUMAN | 82 kDa | TRUE | 18% | 12 | 14 | 15 |
| 753 | DnaJ homolog subfamily C member 13 OS=Homo sapiens GN=DNAJC13 PE=1 SV=5 | DJC13_HUMAN | 254 kDa |  | 7.00% | 11 | 12 | 13 |
| 754 | Engulfment and cell motility protein 1 OS=Homo sapiens GN=ELMO1 PE=1 SV=2 | ELMO1_HUMAN | 84 kDa | TRUE | 17% | 11 | 13 | 14 |
| 755 | Eukaryotic peptide chain release factor subunit 1 OS=Homo sapiens GN=ETF1 PE=1 SV=3 | ERF1_HUMAN | 49 kDa |  | 14% | 6 | 8 | 9 |
| 756 | ERO1-like protein alpha OS=Homo sapiens GN=ERO1A PE=1 SV=2 | ERO1A_HUMAN | 54 kDa | TRUE | 18% | 8 | 10 | 11 |
| 757 | Heat shock protein 105 kDa OS=Homo sapiens GN=HSPH1 PE=1 SV=1 | HS105_HUMAN | 97 kDa | TRUE | 6.80% | 4 | 4 | 8 |
| 758 | Eukaryotic initiation factor 4A-III OS=Homo sapiens GN=EIF4A3 PE=1 SV=4 | IF4A3_HUMAN | 47 kDa | TRUE | 29% | 7 | 8 | 18 |
| 759 | Eukaryotic translation initiation factor 4 gamma 1 OS=Homo sapiens GN=EIF4G1 PE=1 SV=4 | IF4G1_HUMAN | 175 kDa | TRUE | 8.60% | 12 | 12 | 13 |
| 760 | Eukaryotic translation initiation factor 5 OS=Homo sapiens GN=EIF5 PE=1 SV=2 | IF5_HUMAN | 49 kDa |  | 16% | 7 | 9 | 11 |
| 761 | Platelet-activating factor acetylhydrolase IB subunit alpha OS=Homo sapiens GN=PAFAH1B1 PE=1 SV=2 | LIS1_HUMAN | 47 kDa |  | 21% | 9 | 11 | 12 |
| 762 | N-acetylmuramoyl-L-alanine amidase OS=Homo sapiens GN=PGLYRP2 PE=1 SV=1 | PGRP2_HUMAN | 62 kDa |  | 22% | 8 | 10 | 12 |
| 763 | Purine nucleoside phosphorylase OS=Homo sapiens GN=PNP PE=1 SV=2 | PNPH_HUMAN | 32 kDa |  | 35% | 6 | 8 | 8 |
| 764 | Proteasome subunit beta type-10 OS=Homo sapiens GN=PSMB10 PE=1 SV=1 | PSB10_HUMAN | 29 kDa |  | 21% | 6 | 8 | 11 |
| 765 | Tyrosine-protein phosphatase non-receptor type 23 OS=Homo sapiens GN=PTPN23 PE=1 SV=1 | PTN23_HUMAN | 179 kDa | TRUE | 5.90% | 8 | 8 | 8 |
| 766 | Ras-related protein Rap-1A OS=Homo sapiens GN=RAP1A PE=1 SV=1 | RAP1A_HUMAN | 21 kDa | TRUE | 30% | 6 | 8 | 13 |
| 767 | 60 kDa SS-A/Ro ribonucleoprotein OS=Homo sapiens GN=TROVE2 PE=1 SV=2 | RO60_HUMAN | 61 kDa |  | 20% | 10 | 14 | 14 |
| 768 | Serum amyloid A-2 protein OS=Homo sapiens GN=SAA2 PE=1 SV=1 | SAA2_HUMAN | 14 kDa | TRUE | 64% | 3 | 7 | 64 |
| 769 | Sorcin OS=Homo sapiens GN=SRI PE=1 SV=1 | SORCN_HUMAN | 22 kDa |  | 30% | 6 | 10 | 15 |
| 770 | Signal transducer and activator of transcription 3 OS=Homo sapiens GN=STAT3 PE=1 SV=2 | STAT3_HUMAN | 88 kDa |  | 17% | 10 | 12 | 12 |
| 771 | Phenylalanine--tRNA ligase beta subunit OS=Homo sapiens GN=FARSB PE=1 SV=3 | SYFB_HUMAN | 66 kDa |  | 15% | 8 | 9 | 9 |
| 772 | Alpha-synuclein OS=Homo sapiens GN=SNCA PE=1 SV=1 | SYUA_HUMAN | 14 kDa | TRUE | 54% | 6 | 11 | 13 |
| 773 | Tryptophan--tRNA ligase, cytoplasmic OS=Homo sapiens GN=WARS PE=1 SV=2 | SYWC_HUMAN | 53 kDa |  | 17% | 6 | 7 | 7 |
| 774 | Twinfilin-2 OS=Homo sapiens GN=TWF2 PE=1 SV=2 | TWF2_HUMAN | 40 kDa | TRUE | 23% | 6 | 11 | 14 |
| 775 | Lysosomal protective protein OS=Homo sapiens GN=CTSA PE=1 SV=2 | PPGB_HUMAN | 54 kDa |  | 4.40% | 2 | 3 | 3 |
| 776 | Leucine-rich alpha-2-glycoprotein OS=Homo sapiens GN=LRG1 PE=1 SV=2 | A2GL_HUMAN | 38 kDa |  | 28% | 7 | 11 | 13 |
| 777 | Calmodulin-like protein 5 OS=Homo sapiens GN=CALML5 PE=1 SV=2 | CALL5_HUMAN | 16 kDa |  | 60% | 7 | 10 | 13 |
| 778 | F-actin-capping protein subunit alpha-2 OS=Homo sapiens GN=CAPZA2 PE=1 SV=3 | CAZA2_HUMAN | 33 kDa | TRUE | 61% | 8 | 11 | 21 |
| 779 | Cytidine deaminase OS=Homo sapiens GN=CDA PE=1 SV=2 | CDD_HUMAN | 16 kDa |  | 75% | 6 | 10 | 14 |
| 780 | Bifunctional coenzyme A synthase OS=Homo sapiens GN=COASY PE=1 SV=4 | COASY_HUMAN | 62 kDa |  | 16% | 7 | 9 | 11 |
| 781 | Collagen alpha-1(XII) chain OS=Homo sapiens GN=COL12A1 PE=1 SV=2 | COCA1_HUMAN | 333 kDa | TRUE | 2.80% | 8 | 12 | 13 |
| 782 | COP9 signalosome complex subunit 2 OS=Homo sapiens GN=COPS2 PE=1 SV=1 | CSN2_HUMAN | 52 kDa |  | 24% | 8 | 9 | 9 |
| 783 | Cytoplasmic dynein 1 intermediate chain 2 OS=Homo sapiens GN=DYNC1I2 PE=1 SV=3 | DC1I2_HUMAN | 71 kDa | TRUE | 27% | 9 | 11 | 13 |
| 784 | DnaJ homolog subfamily C member 9 OS=Homo sapiens GN=DNAJC9 PE=1 SV=1 | DNJC9_HUMAN | 30 kDa |  | 34% | 7 | 9 | 11 |
| 785 | Dynamin-1-like protein OS=Homo sapiens GN=DNM1L PE=1 SV=2 | DNM1L_HUMAN | 82 kDa |  | 15% | 9 | 12 | 13 |
| 786 | Eukaryotic translation initiation factor 3 subunit C OS=Homo sapiens GN=EIF3C PE=1 SV=1 | EIF3C_HUMAN | 105 kDa |  | 8.90% | 7 | 10 | 12 |
| 787 | Eukaryotic translation initiation factor 3 subunit H OS=Homo sapiens GN=EIF3H PE=1 SV=1 | EIF3H_HUMAN | 40 kDa |  | 27% | 8 | 13 | 14 |
| 788 | Coagulation factor IX OS=Homo sapiens GN=F9 PE=1 SV=2 | FA9_HUMAN | 52 kDa | TRUE | 15% | 6 | 8 | 10 |
| 789 | Filaggrin OS=Homo sapiens GN=FLG PE=1 SV=3 | FILA_HUMAN | 435 kDa |  | 2.20% | 8 | 10 | 11 |
| 790 | Peptidyl-prolyl cis-trans isomerase FKBP5 OS=Homo sapiens GN=FKBP5 PE=1 SV=2 | FKBP5_HUMAN | 51 kDa | TRUE | 30% | 9 | 10 | 11 |
| 791 | Glyoxylate reductase/hydroxypyruvate reductase OS=Homo sapiens GN=GRHPR PE=1 SV=1 | GRHPR_HUMAN | 36 kDa |  | 34% | 8 | 12 | 13 |
| 792 | Microtubule-associated protein 4 OS=Homo sapiens GN=MAP4 PE=1 SV=3 | MAP4_HUMAN | 121 kDa |  | 12% | 10 | 11 | 11 |
| 793 | N-alpha-acetyltransferase 15, NatA auxiliary subunit OS=Homo sapiens GN=NAA15 PE=1 SV=1 | NAA15_HUMAN | 101 kDa |  | 7.40% | 6 | 7 | 7 |
| 794 | 26S proteasome non-ATPase regulatory subunit 4 OS=Homo sapiens GN=PSMD4 PE=1 SV=1 | PSMD4_HUMAN | 41 kDa |  | 23% | 7 | 12 | 17 |
| 795 | Ras-related protein Rab-14 OS=Homo sapiens GN=RAB14 PE=1 SV=4 | RAB14_HUMAN | 24 kDa | TRUE | 54% | 8 | 12 | 16 |
| 796 | 60S ribosomal protein L21 OS=Homo sapiens GN=RPL21 PE=1 SV=2 | RL21_HUMAN | 19 kDa |  | 38% | 6 | 7 | 8 |
| 797 | Protein S100-A2 OS=Homo sapiens GN=S100A2 PE=1 SV=3 | S10A2_HUMAN | 11 kDa |  | 17% | 4 | 7 | 11 |
| 798 | Protein S100-A11 OS=Homo sapiens GN=S100A11 PE=1 SV=2 | S10AB_HUMAN | 12 kDa |  | 39% | 4 | 7 | 11 |
| 799 | Splicing factor 3A subunit 3 OS=Homo sapiens GN=SF3A3 PE=1 SV=1 | SF3A3_HUMAN | 59 kDa |  | 24% | 10 | 10 | 12 |
| 800 | Phosphatidylinositol 3,4,5-trisphosphate 5-phosphatase 1 OS=Homo sapiens GN=INPP5D PE=1 SV=2 | SHIP1_HUMAN | 133 kDa |  | 12% | 9 | 10 | 12 |
| 801 | Tubulin beta-4B chain OS=Homo sapiens GN=TUBB4B PE=1 SV=1 | TBB4B_HUMAN | 50 kDa | TRUE | 65% | 5 | 11 | 108 |
| 802 | Activated RNA polymerase II transcriptional coactivator p15 OS=Homo sapiens GN=SUB1 PE=1 SV=3 | TCP4_HUMAN | 14 kDa |  | 37% | 4 | 8 | 10 |
| 803 | Translin-associated protein X OS=Homo sapiens GN=TSNAX PE=1 SV=1 | TSNAX_HUMAN | 33 kDa |  | 29% | 8 | 10 | 11 |
| 804 | Protein unc-13 homolog D OS=Homo sapiens GN=UNC13D PE=1 SV=1 | UN13D_HUMAN | 123 kDa | TRUE | 12% | 10 | 10 | 11 |
| 805 | Vacuolar protein sorting-associated protein 4B OS=Homo sapiens GN=VPS4B PE=1 SV=2 | VPS4B_HUMAN | 49 kDa | TRUE | 14% | 6 | 9 | 10 |
| 806 | Exportin-7 OS=Homo sapiens GN=XPO7 PE=1 SV=3 | XPO7_HUMAN | 124 kDa | TRUE | 8.60% | 9 | 10 | 13 |
| 807 | 40S ribosomal protein S23 OS=Homo sapiens GN=RPS23 PE=1 SV=3 | RS23_HUMAN | 16 kDa |  | 22% | 3 | 5 | 6 |
| 808 | Protein S100-A6 OS=Homo sapiens GN=S100A6 PE=1 SV=1 | S10A6_HUMAN | 10 kDa |  | 34% | 4 | 6 | 10 |
| 809 | Ig heavy chain V-III region TIL OS=Homo sapiens PE=1 SV=1 | HV304_HUMAN | 12 kDa | TRUE | 26% | 2 | 4 | 13 |
| 810 | Alcohol dehydrogenase class-3 OS=Homo sapiens GN=ADH5 PE=1 SV=4 | ADHX_HUMAN | 40 kDa |  | 19% | 6 | 8 | 9 |
| 811 | Angiotensinogen OS=Homo sapiens GN=AGT PE=1 SV=1 | ANGT_HUMAN | 53 kDa |  | 18% | 7 | 11 | 13 |
| 812 | Histone-arginine methyltransferase CARM1 OS=Homo sapiens GN=CARM1 PE=1 SV=3 | CARM1_HUMAN | 66 kDa |  | 11% | 7 | 8 | 9 |
| 813 | CD2-associated protein OS=Homo sapiens GN=CD2AP PE=1 SV=1 | CD2AP_HUMAN | 71 kDa |  | 6.40% | 3 | 4 | 4 |
| 814 | CAP-Gly domain-containing linker protein 1 OS=Homo sapiens GN=CLIP1 PE=1 SV=2 | CLIP1_HUMAN | 162 kDa | TRUE | 3.80% | 5 | 5 | 6 |
| 815 | Collagen alpha-2(I) chain OS=Homo sapiens GN=COL1A2 PE=1 SV=7 | CO1A2_HUMAN | 129 kDa |  | 3.00% | 4 | 5 | 6 |
| 816 | Collagen alpha-2(V) chain OS=Homo sapiens GN=COL5A2 PE=1 SV=3 | CO5A2_HUMAN | 145 kDa | TRUE | 2.70% | 3 | 3 | 3 |
| 817 | Cartilage acidic protein 1 OS=Homo sapiens GN=CRTAC1 PE=1 SV=2 | CRAC1_HUMAN | 71 kDa |  | 20% | 9 | 10 | 11 |
| 818 | COP9 signalosome complex subunit 6 OS=Homo sapiens GN=COPS6 PE=1 SV=1 | CSN6_HUMAN | 36 kDa |  | 32% | 8 | 11 | 12 |
| 819 | Differentially expressed in FDCP 6 homolog OS=Homo sapiens GN=DEF6 PE=1 SV=1 | DEFI6_HUMAN | 74 kDa |  | 11% | 6 | 7 | 8 |
| 820 | Pre-mRNA-splicing factor ATP-dependent RNA helicase DHX15 OS=Homo sapiens GN=DHX15 PE=1 SV=2 | DHX15_HUMAN | 91 kDa | TRUE | 10% | 7 | 7 | 7 |
| 821 | EH domain-containing protein 4 OS=Homo sapiens GN=EHD4 PE=1 SV=1 | EHD4_HUMAN | 61 kDa | TRUE | 21% | 8 | 10 | 13 |
| 822 | Fumarate hydratase, mitochondrial OS=Homo sapiens GN=FH PE=1 SV=3 | FUMH_HUMAN | 55 kDa |  | 14% | 6 | 7 | 9 |
| 823 | Hydroxyacylglutathione hydrolase, mitochondrial OS=Homo sapiens GN=HAGH PE=1 SV=2 | GLO2_HUMAN | 34 kDa |  | 19% | 4 | 6 | 7 |
| 824 | Mannose-1-phosphate guanyltransferase beta OS=Homo sapiens GN=GMPPB PE=1 SV=2 | GMPPB_HUMAN | 40 kDa |  | 20% | 6 | 7 | 10 |
| 825 | Glutamate--cysteine ligase regulatory subunit OS=Homo sapiens GN=GCLM PE=1 SV=1 | GSH0_HUMAN | 31 kDa | TRUE | 39% | 8 | 9 | 10 |
| 826 | Serine protease HTRA1 OS=Homo sapiens GN=HTRA1 PE=1 SV=1 | HTRA1_HUMAN | 51 kDa | TRUE | 27% | 11 | 13 | 13 |
| 827 | Pro-interleukin-16 OS=Homo sapiens GN=IL16 PE=1 SV=4 | IL16_HUMAN | 142 kDa |  | 4.60% | 5 | 6 | 8 |
| 828 | BTB/POZ domain-containing protein KCTD12 OS=Homo sapiens GN=KCTD12 PE=1 SV=1 | KCD12_HUMAN | 36 kDa |  | 21% | 5 | 9 | 10 |
| 829 | MOB kinase activator 1A OS=Homo sapiens GN=MOB1A PE=1 SV=4 | MOB1A_HUMAN | 25 kDa |  | 21% | 3 | 4 | 7 |
| 830 | Neutrophil gelatinase-associated lipocalin OS=Homo sapiens GN=LCN2 PE=1 SV=2 | NGAL_HUMAN | 23 kDa |  | 40% | 6 | 10 | 11 |
| 831 | Osteoclast-stimulating factor 1 OS=Homo sapiens GN=OSTF1 PE=1 SV=2 | OSTF1_HUMAN | 24 kDa |  | 48% | 8 | 12 | 15 |
| 832 | Pleckstrin homology domain-containing family O member 2 OS=Homo sapiens GN=PLEKHO2 PE=1 SV=1 | PKHO2_HUMAN | 53 kDa |  | 13% | 5 | 5 | 6 |
| 833 | 60S acidic ribosomal protein P2 OS=Homo sapiens GN=RPLP2 PE=1 SV=1 | RLA2_HUMAN | 12 kDa |  | 70% | 5 | 9 | 15 |
| 834 | Heterogeneous nuclear ribonucleoprotein A3 OS=Homo sapiens GN=HNRNPA3 PE=1 SV=2 | ROA3_HUMAN | 40 kDa | TRUE | 14% | 5 | 8 | 17 |
| 835 | 40S ribosomal protein S6 OS=Homo sapiens GN=RPS6 PE=1 SV=1 | RS6_HUMAN | 29 kDa | TRUE | 21% | 5 | 8 | 8 |
| 836 | 40S ribosomal protein S7 OS=Homo sapiens GN=RPS7 PE=1 SV=1 | RS7_HUMAN | 22 kDa |  | 45% | 8 | 10 | 12 |
| 837 | Sec1 family domain-containing protein 1 OS=Homo sapiens GN=SCFD1 PE=1 SV=4 | SCFD1_HUMAN | 72 kDa | TRUE | 17% | 7 | 9 | 10 |
| 838 | Septin-2 OS=Homo sapiens GN=SEPT2 PE=1 SV=1 | SEPT2_HUMAN | 41 kDa |  | 30% | 7 | 9 | 9 |
| 839 | Alpha-soluble NSF attachment protein OS=Homo sapiens GN=NAPA PE=1 SV=3 | SNAA_HUMAN | 33 kDa |  | 46% | 12 | 12 | 13 |
| 840 | Sorting nexin-2 OS=Homo sapiens GN=SNX2 PE=1 SV=2 | SNX2_HUMAN | 58 kDa | TRUE | 20% | 8 | 10 | 15 |
| 841 | Extracellular superoxide dismutase [Cu-Zn] OS=Homo sapiens GN=SOD3 PE=1 SV=2 | SODE_HUMAN | 26 kDa |  | 30% | 5 | 7 | 14 |
| 842 | Signal transducer and activator of transcription 5B OS=Homo sapiens GN=STAT5B PE=1 SV=2 | STA5B_HUMAN | 90 kDa | TRUE | 16% | 10 | 12 | 13 |
| 843 | Target of Myb protein 1 OS=Homo sapiens GN=TOM1 PE=1 SV=2 | TOM1_HUMAN | 54 kDa |  | 26% | 9 | 12 | 14 |
| 844 | UBX domain-containing protein 1 OS=Homo sapiens GN=UBXN1 PE=1 SV=2 | UBXN1_HUMAN | 33 kDa | TRUE | 47% | 7 | 10 | 10 |
| 845 | Neutrophil defensin 1 OS=Homo sapiens GN=DEFA1 PE=1 SV=1 | DEF1_HUMAN | 10 kDa |  | 52% | 3 | 7 | 13 |
| 846 | Translationally-controlled tumor protein OS=Homo sapiens GN=TPT1 PE=1 SV=1 | TCTP_HUMAN | 20 kDa |  | 24% | 4 | 7 | 8 |
| 847 | Ig kappa chain V-III region B6 OS=Homo sapiens PE=1 SV=1 | KV301_HUMAN | 12 kDa | TRUE | 38% | 2 | 4 | 13 |
| 848 | Protein mago nashi homolog 2 OS=Homo sapiens GN=MAGOHB PE=1 SV=1 | MGN2_HUMAN | 17 kDa |  | 17% | 2 | 3 | 3 |
| 849 | Arf-GAP with coiled-coil, ANK repeat and PH domain-containing protein 1 OS=Homo sapiens GN=ACAP1 PE=1 SV=1 | ACAP1_HUMAN | 82 kDa | TRUE | 14% | 8 | 9 | 9 |
| 850 | Aminopeptidase B OS=Homo sapiens GN=RNPEP PE=1 SV=2 | AMPB_HUMAN | 73 kDa |  | 24% | 13 | 13 | 14 |
| 851 | AMP deaminase 2 OS=Homo sapiens GN=AMPD2 PE=1 SV=2 | AMPD2_HUMAN | 101 kDa | TRUE | 7.50% | 7 | 7 | 7 |
| 852 | Beta-2-glycoprotein 1 OS=Homo sapiens GN=APOH PE=1 SV=3 | APOH_HUMAN | 38 kDa |  | 25% | 6 | 7 | 8 |
| 853 | Aflatoxin B1 aldehyde reductase member 2 OS=Homo sapiens GN=AKR7A2 PE=1 SV=3 | ARK72_HUMAN | 40 kDa | TRUE | 18% | 6 | 7 | 13 |
| 854 | A disintegrin and metalloproteinase with thrombospondin motifs 13 OS=Homo sapiens GN=ADAMTS13 PE=1 SV=1 | ATS13_HUMAN | 154 kDa | TRUE | 4.00% | 5 | 6 | 7 |
| 855 | Calumenin OS=Homo sapiens GN=CALU PE=1 SV=2 | CALU_HUMAN | 37 kDa |  | 31% | 6 | 8 | 10 |
| 856 | Cathepsin B OS=Homo sapiens GN=CTSB PE=1 SV=3 | CATB_HUMAN | 38 kDa |  | 27% | 7 | 12 | 12 |
| 857 | 2',3'-cyclic-nucleotide 3'-phosphodiesterase OS=Homo sapiens GN=CNP PE=1 SV=2 | CN37_HUMAN | 48 kDa |  | 28% | 11 | 11 | 12 |
| 858 | DCC-interacting protein 13-alpha OS=Homo sapiens GN=APPL1 PE=1 SV=1 | DP13A_HUMAN | 80 kDa |  | 21% | 9 | 11 | 11 |
| 859 | Eukaryotic translation initiation factor 3 subunit I OS=Homo sapiens GN=EIF3I PE=1 SV=1 | EIF3I_HUMAN | 37 kDa |  | 26% | 8 | 12 | 13 |
| 860 | Coagulation factor XII OS=Homo sapiens GN=F12 PE=1 SV=3 | FA12_HUMAN | 68 kDa | TRUE | 16% | 8 | 10 | 11 |
| 861 | FAS-associated factor 1 OS=Homo sapiens GN=FAF1 PE=1 SV=2 | FAF1_HUMAN | 74 kDa |  | 8.30% | 5 | 6 | 6 |
| 862 | Ficolin-1 OS=Homo sapiens GN=FCN1 PE=1 SV=2 | FCN1_HUMAN | 35 kDa | TRUE | 15% | 4 | 5 | 8 |
| 863 | Ras GTPase-activating protein-binding protein 1 OS=Homo sapiens GN=G3BP1 PE=1 SV=1 | G3BP1_HUMAN | 52 kDa | TRUE | 11% | 4 | 5 | 6 |
| 864 | Guanylate-binding protein 2 OS=Homo sapiens GN=GBP2 PE=1 SV=3 | GBP2_HUMAN | 67 kDa | TRUE | 7.30% | 5 | 8 | 8 |
| 865 | Insulin-like growth factor-binding protein 3 OS=Homo sapiens GN=IGFBP3 PE=1 SV=2 | IBP3_HUMAN | 32 kDa |  | 16% | 4 | 6 | 8 |
| 866 | Keratin, type II cytoskeletal 2 epidermal OS=Homo sapiens GN=KRT2 PE=1 SV=2 | K22E_HUMAN | 65 kDa | TRUE | 26% | 8 | 13 | 28 |
| 867 | UMP-CMP kinase OS=Homo sapiens GN=CMPK1 PE=1 SV=3 | KCY_HUMAN | 22 kDa |  | 37% | 8 | 11 | 13 |
| 868 | Ig lambda chain V-III region SH OS=Homo sapiens PE=1 SV=1 | LV301_HUMAN | 11 kDa |  | 34% | 3 | 6 | 13 |
| 869 | Nucleosome assembly protein 1-like 4 OS=Homo sapiens GN=NAP1L4 PE=1 SV=1 | NP1L4_HUMAN | 43 kDa | TRUE | 18% | 5 | 10 | 12 |
| 870 | Olfactomedin-like protein 2B OS=Homo sapiens GN=OLFML2B PE=2 SV=2 | OLM2B_HUMAN | 84 kDa |  | 8.10% | 5 | 6 | 8 |
| 871 | Oxysterol-binding protein 1 OS=Homo sapiens GN=OSBP PE=1 SV=1 | OSBP1_HUMAN | 89 kDa | TRUE | 7.70% | 6 | 7 | 8 |
| 872 | ATP-dependent 6-phosphofructokinase, platelet type OS=Homo sapiens GN=PFKP PE=1 SV=2 | PFKAP_HUMAN | 86 kDa | TRUE | 19% | 8 | 9 | 18 |
| 873 | Glucose 1,6-bisphosphate synthase OS=Homo sapiens GN=PGM2L1 PE=1 SV=3 | PGM2L_HUMAN | 70 kDa | TRUE | 16% | 8 | 8 | 10 |
| 874 | Procollagen-lysine,2-oxoglutarate 5-dioxygenase 1 OS=Homo sapiens GN=PLOD1 PE=1 SV=2 | PLOD1_HUMAN | 84 kDa |  | 8.30% | 4 | 4 | 5 |
| 875 | Proline-serine-threonine phosphatase-interacting protein 1 OS=Homo sapiens GN=PSTPIP1 PE=1 SV=1 | PPIP1_HUMAN | 48 kDa |  | 20% | 6 | 6 | 7 |
| 876 | Ras-related protein Rab-8A OS=Homo sapiens GN=RAB8A PE=1 SV=1 | RAB8A_HUMAN | 24 kDa | TRUE | 37% | 5 | 7 | 12 |
| 877 | E3 ubiquitin-protein ligase RNF213 OS=Homo sapiens GN=RNF213 PE=1 SV=3 | RN213_HUMAN | 591 kDa | TRUE | 0.94% | 3 | 3 | 3 |
| 878 | 40S ribosomal protein S9 OS=Homo sapiens GN=RPS9 PE=1 SV=3 | RS9_HUMAN | 23 kDa |  | 22% | 4 | 4 | 5 |
| 879 | Small glutamine-rich tetratricopeptide repeat-containing protein alpha OS=Homo sapiens GN=SGTA PE=1 SV=1 | SGTA_HUMAN | 34 kDa | TRUE | 25% | 7 | 9 | 9 |
| 880 | SH3 domain-containing kinase-binding protein 1 OS=Homo sapiens GN=SH3KBP1 PE=1 SV=2 | SH3K1_HUMAN | 73 kDa |  | 18% | 8 | 9 | 10 |
| 881 | Sorting nexin-5 OS=Homo sapiens GN=SNX5 PE=1 SV=1 | SNX5_HUMAN | 47 kDa | TRUE | 26% | 9 | 12 | 16 |
| 882 | SPARC-like protein 1 OS=Homo sapiens GN=SPARCL1 PE=1 SV=2 | SPRL1_HUMAN | 75 kDa | TRUE | 15% | 8 | 10 | 12 |
| 883 | 116 kDa U5 small nuclear ribonucleoprotein component OS=Homo sapiens GN=EFTUD2 PE=1 SV=1 | U5S1_HUMAN | 109 kDa | TRUE | 14% | 9 | 10 | 13 |
| 884 | Ubiquitin carboxyl-terminal hydrolase 7 OS=Homo sapiens GN=USP7 PE=1 SV=2 | UBP7_HUMAN | 128 kDa |  | 13% | 10 | 11 | 11 |
| 885 | Ubiquitin carboxyl-terminal hydrolase isozyme L3 OS=Homo sapiens GN=UCHL3 PE=1 SV=1 | UCHL3_HUMAN | 26 kDa |  | 40% | 8 | 13 | 15 |
| 886 | UDP-glucose 6-dehydrogenase OS=Homo sapiens GN=UGDH PE=1 SV=1 | UGDH_HUMAN | 55 kDa |  | 21% | 8 | 9 | 10 |
| 887 | Ig heavy chain V-III region CAM OS=Homo sapiens PE=1 SV=1 | HV307_HUMAN | 14 kDa | TRUE | 30% | 4 | 5 | 9 |
| 888 | Ig alpha-2 chain C region OS=Homo sapiens GN=IGHA2 PE=1 SV=3 | IGHA2_HUMAN | 37 kDa | TRUE | 46% | 2 | 5 | 123 |
| 889 | Versican core protein OS=Homo sapiens GN=VCAN PE=1 SV=3 | CSPG2_HUMAN | 373 kDa |  | 0.50% | 2 | 2 | 2 |
| 890 | Endoplasmic reticulum resident protein 29 OS=Homo sapiens GN=ERP29 PE=1 SV=4 | ERP29_HUMAN | 29 kDa |  | 15% | 2 | 2 | 2 |
| 891 | Angiopoietin-related protein 3 OS=Homo sapiens GN=ANGPTL3 PE=1 SV=1 | ANGL3_HUMAN | 54 kDa | TRUE | 22% | 8 | 8 | 8 |
| 892 | Annexin A1 OS=Homo sapiens GN=ANXA1 PE=1 SV=2 | ANXA1_HUMAN | 39 kDa | TRUE | 30% | 7 | 10 | 11 |
| 893 | Annexin A3 OS=Homo sapiens GN=ANXA3 PE=1 SV=3 | ANXA3_HUMAN | 36 kDa | TRUE | 29% | 8 | 8 | 8 |
| 894 | Calpain-2 catalytic subunit OS=Homo sapiens GN=CAPN2 PE=1 SV=6 | CAN2_HUMAN | 80 kDa | TRUE | 18% | 9 | 10 | 11 |
| 895 | Chloride intracellular channel protein 4 OS=Homo sapiens GN=CLIC4 PE=1 SV=4 | CLIC4_HUMAN | 29 kDa | TRUE | 21% | 4 | 6 | 6 |
| 896 | Collagen alpha-1(III) chain OS=Homo sapiens GN=COL3A1 PE=1 SV=4 | CO3A1_HUMAN | 139 kDa |  | 4.40% | 6 | 8 | 9 |
| 897 | COP9 signalosome complex subunit 8 OS=Homo sapiens GN=COPS8 PE=1 SV=1 | CSN8_HUMAN | 23 kDa |  | 37% | 5 | 7 | 9 |
| 898 | Probable ATP-dependent RNA helicase DDX46 OS=Homo sapiens GN=DDX46 PE=1 SV=2 | DDX46_HUMAN | 117 kDa | TRUE | 3.70% | 4 | 4 | 4 |
| 899 | DnaJ homolog subfamily A member 2 OS=Homo sapiens GN=DNAJA2 PE=1 SV=1 | DNJA2_HUMAN | 46 kDa |  | 15% | 4 | 5 | 6 |
| 900 | Dihydropyrimidinase-related protein 3 OS=Homo sapiens GN=DPYSL3 PE=1 SV=1 | DPYL3_HUMAN | 62 kDa | TRUE | 27% | 8 | 10 | 23 |
| 901 | F-box only protein 7 OS=Homo sapiens GN=FBXO7 PE=1 SV=1 | FBX7_HUMAN | 59 kDa |  | 11% | 6 | 9 | 10 |
| 902 | GDP-L-fucose synthase OS=Homo sapiens GN=TSTA3 PE=1 SV=1 | FCL_HUMAN | 36 kDa | TRUE | 25% | 6 | 8 | 10 |
| 903 | Fibrinogen-like protein 1 OS=Homo sapiens GN=FGL1 PE=1 SV=3 | FGL1_HUMAN | 36 kDa |  | 18% | 5 | 7 | 7 |
| 904 | Fructosamine-3-kinase OS=Homo sapiens GN=FN3K PE=1 SV=1 | FN3K_HUMAN | 35 kDa |  | 27% | 7 | 9 | 10 |
| 905 | Histidine triad nucleotide-binding protein 1 OS=Homo sapiens GN=HINT1 PE=1 SV=2 | HINT1_HUMAN | 14 kDa |  | 53% | 5 | 8 | 10 |
| 906 | Insulin-degrading enzyme OS=Homo sapiens GN=IDE PE=1 SV=4 | IDE_HUMAN | 118 kDa |  | 4.50% | 4 | 4 | 5 |
| 907 | Gamma-interferon-inducible protein 16 OS=Homo sapiens GN=IFI16 PE=1 SV=3 | IF16_HUMAN | 88 kDa |  | 13% | 8 | 8 | 9 |
| 908 | IST1 homolog OS=Homo sapiens GN=IST1 PE=1 SV=1 | IST1_HUMAN | 40 kDa |  | 20% | 6 | 6 | 7 |
| 909 | Lupus La protein OS=Homo sapiens GN=SSB PE=1 SV=2 | LA_HUMAN | 47 kDa | TRUE | 25% | 8 | 8 | 9 |
| 910 | Latent-transforming growth factor beta-binding protein 1 OS=Homo sapiens GN=LTBP1 PE=1 SV=4 | LTBP1_HUMAN | 187 kDa |  | 3.00% | 5 | 6 | 6 |
| 911 | Myristoylated alanine-rich C-kinase substrate OS=Homo sapiens GN=MARCKS PE=1 SV=4 | MARCS_HUMAN | 32 kDa |  | 31% | 6 | 9 | 12 |
| 912 | Protein-methionine sulfoxide oxidase MICAL1 OS=Homo sapiens GN=MICAL1 PE=1 SV=2 | MICA1_HUMAN | 118 kDa |  | 5.60% | 6 | 7 | 8 |
| 913 | Neutrophil collagenase OS=Homo sapiens GN=MMP8 PE=1 SV=1 | MMP8_HUMAN | 53 kDa |  | 19% | 7 | 8 | 9 |
| 914 | S-methyl-5'-thioadenosine phosphorylase OS=Homo sapiens GN=MTAP PE=1 SV=2 | MTAP_HUMAN | 31 kDa |  | 28% | 5 | 8 | 11 |
| 915 | Nuclear autoantigenic sperm protein OS=Homo sapiens GN=NASP PE=1 SV=2 | NASP_HUMAN | 85 kDa | TRUE | 11% | 7 | 8 | 9 |
| 916 | N-myc-interactor OS=Homo sapiens GN=NMI PE=1 SV=2 | NMI_HUMAN | 35 kDa |  | 26% | 7 | 7 | 7 |
| 917 | Nuclear protein localization protein 4 homolog OS=Homo sapiens GN=NPLOC4 PE=1 SV=3 | NPL4_HUMAN | 68 kDa |  | 13% | 4 | 5 | 5 |
| 918 | Phosphatidylinositol 3-kinase regulatory subunit alpha OS=Homo sapiens GN=PIK3R1 PE=1 SV=2 | P85A_HUMAN | 84 kDa | TRUE | 14% | 7 | 8 | 8 |
| 919 | Polyadenylate-binding protein 4 OS=Homo sapiens GN=PABPC4 PE=1 SV=1 | PABP4_HUMAN | 71 kDa | TRUE | 15% | 5 | 7 | 13 |
| 920 | 26S proteasome non-ATPase regulatory subunit 10 OS=Homo sapiens GN=PSMD10 PE=1 SV=1 | PSD10_HUMAN | 24 kDa | TRUE | 26% | 5 | 7 | 10 |
| 921 | Tyrosine-protein phosphatase non-receptor type 1 OS=Homo sapiens GN=PTPN1 PE=1 SV=1 | PTN1_HUMAN | 50 kDa |  | 17% | 7 | 8 | 11 |
| 922 | 60S ribosomal protein L17 OS=Homo sapiens GN=RPL17 PE=1 SV=3 | RL17_HUMAN | 21 kDa |  | 27% | 4 | 6 | 8 |
| 923 | 60S ribosomal protein L30 OS=Homo sapiens GN=RPL30 PE=1 SV=2 | RL30_HUMAN | 13 kDa |  | 32% | 3 | 4 | 9 |
| 924 | 60S ribosomal protein L35a OS=Homo sapiens GN=RPL35A PE=1 SV=2 | RL35A_HUMAN | 13 kDa |  | 42% | 6 | 7 | 8 |
| 925 | 60S ribosomal protein L7 OS=Homo sapiens GN=RPL7 PE=1 SV=1 | RL7_HUMAN | 29 kDa |  | 28% | 6 | 6 | 8 |
| 926 | 40S ribosomal protein S13 OS=Homo sapiens GN=RPS13 PE=1 SV=2 | RS13_HUMAN | 17 kDa |  | 29% | 4 | 5 | 5 |
| 927 | 40S ribosomal protein S18 OS=Homo sapiens GN=RPS18 PE=1 SV=3 | RS18_HUMAN | 18 kDa |  | 20% | 4 | 6 | 7 |
| 928 | 40S ribosomal protein S21 OS=Homo sapiens GN=RPS21 PE=1 SV=1 | RS21_HUMAN | 9 kDa |  | 39% | 4 | 5 | 6 |
| 929 | SUMO-activating enzyme subunit 2 OS=Homo sapiens GN=UBA2 PE=1 SV=2 | SAE2_HUMAN | 71 kDa |  | 11% | 6 | 7 | 7 |
| 930 | Protein SET OS=Homo sapiens GN=SET PE=1 SV=3 | SET_HUMAN | 33 kDa |  | 23% | 5 | 9 | 11 |
| 931 | Tumor necrosis factor alpha-induced protein 8 OS=Homo sapiens GN=TNFAIP8 PE=1 SV=1 | TFIP8_HUMAN | 23 kDa | TRUE | 29% | 5 | 7 | 9 |
| 932 | Ig lambda chain V-I region NEWM OS=Homo sapiens PE=1 SV=1 | LV105_HUMAN | 11 kDa |  | 17% | 2 | 4 | 12 |
| 933 | Abl interactor 1 OS=Homo sapiens GN=ABI1 PE=1 SV=4 | ABI1_HUMAN | 55 kDa |  | 21% | 6 | 7 | 7 |
| 934 | Band 3 anion transport protein OS=Homo sapiens GN=SLC4A1 PE=1 SV=3 | B3AT_HUMAN | 102 kDa | TRUE | 9.40% | 6 | 9 | 9 |
| 935 | Dipeptidyl peptidase 1 OS=Homo sapiens GN=CTSC PE=1 SV=2 | CATC_HUMAN | 52 kDa |  | 18% | 6 | 9 | 10 |
| 936 | Coiled-coil domain-containing protein 22 OS=Homo sapiens GN=CCDC22 PE=1 SV=1 | CCD22_HUMAN | 71 kDa |  | 21% | 11 | 11 | 13 |
| 937 | Coiled-coil domain-containing protein 93 OS=Homo sapiens GN=CCDC93 PE=1 SV=2 | CCD93_HUMAN | 73 kDa |  | 8.20% | 5 | 5 | 5 |
| 938 | Uroporphyrinogen decarboxylase OS=Homo sapiens GN=UROD PE=1 SV=2 | DCUP_HUMAN | 41 kDa |  | 34% | 6 | 8 | 8 |
| 939 | ATP-dependent RNA helicase DDX3X OS=Homo sapiens GN=DDX3X PE=1 SV=3 | DDX3X_HUMAN | 73 kDa | TRUE | 12% | 5 | 5 | 9 |
| 940 | Probable ATP-dependent RNA helicase DDX5 OS=Homo sapiens GN=DDX5 PE=1 SV=1 | DDX5_HUMAN | 69 kDa | TRUE | 19% | 8 | 8 | 20 |
| 941 | EH domain-containing protein 2 OS=Homo sapiens GN=EHD2 PE=1 SV=2 | EHD2_HUMAN | 61 kDa | TRUE | 15% | 5 | 5 | 8 |
| 942 | Eukaryotic translation initiation factor 3 subunit M OS=Homo sapiens GN=EIF3M PE=1 SV=1 | EIF3M_HUMAN | 43 kDa |  | 18% | 5 | 7 | 9 |
| 943 | Endoplasmic reticulum resident protein 44 OS=Homo sapiens GN=ERP44 PE=1 SV=1 | ERP44_HUMAN | 47 kDa |  | 12% | 5 | 7 | 7 |
| 944 | Coagulation factor VII OS=Homo sapiens GN=F7 PE=1 SV=1 | FA7_HUMAN | 52 kDa | TRUE | 16% | 5 | 6 | 6 |
| 945 | RNA-binding protein FUS OS=Homo sapiens GN=FUS PE=1 SV=1 | FUS_HUMAN | 53 kDa | TRUE | 8.60% | 5 | 8 | 8 |
| 946 | Glutaredoxin-1 OS=Homo sapiens GN=GLRX PE=1 SV=2 | GLRX1_HUMAN | 12 kDa |  | 58% | 3 | 5 | 6 |
| 947 | GMP synthase [glutamine-hydrolyzing] OS=Homo sapiens GN=GMPS PE=1 SV=1 | GUAA_HUMAN | 77 kDa |  | 11% | 6 | 6 | 6 |
| 948 | Core histone macro-H2A.1 OS=Homo sapiens GN=H2AFY PE=1 SV=4 | H2AY_HUMAN | 40 kDa |  | 25% | 6 | 9 | 10 |
| 949 | Hepatocyte growth factor-like protein OS=Homo sapiens GN=MST1 PE=1 SV=2 | HGFL_HUMAN | 80 kDa |  | 7.60% | 5 | 5 | 5 |
| 950 | Laminin subunit alpha-2 OS=Homo sapiens GN=LAMA2 PE=1 SV=4 | LAMA2_HUMAN | 344 kDa |  | 1.60% | 4 | 4 | 4 |
| 951 | LIM and senescent cell antigen-like-containing domain protein 1 OS=Homo sapiens GN=LIMS1 PE=1 SV=4 | LIMS1_HUMAN | 37 kDa |  | 16% | 5 | 5 | 10 |
| 952 | Nck-associated protein 1-like OS=Homo sapiens GN=NCKAP1L PE=1 SV=3 | NCKPL_HUMAN | 128 kDa |  | 4.50% | 4 | 4 | 4 |
| 953 | Nidogen-2 OS=Homo sapiens GN=NID2 PE=1 SV=3 | NID2_HUMAN | 151 kDa | TRUE | 8.60% | 8 | 10 | 13 |
| 954 | Olfactomedin-like protein 3 OS=Homo sapiens GN=OLFML3 PE=2 SV=1 | OLFL3_HUMAN | 46 kDa |  | 17% | 7 | 7 | 7 |
| 955 | Platelet-activating factor acetylhydrolase IB subunit beta OS=Homo sapiens GN=PAFAH1B2 PE=1 SV=1 | PA1B2_HUMAN | 26 kDa |  | 44% | 6 | 6 | 7 |
| 956 | Protein phosphatase 1A OS=Homo sapiens GN=PPM1A PE=1 SV=1 | PPM1A_HUMAN | 42 kDa | TRUE | 24% | 6 | 8 | 15 |
| 957 | Pre-mRNA-processing factor 19 OS=Homo sapiens GN=PRPF19 PE=1 SV=1 | PRP19_HUMAN | 55 kDa |  | 7.70% | 4 | 6 | 6 |
| 958 | Proteasome subunit beta type-9 OS=Homo sapiens GN=PSMB9 PE=1 SV=2 | PSB9_HUMAN | 23 kDa |  | 32% | 7 | 10 | 11 |
| 959 | Paraspeckle component 1 OS=Homo sapiens GN=PSPC1 PE=1 SV=1 | PSPC1_HUMAN | 59 kDa | TRUE | 13% | 5 | 6 | 7 |
| 960 | CAD protein OS=Homo sapiens GN=CAD PE=1 SV=3 | PYR1_HUMAN | 243 kDa | TRUE | 1.90% | 4 | 5 | 6 |
| 961 | DNA repair protein RAD50 OS=Homo sapiens GN=RAD50 PE=1 SV=1 | RAD50_HUMAN | 154 kDa | TRUE | 2.70% | 3 | 4 | 4 |
| 962 | Ran-specific GTPase-activating protein OS=Homo sapiens GN=RANBP1 PE=1 SV=1 | RANG_HUMAN | 23 kDa |  | 30% | 5 | 9 | 10 |
| 963 | Reticulocalbin-1 OS=Homo sapiens GN=RCN1 PE=1 SV=1 | RCN1_HUMAN | 39 kDa |  | 24% | 7 | 9 | 11 |
| 964 | 60S ribosomal protein L23a OS=Homo sapiens GN=RPL23A PE=1 SV=1 | RL23A_HUMAN | 18 kDa |  | 21% | 4 | 9 | 9 |
| 965 | 60S ribosomal protein L28 OS=Homo sapiens GN=RPL28 PE=1 SV=3 | RL28_HUMAN | 16 kDa | TRUE | 19% | 3 | 5 | 6 |
| 966 | 40S ribosomal protein S12 OS=Homo sapiens GN=RPS12 PE=1 SV=3 | RS12_HUMAN | 15 kDa |  | 32% | 4 | 7 | 12 |
| 967 | 40S ribosomal protein S14 OS=Homo sapiens GN=RPS14 PE=1 SV=3 | RS14_HUMAN | 16 kDa |  | 36% | 5 | 9 | 11 |
| 968 | 40S ribosomal protein S15 OS=Homo sapiens GN=RPS15 PE=1 SV=2 | RS15_HUMAN | 17 kDa |  | 42% | 4 | 6 | 10 |
| 969 | 40S ribosomal protein S16 OS=Homo sapiens GN=RPS16 PE=1 SV=2 | RS16_HUMAN | 16 kDa |  | 33% | 5 | 5 | 6 |
| 970 | 40S ribosomal protein S25 OS=Homo sapiens GN=RPS25 PE=1 SV=1 | RS25_HUMAN | 14 kDa |  | 28% | 4 | 7 | 8 |
| 971 | U2 small nuclear ribonucleoprotein B'' OS=Homo sapiens GN=SNRPB2 PE=1 SV=1 | RU2B_HUMAN | 25 kDa | TRUE | 23% | 5 | 7 | 8 |
| 972 | Splicing factor 3B subunit 1 OS=Homo sapiens GN=SF3B1 PE=1 SV=3 | SF3B1_HUMAN | 146 kDa |  | 3.10% | 4 | 5 | 5 |
| 973 | Src kinase-associated phosphoprotein 2 OS=Homo sapiens GN=SKAP2 PE=1 SV=1 | SKAP2_HUMAN | 41 kDa |  | 28% | 8 | 10 | 13 |
| 974 | Serpin B8 OS=Homo sapiens GN=SERPINB8 PE=1 SV=2 | SPB8_HUMAN | 43 kDa | TRUE | 22% | 6 | 8 | 14 |
| 975 | SPARC OS=Homo sapiens GN=SPARC PE=1 SV=1 | SPRC_HUMAN | 35 kDa |  | 20% | 7 | 11 | 12 |
| 976 | Spermine synthase OS=Homo sapiens GN=SMS PE=1 SV=2 | SPSY_HUMAN | 41 kDa |  | 32% | 9 | 9 | 10 |
| 977 | Leucine--tRNA ligase, cytoplasmic OS=Homo sapiens GN=LARS PE=1 SV=2 | SYLC_HUMAN | 134 kDa | TRUE | 8.60% | 7 | 8 | 8 |
| 978 | Arginine--tRNA ligase, cytoplasmic OS=Homo sapiens GN=RARS PE=1 SV=2 | SYRC_HUMAN | 75 kDa |  | 11% | 6 | 6 | 6 |
| 979 | TSC22 domain family protein 4 OS=Homo sapiens GN=TSC22D4 PE=1 SV=2 | T22D4_HUMAN | 41 kDa |  | 34% | 10 | 11 | 11 |
| 980 | Transgelin-2 OS=Homo sapiens GN=TAGLN2 PE=1 SV=3 | TAGL2_HUMAN | 22 kDa |  | 36% | 6 | 9 | 12 |
| 981 | Thioredoxin OS=Homo sapiens GN=TXN PE=1 SV=3 | THIO_HUMAN | 12 kDa |  | 31% | 4 | 7 | 12 |
| 982 | Toll-interacting protein OS=Homo sapiens GN=TOLLIP PE=1 SV=1 | TOLIP_HUMAN | 30 kDa |  | 26% | 7 | 9 | 10 |
| 983 | Tripeptidyl-peptidase 1 OS=Homo sapiens GN=TPP1 PE=1 SV=2 | TPP1_HUMAN | 61 kDa |  | 18% | 7 | 8 | 8 |
| 984 | V-type proton ATPase subunit C 1 OS=Homo sapiens GN=ATP6V1C1 PE=1 SV=4 | VATC1_HUMAN | 44 kDa |  | 18% | 6 | 6 | 6 |
| 985 | V-type proton ATPase subunit H OS=Homo sapiens GN=ATP6V1H PE=1 SV=1 | VATH_HUMAN | 56 kDa | TRUE | 24% | 8 | 9 | 10 |
| 986 | Talin-2 OS=Homo sapiens GN=TLN2 PE=1 SV=4 | TLN2_HUMAN | 272 kDa | TRUE | 7.70% | 4 | 4 | 38 |
| 987 | Serine/threonine-protein phosphatase 6 catalytic subunit OS=Homo sapiens GN=PPP6C PE=1 SV=1 | PPP6_HUMAN | 35 kDa | TRUE | 15% | 4 | 4 | 4 |
| 988 | Ig heavy chain V-II region ARH-77 OS=Homo sapiens PE=4 SV=1 | HV209_HUMAN | 16 kDa |  | 17% | 2 | 4 | 11 |
| 989 | Ig kappa chain V-I region Ni OS=Homo sapiens PE=1 SV=1 | KV121_HUMAN | 12 kDa |  | 30% | 2 | 5 | 12 |
| 990 | AH receptor-interacting protein OS=Homo sapiens GN=AIP PE=1 SV=2 | AIP_HUMAN | 38 kDa |  | 22% | 5 | 6 | 7 |
| 991 | Ankyrin repeat domain-containing protein 13A OS=Homo sapiens GN=ANKRD13A PE=1 SV=3 | AN13A_HUMAN | 68 kDa |  | 22% | 10 | 10 | 10 |
| 992 | AP-1 complex subunit gamma-1 OS=Homo sapiens GN=AP1G1 PE=1 SV=5 | AP1G1_HUMAN | 91 kDa | TRUE | 3.20% | 3 | 3 | 3 |
| 993 | Transcription factor BTF3 OS=Homo sapiens GN=BTF3 PE=1 SV=1 | BTF3_HUMAN | 22 kDa | TRUE | 35% | 4 | 5 | 5 |
| 994 | Complement factor D OS=Homo sapiens GN=CFD PE=1 SV=5 | CFAD_HUMAN | 27 kDa |  | 21% | 3 | 5 | 6 |
| 995 | Collagen alpha-1(XVIII) chain OS=Homo sapiens GN=COL18A1 PE=1 SV=5 | COIA1_HUMAN | 178 kDa |  | 3.30% | 4 | 5 | 7 |
| 996 | Tyrosine-protein kinase CSK OS=Homo sapiens GN=CSK PE=1 SV=1 | CSK_HUMAN | 51 kDa |  | 15% | 6 | 7 | 7 |
| 997 | COP9 signalosome complex subunit 7b OS=Homo sapiens GN=COPS7B PE=1 SV=1 | CSN7B_HUMAN | 30 kDa |  | 30% | 6 | 8 | 9 |
| 998 | EF-hand domain-containing protein D2 OS=Homo sapiens GN=EFHD2 PE=1 SV=1 | EFHD2_HUMAN | 27 kDa |  | 28% | 6 | 8 | 8 |
| 999 | Eukaryotic translation initiation factor 3 subunit G OS=Homo sapiens GN=EIF3G PE=1 SV=2 | EIF3G_HUMAN | 36 kDa |  | 15% | 5 | 6 | 9 |
| 1000 | Epidermal growth factor receptor substrate 15-like 1 OS=Homo sapiens GN=EPS15L1 PE=1 SV=1 | EP15R_HUMAN | 94 kDa |  | 13% | 8 | 8 | 9 |
| 1001 | Hemoglobin subunit theta-1 OS=Homo sapiens GN=HBQ1 PE=1 SV=2 | HBAT_HUMAN | 16 kDa |  | 58% | 7 | 10 | 12 |
| 1002 | Heme-binding protein 1 OS=Homo sapiens GN=HEBP1 PE=1 SV=1 | HEBP1_HUMAN | 21 kDa |  | 37% | 6 | 7 | 9 |
| 1003 | Porphobilinogen deaminase OS=Homo sapiens GN=HMBS PE=1 SV=2 | HEM3_HUMAN | 39 kDa |  | 26% | 9 | 10 | 10 |
| 1004 | High mobility group protein B1 OS=Homo sapiens GN=HMGB1 PE=1 SV=3 | HMGB1_HUMAN | 25 kDa | TRUE | 27% | 5 | 8 | 15 |
| 1005 | Heterogeneous nuclear ribonucleoprotein U-like protein 1 OS=Homo sapiens GN=HNRNPUL1 PE=1 SV=2 | HNRL1_HUMAN | 96 kDa |  | 7.10% | 4 | 4 | 5 |
| 1006 | Heterogeneous nuclear ribonucleoprotein R OS=Homo sapiens GN=HNRNPR PE=1 SV=1 | HNRPR_HUMAN | 71 kDa | TRUE | 25% | 7 | 7 | 20 |
| 1007 | Ig heavy chain V-I region HG3 OS=Homo sapiens PE=3 SV=1 | HV102_HUMAN | 13 kDa | TRUE | 32% | 4 | 6 | 7 |
| 1008 | Eukaryotic translation initiation factor 2 subunit 2 OS=Homo sapiens GN=EIF2S2 PE=1 SV=2 | IF2B_HUMAN | 38 kDa |  | 20% | 5 | 7 | 7 |
| 1009 | Eukaryotic translation initiation factor 4E OS=Homo sapiens GN=EIF4E PE=1 SV=2 | IF4E_HUMAN | 25 kDa |  | 24% | 4 | 5 | 5 |
| 1010 | Interferon-induced protein with tetratricopeptide repeats 3 OS=Homo sapiens GN=IFIT3 PE=1 SV=1 | IFIT3_HUMAN | 56 kDa |  | 18% | 7 | 9 | 10 |
| 1011 | Immunoglobulin-binding protein 1 OS=Homo sapiens GN=IGBP1 PE=1 SV=1 | IGBP1_HUMAN | 39 kDa |  | 17% | 5 | 7 | 7 |
| 1012 | Importin subunit alpha-3 OS=Homo sapiens GN=KPNA4 PE=1 SV=1 | IMA3_HUMAN | 58 kDa |  | 11% | 4 | 4 | 4 |
| 1013 | Kinesin light chain 1 OS=Homo sapiens GN=KLC1 PE=1 SV=2 | KLC1_HUMAN | 65 kDa | TRUE | 12% | 7 | 7 | 7 |
| 1014 | Malate dehydrogenase, cytoplasmic OS=Homo sapiens GN=MDH1 PE=1 SV=4 | MDHC_HUMAN | 36 kDa |  | 24% | 6 | 8 | 9 |
| 1015 | Multiple inositol polyphosphate phosphatase 1 OS=Homo sapiens GN=MINPP1 PE=1 SV=1 | MINP1_HUMAN | 55 kDa |  | 17% | 6 | 7 | 8 |
| 1016 | Myosin-10 OS=Homo sapiens GN=MYH10 PE=1 SV=3 | MYH10_HUMAN | 229 kDa | TRUE | 11% | 4 | 5 | 43 |
| 1017 | Bifunctional 3'-phosphoadenosine 5'-phosphosulfate synthase 1 OS=Homo sapiens GN=PAPSS1 PE=1 SV=2 | PAPS1_HUMAN | 71 kDa | TRUE | 7.10% | 4 | 4 | 4 |
| 1018 | Choline-phosphate cytidylyltransferase A OS=Homo sapiens GN=PCYT1A PE=1 SV=2 | PCY1A_HUMAN | 42 kDa |  | 23% | 7 | 7 | 7 |
| 1019 | Prefoldin subunit 3 OS=Homo sapiens GN=VBP1 PE=1 SV=3 | PFD3_HUMAN | 23 kDa |  | 37% | 6 | 8 | 9 |
| 1020 | Phosphoglycerate mutase 1 OS=Homo sapiens GN=PGAM1 PE=1 SV=2 | PGAM1_HUMAN | 29 kDa |  | 43% | 7 | 8 | 8 |
| 1021 | Phosphatidylinositol-binding clathrin assembly protein OS=Homo sapiens GN=PICALM PE=1 SV=2 | PICAL_HUMAN | 71 kDa |  | 11% | 5 | 6 | 7 |
| 1022 | Phospholipase A-2-activating protein OS=Homo sapiens GN=PLAA PE=1 SV=2 | PLAP_HUMAN | 87 kDa |  | 12% | 7 | 7 | 8 |
| 1023 | Bisphosphoglycerate mutase OS=Homo sapiens GN=BPGM PE=1 SV=2 | PMGE_HUMAN | 30 kDa |  | 41% | 6 | 8 | 9 |
| 1024 | Serum paraoxonase/lactonase 3 OS=Homo sapiens GN=PON3 PE=1 SV=3 | PON3_HUMAN | 40 kDa |  | 23% | 6 | 8 | 18 |
| 1025 | Peptidyl-prolyl cis-trans isomerase D OS=Homo sapiens GN=PPID PE=1 SV=3 | PPID_HUMAN | 41 kDa | TRUE | 18% | 7 | 7 | 7 |
| 1026 | Proteasome subunit beta type-2 OS=Homo sapiens GN=PSMB2 PE=1 SV=1 | PSB2_HUMAN | 23 kDa |  | 20% | 3 | 7 | 8 |
| 1027 | 26S proteasome non-ATPase regulatory subunit 8 OS=Homo sapiens GN=PSMD8 PE=1 SV=2 | PSMD8_HUMAN | 40 kDa |  | 17% | 5 | 6 | 6 |
| 1028 | 26S proteasome non-ATPase regulatory subunit 9 OS=Homo sapiens GN=PSMD9 PE=1 SV=3 | PSMD9_HUMAN | 25 kDa |  | 43% | 9 | 11 | 11 |
| 1029 | Transcriptional activator protein Pur-alpha OS=Homo sapiens GN=PURA PE=1 SV=2 | PURA_HUMAN | 35 kDa | TRUE | 27% | 6 | 7 | 7 |
| 1030 | CTP synthase 1 OS=Homo sapiens GN=CTPS1 PE=1 SV=2 | PYRG1_HUMAN | 67 kDa |  | 7.60% | 5 | 5 | 6 |
| 1031 | Ras-related protein Rab-10 OS=Homo sapiens GN=RAB10 PE=1 SV=1 | RAB10_HUMAN | 23 kDa | TRUE | 29% | 4 | 6 | 12 |
| 1032 | Ras association domain-containing protein 2 OS=Homo sapiens GN=RASSF2 PE=1 SV=1 | RASF2_HUMAN | 38 kDa |  | 35% | 8 | 9 | 9 |
| 1033 | 60S ribosomal protein L9 OS=Homo sapiens GN=RPL9 PE=1 SV=1 | RL9_HUMAN | 22 kDa |  | 45% | 7 | 9 | 10 |
| 1034 | Rho-associated protein kinase 2 OS=Homo sapiens GN=ROCK2 PE=1 SV=4 | ROCK2_HUMAN | 161 kDa | TRUE | 5.00% | 3 | 3 | 7 |
| 1035 | Small nuclear ribonucleoprotein Sm D2 OS=Homo sapiens GN=SNRPD2 PE=1 SV=1 | SMD2_HUMAN | 14 kDa |  | 40% | 5 | 9 | 10 |
| 1036 | Serpin B5 OS=Homo sapiens GN=SERPINB5 PE=1 SV=2 | SPB5_HUMAN | 42 kDa |  | 21% | 7 | 7 | 8 |
| 1037 | Sulfotransferase family cytosolic 1B member 1 OS=Homo sapiens GN=SULT1B1 PE=1 SV=2 | ST1B1_HUMAN | 35 kDa |  | 33% | 7 | 8 | 8 |
| 1038 | Tubulin alpha-4A chain OS=Homo sapiens GN=TUBA4A PE=1 SV=1 | TBA4A_HUMAN | 50 kDa | TRUE | 61% | 4 | 7 | 95 |
| 1039 | Tetranectin OS=Homo sapiens GN=CLEC3B PE=1 SV=3 | TETN_HUMAN | 23 kDa | TRUE | 39% | 5 | 6 | 6 |
| 1040 | Transportin-3 OS=Homo sapiens GN=TNPO3 PE=1 SV=3 | TNPO3_HUMAN | 104 kDa |  | 13% | 8 | 8 | 9 |
| 1041 | V-type proton ATPase subunit D OS=Homo sapiens GN=ATP6V1D PE=1 SV=1 | VATD_HUMAN | 28 kDa |  | 35% | 8 | 8 | 9 |
| 1042 | 60S ribosomal protein L10 OS=Homo sapiens GN=RPL10 PE=1 SV=4 | RL10_HUMAN | 25 kDa |  | 15% | 3 | 4 | 6 |
| 1043 | 60S ribosomal protein L27 OS=Homo sapiens GN=RPL27 PE=1 SV=2 | RL27_HUMAN | 16 kDa | TRUE | 30% | 3 | 5 | 8 |
| 1044 | Ras-related protein Rab-1A OS=Homo sapiens GN=RAB1A PE=1 SV=3 | RAB1A_HUMAN | 23 kDa | TRUE | 53% | 2 | 3 | 19 |
| 1045 | Clathrin light chain A OS=Homo sapiens GN=CLTA PE=1 SV=1 | CLCA_HUMAN | 27 kDa |  | 12% | 4 | 5 | 6 |
| 1046 | Ig lambda chain V-I region NEW OS=Homo sapiens PE=1 SV=1 | LV103_HUMAN | 11 kDa | TRUE | 26% | 3 | 5 | 11 |
| 1047 | Ig lambda chain V region 4A OS=Homo sapiens PE=4 SV=1 | LV001_HUMAN | 12 kDa |  | 21% | 3 | 5 | 8 |
| 1048 | Histone H2B type 2-E OS=Homo sapiens GN=HIST2H2BE PE=1 SV=3 | H2B2E_HUMAN | 14 kDa | TRUE | 63% | 2 | 5 | 74 |
| 1049 | Gamma-adducin OS=Homo sapiens GN=ADD3 PE=1 SV=1 | ADDG_HUMAN | 79 kDa | TRUE | 16% | 8 | 9 | 9 |
| 1050 | Apoptosis inhibitor 5 OS=Homo sapiens GN=API5 PE=1 SV=3 | API5_HUMAN | 59 kDa |  | 14% | 6 | 9 | 9 |
| 1051 | Rho guanine nucleotide exchange factor 2 OS=Homo sapiens GN=ARHGEF2 PE=1 SV=4 | ARHG2_HUMAN | 112 kDa | TRUE | 4.40% | 4 | 4 | 4 |
| 1052 | Biliverdin reductase A OS=Homo sapiens GN=BLVRA PE=1 SV=2 | BIEA_HUMAN | 33 kDa |  | 26% | 5 | 5 | 5 |
| 1053 | 45 kDa calcium-binding protein OS=Homo sapiens GN=SDF4 PE=1 SV=1 | CAB45_HUMAN | 42 kDa |  | 12% | 4 | 5 | 5 |
| 1054 | Calcineurin subunit B type 1 OS=Homo sapiens GN=PPP3R1 PE=1 SV=2 | CANB1_HUMAN | 19 kDa |  | 27% | 4 | 6 | 7 |
| 1055 | E3 ubiquitin-protein ligase CBL OS=Homo sapiens GN=CBL PE=1 SV=2 | CBL_HUMAN | 100 kDa |  | 8.90% | 6 | 6 | 7 |
| 1056 | Cell cycle and apoptosis regulator protein 2 OS=Homo sapiens GN=CCAR2 PE=1 SV=2 | CCAR2_HUMAN | 103 kDa |  | 11% | 7 | 7 | 7 |
| 1057 | Calcium-regulated heat stable protein 1 OS=Homo sapiens GN=CARHSP1 PE=1 SV=2 | CHSP1_HUMAN | 16 kDa |  | 31% | 4 | 6 | 8 |
| 1058 | C-type lectin domain family 11 member A OS=Homo sapiens GN=CLEC11A PE=1 SV=1 | CLC11_HUMAN | 36 kDa |  | 20% | 6 | 6 | 6 |
| 1059 | Casein kinase II subunit alpha OS=Homo sapiens GN=CSNK2A1 PE=1 SV=1 | CSK21_HUMAN | 45 kDa | TRUE | 17% | 7 | 9 | 9 |
| 1060 | Dematin OS=Homo sapiens GN=DMTN PE=1 SV=3 | DEMA_HUMAN | 46 kDa |  | 9.60% | 4 | 5 | 7 |
| 1061 | DnaJ homolog subfamily B member 11 OS=Homo sapiens GN=DNAJB11 PE=1 SV=1 | DJB11_HUMAN | 41 kDa |  | 12% | 4 | 5 | 6 |
| 1062 | DnaJ homolog subfamily A member 4 OS=Homo sapiens GN=DNAJA4 PE=1 SV=1 | DNJA4_HUMAN | 45 kDa | TRUE | 17% | 5 | 7 | 7 |
| 1063 | Dedicator of cytokinesis protein 11 OS=Homo sapiens GN=DOCK11 PE=1 SV=2 | DOC11_HUMAN | 238 kDa | TRUE | 3.70% | 6 | 6 | 6 |
| 1064 | EH domain-binding protein 1-like protein 1 OS=Homo sapiens GN=EHBP1L1 PE=1 SV=2 | EH1L1_HUMAN | 162 kDa | TRUE | 4.50% | 5 | 5 | 5 |
| 1065 | Eukaryotic translation initiation factor 3 subunit D OS=Homo sapiens GN=EIF3D PE=1 SV=1 | EIF3D_HUMAN | 64 kDa |  | 11% | 5 | 6 | 8 |
| 1066 | Eukaryotic translation initiation factor 3 subunit L OS=Homo sapiens GN=EIF3L PE=1 SV=1 | EIF3L_HUMAN | 67 kDa |  | 13% | 6 | 6 | 6 |
| 1067 | ELAV-like protein 1 OS=Homo sapiens GN=ELAVL1 PE=1 SV=2 | ELAV1_HUMAN | 36 kDa |  | 16% | 5 | 7 | 10 |
| 1068 | Galactokinase OS=Homo sapiens GN=GALK1 PE=1 SV=1 | GALK1_HUMAN | 42 kDa | TRUE | 20% | 6 | 7 | 7 |
| 1069 | Glutamine--fructose-6-phosphate aminotransferase [isomerizing] 1 OS=Homo sapiens GN=GFPT1 PE=1 SV=3 | GFPT1_HUMAN | 79 kDa | TRUE | 11% | 6 | 6 | 6 |
| 1070 | Tyrosine-protein kinase HCK OS=Homo sapiens GN=HCK PE=1 SV=5 | HCK_HUMAN | 60 kDa | TRUE | 15% | 7 | 7 | 7 |
| 1071 | Heterogeneous nuclear ribonucleoprotein D-like OS=Homo sapiens GN=HNRNPDL PE=1 SV=3 | HNRDL_HUMAN | 46 kDa | TRUE | 16% | 4 | 7 | 12 |
| 1072 | Eukaryotic translation initiation factor 6 OS=Homo sapiens GN=EIF6 PE=1 SV=1 | IF6_HUMAN | 27 kDa |  | 30% | 5 | 6 | 8 |
| 1073 | Importin-9 OS=Homo sapiens GN=IPO9 PE=1 SV=3 | IPO9_HUMAN | 116 kDa |  | 6.70% | 5 | 7 | 7 |
| 1074 | Immunoglobulin superfamily containing leucine-rich repeat protein OS=Homo sapiens GN=ISLR PE=2 SV=1 | ISLR_HUMAN | 46 kDa |  | 11% | 4 | 7 | 8 |
| 1075 | Integrin alpha-M OS=Homo sapiens GN=ITGAM PE=1 SV=2 | ITAM_HUMAN | 127 kDa | TRUE | 7.20% | 6 | 6 | 6 |
| 1076 | Phosphoribosyl pyrophosphate synthase-associated protein 1 OS=Homo sapiens GN=PRPSAP1 PE=1 SV=2 | KPRA_HUMAN | 39 kDa | TRUE | 18% | 3 | 4 | 10 |
| 1077 | U6 snRNA-associated Sm-like protein LSm4 OS=Homo sapiens GN=LSM4 PE=1 SV=1 | LSM4_HUMAN | 15 kDa |  | 20% | 3 | 8 | 9 |
| 1078 | Acyl-protein thioesterase 1 OS=Homo sapiens GN=LYPLA1 PE=1 SV=1 | LYPA1_HUMAN | 25 kDa |  | 22% | 3 | 5 | 7 |
| 1079 | Microtubule-associated protein 1S OS=Homo sapiens GN=MAP1S PE=1 SV=2 | MAP1S_HUMAN | 112 kDa |  | 10% | 8 | 9 | 9 |
| 1080 | S-adenosylmethionine synthase isoform type-2 OS=Homo sapiens GN=MAT2A PE=1 SV=1 | METK2_HUMAN | 44 kDa | TRUE | 12% | 4 | 6 | 6 |
| 1081 | Glutamine-dependent NAD(+) synthetase OS=Homo sapiens GN=NADSYN1 PE=1 SV=3 | NADE_HUMAN | 79 kDa |  | 8.10% | 5 | 5 | 6 |
| 1082 | Nicotinamide N-methyltransferase OS=Homo sapiens GN=NNMT PE=1 SV=1 | NNMT_HUMAN | 30 kDa |  | 26% | 5 | 7 | 8 |
| 1083 | Nuclear mitotic apparatus protein 1 OS=Homo sapiens GN=NUMA1 PE=1 SV=2 | NUMA1_HUMAN | 238 kDa | TRUE | 3.70% | 6 | 6 | 6 |
| 1084 | Olfactomedin-4 OS=Homo sapiens GN=OLFM4 PE=1 SV=1 | OLFM4_HUMAN | 57 kDa |  | 10% | 4 | 4 | 5 |
| 1085 | Prolyl 4-hydroxylase subunit alpha-1 OS=Homo sapiens GN=P4HA1 PE=1 SV=2 | P4HA1_HUMAN | 61 kDa |  | 17% | 8 | 8 | 8 |
| 1086 | Platelet-activating factor acetylhydrolase OS=Homo sapiens GN=PLA2G7 PE=1 SV=1 | PAFA_HUMAN | 50 kDa | TRUE | 13% | 5 | 6 | 6 |
| 1087 | Serine/threonine-protein phosphatase PP1-alpha catalytic subunit OS=Homo sapiens GN=PPP1CA PE=1 SV=1 | PP1A_HUMAN | 38 kDa | TRUE | 23% | 7 | 8 | 8 |
| 1088 | Serine/threonine-protein phosphatase 2B catalytic subunit alpha isoform OS=Homo sapiens GN=PPP3CA PE=1 SV=1 | PP2BA_HUMAN | 59 kDa | TRUE | 15% | 7 | 8 | 8 |
| 1089 | Prolyl endopeptidase OS=Homo sapiens GN=PREP PE=1 SV=2 | PPCE_HUMAN | 81 kDa |  | 12% | 7 | 7 | 7 |
| 1090 | Ras-related protein Ral-A OS=Homo sapiens GN=RALA PE=1 SV=1 | RALA_HUMAN | 24 kDa | TRUE | 49% | 7 | 9 | 10 |
| 1091 | RAS protein activator like-3 OS=Homo sapiens GN=RASAL3 PE=1 SV=2 | RASL3_HUMAN | 112 kDa |  | 4.90% | 4 | 6 | 6 |
| 1092 | E3 ubiquitin-protein ligase TRIM21 OS=Homo sapiens GN=TRIM21 PE=1 SV=1 | RO52_HUMAN | 54 kDa | TRUE | 20% | 7 | 7 | 7 |
| 1093 | 40S ribosomal protein S10 OS=Homo sapiens GN=RPS10 PE=1 SV=1 | RS10_HUMAN | 19 kDa |  | 29% | 4 | 5 | 5 |
| 1094 | Protein S100-A7 OS=Homo sapiens GN=S100A7 PE=1 SV=4 | S10A7_HUMAN | 11 kDa |  | 43% | 5 | 6 | 9 |
| 1095 | Suppressor of G2 allele of SKP1 homolog OS=Homo sapiens GN=SUGT1 PE=1 SV=3 | SUGT1_HUMAN | ? | TRUE | 0.00% | 8 | 9 | 9 |
| 1096 | E3 ubiquitin/ISG15 ligase TRIM25 OS=Homo sapiens GN=TRIM25 PE=1 SV=2 | TRI25_HUMAN | 71 kDa |  | 11% | 5 | 5 | 6 |
| 1097 | NEDD8-activating enzyme E1 catalytic subunit OS=Homo sapiens GN=UBA3 PE=1 SV=2 | UBA3_HUMAN | 52 kDa |  | 12% | 4 | 5 | 5 |
| 1098 | E3 UFM1-protein ligase 1 OS=Homo sapiens GN=UFL1 PE=1 SV=2 | UFL1_HUMAN | 90 kDa | TRUE | 7.60% | 5 | 5 | 6 |
| 1099 | Fibulin-2 OS=Homo sapiens GN=FBLN2 PE=1 SV=2 | FBLN2_HUMAN | 127 kDa |  | 2.30% | 3 | 3 | 3 |
| 1100 | U6 snRNA-associated Sm-like protein LSm2 OS=Homo sapiens GN=LSM2 PE=1 SV=1 | LSM2_HUMAN | 11 kDa |  | 47% | 4 | 7 | 7 |
| 1101 | Ubiquitin-like modifier-activating enzyme 5 OS=Homo sapiens GN=UBA5 PE=1 SV=1 | UBA5_HUMAN | 45 kDa |  | 13% | 3 | 4 | 4 |
| 1102 | Complement C1r subcomponent-like protein OS=Homo sapiens GN=C1RL PE=1 SV=2 | C1RL_HUMAN | 53 kDa | TRUE | 9.20% | 3 | 5 | 14 |
| 1103 | Ig kappa chain V-III region VG (Fragment) OS=Homo sapiens PE=1 SV=1 | KV309_HUMAN | 13 kDa | TRUE | 32% | 2 | 4 | 13 |
| 1104 | Inositol hexakisphosphate and diphosphoinositol-pentakisphosphate kinase 2 OS=Homo sapiens GN=PPIP5K2 PE=1 SV=3 | VIP2_HUMAN | 140 kDa |  | 1.90% | 2 | 2 | 2 |
| 1105 | Serine/threonine-protein phosphatase 2A 55 kDa regulatory subunit B alpha isoform OS=Homo sapiens GN=PPP2R2A PE=1 SV=1 | 2ABA_HUMAN | 52 kDa |  | 14% | 5 | 6 | 6 |
| 1106 | ATP-binding cassette sub-family F member 1 OS=Homo sapiens GN=ABCF1 PE=1 SV=2 | ABCF1_HUMAN | 96 kDa | TRUE | 10% | 7 | 7 | 8 |
| 1107 | Long-chain-fatty-acid--CoA ligase 4 OS=Homo sapiens GN=ACSL4 PE=1 SV=2 | ACSL4_HUMAN | 79 kDa |  | 8.60% | 4 | 4 | 4 |
| 1108 | Beta-adducin OS=Homo sapiens GN=ADD2 PE=1 SV=3 | ADDB_HUMAN | 81 kDa | TRUE | 4.50% | 3 | 4 | 4 |
| 1109 | Activator of 90 kDa heat shock protein ATPase homolog 1 OS=Homo sapiens GN=AHSA1 PE=1 SV=1 | AHSA1_HUMAN | 38 kDa | TRUE | 13% | 3 | 3 | 4 |
| 1110 | Aldehyde dehydrogenase family 1 member A3 OS=Homo sapiens GN=ALDH1A3 PE=1 SV=2 | AL1A3_HUMAN | 56 kDa | TRUE | 16% | 7 | 9 | 11 |
| 1111 | Rabankyrin-5 OS=Homo sapiens GN=ANKFY1 PE=1 SV=2 | ANFY1_HUMAN | 128 kDa |  | 3.80% | 4 | 4 | 4 |
| 1112 | Apolipoprotein L2 OS=Homo sapiens GN=APOL2 PE=1 SV=1 | APOL2_HUMAN | 37 kDa |  | 19% | 7 | 8 | 9 |
| 1113 | Arginase-1 OS=Homo sapiens GN=ARG1 PE=1 SV=2 | ARGI1_HUMAN | 35 kDa |  | 19% | 5 | 5 | 5 |
| 1114 | ATP synthase subunit beta, mitochondrial OS=Homo sapiens GN=ATP5B PE=1 SV=3 | ATPB_HUMAN | 57 kDa | TRUE | 16% | 6 | 7 | 7 |
| 1115 | Caldesmon OS=Homo sapiens GN=CALD1 PE=1 SV=3 | CALD1_HUMAN | 93 kDa | TRUE | 5.90% | 4 | 6 | 6 |
| 1116 | Cathepsin Z OS=Homo sapiens GN=CTSZ PE=1 SV=1 | CATZ_HUMAN | 34 kDa |  | 18% | 5 | 7 | 8 |
| 1117 | CD109 antigen OS=Homo sapiens GN=CD109 PE=1 SV=2 | CD109_HUMAN | 162 kDa | TRUE | 3.00% | 3 | 3 | 3 |
| 1118 | Chromogranin-A OS=Homo sapiens GN=CHGA PE=1 SV=7 | CMGA_HUMAN | 51 kDa | TRUE | 10% | 3 | 4 | 4 |
| 1119 | Collectin-11 OS=Homo sapiens GN=COLEC11 PE=1 SV=1 | COL11_HUMAN | 29 kDa |  | 24% | 5 | 8 | 9 |
| 1120 | Crk-like protein OS=Homo sapiens GN=CRKL PE=1 SV=1 | CRKL_HUMAN | 34 kDa |  | 33% | 7 | 8 | 8 |
| 1121 | Cold shock domain-containing protein E1 OS=Homo sapiens GN=CSDE1 PE=1 SV=2 | CSDE1_HUMAN | 89 kDa |  | 6.60% | 5 | 5 | 5 |
| 1122 | Transcription elongation factor B polypeptide 2 OS=Homo sapiens GN=TCEB2 PE=1 SV=1 | ELOB_HUMAN | 13 kDa |  | 27% | 5 | 7 | 8 |
| 1123 | Transcription elongation factor B polypeptide 1 OS=Homo sapiens GN=TCEB1 PE=1 SV=1 | ELOC_HUMAN | 12 kDa |  | 51% | 5 | 8 | 8 |
| 1124 | 6-phosphofructo-2-kinase/fructose-2,6-bisphosphatase 2 OS=Homo sapiens GN=PFKFB2 PE=1 SV=2 | F262_HUMAN | 58 kDa | TRUE | 9.90% | 4 | 4 | 4 |
| 1125 | WASH complex subunit FAM21C OS=Homo sapiens GN=FAM21C PE=1 SV=3 | FA21C_HUMAN | 145 kDa | TRUE | 5.00% | 4 | 4 | 4 |
| 1126 | Tyrosine-protein kinase Fgr OS=Homo sapiens GN=FGR PE=1 SV=2 | FGR_HUMAN | 59 kDa | TRUE | 16% | 6 | 6 | 7 |
| 1127 | Guanylate-binding protein 5 OS=Homo sapiens GN=GBP5 PE=1 SV=1 | GBP5_HUMAN | 67 kDa | TRUE | 15% | 5 | 6 | 8 |
| 1128 | Putative glycerol kinase 3 OS=Homo sapiens GN=GK3P PE=5 SV=2 | GLPK3_HUMAN | 61 kDa | TRUE | 9.80% | 5 | 5 | 5 |
| 1129 | Glutathione reductase, mitochondrial OS=Homo sapiens GN=GSR PE=1 SV=2 | GSHR_HUMAN | 56 kDa |  | 12% | 5 | 6 | 6 |
| 1130 | Beta-hexosaminidase subunit alpha OS=Homo sapiens GN=HEXA PE=1 SV=2 | HEXA_HUMAN | 61 kDa |  | 6.60% | 4 | 5 | 5 |
| 1131 | Heme oxygenase 1 OS=Homo sapiens GN=HMOX1 PE=1 SV=1 | HMOX1_HUMAN | 33 kDa |  | 24% | 6 | 6 | 7 |
| 1132 | E3 ubiquitin-protein ligase HUWE1 OS=Homo sapiens GN=HUWE1 PE=1 SV=3 | HUWE1_HUMAN | 482 kDa | TRUE | 1.30% | 4 | 4 | 4 |
| 1133 | Interleukin-18 OS=Homo sapiens GN=IL18 PE=1 SV=1 | IL18_HUMAN | 22 kDa |  | 23% | 4 | 4 | 5 |
| 1134 | Ribosomal protein S6 kinase alpha-1 OS=Homo sapiens GN=RPS6KA1 PE=1 SV=2 | KS6A1_HUMAN | 83 kDa | TRUE | 10% | 4 | 4 | 10 |
| 1135 | Galectin-7 OS=Homo sapiens GN=LGALS7 PE=1 SV=2 | LEG7_HUMAN | 15 kDa |  | 46% | 5 | 7 | 9 |
| 1136 | Nascent polypeptide-associated complex subunit alpha, muscle-specific form OS=Homo sapiens GN=NACA PE=1 SV=1 | NACAM_HUMAN | 205 kDa |  | 2.10% | 3 | 4 | 4 |
| 1137 | Nitrilase homolog 1 OS=Homo sapiens GN=NIT1 PE=1 SV=2 | NIT1_HUMAN | 36 kDa |  | 14% | 4 | 6 | 7 |
| 1138 | Ubiquitin thioesterase OTUB1 OS=Homo sapiens GN=OTUB1 PE=1 SV=2 | OTUB1_HUMAN | 31 kDa |  | 25% | 5 | 6 | 7 |
| 1139 | Phospholipase B-like 1 OS=Homo sapiens GN=PLBD1 PE=1 SV=2 | PLBL1_HUMAN | 63 kDa |  | 7.80% | 3 | 4 | 5 |
| 1140 | 1-phosphatidylinositol 4,5-bisphosphate phosphodiesterase beta-2 OS=Homo sapiens GN=PLCB2 PE=1 SV=2 | PLCB2_HUMAN | 134 kDa |  | 5.10% | 5 | 6 | 7 |
| 1141 | Phosphatidylinositol 3,4,5-trisphosphate-dependent Rac exchanger 1 protein OS=Homo sapiens GN=PREX1 PE=1 SV=3 | PREX1_HUMAN | 186 kDa |  | 5.20% | 6 | 6 | 6 |
| 1142 | Tyrosine-protein phosphatase non-receptor type 12 OS=Homo sapiens GN=PTPN12 PE=1 SV=3 | PTN12_HUMAN | 88 kDa |  | 11% | 6 | 6 | 6 |
| 1143 | Ras-related protein Rab-2A OS=Homo sapiens GN=RAB2A PE=1 SV=1 | RAB2A_HUMAN | 24 kDa |  | 28% | 5 | 8 | 8 |
| 1144 | RNA-binding protein 8A OS=Homo sapiens GN=RBM8A PE=1 SV=1 | RBM8A_HUMAN | 20 kDa |  | 45% | 6 | 8 | 10 |
| 1145 | Replication protein A 70 kDa DNA-binding subunit OS=Homo sapiens GN=RPA1 PE=1 SV=2 | RFA1_HUMAN | 68 kDa |  | 12% | 5 | 6 | 6 |
| 1146 | Rab-interacting lysosomal protein OS=Homo sapiens GN=RILP PE=1 SV=1 | RILP_HUMAN | 44 kDa |  | 20% | 6 | 7 | 8 |
| 1147 | 60S ribosomal protein L18a OS=Homo sapiens GN=RPL18A PE=1 SV=2 | RL18A_HUMAN | 21 kDa |  | 22% | 4 | 4 | 4 |
| 1148 | 40S ribosomal protein S8 OS=Homo sapiens GN=RPS8 PE=1 SV=2 | RS8_HUMAN | 24 kDa |  | 28% | 5 | 6 | 6 |
| 1149 | Ras suppressor protein 1 OS=Homo sapiens GN=RSU1 PE=1 SV=3 | RSU1_HUMAN | 32 kDa |  | 17% | 4 | 5 | 6 |
| 1150 | SUMO-activating enzyme subunit 1 OS=Homo sapiens GN=SAE1 PE=1 SV=1 | SAE1_HUMAN | 38 kDa |  | 31% | 8 | 9 | 10 |
| 1151 | GTP-binding protein SAR1b OS=Homo sapiens GN=SAR1B PE=1 SV=1 | SAR1B_HUMAN | 22 kDa | TRUE | 35% | 6 | 6 | 6 |
| 1152 | Sex hormone-binding globulin OS=Homo sapiens GN=SHBG PE=1 SV=2 | SHBG_HUMAN | 44 kDa |  | 22% | 7 | 9 | 9 |
| 1153 | Serpin B10 OS=Homo sapiens GN=SERPINB10 PE=1 SV=1 | SPB10_HUMAN | 45 kDa |  | 14% | 5 | 5 | 6 |
| 1154 | Cysteine--tRNA ligase, cytoplasmic OS=Homo sapiens GN=CARS PE=1 SV=3 | SYCC_HUMAN | 85 kDa |  | 8.60% | 6 | 6 | 7 |
| 1155 | Tubulin beta-3 chain OS=Homo sapiens GN=TUBB3 PE=1 SV=2 | TBB3_HUMAN | 50 kDa | TRUE | 50% | 6 | 7 | 81 |
| 1156 | Protein TFG OS=Homo sapiens GN=TFG PE=1 SV=2 | TFG_HUMAN | 43 kDa |  | 14% | 5 | 6 | 6 |
| 1157 | TIP41-like protein OS=Homo sapiens GN=TIPRL PE=1 SV=2 | TIPRL_HUMAN | 31 kDa |  | 24% | 6 | 6 | 6 |
| 1158 | Tumor protein D54 OS=Homo sapiens GN=TPD52L2 PE=1 SV=2 | TPD54_HUMAN | 22 kDa |  | 29% | 4 | 5 | 6 |
| 1159 | Cystatin-B OS=Homo sapiens GN=CSTB PE=1 SV=2 | CYTB_HUMAN | 11 kDa |  | 46% | 3 | 5 | 6 |
| 1160 | WD repeat-containing protein 44 OS=Homo sapiens GN=WDR44 PE=1 SV=1 | WDR44_HUMAN | 101 kDa |  | 3.50% | 2 | 2 | 3 |
| 1161 | Serine/arginine-rich splicing factor 2 OS=Homo sapiens GN=SRSF2 PE=1 SV=4 | SRSF2_HUMAN | 25 kDa |  | 14% | 3 | 4 | 5 |
| 1162 | Serine/threonine-protein phosphatase 2A 56 kDa regulatory subunit delta isoform OS=Homo sapiens GN=PPP2R5D PE=1 SV=1 | 2A5D_HUMAN | 70 kDa | TRUE | 2.80% | 2 | 2 | 2 |
| 1163 | Sialic acid synthase OS=Homo sapiens GN=NANS PE=1 SV=2 | SIAS_HUMAN | 40 kDa | TRUE | 12% | 2 | 2 | 3 |
| 1164 | 60S ribosomal protein L19 OS=Homo sapiens GN=RPL19 PE=1 SV=1 | RL19_HUMAN | 23 kDa |  | 12% | 3 | 5 | 6 |
| 1165 | Ig heavy chain V-I region V35 OS=Homo sapiens PE=1 SV=1 | HV103_HUMAN | 13 kDa | TRUE | 27% | 2 | 5 | 10 |
| 1166 | Transcriptional activator protein Pur-beta OS=Homo sapiens GN=PURB PE=1 SV=3 | PURB_HUMAN | 33 kDa | TRUE | 6.40% | 2 | 2 | 2 |
| 1167 | 60S ribosomal protein L34 OS=Homo sapiens GN=RPL34 PE=1 SV=3 | RL34_HUMAN | 13 kDa |  | 21% | 3 | 5 | 7 |
| 1168 | Ig kappa chain V-III region GOL OS=Homo sapiens PE=1 SV=1 | KV307_HUMAN | 12 kDa | TRUE | 61% | 2 | 3 | 39 |
| 1169 | Cytosolic 5'-nucleotidase 3A OS=Homo sapiens GN=NT5C3A PE=1 SV=3 | 5NT3A_HUMAN | 38 kDa | TRUE | 28% | 7 | 8 | 8 |
| 1170 | Protein ABHD14B OS=Homo sapiens GN=ABHD14B PE=1 SV=1 | ABHEB_HUMAN | 22 kDa |  | 33% | 5 | 7 | 7 |
| 1171 | Peroxisomal acyl-coenzyme A oxidase 1 OS=Homo sapiens GN=ACOX1 PE=1 SV=3 | ACOX1_HUMAN | 74 kDa |  | 20% | 7 | 7 | 8 |
| 1172 | Long-chain-fatty-acid--CoA ligase 1 OS=Homo sapiens GN=ACSL1 PE=1 SV=1 | ACSL1_HUMAN | 78 kDa | TRUE | 12% | 7 | 7 | 7 |
| 1173 | Beta-centractin OS=Homo sapiens GN=ACTR1B PE=1 SV=1 | ACTY_HUMAN | 42 kDa | TRUE | 32% | 3 | 6 | 17 |
| 1174 | Arfaptin-1 OS=Homo sapiens GN=ARFIP1 PE=1 SV=2 | ARFP1_HUMAN | 42 kDa |  | 12% | 4 | 4 | 4 |
| 1175 | COP9 signalosome complex subunit 7a OS=Homo sapiens GN=COPS7A PE=1 SV=1 | CSN7A_HUMAN | 30 kDa |  | 31% | 6 | 8 | 9 |
| 1176 | Probable ATP-dependent RNA helicase DDX6 OS=Homo sapiens GN=DDX6 PE=1 SV=2 | DDX6_HUMAN | 54 kDa |  | 18% | 6 | 6 | 7 |
| 1177 | Protein diaphanous homolog 2 OS=Homo sapiens GN=DIAPH2 PE=1 SV=1 | DIAP2_HUMAN | 126 kDa | TRUE | 4.00% | 4 | 4 | 4 |
| 1178 | DnaJ homolog subfamily A member 1 OS=Homo sapiens GN=DNAJA1 PE=1 SV=2 | DNJA1_HUMAN | 45 kDa | TRUE | 20% | 5 | 6 | 6 |
| 1179 | Dipeptidyl peptidase 3 OS=Homo sapiens GN=DPP3 PE=1 SV=2 | DPP3_HUMAN | 83 kDa |  | 10% | 6 | 6 | 6 |
| 1180 | Protein FAM114A2 OS=Homo sapiens GN=FAM114A2 PE=1 SV=4 | F1142_HUMAN | 55 kDa |  | 11% | 5 | 6 | 6 |
| 1181 | Flap endonuclease 1 OS=Homo sapiens GN=FEN1 PE=1 SV=1 | FEN1_HUMAN | 43 kDa | TRUE | 15% | 4 | 4 | 4 |
| 1182 | FYVE, RhoGEF and PH domain-containing protein 3 OS=Homo sapiens GN=FGD3 PE=1 SV=1 | FGD3_HUMAN | 79 kDa |  | 7.30% | 4 | 5 | 6 |
| 1183 | Follistatin-related protein 1 OS=Homo sapiens GN=FSTL1 PE=1 SV=1 | FSTL1_HUMAN | 35 kDa |  | 15% | 4 | 4 | 6 |
| 1184 | Rap1 GTPase-GDP dissociation stimulator 1 OS=Homo sapiens GN=RAP1GDS1 PE=1 SV=3 | GDS1_HUMAN | 66 kDa |  | 16% | 8 | 8 | 8 |
| 1185 | Mannose-1-phosphate guanyltransferase alpha OS=Homo sapiens GN=GMPPA PE=1 SV=1 | GMPPA_HUMAN | 46 kDa |  | 20% | 7 | 8 | 8 |
| 1186 | Hepatocyte growth factor-regulated tyrosine kinase substrate OS=Homo sapiens GN=HGS PE=1 SV=1 | HGS_HUMAN | 86 kDa |  | 5.30% | 4 | 5 | 6 |
| 1187 | cAMP-dependent protein kinase catalytic subunit alpha OS=Homo sapiens GN=PRKACA PE=1 SV=2 | KAPCA_HUMAN | 41 kDa |  | 11% | 4 | 5 | 5 |
| 1188 | Creatine kinase B-type OS=Homo sapiens GN=CKB PE=1 SV=1 | KCRB_HUMAN | 43 kDa |  | 13% | 4 | 4 | 5 |
| 1189 | Putative RNA-binding protein Luc7-like 2 OS=Homo sapiens GN=LUC7L2 PE=1 SV=2 | LC7L2_HUMAN | 47 kDa | TRUE | 14% | 5 | 5 | 6 |
| 1190 | Leukocyte immunoglobulin-like receptor subfamily A member 3 OS=Homo sapiens GN=LILRA3 PE=1 SV=3 | LIRA3_HUMAN | 47 kDa |  | 20% | 6 | 7 | 8 |
| 1191 | U6 snRNA-associated Sm-like protein LSm1 OS=Homo sapiens GN=LSM1 PE=1 SV=1 | LSM1_HUMAN | 15 kDa |  | 37% | 3 | 3 | 4 |
| 1192 | Tyrosine-protein kinase Lyn OS=Homo sapiens GN=LYN PE=1 SV=3 | LYN_HUMAN | 59 kDa | TRUE | 15% | 3 | 4 | 7 |
| 1193 | Mannose-binding protein C OS=Homo sapiens GN=MBL2 PE=1 SV=2 | MBL2_HUMAN | 26 kDa |  | 18% | 4 | 5 | 8 |
| 1194 | Collagenase 3 OS=Homo sapiens GN=MMP13 PE=1 SV=1 | MMP13_HUMAN | 54 kDa |  | 10% | 4 | 4 | 4 |
| 1195 | 72 kDa type IV collagenase OS=Homo sapiens GN=MMP2 PE=1 SV=2 | MMP2_HUMAN | 74 kDa |  | 9.40% | 5 | 7 | 7 |
| 1196 | Methylthioribose-1-phosphate isomerase OS=Homo sapiens GN=MRI1 PE=1 SV=1 | MTNA_HUMAN | 39 kDa |  | 17% | 6 | 6 | 8 |
| 1197 | Myosin light chain 4 OS=Homo sapiens GN=MYL4 PE=1 SV=3 | MYL4_HUMAN | 22 kDa |  | 27% | 5 | 6 | 6 |
| 1198 | Protein phosphatase 1 regulatory subunit 12A OS=Homo sapiens GN=PPP1R12A PE=1 SV=1 | MYPT1_HUMAN | 115 kDa |  | 8.50% | 7 | 7 | 7 |
| 1199 | Na(+)/H(+) exchange regulatory cofactor NHE-RF1 OS=Homo sapiens GN=SLC9A3R1 PE=1 SV=4 | NHRF1_HUMAN | 39 kDa | TRUE | 14% | 4 | 5 | 6 |
| 1200 | Optineurin OS=Homo sapiens GN=OPTN PE=1 SV=2 | OPTN_HUMAN | 66 kDa | TRUE | 10% | 6 | 7 | 7 |
| 1201 | Probable tRNA N6-adenosine threonylcarbamoyltransferase OS=Homo sapiens GN=OSGEP PE=1 SV=1 | OSGEP_HUMAN | 36 kDa |  | 24% | 6 | 6 | 8 |
| 1202 | Prefoldin subunit 2 OS=Homo sapiens GN=PFDN2 PE=1 SV=1 | PFD2_HUMAN | 17 kDa |  | 34% | 4 | 7 | 7 |
| 1203 | Polymeric immunoglobulin receptor OS=Homo sapiens GN=PIGR PE=1 SV=4 | PIGR_HUMAN | 83 kDa |  | 5.80% | 4 | 5 | 5 |
| 1204 | Phosphomannomutase 2 OS=Homo sapiens GN=PMM2 PE=1 SV=1 | PMM2_HUMAN | 28 kDa |  | 17% | 5 | 5 | 5 |
| 1205 | Ras-related protein Rab-21 OS=Homo sapiens GN=RAB21 PE=1 SV=3 | RAB21_HUMAN | 24 kDa |  | 18% | 3 | 5 | 6 |
| 1206 | 60S ribosomal protein L22 OS=Homo sapiens GN=RPL22 PE=1 SV=2 | RL22_HUMAN | 15 kDa | TRUE | 30% | 3 | 5 | 6 |
| 1207 | SAM and SH3 domain-containing protein 3 OS=Homo sapiens GN=SASH3 PE=1 SV=2 | SASH3_HUMAN | 42 kDa |  | 17% | 4 | 6 | 7 |
| 1208 | Protein transport protein Sec24C OS=Homo sapiens GN=SEC24C PE=1 SV=3 | SC24C_HUMAN | 118 kDa | TRUE | 7.20% | 5 | 5 | 5 |
| 1209 | Secernin-1 OS=Homo sapiens GN=SCRN1 PE=1 SV=2 | SCRN1_HUMAN | 46 kDa | TRUE | 12% | 3 | 5 | 5 |
| 1210 | D-3-phosphoglycerate dehydrogenase OS=Homo sapiens GN=PHGDH PE=1 SV=4 | SERA_HUMAN | 57 kDa |  | 15% | 6 | 6 | 6 |
| 1211 | Serine/arginine-rich splicing factor 7 OS=Homo sapiens GN=SRSF7 PE=1 SV=1 | SRSF7_HUMAN | 27 kDa | TRUE | 18% | 4 | 5 | 5 |
| 1212 | Serine/threonine-protein kinase 24 OS=Homo sapiens GN=STK24 PE=1 SV=1 | STK24_HUMAN | 49 kDa | TRUE | 13% | 5 | 6 | 7 |
| 1213 | Tubulin beta-1 chain OS=Homo sapiens GN=TUBB1 PE=1 SV=1 | TBB1_HUMAN | 50 kDa | TRUE | 22% | 5 | 6 | 38 |
| 1214 | Tubulin-folding cofactor B OS=Homo sapiens GN=TBCB PE=1 SV=2 | TBCB_HUMAN | 27 kDa | TRUE | 21% | 5 | 5 | 6 |
| 1215 | Tubulin-specific chaperone cofactor E-like protein OS=Homo sapiens GN=TBCEL PE=1 SV=2 | TBCEL_HUMAN | 48 kDa |  | 11% | 4 | 5 | 7 |
| 1216 | Transportin-1 OS=Homo sapiens GN=TNPO1 PE=1 SV=2 | TNPO1_HUMAN | 102 kDa |  | 6.60% | 5 | 5 | 5 |
| 1217 | Thioredoxin reductase 1, cytoplasmic OS=Homo sapiens GN=TXNRD1 PE=1 SV=3 | TRXR1_HUMAN | 71 kDa |  | 6.80% | 3 | 3 | 3 |
| 1218 | Tumor necrosis factor-inducible gene 6 protein OS=Homo sapiens GN=TNFAIP6 PE=1 SV=2 | TSG6_HUMAN | 31 kDa |  | 19% | 5 | 7 | 7 |
| 1219 | Translin OS=Homo sapiens GN=TSN PE=1 SV=1 | TSN_HUMAN | 26 kDa | TRUE | 21% | 3 | 4 | 5 |
| 1220 | U5 small nuclear ribonucleoprotein 200 kDa helicase OS=Homo sapiens GN=SNRNP200 PE=1 SV=2 | U520_HUMAN | 245 kDa |  | 3.50% | 5 | 5 | 5 |
| 1221 | Ubiquitin carboxyl-terminal hydrolase 47 OS=Homo sapiens GN=USP47 PE=1 SV=3 | UBP47_HUMAN | 157 kDa |  | 6.00% | 5 | 5 | 6 |
| 1222 | Ubiquilin-1 OS=Homo sapiens GN=UBQLN1 PE=1 SV=2 | UBQL1_HUMAN | 63 kDa | TRUE | 12% | 5 | 7 | 7 |
| 1223 | Proto-oncogene vav OS=Homo sapiens GN=VAV1 PE=1 SV=4 | VAV_HUMAN | 98 kDa |  | 5.70% | 5 | 5 | 5 |
| 1224 | WAS/WASL-interacting protein family member 1 OS=Homo sapiens GN=WIPF1 PE=1 SV=3 | WIPF1_HUMAN | 51 kDa |  | 9.10% | 4 | 5 | 6 |
| 1225 | Enhancer of rudimentary homolog OS=Homo sapiens GN=ERH PE=1 SV=1 | ERH_HUMAN | 12 kDa |  | 40% | 4 | 6 | 8 |
| 1226 | Thymidylate kinase OS=Homo sapiens GN=DTYMK PE=1 SV=4 | KTHY_HUMAN | 24 kDa | TRUE | 17% | 4 | 5 | 5 |
| 1227 | Nucleolar protein 58 OS=Homo sapiens GN=NOP58 PE=1 SV=1 | NOP58_HUMAN | 60 kDa |  | 8.30% | 3 | 3 | 3 |
| 1228 | STAM-binding protein OS=Homo sapiens GN=STAMBP PE=1 SV=1 | STABP_HUMAN | 48 kDa | TRUE | 11% | 3 | 3 | 3 |
| 1229 | SEC23-interacting protein OS=Homo sapiens GN=SEC23IP PE=1 SV=1 | S23IP_HUMAN | 111 kDa | TRUE | 3.90% | 3 | 3 | 3 |
| 1230 | Collagen alpha-1(I) chain OS=Homo sapiens GN=COL1A1 PE=1 SV=5 | CO1A1_HUMAN | 139 kDa |  | 1.40% | 2 | 2 | 2 |
| 1231 | V-type proton ATPase subunit F OS=Homo sapiens GN=ATP6V1F PE=1 SV=2 | VATF_HUMAN | 13 kDa | TRUE | 42% | 3 | 5 | 8 |
| 1232 | Leucine-rich repeat-containing protein 47 OS=Homo sapiens GN=LRRC47 PE=1 SV=1 | LRC47_HUMAN | 63 kDa | TRUE | 5.50% | 3 | 3 | 3 |
| 1233 | Insulin-like growth factor-binding protein 5 OS=Homo sapiens GN=IGFBP5 PE=1 SV=1 | IBP5_HUMAN | 31 kDa | TRUE | 6.60% | 2 | 2 | 2 |
| 1234 | Kinesin-like protein KIF1C OS=Homo sapiens GN=KIF1C PE=1 SV=3 | KIF1C_HUMAN | 123 kDa | TRUE | 2.30% | 2 | 2 | 3 |
| 1235 | FYN-binding protein OS=Homo sapiens GN=FYB PE=1 SV=2 | FYB_HUMAN | 85 kDa |  | 5.20% | 3 | 3 | 3 |
| 1236 | ADP-ribosylation factor 5 OS=Homo sapiens GN=ARF5 PE=1 SV=2 | ARF5_HUMAN | 21 kDa | TRUE | 37% | 3 | 5 | 14 |
| 1237 | Ras-related protein Rab-8B OS=Homo sapiens GN=RAB8B PE=1 SV=2 | RAB8B_HUMAN | 24 kDa | TRUE | 37% | 3 | 5 | 13 |
| 1238 | Phosphorylase b kinase gamma catalytic chain, liver/testis isoform OS=Homo sapiens GN=PHKG2 PE=1 SV=1 | PHKG2_HUMAN | 46 kDa |  | 5.70% | 2 | 2 | 2 |
| 1239 | Translational activator GCN1 OS=Homo sapiens GN=GCN1L1 PE=1 SV=6 | GCN1L_HUMAN | ? | TRUE | 0.00% | 2 | 2 | 2 |
| 1240 | E3 ubiquitin-protein ligase LRSAM1 OS=Homo sapiens GN=LRSAM1 PE=1 SV=1 | LRSM1_HUMAN | 84 kDa | TRUE | 3.50% | 2 | 2 | 2 |
| 1241 | Phosphoacetylglucosamine mutase OS=Homo sapiens GN=PGM3 PE=1 SV=1 | AGM1_HUMAN | 60 kDa |  | 10% | 5 | 6 | 7 |
| 1242 | Apolipoprotein(a) OS=Homo sapiens GN=LPA PE=1 SV=1 | APOA_HUMAN | 501 kDa |  | 1.30% | 5 | 5 | 7 |
| 1243 | ADP-ribosylation factor 4 OS=Homo sapiens GN=ARF4 PE=1 SV=3 | ARF4_HUMAN | 21 kDa | TRUE | 37% | 4 | 6 | 12 |
| 1244 | Bone morphogenetic protein 1 OS=Homo sapiens GN=BMP1 PE=1 SV=2 | BMP1_HUMAN | 111 kDa |  | 6.20% | 4 | 5 | 5 |
| 1245 | Calcium-binding protein 39 OS=Homo sapiens GN=CAB39 PE=1 SV=1 | CAB39_HUMAN | 40 kDa |  | 13% | 5 | 7 | 7 |
| 1246 | Carbonyl reductase [NADPH] 1 OS=Homo sapiens GN=CBR1 PE=1 SV=3 | CBR1_HUMAN | 30 kDa |  | 25% | 4 | 5 | 5 |
| 1247 | Charged multivesicular body protein 2a OS=Homo sapiens GN=CHMP2A PE=1 SV=1 | CHM2A_HUMAN | 25 kDa |  | 16% | 3 | 4 | 4 |
| 1248 | Copine-1 OS=Homo sapiens GN=CPNE1 PE=1 SV=1 | CPNE1_HUMAN | 59 kDa |  | 16% | 7 | 8 | 8 |
| 1249 | Adapter molecule crk OS=Homo sapiens GN=CRK PE=1 SV=2 | CRK_HUMAN | 34 kDa |  | 15% | 4 | 4 | 4 |
| 1250 | Cytoplasmic dynein 1 light intermediate chain 1 OS=Homo sapiens GN=DYNC1LI1 PE=1 SV=3 | DC1L1_HUMAN | 57 kDa |  | 8.40% | 3 | 4 | 4 |
| 1251 | DnaJ homolog subfamily B member 2 OS=Homo sapiens GN=DNAJB2 PE=1 SV=3 | DNJB2_HUMAN | 36 kDa |  | 17% | 5 | 5 | 5 |
| 1252 | Ethylmalonyl-CoA decarboxylase OS=Homo sapiens GN=ECHDC1 PE=1 SV=2 | ECHD1_HUMAN | 34 kDa | TRUE | 19% | 4 | 4 | 4 |
| 1253 | Fibulin-5 OS=Homo sapiens GN=FBLN5 PE=1 SV=1 | FBLN5_HUMAN | 50 kDa | TRUE | 11% | 5 | 7 | 7 |
| 1254 | Farnesyl pyrophosphate synthase OS=Homo sapiens GN=FDPS PE=1 SV=4 | FPPS_HUMAN | 48 kDa |  | 14% | 5 | 7 | 7 |
| 1255 | GTPase-activating protein and VPS9 domain-containing protein 1 OS=Homo sapiens GN=GAPVD1 PE=1 SV=2 | GAPD1_HUMAN | 165 kDa | TRUE | 3.70% | 5 | 5 | 5 |
| 1256 | ARF GTPase-activating protein GIT2 OS=Homo sapiens GN=GIT2 PE=1 SV=2 | GIT2_HUMAN | 85 kDa |  | 7.00% | 4 | 5 | 5 |
| 1257 | Histone H1x OS=Homo sapiens GN=H1FX PE=1 SV=1 | H1X_HUMAN | 22 kDa |  | 23% | 4 | 6 | 7 |
| 1258 | Heterogeneous nuclear ribonucleoprotein H3 OS=Homo sapiens GN=HNRNPH3 PE=1 SV=2 | HNRH3_HUMAN | 37 kDa | TRUE | 18% | 5 | 5 | 7 |
| 1259 | Interferon-induced 35 kDa protein OS=Homo sapiens GN=IFI35 PE=1 SV=5 | IN35_HUMAN | 32 kDa |  | 24% | 5 | 5 | 5 |
| 1260 | Importin-7 OS=Homo sapiens GN=IPO7 PE=1 SV=1 | IPO7_HUMAN | 120 kDa |  | 5.90% | 5 | 6 | 8 |
| 1261 | Latexin OS=Homo sapiens GN=LXN PE=1 SV=2 | LXN_HUMAN | 26 kDa |  | 17% | 3 | 4 | 5 |
| 1262 | Alpha-mannosidase 2C1 OS=Homo sapiens GN=MAN2C1 PE=1 SV=1 | MA2C1_HUMAN | 116 kDa |  | 5.00% | 4 | 4 | 5 |
| 1263 | Methionine adenosyltransferase 2 subunit beta OS=Homo sapiens GN=MAT2B PE=1 SV=1 | MAT2B_HUMAN | 38 kDa |  | 10% | 3 | 3 | 3 |
| 1264 | Methylosome protein 50 OS=Homo sapiens GN=WDR77 PE=1 SV=1 | MEP50_HUMAN | 37 kDa |  | 10% | 4 | 5 | 7 |
| 1265 | Mitogen-activated protein kinase 3 OS=Homo sapiens GN=MAPK3 PE=1 SV=4 | MK03_HUMAN | 43 kDa | TRUE | 28% | 5 | 7 | 14 |
| 1266 | NudC domain-containing protein 2 OS=Homo sapiens GN=NUDCD2 PE=1 SV=1 | NUDC2_HUMAN | 18 kDa |  | 35% | 4 | 4 | 4 |
| 1267 | Oncoprotein-induced transcript 3 protein OS=Homo sapiens GN=OIT3 PE=1 SV=2 | OIT3_HUMAN | 60 kDa |  | 4.60% | 2 | 2 | 2 |
| 1268 | Plasminogen activator inhibitor 1 OS=Homo sapiens GN=SERPINE1 PE=1 SV=1 | PAI1_HUMAN | 45 kDa |  | 9.50% | 3 | 4 | 4 |
| 1269 | Serine/threonine-protein kinase PAK 1 OS=Homo sapiens GN=PAK1 PE=1 SV=2 | PAK1_HUMAN | 61 kDa | TRUE | 19% | 6 | 6 | 6 |
| 1270 | Paxillin OS=Homo sapiens GN=PXN PE=1 SV=3 | PAXI_HUMAN | 65 kDa |  | 9.00% | 4 | 5 | 5 |
| 1271 | Astrocytic phosphoprotein PEA-15 OS=Homo sapiens GN=PEA15 PE=1 SV=2 | PEA15_HUMAN | 15 kDa |  | 32% | 3 | 4 | 4 |
| 1272 | Phosphoglycolate phosphatase OS=Homo sapiens GN=PGP PE=1 SV=1 | PGP_HUMAN | 34 kDa |  | 17% | 4 | 5 | 6 |
| 1273 | Geranylgeranyl transferase type-2 subunit alpha OS=Homo sapiens GN=RABGGTA PE=1 SV=2 | PGTA_HUMAN | 65 kDa |  | 13% | 5 | 5 | 5 |
| 1274 | Serine/threonine-protein phosphatase 6 regulatory subunit 3 OS=Homo sapiens GN=PPP6R3 PE=1 SV=2 | PP6R3_HUMAN | 98 kDa | TRUE | 7.90% | 6 | 6 | 7 |
| 1275 | Receptor-type tyrosine-protein phosphatase C OS=Homo sapiens GN=PTPRC PE=1 SV=2 | PTPRC_HUMAN | 147 kDa | TRUE | 4.10% | 5 | 5 | 5 |
| 1276 | Ras-related protein Rab-18 OS=Homo sapiens GN=RAB18 PE=1 SV=1 | RAB18_HUMAN | 23 kDa | TRUE | 33% | 5 | 6 | 6 |
| 1277 | Ras-related protein Rab-31 OS=Homo sapiens GN=RAB31 PE=1 SV=1 | RAB31_HUMAN | 22 kDa |  | 44% | 6 | 6 | 8 |
| 1278 | 60S ribosomal protein L24 OS=Homo sapiens GN=RPL24 PE=1 SV=1 | RL24_HUMAN | 18 kDa |  | 25% | 4 | 5 | 6 |
| 1279 | 40S ribosomal protein S17 OS=Homo sapiens GN=RPS17 PE=1 SV=2 | RS17_HUMAN | 16 kDa | TRUE | 46% | 4 | 4 | 5 |
| 1280 | Adenosylhomocysteinase 2 OS=Homo sapiens GN=AHCYL1 PE=1 SV=2 | SAHH2_HUMAN | 59 kDa | TRUE | 9.20% | 3 | 3 | 6 |
| 1281 | Suprabasin OS=Homo sapiens GN=SBSN PE=1 SV=2 | SBSN_HUMAN | 61 kDa |  | 9.30% | 3 | 4 | 6 |
| 1282 | Septin-5 OS=Homo sapiens GN=SEPT5 PE=1 SV=1 | SEPT5_HUMAN | 43 kDa | TRUE | 8.90% | 3 | 3 | 3 |
| 1283 | Shootin-1 OS=Homo sapiens GN=SHTN1 PE=1 SV=4 | SHOT1_HUMAN | 72 kDa | TRUE | 12% | 6 | 6 | 6 |
| 1284 | Serpin B3 OS=Homo sapiens GN=SERPINB3 PE=1 SV=2 | SPB3_HUMAN | 45 kDa | TRUE | 14% | 4 | 4 | 4 |
| 1285 | Signal recognition particle 14 kDa protein OS=Homo sapiens GN=SRP14 PE=1 SV=2 | SRP14_HUMAN | 15 kDa |  | 24% | 3 | 4 | 4 |
| 1286 | Signal recognition particle 54 kDa protein OS=Homo sapiens GN=SRP54 PE=1 SV=1 | SRP54_HUMAN | 56 kDa |  | 6.30% | 3 | 3 | 3 |
| 1287 | Serine/threonine-protein kinase TAO3 OS=Homo sapiens GN=TAOK3 PE=1 SV=2 | TAOK3_HUMAN | 105 kDa | TRUE | 4.20% | 4 | 4 | 4 |
| 1288 | Tubulin beta-2A chain OS=Homo sapiens GN=TUBB2A PE=1 SV=1 | TBB2A_HUMAN | 50 kDa | TRUE | 59% | 4 | 6 | 105 |
| 1289 | Ubiquitin carboxyl-terminal hydrolase isozyme L5 OS=Homo sapiens GN=UCHL5 PE=1 SV=3 | UCHL5_HUMAN | 38 kDa |  | 14% | 4 | 4 | 4 |
| 1290 | Wiskott-Aldrich syndrome protein OS=Homo sapiens GN=WAS PE=1 SV=4 | WASP_HUMAN | 53 kDa |  | 15% | 6 | 7 | 7 |
| 1291 | WD repeat-containing protein 61 OS=Homo sapiens GN=WDR61 PE=1 SV=1 | WDR61_HUMAN | 34 kDa |  | 15% | 3 | 3 | 3 |
| 1292 | Nuclease-sensitive element-binding protein 1 OS=Homo sapiens GN=YBX1 PE=1 SV=3 | YBOX1_HUMAN | 36 kDa | TRUE | 26% | 3 | 6 | 14 |
| 1293 | Protein PRRC1 OS=Homo sapiens GN=PRRC1 PE=1 SV=1 | PRRC1_HUMAN | 47 kDa | TRUE | 10% | 4 | 4 | 6 |
| 1294 | Sorting nexin-27 OS=Homo sapiens GN=SNX27 PE=1 SV=2 | SNX27_HUMAN | 61 kDa |  | 6.50% | 3 | 4 | 4 |
| 1295 | Probable carboxypeptidase X1 OS=Homo sapiens GN=CPXM1 PE=2 SV=2 | CPXM1_HUMAN | 82 kDa |  | 4.50% | 2 | 2 | 2 |
| 1296 | Brefeldin A-inhibited guanine nucleotide-exchange protein 1 OS=Homo sapiens GN=ARFGEF1 PE=1 SV=2 | BIG1_HUMAN | 209 kDa | TRUE | 1.40% | 2 | 2 | 2 |
| 1297 | BRO1 domain-containing protein BROX OS=Homo sapiens GN=BROX PE=1 SV=1 | BROX_HUMAN | 46 kDa |  | 10% | 3 | 5 | 5 |
| 1298 | Signal recognition particle 9 kDa protein OS=Homo sapiens GN=SRP9 PE=1 SV=2 | SRP09_HUMAN | 10 kDa |  | 40% | 4 | 4 | 4 |
| 1299 | POTE ankyrin domain family member I OS=Homo sapiens GN=POTEI PE=3 SV=1 | POTEI_HUMAN | 121 kDa | TRUE | 14% | 3 | 4 | 103 |
| 1300 | Alpha-2-macroglobulin-like protein 1 OS=Homo sapiens GN=A2ML1 PE=1 SV=3 | A2ML1_HUMAN | 161 kDa |  | 2.80% | 3 | 3 | 3 |
| 1301 | Rho GTPase-activating protein 9 OS=Homo sapiens GN=ARHGAP9 PE=1 SV=2 | RHG09_HUMAN | 83 kDa | TRUE | 3.70% | 3 | 3 | 3 |
| 1302 | Tyrosine-protein kinase Fes/Fps OS=Homo sapiens GN=FES PE=1 SV=3 | FES_HUMAN | 93 kDa |  | 3.80% | 2 | 3 | 3 |
| 1303 | Spermidine synthase OS=Homo sapiens GN=SRM PE=1 SV=1 | SPEE_HUMAN | 34 kDa |  | 7.60% | 2 | 3 | 5 |
| 1304 | FK506-binding protein 15 OS=Homo sapiens GN=FKBP15 PE=1 SV=2 | FKB15_HUMAN | 134 kDa | TRUE | 2.40% | 3 | 4 | 4 |
| 1305 | SH3 and PX domain-containing protein 2B OS=Homo sapiens GN=SH3PXD2B PE=1 SV=3 | SPD2B_HUMAN | 102 kDa |  | 3.00% | 3 | 4 | 5 |
| 1306 | Host cell factor 1 OS=Homo sapiens GN=HCFC1 PE=1 SV=2 | HCFC1_HUMAN | 209 kDa | TRUE | 1.60% | 3 | 4 | 4 |
| 1307 | Histone H2A.V OS=Homo sapiens GN=H2AFV PE=1 SV=3 | H2AV_HUMAN | 14 kDa | TRUE | 31% | 2 | 4 | 28 |
| 1308 | RUN and FYVE domain-containing protein 1 OS=Homo sapiens GN=RUFY1 PE=1 SV=2 | RUFY1_HUMAN | 80 kDa | TRUE | 2.50% | 2 | 2 | 2 |
| 1309 | Bifunctional 3'-phosphoadenosine 5'-phosphosulfate synthase 2 OS=Homo sapiens GN=PAPSS2 PE=1 SV=2 | PAPS2_HUMAN | 70 kDa | TRUE | 5.00% | 2 | 2 | 3 |
| 1310 | Acyl-protein thioesterase 2 OS=Homo sapiens GN=LYPLA2 PE=1 SV=1 | LYPA2_HUMAN | 25 kDa | TRUE | 11% | 2 | 2 | 2 |
| 1311 | 60S ribosomal protein L11 OS=Homo sapiens GN=RPL11 PE=1 SV=2 | RL11_HUMAN | 20 kDa |  | 8.40% | 2 | 2 | 3 |
| 1312 | Ig lambda chain V-II region NEI OS=Homo sapiens PE=1 SV=1 | LV202_HUMAN | 12 kDa | TRUE | 14% | 2 | 3 | 4 |
| 1313 | 6-phosphogluconolactonase OS=Homo sapiens GN=PGLS PE=1 SV=2 | 6PGL_HUMAN | 28 kDa |  | 29% | 5 | 6 | 6 |
| 1314 | Angiopoietin-related protein 6 OS=Homo sapiens GN=ANGPTL6 PE=1 SV=1 | ANGL6_HUMAN | 52 kDa |  | 8.10% | 3 | 3 | 3 |
| 1315 | AP-1 complex subunit sigma-2 OS=Homo sapiens GN=AP1S2 PE=1 SV=1 | AP1S2_HUMAN | 19 kDa |  | 34% | 4 | 5 | 7 |
| 1316 | Apoptotic protease-activating factor 1 OS=Homo sapiens GN=APAF1 PE=1 SV=2 | APAF_HUMAN | 142 kDa | TRUE | 3.60% | 4 | 5 | 5 |
| 1317 | Protein bicaudal D homolog 2 OS=Homo sapiens GN=BICD2 PE=1 SV=1 | BICD2_HUMAN | 94 kDa |  | 5.90% | 4 | 4 | 5 |
| 1318 | Mitotic checkpoint protein BUB3 OS=Homo sapiens GN=BUB3 PE=1 SV=1 | BUB3_HUMAN | 37 kDa |  | 13% | 4 | 4 | 4 |
| 1319 | Caprin-1 OS=Homo sapiens GN=CAPRIN1 PE=1 SV=2 | CAPR1_HUMAN | 78 kDa |  | 7.60% | 5 | 6 | 6 |
| 1320 | Caspase-4 OS=Homo sapiens GN=CASP4 PE=1 SV=1 | CASP4_HUMAN | 43 kDa |  | 13% | 4 | 5 | 5 |
| 1321 | Cholinesterase OS=Homo sapiens GN=BCHE PE=1 SV=1 | CHLE_HUMAN | 68 kDa |  | 9.50% | 4 | 4 | 5 |
| 1322 | Uncharacterized protein C9orf40 OS=Homo sapiens GN=C9orf40 PE=1 SV=1 | CI040_HUMAN | 21 kDa |  | 35% | 3 | 4 | 6 |
| 1323 | Coatomer subunit zeta-1 OS=Homo sapiens GN=COPZ1 PE=1 SV=1 | COPZ1_HUMAN | 20 kDa |  | 32% | 4 | 5 | 6 |
| 1324 | Serine/threonine-protein phosphatase CPPED1 OS=Homo sapiens GN=CPPED1 PE=1 SV=3 | CPPED_HUMAN | 36 kDa |  | 15% | 4 | 5 | 5 |
| 1325 | Probable ATP-dependent RNA helicase DDX58 OS=Homo sapiens GN=DDX58 PE=1 SV=2 | DDX58_HUMAN | 107 kDa | TRUE | 4.30% | 3 | 3 | 3 |
| 1326 | Protein DEK OS=Homo sapiens GN=DEK PE=1 SV=1 | DEK_HUMAN | 43 kDa |  | 12% | 4 | 5 | 5 |
| 1327 | Dedicator of cytokinesis protein 8 OS=Homo sapiens GN=DOCK8 PE=1 SV=3 | DOCK8_HUMAN | 239 kDa | TRUE | 2.50% | 4 | 4 | 4 |
| 1328 | Translation initiation factor eIF-2B subunit beta OS=Homo sapiens GN=EIF2B2 PE=1 SV=3 | EI2BB_HUMAN | 39 kDa |  | 15% | 4 | 4 | 5 |
| 1329 | Eukaryotic translation initiation factor 3 subunit F OS=Homo sapiens GN=EIF3F PE=1 SV=1 | EIF3F_HUMAN | 38 kDa |  | 17% | 4 | 5 | 7 |
| 1330 | Peptidyl-prolyl cis-trans isomerase FKBP10 OS=Homo sapiens GN=FKBP10 PE=1 SV=1 | FKB10_HUMAN | 64 kDa |  | 8.40% | 5 | 5 | 5 |
| 1331 | Heterogeneous nuclear ribonucleoproteins C1/C2 OS=Homo sapiens GN=HNRNPC PE=1 SV=4 | HNRPC_HUMAN | 34 kDa |  | 11% | 3 | 5 | 6 |
| 1332 | Inositol monophosphatase 2 OS=Homo sapiens GN=IMPA2 PE=1 SV=1 | IMPA2_HUMAN | 31 kDa |  | 19% | 4 | 6 | 6 |
| 1333 | Arachidonate 5-lipoxygenase OS=Homo sapiens GN=ALOX5 PE=1 SV=2 | LOX5_HUMAN | 78 kDa | TRUE | 6.10% | 4 | 4 | 4 |
| 1334 | NHL repeat-containing protein 2 OS=Homo sapiens GN=NHLRC2 PE=1 SV=1 | NHLC2_HUMAN | 79 kDa |  | 7.90% | 4 | 4 | 4 |
| 1335 | Nucleosome assembly protein 1-like 1 OS=Homo sapiens GN=NAP1L1 PE=1 SV=1 | NP1L1_HUMAN | 45 kDa | TRUE | 13% | 3 | 5 | 7 |
| 1336 | N-acetylneuraminate lyase OS=Homo sapiens GN=NPL PE=1 SV=1 | NPL_HUMAN | 35 kDa |  | 9.70% | 3 | 5 | 5 |
| 1337 | Nuclear receptor-binding protein OS=Homo sapiens GN=NRBP1 PE=1 SV=1 | NRBP_HUMAN | 60 kDa |  | 11% | 4 | 5 | 6 |
| 1338 | Ubiquitin thioesterase otulin OS=Homo sapiens GN=OTULIN PE=1 SV=3 | OTUL_HUMAN | 40 kDa | TRUE | 11% | 4 | 4 | 4 |
| 1339 | Prolyl 3-hydroxylase 1 OS=Homo sapiens GN=P3H1 PE=1 SV=2 | P3H1_HUMAN | 83 kDa | TRUE | 6.90% | 5 | 5 | 5 |
| 1340 | Phosphatidylinositol transfer protein beta isoform OS=Homo sapiens GN=PITPNB PE=1 SV=2 | PIPNB_HUMAN | 32 kDa | TRUE | 22% | 5 | 5 | 6 |
| 1341 | Peroxiredoxin-4 OS=Homo sapiens GN=PRDX4 PE=1 SV=1 | PRDX4_HUMAN | 31 kDa | TRUE | 16% | 3 | 5 | 7 |
| 1342 | Ras-related protein Rab-44 OS=Homo sapiens GN=RAB44 PE=1 SV=3 | RAB44_HUMAN | 78 kDa | TRUE | 9.00% | 4 | 4 | 6 |
| 1343 | Rho GTPase-activating protein 17 OS=Homo sapiens GN=ARHGAP17 PE=1 SV=1 | RHG17_HUMAN | 95 kDa | TRUE | 6.50% | 4 | 5 | 6 |
| 1344 | Ribose-5-phosphate isomerase OS=Homo sapiens GN=RPIA PE=1 SV=3 | RPIA_HUMAN | 33 kDa | TRUE | 18% | 5 | 5 | 5 |
| 1345 | RNA 3'-terminal phosphate cyclase OS=Homo sapiens GN=RTCA PE=1 SV=1 | RTCA_HUMAN | 39 kDa |  | 17% | 5 | 6 | 6 |
| 1346 | Protein SEC13 homolog OS=Homo sapiens GN=SEC13 PE=1 SV=3 | SEC13_HUMAN | 36 kDa |  | 11% | 3 | 5 | 6 |
| 1347 | Sorting nexin-18 OS=Homo sapiens GN=SNX18 PE=1 SV=2 | SNX18_HUMAN | 69 kDa |  | 7.30% | 4 | 4 | 4 |
| 1348 | Serine/arginine-rich splicing factor 6 OS=Homo sapiens GN=SRSF6 PE=1 SV=2 | SRSF6_HUMAN | 40 kDa | TRUE | 11% | 4 | 4 | 4 |
| 1349 | Signal transducing adapter molecule 1 OS=Homo sapiens GN=STAM PE=1 SV=3 | STAM1_HUMAN | 59 kDa |  | 14% | 6 | 7 | 7 |
| 1350 | Striatin OS=Homo sapiens GN=STRN PE=1 SV=4 | STRN_HUMAN | 86 kDa | TRUE | 6.50% | 3 | 3 | 3 |
| 1351 | Isoleucine--tRNA ligase, cytoplasmic OS=Homo sapiens GN=IARS PE=1 SV=2 | SYIC_HUMAN | 145 kDa | TRUE | 4.80% | 4 | 4 | 4 |
| 1352 | NEDD8-activating enzyme E1 regulatory subunit OS=Homo sapiens GN=NAE1 PE=1 SV=1 | ULA1_HUMAN | 60 kDa |  | 8.20% | 3 | 3 | 3 |
| 1353 | 5'-3' exoribonuclease 2 OS=Homo sapiens GN=XRN2 PE=1 SV=1 | XRN2_HUMAN | 109 kDa |  | 4.40% | 3 | 3 | 3 |
| 1354 | Alpha-hemoglobin-stabilizing protein OS=Homo sapiens GN=AHSP PE=1 SV=1 | AHSP_HUMAN | 12 kDa |  | 30% | 3 | 3 | 4 |
| 1355 | Hippocalcin-like protein 1 OS=Homo sapiens GN=HPCAL1 PE=1 SV=3 | HPCL1_HUMAN | 22 kDa |  | 21% | 4 | 6 | 6 |
| 1356 | U6 snRNA-associated Sm-like protein LSm8 OS=Homo sapiens GN=LSM8 PE=1 SV=3 | LSM8_HUMAN | 10 kDa |  | 44% | 4 | 5 | 6 |
| 1357 | Mannosyl-oligosaccharide 1,2-alpha-mannosidase IA OS=Homo sapiens GN=MAN1A1 PE=1 SV=3 | MA1A1_HUMAN | 73 kDa |  | 6.40% | 3 | 3 | 3 |
| 1358 | Protein Hook homolog 3 OS=Homo sapiens GN=HOOK3 PE=1 SV=2 | HOOK3_HUMAN | 83 kDa | TRUE | 6.10% | 3 | 4 | 5 |
| 1359 | Synembryn-A OS=Homo sapiens GN=RIC8A PE=1 SV=3 | RIC8A_HUMAN | 60 kDa |  | 5.50% | 2 | 2 | 2 |
| 1360 | Small nuclear ribonucleoprotein-associated proteins B and B' OS=Homo sapiens GN=SNRPB PE=1 SV=2 | RSMB_HUMAN | 25 kDa |  | 13% | 3 | 3 | 4 |
| 1361 | Extracellular matrix protein 2 OS=Homo sapiens GN=ECM2 PE=2 SV=1 | ECM2_HUMAN | 80 kDa | TRUE | 4.30% | 3 | 3 | 3 |
| 1362 | U6 snRNA-associated Sm-like protein LSm6 OS=Homo sapiens GN=LSM6 PE=1 SV=1 | LSM6_HUMAN | 9 kDa |  | 25% | 3 | 4 | 5 |
| 1363 | Asialoglycoprotein receptor 2 OS=Homo sapiens GN=ASGR2 PE=1 SV=2 | ASGR2_HUMAN | 35 kDa |  | 11% | 4 | 4 | 4 |
| 1364 | Proline-serine-threonine phosphatase-interacting protein 2 OS=Homo sapiens GN=PSTPIP2 PE=1 SV=4 | PPIP2_HUMAN | 39 kDa |  | 8.70% | 2 | 2 | 2 |
| 1365 | Tyrosine-protein phosphatase non-receptor type 11 OS=Homo sapiens GN=PTPN11 PE=1 SV=2 | PTN11_HUMAN | 68 kDa | TRUE | 3.40% | 2 | 2 | 2 |
| 1366 | Protein LZIC OS=Homo sapiens GN=LZIC PE=1 SV=1 | LZIC_HUMAN | 21 kDa |  | 21% | 3 | 3 | 3 |
| 1367 | Reelin OS=Homo sapiens GN=RELN PE=1 SV=3 | RELN_HUMAN | 388 kDa |  | 1.50% | 3 | 3 | 3 |
| 1368 | Tetratricopeptide repeat protein 37 OS=Homo sapiens GN=TTC37 PE=1 SV=1 | TTC37_HUMAN | 175 kDa |  | 1.50% | 2 | 2 | 2 |
| 1369 | Protein VAC14 homolog OS=Homo sapiens GN=VAC14 PE=1 SV=1 | VAC14_HUMAN | 88 kDa | TRUE | 4.70% | 3 | 3 | 3 |
| 1370 | Protein argonaute-1 OS=Homo sapiens GN=AGO1 PE=1 SV=3 | AGO1_HUMAN | 97 kDa | TRUE | 9.00% | 3 | 4 | 13 |
| 1371 | Protein RCC2 OS=Homo sapiens GN=RCC2 PE=1 SV=2 | RCC2_HUMAN | 56 kDa |  | 4.20% | 2 | 2 | 2 |
| 1372 | UPF0587 protein C1orf123 OS=Homo sapiens GN=C1orf123 PE=1 SV=1 | CA123_HUMAN | 18 kDa |  | 15% | 3 | 4 | 4 |
| 1373 | Serine/threonine-protein phosphatase 4 regulatory subunit 1 OS=Homo sapiens GN=PPP4R1 PE=1 SV=1 | PP4R1_HUMAN | 107 kDa | TRUE | 2.80% | 2 | 2 | 3 |
| 1374 | Ig delta chain C region OS=Homo sapiens GN=IGHD PE=1 SV=2 | IGHD_HUMAN | 42 kDa | TRUE | 5.70% | 2 | 3 | 3 |
| 1375 | Transformer-2 protein homolog beta OS=Homo sapiens GN=TRA2B PE=1 SV=1 | TRA2B_HUMAN | 34 kDa |  | 13% | 3 | 4 | 5 |
| 1376 | Inosine triphosphate pyrophosphatase OS=Homo sapiens GN=ITPA PE=1 SV=2 | ITPA_HUMAN | 21 kDa | TRUE | 16% | 2 | 2 | 3 |
| 1377 | Ras GTPase-activating protein-binding protein 2 OS=Homo sapiens GN=G3BP2 PE=1 SV=2 | G3BP2_HUMAN | 54 kDa | TRUE | 4.80% | 3 | 3 | 3 |
| 1378 | Exocyst complex component 4 OS=Homo sapiens GN=EXOC4 PE=1 SV=1 | EXOC4_HUMAN | 111 kDa |  | 3.20% | 2 | 2 | 2 |
| 1379 | Ras-related protein Rab-5B OS=Homo sapiens GN=RAB5B PE=1 SV=1 | RAB5B_HUMAN | 24 kDa | TRUE | 27% | 2 | 2 | 8 |
| 1380 | 60S ribosomal protein L32 OS=Homo sapiens GN=RPL32 PE=1 SV=2 | RL32_HUMAN | 16 kDa |  | 21% | 3 | 4 | 4 |
| 1381 | Lactoylglutathione lyase OS=Homo sapiens GN=GLO1 PE=1 SV=4 | LGUL_HUMAN | 21 kDa |  | 14% | 3 | 4 | 4 |
| 1382 | ATP-dependent RNA helicase DDX39A OS=Homo sapiens GN=DDX39A PE=1 SV=2 | DX39A_HUMAN | 49 kDa | TRUE | 23% | 2 | 3 | 22 |
| 1383 | 14 kDa phosphohistidine phosphatase OS=Homo sapiens GN=PHPT1 PE=1 SV=1 | PHP14_HUMAN | 14 kDa |  | 16% | 2 | 3 | 4 |
| 1384 | Importin subunit alpha-7 OS=Homo sapiens GN=KPNA6 PE=1 SV=1 | IMA7_HUMAN | 60 kDa | TRUE | 5.80% | 2 | 3 | 3 |
| 1385 | Brain acid soluble protein 1 OS=Homo sapiens GN=BASP1 PE=1 SV=2 | BASP1_HUMAN | 23 kDa |  | 18% | 2 | 2 | 2 |
| 1386 | 40S ribosomal protein S15a OS=Homo sapiens GN=RPS15A PE=1 SV=2 | RS15A_HUMAN | 15 kDa |  | 18% | 3 | 3 | 4 |
| 1387 | Ubiquitin-like modifier-activating enzyme 7 OS=Homo sapiens GN=UBA7 PE=1 SV=2 | UBA7_HUMAN | 112 kDa |  | 2.10% | 2 | 2 | 2 |
| 1388 | Splicing factor 3B subunit 2 OS=Homo sapiens GN=SF3B2 PE=1 SV=2 | SF3B2_HUMAN | 100 kDa |  | 2.10% | 2 | 2 | 2 |
| 1389 | 40S ribosomal protein S20 OS=Homo sapiens GN=RPS20 PE=1 SV=1 | RS20_HUMAN | 13 kDa |  | 19% | 2 | 5 | 5 |
| 1390 | Replication protein A 32 kDa subunit OS=Homo sapiens GN=RPA2 PE=1 SV=1 | RFA2_HUMAN | 29 kDa |  | 7.40% | 2 | 2 | 3 |
| 1391 | Protein transport protein Sec24D OS=Homo sapiens GN=SEC24D PE=1 SV=2 | SC24D_HUMAN | 113 kDa |  | 2.20% | 2 | 2 | 2 |
| 1392 | Clathrin light chain B OS=Homo sapiens GN=CLTB PE=1 SV=1 | CLCB_HUMAN | 25 kDa |  | 13% | 4 | 4 | 4 |
| 1393 | Nucleoprotein TPR OS=Homo sapiens GN=TPR PE=1 SV=3 | TPR_HUMAN | 267 kDa |  | 1.10% | 2 | 2 | 2 |
| 1394 | Ribulose-phosphate 3-epimerase OS=Homo sapiens GN=RPE PE=1 SV=1 | RPE_HUMAN | 25 kDa |  | 16% | 3 | 4 | 7 |
| 1395 | ADP-ribosylation factor-binding protein GGA1 OS=Homo sapiens GN=GGA1 PE=1 SV=1 | GGA1_HUMAN | 70 kDa | TRUE | 2.70% | 2 | 3 | 3 |
| 1396 | 5'-AMP-activated protein kinase catalytic subunit alpha-1 OS=Homo sapiens GN=PRKAA1 PE=1 SV=4 | AAPK1_HUMAN | 64 kDa |  | 3.80% | 2 | 2 | 2 |
| 1397 | 60S ribosomal protein L29 OS=Homo sapiens GN=RPL29 PE=1 SV=2 | RL29_HUMAN | 18 kDa | TRUE | 14% | 2 | 2 | 2 |
| 1398 | Cathepsin S OS=Homo sapiens GN=CTSS PE=1 SV=3 | CATS_HUMAN | 37 kDa |  | 7.60% | 2 | 3 | 4 |
| 1399 | Ig heavy chain V-III region WEA OS=Homo sapiens PE=1 SV=1 | HV302_HUMAN | 12 kDa | TRUE | 36% | 2 | 3 | 12 |
| 1400 | Inhibin beta C chain OS=Homo sapiens GN=INHBC PE=2 SV=1 | INHBC_HUMAN | 38 kDa |  | 4.80% | 2 | 2 | 2 |
| 1401 | Splicing factor U2AF 65 kDa subunit OS=Homo sapiens GN=U2AF2 PE=1 SV=4 | U2AF2_HUMAN | 54 kDa |  | 3.60% | 2 | 2 | 2 |
| 1402 | HLA class I histocompatibility antigen, Cw-12 alpha chain OS=Homo sapiens GN=HLA-C PE=1 SV=2 | 1C12_HUMAN | 41 kDa | TRUE | 27% | 4 | 5 | 11 |
| 1403 | ATP-binding cassette sub-family E member 1 OS=Homo sapiens GN=ABCE1 PE=1 SV=1 | ABCE1_HUMAN | 67 kDa | TRUE | 6.70% | 4 | 4 | 4 |
| 1404 | DNA-(apurinic or apyrimidinic site) lyase OS=Homo sapiens GN=APEX1 PE=1 SV=2 | APEX1_HUMAN | 36 kDa | TRUE | 16% | 4 | 4 | 4 |
| 1405 | ADP-ribosylation factor-like protein 8B OS=Homo sapiens GN=ARL8B PE=1 SV=1 | ARL8B_HUMAN | 22 kDa | TRUE | 27% | 4 | 5 | 5 |
| 1406 | Ataxin-3 OS=Homo sapiens GN=ATXN3 PE=1 SV=4 | ATX3_HUMAN | 42 kDa |  | 9.10% | 3 | 3 | 5 |
| 1407 | Cadherin-1 OS=Homo sapiens GN=CDH1 PE=1 SV=3 | CADH1_HUMAN | 97 kDa |  | 5.00% | 4 | 5 | 5 |
| 1408 | UPF0568 protein C14orf166 OS=Homo sapiens GN=C14orf166 PE=1 SV=1 | CN166_HUMAN | 28 kDa |  | 23% | 4 | 4 | 4 |
| 1409 | COMM domain-containing protein 9 OS=Homo sapiens GN=COMMD9 PE=1 SV=2 | COMD9_HUMAN | 22 kDa | TRUE | 26% | 4 | 5 | 5 |
| 1410 | Casein kinase II subunit beta OS=Homo sapiens GN=CSNK2B PE=1 SV=1 | CSK2B_HUMAN | 25 kDa |  | 25% | 4 | 4 | 4 |
| 1411 | Dynactin subunit 4 OS=Homo sapiens GN=DCTN4 PE=1 SV=1 | DCTN4_HUMAN | 52 kDa |  | 14% | 4 | 4 | 5 |
| 1412 | Aspartyl aminopeptidase OS=Homo sapiens GN=DNPEP PE=1 SV=1 | DNPEP_HUMAN | 52 kDa |  | 7.60% | 3 | 3 | 3 |
| 1413 | Desmoglein-1 OS=Homo sapiens GN=DSG1 PE=1 SV=2 | DSG1_HUMAN | 114 kDa |  | 3.60% | 3 | 3 | 3 |
| 1414 | Dual specificity protein phosphatase 3 OS=Homo sapiens GN=DUSP3 PE=1 SV=1 | DUS3_HUMAN | 20 kDa |  | 35% | 5 | 5 | 5 |
| 1415 | Epidermal growth factor receptor substrate 15 OS=Homo sapiens GN=EPS15 PE=1 SV=2 | EPS15_HUMAN | 99 kDa |  | 5.20% | 4 | 4 | 4 |
| 1416 | Protein FAM98B OS=Homo sapiens GN=FAM98B PE=1 SV=1 | FA98B_HUMAN | 37 kDa |  | 15% | 4 | 4 | 5 |
| 1417 | GRIP1-associated protein 1 OS=Homo sapiens GN=GRIPAP1 PE=1 SV=1 | GRAP1_HUMAN | 96 kDa |  | 8.40% | 4 | 4 | 4 |
| 1418 | Gasdermin-D OS=Homo sapiens GN=GSDMD PE=1 SV=1 | GSDMD_HUMAN | 53 kDa |  | 10% | 4 | 5 | 5 |
| 1419 | Hemoglobin subunit mu OS=Homo sapiens GN=HBM PE=2 SV=1 | HBM_HUMAN | 16 kDa |  | 45% | 4 | 4 | 5 |
| 1420 | Probable E3 ubiquitin-protein ligase HERC4 OS=Homo sapiens GN=HERC4 PE=1 SV=1 | HERC4_HUMAN | 119 kDa | TRUE | 3.60% | 3 | 3 | 3 |
| 1421 | Immunity-related GTPase family Q protein OS=Homo sapiens GN=IRGQ PE=1 SV=1 | IRGQ_HUMAN | 63 kDa |  | 8.80% | 4 | 4 | 4 |
| 1422 | Dual specificity mitogen-activated protein kinase kinase 4 OS=Homo sapiens GN=MAP2K4 PE=1 SV=1 | MP2K4_HUMAN | 44 kDa |  | 13% | 3 | 3 | 4 |
| 1423 | Nuclear migration protein nudC OS=Homo sapiens GN=NUDC PE=1 SV=1 | NUDC_HUMAN | 38 kDa |  | 16% | 5 | 5 | 5 |
| 1424 | Out at first protein homolog OS=Homo sapiens GN=OAF PE=2 SV=1 | OAF_HUMAN | 31 kDa |  | 16% | 4 | 5 | 6 |
| 1425 | Obg-like ATPase 1 OS=Homo sapiens GN=OLA1 PE=1 SV=2 | OLA1_HUMAN | 45 kDa |  | 11% | 4 | 4 | 4 |
| 1426 | Poly(rC)-binding protein 2 OS=Homo sapiens GN=PCBP2 PE=1 SV=1 | PCBP2_HUMAN | 39 kDa | TRUE | 28% | 4 | 5 | 16 |
| 1427 | Peptidyl-prolyl cis-trans isomerase NIMA-interacting 1 OS=Homo sapiens GN=PIN1 PE=1 SV=1 | PIN1_HUMAN | 18 kDa |  | 34% | 5 | 5 | 5 |
| 1428 | Serine/threonine-protein phosphatase 6 regulatory subunit 1 OS=Homo sapiens GN=PPP6R1 PE=1 SV=5 | PP6R1_HUMAN | 97 kDa |  | 9.60% | 4 | 5 | 5 |
| 1429 | Polymerase I and transcript release factor OS=Homo sapiens GN=PTRF PE=1 SV=1 | PTRF_HUMAN | 43 kDa |  | 14% | 4 | 5 | 5 |
| 1430 | Ras-related protein Rab-3D OS=Homo sapiens GN=RAB3D PE=1 SV=1 | RAB3D_HUMAN | 24 kDa | TRUE | 21% | 3 | 4 | 7 |
| 1431 | Ras-related protein Rab-6A OS=Homo sapiens GN=RAB6A PE=1 SV=3 | RAB6A_HUMAN | 24 kDa | TRUE | 33% | 5 | 6 | 8 |
| 1432 | Ran GTPase-activating protein 1 OS=Homo sapiens GN=RANGAP1 PE=1 SV=1 | RAGP1_HUMAN | 64 kDa | TRUE | 8.50% | 4 | 4 | 4 |
| 1433 | Prosaposin OS=Homo sapiens GN=PSAP PE=1 SV=2 | SAP_HUMAN | 58 kDa |  | 12% | 5 | 5 | 6 |
| 1434 | Ribosome maturation protein SBDS OS=Homo sapiens GN=SBDS PE=1 SV=4 | SBDS_HUMAN | 29 kDa | TRUE | 14% | 4 | 4 | 4 |
| 1435 | Small glutamine-rich tetratricopeptide repeat-containing protein beta OS=Homo sapiens GN=SGTB PE=1 SV=1 | SGTB_HUMAN | 33 kDa |  | 12% | 3 | 4 | 4 |
| 1436 | Cohesin subunit SA-2 OS=Homo sapiens GN=STAG2 PE=1 SV=3 | STAG2_HUMAN | 141 kDa | TRUE | 3.20% | 4 | 4 | 4 |
| 1437 | Phenylalanine--tRNA ligase alpha subunit OS=Homo sapiens GN=FARSA PE=1 SV=3 | SYFA_HUMAN | 58 kDa | TRUE | 8.50% | 3 | 3 | 3 |
| 1438 | 3-mercaptopyruvate sulfurtransferase OS=Homo sapiens GN=MPST PE=1 SV=3 | THTM_HUMAN | 33 kDa |  | 14% | 4 | 4 | 4 |
| 1439 | Tumor necrosis factor alpha-induced protein 2 OS=Homo sapiens GN=TNFAIP2 PE=2 SV=2 | TNAP2_HUMAN | 73 kDa |  | 9.80% | 5 | 6 | 6 |
| 1440 | Tetratricopeptide repeat protein 38 OS=Homo sapiens GN=TTC38 PE=1 SV=1 | TTC38_HUMAN | 53 kDa |  | 10% | 4 | 4 | 4 |
| 1441 | Ubiquitin-conjugating enzyme E2 Z OS=Homo sapiens GN=UBE2Z PE=1 SV=2 | UBE2Z_HUMAN | 38 kDa |  | 15% | 4 | 4 | 5 |
| 1442 | Collectin-10 OS=Homo sapiens GN=COLEC10 PE=2 SV=2 | COL10_HUMAN | 31 kDa |  | 12% | 3 | 3 | 3 |
| 1443 | Fibrillin-1 OS=Homo sapiens GN=FBN1 PE=1 SV=3 | FBN1_HUMAN | 312 kDa |  | 1.70% | 5 | 5 | 5 |
| 1444 | Kynureninase OS=Homo sapiens GN=KYNU PE=1 SV=1 | KYNU_HUMAN | 52 kDa |  | 8.60% | 3 | 3 | 3 |
| 1445 | Mini-chromosome maintenance complex-binding protein OS=Homo sapiens GN=MCMBP PE=1 SV=2 | MCMBP_HUMAN | 73 kDa |  | 4.70% | 3 | 3 | 3 |
| 1446 | Pyridoxal-dependent decarboxylase domain-containing protein 1 OS=Homo sapiens GN=PDXDC1 PE=1 SV=2 | PDXD1_HUMAN | 87 kDa |  | 4.70% | 3 | 4 | 4 |
| 1447 | U2 small nuclear ribonucleoprotein A' OS=Homo sapiens GN=SNRPA1 PE=1 SV=2 | RU2A_HUMAN | 28 kDa |  | 15% | 3 | 3 | 3 |
| 1448 | Heterogeneous nuclear ribonucleoprotein H2 OS=Homo sapiens GN=HNRNPH2 PE=1 SV=1 | HNRH2_HUMAN | 49 kDa | TRUE | 21% | 3 | 3 | 12 |
| 1449 | Nuclear factor NF-kappa-B p100 subunit OS=Homo sapiens GN=NFKB2 PE=1 SV=4 | NFKB2_HUMAN | 97 kDa |  | 4.70% | 3 | 3 | 3 |
| 1450 | 60S ribosomal protein L31 OS=Homo sapiens GN=RPL31 PE=1 SV=1 | RL31_HUMAN | 14 kDa |  | 26% | 3 | 3 | 4 |
| 1451 | Vacuolar protein sorting-associated protein 16 homolog OS=Homo sapiens GN=VPS16 PE=1 SV=2 | VPS16_HUMAN | 95 kDa |  | 5.00% | 3 | 3 | 3 |
| 1452 | Endosialin OS=Homo sapiens GN=CD248 PE=1 SV=1 | CD248_HUMAN | 81 kDa |  | 2.90% | 2 | 3 | 4 |
| 1453 | Histone H1.2 OS=Homo sapiens GN=HIST1H1C PE=1 SV=2 | H12_HUMAN | 21 kDa | TRUE | 42% | 4 | 4 | 44 |
| 1454 | LIM and cysteine-rich domains protein 1 OS=Homo sapiens GN=LMCD1 PE=1 SV=1 | LMCD1_HUMAN | 41 kDa |  | 9.00% | 2 | 2 | 2 |
| 1455 | Collagen alpha-1(XV) chain OS=Homo sapiens GN=COL15A1 PE=1 SV=2 | COFA1_HUMAN | 142 kDa |  | 2.40% | 3 | 3 | 3 |
| 1456 | Heterochromatin protein 1-binding protein 3 OS=Homo sapiens GN=HP1BP3 PE=1 SV=1 | HP1B3_HUMAN | 61 kDa | TRUE | 5.40% | 3 | 4 | 4 |
| 1457 | Leucine-rich repeat flightless-interacting protein 2 OS=Homo sapiens GN=LRRFIP2 PE=1 SV=1 | LRRF2_HUMAN | 82 kDa | TRUE | 3.50% | 3 | 3 | 3 |
| 1458 | Bifunctional protein NCOAT OS=Homo sapiens GN=MGEA5 PE=1 SV=2 | NCOAT_HUMAN | ? |  | 0.00% | 3 | 3 | 3 |
| 1459 | 40S ribosomal protein S26 OS=Homo sapiens GN=RPS26 PE=1 SV=3 | RS26_HUMAN | 13 kDa |  | 21% | 2 | 3 | 3 |
| 1460 | Structural maintenance of chromosomes flexible hinge domain-containing protein 1 OS=Homo sapiens GN=SMCHD1 PE=1 SV=2 | SMHD1_HUMAN | 226 kDa | TRUE | 2.10% | 3 | 3 | 3 |
| 1461 | Actin-related protein 10 OS=Homo sapiens GN=ACTR10 PE=1 SV=1 | ARP10_HUMAN | 46 kDa |  | 9.60% | 3 | 4 | 4 |
| 1462 | Eukaryotic translation initiation factor 4H OS=Homo sapiens GN=EIF4H PE=1 SV=5 | IF4H_HUMAN | 27 kDa | TRUE | 17% | 2 | 3 | 3 |
| 1463 | Lysyl oxidase homolog 3 OS=Homo sapiens GN=LOXL3 PE=2 SV=1 | LOXL3_HUMAN | 83 kDa | TRUE | 3.30% | 2 | 2 | 2 |
| 1464 | Myotrophin OS=Homo sapiens GN=MTPN PE=1 SV=2 | MTPN_HUMAN | 13 kDa |  | 28% | 3 | 5 | 5 |
| 1465 | Signal recognition particle subunit SRP72 OS=Homo sapiens GN=SRP72 PE=1 SV=3 | SRP72_HUMAN | 75 kDa | TRUE | 4.00% | 2 | 2 | 2 |
| 1466 | Interferon-induced protein with tetratricopeptide repeats 2 OS=Homo sapiens GN=IFIT2 PE=1 SV=1 | IFIT2_HUMAN | 55 kDa |  | 6.40% | 2 | 3 | 4 |
| 1467 | Cell division cycle and apoptosis regulator protein 1 OS=Homo sapiens GN=CCAR1 PE=1 SV=2 | CCAR1_HUMAN | 133 kDa |  | 2.90% | 3 | 3 | 3 |
| 1468 | Fructose-bisphosphate aldolase C OS=Homo sapiens GN=ALDOC PE=1 SV=2 | ALDOC_HUMAN | 39 kDa | TRUE | 13% | 3 | 4 | 12 |
| 1469 | SWI/SNF complex subunit SMARCC2 OS=Homo sapiens GN=SMARCC2 PE=1 SV=1 | SMRC2_HUMAN | 133 kDa |  | 3.00% | 3 | 3 | 3 |
| 1470 | Structural maintenance of chromosomes protein 2 OS=Homo sapiens GN=SMC2 PE=1 SV=2 | SMC2_HUMAN | 136 kDa |  | 2.60% | 2 | 2 | 2 |
| 1471 | Sushi, von Willebrand factor type A, EGF and pentraxin domain-containing protein 1 OS=Homo sapiens GN=SVEP1 PE=1 SV=3 | SVEP1_HUMAN | 390 kDa | TRUE | 0.87% | 3 | 3 | 3 |
| 1472 | Thioredoxin-dependent peroxide reductase, mitochondrial OS=Homo sapiens GN=PRDX3 PE=1 SV=3 | PRDX3_HUMAN | 28 kDa |  | 9.80% | 2 | 3 | 3 |
| 1473 | 60S ribosomal protein L27a OS=Homo sapiens GN=RPL27A PE=1 SV=2 | RL27A_HUMAN | 17 kDa |  | 19% | 3 | 3 | 3 |
| 1474 | U6 snRNA-associated Sm-like protein LSm7 OS=Homo sapiens GN=LSM7 PE=1 SV=1 | LSM7_HUMAN | 12 kDa |  | 32% | 2 | 3 | 3 |
| 1475 | Sorting nexin-9 OS=Homo sapiens GN=SNX9 PE=1 SV=1 | SNX9_HUMAN | 67 kDa |  | 4.40% | 3 | 3 | 4 |
| 1476 | Anthrax toxin receptor 1 OS=Homo sapiens GN=ANTXR1 PE=1 SV=2 | ANTR1_HUMAN | 63 kDa | TRUE | 4.40% | 2 | 2 | 3 |
| 1477 | Protein canopy homolog 2 OS=Homo sapiens GN=CNPY2 PE=1 SV=1 | CNPY2_HUMAN | 21 kDa |  | 15% | 2 | 2 | 4 |
| 1478 | Ig heavy chain V-I region 5 (Fragment) OS=Homo sapiens GN=IGKV1-5 PE=4 SV=2 | KV110_HUMAN | 13 kDa | TRUE | 15% | 2 | 3 | 5 |
| 1479 | Sister chromatid cohesion protein PDS5 homolog B OS=Homo sapiens GN=PDS5B PE=1 SV=1 | PDS5B_HUMAN | 165 kDa | TRUE | 2.30% | 3 | 4 | 4 |
| 1480 | Catechol O-methyltransferase OS=Homo sapiens GN=COMT PE=1 SV=2 | COMT_HUMAN | 30 kDa |  | 8.50% | 3 | 5 | 5 |
| 1481 | Tropomyosin alpha-1 chain OS=Homo sapiens GN=TPM1 PE=1 SV=2 | TPM1_HUMAN | 33 kDa | TRUE | 32% | 3 | 4 | 30 |
| 1482 | Catenin alpha-1 OS=Homo sapiens GN=CTNNA1 PE=1 SV=1 | CTNA1_HUMAN | 100 kDa |  | 2.40% | 2 | 2 | 2 |
| 1483 | Eosinophil peroxidase OS=Homo sapiens GN=EPX PE=1 SV=2 | PERE_HUMAN | 81 kDa | TRUE | 7.70% | 2 | 2 | 53 |
| 1484 | Protein phosphatase methylesterase 1 OS=Homo sapiens GN=PPME1 PE=1 SV=3 | PPME1_HUMAN | 42 kDa |  | 5.20% | 2 | 2 | 2 |
| 1485 | RNA-binding protein 12 OS=Homo sapiens GN=RBM12 PE=1 SV=1 | RBM12_HUMAN | 97 kDa |  | 3.60% | 3 | 3 | 3 |
| 1486 | V-type proton ATPase subunit G 1 OS=Homo sapiens GN=ATP6V1G1 PE=1 SV=3 | VATG1_HUMAN | 14 kDa |  | 17% | 2 | 3 | 4 |
| 1487 | RWD domain-containing protein 4 OS=Homo sapiens GN=RWDD4 PE=1 SV=3 | RWDD4_HUMAN | 21 kDa |  | 11% | 2 | 2 | 2 |
| 1488 | Macrophage migration inhibitory factor OS=Homo sapiens GN=MIF PE=1 SV=4 | MIF_HUMAN | 12 kDa |  | 23% | 3 | 3 | 3 |
| 1489 | Interferon regulatory factor 3 OS=Homo sapiens GN=IRF3 PE=1 SV=1 | IRF3_HUMAN | 47 kDa |  | 6.30% | 2 | 3 | 3 |
| 1490 | Alpha-actinin-3 OS=Homo sapiens GN=ACTN3 PE=1 SV=2 | ACTN3_HUMAN | 103 kDa | TRUE | 14% | 2 | 3 | 44 |
| 1491 | Matrin-3 OS=Homo sapiens GN=MATR3 PE=1 SV=2 | MATR3_HUMAN | 95 kDa |  | 2.60% | 2 | 2 | 2 |
| 1492 | Beta-2-microglobulin OS=Homo sapiens GN=B2M PE=1 SV=1 | B2MG_HUMAN | 14 kDa |  | 17% | 2 | 4 | 4 |
| 1493 | Cdc42-interacting protein 4 OS=Homo sapiens GN=TRIP10 PE=1 SV=3 | CIP4_HUMAN | 68 kDa | TRUE | 5.00% | 2 | 2 | 2 |
| 1494 | Superoxide dismutase [Cu-Zn] OS=Homo sapiens GN=SOD1 PE=1 SV=2 | SODC_HUMAN | 16 kDa |  | 14% | 2 | 2 | 2 |
| 1495 | Ig kappa chain V-II region FR OS=Homo sapiens PE=1 SV=1 | KV202_HUMAN | 13 kDa |  | 18% | 2 | 3 | 5 |
| 1496 | Dynein light chain 1, cytoplasmic OS=Homo sapiens GN=DYNLL1 PE=1 SV=1 | DYL1_HUMAN | 10 kDa |  | 33% | 2 | 4 | 4 |
| 1497 | Ig lambda chain V-VI region SUT OS=Homo sapiens PE=1 SV=1 | LV603_HUMAN | 12 kDa |  | 21% | 2 | 2 | 4 |
| 1498 | Protein S100-A10 OS=Homo sapiens GN=S100A10 PE=1 SV=2 | S10AA_HUMAN | 11 kDa | TRUE | 18% | 2 | 2 | 3 |
| 1499 | Alanyl-tRNA editing protein Aarsd1 OS=Homo sapiens GN=AARSD1 PE=1 SV=2 | AASD1_HUMAN | 45 kDa |  | 18% | 5 | 5 | 5 |
| 1500 | Serine/threonine-protein phosphatase 6 regulatory ankyrin repeat subunit B OS=Homo sapiens GN=ANKRD44 PE=1 SV=3 | ANR44_HUMAN | 108 kDa |  | 4.20% | 4 | 4 | 5 |
| 1501 | Retroviral-like aspartic protease 1 OS=Homo sapiens GN=ASPRV1 PE=1 SV=1 | APRV1_HUMAN | 37 kDa | TRUE | 24% | 3 | 3 | 5 |
| 1502 | Large proline-rich protein BAG6 OS=Homo sapiens GN=BAG6 PE=1 SV=2 | BAG6_HUMAN | 119 kDa |  | 4.00% | 3 | 4 | 4 |
| 1503 | Biotinidase OS=Homo sapiens GN=BTD PE=1 SV=2 | BTD_HUMAN | 61 kDa |  | 11% | 4 | 4 | 5 |
| 1504 | Corticosteroid-binding globulin OS=Homo sapiens GN=SERPINA6 PE=1 SV=1 | CBG_HUMAN | 45 kDa |  | 11% | 4 | 4 | 4 |
| 1505 | Desmoglein-2 OS=Homo sapiens GN=DSG2 PE=1 SV=2 | DSG2_HUMAN | 122 kDa |  | 5.10% | 5 | 5 | 5 |
| 1506 | Fructose-1,6-bisphosphatase 1 OS=Homo sapiens GN=FBP1 PE=1 SV=5 | F16P1_HUMAN | 37 kDa |  | 12% | 3 | 4 | 4 |
| 1507 | Golgi-associated plant pathogenesis-related protein 1 OS=Homo sapiens GN=GLIPR2 PE=1 SV=3 | GAPR1_HUMAN | 17 kDa |  | 28% | 3 | 3 | 4 |
| 1508 | N-acetylglucosamine-1-phosphotransferase subunit gamma OS=Homo sapiens GN=GNPTG PE=1 SV=1 | GNPTG_HUMAN | 34 kDa |  | 15% | 3 | 4 | 4 |
| 1509 | Histone acetyltransferase type B catalytic subunit OS=Homo sapiens GN=HAT1 PE=1 SV=1 | HAT1_HUMAN | 50 kDa |  | 13% | 4 | 4 | 4 |
| 1510 | Histone deacetylase 1 OS=Homo sapiens GN=HDAC1 PE=1 SV=1 | HDAC1_HUMAN | 55 kDa |  | 12% | 4 | 4 | 4 |
| 1511 | Eukaryotic initiation factor 4A-II OS=Homo sapiens GN=EIF4A2 PE=1 SV=2 | IF4A2_HUMAN | 46 kDa | TRUE | 42% | 3 | 4 | 31 |
| 1512 | DNA replication licensing factor MCM4 OS=Homo sapiens GN=MCM4 PE=1 SV=5 | MCM4_HUMAN | 97 kDa |  | 3.50% | 3 | 3 | 3 |
| 1513 | Nuclear cap-binding protein subunit 1 OS=Homo sapiens GN=NCBP1 PE=1 SV=1 | NCBP1_HUMAN | 92 kDa | TRUE | 5.40% | 3 | 3 | 3 |
| 1514 | Protein NOXP20 OS=Homo sapiens GN=FAM114A1 PE=1 SV=2 | NXP20_HUMAN | 61 kDa |  | 9.80% | 4 | 4 | 4 |
| 1515 | Lysosomal Pro-X carboxypeptidase OS=Homo sapiens GN=PRCP PE=1 SV=1 | PCP_HUMAN | 56 kDa |  | 9.90% | 3 | 3 | 3 |
| 1516 | Programmed cell death protein 10 OS=Homo sapiens GN=PDCD10 PE=1 SV=1 | PDC10_HUMAN | 25 kDa |  | 20% | 4 | 4 | 4 |
| 1517 | Serine/threonine-protein kinase N1 OS=Homo sapiens GN=PKN1 PE=1 SV=2 | PKN1_HUMAN | 104 kDa | TRUE | 5.30% | 4 | 4 | 4 |
| 1518 | Pyridoxine-5'-phosphate oxidase OS=Homo sapiens GN=PNPO PE=1 SV=1 | PNPO_HUMAN | 30 kDa |  | 18% | 3 | 3 | 3 |
| 1519 | Double-strand-break repair protein rad21 homolog OS=Homo sapiens GN=RAD21 PE=1 SV=2 | RAD21_HUMAN | 72 kDa | TRUE | 7.10% | 3 | 3 | 3 |
| 1520 | SAM domain-containing protein SAMSN-1 OS=Homo sapiens GN=SAMSN1 PE=1 SV=1 | SAMN1_HUMAN | 42 kDa |  | 12% | 4 | 4 | 4 |
| 1521 | Serpin B9 OS=Homo sapiens GN=SERPINB9 PE=1 SV=1 | SPB9_HUMAN | 42 kDa | TRUE | 18% | 4 | 4 | 6 |
| 1522 | Methionine--tRNA ligase, cytoplasmic OS=Homo sapiens GN=MARS PE=1 SV=2 | SYMC_HUMAN | 101 kDa |  | 5.10% | 4 | 4 | 4 |
| 1523 | Protein unc-45 homolog A OS=Homo sapiens GN=UNC45A PE=1 SV=1 | UN45A_HUMAN | 103 kDa |  | 8.50% | 5 | 5 | 5 |
| 1524 | Uridine phosphorylase 1 OS=Homo sapiens GN=UPP1 PE=1 SV=1 | UPP1_HUMAN | 34 kDa |  | 13% | 4 | 4 | 4 |
| 1525 | Vacuolar protein sorting-associated protein 26B OS=Homo sapiens GN=VPS26B PE=1 SV=2 | VP26B_HUMAN | 39 kDa | TRUE | 17% | 4 | 4 | 5 |
| 1526 | Persulfide dioxygenase ETHE1, mitochondrial OS=Homo sapiens GN=ETHE1 PE=1 SV=2 | ETHE1_HUMAN | 28 kDa |  | 16% | 3 | 4 | 5 |
| 1527 | Probable aminopeptidase NPEPL1 OS=Homo sapiens GN=NPEPL1 PE=1 SV=3 | PEPL1_HUMAN | 56 kDa |  | 11% | 4 | 4 | 4 |
| 1528 | Histone-binding protein RBBP7 OS=Homo sapiens GN=RBBP7 PE=1 SV=1 | RBBP7_HUMAN | 48 kDa | TRUE | 29% | 4 | 4 | 12 |
| 1529 | Resistin OS=Homo sapiens GN=RETN PE=1 SV=1 | RETN_HUMAN | 11 kDa |  | 29% | 3 | 4 | 5 |
| 1530 | Splicing factor 3B subunit 6 OS=Homo sapiens GN=SF3B6 PE=1 SV=1 | SF3B6_HUMAN | 15 kDa | TRUE | 28% | 3 | 3 | 4 |
| 1531 | Small nuclear ribonucleoprotein Sm D3 OS=Homo sapiens GN=SNRPD3 PE=1 SV=1 | SMD3_HUMAN | 14 kDa |  | 31% | 3 | 3 | 3 |
| 1532 | Secreted phosphoprotein 24 OS=Homo sapiens GN=SPP2 PE=1 SV=1 | SPP24_HUMAN | 24 kDa |  | 16% | 3 | 3 | 3 |
| 1533 | Prostaglandin E synthase 3 OS=Homo sapiens GN=PTGES3 PE=1 SV=1 | TEBP_HUMAN | 19 kDa |  | 29% | 4 | 4 | 4 |
| 1534 | Tsukushin OS=Homo sapiens GN=TSKU PE=2 SV=3 | TSK_HUMAN | 38 kDa | TRUE | 11% | 3 | 4 | 4 |
| 1535 | BRISC and BRCA1-A complex member 1 OS=Homo sapiens GN=BABAM1 PE=1 SV=1 | BABA1_HUMAN | 37 kDa |  | 14% | 3 | 3 | 4 |
| 1536 | COMM domain-containing protein 3 OS=Homo sapiens GN=COMMD3 PE=1 SV=1 | COMD3_HUMAN | 22 kDa |  | 23% | 3 | 4 | 4 |
| 1537 | Band 4.1-like protein 3 OS=Homo sapiens GN=EPB41L3 PE=1 SV=2 | E41L3_HUMAN | 121 kDa | TRUE | 4.60% | 3 | 3 | 4 |
| 1538 | Pyridoxal phosphate phosphatase OS=Homo sapiens GN=PDXP PE=1 SV=2 | PLPP_HUMAN | 32 kDa |  | 17% | 4 | 4 | 4 |
| 1539 | Stabilin-1 OS=Homo sapiens GN=STAB1 PE=1 SV=3 | STAB1_HUMAN | 275 kDa |  | 1.80% | 3 | 3 | 3 |
| 1540 | Echinoderm microtubule-associated protein-like 2 OS=Homo sapiens GN=EML2 PE=1 SV=1 | EMAL2_HUMAN | 71 kDa |  | 4.00% | 2 | 2 | 2 |
| 1541 | Integrin beta-2 OS=Homo sapiens GN=ITGB2 PE=1 SV=2 | ITB2_HUMAN | 85 kDa |  | 4.40% | 3 | 3 | 3 |
| 1542 | Microtubule-associated protein RP/EB family member 2 OS=Homo sapiens GN=MAPRE2 PE=1 SV=1 | MARE2_HUMAN | 37 kDa | TRUE | 9.50% | 2 | 2 | 2 |
| 1543 | Poly(U)-binding-splicing factor PUF60 OS=Homo sapiens GN=PUF60 PE=1 SV=1 | PUF60_HUMAN | 60 kDa |  | 3.00% | 2 | 2 | 2 |
| 1544 | Receptor expression-enhancing protein 5 OS=Homo sapiens GN=REEP5 PE=1 SV=3 | REEP5_HUMAN | 21 kDa |  | 10% | 3 | 5 | 5 |
| 1545 | Nuclear factor NF-kappa-B p105 subunit OS=Homo sapiens GN=NFKB1 PE=1 SV=2 | NFKB1_HUMAN | 105 kDa |  | 4.90% | 4 | 5 | 5 |
| 1546 | Nuclear distribution protein nudE homolog 1 OS=Homo sapiens GN=NDE1 PE=1 SV=2 | NDE1_HUMAN | 39 kDa |  | 6.10% | 2 | 2 | 2 |
| 1547 | Eukaryotic translation initiation factor 1 OS=Homo sapiens GN=EIF1 PE=1 SV=1 | EIF1_HUMAN | 13 kDa | TRUE | 27% | 3 | 3 | 5 |
| 1548 | UPF0687 protein C20orf27 OS=Homo sapiens GN=C20orf27 PE=1 SV=3 | CT027_HUMAN | 19 kDa |  | 24% | 3 | 4 | 4 |
| 1549 | Vacuolar protein sorting-associated protein 4A OS=Homo sapiens GN=VPS4A PE=1 SV=1 | VPS4A_HUMAN | 49 kDa | TRUE | 16% | 4 | 4 | 8 |
| 1550 | Oxysterol-binding protein-related protein 8 OS=Homo sapiens GN=OSBPL8 PE=1 SV=3 | OSBL8_HUMAN | 101 kDa |  | 3.80% | 3 | 3 | 3 |
| 1551 | C-terminal-binding protein 1 OS=Homo sapiens GN=CTBP1 PE=1 SV=2 | CTBP1_HUMAN | 48 kDa | TRUE | 6.40% | 3 | 4 | 4 |
| 1552 | Annexin A7 OS=Homo sapiens GN=ANXA7 PE=1 SV=3 | ANXA7_HUMAN | 53 kDa |  | 5.10% | 2 | 2 | 2 |
| 1553 | Inosine-5'-monophosphate dehydrogenase 1 OS=Homo sapiens GN=IMPDH1 PE=1 SV=2 | IMDH1_HUMAN | 55 kDa | TRUE | 5.60% | 2 | 3 | 3 |
| 1554 | Developmentally-regulated GTP-binding protein 1 OS=Homo sapiens GN=DRG1 PE=1 SV=1 | DRG1_HUMAN | 41 kDa |  | 8.20% | 3 | 3 | 3 |
| 1555 | Protocadherin-18 OS=Homo sapiens GN=PCDH18 PE=2 SV=3 | PCD18_HUMAN | 126 kDa |  | 2.40% | 3 | 3 | 3 |
| 1556 | Gamma-parvin OS=Homo sapiens GN=PARVG PE=1 SV=1 | PARVG_HUMAN | 37 kDa |  | 7.90% | 2 | 3 | 3 |
| 1557 | E3 ubiquitin-protein ligase NEDD4 OS=Homo sapiens GN=NEDD4 PE=1 SV=4 | NEDD4_HUMAN | 149 kDa | TRUE | 2.50% | 2 | 2 | 2 |
| 1558 | Haloacid dehalogenase-like hydrolase domain-containing protein 2 OS=Homo sapiens GN=HDHD2 PE=1 SV=1 | HDHD2_HUMAN | 29 kDa |  | 12% | 2 | 2 | 3 |
| 1559 | Replication protein A 14 kDa subunit OS=Homo sapiens GN=RPA3 PE=1 SV=1 | RFA3_HUMAN | 14 kDa |  | 27% | 2 | 3 | 3 |
| 1560 | DNA fragmentation factor subunit alpha OS=Homo sapiens GN=DFFA PE=1 SV=1 | DFFA_HUMAN | 37 kDa |  | 7.90% | 2 | 2 | 2 |
| 1561 | Dual specificity mitogen-activated protein kinase kinase 2 OS=Homo sapiens GN=MAP2K2 PE=1 SV=1 | MP2K2_HUMAN | 44 kDa | TRUE | 16% | 2 | 2 | 7 |
| 1562 | Serine/threonine-protein kinase MST4 OS=Homo sapiens GN=MST4 PE=1 SV=2 | MST4_HUMAN | ? | TRUE | 0.00% | 2 | 3 | 7 |
| 1563 | Activating signal cointegrator 1 complex subunit 3 OS=Homo sapiens GN=ASCC3 PE=1 SV=3 | ASCC3_HUMAN | 251 kDa | TRUE | 1.50% | 2 | 2 | 2 |
| 1564 | Ras-related protein Rap-1b OS=Homo sapiens GN=RAP1B PE=1 SV=1 | RAP1B_HUMAN | 21 kDa | TRUE | 38% | 2 | 3 | 13 |
| 1565 | U1 small nuclear ribonucleoprotein A OS=Homo sapiens GN=SNRPA PE=1 SV=3 | SNRPA_HUMAN | 31 kDa | TRUE | 18% | 3 | 3 | 7 |
| 1566 | Cystatin-C OS=Homo sapiens GN=CST3 PE=1 SV=1 | CYTC_HUMAN | 16 kDa |  | 18% | 2 | 3 | 4 |
| 1567 | Secreted frizzled-related protein 2 OS=Homo sapiens GN=SFRP2 PE=1 SV=2 | SFRP2_HUMAN | 33 kDa |  | 8.80% | 2 | 2 | 2 |
| 1568 | Gasdermin-A OS=Homo sapiens GN=GSDMA PE=1 SV=4 | GSDMA_HUMAN | 49 kDa |  | 6.10% | 2 | 2 | 2 |
| 1569 | Ras-related protein Rab-35 OS=Homo sapiens GN=RAB35 PE=1 SV=1 | RAB35_HUMAN | 23 kDa | TRUE | 28% | 3 | 3 | 8 |
| 1570 | Band 4.1-like protein 2 OS=Homo sapiens GN=EPB41L2 PE=1 SV=1 | E41L2_HUMAN | 113 kDa | TRUE | 3.60% | 2 | 2 | 2 |
| 1571 | Keratin, type II cytoskeletal 6A OS=Homo sapiens GN=KRT6A PE=1 SV=3 | K2C6A_HUMAN | 60 kDa | TRUE | 35% | 2 | 3 | 56 |
| 1572 | A disintegrin and metalloproteinase with thrombospondin motifs 2 OS=Homo sapiens GN=ADAMTS2 PE=2 SV=2 | ATS2_HUMAN | 135 kDa |  | 2.10% | 2 | 3 | 4 |
| 1573 | Dynactin subunit 3 OS=Homo sapiens GN=DCTN3 PE=1 SV=1 | DCTN3_HUMAN | 21 kDa |  | 10% | 2 | 2 | 2 |
| 1574 | GTP-binding protein 1 OS=Homo sapiens GN=GTPBP1 PE=1 SV=3 | GTPB1_HUMAN | 72 kDa |  | 3.60% | 2 | 3 | 3 |
| 1575 | Gamma-enolase OS=Homo sapiens GN=ENO2 PE=1 SV=3 | ENOG_HUMAN | 47 kDa | TRUE | 15% | 3 | 3 | 5 |
| 1576 | Rab3 GTPase-activating protein catalytic subunit OS=Homo sapiens GN=RAB3GAP1 PE=1 SV=3 | RB3GP_HUMAN | 111 kDa | TRUE | 2.00% | 2 | 2 | 2 |
| 1577 | Tumor susceptibility gene 101 protein OS=Homo sapiens GN=TSG101 PE=1 SV=2 | TS101_HUMAN | 44 kDa |  | 4.90% | 2 | 2 | 2 |
| 1578 | Vacuolar protein sorting-associated protein 33A OS=Homo sapiens GN=VPS33A PE=1 SV=1 | VP33A_HUMAN | 68 kDa |  | 3.20% | 2 | 2 | 2 |
| 1579 | Tenascin-X OS=Homo sapiens GN=TNXB PE=1 SV=4 | TENX_HUMAN | 458 kDa |  | 0.75% | 3 | 3 | 3 |
| 1580 | Trifunctional purine biosynthetic protein adenosine-3 OS=Homo sapiens GN=GART PE=1 SV=1 | PUR2_HUMAN | 108 kDa |  | 1.90% | 2 | 2 | 2 |
| 1581 | Core-binding factor subunit beta OS=Homo sapiens GN=CBFB PE=1 SV=2 | PEBB_HUMAN | 22 kDa |  | 16% | 3 | 4 | 4 |
| 1582 | Docking protein 3 OS=Homo sapiens GN=DOK3 PE=1 SV=2 | DOK3_HUMAN | 53 kDa |  | 4.20% | 2 | 2 | 2 |
| 1583 | N-acylglucosamine 2-epimerase OS=Homo sapiens GN=RENBP PE=1 SV=2 | RENBP_HUMAN | 49 kDa |  | 4.40% | 2 | 2 | 2 |
| 1584 | Complement factor H-related protein 2 OS=Homo sapiens GN=CFHR2 PE=1 SV=1 | FHR2_HUMAN | 31 kDa | TRUE | 35% | 3 | 3 | 25 |
| 1585 | Angiogenin OS=Homo sapiens GN=ANG PE=1 SV=1 | ANGI_HUMAN | 17 kDa |  | 17% | 2 | 3 | 3 |
| 1586 | Eukaryotic translation initiation factor 4 gamma 2 OS=Homo sapiens GN=EIF4G2 PE=1 SV=1 | IF4G2_HUMAN | 102 kDa | TRUE | 2.20% | 2 | 2 | 3 |
| 1587 | Ig lambda-7 chain C region OS=Homo sapiens GN=IGLC7 PE=4 SV=2 | LAC7_HUMAN | 11 kDa | TRUE | 56% | 2 | 2 | 129 |
| 1588 | Fibroblast growth factor 17 OS=Homo sapiens GN=FGF17 PE=1 SV=1 | FGF17_HUMAN | 25 kDa |  | 8.30% | 2 | 3 | 3 |
| 1589 | Ubiquitin-conjugating enzyme E2 L3 OS=Homo sapiens GN=UBE2L3 PE=1 SV=1 | UB2L3_HUMAN | 18 kDa | TRUE | 24% | 2 | 3 | 4 |
| 1590 | Thiosulfate sulfurtransferase/rhodanese-like domain-containing protein 1 OS=Homo sapiens GN=TSTD1 PE=1 SV=3 | TSTD1_HUMAN | 13 kDa | TRUE | 15% | 2 | 2 | 3 |
| 1591 | Elongation factor Tu GTP-binding domain-containing protein 1 OS=Homo sapiens GN=EFTUD1 PE=1 SV=2 | ETUD1_HUMAN | 125 kDa |  | 2.80% | 2 | 2 | 3 |
| 1592 | UDP-N-acetylhexosamine pyrophosphorylase-like protein 1 OS=Homo sapiens GN=UAP1L1 PE=1 SV=2 | UAP1L_HUMAN | 57 kDa |  | 3.70% | 2 | 2 | 2 |
| 1593 | Beta-parvin OS=Homo sapiens GN=PARVB PE=1 SV=1 | PARVB_HUMAN | 42 kDa | TRUE | 7.40% | 2 | 3 | 3 |
| 1594 | Thiopurine S-methyltransferase OS=Homo sapiens GN=TPMT PE=1 SV=1 | TPMT_HUMAN | 28 kDa |  | 7.80% | 2 | 2 | 2 |
| 1595 | Cytosolic Fe-S cluster assembly factor NUBP1 OS=Homo sapiens GN=NUBP1 PE=1 SV=2 | NUBP1_HUMAN | 35 kDa |  | 11% | 2 | 2 | 2 |
| 1596 | Vacuolar protein sorting-associated protein 53 homolog OS=Homo sapiens GN=VPS53 PE=1 SV=1 | VPS53_HUMAN | 80 kDa | TRUE | 4.60% | 2 | 2 | 2 |
| 1597 | 3-ketoacyl-CoA thiolase, mitochondrial OS=Homo sapiens GN=ACAA2 PE=1 SV=2 | THIM_HUMAN | 42 kDa |  | 5.30% | 2 | 3 | 3 |
| 1598 | Transcription factor IIIB 90 kDa subunit OS=Homo sapiens GN=BRF1 PE=1 SV=1 | TF3B_HUMAN | 74 kDa | TRUE | 2.40% | 2 | 3 | 4 |
| 1599 | Ig heavy chain V-I region EU OS=Homo sapiens PE=1 SV=1 | HV101_HUMAN | 12 kDa |  | 23% | 2 | 2 | 2 |
| 1600 | NLR family CARD domain-containing protein 4 OS=Homo sapiens GN=NLRC4 PE=1 SV=2 | NLRC4_HUMAN | 116 kDa | TRUE | 1.60% | 2 | 2 | 2 |
| 1601 | Kelch repeat and BTB domain-containing protein 11 OS=Homo sapiens GN=KBTBD11 PE=1 SV=1 | KBTBB_HUMAN | 66 kDa | TRUE | 2.70% | 2 | 2 | 2 |
| 1602 | Lys-63-specific deubiquitinase BRCC36 OS=Homo sapiens GN=BRCC3 PE=1 SV=2 | BRCC3_HUMAN | 36 kDa |  | 22% | 4 | 4 | 4 |
| 1603 | Copine-3 OS=Homo sapiens GN=CPNE3 PE=1 SV=1 | CPNE3_HUMAN | 60 kDa |  | 8.60% | 4 | 4 | 4 |
| 1604 | Rab GDP dissociation inhibitor alpha OS=Homo sapiens GN=GDI1 PE=1 SV=2 | GDIA_HUMAN | 51 kDa | TRUE | 28% | 4 | 4 | 12 |
| 1605 | GEM-interacting protein OS=Homo sapiens GN=GMIP PE=1 SV=2 | GMIP_HUMAN | 107 kDa | TRUE | 5.30% | 3 | 3 | 4 |
| 1606 | Putative GTP cyclohydrolase 1 type 2 NIF3L1 OS=Homo sapiens GN=NIF3L1 PE=1 SV=2 | GTPC1_HUMAN | ? |  | 0.00% | 4 | 4 | 4 |
| 1607 | Glycylpeptide N-tetradecanoyltransferase 1 OS=Homo sapiens GN=NMT1 PE=1 SV=2 | NMT1_HUMAN | 57 kDa | TRUE | 7.90% | 3 | 3 | 3 |
| 1608 | Receptor-type tyrosine-protein phosphatase epsilon OS=Homo sapiens GN=PTPRE PE=1 SV=1 | PTPRE_HUMAN | 81 kDa |  | 3.90% | 3 | 3 | 3 |
| 1609 | Splicing factor 3A subunit 2 OS=Homo sapiens GN=SF3A2 PE=1 SV=2 | SF3A2_HUMAN | 49 kDa |  | 9.10% | 3 | 3 | 3 |
| 1610 | Serrate RNA effector molecule homolog OS=Homo sapiens GN=SRRT PE=1 SV=1 | SRRT_HUMAN | 101 kDa |  | 4.70% | 4 | 4 | 4 |
| 1611 | Apolipoprotein F OS=Homo sapiens GN=APOF PE=1 SV=2 | APOF_HUMAN | 35 kDa |  | 10% | 3 | 3 | 3 |
| 1612 | BAG family molecular chaperone regulator 5 OS=Homo sapiens GN=BAG5 PE=1 SV=1 | BAG5_HUMAN | 51 kDa | TRUE | 9.80% | 3 | 3 | 3 |
| 1613 | Cleavage and polyadenylation specificity factor subunit 6 OS=Homo sapiens GN=CPSF6 PE=1 SV=2 | CPSF6_HUMAN | 59 kDa |  | 9.30% | 3 | 3 | 3 |
| 1614 | Protein FAM45A OS=Homo sapiens GN=FAM45A PE=2 SV=1 | FA45A_HUMAN | 41 kDa |  | 9.80% | 3 | 3 | 3 |
| 1615 | Golgi phosphoprotein 3 OS=Homo sapiens GN=GOLPH3 PE=1 SV=1 | GOLP3_HUMAN | 34 kDa |  | 17% | 4 | 4 | 4 |
| 1616 | HCLS1-binding protein 3 OS=Homo sapiens GN=HS1BP3 PE=1 SV=1 | H1BP3_HUMAN | 43 kDa |  | 11% | 3 | 4 | 4 |
| 1617 | Leucine-rich repeat-containing protein 59 OS=Homo sapiens GN=LRRC59 PE=1 SV=1 | LRC59_HUMAN | 35 kDa | TRUE | 10% | 3 | 3 | 3 |
| 1618 | Coactosin-like protein OS=Homo sapiens GN=COTL1 PE=1 SV=3 | COTL1_HUMAN | 16 kDa |  | 19% | 3 | 3 | 3 |
| 1619 | ATP-dependent RNA helicase DDX42 OS=Homo sapiens GN=DDX42 PE=1 SV=1 | DDX42_HUMAN | 103 kDa |  | 5.90% | 4 | 4 | 4 |
| 1620 | Tubulin beta-6 chain OS=Homo sapiens GN=TUBB6 PE=1 SV=1 | TBB6_HUMAN | 50 kDa | TRUE | 39% | 3 | 4 | 46 |
| 1621 | Serine/threonine-protein phosphatase 2A 65 kDa regulatory subunit A beta isoform OS=Homo sapiens GN=PPP2R1B PE=1 SV=3 | 2AAB_HUMAN | 66 kDa | TRUE | 17% | 3 | 3 | 10 |
| 1622 | Serine/threonine-protein phosphatase PP1-beta catalytic subunit OS=Homo sapiens GN=PPP1CB PE=1 SV=3 | PP1B_HUMAN | 37 kDa | TRUE | 25% | 3 | 3 | 9 |
| 1623 | Cysteine protease ATG4B OS=Homo sapiens GN=ATG4B PE=1 SV=2 | ATG4B_HUMAN | 44 kDa |  | 12% | 3 | 3 | 3 |
| 1624 | Protocadherin-12 OS=Homo sapiens GN=PCDH12 PE=1 SV=1 | PCD12_HUMAN | 129 kDa |  | 2.20% | 2 | 2 | 2 |
| 1625 | Nuclear inhibitor of protein phosphatase 1 OS=Homo sapiens GN=PPP1R8 PE=1 SV=2 | PP1R8_HUMAN | 38 kDa |  | 18% | 3 | 3 | 3 |
| 1626 | 60S ribosomal protein L23 OS=Homo sapiens GN=RPL23 PE=1 SV=1 | RL23_HUMAN | 15 kDa |  | 24% | 3 | 4 | 4 |
| 1627 | Uridine 5'-monophosphate synthase OS=Homo sapiens GN=UMPS PE=1 SV=1 | UMPS_HUMAN | 52 kDa |  | 8.30% | 4 | 4 | 4 |
| 1628 | NEDD8-conjugating enzyme Ubc12 OS=Homo sapiens GN=UBE2M PE=1 SV=1 | UBC12_HUMAN | 21 kDa | TRUE | 11% | 2 | 2 | 2 |
| 1629 | PDZ domain-containing protein GIPC1 OS=Homo sapiens GN=GIPC1 PE=1 SV=2 | GIPC1_HUMAN | 36 kDa |  | 11% | 3 | 3 | 3 |
| 1630 | Rho GTPase-activating protein 18 OS=Homo sapiens GN=ARHGAP18 PE=1 SV=3 | RHG18_HUMAN | 75 kDa |  | 3.90% | 2 | 2 | 2 |
| 1631 | Prolyl 4-hydroxylase subunit alpha-2 OS=Homo sapiens GN=P4HA2 PE=1 SV=1 | P4HA2_HUMAN | 61 kDa |  | 6.40% | 3 | 3 | 4 |
| 1632 | Tubulin beta-4A chain OS=Homo sapiens GN=TUBB4A PE=1 SV=2 | TBB4A_HUMAN | 50 kDa | TRUE | 60% | 3 | 4 | 82 |
| 1633 | Cytohesin-1 OS=Homo sapiens GN=CYTH1 PE=1 SV=1 | CYH1_HUMAN | 46 kDa |  | 9.80% | 3 | 4 | 4 |
| 1634 | FH1/FH2 domain-containing protein 1 OS=Homo sapiens GN=FHOD1 PE=1 SV=3 | FHOD1_HUMAN | 127 kDa |  | 3.80% | 2 | 2 | 2 |
| 1635 | Ras-related protein Rab-32 OS=Homo sapiens GN=RAB32 PE=1 SV=3 | RAB32_HUMAN | 25 kDa |  | 13% | 3 | 3 | 3 |
| 1636 | GDP-mannose 4,6 dehydratase OS=Homo sapiens GN=GMDS PE=1 SV=1 | GMDS_HUMAN | 42 kDa |  | 8.30% | 3 | 3 | 3 |
| 1637 | E3 ubiquitin-protein ligase TRIM22 OS=Homo sapiens GN=TRIM22 PE=1 SV=1 | TRI22_HUMAN | 57 kDa |  | 7.60% | 2 | 2 | 2 |
| 1638 | Rho guanine nucleotide exchange factor 7 OS=Homo sapiens GN=ARHGEF7 PE=1 SV=2 | ARHG7_HUMAN | 90 kDa |  | 4.00% | 2 | 2 | 2 |
| 1639 | Branched-chain-amino-acid aminotransferase, cytosolic OS=Homo sapiens GN=BCAT1 PE=1 SV=3 | BCAT1_HUMAN | 43 kDa |  | 8.80% | 3 | 4 | 4 |
| 1640 | Phosphatidylinositol transfer protein alpha isoform OS=Homo sapiens GN=PITPNA PE=1 SV=2 | PIPNA_HUMAN | 32 kDa |  | 11% | 2 | 2 | 2 |
| 1641 | Monoglyceride lipase OS=Homo sapiens GN=MGLL PE=1 SV=2 | MGLL_HUMAN | 33 kDa |  | 8.60% | 2 | 2 | 2 |
| 1642 | Tubulin gamma-1 chain OS=Homo sapiens GN=TUBG1 PE=1 SV=2 | TBG1_HUMAN | 51 kDa |  | 8.20% | 2 | 2 | 3 |
| 1643 | Ubiquitin-conjugating enzyme E2 K OS=Homo sapiens GN=UBE2K PE=1 SV=3 | UBE2K_HUMAN | 22 kDa |  | 14% | 2 | 3 | 3 |
| 1644 | Golgi resident protein GCP60 OS=Homo sapiens GN=ACBD3 PE=1 SV=4 | GCP60_HUMAN | 61 kDa |  | 7.00% | 2 | 3 | 3 |
| 1645 | Prefoldin subunit 4 OS=Homo sapiens GN=PFDN4 PE=1 SV=1 | PFD4_HUMAN | 15 kDa |  | 20% | 2 | 2 | 3 |
| 1646 | Multifunctional methyltransferase subunit TRM112-like protein OS=Homo sapiens GN=TRMT112 PE=1 SV=1 | TR112_HUMAN | 14 kDa |  | 22% | 2 | 2 | 2 |
| 1647 | AP-2 complex subunit sigma OS=Homo sapiens GN=AP2S1 PE=1 SV=2 | AP2S1_HUMAN | 17 kDa |  | 19% | 2 | 3 | 3 |
| 1648 | Splicing factor 3B subunit 4 OS=Homo sapiens GN=SF3B4 PE=1 SV=1 | SF3B4_HUMAN | 44 kDa |  | 10% | 2 | 3 | 3 |
| 1649 | 60S ribosomal protein L36 OS=Homo sapiens GN=RPL36 PE=1 SV=3 | RL36_HUMAN | 12 kDa |  | 18% | 2 | 2 | 2 |
| 1650 | Glycerophosphocholine phosphodiesterase GPCPD1 OS=Homo sapiens GN=GPCPD1 PE=1 SV=2 | GPCP1_HUMAN | 76 kDa |  | 4.30% | 3 | 3 | 3 |
| 1651 | Signal-induced proliferation-associated protein 1 OS=Homo sapiens GN=SIPA1 PE=1 SV=1 | SIPA1_HUMAN | 112 kDa |  | 2.70% | 2 | 2 | 2 |
| 1652 | Oxidoreductase HTATIP2 OS=Homo sapiens GN=HTATIP2 PE=1 SV=2 | HTAI2_HUMAN | 27 kDa |  | 8.70% | 2 | 2 | 2 |
| 1653 | Complement component 1 Q subcomponent-binding protein, mitochondrial OS=Homo sapiens GN=C1QBP PE=1 SV=1 | C1QBP_HUMAN | 31 kDa |  | 12% | 2 | 2 | 2 |
| 1654 | Protein phosphatase Slingshot homolog 3 OS=Homo sapiens GN=SSH3 PE=1 SV=2 | SSH3_HUMAN | 73 kDa | TRUE | 3.80% | 2 | 2 | 3 |
| 1655 | CDK5 regulatory subunit-associated protein 3 OS=Homo sapiens GN=CDK5RAP3 PE=1 SV=2 | CK5P3_HUMAN | 57 kDa |  | 5.30% | 2 | 3 | 3 |
| 1656 | Protein XRP2 OS=Homo sapiens GN=RP2 PE=1 SV=4 | XRP2_HUMAN | 40 kDa |  | 10.00% | 3 | 3 | 3 |
| 1657 | Ig kappa chain V-III region IARC/BL41 OS=Homo sapiens PE=4 SV=1 | KV311_HUMAN | 14 kDa | TRUE | 24% | 2 | 3 | 4 |
| 1658 | Small nuclear ribonucleoprotein Sm D1 OS=Homo sapiens GN=SNRPD1 PE=1 SV=1 | SMD1_HUMAN | 13 kDa |  | 28% | 2 | 3 | 3 |
| 1659 | Phostensin OS=Homo sapiens GN=PPP1R18 PE=1 SV=1 | PPR18_HUMAN | 68 kDa |  | 4.60% | 2 | 2 | 2 |
| 1660 | Epsin-1 OS=Homo sapiens GN=EPN1 PE=1 SV=2 | EPN1_HUMAN | 60 kDa |  | 6.80% | 3 | 3 | 3 |
| 1661 | COMM domain-containing protein 5 OS=Homo sapiens GN=COMMD5 PE=1 SV=1 | COMD5_HUMAN | 25 kDa |  | 21% | 3 | 3 | 4 |
| 1662 | Dickkopf-related protein 3 OS=Homo sapiens GN=DKK3 PE=1 SV=2 | DKK3_HUMAN | 38 kDa |  | 11% | 3 | 3 | 3 |
| 1663 | Serine/threonine-protein kinase 38 OS=Homo sapiens GN=STK38 PE=1 SV=1 | STK38_HUMAN | 54 kDa | TRUE | 5.80% | 2 | 2 | 2 |
| 1664 | Protein-tyrosine kinase 2-beta OS=Homo sapiens GN=PTK2B PE=1 SV=2 | FAK2_HUMAN | 116 kDa |  | 2.10% | 2 | 2 | 2 |
| 1665 | Collagen type IV alpha-3-binding protein OS=Homo sapiens GN=COL4A3BP PE=1 SV=1 | C43BP_HUMAN | 71 kDa |  | 4.60% | 3 | 3 | 3 |
| 1666 | TAR DNA-binding protein 43 OS=Homo sapiens GN=TARDBP PE=1 SV=1 | TADBP_HUMAN | 45 kDa | TRUE | 8.50% | 2 | 3 | 3 |
| 1667 | D-dopachrome decarboxylase OS=Homo sapiens GN=DDT PE=1 SV=3 | DOPD_HUMAN | 13 kDa |  | 15% | 2 | 2 | 2 |
| 1668 | Ras-related GTP-binding protein C OS=Homo sapiens GN=RRAGC PE=1 SV=1 | RRAGC_HUMAN | 44 kDa |  | 6.50% | 2 | 2 | 2 |
| 1669 | Vascular non-inflammatory molecule 2 OS=Homo sapiens GN=VNN2 PE=1 SV=3 | VNN2_HUMAN | 59 kDa |  | 4.20% | 2 | 2 | 3 |
| 1670 | Ras-related protein Rab-27A OS=Homo sapiens GN=RAB27A PE=1 SV=3 | RB27A_HUMAN | 25 kDa |  | 12% | 3 | 3 | 3 |
| 1671 | Regulator of G-protein signaling 10 OS=Homo sapiens GN=RGS10 PE=1 SV=2 | RGS10_HUMAN | 20 kDa |  | 16% | 2 | 2 | 3 |
| 1672 | tRNA (cytosine(34)-C(5))-methyltransferase OS=Homo sapiens GN=NSUN2 PE=1 SV=2 | NSUN2_HUMAN | 86 kDa |  | 2.50% | 2 | 2 | 2 |
| 1673 | Dihydropyrimidine dehydrogenase [NADP(+)] OS=Homo sapiens GN=DPYD PE=1 SV=2 | DPYD_HUMAN | 111 kDa |  | 2.30% | 2 | 2 | 2 |
| 1674 | Endophilin-B1 OS=Homo sapiens GN=SH3GLB1 PE=1 SV=1 | SHLB1_HUMAN | 41 kDa | TRUE | 7.40% | 2 | 2 | 3 |
| 1675 | LanC-like protein 1 OS=Homo sapiens GN=LANCL1 PE=1 SV=1 | LANC1_HUMAN | 45 kDa |  | 5.00% | 2 | 2 | 2 |
| 1676 | Laminin subunit alpha-5 OS=Homo sapiens GN=LAMA5 PE=1 SV=8 | LAMA5_HUMAN | 400 kDa |  | 0.95% | 2 | 2 | 2 |
| 1677 | Acetyl-CoA acetyltransferase, mitochondrial OS=Homo sapiens GN=ACAT1 PE=1 SV=1 | THIL_HUMAN | 45 kDa |  | 6.10% | 2 | 2 | 2 |
| 1678 | Tetratricopeptide repeat protein 1 OS=Homo sapiens GN=TTC1 PE=1 SV=1 | TTC1_HUMAN | 34 kDa | TRUE | 6.20% | 2 | 2 | 3 |
| 1679 | Selenide, water dikinase 1 OS=Homo sapiens GN=SEPHS1 PE=1 SV=2 | SPS1_HUMAN | 43 kDa |  | 6.90% | 2 | 3 | 3 |
| 1680 | Barrier-to-autointegration factor OS=Homo sapiens GN=BANF1 PE=1 SV=1 | BAF_HUMAN | 10 kDa |  | 21% | 2 | 2 | 2 |
| 1681 | Kallikrein-10 OS=Homo sapiens GN=KLK10 PE=1 SV=3 | KLK10_HUMAN | 30 kDa |  | 9.40% | 2 | 3 | 3 |
| 1682 | DAZ-associated protein 1 OS=Homo sapiens GN=DAZAP1 PE=1 SV=1 | DAZP1_HUMAN | 43 kDa |  | 10% | 2 | 2 | 2 |
| 1683 | DNA replication licensing factor MCM2 OS=Homo sapiens GN=MCM2 PE=1 SV=4 | MCM2_HUMAN | 102 kDa |  | 2.10% | 2 | 2 | 2 |
| 1684 | CTP synthase 2 OS=Homo sapiens GN=CTPS2 PE=1 SV=1 | PYRG2_HUMAN | 66 kDa |  | 4.30% | 2 | 2 | 2 |
| 1685 | BRISC complex subunit Abro1 OS=Homo sapiens GN=FAM175B PE=1 SV=2 | F175B_HUMAN | 47 kDa |  | 3.90% | 2 | 2 | 2 |
| 1686 | Neurobeachin-like protein 2 OS=Homo sapiens GN=NBEAL2 PE=1 SV=2 | NBEL2_HUMAN | 303 kDa |  | 0.91% | 2 | 2 | 2 |
| 1687 | Proteoglycan 4 OS=Homo sapiens GN=PRG4 PE=1 SV=2 | PRG4_HUMAN | 151 kDa |  | 1.50% | 2 | 2 | 2 |
| 1688 | Paired amphipathic helix protein Sin3a OS=Homo sapiens GN=SIN3A PE=1 SV=2 | SIN3A_HUMAN | 145 kDa | TRUE | 1.80% | 2 | 2 | 2 |
| 1689 | Tubulin alpha-1A chain OS=Homo sapiens GN=TUBA1A PE=1 SV=1 | TBA1A_HUMAN | 50 kDa | TRUE | 66% | 2 | 3 | 107 |
| 1690 | Syntaxin-binding protein 5 OS=Homo sapiens GN=STXBP5 PE=1 SV=1 | STXB5_HUMAN | 128 kDa |  | 2.50% | 2 | 2 | 2 |
| 1691 | Charged multivesicular body protein 4b OS=Homo sapiens GN=CHMP4B PE=1 SV=1 | CHM4B_HUMAN | 25 kDa |  | 9.40% | 2 | 2 | 2 |
| 1692 | Leucine-rich repeat-containing protein 57 OS=Homo sapiens GN=LRRC57 PE=1 SV=1 | LRC57_HUMAN | 27 kDa |  | 6.70% | 2 | 2 | 3 |
| 1693 | Double-strand break repair protein MRE11A OS=Homo sapiens GN=MRE11A PE=1 SV=3 | MRE11_HUMAN | 81 kDa | TRUE | 2.30% | 2 | 2 | 2 |
| 1694 | Lysyl oxidase homolog 2 OS=Homo sapiens GN=LOXL2 PE=1 SV=1 | LOXL2_HUMAN | 87 kDa | TRUE | 3.50% | 2 | 2 | 2 |
| 1695 | Synaptobrevin homolog YKT6 OS=Homo sapiens GN=YKT6 PE=1 SV=1 | YKT6_HUMAN | 22 kDa |  | 11% | 2 | 3 | 3 |
| 1696 | Caspase-8 OS=Homo sapiens GN=CASP8 PE=1 SV=1 | CASP8_HUMAN | 55 kDa | TRUE | 3.80% | 2 | 2 | 2 |
| 1697 | BUB3-interacting and GLEBS motif-containing protein ZNF207 OS=Homo sapiens GN=ZNF207 PE=1 SV=1 | ZN207_HUMAN | 51 kDa |  | 4.20% | 2 | 2 | 2 |
| 1698 | Filaggrin-2 OS=Homo sapiens GN=FLG2 PE=1 SV=1 | FILA2_HUMAN | 248 kDa |  | 1.00% | 2 | 2 | 3 |
| 1699 | Splicing factor 3B subunit 5 OS=Homo sapiens GN=SF3B5 PE=1 SV=1 | SF3B5_HUMAN | 10 kDa |  | 26% | 2 | 2 | 3 |
| 1700 | UPF0364 protein C6orf211 OS=Homo sapiens GN=C6orf211 PE=1 SV=1 | CF211_HUMAN | ? |  | 0.00% | 2 | 3 | 4 |
| 1701 | Guanine nucleotide-binding protein G(I)/G(S)/G(T) subunit beta-1 OS=Homo sapiens GN=GNB1 PE=1 SV=3 | GBB1_HUMAN | 37 kDa | TRUE | 24% | 2 | 3 | 18 |
| 1702 | Cysteine-rich secretory protein LCCL domain-containing 2 OS=Homo sapiens GN=CRISPLD2 PE=2 SV=1 | CRLD2_HUMAN | 56 kDa |  | 8.90% | 3 | 3 | 3 |
| 1703 | Aminopeptidase N OS=Homo sapiens GN=ANPEP PE=1 SV=4 | AMPN_HUMAN | 110 kDa | TRUE | 2.20% | 2 | 2 | 2 |
| 1704 | RILP-like protein 2 OS=Homo sapiens GN=RILPL2 PE=1 SV=1 | RIPL2_HUMAN | 24 kDa | TRUE | 12% | 3 | 3 | 3 |
| 1705 | Quinone oxidoreductase PIG3 OS=Homo sapiens GN=TP53I3 PE=1 SV=2 | QORX_HUMAN | 36 kDa |  | 7.50% | 2 | 2 | 2 |
| 1706 | Ig lambda chain V-II region NIG-84 OS=Homo sapiens PE=1 SV=1 | LV211_HUMAN | 12 kDa | TRUE | 21% | 2 | 2 | 2 |
| 1707 | Putative small nuclear ribonucleoprotein G-like protein 15 OS=Homo sapiens GN=SNRPGP15 PE=5 SV=2 | RUXGL_HUMAN | 9 kDa |  | 18% | 2 | 2 | 3 |
| 1708 | N-alpha-acetyltransferase 10 OS=Homo sapiens GN=NAA10 PE=1 SV=1 | NAA10_HUMAN | 26 kDa |  | 16% | 3 | 3 | 3 |
| 1709 | Ribosyldihydronicotinamide dehydrogenase [quinone] OS=Homo sapiens GN=NQO2 PE=1 SV=5 | NQO2_HUMAN | 26 kDa |  | 13% | 3 | 3 | 3 |
| 1710 | Syntaxin-7 OS=Homo sapiens GN=STX7 PE=1 SV=4 | STX7_HUMAN | 30 kDa |  | 16% | 3 | 3 | 3 |
| 1711 | Caspase recruitment domain-containing protein 8 OS=Homo sapiens GN=CARD8 PE=1 SV=1 | CARD8_HUMAN | 49 kDa |  | 8.80% | 3 | 3 | 3 |
| 1712 | Procollagen-lysine,2-oxoglutarate 5-dioxygenase 3 OS=Homo sapiens GN=PLOD3 PE=1 SV=1 | PLOD3_HUMAN | 85 kDa |  | 5.30% | 3 | 3 | 3 |
| 1713 | Vacuolar protein-sorting-associated protein 25 OS=Homo sapiens GN=VPS25 PE=1 SV=1 | VPS25_HUMAN | 21 kDa |  | 20% | 3 | 3 | 3 |
| 1714 | Peptidyl-glycine alpha-amidating monooxygenase OS=Homo sapiens GN=PAM PE=1 SV=2 | AMD_HUMAN | 108 kDa | TRUE | 5.10% | 3 | 3 | 3 |
| 1715 | Peptidyl-prolyl cis-trans isomerase FKBP9 OS=Homo sapiens GN=FKBP9 PE=1 SV=2 | FKBP9_HUMAN | 63 kDa |  | 5.40% | 3 | 3 | 3 |
| 1716 | Armadillo repeat-containing protein 8 OS=Homo sapiens GN=ARMC8 PE=1 SV=2 | ARMC8_HUMAN | 76 kDa |  | 5.60% | 3 | 3 | 3 |
| 1717 | Cadherin-11 OS=Homo sapiens GN=CDH11 PE=2 SV=2 | CAD11_HUMAN | 88 kDa |  | 4.80% | 3 | 3 | 3 |
| 1718 | Protein MEMO1 OS=Homo sapiens GN=MEMO1 PE=1 SV=1 | MEMO1_HUMAN | 34 kDa |  | 9.10% | 2 | 2 | 2 |
| 1719 | Papilin OS=Homo sapiens GN=PAPLN PE=2 SV=4 | PPN_HUMAN | 138 kDa |  | 3.60% | 3 | 3 | 3 |
| 1720 | Serine/threonine-protein phosphatase 2A 56 kDa regulatory subunit alpha isoform OS=Homo sapiens GN=PPP2R5A PE=1 SV=1 | 2A5A_HUMAN | 56 kDa | TRUE | 5.80% | 2 | 2 | 2 |
| 1721 | Coiled-coil domain-containing protein 6 OS=Homo sapiens GN=CCDC6 PE=1 SV=2 | CCDC6_HUMAN | 53 kDa |  | 6.50% | 3 | 3 | 3 |
| 1722 | Pentraxin-related protein PTX3 OS=Homo sapiens GN=PTX3 PE=1 SV=3 | PTX3_HUMAN | 42 kDa |  | 11% | 3 | 3 | 3 |
| 1723 | Echinoderm microtubule-associated protein-like 4 OS=Homo sapiens GN=EML4 PE=1 SV=3 | EMAL4_HUMAN | 109 kDa |  | 2.40% | 2 | 2 | 2 |
| 1724 | Cystatin-A OS=Homo sapiens GN=CSTA PE=1 SV=1 | CYTA_HUMAN | 11 kDa |  | 31% | 2 | 2 | 2 |
| 1725 | WASH complex subunit strumpellin OS=Homo sapiens GN=KIAA0196 PE=1 SV=1 | STRUM_HUMAN | 134 kDa | TRUE | 2.40% | 2 | 2 | 2 |
| 1726 | Protein transport protein Sec23B OS=Homo sapiens GN=SEC23B PE=1 SV=2 | SC23B_HUMAN | 86 kDa | TRUE | 8.30% | 2 | 2 | 7 |
| 1727 | AP-2 complex subunit alpha-2 OS=Homo sapiens GN=AP2A2 PE=1 SV=2 | AP2A2_HUMAN | 104 kDa | TRUE | 12% | 2 | 2 | 12 |
| 1728 | Eukaryotic translation initiation factor 3 subunit K OS=Homo sapiens GN=EIF3K PE=1 SV=1 | EIF3K_HUMAN | 25 kDa |  | 13% | 2 | 2 | 2 |
| 1729 | Dystroglycan OS=Homo sapiens GN=DAG1 PE=1 SV=2 | DAG1_HUMAN | 97 kDa |  | 5.50% | 2 | 2 | 2 |
| 1730 | Ubiquitin-conjugating enzyme E2 N OS=Homo sapiens GN=UBE2N PE=1 SV=1 | UBE2N_HUMAN | 17 kDa |  | 14% | 2 | 2 | 2 |
| 1731 | Protein-glutamine gamma-glutamyltransferase E OS=Homo sapiens GN=TGM3 PE=1 SV=4 | TGM3_HUMAN | 77 kDa |  | 3.90% | 3 | 3 | 3 |
| 1732 | Protein phosphatase 1 regulatory subunit 21 OS=Homo sapiens GN=PPP1R21 PE=1 SV=1 | PPR21_HUMAN | 88 kDa |  | 3.30% | 2 | 2 | 2 |
| 1733 | Ras-related protein Rab-4B OS=Homo sapiens GN=RAB4B PE=1 SV=1 | RAB4B_HUMAN | 24 kDa | TRUE | 16% | 2 | 2 | 4 |
| 1734 | L-amino-acid oxidase OS=Homo sapiens GN=IL4I1 PE=1 SV=1 | OXLA_HUMAN | 63 kDa |  | 5.10% | 2 | 2 | 2 |
| 1735 | TBC1 domain family member 13 OS=Homo sapiens GN=TBC1D13 PE=1 SV=3 | TBC13_HUMAN | 47 kDa |  | 6.50% | 2 | 2 | 2 |
| 1736 | Cytoplasmic FMR1-interacting protein 1 OS=Homo sapiens GN=CYFIP1 PE=1 SV=1 | CYFP1_HUMAN | 145 kDa | TRUE | 5.70% | 2 | 2 | 8 |
| 1737 | Vacuolar protein sorting-associated protein 37B OS=Homo sapiens GN=VPS37B PE=1 SV=1 | VP37B_HUMAN | 31 kDa |  | 10% | 2 | 2 | 2 |
| 1738 | N-terminal kinase-like protein OS=Homo sapiens GN=SCYL1 PE=1 SV=1 | NTKL_HUMAN | 90 kDa |  | 2.50% | 2 | 2 | 2 |
| 1739 | ADAM DEC1 OS=Homo sapiens GN=ADAMDEC1 PE=1 SV=2 | ADEC1_HUMAN | 53 kDa |  | 5.10% | 2 | 3 | 3 |
| 1740 | Alcohol dehydrogenase [NADP(+)] OS=Homo sapiens GN=AKR1A1 PE=1 SV=3 | AK1A1_HUMAN | 37 kDa |  | 10% | 2 | 3 | 3 |
| 1741 | AP-1 complex subunit sigma-1A OS=Homo sapiens GN=AP1S1 PE=1 SV=1 | AP1S1_HUMAN | 19 kDa |  | 18% | 2 | 2 | 2 |
| 1742 | Myosin regulatory light polypeptide 9 OS=Homo sapiens GN=MYL9 PE=1 SV=4 | MYL9_HUMAN | 20 kDa | TRUE | 60% | 2 | 3 | 21 |
| 1743 | Ribose-phosphate pyrophosphokinase 2 OS=Homo sapiens GN=PRPS2 PE=1 SV=2 | PRPS2_HUMAN | 35 kDa | TRUE | 30% | 2 | 3 | 22 |
| 1744 | Ras-related protein Rab-5A OS=Homo sapiens GN=RAB5A PE=1 SV=2 | RAB5A_HUMAN | 24 kDa | TRUE | 29% | 2 | 2 | 8 |
| 1745 | Tumor necrosis factor receptor type 1-associated DEATH domain protein OS=Homo sapiens GN=TRADD PE=1 SV=2 | TRADD_HUMAN | 34 kDa | TRUE | 9.30% | 2 | 2 | 2 |
| 1746 | Interleukin-1 beta OS=Homo sapiens GN=IL1B PE=1 SV=2 | IL1B_HUMAN | 31 kDa |  | 9.70% | 2 | 2 | 2 |
| 1747 | (E3-independent) E2 ubiquitin-conjugating enzyme OS=Homo sapiens GN=UBE2O PE=1 SV=3 | UBE2O_HUMAN | 141 kDa |  | 1.50% | 2 | 2 | 3 |
| 1748 | Tether containing UBX domain for GLUT4 OS=Homo sapiens GN=ASPSCR1 PE=1 SV=1 | ASPC1_HUMAN | 60 kDa |  | 5.60% | 2 | 2 | 2 |
| 1749 | Osteopontin OS=Homo sapiens GN=SPP1 PE=1 SV=1 | OSTP_HUMAN | 35 kDa |  | 10% | 2 | 2 | 3 |
| 1750 | Ubiquitin conjugation factor E4 B OS=Homo sapiens GN=UBE4B PE=1 SV=1 | UBE4B_HUMAN | 146 kDa | TRUE | 1.80% | 2 | 2 | 2 |
| 1751 | Programmed cell death protein 5 OS=Homo sapiens GN=PDCD5 PE=1 SV=3 | PDCD5_HUMAN | 14 kDa | TRUE | 19% | 2 | 2 | 2 |
| 1752 | Thioredoxin domain-containing protein 5 OS=Homo sapiens GN=TXNDC5 PE=1 SV=2 | TXND5_HUMAN | 48 kDa |  | 6.70% | 2 | 2 | 2 |
| 1753 | Phosphopantothenoylcysteine decarboxylase OS=Homo sapiens GN=PPCDC PE=1 SV=2 | COAC_HUMAN | 22 kDa |  | 14% | 2 | 3 | 3 |
| 1754 | Poly [ADP-ribose] polymerase 14 OS=Homo sapiens GN=PARP14 PE=1 SV=3 | PAR14_HUMAN | 203 kDa | TRUE | 1.90% | 2 | 2 | 2 |
| 1755 | Cytosolic acyl coenzyme A thioester hydrolase OS=Homo sapiens GN=ACOT7 PE=1 SV=3 | BACH_HUMAN | 42 kDa |  | 11% | 3 | 3 | 3 |
| 1756 | FAS-associated death domain protein OS=Homo sapiens GN=FADD PE=1 SV=1 | FADD_HUMAN | 23 kDa |  | 12% | 3 | 3 | 3 |
| 1757 | WD repeat domain phosphoinositide-interacting protein 2 OS=Homo sapiens GN=WIPI2 PE=1 SV=1 | WIPI2_HUMAN | 49 kDa |  | 9.90% | 2 | 2 | 2 |
| 1758 | Destrin OS=Homo sapiens GN=DSTN PE=1 SV=3 | DEST_HUMAN | 19 kDa | TRUE | 21% | 2 | 2 | 18 |
| 1759 | TATA-binding protein-associated factor 2N OS=Homo sapiens GN=TAF15 PE=1 SV=1 | RBP56_HUMAN | 62 kDa | TRUE | 11% | 2 | 2 | 6 |
| 1760 | Platelet basic protein OS=Homo sapiens GN=PPBP PE=1 SV=3 | CXCL7_HUMAN | 14 kDa |  | 20% | 2 | 3 | 3 |
| 1761 | Integrin beta-1 OS=Homo sapiens GN=ITGB1 PE=1 SV=2 | ITB1_HUMAN | 88 kDa | TRUE | 3.80% | 2 | 2 | 2 |
| 1762 | Radixin OS=Homo sapiens GN=RDX PE=1 SV=1 | RADI_HUMAN | 69 kDa | TRUE | 20% | 2 | 2 | 20 |
| 1763 | ATP synthase subunit alpha, mitochondrial OS=Homo sapiens GN=ATP5A1 PE=1 SV=1 | ATPA_HUMAN | 60 kDa |  | 4.20% | 2 | 2 | 2 |
| 1764 | Histone H2A type 2-B OS=Homo sapiens GN=HIST2H2AB PE=1 SV=3 | H2A2B_HUMAN | 14 kDa | TRUE | 62% | 2 | 2 | 37 |
| 1765 | BolA-like protein 2 OS=Homo sapiens GN=BOLA2 PE=1 SV=1 | BOLA2_HUMAN | 10 kDa |  | 41% | 2 | 3 | 3 |
| 1766 | Palladin OS=Homo sapiens GN=PALLD PE=1 SV=3 | PALLD_HUMAN | 151 kDa |  | 2.60% | 2 | 2 | 2 |
| 1767 | Nucleolar RNA helicase 2 OS=Homo sapiens GN=DDX21 PE=1 SV=5 | DDX21_HUMAN | 87 kDa | TRUE | 2.80% | 2 | 2 | 2 |
| 1768 | MAP kinase-activated protein kinase 2 OS=Homo sapiens GN=MAPKAPK2 PE=1 SV=1 | MAPK2_HUMAN | 46 kDa | TRUE | 11% | 2 | 2 | 9 |
| 1769 | DNA dC->dU-editing enzyme APOBEC-3A OS=Homo sapiens GN=APOBEC3A PE=1 SV=3 | ABC3A_HUMAN | 23 kDa |  | 9.00% | 2 | 2 | 2 |
| 1770 | Double-stranded RNA-binding protein Staufen homolog 1 OS=Homo sapiens GN=STAU1 PE=1 SV=2 | STAU1_HUMAN | 63 kDa |  | 3.50% | 2 | 2 | 2 |
| 1771 | Clathrin interactor 1 OS=Homo sapiens GN=CLINT1 PE=1 SV=1 | EPN4_HUMAN | 68 kDa |  | 4.00% | 2 | 2 | 2 |
| 1772 | Regulator of G-protein signaling 14 OS=Homo sapiens GN=RGS14 PE=1 SV=4 | RGS14_HUMAN | 61 kDa |  | 4.80% | 2 | 2 | 2 |
| 1773 | Lambda-crystallin homolog OS=Homo sapiens GN=CRYL1 PE=1 SV=3 | CRYL1_HUMAN | 35 kDa |  | 7.80% | 2 | 2 | 3 |
| 1774 | 2-5A-dependent ribonuclease OS=Homo sapiens GN=RNASEL PE=1 SV=2 | RN5A_HUMAN | 84 kDa |  | 2.70% | 2 | 2 | 2 |
| 1775 | Interleukin-1 receptor-associated kinase 4 OS=Homo sapiens GN=IRAK4 PE=1 SV=1 | IRAK4_HUMAN | 52 kDa |  | 5.40% | 2 | 2 | 2 |
| 1776 | WD repeat and FYVE domain-containing protein 1 OS=Homo sapiens GN=WDFY1 PE=1 SV=1 | WDFY1_HUMAN | 46 kDa | TRUE | 6.60% | 2 | 2 | 3 |
| 1777 | CapZ-interacting protein OS=Homo sapiens GN=RCSD1 PE=1 SV=1 | CPZIP_HUMAN | 45 kDa |  | 5.50% | 2 | 2 | 2 |
| 1778 | F-box only protein 50 OS=Homo sapiens GN=NCCRP1 PE=1 SV=1 | FBX50_HUMAN | 31 kDa |  | 8.40% | 2 | 3 | 3 |
| 1779 | Melanoma-associated antigen D2 OS=Homo sapiens GN=MAGED2 PE=1 SV=2 | MAGD2_HUMAN | 65 kDa |  | 5.30% | 2 | 2 | 2 |
| 1780 | Vacuolar protein sorting-associated protein VTA1 homolog OS=Homo sapiens GN=VTA1 PE=1 SV=1 | VTA1_HUMAN | 34 kDa |  | 5.50% | 2 | 2 | 2 |
| 1781 | GTPase KRas OS=Homo sapiens GN=KRAS PE=1 SV=1 | RASK_HUMAN | 22 kDa |  | 12% | 2 | 3 | 3 |
| 1782 | Nucleolar protein 56 OS=Homo sapiens GN=NOP56 PE=1 SV=4 | NOP56_HUMAN | 66 kDa |  | 3.50% | 2 | 2 | 2 |
| 1783 | Kinesin light chain 4 OS=Homo sapiens GN=KLC4 PE=1 SV=3 | KLC4_HUMAN | 69 kDa | TRUE | 3.20% | 2 | 2 | 2 |
| 1784 | Striatin-3 OS=Homo sapiens GN=STRN3 PE=1 SV=3 | STRN3_HUMAN | 87 kDa |  | 3.50% | 2 | 2 | 2 |
| 1785 | Hexokinase-1 OS=Homo sapiens GN=HK1 PE=1 SV=3 | HXK1_HUMAN | 102 kDa | TRUE | 2.40% | 2 | 2 | 2 |
| 1786 | Erythrocyte membrane protein band 4.2 OS=Homo sapiens GN=EPB42 PE=1 SV=3 | EPB42_HUMAN | 77 kDa |  | 4.30% | 2 | 2 | 2 |
| 1787 | Ubiquitin-conjugating enzyme E2 variant 1 OS=Homo sapiens GN=UBE2V1 PE=1 SV=2 | UB2V1_HUMAN | 16 kDa | TRUE | 14% | 2 | 2 | 2 |
| 1788 | Zinc finger protein ZPR1 OS=Homo sapiens GN=ZPR1 PE=1 SV=1 | ZPR1_HUMAN | 51 kDa |  | 4.10% | 2 | 2 | 2 |
| 1789 | Fibroleukin OS=Homo sapiens GN=FGL2 PE=1 SV=1 | FGL2_HUMAN | 50 kDa | TRUE | 3.90% | 2 | 2 | 2 |
| 1790 | [Protein ADP-ribosylarginine] hydrolase OS=Homo sapiens GN=ADPRH PE=1 SV=1 | ADPRH_HUMAN | 40 kDa |  | 7.60% | 2 | 3 | 3 |
| 1791 | T-complex protein 11-like protein 1 OS=Homo sapiens GN=TCP11L1 PE=1 SV=1 | T11L1_HUMAN | 57 kDa | TRUE | 3.70% | 2 | 2 | 2 |
| 1792 | Multimerin-2 OS=Homo sapiens GN=MMRN2 PE=1 SV=2 | MMRN2_HUMAN | 104 kDa |  | 2.10% | 2 | 3 | 3 |
| 1793 | Transmembrane glycoprotein NMB OS=Homo sapiens GN=GPNMB PE=1 SV=2 | GPNMB_HUMAN | 64 kDa |  | 4.70% | 2 | 3 | 3 |
| 1794 | Phospholipid hydroperoxide glutathione peroxidase, mitochondrial OS=Homo sapiens GN=GPX4 PE=1 SV=3 | GPX4_HUMAN | 22 kDa |  | 13% | 2 | 2 | 2 |
| 1795 | Pleckstrin homology domain-containing family F member 2 OS=Homo sapiens GN=PLEKHF2 PE=1 SV=1 | PKHF2_HUMAN | 28 kDa |  | 8.80% | 2 | 3 | 3 |
| 1796 | TBC1 domain family member 24 OS=Homo sapiens GN=TBC1D24 PE=1 SV=2 | TBC24_HUMAN | 63 kDa |  | 3.90% | 2 | 2 | 2 |
| 1797 | 40S ribosomal protein S27 OS=Homo sapiens GN=RPS27 PE=1 SV=3 | RS27_HUMAN | 9 kDa |  | 23% | 2 | 2 | 2 |
| 1798 | MOB kinase activator 2 OS=Homo sapiens GN=MOB2 PE=1 SV=1 | MOB2_HUMAN | 27 kDa |  | 6.80% | 2 | 2 | 2 |
| 1799 | WD repeat-containing protein 26 OS=Homo sapiens GN=WDR26 PE=1 SV=3 | WDR26_HUMAN | 72 kDa |  | 3.00% | 2 | 2 | 2 |
| 1800 | Amidophosphoribosyltransferase OS=Homo sapiens GN=PPAT PE=1 SV=1 | PUR1_HUMAN | 57 kDa |  | 5.00% | 2 | 2 | 2 |
| 1801 | rRNA 2'-O-methyltransferase fibrillarin OS=Homo sapiens GN=FBL PE=1 SV=2 | FBRL_HUMAN | 34 kDa |  | 4.70% | 2 | 3 | 3 |
| 1802 | CD177 antigen OS=Homo sapiens GN=CD177 PE=1 SV=2 | CD177_HUMAN | 46 kDa |  | 3.90% | 2 | 2 | 2 |
| 1803 | Arf-GAP domain and FG repeat-containing protein 1 OS=Homo sapiens GN=AGFG1 PE=1 SV=2 | AGFG1_HUMAN | 58 kDa | TRUE | 4.40% | 2 | 2 | 2 |
| 1804 | Poly(ADP-ribose) glycohydrolase ARH3 OS=Homo sapiens GN=ADPRHL2 PE=1 SV=1 | ARHL2_HUMAN | 39 kDa |  | 8.50% | 2 | 2 | 2 |
| 1805 | BAG family molecular chaperone regulator 2 OS=Homo sapiens GN=BAG2 PE=1 SV=1 | BAG2_HUMAN | 24 kDa |  | 10.00% | 2 | 2 | 2 |
| 1806 | Carbonic anhydrase 3 OS=Homo sapiens GN=CA3 PE=1 SV=3 | CAH3_HUMAN | 30 kDa |  | 10% | 2 | 2 | 2 |
| 1807 | Mitochondrial enolase superfamily member 1 OS=Homo sapiens GN=ENOSF1 PE=1 SV=1 | ENOF1_HUMAN | 50 kDa |  | 5.00% | 2 | 2 | 2 |
| 1808 | Peptidyl-prolyl cis-trans isomerase FKBP1A OS=Homo sapiens GN=FKBP1A PE=1 SV=2 | FKB1A_HUMAN | 12 kDa |  | 25% | 2 | 2 | 2 |
| 1809 | Pre-mRNA-splicing regulator WTAP OS=Homo sapiens GN=WTAP PE=1 SV=2 | FL2D_HUMAN | 44 kDa |  | 6.80% | 2 | 2 | 2 |
| 1810 | Inositol polyphosphate 1-phosphatase OS=Homo sapiens GN=INPP1 PE=1 SV=1 | INPP_HUMAN | 44 kDa |  | 6.30% | 2 | 2 | 2 |
| 1811 | Lipoprotein lipase OS=Homo sapiens GN=LPL PE=1 SV=1 | LIPL_HUMAN | 53 kDa |  | 5.70% | 2 | 2 | 2 |
| 1812 | Ras-related protein Rab-6B OS=Homo sapiens GN=RAB6B PE=1 SV=1 | RAB6B_HUMAN | 23 kDa | TRUE | 28% | 2 | 2 | 6 |
| 1813 | Tropomodulin-1 OS=Homo sapiens GN=TMOD1 PE=1 SV=1 | TMOD1_HUMAN | 41 kDa | TRUE | 9.20% | 2 | 2 | 6 |
| 1814 | Small nuclear ribonucleoprotein E OS=Homo sapiens GN=SNRPE PE=1 SV=1 | RUXE_HUMAN | 11 kDa |  | 40% | 2 | 2 | 2 |
| 1815 | Casein kinase I isoform alpha OS=Homo sapiens GN=CSNK1A1 PE=1 SV=2 | KC1A_HUMAN | 39 kDa |  | 6.20% | 2 | 2 | 2 |
| 1816 | Apoptosis-associated speck-like protein containing a CARD OS=Homo sapiens GN=PYCARD PE=1 SV=2 | ASC_HUMAN | 22 kDa |  | 14% | 2 | 2 | 2 |
| 1817 | Cytochrome b5 OS=Homo sapiens GN=CYB5A PE=1 SV=2 | CYB5_HUMAN | 15 kDa |  | 26% | 2 | 2 | 2 |
| 1818 | Interactor protein for cytohesin exchange factors 1 OS=Homo sapiens GN=IPCEF1 PE=1 SV=1 | ICEF1_HUMAN | 49 kDa |  | 6.60% | 2 | 2 | 2 |
| 1819 | Retinoid-binding protein 7 OS=Homo sapiens GN=RBP7 PE=1 SV=1 | RET7_HUMAN | 16 kDa |  | 14% | 2 | 2 | 2 |
| 1820 | GTPase IMAP family member 7 OS=Homo sapiens GN=GIMAP7 PE=1 SV=1 | GIMA7_HUMAN | 35 kDa |  | 9.00% | 2 | 2 | 2 |
| 1821 | Rieske domain-containing protein OS=Homo sapiens GN=RFESD PE=1 SV=1 | RFESD_HUMAN | 18 kDa |  | 15% | 2 | 2 | 2 |
| 1822 | DNA-directed RNA polymerases I and III subunit RPAC1 OS=Homo sapiens GN=POLR1C PE=1 SV=1 | RPAC1_HUMAN | 39 kDa |  | 8.40% | 2 | 2 | 2 |
| 1823 | Proto-oncogene c-Rel OS=Homo sapiens GN=REL PE=1 SV=1 | REL_HUMAN | 69 kDa |  | 3.90% | 2 | 2 | 2 |
| 1824 | Signal transducer and activator of transcription 5A OS=Homo sapiens GN=STAT5A PE=1 SV=1 | STA5A_HUMAN | 91 kDa | TRUE | 15% | 2 | 2 | 12 |
| 1825 | Aldehyde dehydrogenase, mitochondrial OS=Homo sapiens GN=ALDH2 PE=1 SV=2 | ALDH2_HUMAN | 56 kDa | TRUE | 6.20% | 2 | 2 | 4 |
| 1826 | Methionine aminopeptidase 2 OS=Homo sapiens GN=METAP2 PE=1 SV=1 | MAP2_HUMAN | 53 kDa |  | 6.50% | 2 | 2 | 2 |
| 1827 | Nucleoredoxin OS=Homo sapiens GN=NXN PE=1 SV=2 | NXN_HUMAN | 48 kDa |  | 7.40% | 2 | 2 | 2 |
| 1828 | Engulfment and cell motility protein 2 OS=Homo sapiens GN=ELMO2 PE=1 SV=2 | ELMO2_HUMAN | 83 kDa | TRUE | 7.40% | 2 | 2 | 7 |
| 1829 | Protein NDRG2 OS=Homo sapiens GN=NDRG2 PE=1 SV=2 | NDRG2_HUMAN | 41 kDa |  | 5.10% | 2 | 2 | 2 |
| 1830 | Apoptosis regulator BAX OS=Homo sapiens GN=BAX PE=1 SV=1 | BAX_HUMAN | 21 kDa |  | 13% | 2 | 2 | 2 |
| 1831 | Biglycan OS=Homo sapiens GN=BGN PE=1 SV=2 | PGS1_HUMAN | 42 kDa | TRUE | 5.40% | 2 | 2 | 2 |
| 1832 | UPF0693 protein C10orf32 OS=Homo sapiens GN=C10orf32 PE=3 SV=1 | CJ032_HUMAN | 12 kDa |  | 26% | 2 | 2 | 2 |
| 1833 | Protein THEMIS2 OS=Homo sapiens GN=THEMIS2 PE=1 SV=1 | THMS2_HUMAN | 72 kDa |  | 4.40% | 2 | 2 | 2 |
| 1834 | DNA repair protein XRCC4 OS=Homo sapiens GN=XRCC4 PE=1 SV=2 | XRCC4_HUMAN | 38 kDa |  | 7.70% | 2 | 2 | 2 |
| 1835 | Ubiquitin-like protein 4A OS=Homo sapiens GN=UBL4A PE=1 SV=1 | UBL4A_HUMAN | 18 kDa |  | 11% | 2 | 2 | 2 |
| 1836 | O-phosphoseryl-tRNA(Sec) selenium transferase OS=Homo sapiens GN=SEPSECS PE=1 SV=2 | SPCS_HUMAN | 56 kDa | TRUE | 4.40% | 2 | 2 | 2 |
| 1837 | PDZ and LIM domain protein 5 OS=Homo sapiens GN=PDLIM5 PE=1 SV=5 | PDLI5_HUMAN | 64 kDa |  | 3.40% | 2 | 2 | 2 |
| 1838 | Exosome complex component RRP42 OS=Homo sapiens GN=EXOSC7 PE=1 SV=3 | EXOS7_HUMAN | 32 kDa |  | 11% | 2 | 2 | 2 |
| 1839 | Phosphatidate phosphatase LPIN2 OS=Homo sapiens GN=LPIN2 PE=1 SV=1 | LPIN2_HUMAN | 99 kDa | TRUE | 2.30% | 2 | 2 | 2 |
| 1840 | Alpha-galactosidase A OS=Homo sapiens GN=GLA PE=1 SV=1 | AGAL_HUMAN | 49 kDa |  | 6.80% | 2 | 2 | 2 |
| 1841 | UPF0553 protein C9orf64 OS=Homo sapiens GN=C9orf64 PE=1 SV=1 | CI064_HUMAN | 39 kDa | TRUE | 6.70% | 2 | 2 | 2 |
| 1842 | Histone H1.0 OS=Homo sapiens GN=H1F0 PE=1 SV=3 | H10_HUMAN | 21 kDa |  | 11% | 2 | 2 | 2 |
| 1843 | Geranylgeranyl pyrophosphate synthase OS=Homo sapiens GN=GGPS1 PE=1 SV=1 | GGPPS_HUMAN | 35 kDa |  | 6.30% | 2 | 2 | 2 |
| 1844 | Protein PML OS=Homo sapiens GN=PML PE=1 SV=3 | PML_HUMAN | 98 kDa | TRUE | 2.30% | 2 | 2 | 2 |
| 1845 | Protein FAM63A OS=Homo sapiens GN=FAM63A PE=1 SV=2 | FA63A_HUMAN | 52 kDa |  | 8.10% | 2 | 2 | 2 |
| 1846 | NHP2-like protein 1 OS=Homo sapiens GN=SNU13 PE=1 SV=3 | NH2L1_HUMAN | 14 kDa |  | 16% | 2 | 2 | 2 |
| 1847 | Pantetheinase OS=Homo sapiens GN=VNN1 PE=1 SV=2 | VNN1_HUMAN | 57 kDa |  | 3.50% | 2 | 2 | 2 |
| 1848 | Actin-related protein 2/3 complex subunit 5-like protein OS=Homo sapiens GN=ARPC5L PE=1 SV=1 | ARP5L_HUMAN | 17 kDa | TRUE | 12% | 2 | 2 | 2 |
| 1849 | Protein farnesyltransferase subunit beta OS=Homo sapiens GN=FNTB PE=1 SV=1 | FNTB_HUMAN | 49 kDa |  | 5.30% | 2 | 2 | 2 |
| 1850 | Superoxide dismutase [Mn], mitochondrial OS=Homo sapiens GN=SOD2 PE=1 SV=2 | SODM_HUMAN | 25 kDa |  | 9.50% | 2 | 2 | 2 |
| 1851 | Metalloproteinase inhibitor 1 OS=Homo sapiens GN=TIMP1 PE=1 SV=1 | TIMP1_HUMAN | 23 kDa |  | 9.20% | 2 | 2 | 2 |
| 1852 | DDRGK domain-containing protein 1 OS=Homo sapiens GN=DDRGK1 PE=1 SV=2 | DDRGK_HUMAN | 36 kDa |  | 7.60% | 2 | 2 | 2 |
| 1853 | Serine protease HTRA2, mitochondrial OS=Homo sapiens GN=HTRA2 PE=1 SV=2 | HTRA2_HUMAN | 49 kDa | TRUE | 5.00% | 2 | 2 | 2 |
| 1854 | Cytoplasmic aconitate hydratase OS=Homo sapiens GN=ACO1 PE=1 SV=3 | ACOC_HUMAN | 98 kDa |  | 2.00% | 2 | 2 | 2 |
| 1855 | Ribonuclease 7 OS=Homo sapiens GN=RNASE7 PE=1 SV=2 | RNAS7_HUMAN | 17 kDa |  | 15% | 2 | 2 | 2 |
| 1856 | Cleavage and polyadenylation specificity factor subunit 5 OS=Homo sapiens GN=NUDT21 PE=1 SV=1 | CPSF5_HUMAN | 26 kDa |  | 8.40% | 2 | 2 | 2 |
| 1857 | V-type proton ATPase subunit d 1 OS=Homo sapiens GN=ATP6V0D1 PE=1 SV=1 | VA0D1_HUMAN | 40 kDa |  | 4.60% | 2 | 2 | 2 |
| 1858 | Inorganic pyrophosphatase 2, mitochondrial OS=Homo sapiens GN=PPA2 PE=1 SV=2 | IPYR2_HUMAN | 38 kDa |  | 9.90% | 2 | 2 | 4 |
| 1859 | Ribonucleoprotein PTB-binding 1 OS=Homo sapiens GN=RAVER1 PE=1 SV=1 | RAVR1_HUMAN | 64 kDa |  | 6.10% | 2 | 2 | 2 |
| 1860 | Rab proteins geranylgeranyltransferase component A 1 OS=Homo sapiens GN=CHM PE=1 SV=3 | RAE1_HUMAN | 73 kDa |  | 2.30% | 2 | 2 | 2 |
| 1861 | Merlin OS=Homo sapiens GN=NF2 PE=1 SV=1 | MERL_HUMAN | 70 kDa | TRUE | 3.90% | 2 | 2 | 2 |
| 1862 | Quinone oxidoreductase-like protein 1 OS=Homo sapiens GN=CRYZL1 PE=1 SV=2 | QORL1_HUMAN | 39 kDa | TRUE | 7.70% | 2 | 2 | 2 |
| 1863 | IQ and ubiquitin-like domain-containing protein OS=Homo sapiens GN=IQUB PE=1 SV=2 | IQUB_HUMAN | 93 kDa |  | 2.00% | 2 | 2 | 2 |
| 1864 | E3 ubiquitin-protein ligase BRE1B OS=Homo sapiens GN=RNF40 PE=1 SV=4 | BRE1B_HUMAN | 114 kDa |  | 1.70% | 2 | 2 | 2 |
| 1865 | PHD finger-like domain-containing protein 5A OS=Homo sapiens GN=PHF5A PE=1 SV=1 | PHF5A_HUMAN | 12 kDa |  | 14% | 2 | 2 | 2 |
| END OF FILE | | | | | | | | |
