# Supplementary material for: Proteomic Analysis of Exudates from Chronic Ulcer of Diabetic Foot Treated with Scorpion Antimicrobial Peptide
Source: Mediators Inflamm. 2022 Oct 3;2022:5852786. doi: 10.1155/2022/5852786 (PMC9550419; doi:10.1155/2022/5852786)
Supplement: Supplementary Materials — Bacteriological identification of diabetic foot ulcer wounds is available on Supplementary Table 1–3. Identification results by mass spectrometry is available on Supplementary Table 4; analysis of proteins in diabetic wound exudate by iTRAQ is available on Supplementary Table 5; IPA technology for the annotation of differential proteins is available on Supplementary Table 6; classical signal pathway analysis of differential proteins is available on Supplementary Table 7; analysis of upstream regulatory factors is available on Supplementary Table 8; analysis of possible interaction networks in differential proteins is available on Supplementary Table 9. [file 5852786.f1.zip › Supplementary Table 5.docx]

Supplementary Table 5 Analysis of proteins in diabetic wound exudate by iTRAQ

| No. | Identified Proteins (1865/1893) | Accession | Molecular Weight | Protein Grouping Ambiguity | Friedman Test (p-value) Benjamini-Hochberg (p< 0.03067) | B-A | C-A | D-A | Serum-1 Normalized Intensity | Std Dev(Serum-1) | Serum-2 | Std Dev(Serum-2) | B-1 Normalized Intensity | Std Dev(B-1) | B-1 Fold Change Ratio | B-2 Normalized Intensity | Std Dev(B-2) | B-2 Fold Change Ratio | C-1 Normalized Intensity | Std Dev(C-1) | C-1 Fold Change Ratio | C-2 Normalized Intensity | Std Dev(C-2) | C-2 Fold Change Ratio | D-1 Normalized Intensity | Std Dev(D-1) | D-1 Fold Change Ratio | D-2 Normalized Intensity | Std Dev(D-2) | D-2 Fold Change Ratio |
| --- | --- | --- | --- | --- | --- | --- | --- | --- | --- | --- | --- | --- | --- | --- | --- | --- | --- | --- | --- | --- | --- | --- | --- | --- | --- | --- | --- | --- | --- | --- |
| 6 | Fibrinogen alpha chain OS=Homo sapiens GN=FGA PE=1 SV=2 | FIBA_HUMAN | 95 kDa | TRUE | 0.0001 | -5.00 | -5.00 | -5.00 | 19.68 | 1.399482 | 19.69 | 1.43589 | 17.59 | 0.643611 | 0.2 | 17.34 | 0.660901 | 0.2 | 17.32 | 0.587241 | 0.2 | 17.41 | 0.599582 | 0.2 | 17.27 | 0.598326 | 0.2 | 16.94 | 0.74573 | 0.2 |
| 9 | Fibrinogen gamma chain OS=Homo sapiens GN=FGG PE=1 SV=3 | FIBG_HUMAN | 52 kDa |  | 0.0001 | -2.86 | -3.33 | -5.00 | 19.5 | 1.270136 | 19.68 | 1.262415 | 18.12 | 0.762718 | 0.4 | 18.05 | 0.77846 | 0.3 | 17.82 | 0.673411 | 0.3 | 17.9 | 0.644169 | 0.3 | 17.54 | 0.678878 | 0.2 | 17.32 | 0.882728 | 0.2 |
| 4 | Fibrinogen beta chain OS=Homo sapiens GN=FGB PE=1 SV=2 | FIBB_HUMAN | 56 kDa | TRUE | 0.0001 | -3.33 | -3.33 | -4.00 | 19.37 | 1.095925 | 19.51 | 1.041534 | 17.79 | 0.655113 | 0.3 | 17.66 | 0.693773 | 0.3 | 17.8 | 0.644807 | 0.3 | 17.88 | 0.641151 | 0.3 | 17.44 | 0.653345 | 0.3 | 17.33 | 0.753769 | 0.2 |
| 328 | Cadherin-5 OS=Homo sapiens GN=CDH5 PE=1 SV=5 | CADH5_HUMAN | 88 kDa |  | 0.0001 | -3.33 | -4.00 | -3.33 | 20.03 | 1.003002 | 19.83 | 0.961533 | 18.39 | 0.627953 | 0.3 | 18.2 | 0.58379 | 0.3 | 18.02 | 0.553766 | 0.2 | 18.14 | 0.55639 | 0.3 | 18.08 | 0.537139 | 0.3 | 17.99 | 0.578607 | 0.3 |
| 1442 | Collectin-10 OS=Homo sapiens GN=COLEC10 PE=2 SV=2 | COL10_HUMAN | 31 kDa |  | 0.00094 | -1.67 | -2.86 | -3.33 | 18.93 | 0.499714 | 18.96 | 0.49194 | 18.19 | 0.498481 | 0.6 | 18.12 | 0.505664 | 0.6 | 17.29 | 0.535545 | 0.3 | 17.45 | 0.500871 | 0.4 | 17.28 | 0.523985 | 0.3 | 17.19 | 0.494627 | 0.3 |
| 1363 | Asialoglycoprotein receptor 2 OS=Homo sapiens GN=ASGR2 PE=1 SV=2 | ASGR2_HUMAN | 35 kDa |  | 0.00041 | -2.22 | -2.86 | -3.33 | 19.51 | 0.545404 | 19.34 | 0.535694 | 18.34 | 0.512858 | 0.4 | 18.24 | 0.533477 | 0.5 | 17.97 | 0.538172 | 0.3 | 17.95 | 0.510297 | 0.4 | 17.88 | 0.515816 | 0.3 | 17.81 | 0.565271 | 0.3 |
| 1193 | Mannose-binding protein C OS=Homo sapiens GN=MBL2 PE=1 SV=2 | MBL2_HUMAN | 26 kDa |  | 0.00013 | -1.54 | -2.50 | -2.86 | 19.03 | 0.603008 | 19.12 | 0.600512 | 18.48 | 0.586246 | 0.7 | 18.45 | 0.56704 | 0.6 | 17.71 | 0.5352 | 0.4 | 17.81 | 0.535718 | 0.4 | 17.81 | 0.524869 | 0.4 | 17.46 | 0.659223 | 0.3 |
| 903 | Fibrinogen-like protein 1 OS=Homo sapiens GN=FGL1 PE=1 SV=3 | FGL1_HUMAN | 36 kDa |  | 0.00026 | -2.50 | -2.50 | -2.86 | 19.44 | 0.655448 | 19.46 | 0.673571 | 18.28 | 0.523267 | 0.4 | 18.26 | 0.556488 | 0.4 | 17.99 | 0.588269 | 0.4 | 18.18 | 0.558214 | 0.4 | 17.99 | 0.564893 | 0.4 | 17.64 | 0.767724 | 0.3 |
| 65 | Serum amyloid A-1 protein OS=Homo sapiens GN=SAA1 PE=1 SV=1 | SAA1_HUMAN | 14 kDa | TRUE | 0.0022 | 1.15 | -1.05 | -2.86 | 17.94 | 0.667893 | 17.85 | 0.672309 | 18.14 | 0.681413 | 1.1 | 18.13 | 0.705906 | 1.2 | 17.93 | 0.617635 | 1 | 17.82 | 0.748506 | 0.9 | 16.68 | 0.912217 | 0.4 | 16.31 | 1.060573 | 0.3 |
| 218 | Lipopolysaccharide-binding protein OS=Homo sapiens GN=LBP PE=1 SV=3 | LBP_HUMAN | 53 kDa | TRUE | 0.0001 | -1.54 | -2.50 | -2.50 | 18.87 | 0.698009 | 18.89 | 0.626968 | 18.24 | 0.588518 | 0.7 | 18.19 | 0.564705 | 0.6 | 17.69 | 0.627648 | 0.4 | 17.76 | 0.623656 | 0.4 | 17.7 | 0.599778 | 0.4 | 17.74 | 0.671477 | 0.4 |
| 1119 | Collectin-11 OS=Homo sapiens GN=COLEC11 PE=1 SV=1 | COL11_HUMAN | 29 kDa |  | 0.0001 | -1.67 | -2.50 | -2.50 | 18.62 | 0.655568 | 18.63 | 0.682436 | 17.84 | 0.537964 | 0.6 | 17.88 | 0.62362 | 0.6 | 17.29 | 0.514583 | 0.4 | 17.33 | 0.606639 | 0.4 | 17.29 | 0.532474 | 0.4 | 17.16 | 0.62291 | 0.4 |
| 140 | Coagulation factor XIII A chain OS=Homo sapiens GN=F13A1 PE=1 SV=4 | F13A_HUMAN | 83 kDa |  | 0.0001 | -2.00 | -2.50 | -2.50 | 19.6 | 0.752115 | 19.61 | 0.771075 | 18.64 | 0.623967 | 0.5 | 18.52 | 0.625123 | 0.5 | 18.42 | 0.566391 | 0.4 | 18.4 | 0.544265 | 0.4 | 18.37 | 0.547679 | 0.4 | 18.43 | 0.605122 | 0.4 |
| 1039 | Tetranectin OS=Homo sapiens GN=CLEC3B PE=1 SV=3 | TETN_HUMAN | 23 kDa | TRUE | 0.0003 | -2.50 | -2.50 | -2.50 | 19.21 | 0.788308 | 19.11 | 0.841647 | 17.84 | 0.517673 | 0.4 | 17.76 | 0.515629 | 0.4 | 17.76 | 0.506197 | 0.4 | 17.71 | 0.573412 | 0.4 | 17.76 | 0.525622 | 0.4 | 17.75 | 0.510464 | 0.4 |
| 1024 | Serum paraoxonase/lactonase 3 OS=Homo sapiens GN=PON3 PE=1 SV=3 | PON3_HUMAN | 40 kDa |  | 0.0001 | -1.82 | -2.22 | -2.50 | 19.13 | 0.593074 | 18.98 | 0.590484 | 18.25 | 0.518249 | 0.5 | 18.2 | 0.513968 | 0.6 | 17.96 | 0.537057 | 0.4 | 18.02 | 0.51757 | 0.5 | 17.81 | 0.537317 | 0.4 | 17.71 | 0.55616 | 0.4 |
| 854 | A disintegrin and metalloproteinase with thrombospondin motifs 13 OS=Homo sapiens GN=ADAMTS13 PE=1 SV=1 | ATS13_HUMAN | 154 kDa | TRUE | 0.0001 | -1.82 | -2.00 | -2.50 | 18.95 | 0.623572 | 18.91 | 0.554979 | 18.21 | 0.527599 | 0.6 | 18.03 | 0.527288 | 0.5 | 17.83 | 0.509295 | 0.5 | 17.9 | 0.56952 | 0.5 | 17.74 | 0.513213 | 0.4 | 17.69 | 0.556708 | 0.4 |
| 1233 | Insulin-like growth factor-binding protein 5 OS=Homo sapiens GN=IGFBP5 PE=1 SV=1 | IBP5_HUMAN | 31 kDa | TRUE | 0.026 | -2.22 | -2.50 | -2.22 | 20.48 | 0.798486 | 20.48 | 0.827676 | 19.37 | 0.525761 | 0.5 | 19.25 | 0.53732 | 0.4 | 19.16 | 0.524008 | 0.4 | 19.08 | 0.535375 | 0.4 | 19.31 | 0.522034 | 0.4 | 19.34 | 0.527014 | 0.5 |
| 865 | Insulin-like growth factor-binding protein 3 OS=Homo sapiens GN=IGFBP3 PE=1 SV=2 | IBP3_HUMAN | 32 kDa |  | 0.0001 | -1.67 | -2.22 | -2.22 | 19.46 | 0.541319 | 19.28 | 0.584183 | 18.6 | 0.525112 | 0.6 | 18.55 | 0.504801 | 0.6 | 18.28 | 0.504748 | 0.4 | 18.34 | 0.513867 | 0.5 | 18.27 | 0.517837 | 0.4 | 18.33 | 0.513239 | 0.5 |
| 168 | C4b-binding protein beta chain OS=Homo sapiens GN=C4BPB PE=1 SV=1 | C4BPB_HUMAN | 28 kDa |  | 0.0001 | -1.82 | -2.22 | -2.22 | 19.21 | 0.723353 | 19.29 | 0.753776 | 18.39 | 0.65016 | 0.6 | 18.36 | 0.654543 | 0.5 | 18.16 | 0.592968 | 0.5 | 18.14 | 0.601355 | 0.4 | 18.14 | 0.559663 | 0.5 | 18.1 | 0.59932 | 0.4 |
| 26 | C4b-binding protein alpha chain OS=Homo sapiens GN=C4BPA PE=1 SV=2 | C4BPA_HUMAN | 67 kDa |  | 0.0001 | -1.43 | -2.00 | -2.22 | 18.78 | 0.784339 | 18.8 | 0.781361 | 18.17 | 0.809896 | 0.7 | 18.13 | 0.819821 | 0.7 | 17.65 | 0.681025 | 0.5 | 17.72 | 0.647868 | 0.5 | 17.75 | 0.652349 | 0.5 | 17.64 | 0.721582 | 0.4 |
| 891 | Angiopoietin-related protein 3 OS=Homo sapiens GN=ANGPTL3 PE=1 SV=1 | ANGL3_HUMAN | 54 kDa | TRUE | 0.0001 | -1.54 | -2.00 | -2.22 | 18.15 | 0.558363 | 18.22 | 0.620303 | 17.55 | 0.57233 | 0.7 | 17.55 | 0.542185 | 0.6 | 17.14 | 0.53729 | 0.5 | 17.18 | 0.53655 | 0.5 | 17.18 | 0.537558 | 0.5 | 16.94 | 0.583266 | 0.4 |
| 546 | Prenylcysteine oxidase 1 OS=Homo sapiens GN=PCYOX1 PE=1 SV=3 | PCYOX_HUMAN | 57 kDa |  | 0.0022 | -1.67 | -1.82 | -2.22 | 19.49 | 0.777541 | 19.5 | 0.674509 | 18.71 | 0.606926 | 0.6 | 18.65 | 0.594578 | 0.6 | 18.36 | 0.554891 | 0.5 | 18.67 | 0.644359 | 0.6 | 18.28 | 0.557313 | 0.4 | 18.56 | 0.724221 | 0.5 |
| 1505 | Desmoglein-2 OS=Homo sapiens GN=DSG2 PE=1 SV=2 | DSG2_HUMAN | 122 kDa |  | 0.00013 | -2.00 | -1.82 | -2.22 | 19.32 | 0.70104 | 19.22 | 0.684208 | 18.23 | 0.507659 | 0.5 | 18.2 | 0.512805 | 0.5 | 18.2 | 0.601769 | 0.5 | 18.38 | 0.549121 | 0.6 | 18.01 | 0.511059 | 0.4 | 18.07 | 0.512275 | 0.5 |
| 1 | Apolipoprotein B-100 OS=Homo sapiens GN=APOB PE=1 SV=2 | APOB_HUMAN | 516 kDa | TRUE | 0.0001 | -1.67 | -2.00 | -2.00 | 19.03 | 0.766872 | 19.1 | 0.729477 | 18.39 | 0.715277 | 0.6 | 18.35 | 0.729877 | 0.6 | 18.08 | 0.665646 | 0.5 | 18.13 | 0.621779 | 0.5 | 18.04 | 0.603233 | 0.5 | 18 | 0.696913 | 0.5 |
| 1203 | Polymeric immunoglobulin receptor OS=Homo sapiens GN=PIGR PE=1 SV=4 | PIGR_HUMAN | 83 kDa |  | 0.00019 | -1.67 | -2.00 | -2.00 | 19.44 | 0.644811 | 19.48 | 0.559352 | 18.76 | 0.536368 | 0.6 | 18.7 | 0.51858 | 0.6 | 18.5 | 0.528409 | 0.5 | 18.5 | 0.521771 | 0.5 | 18.5 | 0.546042 | 0.5 | 18.39 | 0.529813 | 0.5 |
| 31 | Prothrombin OS=Homo sapiens GN=F2 PE=1 SV=2 | THRB_HUMAN | 70 kDa | TRUE | 0.0001 | -1.82 | -2.00 | -2.00 | 19.32 | 0.897195 | 19.35 | 0.85882 | 18.5 | 0.732354 | 0.6 | 18.43 | 0.73702 | 0.5 | 18.34 | 0.619869 | 0.5 | 18.37 | 0.582692 | 0.5 | 18.48 | 0.582224 | 0.5 | 18.46 | 0.619509 | 0.5 |
| 367 | Coagulation factor XIII B chain OS=Homo sapiens GN=F13B PE=1 SV=3 | F13B_HUMAN | 76 kDa |  | 0.0001 | -2.00 | -2.00 | -2.00 | 18.73 | 0.63732 | 18.68 | 0.789931 | 17.74 | 0.624375 | 0.5 | 17.72 | 0.612269 | 0.5 | 17.69 | 0.516284 | 0.5 | 17.7 | 0.518078 | 0.5 | 17.75 | 0.531281 | 0.5 | 17.77 | 0.563446 | 0.5 |
| 282 | Beta-Ala-His dipeptidase OS=Homo sapiens GN=CNDP1 PE=1 SV=4 | CNDP1_HUMAN | 57 kDa |  | 0.0001 | -1.11 | -1.82 | -2.00 | 18.99 | 0.597221 | 18.93 | 0.604029 | 18.8 | 0.576422 | 0.9 | 18.73 | 0.548721 | 0.9 | 17.98 | 0.562548 | 0.5 | 18.12 | 0.557391 | 0.6 | 17.96 | 0.562775 | 0.5 | 17.93 | 0.694035 | 0.5 |
| 245 | Glutathione peroxidase 3 OS=Homo sapiens GN=GPX3 PE=1 SV=2 | GPX3_HUMAN | 26 kDa |  | 0.0001 | -1.54 | -1.82 | -2.00 | 19.43 | 0.675583 | 19.47 | 0.661159 | 18.85 | 0.755034 | 0.7 | 18.74 | 0.732248 | 0.6 | 18.58 | 0.63501 | 0.6 | 18.57 | 0.598017 | 0.5 | 18.49 | 0.632195 | 0.5 | 18.54 | 0.634252 | 0.5 |
| 555 | Monocyte differentiation antigen CD14 OS=Homo sapiens GN=CD14 PE=1 SV=2 | CD14_HUMAN | 40 kDa |  | 0.0001 | -1.67 | -1.82 | -2.00 | 19.22 | 0.737877 | 19.24 | 0.687169 | 18.57 | 0.59675 | 0.6 | 18.59 | 0.581121 | 0.6 | 18.21 | 0.622142 | 0.5 | 18.43 | 0.581447 | 0.6 | 18.17 | 0.580288 | 0.5 | 18.34 | 0.680256 | 0.5 |
| 1314 | Angiopoietin-related protein 6 OS=Homo sapiens GN=ANGPTL6 PE=1 SV=1 | ANGL6_HUMAN | 52 kDa |  | 0.022 | -1.82 | -1.82 | -2.00 | 19.02 | 0.619514 | 19.01 | 0.656295 | 18.02 | 0.521277 | 0.5 | 18.16 | 0.507588 | 0.6 | 18.05 | 0.49932 | 0.5 | 18.36 | 0.566369 | 0.6 | 18.03 | 0.553034 | 0.5 | 17.95 | 0.53173 | 0.5 |
| 68 | Complement component C9 OS=Homo sapiens GN=C9 PE=1 SV=2 | CO9_HUMAN | 63 kDa | TRUE | 0.0001 | -1.25 | -1.67 | -2.00 | 18.83 | 0.677232 | 18.85 | 0.66803 | 18.48 | 0.675056 | 0.8 | 18.49 | 0.666425 | 0.8 | 18.09 | 0.627156 | 0.6 | 18.16 | 0.642518 | 0.6 | 17.95 | 0.589017 | 0.5 | 17.94 | 0.72009 | 0.5 |
| 287 | Ficolin-2 OS=Homo sapiens GN=FCN2 PE=1 SV=2 | FCN2_HUMAN | 34 kDa | TRUE | 0.0001 | -1.25 | -1.67 | -2.00 | 19.04 | 0.673291 | 18.97 | 0.715498 | 18.56 | 0.675134 | 0.8 | 18.5 | 0.606811 | 0.8 | 18.21 | 0.593636 | 0.6 | 18.23 | 0.599822 | 0.6 | 18.08 | 0.577264 | 0.5 | 17.97 | 0.688584 | 0.5 |
| 5 | Complement C4-B OS=Homo sapiens GN=C4B PE=1 SV=2 | CO4B_HUMAN | 193 kDa | TRUE | 0.00017 | -1.54 | -1.67 | -2.00 | 19.82 | 0.651103 | 19.77 | 0.710576 | 19.25 | 0.609081 | 0.7 | 19.16 | 0.619898 | 0.6 | 19.03 | 0.605482 | 0.6 | 18.96 | 0.576624 | 0.6 | 18.85 | 0.585897 | 0.5 | 18.79 | 0.66352 | 0.5 |
| 99 | Apolipoprotein L1 OS=Homo sapiens GN=APOL1 PE=1 SV=5 | APOL1_HUMAN | 44 kDa |  | 0.0001 | -1.67 | -1.67 | -2.00 | 19.4 | 0.769237 | 19.46 | 0.775105 | 18.75 | 0.674967 | 0.6 | 18.69 | 0.704799 | 0.6 | 18.71 | 0.62836 | 0.6 | 18.66 | 0.59606 | 0.6 | 18.5 | 0.580011 | 0.5 | 18.49 | 0.641839 | 0.5 |
| 534 | Cartilage oligomeric matrix protein OS=Homo sapiens GN=COMP PE=1 SV=2 | COMP_HUMAN | 83 kDa | TRUE | 0.0001 | -1.67 | -1.67 | -2.00 | 18.35 | 0.580224 | 18.32 | 0.59869 | 17.68 | 0.550397 | 0.6 | 17.69 | 0.552659 | 0.6 | 17.5 | 0.560571 | 0.6 | 17.55 | 0.52168 | 0.6 | 17.34 | 0.510533 | 0.5 | 17.31 | 0.540109 | 0.5 |
| 78 | Serum paraoxonase/arylesterase 1 OS=Homo sapiens GN=PON1 PE=1 SV=3 | PON1_HUMAN | 40 kDa |  | 0.0001 | -1.67 | -1.54 | -2.00 | 18.8 | 0.724347 | 18.79 | 0.689972 | 18.15 | 0.624038 | 0.6 | 18.11 | 0.637267 | 0.6 | 18.07 | 0.565757 | 0.6 | 18.18 | 0.614605 | 0.7 | 17.93 | 0.572814 | 0.5 | 17.85 | 0.639464 | 0.5 |
| 608 | Nesprin-1 OS=Homo sapiens GN=SYNE1 PE=1 SV=4 | SYNE1_HUMAN | 1011 kDa | TRUE | 0.026 | -1.43 | -1.43 | -2.00 | 16.8 | 0.483958 | 16.29 | 0.570016 | 15.96 | 0.483398 | 0.6 | 16.02 | 0.488469 | 0.8 | 16.04 | 0.480799 | 0.6 | 15.95 | 0.476082 | 0.8 | 15.56 | 0.479273 | 0.4 | 15.62 | 0.497378 | 0.6 |
| 58 | Complement C1r subcomponent OS=Homo sapiens GN=C1R PE=1 SV=2 | C1R_HUMAN | 80 kDa | TRUE | 0.0001 | -1.67 | -2.00 | -1.82 | 19.49 | 0.641443 | 19.53 | 0.643754 | 18.78 | 0.625025 | 0.6 | 18.76 | 0.629004 | 0.6 | 18.6 | 0.560253 | 0.5 | 18.63 | 0.599247 | 0.5 | 18.65 | 0.552878 | 0.6 | 18.58 | 0.628257 | 0.5 |
| 390 | Carboxypeptidase B2 OS=Homo sapiens GN=CPB2 PE=1 SV=2 | CBPB2_HUMAN | 48 kDa |  | 0.0001 | -1.67 | -2.00 | -1.82 | 19.15 | 0.626095 | 19.13 | 0.617093 | 18.5 | 0.622319 | 0.6 | 18.46 | 0.640902 | 0.6 | 18.24 | 0.544356 | 0.5 | 18.3 | 0.608416 | 0.5 | 18.35 | 0.555273 | 0.6 | 18.28 | 0.658124 | 0.5 |
| 426 | Haptoglobin-related protein OS=Homo sapiens GN=HPR PE=2 SV=2 | HPTR_HUMAN | 39 kDa | TRUE | 0.0001 | -1.25 | -1.82 | -1.82 | 19.27 | 0.675978 | 19.28 | 0.628416 | 18.94 | 0.639803 | 0.8 | 18.91 | 0.65899 | 0.8 | 18.36 | 0.710307 | 0.5 | 18.51 | 0.579755 | 0.6 | 18.42 | 0.627164 | 0.5 | 18.57 | 0.825673 | 0.6 |
| 343 | Protein Z-dependent protease inhibitor OS=Homo sapiens GN=SERPINA10 PE=1 SV=1 | ZPI_HUMAN | 51 kDa |  | 0.0001 | -1.54 | -1.82 | -1.82 | 18.57 | 0.658062 | 18.61 | 0.645481 | 17.97 | 0.516056 | 0.7 | 17.93 | 0.545475 | 0.6 | 17.68 | 0.528481 | 0.5 | 17.75 | 0.535658 | 0.6 | 17.69 | 0.519958 | 0.6 | 17.6 | 0.719359 | 0.5 |
| 1492 | Beta-2-microglobulin OS=Homo sapiens GN=B2M PE=1 SV=1 | B2MG_HUMAN | 14 kDa |  | 0.013 | -1.54 | -1.82 | -1.82 | 19.02 | 0.554262 | 18.7 | 0.598987 | 18.35 | 0.507093 | 0.6 | 18.18 | 0.501626 | 0.7 | 18.14 | 0.502952 | 0.5 | 17.88 | 0.503188 | 0.6 | 18.28 | 0.513278 | 0.6 | 17.63 | 0.545021 | 0.5 |
| 62 | Complement C1s subcomponent OS=Homo sapiens GN=C1S PE=1 SV=1 | C1S_HUMAN | 77 kDa | TRUE | 0.0001 | -1.54 | -1.67 | -1.82 | 19.15 | 0.659244 | 19.2 | 0.639906 | 18.58 | 0.681667 | 0.7 | 18.55 | 0.705023 | 0.6 | 18.35 | 0.617708 | 0.6 | 18.36 | 0.578403 | 0.6 | 18.36 | 0.576492 | 0.6 | 18.34 | 0.709241 | 0.5 |
| 1624 | Protocadherin-12 OS=Homo sapiens GN=PCDH12 PE=1 SV=1 | PCD12_HUMAN | 129 kDa |  | 0.026 | -1.43 | -1.54 | -1.82 | 18.92 | 0.575621 | 18.78 | 0.498395 | 18.35 | 0.51222 | 0.7 | 18.35 | 0.530503 | 0.7 | 18.24 | 0.545526 | 0.6 | 18.34 | 0.50411 | 0.7 | 18.04 | 0.508658 | 0.5 | 18.1 | 0.540929 | 0.6 |
| 1367 | Reelin OS=Homo sapiens GN=RELN PE=1 SV=3 | RELN_HUMAN | 388 kDa |  | 0.0089 | -1.54 | -1.82 | -1.67 | 17.8 | 0.650303 | 18.03 | 0.545256 | 17.19 | 0.526025 | 0.7 | 17.23 | 0.506178 | 0.6 | 17 | 0.522635 | 0.6 | 17.16 | 0.498788 | 0.5 | 17.04 | 0.502358 | 0.6 | 17.19 | 0.582624 | 0.6 |
| 180 | CD5 antigen-like OS=Homo sapiens GN=CD5L PE=1 SV=1 | CD5L_HUMAN | 38 kDa |  | 0.0001 | -1.67 | -1.82 | -1.67 | 18.95 | 0.705607 | 18.97 | 0.6588 | 18.26 | 0.583174 | 0.6 | 18.25 | 0.604481 | 0.6 | 18.13 | 0.551969 | 0.6 | 18.06 | 0.558218 | 0.5 | 18.22 | 0.558071 | 0.6 | 18.17 | 0.57706 | 0.6 |
| 860 | Coagulation factor XII OS=Homo sapiens GN=F12 PE=1 SV=3 | FA12_HUMAN | 68 kDa | TRUE | 0.0001 | -1.67 | -1.82 | -1.67 | 18.92 | 0.629598 | 18.81 | 0.584375 | 18.22 | 0.590653 | 0.6 | 18.16 | 0.613285 | 0.6 | 18.05 | 0.522836 | 0.5 | 18.11 | 0.522819 | 0.6 | 18.15 | 0.520661 | 0.6 | 18.17 | 0.510163 | 0.6 |
| 615 | Adiponectin OS=Homo sapiens GN=ADIPOQ PE=1 SV=1 | ADIPO_HUMAN | 26 kDa |  | 0.00015 | -1.25 | -1.67 | -1.67 | 18.19 | 0.555054 | 18.17 | 0.575281 | 17.77 | 0.647386 | 0.8 | 17.84 | 0.593024 | 0.8 | 17.44 | 0.533614 | 0.6 | 17.52 | 0.533653 | 0.6 | 17.46 | 0.532084 | 0.6 | 17.43 | 0.551481 | 0.6 |
| 126 | Phosphatidylinositol-glycan-specific phospholipase D OS=Homo sapiens GN=GPLD1 PE=1 SV=3 | PHLD_HUMAN | 92 kDa | TRUE | 0.0001 | -1.43 | -1.67 | -1.67 | 19.41 | 0.668032 | 19.39 | 0.685104 | 18.95 | 0.626548 | 0.7 | 18.94 | 0.630122 | 0.7 | 18.68 | 0.610312 | 0.6 | 18.7 | 0.572612 | 0.6 | 18.61 | 0.569463 | 0.6 | 18.61 | 0.656183 | 0.6 |
| 133 | Coagulation factor V OS=Homo sapiens GN=F5 PE=1 SV=4 | FA5_HUMAN | 252 kDa | TRUE | 0.0001 | -1.43 | -1.67 | -1.67 | 18.7 | 0.719677 | 18.65 | 0.731636 | 18.14 | 0.587799 | 0.7 | 18.12 | 0.587397 | 0.7 | 17.98 | 0.526984 | 0.6 | 18.02 | 0.58505 | 0.6 | 17.98 | 0.536244 | 0.6 | 17.92 | 0.606715 | 0.6 |
| 725 | Fetuin-B OS=Homo sapiens GN=FETUB PE=1 SV=2 | FETUB_HUMAN | 42 kDa |  | 0.0001 | -1.43 | -1.67 | -1.67 | 18.73 | 0.630341 | 18.65 | 0.613728 | 18.29 | 0.553381 | 0.7 | 18.28 | 0.563188 | 0.7 | 18.05 | 0.569289 | 0.6 | 18 | 0.542646 | 0.6 | 18.06 | 0.607942 | 0.6 | 18.04 | 0.600977 | 0.6 |
| 276 | Complement factor H-related protein 5 OS=Homo sapiens GN=CFHR5 PE=1 SV=1 | FHR5_HUMAN | 64 kDa | TRUE | 0.0001 | -1.54 | -1.67 | -1.67 | 19.45 | 0.602174 | 19.46 | 0.619239 | 18.86 | 0.559019 | 0.7 | 18.81 | 0.566714 | 0.6 | 18.69 | 0.570941 | 0.6 | 18.76 | 0.59643 | 0.6 | 18.67 | 0.572794 | 0.6 | 18.64 | 0.591757 | 0.6 |
| 17 | Apolipoprotein A-IV OS=Homo sapiens GN=APOA4 PE=1 SV=3 | APOA4_HUMAN | 45 kDa | TRUE | 0.0001 | -1.67 | -1.67 | -1.67 | 19.08 | 0.711007 | 19.12 | 0.684409 | 18.42 | 0.745622 | 0.6 | 18.4 | 0.71967 | 0.6 | 18.44 | 0.624069 | 0.6 | 18.45 | 0.612936 | 0.6 | 18.37 | 0.593226 | 0.6 | 18.34 | 0.631823 | 0.6 |
| 33 | Inter-alpha-trypsin inhibitor heavy chain H4 OS=Homo sapiens GN=ITIH4 PE=1 SV=4 | ITIH4_HUMAN | 103 kDa | TRUE | 0.0001 | -1.67 | -1.67 | -1.67 | 19.06 | 0.754431 | 19.09 | 0.736716 | 18.36 | 0.689113 | 0.6 | 18.34 | 0.68883 | 0.6 | 18.24 | 0.605912 | 0.6 | 18.3 | 0.614588 | 0.6 | 18.31 | 0.578892 | 0.6 | 18.29 | 0.660944 | 0.6 |
| 318 | Protein AMBP OS=Homo sapiens GN=AMBP PE=1 SV=1 | AMBP_HUMAN | 39 kDa |  | 0.00014 | -1.67 | -1.67 | -1.67 | 18.84 | 0.798973 | 18.9 | 0.807581 | 18.07 | 0.642849 | 0.6 | 18.04 | 0.614275 | 0.6 | 18.01 | 0.529522 | 0.6 | 18.1 | 0.593218 | 0.6 | 18.12 | 0.534301 | 0.6 | 18.27 | 0.583646 | 0.6 |
| 1267 | Oncoprotein-induced transcript 3 protein OS=Homo sapiens GN=OIT3 PE=1 SV=2 | OIT3_HUMAN | 60 kDa |  | 0.013 | -1.67 | -1.67 | -1.67 | 19.6 | 0.582669 | 19.59 | 0.561937 | 18.96 | 0.508199 | 0.6 | 18.95 | 0.508199 | 0.6 | 18.84 | 0.509454 | 0.6 | 18.89 | 0.506563 | 0.6 | 18.79 | 0.508472 | 0.6 | 18.9 | 0.509196 | 0.6 |
| 74 | Apolipoprotein D OS=Homo sapiens GN=APOD PE=1 SV=1 | APOD_HUMAN | 21 kDa |  | 0.0001 | -1.18 | -1.54 | -1.67 | 19.58 | 0.644858 | 19.7 | 0.636742 | 19.37 | 0.659404 | 0.9 | 19.36 | 0.67055 | 0.8 | 19.08 | 0.602465 | 0.7 | 19.06 | 0.645973 | 0.6 | 18.96 | 0.650216 | 0.6 | 18.94 | 0.568359 | 0.6 |
| 1717 | Cadherin-11 OS=Homo sapiens GN=CDH11 PE=2 SV=2 | CAD11_HUMAN | 88 kDa |  | 0.0056 | -1.18 | -1.54 | -1.67 | 18.87 | 0.859726 | 18.78 | 0.880046 | 18.58 | 0.523634 | 0.8 | 18.62 | 0.508203 | 0.9 | 18.2 | 0.506427 | 0.6 | 18.4 | 0.503269 | 0.7 | 18.18 | 0.533492 | 0.6 | 18.15 | 0.510888 | 0.6 |
| 264 | Retinol-binding protein 4 OS=Homo sapiens GN=RBP4 PE=1 SV=3 | RET4_HUMAN | 23 kDa | TRUE | 0.00064 | -1.67 | -1.54 | -1.67 | 16.36 | 0.585924 | 16.39 | 0.622884 | 15.62 | 0.541437 | 0.6 | 15.67 | 0.555128 | 0.6 | 15.59 | 0.547702 | 0.6 | 15.78 | 0.607804 | 0.7 | 15.69 | 0.514035 | 0.6 | 15.67 | 0.555159 | 0.6 |
| 436 | Adipocyte plasma membrane-associated protein OS=Homo sapiens GN=APMAP PE=1 SV=2 | APMAP_HUMAN | 46 kDa |  | 0.0001 | 1.00 | -1.43 | -1.67 | 18.79 | 0.569992 | 18.81 | 0.568075 | 18.76 | 0.594741 | 1 | 18.85 | 0.55065 | 1 | 18.33 | 0.564103 | 0.7 | 18.39 | 0.552396 | 0.7 | 18.15 | 0.580231 | 0.6 | 18.11 | 0.672279 | 0.6 |
| 95 | Complement component C8 beta chain OS=Homo sapiens GN=C8B PE=1 SV=3 | CO8B_HUMAN | 67 kDa |  | 0.0001 | -1.11 | -1.43 | -1.67 | 18.31 | 0.640541 | 18.28 | 0.63611 | 18.14 | 0.609126 | 0.9 | 18.15 | 0.618196 | 0.9 | 17.8 | 0.549811 | 0.7 | 17.81 | 0.57269 | 0.7 | 17.68 | 0.567752 | 0.6 | 17.62 | 0.706969 | 0.6 |
| 407 | IgGFc-binding protein OS=Homo sapiens GN=FCGBP PE=1 SV=3 | FCGBP_HUMAN | 572 kDa | TRUE | 0.0001 | -1.11 | -1.43 | -1.67 | 19.05 | 0.640855 | 19.04 | 0.643358 | 18.84 | 0.544119 | 0.9 | 18.84 | 0.556406 | 0.9 | 18.45 | 0.544419 | 0.7 | 18.52 | 0.563546 | 0.7 | 18.39 | 0.566136 | 0.6 | 18.42 | 0.568929 | 0.6 |
| 94 | Vitamin K-dependent protein S OS=Homo sapiens GN=PROS1 PE=1 SV=1 | PROS_HUMAN | 75 kDa |  | 0.0001 | -1.25 | -1.43 | -1.67 | 18.88 | 0.648812 | 18.92 | 0.635296 | 18.59 | 0.678038 | 0.8 | 18.54 | 0.665972 | 0.8 | 18.43 | 0.642242 | 0.7 | 18.31 | 0.611732 | 0.7 | 18.23 | 0.617025 | 0.6 | 18.22 | 0.651162 | 0.6 |
| 164 | Afamin OS=Homo sapiens GN=AFM PE=1 SV=1 | AFAM_HUMAN | 69 kDa | TRUE | 0.0001 | -1.43 | -1.43 | -1.67 | 19.04 | 0.575923 | 19.03 | 0.588323 | 18.51 | 0.582846 | 0.7 | 18.48 | 0.577071 | 0.7 | 18.54 | 0.549251 | 0.7 | 18.44 | 0.551171 | 0.7 | 18.42 | 0.547002 | 0.6 | 18.26 | 0.579585 | 0.6 |
| 53 | Alpha-2-HS-glycoprotein OS=Homo sapiens GN=AHSG PE=1 SV=1 | FETUA_HUMAN | 39 kDa | TRUE | 0.0001 | -1.67 | -1.43 | -1.67 | 18.56 | 0.707185 | 18.58 | 0.72542 | 17.87 | 0.635626 | 0.6 | 17.87 | 0.666268 | 0.6 | 17.98 | 0.587452 | 0.7 | 18.05 | 0.559123 | 0.7 | 17.96 | 0.544096 | 0.6 | 17.86 | 0.656108 | 0.6 |
| 98 | Serum amyloid P-component OS=Homo sapiens GN=APCS PE=1 SV=2 | SAMP_HUMAN | 25 kDa |  | 0.0001 | -1.67 | -1.43 | -1.67 | 19.25 | 0.654279 | 19.24 | 0.676379 | 18.59 | 0.752977 | 0.6 | 18.58 | 0.734734 | 0.6 | 18.7 | 0.650404 | 0.7 | 18.69 | 0.585541 | 0.7 | 18.49 | 0.581674 | 0.6 | 18.49 | 0.588947 | 0.6 |
| 1424 | Out at first protein homolog OS=Homo sapiens GN=OAF PE=2 SV=1 | OAF_HUMAN | 31 kDa |  | 0.024 | -1.18 | -1.33 | -1.67 | 18.59 | 0.585026 | 18.46 | 0.629544 | 18.28 | 0.527056 | 0.8 | 18.32 | 0.511076 | 0.9 | 18.01 | 0.505138 | 0.7 | 18.07 | 0.528316 | 0.8 | 17.98 | 0.506867 | 0.6 | 17.87 | 0.625863 | 0.6 |
| 265 | C-reactive protein OS=Homo sapiens GN=CRP PE=1 SV=1 | CRP_HUMAN | 25 kDa |  | 0.0011 | 1.00 | -1.25 | -1.67 | 18.98 | 0.705316 | 19.05 | 0.66228 | 18.91 | 0.920106 | 1 | 18.98 | 0.918147 | 1 | 18.7 | 0.783673 | 0.8 | 18.68 | 0.612775 | 0.8 | 18.38 | 0.729338 | 0.6 | 18.43 | 0.726543 | 0.6 |
| 586 | Cholesteryl ester transfer protein OS=Homo sapiens GN=CETP PE=1 SV=2 | CETP_HUMAN | 55 kDa |  | 0.00071 | -1.33 | -1.67 | -1.54 | 19.32 | 0.674452 | 19.3 | 0.730903 | 18.91 | 0.582645 | 0.8 | 18.82 | 0.582798 | 0.7 | 18.63 | 0.726508 | 0.6 | 18.57 | 0.684648 | 0.6 | 18.58 | 0.576058 | 0.6 | 18.78 | 0.609967 | 0.7 |
| 7 | Fibronectin OS=Homo sapiens GN=FN1 PE=1 SV=4 | FINC_HUMAN | 263 kDa |  | 0.0001 | -1.67 | -1.67 | -1.54 | 18.57 | 0.663212 | 18.63 | 0.653097 | 17.85 | 0.681748 | 0.6 | 17.83 | 0.670384 | 0.6 | 17.89 | 0.596306 | 0.6 | 17.94 | 0.614344 | 0.6 | 17.96 | 0.556607 | 0.6 | 18.02 | 0.606794 | 0.7 |
| 944 | Coagulation factor VII OS=Homo sapiens GN=F7 PE=1 SV=1 | FA7_HUMAN | 52 kDa | TRUE | 0.0038 | -1.43 | -1.54 | -1.54 | 18.1 | 0.584442 | 18.07 | 0.573901 | 17.7 | 0.583982 | 0.7 | 17.63 | 0.585877 | 0.7 | 17.43 | 0.526069 | 0.6 | 17.63 | 0.519271 | 0.7 | 17.45 | 0.511155 | 0.6 | 17.6 | 0.539976 | 0.7 |
| 24 | Clusterin OS=Homo sapiens GN=CLU PE=1 SV=1 | CLUS_HUMAN | 52 kDa | TRUE | 0.0001 | -1.54 | -1.54 | -1.54 | 18.69 | 0.706056 | 18.66 | 0.736729 | 18.04 | 0.695474 | 0.6 | 18.05 | 0.699871 | 0.7 | 17.99 | 0.656532 | 0.6 | 18.06 | 0.61368 | 0.7 | 18.04 | 0.565332 | 0.6 | 18.03 | 0.648578 | 0.7 |
| 72 | Transthyretin OS=Homo sapiens GN=TTR PE=1 SV=1 | TTHY_HUMAN | 16 kDa |  | 0.0001 | -1.82 | -1.54 | -1.54 | 18.78 | 0.631317 | 18.77 | 0.719476 | 17.9 | 0.766418 | 0.5 | 17.9 | 0.766667 | 0.6 | 18.17 | 0.59725 | 0.7 | 18.16 | 0.568946 | 0.6 | 18.17 | 0.543225 | 0.6 | 18.2 | 0.632608 | 0.7 |
| 344 | Alpha-1-acid glycoprotein 2 OS=Homo sapiens GN=ORM2 PE=1 SV=2 | A1AG2_HUMAN | 24 kDa | TRUE | 0.001 | -1.25 | -1.43 | -1.54 | 18.4 | 0.620436 | 18.43 | 0.634709 | 18.09 | 0.599993 | 0.8 | 18.07 | 0.587299 | 0.8 | 18 | 0.562211 | 0.7 | 17.89 | 0.53764 | 0.7 | 17.87 | 0.524367 | 0.7 | 17.66 | 0.556044 | 0.6 |
| 57 | Serum amyloid A-4 protein OS=Homo sapiens GN=SAA4 PE=1 SV=2 | SAA4_HUMAN | 15 kDa |  | 0.00021 | -1.54 | -1.43 | -1.54 | 19.25 | 0.773966 | 19.55 | 0.751514 | 18.78 | 0.794358 | 0.7 | 18.77 | 0.800956 | 0.6 | 18.76 | 0.793924 | 0.7 | 18.99 | 0.681203 | 0.7 | 18.71 | 0.601591 | 0.7 | 18.87 | 0.712038 | 0.6 |
| 536 | Complement factor H-related protein 3 OS=Homo sapiens GN=CFHR3 PE=1 SV=2 | FHR3_HUMAN | 37 kDa | TRUE | 0.0001 | -1.25 | -1.33 | -1.54 | 18.58 | 0.590165 | 18.54 | 0.587567 | 18.33 | 0.629354 | 0.8 | 18.27 | 0.594537 | 0.8 | 18.24 | 0.643973 | 0.8 | 18.11 | 0.561106 | 0.7 | 18.03 | 0.53119 | 0.7 | 17.85 | 0.572731 | 0.6 |
| 1081 | Glutamine-dependent NAD(+) synthetase OS=Homo sapiens GN=NADSYN1 PE=1 SV=3 | NADE_HUMAN | 79 kDa |  | 0.0038 | -1.43 | -1.25 | -1.54 | 18.1 | 0.550901 | 17.96 | 0.555911 | 17.44 | 0.521453 | 0.6 | 17.56 | 0.525058 | 0.8 | 17.54 | 0.535279 | 0.7 | 17.76 | 0.554216 | 0.9 | 17.46 | 0.518023 | 0.6 | 17.52 | 0.549243 | 0.7 |
| 1213 | Tubulin beta-1 chain OS=Homo sapiens GN=TUBB1 PE=1 SV=1 | TBB1_HUMAN | 50 kDa | TRUE | 0.0016 | -1.05 | -1.11 | -1.54 | 18.54 | 0.629547 | 18.33 | 0.593194 | 18.4 | 0.536613 | 0.9 | 18.33 | 0.542544 | 1 | 18.13 | 0.54472 | 0.8 | 18.28 | 0.564151 | 1 | 17.78 | 0.525557 | 0.6 | 17.82 | 0.621109 | 0.7 |
| 866 | Keratin, type II cytoskeletal 2 epidermal OS=Homo sapiens GN=KRT2 PE=1 SV=2 | K22E_HUMAN | 65 kDa | TRUE | 0.0001 | -1.67 | 1.00 | -1.54 | 18.27 | 0.52736 | 18.24 | 0.565064 | 17.49 | 0.560666 | 0.6 | 17.58 | 0.563098 | 0.6 | 18.25 | 0.539889 | 1 | 18.3 | 0.607587 | 1 | 17.64 | 0.522365 | 0.6 | 17.7 | 0.519594 | 0.7 |
| 1597 | 3-ketoacyl-CoA thiolase, mitochondrial OS=Homo sapiens GN=ACAA2 PE=1 SV=2 | THIM_HUMAN | 42 kDa |  | 0.019 | 2.45 | 1.70 | -1.54 | 18.28 | 0.529912 | 17.7 | 0.543703 | 19.89 | 1.37853 | 3.1 | 18.52 | 0.944252 | 1.8 | 19.3 | 1.83548 | 2 | 18.19 | 0.541783 | 1.4 | 17.93 | 0.569644 | 0.8 | 16.75 | 1.04164 | 0.5 |
| 748 | Apolipoprotein C-IV OS=Homo sapiens GN=APOC4 PE=1 SV=1 | APOC4_HUMAN | 15 kDa |  | 0.0001 | -2.00 | -2.00 | -1.43 | 19.5 | 0.643259 | 19.47 | 0.59945 | 18.44 | 0.689217 | 0.5 | 18.45 | 0.789029 | 0.5 | 18.59 | 0.597515 | 0.5 | 18.59 | 0.595726 | 0.5 | 18.91 | 0.536224 | 0.7 | 18.88 | 0.638013 | 0.7 |
| 533 | Complement C4-A OS=Homo sapiens GN=C4A PE=1 SV=2 | CO4A_HUMAN | 193 kDa | TRUE | 0.024 | -1.18 | -1.82 | -1.43 | 19.02 | 0.655093 | 19.32 | 0.592397 | 18.92 | 0.529929 | 0.9 | 19.03 | 0.591666 | 0.8 | 18.3 | 1.040657 | 0.5 | 18.48 | 0.666096 | 0.6 | 18.48 | 0.558821 | 0.7 | 18.75 | 1.028856 | 0.7 |
| 1242 | Apolipoprotein(a) OS=Homo sapiens GN=LPA PE=1 SV=1 | APOA_HUMAN | 501 kDa |  | 0.00049 | -1.67 | -1.67 | -1.43 | 19.78 | 0.849343 | 19.86 | 0.76728 | 19.06 | 0.553974 | 0.6 | 19.06 | 0.551516 | 0.6 | 19.14 | 0.534062 | 0.6 | 19.23 | 0.636818 | 0.6 | 19.29 | 0.519561 | 0.7 | 19.33 | 0.529768 | 0.7 |
| 1534 | Tsukushin OS=Homo sapiens GN=TSKU PE=2 SV=3 | TSK_HUMAN | 38 kDa | TRUE | 0.0089 | -2.00 | -1.67 | -1.43 | 18.74 | 0.565859 | 18.74 | 0.528385 | 17.88 | 0.63405 | 0.5 | 17.79 | 0.57706 | 0.5 | 18 | 0.493801 | 0.6 | 18.03 | 0.495699 | 0.6 | 17.97 | 0.496451 | 0.6 | 18.35 | 0.618501 | 0.8 |
| 1585 | Angiogenin OS=Homo sapiens GN=ANG PE=1 SV=1 | ANGI_HUMAN | 17 kDa |  | 0.019 | -1.82 | -1.54 | -1.43 | 18.45 | 0.5088 | 18.48 | 0.576837 | 17.67 | 0.503568 | 0.6 | 17.43 | 0.53897 | 0.5 | 17.98 | 0.632981 | 0.7 | 17.84 | 0.503556 | 0.6 | 18.01 | 0.492305 | 0.7 | 17.88 | 0.504212 | 0.7 |
| 115 | Ficolin-3 OS=Homo sapiens GN=FCN3 PE=1 SV=2 | FCN3_HUMAN | 33 kDa | TRUE | 0.0001 | -1.25 | -1.43 | -1.43 | 19.34 | 0.700222 | 19.2 | 0.625862 | 18.97 | 0.740912 | 0.8 | 18.92 | 0.713285 | 0.8 | 18.81 | 0.57524 | 0.7 | 18.79 | 0.626939 | 0.7 | 18.81 | 0.581176 | 0.7 | 18.71 | 0.679285 | 0.7 |
| 120 | Plasma kallikrein OS=Homo sapiens GN=KLKB1 PE=1 SV=1 | KLKB1_HUMAN | 71 kDa |  | 0.0001 | -1.25 | -1.43 | -1.43 | 19.24 | 0.585012 | 19.2 | 0.592708 | 18.83 | 0.562157 | 0.8 | 18.81 | 0.555945 | 0.8 | 18.7 | 0.535945 | 0.7 | 18.63 | 0.568116 | 0.7 | 18.71 | 0.524011 | 0.7 | 18.68 | 0.560602 | 0.7 |
| 2 | Complement C3 OS=Homo sapiens GN=C3 PE=1 SV=2 | CO3_HUMAN | 187 kDa | TRUE | 0.0001 | -1.33 | -1.43 | -1.43 | 18.43 | 0.738031 | 18.53 | 0.698992 | 18.06 | 0.732962 | 0.8 | 18.04 | 0.755044 | 0.7 | 17.97 | 0.65054 | 0.7 | 17.99 | 0.618292 | 0.7 | 17.95 | 0.612357 | 0.7 | 17.95 | 0.689537 | 0.7 |
| 34 | Plasminogen OS=Homo sapiens GN=PLG PE=1 SV=2 | PLMN_HUMAN | 91 kDa | TRUE | 0.0001 | -1.33 | -1.43 | -1.43 | 18.7 | 0.652983 | 18.68 | 0.658666 | 18.27 | 0.740328 | 0.8 | 18.25 | 0.716309 | 0.7 | 18.19 | 0.617147 | 0.7 | 18.14 | 0.592593 | 0.7 | 18.21 | 0.566156 | 0.7 | 18.12 | 0.620673 | 0.7 |
| 482 | Coagulation factor XI OS=Homo sapiens GN=F11 PE=1 SV=1 | FA11_HUMAN | 70 kDa |  | 0.0001 | -1.33 | -1.43 | -1.43 | 18.82 | 0.546115 | 18.76 | 0.595822 | 18.38 | 0.57639 | 0.7 | 18.37 | 0.595195 | 0.8 | 18.33 | 0.568712 | 0.7 | 18.34 | 0.538437 | 0.7 | 18.28 | 0.534232 | 0.7 | 18.26 | 0.64875 | 0.7 |
| 500 | Immunoglobulin J chain OS=Homo sapiens GN=JCHAIN PE=1 SV=4 | IGJ_HUMAN | 18 kDa |  | 0.0042 | -1.33 | -1.43 | -1.43 | 19.47 | 0.758756 | 19.49 | 0.781722 | 19.09 | 0.765892 | 0.8 | 19.05 | 0.705903 | 0.7 | 18.94 | 0.552598 | 0.7 | 18.99 | 0.553541 | 0.7 | 18.99 | 0.557733 | 0.7 | 18.94 | 0.588634 | 0.7 |
| 809 | Ig heavy chain V-III region TIL OS=Homo sapiens PE=1 SV=1 | HV304_HUMAN | 12 kDa | TRUE | 0.011 | -1.33 | -1.43 | -1.43 | 18.94 | 0.578414 | 18.83 | 0.561533 | 18.53 | 0.533501 | 0.8 | 18.41 | 0.552738 | 0.7 | 18.34 | 0.510837 | 0.7 | 18.34 | 0.520189 | 0.7 | 18.37 | 0.522068 | 0.7 | 18.3 | 0.526137 | 0.7 |
| 487 | Ig mu heavy chain disease protein OS=Homo sapiens PE=1 SV=1 | MUCB_HUMAN | 43 kDa | TRUE | 0.0019 | -1.43 | -1.43 | -1.43 | 18.62 | 0.548327 | 18.72 | 0.542854 | 18.16 | 0.543183 | 0.7 | 18.2 | 0.560515 | 0.7 | 18.07 | 0.554873 | 0.7 | 18.19 | 0.553883 | 0.7 | 18.17 | 0.562138 | 0.7 | 18.12 | 0.549882 | 0.7 |
| 36 | Vitronectin OS=Homo sapiens GN=VTN PE=1 SV=1 | VTNC_HUMAN | 54 kDa | TRUE | 0.0001 | -1.54 | -1.43 | -1.43 | 18.67 | 0.637709 | 18.75 | 0.607558 | 18.12 | 0.723619 | 0.7 | 18.11 | 0.695381 | 0.6 | 18.13 | 0.617297 | 0.7 | 18.16 | 0.572041 | 0.7 | 18.18 | 0.565537 | 0.7 | 18.26 | 0.639141 | 0.7 |
| 134 | von Willebrand factor OS=Homo sapiens GN=VWF PE=1 SV=4 | VWF_HUMAN | 309 kDa | TRUE | 0.0001 | -1.25 | -1.33 | -1.43 | 18.7 | 0.63903 | 18.63 | 0.655599 | 18.31 | 0.572295 | 0.8 | 18.31 | 0.556075 | 0.8 | 18.16 | 0.56565 | 0.7 | 18.27 | 0.559553 | 0.8 | 18.14 | 0.530589 | 0.7 | 18.21 | 0.578058 | 0.7 |
| 271 | Extracellular matrix protein 1 OS=Homo sapiens GN=ECM1 PE=1 SV=2 | ECM1_HUMAN | 61 kDa |  | 0.0001 | -1.33 | -1.33 | -1.43 | 19.11 | 0.605597 | 19.07 | 0.602847 | 18.62 | 0.71866 | 0.7 | 18.71 | 0.608064 | 0.8 | 18.52 | 0.590894 | 0.7 | 18.68 | 0.676687 | 0.8 | 18.51 | 0.539288 | 0.7 | 18.48 | 0.596133 | 0.7 |
| 325 | Ig heavy chain V-III region BUT OS=Homo sapiens PE=1 SV=1 | HV306_HUMAN | 12 kDa | TRUE | 0.0012 | -1.54 | -1.33 | -1.43 | 18.61 | 0.596157 | 18.61 | 0.597003 | 18.01 | 0.568319 | 0.7 | 17.99 | 0.572021 | 0.6 | 18.19 | 0.529033 | 0.7 | 18.22 | 0.507844 | 0.8 | 18.14 | 0.509582 | 0.7 | 18.11 | 0.506652 | 0.7 |
| 136 | Keratin, type II cytoskeletal 1 OS=Homo sapiens GN=KRT1 PE=1 SV=6 | K2C1_HUMAN | 66 kDa | TRUE | 0.0001 | -2.00 | -1.33 | -1.43 | 18.59 | 0.669711 | 18.57 | 0.641657 | 17.72 | 0.627023 | 0.5 | 17.7 | 0.607563 | 0.5 | 18.22 | 0.582826 | 0.8 | 18.15 | 0.54661 | 0.7 | 18.09 | 0.556742 | 0.7 | 18.08 | 0.572405 | 0.7 |
| 1705 | Quinone oxidoreductase PIG3 OS=Homo sapiens GN=TP53I3 PE=1 SV=2 | QORX_HUMAN | 36 kDa |  | 0.011 | -2.00 | -1.33 | -1.43 | 18.91 | 0.660453 | 18.44 | 0.498037 | 17.75 | 0.493329 | 0.4 | 17.7 | 0.494338 | 0.6 | 18.11 | 0.4921 | 0.6 | 18.2 | 0.494669 | 0.9 | 18.26 | 0.506795 | 0.6 | 18.14 | 0.511366 | 0.8 |
| 1102 | Complement C1r subcomponent-like protein OS=Homo sapiens GN=C1RL PE=1 SV=2 | C1RL_HUMAN | 53 kDa | TRUE | 0.014 | -2.00 | -1.33 | -1.43 | 18.88 | 0.799285 | 18.71 | 0.65534 | 17.82 | 0.645286 | 0.5 | 17.79 | 0.623218 | 0.5 | 18.16 | 0.517524 | 0.6 | 18.51 | 0.609506 | 0.9 | 18.1 | 0.522966 | 0.6 | 18.28 | 0.622616 | 0.8 |
| 365 | Insulin-like growth factor-binding protein complex acid labile subunit OS=Homo sapiens GN=IGFALS PE=1 SV=1 | ALS_HUMAN | 66 kDa |  | 0.0001 | -1.25 | -1.25 | -1.43 | 18.62 | 0.569127 | 18.59 | 0.586626 | 18.23 | 0.522148 | 0.8 | 18.2 | 0.526843 | 0.8 | 18.19 | 0.530946 | 0.7 | 18.35 | 0.583018 | 0.9 | 18.05 | 0.518126 | 0.7 | 18.13 | 0.657984 | 0.7 |
| 1476 | Anthrax toxin receptor 1 OS=Homo sapiens GN=ANTXR1 PE=1 SV=2 | ANTR1_HUMAN | 63 kDa | TRUE | 0.026 | -1.18 | -1.18 | -1.43 | 17.88 | 0.51512 | 17.81 | 0.540474 | 17.57 | 0.487641 | 0.8 | 17.65 | 0.497026 | 0.9 | 17.47 | 0.501555 | 0.8 | 17.68 | 0.497192 | 0.9 | 17.31 | 0.487737 | 0.7 | 17.33 | 0.509495 | 0.7 |
| 1504 | Corticosteroid-binding globulin OS=Homo sapiens GN=SERPINA6 PE=1 SV=1 | CBG_HUMAN | 45 kDa |  | 0.012 | -1.33 | -1.54 | -1.33 | 18.94 | 0.726769 | 18.83 | 0.575276 | 18.48 | 0.502236 | 0.7 | 18.45 | 0.503622 | 0.8 | 18.28 | 0.514017 | 0.6 | 18.3 | 0.513867 | 0.7 | 18.41 | 0.518199 | 0.7 | 18.54 | 0.543515 | 0.8 |
| 119 | Fibulin-1 OS=Homo sapiens GN=FBLN1 PE=1 SV=4 | FBLN1_HUMAN | 77 kDa | TRUE | 0.0001 | -1.25 | -1.43 | -1.33 | 18.78 | 0.728132 | 18.78 | 0.698944 | 18.47 | 0.595236 | 0.8 | 18.43 | 0.578854 | 0.8 | 18.22 | 0.555518 | 0.7 | 18.27 | 0.579741 | 0.7 | 18.38 | 0.531552 | 0.7 | 18.39 | 0.585936 | 0.8 |
| 1118 | Chromogranin-A OS=Homo sapiens GN=CHGA PE=1 SV=7 | CMGA_HUMAN | 51 kDa | TRUE | 0.0056 | -1.43 | -1.43 | -1.33 | 17.87 | 0.599291 | 17.79 | 0.633661 | 17.28 | 0.514286 | 0.7 | 17.24 | 0.509941 | 0.7 | 17.31 | 0.531938 | 0.7 | 17.28 | 0.503273 | 0.7 | 17.36 | 0.531072 | 0.7 | 17.41 | 0.624944 | 0.8 |
| 1304 | FK506-binding protein 15 OS=Homo sapiens GN=FKBP15 PE=1 SV=2 | FKB15_HUMAN | 134 kDa | TRUE | 0.0067 | -1.54 | -1.43 | -1.33 | 18.46 | 0.626876 | 18.74 | 0.617475 | 17.91 | 0.536361 | 0.7 | 18.09 | 0.604485 | 0.6 | 18.1 | 0.498893 | 0.8 | 18.1 | 0.503911 | 0.6 | 18.19 | 0.504043 | 0.8 | 18.19 | 0.497657 | 0.7 |
| 1281 | Suprabasin OS=Homo sapiens GN=SBSN PE=1 SV=2 | SBSN_HUMAN | 61 kDa |  | 0.0056 | -2.50 | -1.43 | -1.33 | 17.37 | 0.85482 | 17.25 | 0.667917 | 15.99 | 1.227767 | 0.4 | 16 | 1.33627 | 0.4 | 16.81 | 0.513997 | 0.7 | 16.74 | 0.684911 | 0.7 | 16.94 | 0.510154 | 0.7 | 16.99 | 0.502047 | 0.8 |
| 1086 | Platelet-activating factor acetylhydrolase OS=Homo sapiens GN=PLA2G7 PE=1 SV=1 | PAFA_HUMAN | 50 kDa | TRUE | 0.00015 | -1.05 | -1.33 | -1.33 | 18.45 | 0.642723 | 18.57 | 0.633876 | 18.46 | 0.504995 | 1 | 18.5 | 0.520425 | 0.9 | 18.13 | 0.512589 | 0.8 | 18.12 | 0.528511 | 0.7 | 18.09 | 0.54557 | 0.8 | 17.97 | 0.514608 | 0.7 |
| 1625 | Nuclear inhibitor of protein phosphatase 1 OS=Homo sapiens GN=PPP1R8 PE=1 SV=2 | PP1R8_HUMAN | 38 kDa |  | 0.0051 | -1.54 | -1.33 | -1.33 | 16.98 | 0.754079 | 16.87 | 0.534222 | 16.25 | 0.496698 | 0.6 | 16.34 | 0.485837 | 0.7 | 16.46 | 0.486336 | 0.7 | 16.53 | 0.515729 | 0.8 | 16.52 | 0.484103 | 0.7 | 16.53 | 0.484164 | 0.8 |
| 220 | Keratin, type I cytoskeletal 9 OS=Homo sapiens GN=KRT9 PE=1 SV=3 | K1C9_HUMAN | 62 kDa | TRUE | 0.0001 | -1.67 | -1.25 | -1.33 | 18.63 | 0.656914 | 18.56 | 0.637808 | 17.96 | 0.659202 | 0.6 | 17.92 | 0.661108 | 0.6 | 18.26 | 0.572453 | 0.8 | 18.2 | 0.521974 | 0.8 | 18.16 | 0.554914 | 0.7 | 18.24 | 0.570279 | 0.8 |
| 1769 | DNA dC->dU-editing enzyme APOBEC-3A OS=Homo sapiens GN=APOBEC3A PE=1 SV=3 | ABC3A_HUMAN | 23 kDa |  | 0.019 | -1.67 | -1.25 | -1.33 | 19.04 | 0.507573 | 18.88 | 0.496772 | 18.27 | 0.49761 | 0.6 | 18.26 | 0.498785 | 0.6 | 18.43 | 0.497334 | 0.7 | 18.73 | 0.525673 | 0.9 | 18.5 | 0.504716 | 0.7 | 18.5 | 0.503933 | 0.8 |
| 3 | Apolipoprotein A-I OS=Homo sapiens GN=APOA1 PE=1 SV=1 | APOA1_HUMAN | 31 kDa | TRUE | 0.0001 | -1.54 | -1.18 | -1.33 | 18.42 | 0.798293 | 18.51 | 0.752783 | 17.87 | 0.931588 | 0.7 | 17.85 | 0.938901 | 0.6 | 18.28 | 0.693502 | 0.9 | 18.24 | 0.650781 | 0.8 | 18.19 | 0.621569 | 0.8 | 18.15 | 0.637057 | 0.7 |
| 1501 | Retroviral-like aspartic protease 1 OS=Homo sapiens GN=ASPRV1 PE=1 SV=1 | APRV1_HUMAN | 37 kDa | TRUE | 0.0056 | -1.67 | -1.54 | -1.25 | 16.08 | 0.508002 | 15.93 | 0.56591 | 15.33 | 0.629026 | 0.6 | 15.29 | 0.658859 | 0.6 | 15.41 | 0.536804 | 0.6 | 15.45 | 0.641306 | 0.7 | 15.78 | 0.508631 | 0.8 | 15.66 | 0.603886 | 0.8 |
| 1644 | Golgi resident protein GCP60 OS=Homo sapiens GN=ACBD3 PE=1 SV=4 | GCP60_HUMAN | 61 kDa |  | 0.011 | -1.67 | -1.33 | -1.25 | 17.66 | 0.56283 | 17.77 | 0.511916 | 16.9 | 0.535985 | 0.6 | 16.99 | 0.53882 | 0.6 | 17.06 | 0.509932 | 0.7 | 17.41 | 0.50356 | 0.8 | 17.39 | 0.505186 | 0.8 | 17.5 | 0.485804 | 0.8 |
| 437 | Apolipoprotein C-I OS=Homo sapiens GN=APOC1 PE=1 SV=1 | APOC1_HUMAN | 9 kDa | TRUE | 0.0018 | -1.82 | -1.25 | -1.25 | 19.57 | 0.632315 | 19.77 | 0.60733 | 18.82 | 0.854639 | 0.6 | 18.77 | 0.890612 | 0.5 | 19.33 | 0.817553 | 0.9 | 19.19 | 0.642892 | 0.7 | 19.48 | 0.688732 | 0.9 | 19.31 | 0.600613 | 0.7 |
| 1687 | Proteoglycan 4 OS=Homo sapiens GN=PRG4 PE=1 SV=2 | PRG4_HUMAN | 151 kDa |  | 0.019 | -1.43 | -1.43 | -1.18 | 19 | 0.511776 | 18.98 | 0.528406 | 18.44 | 0.500183 | 0.7 | 18.42 | 0.504596 | 0.7 | 18.41 | 0.500533 | 0.7 | 18.48 | 0.520033 | 0.7 | 18.49 | 0.504497 | 0.7 | 18.95 | 0.648333 | 1 |
| 301 | Alpha-1B-glycoprotein OS=Homo sapiens GN=A1BG PE=1 SV=4 | A1BG_HUMAN | 54 kDa |  | 0.0001 | -1.43 | -1.25 | -1.18 | 18.73 | 0.576541 | 18.74 | 0.565025 | 18.22 | 0.535597 | 0.7 | 18.19 | 0.524593 | 0.7 | 18.36 | 0.521187 | 0.8 | 18.38 | 0.524938 | 0.8 | 18.42 | 0.518703 | 0.8 | 18.54 | 0.585196 | 0.9 |
| 49 | Ig alpha-1 chain C region OS=Homo sapiens GN=IGHA1 PE=1 SV=2 | IGHA1_HUMAN | 38 kDa | TRUE | 0.0001 | -1.54 | -1.25 | -1.18 | 18.81 | 0.632247 | 18.92 | 0.623077 | 18.3 | 0.631694 | 0.7 | 18.29 | 0.604591 | 0.6 | 18.68 | 0.553643 | 0.9 | 18.48 | 0.53365 | 0.7 | 18.62 | 0.575281 | 0.9 | 18.58 | 0.564991 | 0.8 |
| 852 | Beta-2-glycoprotein 1 OS=Homo sapiens GN=APOH PE=1 SV=3 | APOH_HUMAN | 38 kDa |  | 0.0001 | -1.67 | -1.18 | -1.18 | 19.27 | 0.59478 | 19.32 | 0.581378 | 18.55 | 0.613932 | 0.6 | 18.63 | 0.637743 | 0.6 | 19.19 | 0.536003 | 0.9 | 18.99 | 0.516644 | 0.8 | 19.14 | 0.53371 | 0.9 | 19.06 | 0.517232 | 0.8 |
| 669 | Desmoplakin OS=Homo sapiens GN=DSP PE=1 SV=3 | DESP_HUMAN | 332 kDa | TRUE | 0.0001 | -2.00 | -1.18 | -1.18 | 18.44 | 0.574758 | 18.49 | 0.601113 | 17.51 | 0.553745 | 0.5 | 17.57 | 0.580945 | 0.5 | 18.31 | 0.537703 | 0.9 | 18.27 | 0.518854 | 0.8 | 18.28 | 0.5513 | 0.9 | 18.22 | 0.597578 | 0.8 |
| 38 | Apolipoprotein E OS=Homo sapiens GN=APOE PE=1 SV=1 | APOE_HUMAN | 36 kDa |  | 0.0001 | -1.67 | -1.25 | -1.11 | 18.99 | 0.684587 | 19.07 | 0.637016 | 18.4 | 0.731704 | 0.6 | 18.4 | 0.741242 | 0.6 | 18.66 | 0.701035 | 0.8 | 18.75 | 0.648848 | 0.8 | 18.82 | 0.63488 | 0.9 | 18.85 | 0.608265 | 0.9 |
| 592 | Glycogenin-1 OS=Homo sapiens GN=GYG1 PE=1 SV=4 | GLYG_HUMAN | 39 kDa |  | 0.0001 | 1.65 | 1.25 | -1.05 | 18.02 | 0.587391 | 18.04 | 0.609673 | 18.78 | 0.779226 | 1.7 | 18.72 | 0.71969 | 1.6 | 18.38 | 0.557067 | 1.3 | 18.31 | 0.534653 | 1.2 | 17.93 | 0.553025 | 1 | 17.95 | 0.535305 | 0.9 |
| 1094 | Protein S100-A7 OS=Homo sapiens GN=S100A7 PE=1 SV=4 | S10A7_HUMAN | 11 kDa |  | 0.0001 | -3.33 | -2.22 | 1.00 | 17.27 | 0.505208 | 17.4 | 0.506741 | 15.53 | 0.600528 | 0.3 | 15.44 | 0.661331 | 0.3 | 16.19 | 0.490995 | 0.5 | 15.99 | 0.518136 | 0.4 | 17.42 | 0.499151 | 1.1 | 17.26 | 0.494115 | 0.9 |
| 489 | Protein-arginine deiminase type-2 OS=Homo sapiens GN=PADI2 PE=1 SV=2 | PADI2_HUMAN | 76 kDa |  | 0.0001 | 1.65 | 1.35 | 1.00 | 17 | 0.697839 | 16.91 | 0.73422 | 17.69 | 0.623943 | 1.6 | 17.69 | 0.603621 | 1.7 | 17.43 | 0.557537 | 1.3 | 17.43 | 0.538568 | 1.4 | 16.92 | 0.536665 | 1 | 16.9 | 0.555648 | 1 |
| 1731 | Protein-glutamine gamma-glutamyltransferase E OS=Homo sapiens GN=TGM3 PE=1 SV=4 | TGM3_HUMAN | 77 kDa |  | 0.0022 | -1.67 | -1.11 | 1.05 | 17.64 | 0.519 | 17.47 | 0.646149 | 16.83 | 0.530365 | 0.6 | 16.75 | 0.576738 | 0.6 | 17.36 | 0.495006 | 0.8 | 17.43 | 0.504049 | 1 | 17.56 | 0.496524 | 1 | 17.64 | 0.513529 | 1.1 |
| 1721 | Coiled-coil domain-containing protein 6 OS=Homo sapiens GN=CCDC6 PE=1 SV=2 | CCDC6_HUMAN | 53 kDa |  | 0.0089 | -1.43 | -1.05 | 1.05 | 17.84 | 0.524824 | 17.84 | 0.508651 | 17.34 | 0.533325 | 0.7 | 17.33 | 0.521488 | 0.7 | 17.77 | 0.521581 | 1 | 17.69 | 0.502311 | 0.9 | 17.71 | 0.505705 | 0.9 | 18.1 | 0.530855 | 1.2 |
| 1108 | Beta-adducin OS=Homo sapiens GN=ADD2 PE=1 SV=3 | ADDB_HUMAN | 81 kDa | TRUE | 0.0022 | 1.25 | 1.50 | 1.05 | 18.1 | 0.642108 | 18.16 | 0.681701 | 18.33 | 0.56715 | 1.2 | 18.55 | 0.517215 | 1.3 | 18.45 | 0.54585 | 1.3 | 18.86 | 0.664586 | 1.7 | 18.01 | 0.506535 | 1 | 18.17 | 0.588323 | 1.1 |
| 1749 | Osteopontin OS=Homo sapiens GN=SPP1 PE=1 SV=1 | OSTP_HUMAN | 35 kDa |  | 0.013 | -1.43 | -1.18 | 1.10 | 17.57 | 0.491552 | 17.6 | 0.501221 | 17.04 | 0.509303 | 0.7 | 17.1 | 0.506105 | 0.7 | 17.2 | 0.488608 | 0.8 | 17.47 | 0.48666 | 0.9 | 17.65 | 0.50165 | 1.1 | 17.69 | 0.507539 | 1.1 |
| 59 | Alpha-1-antichymotrypsin OS=Homo sapiens GN=SERPINA3 PE=1 SV=2 | AACT_HUMAN | 48 kDa |  | 0.0001 | 1.40 | 1.20 | 1.10 | 17.72 | 0.695318 | 17.73 | 0.646256 | 18.15 | 0.801205 | 1.4 | 18.18 | 0.784272 | 1.4 | 17.96 | 0.652501 | 1.2 | 18.01 | 0.581115 | 1.2 | 17.8 | 0.575235 | 1.1 | 17.85 | 0.737836 | 1.1 |
| 1218 | Tumor necrosis factor-inducible gene 6 protein OS=Homo sapiens GN=TNFAIP6 PE=1 SV=2 | TSG6_HUMAN | 31 kDa |  | 0.0032 | 1.55 | 1.30 | 1.10 | 18.68 | 1.049077 | 18.7 | 0.863326 | 19.39 | 0.541232 | 1.6 | 19.35 | 0.55351 | 1.5 | 19.01 | 0.622076 | 1.3 | 19.02 | 0.521194 | 1.3 | 18.8 | 0.555672 | 1.1 | 18.82 | 0.624427 | 1.1 |
| 257 | Ankyrin-1 OS=Homo sapiens GN=ANK1 PE=1 SV=3 | ANK1_HUMAN | 206 kDa |  | 0.0001 | 1.55 | 1.35 | 1.10 | 17.44 | 0.776155 | 17.35 | 0.765605 | 17.97 | 0.664272 | 1.5 | 18 | 0.632154 | 1.6 | 17.75 | 0.581225 | 1.3 | 17.85 | 0.562956 | 1.4 | 17.49 | 0.51663 | 1 | 17.57 | 0.648691 | 1.2 |
| 272 | Keratin, type I cytoskeletal 10 OS=Homo sapiens GN=KRT10 PE=1 SV=6 | K1C10_HUMAN | 59 kDa | TRUE | 0.0001 | -1.43 | 1.35 | 1.10 | 17.89 | 0.65128 | 17.9 | 0.63553 | 17.41 | 0.668797 | 0.7 | 17.42 | 0.731485 | 0.7 | 18.37 | 0.575539 | 1.4 | 18.23 | 0.560175 | 1.3 | 17.97 | 0.545254 | 1.1 | 18 | 0.564133 | 1.1 |
| 1681 | Kallikrein-10 OS=Homo sapiens GN=KLK10 PE=1 SV=3 | KLK10_HUMAN | 30 kDa |  | 0.026 | -1.54 | 1.05 | 1.15 | 18.01 | 0.520603 | 18.13 | 0.4947 | 17.46 | 0.580935 | 0.7 | 17.43 | 0.619705 | 0.6 | 18 | 0.498013 | 1 | 18.21 | 0.492526 | 1.1 | 18.15 | 0.492349 | 1.1 | 18.36 | 0.539071 | 1.2 |
| 335 | Unconventional myosin-If OS=Homo sapiens GN=MYO1F PE=1 SV=3 | MYO1F_HUMAN | 125 kDa | TRUE | 0.0001 | 1.50 | 1.20 | 1.15 | 17.74 | 0.798366 | 17.71 | 0.787618 | 18.23 | 0.572732 | 1.5 | 18.26 | 0.575011 | 1.5 | 17.93 | 0.533764 | 1.1 | 18.07 | 0.552817 | 1.3 | 17.81 | 0.521915 | 1.1 | 17.91 | 0.654249 | 1.2 |
| 869 | Nucleosome assembly protein 1-like 4 OS=Homo sapiens GN=NAP1L4 PE=1 SV=1 | NP1L4_HUMAN | 43 kDa | TRUE | 0.003 | 1.50 | 1.25 | 1.15 | 17.12 | 0.592747 | 17.13 | 0.662482 | 17.66 | 0.541975 | 1.5 | 17.75 | 0.578195 | 1.5 | 17.47 | 0.493728 | 1.3 | 17.44 | 0.520764 | 1.2 | 17.21 | 0.517714 | 1.1 | 17.39 | 0.600221 | 1.2 |
| 386 | 1-phosphatidylinositol 4,5-bisphosphate phosphodiesterase gamma-2 OS=Homo sapiens GN=PLCG2 PE=1 SV=4 | PLCG2_HUMAN | 148 kDa | TRUE | 0.0001 | 1.45 | 1.25 | 1.15 | 18.26 | 0.637764 | 18.18 | 0.729119 | 18.75 | 0.602718 | 1.4 | 18.75 | 0.595489 | 1.5 | 18.49 | 0.537341 | 1.2 | 18.53 | 0.522787 | 1.3 | 18.38 | 0.525543 | 1.1 | 18.42 | 0.538687 | 1.2 |
| 258 | Collagen alpha-1(VI) chain OS=Homo sapiens GN=COL6A1 PE=1 SV=3 | CO6A1_HUMAN | 109 kDa | TRUE | 0.0001 | 1.35 | 1.40 | 1.15 | 17.7 | 0.795971 | 17.59 | 0.782785 | 18.04 | 0.567689 | 1.3 | 18.04 | 0.532471 | 1.4 | 18.1 | 0.520886 | 1.3 | 18.18 | 0.558648 | 1.5 | 17.85 | 0.529393 | 1.1 | 17.88 | 0.548809 | 1.2 |
| 1084 | Olfactomedin-4 OS=Homo sapiens GN=OLFM4 PE=1 SV=1 | OLFM4_HUMAN | 57 kDa |  | 0.0034 | 1.90 | 1.45 | 1.15 | 16.93 | 0.530254 | 17.01 | 0.506224 | 17.81 | 0.99843 | 2 | 17.78 | 1.016955 | 1.8 | 17.55 | 0.545769 | 1.5 | 17.45 | 0.510641 | 1.4 | 17.24 | 0.501479 | 1.2 | 17.13 | 0.752904 | 1.1 |
| 1344 | Ribose-5-phosphate isomerase OS=Homo sapiens GN=RPIA PE=1 SV=3 | RPIA_HUMAN | 33 kDa | TRUE | 0.015 | 1.65 | 1.40 | 1.20 | 17.47 | 0.759046 | 17.09 | 0.745079 | 17.98 | 0.565804 | 1.4 | 17.96 | 0.590051 | 1.9 | 17.74 | 0.496189 | 1.2 | 17.84 | 0.501309 | 1.6 | 17.52 | 0.492164 | 1 | 17.59 | 0.504404 | 1.4 |
| 242 | Spectrin beta chain, erythrocytic OS=Homo sapiens GN=SPTB PE=1 SV=5 | SPTB1_HUMAN | 246 kDa | TRUE | 0.0001 | 1.45 | 1.40 | 1.20 | 17.7 | 0.877293 | 17.65 | 0.893366 | 18.16 | 0.54389 | 1.4 | 18.19 | 0.56119 | 1.5 | 18.1 | 0.531885 | 1.3 | 18.17 | 0.575912 | 1.5 | 17.86 | 0.531689 | 1.2 | 17.82 | 0.558206 | 1.2 |
| 87 | Collagen alpha-3(VI) chain OS=Homo sapiens GN=COL6A3 PE=1 SV=5 | CO6A3_HUMAN | 344 kDa |  | 0.0001 | 1.40 | 1.40 | 1.20 | 17.96 | 0.776987 | 17.94 | 0.725555 | 18.39 | 0.557582 | 1.4 | 18.42 | 0.558565 | 1.4 | 18.37 | 0.551333 | 1.3 | 18.46 | 0.54797 | 1.5 | 18.14 | 0.530451 | 1.2 | 18.17 | 0.552272 | 1.2 |
| 1572 | A disintegrin and metalloproteinase with thrombospondin motifs 2 OS=Homo sapiens GN=ADAMTS2 PE=2 SV=2 | ATS2_HUMAN | 135 kDa |  | 0.026 | 1.35 | 1.50 | 1.20 | 18.03 | 0.70659 | 17.91 | 0.59755 | 18.37 | 0.500452 | 1.3 | 18.39 | 0.505952 | 1.4 | 18.58 | 0.525662 | 1.5 | 18.48 | 0.504024 | 1.5 | 18.22 | 0.497439 | 1.2 | 18.22 | 0.498398 | 1.2 |
| 862 | Ficolin-1 OS=Homo sapiens GN=FCN1 PE=1 SV=2 | FCN1_HUMAN | 35 kDa | TRUE | 0.0012 | 1.60 | 1.45 | 1.20 | 18.68 | 0.61628 | 18.64 | 0.618071 | 19.34 | 0.543132 | 1.6 | 19.33 | 0.541589 | 1.6 | 19.22 | 0.517113 | 1.5 | 19.12 | 0.533134 | 1.4 | 19 | 0.548543 | 1.3 | 18.82 | 0.521948 | 1.1 |
| 1100 | U6 snRNA-associated Sm-like protein LSm2 OS=Homo sapiens GN=LSM2 PE=1 SV=1 | LSM2_HUMAN | 11 kDa |  | 0.00041 | 1.70 | 1.30 | 1.25 | 17.51 | 0.735518 | 17.48 | 0.776254 | 18.17 | 0.541329 | 1.6 | 18.24 | 0.544511 | 1.8 | 17.71 | 0.514682 | 1.2 | 17.9 | 0.525765 | 1.4 | 17.81 | 0.545406 | 1.2 | 17.85 | 0.596753 | 1.3 |
| 56 | Spectrin beta chain, non-erythrocytic 1 OS=Homo sapiens GN=SPTBN1 PE=1 SV=2 | SPTB2_HUMAN | 275 kDa | TRUE | 0.0001 | 1.50 | 1.45 | 1.25 | 17.81 | 0.829741 | 17.79 | 0.790468 | 18.37 | 0.585193 | 1.5 | 18.39 | 0.58201 | 1.5 | 18.24 | 0.550597 | 1.4 | 18.29 | 0.552021 | 1.5 | 18.06 | 0.535368 | 1.2 | 18.13 | 0.557008 | 1.3 |
| 66 | Spectrin alpha chain, erythrocytic 1 OS=Homo sapiens GN=SPTA1 PE=1 SV=5 | SPTA1_HUMAN | 280 kDa | TRUE | 0.0001 | 1.55 | 1.50 | 1.25 | 17.33 | 0.844818 | 17.29 | 0.867336 | 17.92 | 0.5881 | 1.5 | 17.94 | 0.595783 | 1.6 | 17.84 | 0.538025 | 1.4 | 17.88 | 0.561872 | 1.6 | 17.63 | 0.526513 | 1.3 | 17.6 | 0.571055 | 1.2 |
| 1529 | Resistin OS=Homo sapiens GN=RETN PE=1 SV=1 | RETN_HUMAN | 11 kDa |  | 0.0022 | 2.20 | 1.60 | 1.25 | 18.2 | 1.154635 | 18.21 | 0.95642 | 19.47 | 0.833056 | 2.2 | 19.54 | 0.818726 | 2.2 | 18.85 | 0.56355 | 1.6 | 18.91 | 0.562494 | 1.6 | 18.42 | 0.644893 | 1.3 | 18.43 | 0.56137 | 1.2 |
| 1433 | Prosaposin OS=Homo sapiens GN=PSAP PE=1 SV=2 | SAP_HUMAN | 58 kDa |  | 0.00073 | 1.55 | 1.65 | 1.25 | 16.74 | 0.574351 | 16.73 | 0.6475 | 17.3 | 0.532241 | 1.5 | 17.32 | 0.532126 | 1.6 | 17.23 | 0.501448 | 1.4 | 17.59 | 0.708637 | 1.9 | 17.01 | 0.499293 | 1.2 | 17.07 | 0.503488 | 1.3 |
| 1816 | Apoptosis-associated speck-like protein containing a CARD OS=Homo sapiens GN=PYCARD PE=1 SV=2 | ASC_HUMAN | 22 kDa |  | 0.026 | 1.65 | 1.30 | 1.30 | 16.22 | 0.486722 | 15.98 | 0.543717 | 16.93 | 0.48753 | 1.6 | 16.78 | 0.483699 | 1.7 | 16.51 | 0.478928 | 1.2 | 16.46 | 0.55836 | 1.4 | 16.48 | 0.483976 | 1.2 | 16.47 | 0.477934 | 1.4 |
| 486 | Interstitial collagenase OS=Homo sapiens GN=MMP1 PE=1 SV=3 | MMP1_HUMAN | 54 kDa |  | 0.0001 | 1.60 | 1.40 | 1.30 | 17.86 | 0.659793 | 17.97 | 0.674595 | 18.54 | 0.56134 | 1.6 | 18.61 | 0.561961 | 1.6 | 18.4 | 0.526492 | 1.5 | 18.37 | 0.510304 | 1.3 | 18.34 | 0.521351 | 1.4 | 18.21 | 0.650535 | 1.2 |
| 347 | Cullin-3 OS=Homo sapiens GN=CUL3 PE=1 SV=2 | CUL3_HUMAN | 89 kDa | TRUE | 0.00014 | 1.40 | 1.40 | 1.30 | 17.57 | 0.762443 | 17.42 | 0.870526 | 17.98 | 0.58232 | 1.3 | 17.99 | 0.585792 | 1.5 | 17.91 | 0.546958 | 1.3 | 18.03 | 0.537878 | 1.5 | 17.86 | 0.512668 | 1.2 | 17.88 | 0.546138 | 1.4 |
| 1114 | ATP synthase subunit beta, mitochondrial OS=Homo sapiens GN=ATP5B PE=1 SV=3 | ATPB_HUMAN | 57 kDa | TRUE | 0.011 | 1.40 | 1.45 | 1.30 | 17.19 | 0.698462 | 17.17 | 0.695113 | 17.73 | 0.525378 | 1.4 | 17.62 | 0.552913 | 1.4 | 17.69 | 0.555412 | 1.4 | 17.7 | 0.522028 | 1.5 | 17.51 | 0.49648 | 1.2 | 17.61 | 0.493353 | 1.4 |
| 956 | Protein phosphatase 1A OS=Homo sapiens GN=PPM1A PE=1 SV=1 | PPM1A_HUMAN | 42 kDa | TRUE | 0.016 | 1.40 | 1.45 | 1.30 | 17.59 | 0.628065 | 17.52 | 0.710801 | 18 | 0.60212 | 1.4 | 17.99 | 0.529718 | 1.4 | 18.02 | 0.673116 | 1.5 | 18.01 | 0.535455 | 1.4 | 17.86 | 0.498372 | 1.2 | 18.04 | 0.675183 | 1.4 |
| 1372 | UPF0587 protein C1orf123 OS=Homo sapiens GN=C1orf123 PE=1 SV=1 | CA123_HUMAN | 18 kDa |  | 0.0038 | 1.05 | 1.50 | 1.30 | 17.78 | 0.565005 | 18.02 | 0.551087 | 17.91 | 0.514523 | 1.1 | 18.02 | 0.517218 | 1 | 18.36 | 0.508558 | 1.5 | 18.64 | 0.650834 | 1.5 | 18.23 | 0.510542 | 1.4 | 18.25 | 0.516312 | 1.2 |
| 1149 | Ras suppressor protein 1 OS=Homo sapiens GN=RSU1 PE=1 SV=3 | RSU1_HUMAN | 32 kDa |  | 0.0039 | 1.85 | 1.25 | 1.30 | 17.65 | 0.865695 | 17.57 | 0.61368 | 18.46 | 0.570302 | 1.8 | 18.54 | 0.58556 | 1.9 | 17.89 | 0.58053 | 1.3 | 17.88 | 0.513067 | 1.2 | 17.95 | 0.525846 | 1.3 | 17.97 | 0.508455 | 1.3 |
| 1293 | Protein PRRC1 OS=Homo sapiens GN=PRRC1 PE=1 SV=1 | PRRC1_HUMAN | 47 kDa | TRUE | 0.027 | 1.60 | 1.25 | 1.30 | 16.87 | 0.8045 | 17 | 0.802466 | 17.65 | 0.507123 | 1.7 | 17.65 | 0.521581 | 1.5 | 17.27 | 0.518287 | 1.4 | 17.13 | 0.5085 | 1.1 | 17.2 | 0.511475 | 1.3 | 17.27 | 0.695125 | 1.3 |
| 316 | Exportin-1 OS=Homo sapiens GN=XPO1 PE=1 SV=1 | XPO1_HUMAN | 123 kDa | TRUE | 0.0001 | 1.65 | 1.30 | 1.30 | 17.33 | 0.790629 | 17.36 | 0.701353 | 18.07 | 0.574569 | 1.7 | 18.06 | 0.580848 | 1.6 | 17.65 | 0.530777 | 1.3 | 17.71 | 0.542798 | 1.3 | 17.72 | 0.51204 | 1.3 | 17.77 | 0.560876 | 1.3 |
| 391 | Dedicator of cytokinesis protein 2 OS=Homo sapiens GN=DOCK2 PE=1 SV=2 | DOCK2_HUMAN | 212 kDa | TRUE | 0.0001 | 1.45 | 1.30 | 1.30 | 17.24 | 0.669704 | 17.14 | 0.708148 | 17.71 | 0.532812 | 1.4 | 17.68 | 0.575474 | 1.5 | 17.6 | 0.54923 | 1.3 | 17.51 | 0.499836 | 1.3 | 17.56 | 0.509787 | 1.3 | 17.49 | 0.542954 | 1.3 |
| 1436 | Cohesin subunit SA-2 OS=Homo sapiens GN=STAG2 PE=1 SV=3 | STAG2_HUMAN | 141 kDa | TRUE | 0.0037 | 1.75 | 1.40 | 1.30 | 15.76 | 0.538224 | 15.52 | 0.547203 | 16.46 | 0.591453 | 1.7 | 16.37 | 0.608948 | 1.8 | 16.09 | 0.504716 | 1.3 | 16.14 | 0.518345 | 1.5 | 16.19 | 0.49717 | 1.3 | 16.08 | 1.032074 | 1.3 |
| 667 | Cullin-2 OS=Homo sapiens GN=CUL2 PE=1 SV=2 | CUL2_HUMAN | 87 kDa |  | 0.00028 | 1.60 | 1.40 | 1.30 | 17.24 | 0.799639 | 17.28 | 0.734543 | 17.9 | 0.567875 | 1.6 | 17.92 | 0.551227 | 1.6 | 17.72 | 0.519933 | 1.4 | 17.71 | 0.50605 | 1.4 | 17.65 | 0.511571 | 1.3 | 17.64 | 0.572709 | 1.3 |
| 825 | Glutamate--cysteine ligase regulatory subunit OS=Homo sapiens GN=GCLM PE=1 SV=1 | GSH0_HUMAN | 31 kDa | TRUE | 0.026 | 1.55 | 1.40 | 1.30 | 18.23 | 0.889548 | 18.23 | 0.945479 | 18.9 | 0.569508 | 1.6 | 18.82 | 0.587181 | 1.5 | 18.68 | 0.568415 | 1.4 | 18.63 | 0.567669 | 1.4 | 18.51 | 0.558528 | 1.3 | 18.54 | 0.570935 | 1.3 |
| 261 | Laminin subunit beta-1 OS=Homo sapiens GN=LAMB1 PE=1 SV=2 | LAMB1_HUMAN | 198 kDa | TRUE | 0.0001 | 1.50 | 1.40 | 1.30 | 17.37 | 0.764488 | 17.38 | 0.727731 | 17.93 | 0.560088 | 1.5 | 17.94 | 0.542729 | 1.5 | 17.88 | 0.524604 | 1.4 | 17.84 | 0.514366 | 1.4 | 17.77 | 0.518501 | 1.3 | 17.77 | 0.535549 | 1.3 |
| 284 | 1,4-alpha-glucan-branching enzyme OS=Homo sapiens GN=GBE1 PE=1 SV=3 | GLGB_HUMAN | 80 kDa |  | 0.0001 | 1.50 | 1.40 | 1.30 | 18.16 | 0.770517 | 18.18 | 0.730848 | 18.7 | 0.620765 | 1.5 | 18.71 | 0.583233 | 1.5 | 18.62 | 0.540601 | 1.4 | 18.67 | 0.555539 | 1.4 | 18.52 | 0.520836 | 1.3 | 18.49 | 0.568924 | 1.3 |
| 754 | Engulfment and cell motility protein 1 OS=Homo sapiens GN=ELMO1 PE=1 SV=2 | ELMO1_HUMAN | 84 kDa | TRUE | 0.002 | 1.45 | 1.40 | 1.30 | 16.95 | 0.577924 | 17.1 | 0.587404 | 17.53 | 0.577833 | 1.5 | 17.56 | 0.519469 | 1.4 | 17.42 | 0.524204 | 1.4 | 17.62 | 0.550716 | 1.4 | 17.37 | 0.532169 | 1.3 | 17.48 | 0.553526 | 1.3 |
| 1074 | Immunoglobulin superfamily containing leucine-rich repeat protein OS=Homo sapiens GN=ISLR PE=2 SV=1 | ISLR_HUMAN | 46 kDa |  | 0.0034 | 1.45 | 1.40 | 1.30 | 17.72 | 0.606345 | 17.7 | 0.587024 | 18.24 | 0.536417 | 1.4 | 18.27 | 0.523029 | 1.5 | 18.1 | 0.491226 | 1.3 | 18.24 | 0.497641 | 1.5 | 18.04 | 0.492107 | 1.3 | 18.01 | 0.517145 | 1.3 |
| 476 | Actin-related protein 2/3 complex subunit 5 OS=Homo sapiens GN=ARPC5 PE=1 SV=3 | ARPC5_HUMAN | 16 kDa | TRUE | 0.00013 | 1.60 | 1.45 | 1.30 | 17.84 | 0.84177 | 17.82 | 0.780359 | 18.55 | 0.627228 | 1.6 | 18.55 | 0.629697 | 1.6 | 18.43 | 0.523919 | 1.5 | 18.25 | 0.524244 | 1.4 | 18.19 | 0.533936 | 1.3 | 18.16 | 0.527688 | 1.3 |
| 524 | Ras-related protein Rab-11B OS=Homo sapiens GN=RAB11B PE=1 SV=4 | RB11B_HUMAN | 24 kDa |  | 0.0001 | 1.55 | 1.45 | 1.30 | 18.35 | 0.884542 | 18.32 | 0.81727 | 18.92 | 0.527976 | 1.5 | 18.95 | 0.527542 | 1.6 | 18.79 | 0.550187 | 1.4 | 18.82 | 0.578444 | 1.5 | 18.61 | 0.531692 | 1.3 | 18.62 | 0.529211 | 1.3 |
| 45 | Spectrin alpha chain, non-erythrocytic 1 OS=Homo sapiens GN=SPTAN1 PE=1 SV=3 | SPTN1_HUMAN | 285 kDa | TRUE | 0.0001 | 1.50 | 1.45 | 1.30 | 18.09 | 0.873257 | 18.12 | 0.813806 | 18.66 | 0.600058 | 1.5 | 18.7 | 0.600785 | 1.5 | 18.62 | 0.554435 | 1.5 | 18.6 | 0.548952 | 1.4 | 18.41 | 0.531662 | 1.3 | 18.43 | 0.560179 | 1.3 |
| 307 | AMP deaminase 3 OS=Homo sapiens GN=AMPD3 PE=1 SV=1 | AMPD3_HUMAN | 89 kDa | TRUE | 0.0001 | 1.40 | 1.45 | 1.30 | 17.96 | 0.844216 | 17.96 | 0.834042 | 18.44 | 0.551276 | 1.4 | 18.47 | 0.547862 | 1.4 | 18.4 | 0.537389 | 1.4 | 18.46 | 0.550177 | 1.5 | 18.28 | 0.529277 | 1.3 | 18.28 | 0.589225 | 1.3 |
| 1170 | Protein ABHD14B OS=Homo sapiens GN=ABHD14B PE=1 SV=1 | ABHEB_HUMAN | 22 kDa |  | 0.004 | 1.35 | 1.50 | 1.30 | 18.03 | 0.6167 | 18.08 | 0.850615 | 18.48 | 0.549441 | 1.4 | 18.47 | 0.587864 | 1.3 | 18.71 | 0.53053 | 1.6 | 18.48 | 0.50993 | 1.4 | 18.43 | 0.501679 | 1.3 | 18.39 | 0.517679 | 1.3 |
| 1252 | Ethylmalonyl-CoA decarboxylase OS=Homo sapiens GN=ECHDC1 PE=1 SV=2 | ECHD1_HUMAN | 34 kDa | TRUE | 0.0079 | 1.40 | 1.65 | 1.30 | 16.78 | 0.70526 | 16.74 | 0.69847 | 17.26 | 0.517085 | 1.4 | 17.22 | 0.531385 | 1.4 | 17.55 | 0.541066 | 1.8 | 17.32 | 0.510195 | 1.5 | 17.15 | 0.512677 | 1.3 | 17.11 | 0.506864 | 1.3 |
| 724 | Fatty acid-binding protein, epidermal OS=Homo sapiens GN=FABP5 PE=1 SV=3 | FABP5_HUMAN | 15 kDa |  | 0.0001 | -1.67 | 1.00 | 1.35 | 18.88 | 0.556694 | 18.88 | 0.58709 | 18.18 | 0.790529 | 0.6 | 18.2 | 0.82316 | 0.6 | 18.92 | 0.556427 | 1 | 18.83 | 0.531855 | 1 | 19.29 | 0.565944 | 1.3 | 19.38 | 0.575683 | 1.4 |
| 280 | Serine/threonine-protein kinase 10 OS=Homo sapiens GN=STK10 PE=1 SV=1 | STK10_HUMAN | 112 kDa | TRUE | 0.0001 | 1.60 | 1.40 | 1.35 | 17.92 | 0.804814 | 17.83 | 0.826757 | 18.5 | 0.57228 | 1.5 | 18.49 | 0.592536 | 1.7 | 18.31 | 0.525506 | 1.3 | 18.41 | 0.531866 | 1.5 | 18.3 | 0.516047 | 1.3 | 18.33 | 0.554057 | 1.4 |
| 285 | Drebrin-like protein OS=Homo sapiens GN=DBNL PE=1 SV=1 | DBNL_HUMAN | 48 kDa | TRUE | 0.0001 | 1.55 | 1.40 | 1.35 | 17.81 | 0.727977 | 17.87 | 0.762269 | 18.45 | 0.601356 | 1.6 | 18.41 | 0.614491 | 1.5 | 18.3 | 0.548675 | 1.4 | 18.34 | 0.549285 | 1.4 | 18.24 | 0.512784 | 1.4 | 18.25 | 0.536606 | 1.3 |
| 650 | cAMP-dependent protein kinase type II-alpha regulatory subunit OS=Homo sapiens GN=PRKAR2A PE=1 SV=2 | KAP2_HUMAN | 46 kDa |  | 0.0001 | 1.55 | 1.40 | 1.35 | 17.66 | 0.784511 | 17.61 | 0.816133 | 18.19 | 0.59843 | 1.5 | 18.26 | 0.597638 | 1.6 | 18.16 | 0.518923 | 1.4 | 18.12 | 0.513309 | 1.4 | 18.15 | 0.53194 | 1.4 | 18.02 | 0.530674 | 1.3 |
| 589 | Protein FAM49B OS=Homo sapiens GN=FAM49B PE=1 SV=1 | FA49B_HUMAN | 37 kDa |  | 0.0001 | 1.50 | 1.40 | 1.35 | 17.98 | 0.698043 | 17.94 | 0.70247 | 18.45 | 0.6175 | 1.5 | 18.44 | 0.625907 | 1.5 | 18.44 | 0.544625 | 1.4 | 18.41 | 0.526219 | 1.4 | 18.36 | 0.520838 | 1.3 | 18.36 | 0.527381 | 1.4 |
| 614 | Alpha-adducin OS=Homo sapiens GN=ADD1 PE=1 SV=2 | ADDA_HUMAN | 81 kDa | TRUE | 0.0001 | 1.85 | 1.45 | 1.35 | 17.98 | 0.735586 | 17.94 | 0.732853 | 18.77 | 0.583253 | 1.8 | 18.8 | 0.585829 | 1.9 | 18.52 | 0.558119 | 1.4 | 18.47 | 0.552871 | 1.5 | 18.27 | 0.574405 | 1.3 | 18.4 | 0.592628 | 1.4 |
| 442 | 26S proteasome non-ATPase regulatory subunit 13 OS=Homo sapiens GN=PSMD13 PE=1 SV=2 | PSD13_HUMAN | 43 kDa |  | 0.0001 | 1.70 | 1.45 | 1.35 | 17.12 | 0.923136 | 17.03 | 0.870243 | 17.79 | 0.576179 | 1.7 | 17.78 | 0.585507 | 1.7 | 17.58 | 0.523268 | 1.4 | 17.57 | 0.564548 | 1.5 | 17.5 | 0.52418 | 1.3 | 17.46 | 0.627075 | 1.4 |
| 706 | Ras-related protein Rab-5C OS=Homo sapiens GN=RAB5C PE=1 SV=2 | RAB5C_HUMAN | 23 kDa | TRUE | 0.0011 | 1.60 | 1.45 | 1.35 | 16.72 | 0.594283 | 16.7 | 0.598013 | 17.37 | 0.579864 | 1.6 | 17.38 | 0.569138 | 1.6 | 17.23 | 0.513979 | 1.4 | 17.33 | 0.511379 | 1.5 | 17.08 | 0.511814 | 1.3 | 17.19 | 0.52966 | 1.4 |
| 85 | Vinculin OS=Homo sapiens GN=VCL PE=1 SV=4 | VINC_HUMAN | 124 kDa |  | 0.0001 | 1.50 | 1.45 | 1.35 | 17.89 | 0.77955 | 17.91 | 0.742845 | 18.48 | 0.563981 | 1.5 | 18.47 | 0.554645 | 1.5 | 18.44 | 0.547461 | 1.5 | 18.42 | 0.543211 | 1.4 | 18.29 | 0.514949 | 1.3 | 18.36 | 0.554981 | 1.4 |
| 578 | Protein phosphatase 1B OS=Homo sapiens GN=PPM1B PE=1 SV=1 | PPM1B_HUMAN | 53 kDa | TRUE | 0.0053 | 1.50 | 1.45 | 1.35 | 18.03 | 0.825462 | 17.99 | 0.814267 | 18.57 | 0.534032 | 1.5 | 18.52 | 0.524891 | 1.5 | 18.53 | 0.51927 | 1.4 | 18.47 | 0.525221 | 1.5 | 18.39 | 0.517348 | 1.3 | 18.41 | 0.534864 | 1.4 |
| 652 | Protein disulfide-isomerase A3 OS=Homo sapiens GN=PDIA3 PE=1 SV=4 | PDIA3_HUMAN | 57 kDa | TRUE | 0.0001 | 1.35 | 1.45 | 1.35 | 17.5 | 0.626614 | 17.48 | 0.631939 | 17.91 | 0.540849 | 1.3 | 17.99 | 0.537727 | 1.4 | 18.04 | 0.513836 | 1.5 | 18 | 0.517125 | 1.4 | 17.9 | 0.513142 | 1.3 | 17.95 | 0.645538 | 1.4 |
| 943 | Endoplasmic reticulum resident protein 44 OS=Homo sapiens GN=ERP44 PE=1 SV=1 | ERP44_HUMAN | 47 kDa |  | 0.0014 | 1.30 | 1.45 | 1.35 | 18.12 | 0.612204 | 18.18 | 0.72074 | 18.54 | 0.538753 | 1.3 | 18.58 | 0.544863 | 1.3 | 18.68 | 0.512723 | 1.5 | 18.68 | 0.529588 | 1.4 | 18.6 | 0.508528 | 1.4 | 18.58 | 0.524709 | 1.3 |
| 1562 | Serine/threonine-protein kinase MST4 OS=Homo sapiens GN=MST4 PE=1 SV=2 | MST4_HUMAN | ? | TRUE | 0.019 | 1.45 | 1.45 | 1.35 | 18.68 | 0.513101 | 18.3 | 0.52035 | 19.05 | 0.634881 | 1.3 | 18.96 | 0.510416 | 1.6 | 19.09 | 0.515895 | 1.3 | 18.99 | 0.503123 | 1.6 | 19.03 | 0.51695 | 1.3 | 18.8 | 0.514594 | 1.4 |
| 973 | Src kinase-associated phosphoprotein 2 OS=Homo sapiens GN=SKAP2 PE=1 SV=1 | SKAP2_HUMAN | 41 kDa |  | 0.019 | 1.30 | 1.45 | 1.35 | 17.66 | 0.96997 | 17.26 | 0.949549 | 17.8 | 0.531496 | 1.2 | 17.71 | 0.505904 | 1.4 | 17.95 | 0.535285 | 1.3 | 17.85 | 0.53454 | 1.6 | 17.84 | 0.511365 | 1.2 | 17.91 | 0.591872 | 1.5 |
| 479 | Cytokine receptor-like factor 3 OS=Homo sapiens GN=CRLF3 PE=1 SV=2 | CRLF3_HUMAN | 50 kDa | TRUE | 0.0001 | 1.55 | 1.50 | 1.35 | 17.64 | 0.683541 | 17.58 | 0.670186 | 18.19 | 0.5476 | 1.5 | 18.26 | 0.544895 | 1.6 | 18.08 | 0.502594 | 1.4 | 18.18 | 0.530937 | 1.6 | 18.06 | 0.514947 | 1.3 | 18.09 | 0.537884 | 1.4 |
| 104 | Transforming growth factor-beta-induced protein ig-h3 OS=Homo sapiens GN=TGFBI PE=1 SV=1 | BGH3_HUMAN | 75 kDa | TRUE | 0.0001 | 1.45 | 1.50 | 1.35 | 17.73 | 0.820875 | 17.77 | 0.800777 | 18.19 | 0.661851 | 1.5 | 18.23 | 0.659189 | 1.4 | 18.28 | 0.614288 | 1.5 | 18.29 | 0.555331 | 1.5 | 18.09 | 0.547071 | 1.3 | 18.17 | 0.63816 | 1.4 |
| 1038 | Tubulin alpha-4A chain OS=Homo sapiens GN=TUBA4A PE=1 SV=1 | TBA4A_HUMAN | 50 kDa | TRUE | 0.0064 | 1.30 | 1.50 | 1.35 | 16.83 | 0.736282 | 16.74 | 0.670818 | 17.15 | 0.556318 | 1.2 | 17.16 | 0.50837 | 1.4 | 17.26 | 0.510991 | 1.4 | 17.25 | 0.592009 | 1.6 | 17.18 | 0.564898 | 1.3 | 17.12 | 0.578856 | 1.4 |
| 746 | Histidine--tRNA ligase, cytoplasmic OS=Homo sapiens GN=HARS PE=1 SV=2 | SYHC_HUMAN | 57 kDa |  | 0.017 | 1.20 | 1.50 | 1.35 | 18.28 | 0.548117 | 17.99 | 0.497435 | 18.42 | 0.501976 | 1.1 | 18.37 | 0.500186 | 1.3 | 18.62 | 0.505681 | 1.3 | 18.8 | 0.505882 | 1.7 | 18.54 | 0.497886 | 1.2 | 18.61 | 0.495781 | 1.5 |
| 409 | Glutathione S-transferase P OS=Homo sapiens GN=GSTP1 PE=1 SV=2 | GSTP1_HUMAN | 23 kDa |  | 0.0062 | 1.30 | 1.55 | 1.35 | 17.94 | 0.85605 | 17.92 | 0.804975 | 18.26 | 0.679848 | 1.3 | 18.27 | 0.69683 | 1.3 | 18.48 | 0.659108 | 1.6 | 18.42 | 0.591728 | 1.5 | 18.34 | 0.531974 | 1.3 | 18.41 | 0.553282 | 1.4 |
| 861 | FAS-associated factor 1 OS=Homo sapiens GN=FAF1 PE=1 SV=2 | FAF1_HUMAN | 74 kDa |  | 0.0087 | 1.40 | 1.60 | 1.35 | 17.35 | 1.048253 | 17.29 | 0.951264 | 17.77 | 0.530104 | 1.3 | 17.84 | 0.509651 | 1.5 | 17.88 | 0.505657 | 1.4 | 17.93 | 0.622469 | 1.8 | 17.77 | 0.495011 | 1.3 | 17.62 | 0.514993 | 1.4 |
| 295 | Cathelicidin antimicrobial peptide OS=Homo sapiens GN=CAMP PE=1 SV=1 | CAMP_HUMAN | 19 kDa | TRUE | 0.0001 | 1.90 | 1.75 | 1.35 | 18.4 | 1.041837 | 18.4 | 1.026504 | 19.13 | 0.810844 | 1.9 | 19.13 | 0.81744 | 1.9 | 19.24 | 0.585034 | 1.8 | 19.04 | 0.619577 | 1.7 | 18.8 | 0.564243 | 1.4 | 18.66 | 0.592901 | 1.3 |
| 934 | Band 3 anion transport protein OS=Homo sapiens GN=SLC4A1 PE=1 SV=3 | B3AT_HUMAN | 102 kDa | TRUE | 0.0001 | 1.40 | 1.75 | 1.35 | 17.96 | 0.780749 | 17.98 | 0.79046 | 18.37 | 0.542411 | 1.4 | 18.43 | 0.586297 | 1.4 | 18.77 | 0.57839 | 1.8 | 18.71 | 0.546245 | 1.7 | 18.43 | 0.546979 | 1.4 | 18.34 | 0.564652 | 1.3 |
| 900 | Dihydropyrimidinase-related protein 3 OS=Homo sapiens GN=DPYSL3 PE=1 SV=1 | DPYL3_HUMAN | 62 kDa | TRUE | 0.0001 | 1.00 | 1.25 | 1.40 | 18.39 | 0.755887 | 18.44 | 0.646307 | 18.44 | 0.538429 | 1 | 18.41 | 0.512776 | 1 | 18.69 | 0.552287 | 1.3 | 18.67 | 0.515126 | 1.2 | 18.95 | 0.584954 | 1.4 | 18.96 | 0.568162 | 1.4 |
| 621 | Cullin-5 OS=Homo sapiens GN=CUL5 PE=1 SV=4 | CUL5_HUMAN | 91 kDa |  | 0.0076 | 1.45 | 1.30 | 1.40 | 17.38 | 0.645614 | 17.29 | 0.652739 | 17.85 | 0.643109 | 1.4 | 17.85 | 0.609276 | 1.5 | 17.67 | 0.51786 | 1.2 | 17.8 | 0.531083 | 1.4 | 17.72 | 0.504637 | 1.3 | 17.79 | 0.552892 | 1.5 |
| 783 | Cytoplasmic dynein 1 intermediate chain 2 OS=Homo sapiens GN=DYNC1I2 PE=1 SV=3 | DC1I2_HUMAN | 71 kDa | TRUE | 0.0013 | 1.10 | 1.30 | 1.40 | 17.48 | 0.900851 | 17.32 | 0.845831 | 17.49 | 0.550897 | 1 | 17.52 | 0.54122 | 1.2 | 17.71 | 0.566214 | 1.2 | 17.78 | 0.499795 | 1.4 | 17.9 | 0.592982 | 1.3 | 17.96 | 0.605765 | 1.5 |
| 1009 | Eukaryotic translation initiation factor 4E OS=Homo sapiens GN=EIF4E PE=1 SV=2 | IF4E_HUMAN | 25 kDa |  | 0.0001 | 1.00 | 1.30 | 1.40 | 17.88 | 0.519212 | 18.01 | 0.51103 | 17.82 | 0.524565 | 1 | 17.97 | 0.503201 | 1 | 18.33 | 0.500537 | 1.4 | 18.29 | 0.522126 | 1.2 | 18.41 | 0.520443 | 1.4 | 18.51 | 0.509863 | 1.4 |
| 1175 | COP9 signalosome complex subunit 7a OS=Homo sapiens GN=COPS7A PE=1 SV=1 | CSN7A_HUMAN | 30 kDa |  | 0.021 | 1.45 | 1.30 | 1.40 | 17.7 | 0.879216 | 17.74 | 0.942999 | 18.16 | 0.524183 | 1.5 | 18.19 | 0.518359 | 1.4 | 18.07 | 0.501743 | 1.3 | 18.12 | 0.499281 | 1.3 | 18.18 | 0.507327 | 1.4 | 18.14 | 0.500845 | 1.4 |
| 67 | Cytoplasmic dynein 1 heavy chain 1 OS=Homo sapiens GN=DYNC1H1 PE=1 SV=5 | DYHC1_HUMAN | 532 kDa | TRUE | 0.0001 | 1.25 | 1.30 | 1.40 | 17.29 | 0.848077 | 17.32 | 0.774239 | 17.61 | 0.555935 | 1.2 | 17.64 | 0.587041 | 1.3 | 17.63 | 0.51922 | 1.3 | 17.67 | 0.535701 | 1.3 | 17.77 | 0.524371 | 1.4 | 17.82 | 0.524862 | 1.4 |
| 604 | U1 small nuclear ribonucleoprotein 70 kDa OS=Homo sapiens GN=SNRNP70 PE=1 SV=2 | RU17_HUMAN | 52 kDa |  | 0.0004 | 1.25 | 1.30 | 1.40 | 17.98 | 0.758336 | 18.01 | 0.920298 | 18.24 | 0.513391 | 1.2 | 18.31 | 0.513837 | 1.3 | 18.34 | 0.518615 | 1.3 | 18.34 | 0.556058 | 1.3 | 18.58 | 0.567239 | 1.5 | 18.46 | 0.541655 | 1.3 |
| 668 | L-xylulose reductase OS=Homo sapiens GN=DCXR PE=1 SV=2 | DCXR_HUMAN | 26 kDa |  | 0.00058 | 1.60 | 1.35 | 1.40 | 18.11 | 0.689301 | 18.2 | 0.752587 | 18.88 | 0.628167 | 1.7 | 18.83 | 0.630231 | 1.5 | 18.56 | 0.56865 | 1.4 | 18.55 | 0.524211 | 1.3 | 18.55 | 0.507199 | 1.4 | 18.69 | 0.614381 | 1.4 |
| 235 | DNA damage-binding protein 1 OS=Homo sapiens GN=DDB1 PE=1 SV=1 | DDB1_HUMAN | 127 kDa |  | 0.0001 | 1.55 | 1.35 | 1.40 | 18 | 0.879709 | 18 | 0.875595 | 18.53 | 0.68596 | 1.5 | 18.56 | 0.654414 | 1.6 | 18.36 | 0.525874 | 1.3 | 18.39 | 0.562606 | 1.4 | 18.48 | 0.542975 | 1.4 | 18.48 | 0.547398 | 1.4 |
| 873 | Glucose 1,6-bisphosphate synthase OS=Homo sapiens GN=PGM2L1 PE=1 SV=3 | PGM2L_HUMAN | 70 kDa | TRUE | 0.0001 | 1.50 | 1.35 | 1.40 | 18.12 | 0.64178 | 18.16 | 0.580332 | 18.68 | 0.552841 | 1.5 | 18.7 | 0.542955 | 1.5 | 18.52 | 0.530175 | 1.3 | 18.61 | 0.501012 | 1.4 | 18.53 | 0.53475 | 1.4 | 18.63 | 0.581835 | 1.4 |
| 511 | Protein arginine N-methyltransferase 5 OS=Homo sapiens GN=PRMT5 PE=1 SV=4 | ANM5_HUMAN | 73 kDa |  | 0.00061 | 1.50 | 1.35 | 1.40 | 16.79 | 0.832413 | 16.65 | 0.869617 | 17.31 | 0.539997 | 1.4 | 17.34 | 0.553708 | 1.6 | 17.12 | 0.562002 | 1.3 | 17.08 | 0.51402 | 1.4 | 17.18 | 0.539856 | 1.3 | 17.18 | 0.540987 | 1.5 |
| 291 | E3 ubiquitin-protein ligase RNF123 OS=Homo sapiens GN=RNF123 PE=1 SV=1 | RN123_HUMAN | 149 kDa | TRUE | 0.0001 | 1.65 | 1.40 | 1.40 | 18.12 | 1.011366 | 18.14 | 0.982063 | 18.86 | 0.574976 | 1.7 | 18.82 | 0.581948 | 1.6 | 18.65 | 0.568226 | 1.4 | 18.58 | 0.540987 | 1.4 | 18.6 | 0.541427 | 1.4 | 18.62 | 0.576701 | 1.4 |
| 69 | Serotransferrin OS=Homo sapiens GN=TF PE=1 SV=3 | TRFE_HUMAN | 77 kDa | TRUE | 0.0001 | 1.00 | 1.40 | 1.40 | 18.35 | 0.698666 | 18.42 | 0.68925 | 18.38 | 0.668846 | 1 | 18.39 | 0.674261 | 1 | 18.96 | 0.633827 | 1.5 | 18.82 | 0.591185 | 1.3 | 18.88 | 0.589519 | 1.5 | 18.76 | 0.621227 | 1.3 |
| 838 | Septin-2 OS=Homo sapiens GN=SEPT2 PE=1 SV=1 | SEPT2_HUMAN | 41 kDa |  | 0.00081 | 1.00 | 1.40 | 1.40 | 18.48 | 0.859109 | 18.65 | 1.015049 | 18.65 | 0.529267 | 1.1 | 18.56 | 0.601117 | 0.9 | 19.04 | 0.543061 | 1.5 | 19 | 0.65508 | 1.3 | 19.11 | 0.570222 | 1.5 | 19.06 | 0.594493 | 1.3 |
| 234 | Calpain-1 catalytic subunit OS=Homo sapiens GN=CAPN1 PE=1 SV=1 | CAN1_HUMAN | 82 kDa |  | 0.0001 | 1.70 | 1.45 | 1.40 | 18.06 | 0.838934 | 18.04 | 0.783164 | 18.82 | 0.653573 | 1.7 | 18.79 | 0.665496 | 1.7 | 18.53 | 0.534601 | 1.4 | 18.63 | 0.600969 | 1.5 | 18.54 | 0.552928 | 1.4 | 18.54 | 0.555385 | 1.4 |
| 198 | Adenosylhomocysteinase OS=Homo sapiens GN=AHCY PE=1 SV=4 | SAHH_HUMAN | 48 kDa |  | 0.0001 | 1.45 | 1.45 | 1.40 | 18.11 | 0.911509 | 17.94 | 0.80144 | 18.56 | 0.531453 | 1.4 | 18.54 | 0.569105 | 1.5 | 18.61 | 0.56495 | 1.4 | 18.55 | 0.526664 | 1.5 | 18.45 | 0.522628 | 1.3 | 18.5 | 0.533187 | 1.5 |
| 445 | COP9 signalosome complex subunit 5 OS=Homo sapiens GN=COPS5 PE=1 SV=4 | CSN5_HUMAN | 38 kDa |  | 0.013 | 1.45 | 1.45 | 1.40 | 17.64 | 0.953817 | 17.49 | 0.876946 | 18.05 | 0.570337 | 1.4 | 18.05 | 0.579913 | 1.5 | 18.05 | 0.522095 | 1.4 | 18.03 | 0.534028 | 1.5 | 18.03 | 0.510291 | 1.3 | 18.02 | 0.517108 | 1.5 |
| 305 | Phosphoribosylformylglycinamidine synthase OS=Homo sapiens GN=PFAS PE=1 SV=4 | PUR4_HUMAN | 145 kDa | TRUE | 0.0001 | 1.40 | 1.45 | 1.40 | 18.1 | 0.90009 | 17.96 | 0.896148 | 18.49 | 0.607282 | 1.4 | 18.46 | 0.58293 | 1.4 | 18.6 | 0.563341 | 1.4 | 18.58 | 0.580969 | 1.5 | 18.48 | 0.551774 | 1.3 | 18.55 | 0.57199 | 1.5 |
| 423 | Hsp90 co-chaperone Cdc37 OS=Homo sapiens GN=CDC37 PE=1 SV=1 | CDC37_HUMAN | 44 kDa | TRUE | 0.0026 | 1.15 | 1.45 | 1.40 | 18.16 | 0.7848 | 18 | 0.724793 | 18.26 | 0.647438 | 1 | 18.38 | 0.611754 | 1.3 | 18.57 | 0.553439 | 1.4 | 18.55 | 0.578652 | 1.5 | 18.57 | 0.537567 | 1.3 | 18.58 | 0.538005 | 1.5 |
| 572 | Macrophage-capping protein OS=Homo sapiens GN=CAPG PE=1 SV=2 | CAPG_HUMAN | 38 kDa |  | 0.0001 | 1.00 | 1.45 | 1.40 | 18.36 | 0.745817 | 18.33 | 0.796528 | 18.45 | 0.627642 | 1 | 18.43 | 0.649641 | 1 | 18.9 | 0.610186 | 1.5 | 18.77 | 0.601385 | 1.4 | 18.84 | 0.570015 | 1.4 | 18.78 | 0.62261 | 1.4 |
| 792 | Microtubule-associated protein 4 OS=Homo sapiens GN=MAP4 PE=1 SV=3 | MAP4_HUMAN | 121 kDa |  | 0.0001 | 1.20 | 1.45 | 1.40 | 17.73 | 0.679454 | 17.63 | 0.769537 | 17.8 | 0.528413 | 1.1 | 17.96 | 0.533495 | 1.3 | 18.14 | 0.540655 | 1.3 | 18.26 | 0.56931 | 1.6 | 18.19 | 0.540593 | 1.4 | 18.2 | 0.511896 | 1.4 |
| 498 | Glycogen [starch] synthase, muscle OS=Homo sapiens GN=GYS1 PE=1 SV=2 | GYS1_HUMAN | 84 kDa | TRUE | 0.0001 | 1.95 | 1.50 | 1.40 | 17.57 | 0.783981 | 17.6 | 0.77074 | 18.51 | 0.632794 | 2 | 18.47 | 0.637423 | 1.9 | 18.16 | 0.583083 | 1.5 | 18.19 | 0.518095 | 1.5 | 17.97 | 0.506212 | 1.3 | 18.12 | 0.598727 | 1.5 |
| 570 | Amyloid beta A4 precursor protein-binding family B member 1-interacting protein OS=Homo sapiens GN=APBB1IP PE=1 SV=1 | AB1IP_HUMAN | 73 kDa | TRUE | 0.0001 | 1.60 | 1.50 | 1.40 | 17.95 | 0.665737 | 18.03 | 0.640958 | 18.64 | 0.563393 | 1.6 | 18.69 | 0.569125 | 1.6 | 18.56 | 0.512746 | 1.5 | 18.53 | 0.528584 | 1.5 | 18.43 | 0.512111 | 1.4 | 18.44 | 0.537632 | 1.4 |
| 1169 | Cytosolic 5'-nucleotidase 3A OS=Homo sapiens GN=NT5C3A PE=1 SV=3 | 5NT3A_HUMAN | 38 kDa | TRUE | 0.0023 | 1.50 | 1.50 | 1.40 | 17.5 | 0.64096 | 17.3 | 0.651649 | 17.91 | 0.52403 | 1.4 | 17.99 | 0.514385 | 1.6 | 18.04 | 0.553907 | 1.4 | 17.96 | 0.519093 | 1.6 | 17.99 | 0.522129 | 1.4 | 17.82 | 0.508804 | 1.4 |
| 1154 | Cysteine--tRNA ligase, cytoplasmic OS=Homo sapiens GN=CARS PE=1 SV=3 | SYCC_HUMAN | 85 kDa |  | 0.0021 | 1.15 | 1.50 | 1.40 | 17.54 | 0.674182 | 17.44 | 0.73476 | 17.66 | 0.513876 | 1.1 | 17.71 | 0.503346 | 1.2 | 18.01 | 0.560346 | 1.4 | 18.04 | 0.528412 | 1.6 | 17.95 | 0.517258 | 1.4 | 17.95 | 0.56672 | 1.4 |
| 913 | Neutrophil collagenase OS=Homo sapiens GN=MMP8 PE=1 SV=1 | MMP8_HUMAN | 53 kDa |  | 0.0005 | 1.75 | 1.55 | 1.40 | 18.16 | 0.730017 | 17.88 | 0.738325 | 18.77 | 0.545179 | 1.6 | 18.81 | 0.510471 | 1.9 | 18.6 | 0.512426 | 1.4 | 18.63 | 0.505006 | 1.7 | 18.48 | 0.49718 | 1.3 | 18.38 | 0.596631 | 1.5 |
| 779 | Cytidine deaminase OS=Homo sapiens GN=CDA PE=1 SV=2 | CDD_HUMAN | 16 kDa |  | 0.013 | -1.05 | 1.55 | 1.40 | 18.32 | 0.821464 | 18.38 | 0.945844 | 18.37 | 0.743767 | 1 | 18.29 | 0.662895 | 0.9 | 18.99 | 0.614524 | 1.7 | 18.78 | 0.632691 | 1.4 | 18.75 | 0.52088 | 1.4 | 18.79 | 0.554081 | 1.4 |
| 819 | Differentially expressed in FDCP 6 homolog OS=Homo sapiens GN=DEF6 PE=1 SV=1 | DEFI6_HUMAN | 74 kDa |  | 0.00024 | 1.65 | 1.55 | 1.40 | 17.43 | 0.719273 | 17.49 | 0.837294 | 18.13 | 0.536908 | 1.7 | 18.17 | 0.572637 | 1.6 | 18.02 | 0.50742 | 1.6 | 17.98 | 0.51662 | 1.5 | 17.87 | 0.504448 | 1.4 | 17.95 | 0.508434 | 1.4 |
| 523 | Ras-related protein Rab-7a OS=Homo sapiens GN=RAB7A PE=1 SV=1 | RAB7A_HUMAN | 23 kDa |  | 0.0001 | 1.50 | 1.55 | 1.40 | 17.92 | 0.753688 | 17.98 | 0.767262 | 18.52 | 0.578195 | 1.5 | 18.53 | 0.574149 | 1.5 | 18.54 | 0.539619 | 1.6 | 18.51 | 0.558131 | 1.5 | 18.41 | 0.529033 | 1.4 | 18.39 | 0.593345 | 1.4 |
| 630 | Glutaminyl-peptide cyclotransferase OS=Homo sapiens GN=QPCT PE=1 SV=1 | QPCT_HUMAN | 41 kDa |  | 0.00053 | 1.30 | 1.55 | 1.40 | 18.2 | 0.652094 | 18.27 | 0.549059 | 18.57 | 0.606919 | 1.3 | 18.64 | 0.583991 | 1.3 | 18.77 | 0.572056 | 1.5 | 18.92 | 0.573455 | 1.6 | 18.64 | 0.523039 | 1.4 | 18.81 | 0.585113 | 1.4 |
| 1179 | Dipeptidyl peptidase 3 OS=Homo sapiens GN=DPP3 PE=1 SV=2 | DPP3_HUMAN | 83 kDa |  | 0.00094 | 1.65 | 1.60 | 1.40 | 17.18 | 0.654505 | 17.21 | 0.645648 | 17.8 | 0.582323 | 1.6 | 17.9 | 0.614803 | 1.7 | 17.85 | 0.499447 | 1.6 | 17.88 | 0.513531 | 1.6 | 17.68 | 0.497719 | 1.4 | 17.68 | 0.497743 | 1.4 |
| 294 | Actin-related protein 2/3 complex subunit 1B OS=Homo sapiens GN=ARPC1B PE=1 SV=3 | ARC1B_HUMAN | 41 kDa | TRUE | 0.0001 | 1.60 | 1.60 | 1.40 | 17.81 | 0.865539 | 17.83 | 0.903911 | 18.44 | 0.736983 | 1.6 | 18.47 | 0.755037 | 1.6 | 18.52 | 0.617646 | 1.7 | 18.37 | 0.569052 | 1.5 | 18.33 | 0.582383 | 1.5 | 18.2 | 0.618537 | 1.3 |
| 338 | Adenylosuccinate synthetase isozyme 2 OS=Homo sapiens GN=ADSS PE=1 SV=3 | PURA2_HUMAN | 50 kDa |  | 0.0001 | 1.15 | 1.75 | 1.40 | 17.46 | 0.796689 | 17.43 | 0.784733 | 17.67 | 0.551562 | 1.1 | 17.62 | 0.542533 | 1.2 | 18.26 | 0.637735 | 1.8 | 18.15 | 0.623829 | 1.7 | 17.88 | 0.567536 | 1.4 | 17.87 | 0.561951 | 1.4 |
| 1197 | Myosin light chain 4 OS=Homo sapiens GN=MYL4 PE=1 SV=3 | MYL4_HUMAN | 22 kDa |  | 0.00017 | 1.50 | 1.80 | 1.40 | 17.71 | 0.754315 | 17.71 | 0.818107 | 18.2 | 0.525733 | 1.4 | 18.35 | 0.560393 | 1.6 | 18.58 | 0.540248 | 1.8 | 18.57 | 0.506637 | 1.8 | 18.19 | 0.503401 | 1.4 | 18.15 | 0.502022 | 1.4 |
| 893 | Annexin A3 OS=Homo sapiens GN=ANXA3 PE=1 SV=3 | ANXA3_HUMAN | 36 kDa | TRUE | 0.0001 | 1.65 | 2.10 | 1.40 | 18 | 0.682955 | 17.97 | 0.65899 | 18.7 | 0.565063 | 1.7 | 18.67 | 0.510519 | 1.6 | 19.07 | 0.503673 | 2.1 | 19.06 | 0.517604 | 2.1 | 18.42 | 0.512511 | 1.4 | 18.47 | 0.545978 | 1.4 |
| 678 | Platelet factor 4 OS=Homo sapiens GN=PF4 PE=1 SV=2 | PLF4_HUMAN | 11 kDa |  | 0.0016 | 1.00 | -1.11 | 1.45 | 18.5 | 0.568195 | 18.49 | 0.574382 | 18.48 | 0.642673 | 1 | 18.49 | 0.637446 | 1 | 18.43 | 0.535433 | 1 | 18.24 | 0.564209 | 0.8 | 19.1 | 0.608793 | 1.5 | 19 | 0.612157 | 1.4 |
| 925 | 60S ribosomal protein L7 OS=Homo sapiens GN=RPL7 PE=1 SV=1 | RL7_HUMAN | 29 kDa |  | 0.00035 | 1.10 | 1.15 | 1.45 | 17.94 | 0.672176 | 17.81 | 0.632553 | 17.92 | 0.538322 | 1 | 18.04 | 0.569938 | 1.2 | 18.09 | 0.537676 | 1.1 | 18.03 | 0.503062 | 1.2 | 18.45 | 0.520175 | 1.4 | 18.43 | 0.582129 | 1.5 |
| 1241 | Phosphoacetylglucosamine mutase OS=Homo sapiens GN=PGM3 PE=1 SV=1 | AGM1_HUMAN | 60 kDa |  | 0.014 | 1.00 | 1.15 | 1.45 | 18.55 | 0.812105 | 18.54 | 0.784079 | 18.48 | 0.52925 | 1 | 18.58 | 0.518503 | 1 | 18.7 | 0.567923 | 1.1 | 18.85 | 0.616833 | 1.2 | 19.05 | 0.622443 | 1.4 | 19.14 | 0.621776 | 1.5 |
| 312 | Interleukin enhancer-binding factor 3 OS=Homo sapiens GN=ILF3 PE=1 SV=3 | ILF3_HUMAN | 95 kDa | TRUE | 0.0001 | 1.20 | 1.25 | 1.45 | 17.89 | 0.83159 | 17.91 | 0.803701 | 18.19 | 0.533023 | 1.2 | 18.15 | 0.534044 | 1.2 | 18.23 | 0.554086 | 1.3 | 18.24 | 0.539389 | 1.2 | 18.42 | 0.56181 | 1.5 | 18.38 | 0.556664 | 1.4 |
| 993 | Transcription factor BTF3 OS=Homo sapiens GN=BTF3 PE=1 SV=1 | BTF3_HUMAN | 22 kDa | TRUE | 0.006 | 1.05 | 1.25 | 1.45 | 18.45 | 0.658213 | 18.56 | 0.786458 | 18.45 | 0.594 | 1 | 18.61 | 0.626033 | 1.1 | 18.71 | 0.515143 | 1.2 | 18.82 | 0.588228 | 1.3 | 18.98 | 0.527935 | 1.4 | 19.08 | 0.558644 | 1.5 |
| 297 | DNA-dependent protein kinase catalytic subunit OS=Homo sapiens GN=PRKDC PE=1 SV=3 | PRKDC_HUMAN | 469 kDa | TRUE | 0.0001 | 1.60 | 1.30 | 1.45 | 16.99 | 0.679006 | 16.92 | 0.644841 | 17.61 | 0.594677 | 1.6 | 17.58 | 0.587085 | 1.6 | 17.29 | 0.505925 | 1.2 | 17.38 | 0.50864 | 1.4 | 17.48 | 0.510101 | 1.4 | 17.48 | 0.528249 | 1.5 |
| 722 | Coatomer subunit epsilon OS=Homo sapiens GN=COPE PE=1 SV=3 | COPE_HUMAN | 34 kDa | TRUE | 0.00071 | 1.30 | 1.30 | 1.45 | 17.17 | 0.86912 | 17.26 | 0.67883 | 17.56 | 0.53854 | 1.3 | 17.61 | 0.559078 | 1.3 | 17.56 | 0.501057 | 1.4 | 17.57 | 0.580048 | 1.2 | 17.76 | 0.515227 | 1.5 | 17.78 | 0.524884 | 1.4 |
| 686 | Serpin B6 OS=Homo sapiens GN=SERPINB6 PE=1 SV=3 | SPB6_HUMAN | 43 kDa | TRUE | 0.012 | 1.30 | 1.30 | 1.45 | 17.67 | 0.74101 | 17.79 | 0.653341 | 18.15 | 0.540101 | 1.4 | 18.08 | 0.521006 | 1.2 | 18.15 | 0.519982 | 1.4 | 18.02 | 0.519489 | 1.2 | 18.21 | 0.522452 | 1.5 | 18.23 | 0.592146 | 1.4 |
| 516 | Eukaryotic peptide chain release factor GTP-binding subunit ERF3A OS=Homo sapiens GN=GSPT1 PE=1 SV=1 | ERF3A_HUMAN | 56 kDa |  | 0.011 | 1.25 | 1.30 | 1.45 | 18.14 | 0.714253 | 18.17 | 0.683676 | 18.47 | 0.549707 | 1.3 | 18.34 | 0.643112 | 1.2 | 18.59 | 0.545116 | 1.4 | 18.4 | 0.53084 | 1.2 | 18.67 | 0.573635 | 1.5 | 18.67 | 0.619839 | 1.4 |
| 311 | Heterogeneous nuclear ribonucleoprotein L OS=Homo sapiens GN=HNRNPL PE=1 SV=2 | HNRPL_HUMAN | 64 kDa |  | 0.00041 | 1.20 | 1.30 | 1.45 | 17.75 | 0.857167 | 17.58 | 0.796995 | 17.93 | 0.550067 | 1.1 | 17.97 | 0.552559 | 1.3 | 17.97 | 0.529584 | 1.2 | 17.99 | 0.540983 | 1.4 | 18.19 | 0.545676 | 1.4 | 18.16 | 0.56522 | 1.5 |
| 929 | SUMO-activating enzyme subunit 2 OS=Homo sapiens GN=UBA2 PE=1 SV=2 | SAE2_HUMAN | 71 kDa |  | 0.00056 | 1.00 | 1.30 | 1.45 | 17.47 | 0.664372 | 17.43 | 0.641867 | 17.45 | 0.524483 | 1 | 17.41 | 0.55994 | 1 | 17.73 | 0.525168 | 1.2 | 17.87 | 0.529542 | 1.4 | 17.96 | 0.511717 | 1.4 | 17.98 | 0.500065 | 1.5 |
| 387 | Ribose-phosphate pyrophosphokinase 1 OS=Homo sapiens GN=PRPS1 PE=1 SV=2 | PRPS1_HUMAN | 35 kDa | TRUE | 0.0017 | 1.75 | 1.30 | 1.45 | 17.16 | 1.073477 | 17.14 | 0.833312 | 17.98 | 0.621445 | 1.7 | 18.04 | 0.581388 | 1.8 | 17.54 | 0.539088 | 1.3 | 17.53 | 0.515124 | 1.3 | 17.76 | 0.531315 | 1.5 | 17.57 | 0.628574 | 1.4 |
| 1516 | Programmed cell death protein 10 OS=Homo sapiens GN=PDCD10 PE=1 SV=1 | PDC10_HUMAN | 25 kDa |  | 0.01 | 1.50 | 1.30 | 1.45 | 18.38 | 0.626798 | 18.51 | 0.549988 | 19.02 | 0.523229 | 1.5 | 19.12 | 0.530155 | 1.5 | 18.75 | 0.522066 | 1.3 | 18.9 | 0.501138 | 1.3 | 19 | 0.520368 | 1.5 | 18.95 | 0.506751 | 1.4 |
| 540 | Glutathione peroxidase 1 OS=Homo sapiens GN=GPX1 PE=1 SV=4 | GPX1_HUMAN | 22 kDa |  | 0.0006 | 1.30 | 1.30 | 1.45 | 18.2 | 0.716951 | 18.18 | 0.665372 | 18.56 | 0.563239 | 1.3 | 18.53 | 0.605457 | 1.3 | 18.55 | 0.519691 | 1.3 | 18.49 | 0.580982 | 1.3 | 18.72 | 0.537662 | 1.5 | 18.68 | 0.55901 | 1.4 |
| 216 | Protein disulfide-isomerase A4 OS=Homo sapiens GN=PDIA4 PE=1 SV=2 | PDIA4_HUMAN | 73 kDa |  | 0.0001 | 1.20 | 1.30 | 1.45 | 18.18 | 0.681334 | 18.09 | 0.687197 | 18.4 | 0.586895 | 1.2 | 18.35 | 0.547713 | 1.2 | 18.6 | 0.549936 | 1.3 | 18.5 | 0.553521 | 1.3 | 18.65 | 0.537913 | 1.4 | 18.62 | 0.548457 | 1.5 |
| 448 | Heterogeneous nuclear ribonucleoprotein M OS=Homo sapiens GN=HNRNPM PE=1 SV=3 | HNRPM_HUMAN | 78 kDa |  | 0.0001 | 1.20 | 1.30 | 1.45 | 18.15 | 0.745506 | 18.18 | 0.883596 | 18.41 | 0.570908 | 1.2 | 18.35 | 0.560211 | 1.2 | 18.53 | 0.571124 | 1.3 | 18.46 | 0.546677 | 1.3 | 18.62 | 0.547867 | 1.4 | 18.7 | 0.553921 | 1.5 |
| 320 | ATP-dependent RNA helicase A OS=Homo sapiens GN=DHX9 PE=1 SV=4 | DHX9_HUMAN | 141 kDa |  | 0.0001 | 1.55 | 1.35 | 1.45 | 17.48 | 0.822035 | 17.42 | 0.714132 | 18.1 | 0.616503 | 1.6 | 18.04 | 0.602055 | 1.5 | 17.81 | 0.522334 | 1.3 | 17.88 | 0.523374 | 1.4 | 17.94 | 0.523623 | 1.4 | 17.97 | 0.597021 | 1.5 |
| 984 | V-type proton ATPase subunit C 1 OS=Homo sapiens GN=ATP6V1C1 PE=1 SV=4 | VATC1_HUMAN | 44 kDa |  | 0.029 | 1.55 | 1.35 | 1.45 | 18.07 | 0.795709 | 18.09 | 0.778429 | 18.75 | 0.557791 | 1.5 | 18.8 | 0.557633 | 1.6 | 18.5 | 0.545408 | 1.3 | 18.56 | 0.503994 | 1.4 | 18.55 | 0.503543 | 1.4 | 18.62 | 0.523623 | 1.5 |
| 132 | Clathrin heavy chain 1 OS=Homo sapiens GN=CLTC PE=1 SV=5 | CLH1_HUMAN | 192 kDa | TRUE | 0.0001 | 1.50 | 1.35 | 1.45 | 17.79 | 0.799267 | 17.81 | 0.809106 | 18.4 | 0.642941 | 1.5 | 18.41 | 0.641251 | 1.5 | 18.12 | 0.567368 | 1.3 | 18.21 | 0.534247 | 1.4 | 18.25 | 0.534052 | 1.4 | 18.37 | 0.640712 | 1.5 |
| 879 | Small glutamine-rich tetratricopeptide repeat-containing protein alpha OS=Homo sapiens GN=SGTA PE=1 SV=1 | SGTA_HUMAN | 34 kDa | TRUE | 0.023 | 1.45 | 1.35 | 1.45 | 17.77 | 0.986898 | 17.89 | 0.8232 | 18.34 | 0.568161 | 1.5 | 18.33 | 0.58715 | 1.4 | 18.25 | 0.52582 | 1.4 | 18.29 | 0.531655 | 1.3 | 18.4 | 0.52724 | 1.5 | 18.44 | 0.516602 | 1.4 |
| 402 | Ubiquitin-like modifier-activating enzyme 6 OS=Homo sapiens GN=UBA6 PE=1 SV=1 | UBA6_HUMAN | 118 kDa |  | 0.0001 | 1.40 | 1.35 | 1.45 | 17.68 | 0.713235 | 17.61 | 0.75558 | 18.11 | 0.562618 | 1.4 | 18.1 | 0.552189 | 1.4 | 18.12 | 0.520681 | 1.3 | 18.06 | 0.561711 | 1.4 | 18.13 | 0.526209 | 1.4 | 18.13 | 0.541051 | 1.5 |
| 517 | Hypoxanthine-guanine phosphoribosyltransferase OS=Homo sapiens GN=HPRT1 PE=1 SV=2 | HPRT_HUMAN | 25 kDa |  | 0.0001 | 1.40 | 1.35 | 1.45 | 17.48 | 0.744625 | 17.45 | 0.661514 | 17.87 | 0.605605 | 1.4 | 17.94 | 0.589439 | 1.4 | 17.95 | 0.509012 | 1.4 | 17.85 | 0.529321 | 1.3 | 17.93 | 0.506858 | 1.4 | 17.98 | 0.519517 | 1.5 |
| 531 | Rho guanine nucleotide exchange factor 1 OS=Homo sapiens GN=ARHGEF1 PE=1 SV=2 | ARHG1_HUMAN | 102 kDa | TRUE | 0.00015 | 1.35 | 1.35 | 1.45 | 17.61 | 0.862154 | 17.74 | 0.86157 | 18.05 | 0.557961 | 1.4 | 18.06 | 0.54691 | 1.3 | 18.1 | 0.531732 | 1.4 | 18.08 | 0.53932 | 1.3 | 18.21 | 0.522259 | 1.5 | 18.17 | 0.535613 | 1.4 |
| 332 | Glucosidase 2 subunit beta OS=Homo sapiens GN=PRKCSH PE=1 SV=2 | GLU2B_HUMAN | 59 kDa | TRUE | 0.0019 | 1.20 | 1.35 | 1.45 | 17.75 | 0.788834 | 17.8 | 0.7648 | 18.03 | 0.551726 | 1.2 | 18.06 | 0.542674 | 1.2 | 18.28 | 0.631704 | 1.4 | 18.16 | 0.534729 | 1.3 | 18.28 | 0.648722 | 1.5 | 18.28 | 0.596266 | 1.4 |
| 268 | Fermitin family homolog 3 OS=Homo sapiens GN=FERMT3 PE=1 SV=1 | URP2_HUMAN | 76 kDa |  | 0.0001 | 1.70 | 1.40 | 1.45 | 18.19 | 0.814207 | 18.28 | 0.794653 | 18.97 | 0.607365 | 1.8 | 18.98 | 0.627088 | 1.6 | 18.64 | 0.517146 | 1.4 | 18.64 | 0.536851 | 1.4 | 18.7 | 0.5305 | 1.5 | 18.7 | 0.624482 | 1.4 |
| 899 | DnaJ homolog subfamily A member 2 OS=Homo sapiens GN=DNAJA2 PE=1 SV=1 | DNJA2_HUMAN | 46 kDa |  | 0.012 | 1.65 | 1.40 | 1.45 | 16.94 | 0.670312 | 16.76 | 0.726919 | 17.55 | 0.507797 | 1.6 | 17.52 | 0.521833 | 1.7 | 17.2 | 0.507523 | 1.2 | 17.45 | 0.641667 | 1.6 | 17.44 | 0.500182 | 1.4 | 17.37 | 0.505066 | 1.5 |
| 568 | Exportin-2 OS=Homo sapiens GN=CSE1L PE=1 SV=3 | XPO2_HUMAN | 110 kDa |  | 0.011 | 1.55 | 1.40 | 1.45 | 17.75 | 0.852207 | 17.69 | 0.822247 | 18.3 | 0.562611 | 1.5 | 18.33 | 0.584178 | 1.6 | 18.16 | 0.50586 | 1.3 | 18.24 | 0.507239 | 1.5 | 18.23 | 0.503556 | 1.4 | 18.29 | 0.521762 | 1.5 |
| 665 | Chromobox protein homolog 3 OS=Homo sapiens GN=CBX3 PE=1 SV=4 | CBX3_HUMAN | 21 kDa | TRUE | 0.0093 | 1.45 | 1.40 | 1.45 | 17.68 | 0.826511 | 17.64 | 0.721908 | 18.08 | 0.5208 | 1.4 | 18.18 | 0.563884 | 1.5 | 18.14 | 0.543866 | 1.4 | 18.03 | 0.527063 | 1.4 | 18.22 | 0.549438 | 1.5 | 18.08 | 0.559868 | 1.4 |
| 616 | Adipocyte enhancer-binding protein 1 OS=Homo sapiens GN=AEBP1 PE=1 SV=1 | AEBP1_HUMAN | 131 kDa | TRUE | 0.0001 | 1.40 | 1.40 | 1.45 | 18.53 | 0.728303 | 18.43 | 0.743716 | 18.93 | 0.591813 | 1.4 | 18.89 | 0.572731 | 1.4 | 18.88 | 0.538742 | 1.3 | 18.98 | 0.523936 | 1.5 | 18.99 | 0.531524 | 1.4 | 19.02 | 0.547575 | 1.5 |
| 1256 | ARF GTPase-activating protein GIT2 OS=Homo sapiens GN=GIT2 PE=1 SV=2 | GIT2_HUMAN | 85 kDa |  | 0.003 | 1.40 | 1.40 | 1.45 | 17.38 | 0.548844 | 17.28 | 0.513671 | 17.81 | 0.502706 | 1.3 | 17.82 | 0.506849 | 1.5 | 17.74 | 0.5106 | 1.3 | 17.86 | 0.504026 | 1.5 | 17.85 | 0.503888 | 1.4 | 17.89 | 0.491969 | 1.5 |
| 288 | Protein flightless-1 homolog OS=Homo sapiens GN=FLII PE=1 SV=2 | FLII_HUMAN | 145 kDa | TRUE | 0.0036 | 1.40 | 1.40 | 1.45 | 17.94 | 0.87249 | 17.95 | 0.81964 | 18.39 | 0.54158 | 1.4 | 18.39 | 0.53125 | 1.4 | 18.42 | 0.532292 | 1.4 | 18.39 | 0.522235 | 1.4 | 18.43 | 0.530944 | 1.4 | 18.45 | 0.555393 | 1.5 |
| 336 | Serine/threonine-protein phosphatase 2A catalytic subunit alpha isoform OS=Homo sapiens GN=PPP2CA PE=1 SV=1 | PP2AA_HUMAN | 36 kDa | TRUE | 0.0016 | 1.30 | 1.40 | 1.45 | 18.29 | 0.828768 | 18.36 | 0.817987 | 18.68 | 0.594338 | 1.3 | 18.71 | 0.596326 | 1.3 | 18.77 | 0.571148 | 1.4 | 18.85 | 0.5512 | 1.4 | 18.82 | 0.548618 | 1.5 | 18.87 | 0.658834 | 1.4 |
| 1050 | Apoptosis inhibitor 5 OS=Homo sapiens GN=API5 PE=1 SV=3 | API5_HUMAN | 59 kDa |  | 0.0061 | 1.30 | 1.40 | 1.45 | 17.57 | 0.680767 | 17.53 | 0.639585 | 17.92 | 0.515779 | 1.3 | 17.96 | 0.519571 | 1.3 | 17.94 | 0.50026 | 1.3 | 18.07 | 0.499499 | 1.5 | 18.12 | 0.515052 | 1.5 | 17.98 | 0.596549 | 1.4 |
| 273 | Kinesin-1 heavy chain OS=Homo sapiens GN=KIF5B PE=1 SV=1 | KINH_HUMAN | 110 kDa | TRUE | 0.0001 | 1.25 | 1.40 | 1.45 | 17.42 | 0.910118 | 17.29 | 0.733619 | 17.71 | 0.547268 | 1.2 | 17.72 | 0.542424 | 1.3 | 17.78 | 0.573831 | 1.3 | 17.86 | 0.514617 | 1.5 | 17.89 | 0.554996 | 1.4 | 17.86 | 0.611273 | 1.5 |
| 324 | Splicing factor, proline- and glutamine-rich OS=Homo sapiens GN=SFPQ PE=1 SV=2 | SFPQ_HUMAN | 76 kDa | TRUE | 0.0001 | 1.25 | 1.40 | 1.45 | 17.72 | 0.733713 | 17.52 | 0.645937 | 17.95 | 0.608207 | 1.1 | 17.99 | 0.600056 | 1.4 | 18.08 | 0.588971 | 1.3 | 18.07 | 0.563017 | 1.5 | 18.24 | 0.576339 | 1.4 | 18.12 | 0.588626 | 1.5 |
| 1483 | Eosinophil peroxidase OS=Homo sapiens GN=EPX PE=1 SV=2 | PERE_HUMAN | 81 kDa | TRUE | 0.013 | -1.25 | 1.40 | 1.45 | 18.26 | 0.525323 | 18.32 | 0.508412 | 17.96 | 0.504968 | 0.8 | 17.95 | 0.500777 | 0.8 | 18.7 | 0.511483 | 1.4 | 18.85 | 0.507621 | 1.4 | 18.78 | 0.49692 | 1.4 | 18.94 | 0.499625 | 1.5 |
| 858 | DCC-interacting protein 13-alpha OS=Homo sapiens GN=APPL1 PE=1 SV=1 | DP13A_HUMAN | 80 kDa |  | 0.0012 | 1.65 | 1.45 | 1.45 | 17.51 | 0.896424 | 17.61 | 0.783802 | 18.22 | 0.531193 | 1.7 | 18.28 | 0.547849 | 1.6 | 18.05 | 0.595202 | 1.4 | 18.19 | 0.520689 | 1.5 | 18 | 0.502294 | 1.4 | 18.17 | 0.587181 | 1.5 |
| 529 | Serine/arginine-rich splicing factor 1 OS=Homo sapiens GN=SRSF1 PE=1 SV=2 | SRSF1_HUMAN | 28 kDa | TRUE | 0.00094 | 1.55 | 1.45 | 1.45 | 18.28 | 0.705452 | 18.19 | 0.689287 | 18.82 | 0.618593 | 1.5 | 18.82 | 0.595769 | 1.6 | 18.76 | 0.534588 | 1.4 | 18.8 | 0.534779 | 1.5 | 18.72 | 0.518106 | 1.4 | 18.76 | 0.573545 | 1.5 |
| 206 | Tripeptidyl-peptidase 2 OS=Homo sapiens GN=TPP2 PE=1 SV=4 | TPP2_HUMAN | 138 kDa |  | 0.0001 | 1.50 | 1.45 | 1.45 | 17.72 | 0.810502 | 17.74 | 0.810712 | 18.37 | 0.572446 | 1.5 | 18.33 | 0.561704 | 1.5 | 18.29 | 0.542363 | 1.5 | 18.24 | 0.54885 | 1.4 | 18.23 | 0.53392 | 1.4 | 18.29 | 0.549319 | 1.5 |
| 793 | N-alpha-acetyltransferase 15, NatA auxiliary subunit OS=Homo sapiens GN=NAA15 PE=1 SV=1 | NAA15_HUMAN | 101 kDa |  | 0.0085 | 1.50 | 1.45 | 1.45 | 17.57 | 0.899135 | 17.54 | 0.720681 | 18.08 | 0.528525 | 1.5 | 18.1 | 0.505842 | 1.5 | 18.05 | 0.534954 | 1.4 | 18.07 | 0.601038 | 1.5 | 18.08 | 0.503976 | 1.5 | 18 | 0.53151 | 1.4 |
| 279 | Puromycin-sensitive aminopeptidase OS=Homo sapiens GN=NPEPPS PE=1 SV=2 | PSA_HUMAN | 103 kDa |  | 0.0001 | 1.45 | 1.45 | 1.45 | 17.88 | 0.773367 | 17.96 | 0.811737 | 18.44 | 0.574574 | 1.5 | 18.44 | 0.581355 | 1.4 | 18.48 | 0.520493 | 1.5 | 18.4 | 0.518861 | 1.4 | 18.4 | 0.519929 | 1.5 | 18.39 | 0.552003 | 1.4 |
| 658 | Transport and Golgi organization protein 2 homolog OS=Homo sapiens GN=TANGO2 PE=2 SV=1 | TNG2_HUMAN | 31 kDa |  | 0.00014 | 1.45 | 1.45 | 1.45 | 17.83 | 0.788145 | 17.77 | 0.770429 | 18.34 | 0.544624 | 1.4 | 18.34 | 0.539917 | 1.5 | 18.29 | 0.562297 | 1.4 | 18.35 | 0.567233 | 1.5 | 18.26 | 0.523666 | 1.4 | 18.27 | 0.583092 | 1.5 |
| 283 | Protein DDI1 homolog 2 OS=Homo sapiens GN=DDI2 PE=1 SV=1 | DDI2_HUMAN | 45 kDa |  | 0.0035 | 1.40 | 1.45 | 1.45 | 18.03 | 1.330152 | 17.92 | 1.210912 | 18.36 | 0.567922 | 1.4 | 18.29 | 0.576885 | 1.4 | 18.43 | 0.526697 | 1.4 | 18.42 | 0.537049 | 1.5 | 18.41 | 0.525239 | 1.4 | 18.41 | 0.562443 | 1.5 |
| 897 | COP9 signalosome complex subunit 8 OS=Homo sapiens GN=COPS8 PE=1 SV=1 | CSN8_HUMAN | 23 kDa |  | 0.00066 | 1.30 | 1.45 | 1.45 | 17.89 | 0.624295 | 17.82 | 0.918022 | 18.21 | 0.498921 | 1.2 | 18.24 | 0.510352 | 1.4 | 18.43 | 0.545875 | 1.4 | 18.35 | 0.49392 | 1.5 | 18.37 | 0.521239 | 1.4 | 18.27 | 0.554332 | 1.5 |
| 1340 | Phosphatidylinositol transfer protein beta isoform OS=Homo sapiens GN=PITPNB PE=1 SV=2 | PIPNB_HUMAN | 32 kDa | TRUE | 0.012 | 1.20 | 1.45 | 1.45 | 18.19 | 0.590089 | 18.11 | 0.711631 | 18.4 | 0.70796 | 1.2 | 18.3 | 0.583157 | 1.2 | 18.71 | 0.580728 | 1.5 | 18.57 | 0.548482 | 1.4 | 18.67 | 0.50343 | 1.4 | 18.67 | 0.527689 | 1.5 |
| 1445 | Mini-chromosome maintenance complex-binding protein OS=Homo sapiens GN=MCMBP PE=1 SV=2 | MCMBP_HUMAN | 73 kDa |  | 0.024 | 1.05 | 1.45 | 1.45 | 17.21 | 0.51184 | 17.33 | 0.503665 | 17.33 | 0.651341 | 1.1 | 17.37 | 0.674055 | 1 | 17.92 | 0.60031 | 1.6 | 17.68 | 0.491652 | 1.3 | 17.84 | 0.580793 | 1.5 | 17.77 | 0.483844 | 1.4 |
| 135 | EH domain-containing protein 1 OS=Homo sapiens GN=EHD1 PE=1 SV=2 | EHD1_HUMAN | 61 kDa | TRUE | 0.0001 | 1.70 | 1.50 | 1.45 | 17.62 | 0.800767 | 17.61 | 0.783763 | 18.27 | 0.622398 | 1.7 | 18.29 | 0.639069 | 1.7 | 18.17 | 0.564136 | 1.5 | 18.18 | 0.54746 | 1.5 | 18.14 | 0.533731 | 1.5 | 18.14 | 0.605854 | 1.4 |
| 953 | Nidogen-2 OS=Homo sapiens GN=NID2 PE=1 SV=3 | NID2_HUMAN | 151 kDa | TRUE | 0.0041 | 1.60 | 1.50 | 1.45 | 18.12 | 0.867962 | 18.14 | 0.784002 | 18.72 | 0.537578 | 1.6 | 18.71 | 0.564893 | 1.6 | 18.67 | 0.567513 | 1.5 | 18.69 | 0.537443 | 1.5 | 18.63 | 0.526971 | 1.4 | 18.68 | 0.541909 | 1.5 |
| 918 | Phosphatidylinositol 3-kinase regulatory subunit alpha OS=Homo sapiens GN=PIK3R1 PE=1 SV=2 | P85A_HUMAN | 84 kDa | TRUE | 0.0037 | 1.55 | 1.50 | 1.45 | 17.31 | 0.664419 | 17.31 | 0.63818 | 17.93 | 0.526263 | 1.5 | 17.99 | 0.532461 | 1.6 | 17.87 | 0.511565 | 1.5 | 17.92 | 0.499168 | 1.5 | 17.95 | 0.535653 | 1.5 | 17.82 | 0.540135 | 1.4 |
| 447 | Glia maturation factor gamma OS=Homo sapiens GN=GMFG PE=1 SV=1 | GMFG_HUMAN | 17 kDa | TRUE | 0.016 | 1.55 | 1.50 | 1.45 | 18.02 | 1.118846 | 17.95 | 1.169229 | 18.54 | 0.648887 | 1.5 | 18.54 | 0.633412 | 1.6 | 18.57 | 0.559211 | 1.5 | 18.48 | 0.552079 | 1.5 | 18.47 | 0.541792 | 1.4 | 18.41 | 0.579933 | 1.5 |
| 302 | Glucose-6-phosphate isomerase OS=Homo sapiens GN=GPI PE=1 SV=4 | G6PI_HUMAN | 63 kDa | TRUE | 0.00048 | 1.40 | 1.50 | 1.45 | 18.79 | 0.795762 | 18.88 | 0.713873 | 19.25 | 0.563461 | 1.4 | 19.31 | 0.569647 | 1.4 | 19.33 | 0.563323 | 1.5 | 19.39 | 0.618062 | 1.5 | 19.32 | 0.544108 | 1.5 | 19.33 | 0.549008 | 1.4 |
| 113 | Major vault protein OS=Homo sapiens GN=MVP PE=1 SV=4 | MVP_HUMAN | 99 kDa |  | 0.0001 | 1.80 | 1.55 | 1.45 | 17.49 | 0.88803 | 17.57 | 0.751589 | 18.37 | 0.629281 | 1.8 | 18.38 | 0.639618 | 1.8 | 18.14 | 0.559335 | 1.6 | 18.15 | 0.551468 | 1.5 | 17.97 | 0.531074 | 1.4 | 18.03 | 0.603192 | 1.5 |
| 417 | Proteasome subunit beta type-4 OS=Homo sapiens GN=PSMB4 PE=1 SV=4 | PSB4_HUMAN | 29 kDa | TRUE | 0.023 | 1.50 | 1.55 | 1.45 | 17.39 | 0.973463 | 17.42 | 0.947675 | 17.91 | 0.557772 | 1.5 | 17.89 | 0.572186 | 1.5 | 18.05 | 0.532081 | 1.6 | 18.02 | 0.557954 | 1.5 | 17.95 | 0.546038 | 1.5 | 17.97 | 0.565449 | 1.4 |
| 958 | Proteasome subunit beta type-9 OS=Homo sapiens GN=PSMB9 PE=1 SV=2 | PSB9_HUMAN | 23 kDa |  | 0.00023 | 1.30 | 1.55 | 1.45 | 18.38 | 0.759634 | 18.44 | 0.718608 | 18.72 | 0.52901 | 1.3 | 18.8 | 0.531795 | 1.3 | 18.9 | 0.512469 | 1.5 | 19.07 | 0.592078 | 1.6 | 18.86 | 0.525939 | 1.5 | 18.89 | 0.584862 | 1.4 |
| 1058 | C-type lectin domain family 11 member A OS=Homo sapiens GN=CLEC11A PE=1 SV=1 | CLC11_HUMAN | 36 kDa |  | 0.011 | 1.25 | 1.55 | 1.45 | 17.25 | 0.955076 | 17.25 | 0.977889 | 17.55 | 0.511099 | 1.2 | 17.63 | 0.52328 | 1.3 | 17.82 | 0.571252 | 1.5 | 17.95 | 0.509883 | 1.6 | 17.79 | 0.523654 | 1.4 | 17.81 | 0.576271 | 1.5 |
| 1265 | Mitogen-activated protein kinase 3 OS=Homo sapiens GN=MAPK3 PE=1 SV=4 | MK03_HUMAN | 43 kDa | TRUE | 0.0012 | 1.20 | 1.55 | 1.45 | 18.1 | 0.890012 | 18.15 | 0.907064 | 18.29 | 0.51391 | 1.2 | 18.35 | 0.51991 | 1.2 | 18.72 | 0.52473 | 1.6 | 18.71 | 0.514696 | 1.5 | 18.67 | 0.511117 | 1.5 | 18.63 | 0.514311 | 1.4 |
| 538 | Guanylate-binding protein 1 OS=Homo sapiens GN=GBP1 PE=1 SV=2 | GBP1_HUMAN | 68 kDa | TRUE | 0.0001 | 1.00 | 1.55 | 1.45 | 18.08 | 0.717066 | 18.06 | 0.801109 | 18.09 | 0.581266 | 1 | 18.09 | 0.55957 | 1 | 18.63 | 0.554514 | 1.5 | 18.73 | 0.544395 | 1.6 | 18.58 | 0.542534 | 1.4 | 18.58 | 0.577862 | 1.5 |
| 253 | Protein-arginine deiminase type-4 OS=Homo sapiens GN=PADI4 PE=1 SV=2 | PADI4_HUMAN | 74 kDa | TRUE | 0.0001 | 1.60 | 1.60 | 1.45 | 17.76 | 0.930356 | 17.88 | 0.936852 | 18.54 | 0.574543 | 1.7 | 18.52 | 0.581375 | 1.5 | 18.52 | 0.53352 | 1.7 | 18.44 | 0.533462 | 1.5 | 18.33 | 0.533444 | 1.5 | 18.35 | 0.634163 | 1.4 |
| 885 | Ubiquitin carboxyl-terminal hydrolase isozyme L3 OS=Homo sapiens GN=UCHL3 PE=1 SV=1 | UCHL3_HUMAN | 26 kDa |  | 0.0001 | 1.15 | 1.60 | 1.45 | 17.68 | 0.761111 | 17.71 | 0.787055 | 17.91 | 0.576593 | 1.2 | 17.92 | 0.610591 | 1.1 | 18.31 | 0.523584 | 1.6 | 18.32 | 0.565141 | 1.6 | 18.2 | 0.536167 | 1.5 | 18.15 | 0.569435 | 1.4 |
| 461 | Argininosuccinate lyase OS=Homo sapiens GN=ASL PE=1 SV=4 | ARLY_HUMAN | 52 kDa | TRUE | 0.0001 | 1.80 | 1.70 | 1.45 | 17.57 | 0.726115 | 17.54 | 0.782167 | 18.38 | 0.554449 | 1.8 | 18.35 | 0.554975 | 1.8 | 18.34 | 0.537919 | 1.7 | 18.28 | 0.517528 | 1.7 | 18.14 | 0.509852 | 1.5 | 18.07 | 0.564374 | 1.4 |
| 515 | Collagen alpha-1(V) chain OS=Homo sapiens GN=COL5A1 PE=1 SV=3 | CO5A1_HUMAN | 184 kDa |  | 0.0001 | 1.55 | 1.75 | 1.45 | 17.15 | 1.08268 | 17.25 | 1.062054 | 17.84 | 0.55554 | 1.6 | 17.9 | 0.618982 | 1.5 | 18.05 | 0.540259 | 1.8 | 17.96 | 0.54833 | 1.7 | 17.72 | 0.552553 | 1.5 | 17.62 | 0.697294 | 1.4 |
| 125 | Leukotriene A-4 hydrolase OS=Homo sapiens GN=LTA4H PE=1 SV=2 | LKHA4_HUMAN | 69 kDa | TRUE | 0.0001 | 1.65 | 1.80 | 1.45 | 17.99 | 0.77063 | 17.91 | 0.841824 | 18.63 | 0.695632 | 1.6 | 18.66 | 0.68444 | 1.7 | 18.75 | 0.60619 | 1.8 | 18.68 | 0.58481 | 1.8 | 18.45 | 0.53869 | 1.4 | 18.49 | 0.573679 | 1.5 |
| 75 | Glycogen phosphorylase, liver form OS=Homo sapiens GN=PYGL PE=1 SV=4 | PYGL_HUMAN | 97 kDa | TRUE | 0.0001 | 2.30 | 2.20 | 1.45 | 17.94 | 0.999148 | 17.95 | 1.053781 | 19.1 | 0.661579 | 2.3 | 19.11 | 0.663591 | 2.3 | 19.08 | 0.60843 | 2.3 | 18.91 | 0.564765 | 2.1 | 18.48 | 0.551927 | 1.5 | 18.4 | 0.617495 | 1.4 |
| 1586 | Eukaryotic translation initiation factor 4 gamma 2 OS=Homo sapiens GN=EIF4G2 PE=1 SV=1 | IF4G2_HUMAN | 102 kDa | TRUE | 0.026 | 1.00 | 1.25 | 1.45 | 17.2 | 0.509176 | 17.18 | 0.574266 | 17.2 | 0.550651 | 1 | 17.14 | 0.490568 | 1 | 17.41 | 0.502081 | 1.2 | 17.58 | 0.485384 | 1.3 | 17.64 | 0.495863 | 1.3 | 17.85 | 0.504036 | 1.6 |
| 1506 | Fructose-1,6-bisphosphatase 1 OS=Homo sapiens GN=FBP1 PE=1 SV=5 | F16P1_HUMAN | 37 kDa |  | 0.029 | -1.11 | 1.35 | 1.45 | 17.59 | 1.479403 | 17.32 | 1.070096 | 17.24 | 0.546202 | 0.9 | 17.13 | 0.488061 | 0.9 | 17.99 | 0.638225 | 1.3 | 17.87 | 0.545667 | 1.4 | 18.13 | 0.970247 | 1.3 | 18.14 | 0.869103 | 1.6 |
| 1033 | 60S ribosomal protein L9 OS=Homo sapiens GN=RPL9 PE=1 SV=1 | RL9_HUMAN | 22 kDa |  | 0.0037 | 1.65 | 1.40 | 1.45 | 16.66 | 0.821109 | 16.41 | 0.64337 | 17.16 | 0.61325 | 1.5 | 17.17 | 0.627724 | 1.8 | 16.84 | 0.516595 | 1.2 | 17.02 | 0.544235 | 1.6 | 17.02 | 0.497927 | 1.3 | 17.06 | 0.5141 | 1.6 |
| 1107 | Long-chain-fatty-acid--CoA ligase 4 OS=Homo sapiens GN=ACSL4 PE=1 SV=2 | ACSL4_HUMAN | 79 kDa |  | 0.024 | 1.35 | 1.40 | 1.45 | 16.98 | 0.78799 | 16.71 | 0.648759 | 17.29 | 0.524313 | 1.2 | 17.26 | 0.504329 | 1.5 | 17.32 | 0.484464 | 1.3 | 17.23 | 0.488797 | 1.5 | 17.37 | 0.497641 | 1.3 | 17.39 | 0.536145 | 1.6 |
| 1549 | Vacuolar protein sorting-associated protein 4A OS=Homo sapiens GN=VPS4A PE=1 SV=1 | VPS4A_HUMAN | 49 kDa | TRUE | 0.012 | 1.30 | 1.50 | 1.45 | 17.46 | 0.594998 | 17.26 | 0.562095 | 17.7 | 0.587299 | 1.2 | 17.77 | 0.612946 | 1.4 | 17.87 | 0.503636 | 1.3 | 17.99 | 0.523612 | 1.7 | 17.85 | 0.496308 | 1.3 | 17.91 | 0.499526 | 1.6 |
| 1188 | Creatine kinase B-type OS=Homo sapiens GN=CKB PE=1 SV=1 | KCRB_HUMAN | 43 kDa |  | 0.027 | 1.35 | 1.15 | 1.50 | 17.49 | 0.769781 | 17.6 | 0.716555 | 18.02 | 0.499011 | 1.5 | 17.89 | 0.520368 | 1.2 | 17.82 | 0.549987 | 1.2 | 17.73 | 0.495045 | 1.1 | 18.17 | 0.529256 | 1.6 | 18.09 | 0.525017 | 1.4 |
| 1148 | 40S ribosomal protein S8 OS=Homo sapiens GN=RPS8 PE=1 SV=2 | RS8_HUMAN | 24 kDa |  | 0.024 | 1.15 | 1.20 | 1.50 | 18.14 | 0.826491 | 18.18 | 0.79383 | 18.39 | 0.506649 | 1.2 | 18.35 | 0.503172 | 1.1 | 18.5 | 0.53508 | 1.3 | 18.32 | 0.512114 | 1.1 | 18.73 | 0.58254 | 1.5 | 18.75 | 0.607397 | 1.5 |
| 1343 | Rho GTPase-activating protein 17 OS=Homo sapiens GN=ARHGAP17 PE=1 SV=1 | RHG17_HUMAN | 95 kDa | TRUE | 0.013 | 1.30 | 1.25 | 1.50 | 17.15 | 0.708285 | 17.19 | 0.622768 | 17.41 | 0.495789 | 1.2 | 17.61 | 0.541418 | 1.4 | 17.43 | 0.494958 | 1.2 | 17.54 | 0.56023 | 1.3 | 17.68 | 0.510518 | 1.5 | 17.76 | 0.498121 | 1.5 |
| 1790 | [Protein ADP-ribosylarginine] hydrolase OS=Homo sapiens GN=ADPRH PE=1 SV=1 | ADPRH_HUMAN | 40 kDa |  | 0.026 | 1.10 | 1.25 | 1.50 | 17.5 | 0.517347 | 17.24 | 0.570646 | 17.43 | 0.482658 | 1 | 17.43 | 0.512439 | 1.2 | 17.6 | 0.493534 | 1.1 | 17.74 | 0.512411 | 1.4 | 17.85 | 0.53649 | 1.3 | 17.96 | 0.502932 | 1.7 |
| 1258 | Heterogeneous nuclear ribonucleoprotein H3 OS=Homo sapiens GN=HNRNPH3 PE=1 SV=2 | HNRH3_HUMAN | 37 kDa | TRUE | 0.00021 | 1.35 | 1.30 | 1.50 | 18.54 | 0.607637 | 18.7 | 0.667515 | 19.02 | 0.527535 | 1.4 | 19.08 | 0.523561 | 1.3 | 19.03 | 0.537467 | 1.4 | 19.01 | 0.536696 | 1.2 | 19.21 | 0.529867 | 1.6 | 19.22 | 0.563594 | 1.4 |
| 1277 | Ras-related protein Rab-31 OS=Homo sapiens GN=RAB31 PE=1 SV=1 | RAB31_HUMAN | 22 kDa |  | 0.0001 | 1.20 | 1.30 | 1.50 | 18.16 | 0.595859 | 18.28 | 0.542114 | 18.42 | 0.528836 | 1.2 | 18.47 | 0.530335 | 1.2 | 18.63 | 0.506982 | 1.4 | 18.56 | 0.513254 | 1.2 | 18.72 | 0.509312 | 1.5 | 18.82 | 0.525393 | 1.5 |
| 262 | Vesicle-fusing ATPase OS=Homo sapiens GN=NSF PE=1 SV=3 | NSF_HUMAN | 83 kDa | TRUE | 0.0001 | 1.40 | 1.30 | 1.50 | 18.06 | 0.781016 | 18.06 | 0.78438 | 18.56 | 0.568574 | 1.4 | 18.56 | 0.558544 | 1.4 | 18.41 | 0.530817 | 1.3 | 18.44 | 0.530888 | 1.3 | 18.56 | 0.534859 | 1.5 | 18.59 | 0.565105 | 1.5 |
| 427 | Lamin-B2 OS=Homo sapiens GN=LMNB2 PE=1 SV=4 | LMNB2_HUMAN | 70 kDa | TRUE | 0.0001 | 1.35 | 1.30 | 1.50 | 17.81 | 0.637637 | 17.87 | 0.741837 | 18.2 | 0.521289 | 1.3 | 18.29 | 0.542581 | 1.4 | 18.21 | 0.502286 | 1.3 | 18.19 | 0.511599 | 1.3 | 18.43 | 0.518106 | 1.5 | 18.43 | 0.517999 | 1.5 |
| 1056 | Cell cycle and apoptosis regulator protein 2 OS=Homo sapiens GN=CCAR2 PE=1 SV=2 | CCAR2_HUMAN | 103 kDa |  | 0.0001 | 1.15 | 1.30 | 1.50 | 16.81 | 0.690708 | 16.85 | 0.648137 | 16.95 | 0.507205 | 1.1 | 17.08 | 0.54489 | 1.2 | 17.14 | 0.499161 | 1.3 | 17.19 | 0.507362 | 1.3 | 17.35 | 0.514122 | 1.5 | 17.43 | 0.500073 | 1.5 |
| 1439 | Tumor necrosis factor alpha-induced protein 2 OS=Homo sapiens GN=TNFAIP2 PE=2 SV=2 | TNAP2_HUMAN | 73 kDa |  | 0.0087 | 1.15 | 1.30 | 1.50 | 17.15 | 0.66132 | 17.19 | 0.737812 | 17.33 | 0.629323 | 1.2 | 17.27 | 0.57075 | 1.1 | 17.58 | 0.553562 | 1.3 | 17.61 | 0.498009 | 1.3 | 17.63 | 0.527552 | 1.4 | 17.89 | 0.608231 | 1.6 |
| 1303 | Spermidine synthase OS=Homo sapiens GN=SRM PE=1 SV=1 | SPEE_HUMAN | 34 kDa |  | 0.026 | 1.00 | 1.30 | 1.50 | 18.79 | 0.599691 | 18.85 | 0.537116 | 18.83 | 0.519916 | 1 | 18.83 | 0.517146 | 1 | 19.16 | 0.604792 | 1.3 | 19.2 | 0.511523 | 1.3 | 19.28 | 0.536809 | 1.4 | 19.53 | 0.550674 | 1.6 |
| 979 | TSC22 domain family protein 4 OS=Homo sapiens GN=TSC22D4 PE=1 SV=2 | T22D4_HUMAN | 41 kDa |  | 0.00026 | 1.40 | 1.35 | 1.50 | 17.97 | 0.621797 | 17.94 | 0.672541 | 18.37 | 0.5206 | 1.4 | 18.41 | 0.535269 | 1.4 | 18.27 | 0.529745 | 1.3 | 18.37 | 0.537211 | 1.4 | 18.56 | 0.604313 | 1.6 | 18.44 | 0.541967 | 1.4 |
| 583 | Sorting nexin-1 OS=Homo sapiens GN=SNX1 PE=1 SV=3 | SNX1_HUMAN | 59 kDa | TRUE | 0.0001 | 1.30 | 1.35 | 1.50 | 17.95 | 0.650412 | 18.06 | 0.620311 | 18.38 | 0.543364 | 1.3 | 18.42 | 0.525942 | 1.3 | 18.42 | 0.511797 | 1.4 | 18.43 | 0.529253 | 1.3 | 18.57 | 0.506553 | 1.5 | 18.65 | 0.52432 | 1.5 |
| 503 | Non-POU domain-containing octamer-binding protein OS=Homo sapiens GN=NONO PE=1 SV=4 | NONO_HUMAN | 54 kDa | TRUE | 0.022 | 1.30 | 1.35 | 1.50 | 18.45 | 0.762087 | 18.53 | 0.748225 | 18.75 | 0.574921 | 1.3 | 18.84 | 0.574891 | 1.3 | 18.87 | 0.593627 | 1.4 | 18.89 | 0.526468 | 1.3 | 18.99 | 0.556656 | 1.5 | 19.04 | 0.556802 | 1.5 |
| 826 | Serine protease HTRA1 OS=Homo sapiens GN=HTRA1 PE=1 SV=1 | HTRA1_HUMAN | 51 kDa | TRUE | 0.0001 | 1.30 | 1.35 | 1.50 | 18.64 | 0.656884 | 18.4 | 0.74344 | 18.86 | 0.606526 | 1.2 | 18.9 | 0.615128 | 1.4 | 18.96 | 0.52506 | 1.3 | 18.84 | 0.526287 | 1.4 | 19.06 | 0.507148 | 1.4 | 19.03 | 0.519766 | 1.6 |
| 227 | Dihydropyrimidinase-related protein 2 OS=Homo sapiens GN=DPYSL2 PE=1 SV=1 | DPYL2_HUMAN | 62 kDa | TRUE | 0.0001 | 1.25 | 1.35 | 1.50 | 17.57 | 0.890841 | 17.49 | 0.771173 | 17.77 | 0.536902 | 1.2 | 17.82 | 0.528938 | 1.3 | 17.91 | 0.541161 | 1.3 | 17.88 | 0.556352 | 1.4 | 18.12 | 0.579051 | 1.5 | 18.08 | 0.570788 | 1.5 |
| 1550 | Oxysterol-binding protein-related protein 8 OS=Homo sapiens GN=OSBPL8 PE=1 SV=3 | OSBL8_HUMAN | 101 kDa |  | 0.017 | 1.25 | 1.35 | 1.50 | 18.28 | 0.58396 | 18.26 | 0.589169 | 18.71 | 0.555133 | 1.3 | 18.57 | 0.509046 | 1.2 | 18.75 | 0.527254 | 1.4 | 18.62 | 0.513443 | 1.3 | 18.86 | 0.516916 | 1.5 | 18.82 | 0.559867 | 1.5 |
| 853 | Aflatoxin B1 aldehyde reductase member 2 OS=Homo sapiens GN=AKR7A2 PE=1 SV=3 | ARK72_HUMAN | 40 kDa | TRUE | 0.028 | 1.25 | 1.35 | 1.50 | 18.39 | 1.09385 | 18.46 | 0.882383 | 18.74 | 0.546258 | 1.3 | 18.73 | 0.541363 | 1.2 | 18.89 | 0.53509 | 1.4 | 18.84 | 0.530403 | 1.3 | 18.91 | 0.531278 | 1.5 | 19.03 | 0.559515 | 1.5 |
| 1799 | WD repeat-containing protein 26 OS=Homo sapiens GN=WDR26 PE=1 SV=3 | WDR26_HUMAN | 72 kDa |  | 0.019 | 1.20 | 1.35 | 1.50 | 18.01 | 0.557343 | 18.36 | 0.513871 | 18.48 | 0.493903 | 1.4 | 18.3 | 0.505855 | 1 | 18.49 | 0.493903 | 1.4 | 18.76 | 0.519031 | 1.3 | 18.74 | 0.54702 | 1.7 | 18.75 | 0.500951 | 1.3 |
| 952 | Nck-associated protein 1-like OS=Homo sapiens GN=NCKAP1L PE=1 SV=3 | NCKPL_HUMAN | 128 kDa |  | 0.00063 | 1.70 | 1.40 | 1.50 | 18.24 | 0.513304 | 18.29 | 0.528063 | 19.01 | 0.514749 | 1.7 | 19.09 | 0.518004 | 1.7 | 18.73 | 0.590847 | 1.4 | 18.78 | 0.513126 | 1.4 | 18.77 | 0.503879 | 1.4 | 18.93 | 0.594468 | 1.6 |
| 908 | IST1 homolog OS=Homo sapiens GN=IST1 PE=1 SV=1 | IST1_HUMAN | 40 kDa |  | 0.0059 | 1.65 | 1.40 | 1.50 | 16.74 | 0.710348 | 16.59 | 0.577027 | 17.37 | 0.576725 | 1.5 | 17.47 | 0.54922 | 1.8 | 17.14 | 0.524733 | 1.3 | 17.21 | 0.52211 | 1.5 | 17.25 | 0.496281 | 1.4 | 17.26 | 0.507078 | 1.6 |
| 143 | 26S proteasome non-ATPase regulatory subunit 2 OS=Homo sapiens GN=PSMD2 PE=1 SV=3 | PSMD2_HUMAN | 100 kDa | TRUE | 0.0001 | 1.55 | 1.40 | 1.50 | 17.78 | 0.781408 | 17.91 | 0.763234 | 18.36 | 0.57858 | 1.6 | 18.37 | 0.591978 | 1.5 | 18.31 | 0.593792 | 1.5 | 18.31 | 0.534048 | 1.3 | 18.36 | 0.573921 | 1.5 | 18.48 | 0.605991 | 1.5 |
| 1326 | Protein DEK OS=Homo sapiens GN=DEK PE=1 SV=1 | DEK_HUMAN | 43 kDa |  | 0.015 | 1.55 | 1.40 | 1.50 | 17.87 | 0.610236 | 17.81 | 0.619811 | 18.35 | 0.528857 | 1.4 | 18.58 | 0.549984 | 1.7 | 18.32 | 0.513256 | 1.4 | 18.35 | 0.510918 | 1.4 | 18.5 | 0.51839 | 1.5 | 18.43 | 0.551794 | 1.5 |
| 378 | Vacuolar protein sorting-associated protein 35 OS=Homo sapiens GN=VPS35 PE=1 SV=2 | VPS35_HUMAN | 92 kDa | TRUE | 0.0001 | 1.50 | 1.40 | 1.50 | 17.75 | 0.700898 | 17.75 | 0.710002 | 18.36 | 0.660111 | 1.5 | 18.39 | 0.686405 | 1.5 | 18.19 | 0.563495 | 1.4 | 18.23 | 0.536778 | 1.4 | 18.28 | 0.539565 | 1.5 | 18.35 | 0.573889 | 1.5 |
| 323 | 26S proteasome non-ATPase regulatory subunit 12 OS=Homo sapiens GN=PSMD12 PE=1 SV=3 | PSD12_HUMAN | 53 kDa |  | 0.00046 | 1.40 | 1.40 | 1.50 | 17.47 | 0.865037 | 17.42 | 0.869866 | 17.86 | 0.592839 | 1.4 | 17.89 | 0.567598 | 1.4 | 17.97 | 0.561414 | 1.4 | 17.9 | 0.541752 | 1.4 | 17.88 | 0.555504 | 1.4 | 18.02 | 0.605662 | 1.6 |
| 241 | 26S proteasome non-ATPase regulatory subunit 1 OS=Homo sapiens GN=PSMD1 PE=1 SV=2 | PSMD1_HUMAN | 106 kDa |  | 0.0001 | 1.35 | 1.40 | 1.50 | 17.48 | 0.775402 | 17.57 | 0.740235 | 17.89 | 0.55405 | 1.4 | 17.92 | 0.563751 | 1.3 | 17.98 | 0.570225 | 1.4 | 18 | 0.563856 | 1.4 | 18.05 | 0.540251 | 1.5 | 18.09 | 0.580572 | 1.5 |
| 419 | Alanine--tRNA ligase, cytoplasmic OS=Homo sapiens GN=AARS PE=1 SV=2 | SYAC_HUMAN | 107 kDa |  | 0.0001 | 1.35 | 1.40 | 1.50 | 18.31 | 0.685458 | 18.23 | 0.72435 | 18.68 | 0.562804 | 1.3 | 18.74 | 0.565344 | 1.4 | 18.71 | 0.553731 | 1.3 | 18.82 | 0.571506 | 1.5 | 18.81 | 0.554022 | 1.4 | 18.9 | 0.541652 | 1.6 |
| 432 | 26S proteasome non-ATPase regulatory subunit 6 OS=Homo sapiens GN=PSMD6 PE=1 SV=1 | PSMD6_HUMAN | 46 kDa |  | 0.0001 | 1.35 | 1.40 | 1.50 | 17.86 | 0.696925 | 17.8 | 0.806447 | 18.29 | 0.557472 | 1.3 | 18.24 | 0.536727 | 1.4 | 18.32 | 0.517659 | 1.4 | 18.29 | 0.512203 | 1.4 | 18.38 | 0.544065 | 1.4 | 18.43 | 0.511034 | 1.6 |
| 1355 | Hippocalcin-like protein 1 OS=Homo sapiens GN=HPCAL1 PE=1 SV=3 | HPCL1_HUMAN | 22 kDa |  | 0.018 | 1.30 | 1.40 | 1.50 | 17.7 | 1.075965 | 17.78 | 0.96314 | 18.07 | 0.513649 | 1.4 | 17.95 | 0.494936 | 1.2 | 18.2 | 0.501736 | 1.5 | 18.11 | 0.501395 | 1.3 | 18.35 | 0.556237 | 1.6 | 18.33 | 0.586436 | 1.4 |
| 597 | Keratin, type I cytoskeletal 16 OS=Homo sapiens GN=KRT16 PE=1 SV=4 | K1C16_HUMAN | 51 kDa | TRUE | 0.0001 | -1.18 | 1.40 | 1.50 | 17.33 | 0.760966 | 17.37 | 0.716852 | 17.01 | 0.550514 | 0.8 | 17.12 | 0.597249 | 0.9 | 17.79 | 0.667384 | 1.4 | 17.89 | 0.628783 | 1.4 | 17.97 | 0.581592 | 1.5 | 17.96 | 0.589005 | 1.5 |
| 326 | 14-3-3 protein eta OS=Homo sapiens GN=YWHAH PE=1 SV=4 | 1433F_HUMAN | 28 kDa | TRUE | 0.0001 | 1.80 | 1.45 | 1.50 | 18.14 | 0.78408 | 18.15 | 0.6544 | 18.93 | 0.742284 | 1.8 | 18.99 | 0.73167 | 1.8 | 18.74 | 0.553012 | 1.5 | 18.61 | 0.556691 | 1.4 | 18.7 | 0.551304 | 1.5 | 18.72 | 0.557188 | 1.5 |
| 142 | F-actin-capping protein subunit beta OS=Homo sapiens GN=CAPZB PE=1 SV=4 | CAPZB_HUMAN | 31 kDa |  | 0.0001 | 1.75 | 1.45 | 1.50 | 17.88 | 0.900088 | 17.92 | 0.806085 | 18.59 | 0.738038 | 1.8 | 18.6 | 0.764625 | 1.7 | 18.43 | 0.563446 | 1.5 | 18.42 | 0.546689 | 1.4 | 18.43 | 0.565466 | 1.5 | 18.46 | 0.592457 | 1.5 |
| 806 | Exportin-7 OS=Homo sapiens GN=XPO7 PE=1 SV=3 | XPO7_HUMAN | 124 kDa | TRUE | 0.00031 | 1.70 | 1.45 | 1.50 | 17.51 | 0.780885 | 17.57 | 0.781521 | 18.19 | 0.587619 | 1.7 | 18.25 | 0.586765 | 1.7 | 18.05 | 0.611624 | 1.4 | 18.21 | 0.615025 | 1.5 | 18.1 | 0.555369 | 1.5 | 18.18 | 0.540474 | 1.5 |
| 954 | Olfactomedin-like protein 3 OS=Homo sapiens GN=OLFML3 PE=2 SV=1 | OLFL3_HUMAN | 46 kDa |  | 0.028 | 1.30 | 1.45 | 1.50 | 17.69 | 0.78659 | 17.75 | 0.737953 | 18.04 | 0.613905 | 1.3 | 18.14 | 0.66527 | 1.3 | 18.23 | 0.526394 | 1.5 | 18.22 | 0.555911 | 1.4 | 18.29 | 0.534355 | 1.5 | 18.31 | 0.529856 | 1.5 |
| 1341 | Peroxiredoxin-4 OS=Homo sapiens GN=PRDX4 PE=1 SV=1 | PRDX4_HUMAN | 31 kDa | TRUE | 0.0015 | 1.20 | 1.45 | 1.50 | 17.82 | 0.526045 | 17.76 | 0.560694 | 18.02 | 0.499378 | 1.2 | 18 | 0.49747 | 1.2 | 18.31 | 0.539498 | 1.4 | 18.38 | 0.524536 | 1.5 | 18.37 | 0.504192 | 1.5 | 18.37 | 0.538103 | 1.5 |
| 733 | Metastasis-associated protein MTA2 OS=Homo sapiens GN=MTA2 PE=1 SV=1 | MTA2_HUMAN | 75 kDa |  | 0.004 | 1.15 | 1.45 | 1.50 | 17.14 | 0.57889 | 17.3 | 0.580267 | 17.34 | 0.588703 | 1.1 | 17.48 | 0.797917 | 1.2 | 17.72 | 0.533984 | 1.5 | 17.72 | 0.506651 | 1.4 | 17.83 | 0.511964 | 1.6 | 17.83 | 0.5086 | 1.4 |
| 1109 | Activator of 90 kDa heat shock protein ATPase homolog 1 OS=Homo sapiens GN=AHSA1 PE=1 SV=1 | AHSA1_HUMAN | 38 kDa | TRUE | 0.0067 | 1.55 | 1.45 | 1.50 | 16.95 | 0.517272 | 16.84 | 0.54763 | 17.5 | 0.517504 | 1.5 | 17.5 | 0.49666 | 1.6 | 17.29 | 0.500876 | 1.3 | 17.5 | 0.590475 | 1.6 | 17.41 | 0.49074 | 1.4 | 17.53 | 0.502604 | 1.6 |
| 699 | Inorganic pyrophosphatase OS=Homo sapiens GN=PPA1 PE=1 SV=2 | IPYR_HUMAN | 33 kDa |  | 0.01 | 1.35 | 1.45 | 1.50 | 18.02 | 0.921946 | 18.07 | 0.915878 | 18.5 | 0.534458 | 1.4 | 18.42 | 0.54357 | 1.3 | 18.7 | 0.553612 | 1.6 | 18.44 | 0.541259 | 1.3 | 18.62 | 0.548731 | 1.5 | 18.62 | 0.546767 | 1.5 |
| 1648 | Splicing factor 3B subunit 4 OS=Homo sapiens GN=SF3B4 PE=1 SV=1 | SF3B4_HUMAN | 44 kDa |  | 0.026 | 1.65 | 1.50 | 1.50 | 16.65 | 0.500362 | 16.53 | 0.488007 | 17.36 | 0.498125 | 1.6 | 17.31 | 0.491185 | 1.7 | 17.25 | 0.486421 | 1.5 | 17.07 | 0.490439 | 1.5 | 17.37 | 0.503006 | 1.7 | 16.9 | 0.496051 | 1.3 |
| 231 | Neutrophil cytosol factor 2 OS=Homo sapiens GN=NCF2 PE=1 SV=2 | NCF2_HUMAN | 60 kDa |  | 0.0001 | 1.50 | 1.50 | 1.50 | 17.97 | 0.846204 | 17.95 | 0.825321 | 18.53 | 0.555832 | 1.5 | 18.52 | 0.581145 | 1.5 | 18.55 | 0.545216 | 1.5 | 18.47 | 0.56276 | 1.5 | 18.48 | 0.544269 | 1.5 | 18.49 | 0.570396 | 1.5 |
| 610 | Tyrosine--tRNA ligase, cytoplasmic OS=Homo sapiens GN=YARS PE=1 SV=4 | SYYC_HUMAN | 59 kDa |  | 0.0001 | 1.40 | 1.50 | 1.50 | 18.04 | 0.831726 | 18.03 | 0.738789 | 18.5 | 0.553021 | 1.4 | 18.53 | 0.530294 | 1.4 | 18.57 | 0.539228 | 1.5 | 18.57 | 0.574699 | 1.5 | 18.59 | 0.509353 | 1.5 | 18.6 | 0.525635 | 1.5 |
| 293 | Zyxin OS=Homo sapiens GN=ZYX PE=1 SV=1 | ZYX_HUMAN | 61 kDa | TRUE | 0.00053 | 1.35 | 1.50 | 1.50 | 17.39 | 0.930438 | 17.32 | 0.874363 | 17.77 | 0.519636 | 1.3 | 17.75 | 0.524203 | 1.4 | 17.91 | 0.545496 | 1.5 | 17.84 | 0.515892 | 1.5 | 17.86 | 0.525161 | 1.4 | 17.92 | 0.532453 | 1.6 |
| 1376 | Inosine triphosphate pyrophosphatase OS=Homo sapiens GN=ITPA PE=1 SV=2 | ITPA_HUMAN | 21 kDa | TRUE | 0.022 | 1.10 | 1.50 | 1.50 | 16.95 | 0.589555 | 17.17 | 0.559362 | 17.16 | 0.504759 | 1.2 | 17.22 | 0.501285 | 1 | 17.68 | 0.544236 | 1.7 | 17.59 | 0.49552 | 1.3 | 17.6 | 0.531852 | 1.6 | 17.7 | 0.540697 | 1.4 |
| 403 | Protein argonaute-2 OS=Homo sapiens GN=AGO2 PE=1 SV=3 | AGO2_HUMAN | 97 kDa | TRUE | 0.00011 | 1.45 | 1.55 | 1.50 | 17.1 | 0.804812 | 17.3 | 0.71998 | 17.67 | 0.624109 | 1.5 | 17.72 | 0.610957 | 1.4 | 17.82 | 0.522942 | 1.7 | 17.71 | 0.500196 | 1.4 | 17.75 | 0.514621 | 1.6 | 17.74 | 0.662541 | 1.4 |
| 1392 | Clathrin light chain B OS=Homo sapiens GN=CLTB PE=1 SV=1 | CLCB_HUMAN | 25 kDa |  | 0.012 | 1.25 | 1.55 | 1.50 | 16.96 | 0.68747 | 17.16 | 0.874967 | 17.23 | 0.510914 | 1.2 | 17.5 | 0.642178 | 1.3 | 17.44 | 0.494813 | 1.4 | 17.85 | 1.036905 | 1.7 | 17.66 | 0.514133 | 1.6 | 17.73 | 0.506989 | 1.4 |
| 627 | Dual specificity mitogen-activated protein kinase kinase 3 OS=Homo sapiens GN=MAP2K3 PE=1 SV=2 | MP2K3_HUMAN | 39 kDa |  | 0.02 | 1.60 | 1.55 | 1.50 | 17.79 | 0.706686 | 17.77 | 0.697639 | 18.4 | 0.565942 | 1.5 | 18.44 | 0.620633 | 1.7 | 18.43 | 0.54179 | 1.6 | 18.39 | 0.531964 | 1.5 | 18.38 | 0.519056 | 1.5 | 18.41 | 0.514865 | 1.5 |
| 190 | Pyruvate kinase PKLR OS=Homo sapiens GN=PKLR PE=1 SV=2 | KPYR_HUMAN | 62 kDa | TRUE | 0.0001 | 1.40 | 1.55 | 1.50 | 17.78 | 0.973808 | 17.81 | 0.908882 | 18.25 | 0.596495 | 1.4 | 18.25 | 0.591842 | 1.4 | 18.35 | 0.558135 | 1.5 | 18.39 | 0.559847 | 1.6 | 18.28 | 0.547091 | 1.5 | 18.35 | 0.573746 | 1.5 |
| 146 | Lumican OS=Homo sapiens GN=LUM PE=1 SV=2 | LUM_HUMAN | 38 kDa |  | 0.0001 | 1.50 | 1.60 | 1.50 | 17.68 | 0.883084 | 17.7 | 0.762361 | 18.27 | 0.565166 | 1.5 | 18.31 | 0.559806 | 1.5 | 18.34 | 0.559395 | 1.6 | 18.31 | 0.57376 | 1.6 | 18.21 | 0.546811 | 1.5 | 18.29 | 0.584825 | 1.5 |
| 1733 | Ras-related protein Rab-4B OS=Homo sapiens GN=RAB4B PE=1 SV=1 | RAB4B_HUMAN | 24 kDa | TRUE | 0.011 | 1.45 | 1.65 | 1.50 | 16.98 | 0.4844 | 17.09 | 0.519384 | 17.51 | 0.48595 | 1.4 | 17.64 | 0.489757 | 1.5 | 17.71 | 0.482302 | 1.7 | 17.77 | 0.489077 | 1.6 | 17.53 | 0.483682 | 1.5 | 17.66 | 0.489255 | 1.5 |
| 716 | Acetyl-CoA acetyltransferase, cytosolic OS=Homo sapiens GN=ACAT2 PE=1 SV=2 | THIC_HUMAN | 41 kDa | TRUE | 0.0001 | 1.15 | 1.65 | 1.50 | 18 | 0.709333 | 18.11 | 0.786402 | 18.2 | 0.554948 | 1.2 | 18.2 | 0.569472 | 1.1 | 18.83 | 0.570608 | 1.8 | 18.73 | 0.577999 | 1.5 | 18.58 | 0.533469 | 1.5 | 18.68 | 0.536931 | 1.5 |
| 756 | ERO1-like protein alpha OS=Homo sapiens GN=ERO1A PE=1 SV=2 | ERO1A_HUMAN | 54 kDa | TRUE | 0.0001 | 1.35 | 1.70 | 1.50 | 17.82 | 0.764851 | 17.79 | 0.900683 | 18.18 | 0.521946 | 1.3 | 18.19 | 0.524751 | 1.4 | 18.62 | 0.554654 | 1.7 | 18.53 | 0.516054 | 1.7 | 18.36 | 0.525201 | 1.5 | 18.35 | 0.523432 | 1.5 |
| 876 | Ras-related protein Rab-8A OS=Homo sapiens GN=RAB8A PE=1 SV=1 | RAB8A_HUMAN | 24 kDa | TRUE | 0.012 | 1.60 | 1.70 | 1.50 | 18.27 | 0.549357 | 18.27 | 0.504842 | 18.91 | 0.511321 | 1.6 | 18.92 | 0.547665 | 1.6 | 19.08 | 0.640927 | 1.8 | 18.9 | 0.538292 | 1.6 | 18.87 | 0.511933 | 1.5 | 18.89 | 0.537776 | 1.5 |
| 892 | Annexin A1 OS=Homo sapiens GN=ANXA1 PE=1 SV=2 | ANXA1_HUMAN | 39 kDa | TRUE | 0.0029 | 1.55 | 1.75 | 1.50 | 17.37 | 1.068834 | 17.33 | 0.990557 | 17.96 | 0.51713 | 1.5 | 17.97 | 0.520542 | 1.6 | 18.16 | 0.515035 | 1.8 | 18.07 | 0.502205 | 1.7 | 17.91 | 0.507471 | 1.5 | 17.88 | 0.511728 | 1.5 |
| 252 | Keratin, type I cytoskeletal 14 OS=Homo sapiens GN=KRT14 PE=1 SV=4 | K1C14_HUMAN | 52 kDa | TRUE | 0.0001 | -1.25 | 1.75 | 1.50 | 17.08 | 0.694022 | 17.12 | 0.656067 | 16.79 | 0.562801 | 0.8 | 16.84 | 0.558544 | 0.8 | 17.98 | 0.714873 | 1.9 | 17.74 | 0.556233 | 1.6 | 17.72 | 0.544588 | 1.5 | 17.69 | 0.569865 | 1.5 |
| 613 | Cytosolic purine 5'-nucleotidase OS=Homo sapiens GN=NT5C2 PE=1 SV=1 | 5NTC_HUMAN | 65 kDa |  | 0.00024 | 2.00 | 1.80 | 1.50 | 16.95 | 0.967726 | 16.7 | 0.968023 | 17.8 | 0.599614 | 1.9 | 17.75 | 0.643841 | 2.1 | 17.67 | 0.524565 | 1.7 | 17.64 | 0.517997 | 1.9 | 17.36 | 0.520604 | 1.4 | 17.32 | 0.574167 | 1.6 |
| 1328 | Translation initiation factor eIF-2B subunit beta OS=Homo sapiens GN=EIF2B2 PE=1 SV=3 | EI2BB_HUMAN | 39 kDa |  | 0.016 | 1.35 | 1.25 | 1.55 | 16.43 | 0.535496 | 16.51 | 0.611814 | 16.9 | 0.49269 | 1.4 | 16.93 | 0.492203 | 1.3 | 16.78 | 0.515304 | 1.3 | 16.83 | 0.492472 | 1.2 | 17.19 | 0.495327 | 1.7 | 16.97 | 0.526804 | 1.4 |
| 1782 | Nucleolar protein 56 OS=Homo sapiens GN=NOP56 PE=1 SV=4 | NOP56_HUMAN | 66 kDa |  | 0.013 | 1.20 | 1.25 | 1.55 | 17.64 | 0.489048 | 17.49 | 0.500684 | 17.87 | 0.503208 | 1.2 | 17.8 | 0.49363 | 1.2 | 17.91 | 0.493612 | 1.2 | 17.9 | 0.499383 | 1.3 | 18.09 | 0.492618 | 1.4 | 18.21 | 0.491154 | 1.7 |
| 550 | 60S ribosomal protein L4 OS=Homo sapiens GN=RPL4 PE=1 SV=5 | RL4_HUMAN | 48 kDa | TRUE | 0.0001 | 1.45 | 1.30 | 1.55 | 18.36 | 0.719449 | 18.48 | 0.70562 | 18.81 | 0.59479 | 1.5 | 18.85 | 0.625941 | 1.4 | 18.82 | 0.538905 | 1.4 | 18.81 | 0.543218 | 1.2 | 19.13 | 0.604813 | 1.7 | 19.03 | 0.609105 | 1.4 |
| 1178 | DnaJ homolog subfamily A member 1 OS=Homo sapiens GN=DNAJA1 PE=1 SV=2 | DNJA1_HUMAN | 45 kDa | TRUE | 0.005 | 1.30 | 1.30 | 1.55 | 17.7 | 0.833516 | 17.46 | 0.638567 | 17.99 | 0.499923 | 1.2 | 17.95 | 0.49855 | 1.4 | 17.91 | 0.516213 | 1.2 | 17.88 | 0.503807 | 1.4 | 18.16 | 0.533544 | 1.4 | 18.22 | 0.516285 | 1.7 |
| 554 | SH3 domain-binding protein 1 OS=Homo sapiens GN=SH3BP1 PE=1 SV=3 | 3BP1_HUMAN | 76 kDa | TRUE | 0.0001 | 1.30 | 1.50 | 1.55 | 17.5 | 0.873501 | 17.32 | 0.946203 | 17.82 | 0.503195 | 1.2 | 17.78 | 0.509674 | 1.4 | 18 | 0.504704 | 1.4 | 17.98 | 0.517317 | 1.6 | 18.01 | 0.511694 | 1.4 | 18.09 | 0.538916 | 1.7 |
| 1135 | Galectin-7 OS=Homo sapiens GN=LGALS7 PE=1 SV=2 | LEG7_HUMAN | 15 kDa |  | 0.00049 | 1.05 | 1.50 | 1.55 | 17.92 | 1.085238 | 17.72 | 0.845626 | 17.9 | 0.532434 | 1 | 17.87 | 0.542603 | 1.1 | 18.33 | 0.539197 | 1.4 | 18.35 | 0.562997 | 1.6 | 18.47 | 0.544017 | 1.4 | 18.51 | 0.551688 | 1.7 |
| 1389 | 40S ribosomal protein S20 OS=Homo sapiens GN=RPS20 PE=1 SV=1 | RS20_HUMAN | 13 kDa |  | 0.019 | 1.30 | 1.20 | 1.55 | 18.36 | 1.020208 | 18.24 | 0.912414 | 18.63 | 0.526049 | 1.2 | 18.68 | 0.528525 | 1.4 | 18.58 | 0.538722 | 1.2 | 18.51 | 0.538094 | 1.2 | 18.88 | 0.520165 | 1.5 | 18.85 | 0.522133 | 1.6 |
| 1035 | Small nuclear ribonucleoprotein Sm D2 OS=Homo sapiens GN=SNRPD2 PE=1 SV=1 | SMD2_HUMAN | 14 kDa |  | 0.0001 | 1.25 | 1.25 | 1.55 | 18.14 | 0.587824 | 18.21 | 0.575664 | 18.47 | 0.529643 | 1.3 | 18.47 | 0.524914 | 1.2 | 18.51 | 0.527072 | 1.3 | 18.48 | 0.50793 | 1.2 | 18.75 | 0.532714 | 1.5 | 18.84 | 0.569524 | 1.6 |
| 356 | Signal transducer and activator of transcription 1-alpha/beta OS=Homo sapiens GN=STAT1 PE=1 SV=2 | STAT1_HUMAN | 87 kDa | TRUE | 0.0001 | 1.05 | 1.25 | 1.55 | 18.08 | 0.78248 | 18.06 | 0.775096 | 18.11 | 0.556276 | 1 | 18.15 | 0.574308 | 1.1 | 18.38 | 0.577102 | 1.2 | 18.4 | 0.535365 | 1.3 | 18.65 | 0.602069 | 1.5 | 18.72 | 0.593579 | 1.6 |
| 372 | 60S ribosomal protein L5 OS=Homo sapiens GN=RPL5 PE=1 SV=3 | RL5_HUMAN | 34 kDa |  | 0.0001 | 1.05 | 1.25 | 1.55 | 17.85 | 0.76904 | 17.98 | 0.851021 | 18.03 | 0.539733 | 1.1 | 18 | 0.541246 | 1 | 18.24 | 0.528761 | 1.3 | 18.21 | 0.562808 | 1.2 | 18.5 | 0.568035 | 1.6 | 18.56 | 0.544314 | 1.5 |
| 928 | 40S ribosomal protein S21 OS=Homo sapiens GN=RPS21 PE=1 SV=1 | RS21_HUMAN | 9 kDa |  | 0.0074 | 1.05 | 1.25 | 1.55 | 18.5 | 0.782837 | 18.54 | 0.707054 | 18.46 | 0.525324 | 1 | 18.6 | 0.511274 | 1.1 | 18.76 | 0.549984 | 1.2 | 18.84 | 0.539094 | 1.3 | 19.11 | 0.621579 | 1.6 | 19.09 | 0.595684 | 1.5 |
| 1150 | SUMO-activating enzyme subunit 1 OS=Homo sapiens GN=SAE1 PE=1 SV=1 | SAE1_HUMAN | 38 kDa |  | 0.0001 | -1.05 | 1.25 | 1.55 | 17.67 | 0.731339 | 17.61 | 0.686959 | 17.59 | 0.529284 | 0.9 | 17.66 | 0.581378 | 1 | 17.97 | 0.509737 | 1.2 | 18.04 | 0.501955 | 1.3 | 18.21 | 0.535941 | 1.5 | 18.28 | 0.564587 | 1.6 |
| 1496 | Dynein light chain 1, cytoplasmic OS=Homo sapiens GN=DYNLL1 PE=1 SV=1 | DYL1_HUMAN | 10 kDa |  | 0.026 | -1.05 | 1.25 | 1.55 | 17.99 | 0.637162 | 18.08 | 0.746957 | 17.91 | 0.538254 | 1 | 17.86 | 0.536215 | 0.9 | 18.47 | 0.555569 | 1.4 | 18.23 | 0.552916 | 1.1 | 18.64 | 0.598486 | 1.6 | 18.66 | 0.543225 | 1.5 |
| 835 | 40S ribosomal protein S6 OS=Homo sapiens GN=RPS6 PE=1 SV=1 | RS6_HUMAN | 29 kDa | TRUE | 0.0062 | 1.15 | 1.30 | 1.55 | 18.56 | 0.628018 | 18.48 | 0.589919 | 18.69 | 0.52697 | 1.1 | 18.75 | 0.542694 | 1.2 | 18.77 | 0.537417 | 1.2 | 18.96 | 0.569865 | 1.4 | 19.11 | 0.58187 | 1.5 | 19.19 | 0.63574 | 1.6 |
| 796 | 60S ribosomal protein L21 OS=Homo sapiens GN=RPL21 PE=1 SV=2 | RL21_HUMAN | 19 kDa |  | 0.0001 | 1.25 | 1.30 | 1.55 | 17.43 | 0.699485 | 17.49 | 0.637344 | 17.72 | 0.509642 | 1.3 | 17.73 | 0.516353 | 1.2 | 17.89 | 0.540622 | 1.3 | 17.87 | 0.517719 | 1.3 | 18.06 | 0.538189 | 1.5 | 18.15 | 0.541479 | 1.6 |
| 1846 | NHP2-like protein 1 OS=Homo sapiens GN=SNU13 PE=1 SV=3 | NH2L1_HUMAN | 14 kDa |  | 0.026 | 1.20 | 1.30 | 1.55 | 18 | 0.543392 | 18.15 | 0.519182 | 18.43 | 0.500263 | 1.3 | 18.29 | 0.497005 | 1.1 | 18.55 | 0.503433 | 1.5 | 18.33 | 0.497158 | 1.1 | 18.71 | 0.500183 | 1.6 | 18.73 | 0.548161 | 1.5 |
| 1189 | Putative RNA-binding protein Luc7-like 2 OS=Homo sapiens GN=LUC7L2 PE=1 SV=2 | LC7L2_HUMAN | 47 kDa | TRUE | 0.0001 | 1.00 | 1.30 | 1.55 | 18.45 | 0.528297 | 18.48 | 0.583752 | 18.39 | 0.532962 | 1 | 18.4 | 0.547063 | 1 | 18.84 | 0.564759 | 1.3 | 18.82 | 0.51029 | 1.3 | 19.11 | 0.564807 | 1.6 | 19.13 | 0.588849 | 1.5 |
| 997 | COP9 signalosome complex subunit 7b OS=Homo sapiens GN=COPS7B PE=1 SV=1 | CSN7B_HUMAN | 30 kDa |  | 0.0037 | 1.50 | 1.35 | 1.55 | 17.25 | 0.798 | 17.3 | 0.773416 | 17.84 | 0.518783 | 1.5 | 17.79 | 0.533993 | 1.5 | 17.68 | 0.505542 | 1.4 | 17.66 | 0.560587 | 1.3 | 17.87 | 0.567863 | 1.6 | 17.84 | 0.505511 | 1.5 |
| 1106 | ATP-binding cassette sub-family F member 1 OS=Homo sapiens GN=ABCF1 PE=1 SV=2 | ABCF1_HUMAN | 96 kDa | TRUE | 0.0001 | 1.10 | 1.35 | 1.55 | 17.29 | 0.704257 | 17.36 | 0.553021 | 17.43 | 0.528763 | 1.1 | 17.49 | 0.54605 | 1.1 | 17.68 | 0.498709 | 1.4 | 17.7 | 0.556909 | 1.3 | 17.87 | 0.502187 | 1.5 | 18 | 0.520947 | 1.6 |
| 308 | Bactericidal permeability-increasing protein OS=Homo sapiens GN=BPI PE=1 SV=4 | BPI_HUMAN | 54 kDa |  | 0.0001 | 1.65 | 1.40 | 1.55 | 18.05 | 1.11161 | 18.23 | 0.899506 | 18.88 | 0.623342 | 1.7 | 18.93 | 0.634907 | 1.6 | 18.56 | 0.591783 | 1.4 | 18.65 | 0.541277 | 1.4 | 18.56 | 0.541939 | 1.5 | 18.73 | 0.68929 | 1.6 |
| 114 | Endoplasmin OS=Homo sapiens GN=HSP90B1 PE=1 SV=1 | ENPL_HUMAN | 92 kDa | TRUE | 0.0001 | 1.50 | 1.40 | 1.55 | 17.81 | 0.816061 | 17.89 | 0.772335 | 18.41 | 0.605307 | 1.5 | 18.42 | 0.60127 | 1.5 | 18.39 | 0.565477 | 1.5 | 18.29 | 0.549759 | 1.3 | 18.49 | 0.546614 | 1.6 | 18.45 | 0.583543 | 1.5 |
| 611 | Thioredoxin-like protein 1 OS=Homo sapiens GN=TXNL1 PE=1 SV=3 | TXNL1_HUMAN | 32 kDa |  | 0.00023 | 1.00 | 1.40 | 1.55 | 17.57 | 1.015265 | 17.56 | 0.725905 | 17.63 | 0.680583 | 1 | 17.57 | 0.635436 | 1 | 18.07 | 0.587791 | 1.4 | 17.96 | 0.547043 | 1.4 | 18.18 | 0.60944 | 1.5 | 18.21 | 0.64851 | 1.6 |
| 469 | Protein phosphatase 1 regulatory subunit 7 OS=Homo sapiens GN=PPP1R7 PE=1 SV=1 | PP1R7_HUMAN | 42 kDa |  | 0.0026 | 1.40 | 1.45 | 1.55 | 17.08 | 0.969581 | 17.1 | 0.8694 | 17.54 | 0.537417 | 1.4 | 17.61 | 0.534127 | 1.4 | 17.61 | 0.540467 | 1.5 | 17.61 | 0.529378 | 1.4 | 17.63 | 0.518527 | 1.5 | 17.74 | 0.599098 | 1.6 |
| 213 | Dynactin subunit 2 OS=Homo sapiens GN=DCTN2 PE=1 SV=4 | DCTN2_HUMAN | 44 kDa | TRUE | 0.0001 | 1.35 | 1.45 | 1.55 | 17.83 | 0.858819 | 17.88 | 0.821547 | 18.25 | 0.600757 | 1.4 | 18.28 | 0.578095 | 1.3 | 18.4 | 0.527289 | 1.5 | 18.34 | 0.577702 | 1.4 | 18.39 | 0.529557 | 1.5 | 18.46 | 0.555721 | 1.6 |
| 435 | Septin-7 OS=Homo sapiens GN=SEPT7 PE=1 SV=2 | SEPT7_HUMAN | 51 kDa | TRUE | 0.0085 | 1.35 | 1.45 | 1.55 | 18.26 | 0.840988 | 18.35 | 0.743132 | 18.72 | 0.590871 | 1.4 | 18.74 | 0.565836 | 1.3 | 18.81 | 0.5995 | 1.5 | 18.75 | 0.538035 | 1.4 | 18.87 | 0.572476 | 1.6 | 18.9 | 0.588087 | 1.5 |
| 474 | General vesicular transport factor p115 OS=Homo sapiens GN=USO1 PE=1 SV=2 | USO1_HUMAN | 108 kDa | TRUE | 0.00056 | 1.20 | 1.45 | 1.55 | 18.39 | 0.90628 | 18.39 | 0.863386 | 18.66 | 0.558911 | 1.2 | 18.61 | 0.558436 | 1.2 | 18.84 | 0.551143 | 1.5 | 18.79 | 0.550002 | 1.4 | 18.88 | 0.524706 | 1.5 | 18.91 | 0.580643 | 1.6 |
| 141 | Cullin-associated NEDD8-dissociated protein 1 OS=Homo sapiens GN=CAND1 PE=1 SV=2 | CAND1_HUMAN | 136 kDa |  | 0.0001 | 1.55 | 1.50 | 1.55 | 17.53 | 0.971478 | 17.48 | 0.891437 | 18.08 | 0.568676 | 1.5 | 18.1 | 0.567635 | 1.6 | 18.1 | 0.537812 | 1.5 | 18.03 | 0.533551 | 1.5 | 18.08 | 0.526024 | 1.5 | 18.1 | 0.561353 | 1.6 |
| 767 | 60 kDa SS-A/Ro ribonucleoprotein OS=Homo sapiens GN=TROVE2 PE=1 SV=2 | RO60_HUMAN | 61 kDa |  | 0.0001 | 1.50 | 1.50 | 1.55 | 18.66 | 0.8003 | 18.61 | 0.706809 | 19.17 | 0.591555 | 1.5 | 19.14 | 0.584515 | 1.5 | 19.25 | 0.534133 | 1.5 | 19.19 | 0.531531 | 1.5 | 19.22 | 0.54912 | 1.5 | 19.29 | 0.595106 | 1.6 |
| 306 | Aldehyde dehydrogenase family 16 member A1 OS=Homo sapiens GN=ALDH16A1 PE=1 SV=2 | A16A1_HUMAN | 85 kDa |  | 0.00076 | 1.50 | 1.50 | 1.55 | 17.8 | 0.907918 | 17.96 | 0.854535 | 18.43 | 0.531787 | 1.6 | 18.4 | 0.528575 | 1.4 | 18.39 | 0.551738 | 1.5 | 18.49 | 0.541831 | 1.5 | 18.44 | 0.51516 | 1.6 | 18.46 | 0.630039 | 1.5 |
| 41 | Filamin-A OS=Homo sapiens GN=FLNA PE=1 SV=4 | FLNA_HUMAN | 281 kDa | TRUE | 0.0001 | 1.45 | 1.50 | 1.55 | 17.98 | 0.822306 | 18.06 | 0.824516 | 18.55 | 0.586649 | 1.5 | 18.55 | 0.58446 | 1.4 | 18.62 | 0.558388 | 1.6 | 18.52 | 0.554757 | 1.4 | 18.58 | 0.552904 | 1.6 | 18.58 | 0.559313 | 1.5 |
| 998 | EF-hand domain-containing protein D2 OS=Homo sapiens GN=EFHD2 PE=1 SV=1 | EFHD2_HUMAN | 27 kDa |  | 0.016 | 1.45 | 1.50 | 1.55 | 17.77 | 0.698231 | 17.9 | 0.735491 | 18.4 | 0.501049 | 1.5 | 18.41 | 0.505518 | 1.4 | 18.47 | 0.501121 | 1.6 | 18.37 | 0.504516 | 1.4 | 18.45 | 0.501418 | 1.6 | 18.46 | 0.518011 | 1.5 |
| 728 | Glutamate--cysteine ligase catalytic subunit OS=Homo sapiens GN=GCLC PE=1 SV=2 | GSH1_HUMAN | 73 kDa | TRUE | 0.00022 | 1.35 | 1.50 | 1.55 | 16.8 | 0.721925 | 16.73 | 0.75673 | 17.22 | 0.526062 | 1.3 | 17.23 | 0.523224 | 1.4 | 17.36 | 0.50398 | 1.5 | 17.34 | 0.546913 | 1.5 | 17.41 | 0.500977 | 1.6 | 17.32 | 0.566338 | 1.5 |
| 412 | Nucleoside diphosphate kinase B OS=Homo sapiens GN=NME2 PE=1 SV=1 | NDKB_HUMAN | 17 kDa | TRUE | 0.0019 | 1.35 | 1.50 | 1.55 | 18.16 | 0.801743 | 18.16 | 0.88097 | 18.5 | 0.668313 | 1.3 | 18.54 | 0.648504 | 1.4 | 18.72 | 0.542492 | 1.5 | 18.69 | 0.518523 | 1.5 | 18.84 | 0.54254 | 1.6 | 18.74 | 0.623959 | 1.5 |
| 298 | 26S protease regulatory subunit 10B OS=Homo sapiens GN=PSMC6 PE=1 SV=1 | PRS10_HUMAN | 44 kDa | TRUE | 0.0001 | 1.30 | 1.50 | 1.55 | 17.46 | 0.8023 | 17.45 | 0.746906 | 17.82 | 0.566507 | 1.3 | 17.83 | 0.600186 | 1.3 | 17.97 | 0.535438 | 1.5 | 18.05 | 0.543795 | 1.5 | 18.12 | 0.554545 | 1.6 | 18.08 | 0.537191 | 1.5 |
| 1002 | Heme-binding protein 1 OS=Homo sapiens GN=HEBP1 PE=1 SV=1 | HEBP1_HUMAN | 21 kDa |  | 0.0001 | 1.25 | 1.50 | 1.55 | 18.04 | 0.649215 | 18.03 | 0.607401 | 18.33 | 0.525614 | 1.2 | 18.42 | 0.557046 | 1.3 | 18.66 | 0.522158 | 1.5 | 18.61 | 0.50934 | 1.5 | 18.61 | 0.511315 | 1.5 | 18.65 | 0.535353 | 1.6 |
| 1546 | Nuclear distribution protein nudE homolog 1 OS=Homo sapiens GN=NDE1 PE=1 SV=2 | NDE1_HUMAN | 39 kDa |  | 0.026 | 1.40 | 1.55 | 1.55 | 16.03 | 0.504977 | 15.93 | 0.51047 | 16.45 | 0.479737 | 1.3 | 16.53 | 0.485588 | 1.5 | 16.5 | 0.481924 | 1.4 | 16.72 | 0.492226 | 1.7 | 16.69 | 0.481124 | 1.6 | 16.55 | 0.562975 | 1.5 |
| 153 | Protein diaphanous homolog 1 OS=Homo sapiens GN=DIAPH1 PE=1 SV=2 | DIAP1_HUMAN | 141 kDa |  | 0.0001 | 1.75 | 1.55 | 1.55 | 17.57 | 0.899118 | 17.53 | 0.837035 | 18.3 | 0.603688 | 1.7 | 18.31 | 0.596343 | 1.8 | 18.14 | 0.526153 | 1.5 | 18.18 | 0.538234 | 1.6 | 18.13 | 0.51191 | 1.5 | 18.17 | 0.561377 | 1.6 |
| 374 | Aspartate--tRNA ligase, cytoplasmic OS=Homo sapiens GN=DARS PE=1 SV=2 | SYDC_HUMAN | 57 kDa | TRUE | 0.0001 | 1.65 | 1.55 | 1.55 | 17.88 | 0.926909 | 17.89 | 0.929573 | 18.53 | 0.605065 | 1.7 | 18.49 | 0.570983 | 1.6 | 18.53 | 0.556455 | 1.6 | 18.43 | 0.520312 | 1.5 | 18.53 | 0.533781 | 1.6 | 18.49 | 0.57743 | 1.5 |
| 196 | Ubiquitin carboxyl-terminal hydrolase 14 OS=Homo sapiens GN=USP14 PE=1 SV=3 | UBP14_HUMAN | 56 kDa | TRUE | 0.0001 | 1.50 | 1.55 | 1.55 | 17.44 | 0.784032 | 17.37 | 0.84764 | 17.97 | 0.608005 | 1.5 | 17.96 | 0.601149 | 1.5 | 18.13 | 0.609469 | 1.6 | 17.95 | 0.541089 | 1.5 | 18.03 | 0.543446 | 1.5 | 18 | 0.581579 | 1.6 |
| 244 | Dynactin subunit 1 OS=Homo sapiens GN=DCTN1 PE=1 SV=3 | DCTN1_HUMAN | 142 kDa | TRUE | 0.0001 | 1.50 | 1.55 | 1.55 | 17.79 | 0.769163 | 17.87 | 0.760765 | 18.36 | 0.531117 | 1.5 | 18.42 | 0.525187 | 1.5 | 18.43 | 0.519565 | 1.6 | 18.45 | 0.560455 | 1.5 | 18.41 | 0.522983 | 1.6 | 18.46 | 0.523903 | 1.5 |
| 89 | 14-3-3 protein epsilon OS=Homo sapiens GN=YWHAE PE=1 SV=1 | 1433E_HUMAN | 29 kDa | TRUE | 0.0001 | 1.45 | 1.55 | 1.55 | 17.69 | 0.869474 | 17.68 | 0.879982 | 18.23 | 0.702589 | 1.4 | 18.27 | 0.683709 | 1.5 | 18.23 | 0.580992 | 1.5 | 18.28 | 0.567264 | 1.6 | 18.26 | 0.538029 | 1.5 | 18.33 | 0.550824 | 1.6 |
| 551 | Serine/threonine-protein kinase 4 OS=Homo sapiens GN=STK4 PE=1 SV=2 | STK4_HUMAN | 56 kDa |  | 0.0001 | 1.40 | 1.55 | 1.55 | 17.82 | 0.736288 | 17.69 | 0.831318 | 18.24 | 0.522414 | 1.3 | 18.28 | 0.530342 | 1.5 | 18.38 | 0.523183 | 1.5 | 18.34 | 0.53063 | 1.6 | 18.37 | 0.51469 | 1.5 | 18.35 | 0.545112 | 1.6 |
| 189 | Neutral alpha-glucosidase AB OS=Homo sapiens GN=GANAB PE=1 SV=3 | GANAB_HUMAN | 107 kDa |  | 0.0001 | 1.30 | 1.55 | 1.55 | 17.57 | 0.833545 | 17.53 | 0.885276 | 17.93 | 0.545585 | 1.3 | 17.96 | 0.534268 | 1.3 | 18.15 | 0.542851 | 1.5 | 18.16 | 0.55088 | 1.6 | 18.15 | 0.537273 | 1.5 | 18.22 | 0.565226 | 1.6 |
| 444 | Tropomodulin-3 OS=Homo sapiens GN=TMOD3 PE=1 SV=1 | TMOD3_HUMAN | 40 kDa | TRUE | 0.0001 | 1.80 | 1.60 | 1.55 | 17.47 | 0.791079 | 17.4 | 0.700988 | 18.25 | 0.537854 | 1.7 | 18.33 | 0.530683 | 1.9 | 18.07 | 0.52931 | 1.5 | 18.16 | 0.529738 | 1.7 | 18.02 | 0.521625 | 1.5 | 18.05 | 0.547293 | 1.6 |
| 473 | Tubulin--tyrosine ligase-like protein 12 OS=Homo sapiens GN=TTLL12 PE=1 SV=2 | TTL12_HUMAN | 74 kDa |  | 0.0001 | 1.55 | 1.60 | 1.55 | 17.77 | 0.760892 | 17.86 | 0.671378 | 18.43 | 0.56008 | 1.6 | 18.43 | 0.573978 | 1.5 | 18.45 | 0.518846 | 1.6 | 18.53 | 0.558138 | 1.6 | 18.47 | 0.512798 | 1.6 | 18.44 | 0.583045 | 1.5 |
| 600 | Serine/threonine-protein kinase PAK 2 OS=Homo sapiens GN=PAK2 PE=1 SV=3 | PAK2_HUMAN | 58 kDa | TRUE | 0.0001 | 1.55 | 1.60 | 1.55 | 17.53 | 0.643985 | 17.61 | 0.651855 | 18.16 | 0.582816 | 1.6 | 18.13 | 0.553284 | 1.5 | 18.27 | 0.552124 | 1.7 | 18.17 | 0.529619 | 1.5 | 18.17 | 0.512004 | 1.6 | 18.16 | 0.526017 | 1.5 |
| 906 | Insulin-degrading enzyme OS=Homo sapiens GN=IDE PE=1 SV=4 | IDE_HUMAN | 118 kDa |  | 0.001 | 1.25 | 1.60 | 1.55 | 17.35 | 0.597287 | 17.36 | 0.550185 | 17.56 | 0.524493 | 1.2 | 17.74 | 0.529998 | 1.3 | 17.89 | 0.517509 | 1.5 | 18.09 | 0.536409 | 1.7 | 17.97 | 0.501842 | 1.5 | 18.03 | 0.576313 | 1.6 |
| 815 | Collagen alpha-2(I) chain OS=Homo sapiens GN=COL1A2 PE=1 SV=7 | CO1A2_HUMAN | 129 kDa |  | 0.0097 | -1.11 | 1.60 | 1.55 | 18.07 | 0.663417 | 18.16 | 0.549082 | 17.94 | 0.536019 | 0.9 | 18.05 | 0.640077 | 0.9 | 18.83 | 0.629415 | 1.7 | 18.77 | 0.550852 | 1.5 | 18.79 | 0.607334 | 1.6 | 18.79 | 0.572929 | 1.5 |
| 109 | ATP-citrate synthase OS=Homo sapiens GN=ACLY PE=1 SV=3 | ACLY_HUMAN | 121 kDa |  | 0.0001 | 1.65 | 1.65 | 1.55 | 18.1 | 0.867271 | 17.99 | 0.798631 | 18.73 | 0.611487 | 1.6 | 18.74 | 0.610906 | 1.7 | 18.74 | 0.557096 | 1.6 | 18.68 | 0.55836 | 1.7 | 18.66 | 0.541195 | 1.5 | 18.63 | 0.562664 | 1.6 |
| 649 | Adenylate kinase isoenzyme 1 OS=Homo sapiens GN=AK1 PE=1 SV=3 | KAD1_HUMAN | 22 kDa | TRUE | 0.0001 | 1.40 | 1.70 | 1.55 | 17.75 | 0.683377 | 17.68 | 0.918071 | 18.13 | 0.517309 | 1.3 | 18.21 | 0.535694 | 1.5 | 18.5 | 0.525513 | 1.7 | 18.49 | 0.557657 | 1.7 | 18.3 | 0.511131 | 1.5 | 18.35 | 0.535576 | 1.6 |
| 1458 | Bifunctional protein NCOAT OS=Homo sapiens GN=MGEA5 PE=1 SV=2 | NCOAT_HUMAN | ? |  | 0.0097 | 1.45 | 1.70 | 1.55 | 17.92 | 0.502835 | 17.82 | 0.52237 | 18.36 | 0.505532 | 1.4 | 18.36 | 0.505032 | 1.5 | 18.78 | 0.678872 | 1.8 | 18.46 | 0.495064 | 1.6 | 18.53 | 0.511212 | 1.5 | 18.47 | 0.498655 | 1.6 |
| 255 | UTP--glucose-1-phosphate uridylyltransferase OS=Homo sapiens GN=UGP2 PE=1 SV=5 | UGPA_HUMAN | 57 kDa | TRUE | 0.0001 | 1.85 | 1.80 | 1.55 | 17.8 | 0.931413 | 17.86 | 0.874377 | 18.72 | 0.552834 | 1.9 | 18.69 | 0.558674 | 1.8 | 18.72 | 0.526718 | 1.9 | 18.59 | 0.523258 | 1.7 | 18.39 | 0.516401 | 1.6 | 18.32 | 0.578388 | 1.5 |
| 1183 | Follistatin-related protein 1 OS=Homo sapiens GN=FSTL1 PE=1 SV=1 | FSTL1_HUMAN | 35 kDa |  | 0.0019 | 1.85 | 1.80 | 1.55 | 16.35 | 0.833943 | 16.55 | 1.01261 | 17.35 | 0.504479 | 2 | 17.22 | 0.506218 | 1.7 | 17.33 | 0.560837 | 1.9 | 17.24 | 0.498133 | 1.7 | 17.03 | 0.498097 | 1.6 | 16.94 | 0.621507 | 1.5 |
| 187 | Actin-related protein 2 OS=Homo sapiens GN=ACTR2 PE=1 SV=1 | ARP2_HUMAN | 45 kDa | TRUE | 0.0001 | 1.75 | 1.95 | 1.55 | 17.48 | 0.782881 | 17.56 | 0.764491 | 18.3 | 0.566437 | 1.8 | 18.29 | 0.564685 | 1.7 | 18.46 | 0.568704 | 2 | 18.44 | 0.601331 | 1.9 | 18.18 | 0.526199 | 1.6 | 18.14 | 0.545072 | 1.5 |
| 350 | Myosin regulatory light chain 12A OS=Homo sapiens GN=MYL12A PE=1 SV=2 | ML12A_HUMAN | 20 kDa | TRUE | 0.0001 | 1.25 | 1.95 | 1.55 | 17.46 | 0.667448 | 17.63 | 0.671457 | 17.72 | 0.698085 | 1.3 | 17.75 | 0.668102 | 1.2 | 18.47 | 0.602968 | 2.1 | 18.35 | 0.595738 | 1.8 | 18.14 | 0.533441 | 1.6 | 18.2 | 0.566394 | 1.5 |
| 1656 | Protein XRP2 OS=Homo sapiens GN=RP2 PE=1 SV=4 | XRP2_HUMAN | 40 kDa |  | 0.024 | 1.75 | 2.10 | 1.55 | 16.41 | 1.13635 | 16.52 | 0.8785 | 17.33 | 0.497329 | 1.8 | 17.29 | 0.498088 | 1.7 | 17.48 | 0.514068 | 2.1 | 17.57 | 0.503274 | 2.1 | 17.18 | 0.502873 | 1.6 | 16.98 | 0.555542 | 1.5 |
| 1649 | 60S ribosomal protein L36 OS=Homo sapiens GN=RPL36 PE=1 SV=3 | RL36_HUMAN | 12 kDa |  | 0.011 | -1.11 | 1.05 | 1.60 | 17.94 | 0.546901 | 17.75 | 0.497917 | 17.68 | 0.489947 | 0.8 | 17.73 | 0.496796 | 1 | 17.94 | 0.519938 | 1 | 17.91 | 0.493448 | 1.1 | 18.54 | 0.552537 | 1.5 | 18.54 | 0.521709 | 1.7 |
| 683 | 40S ribosomal protein S4, X isoform OS=Homo sapiens GN=RPS4X PE=1 SV=2 | RS4X_HUMAN | 30 kDa |  | 0.0071 | 1.20 | 1.15 | 1.60 | 18.36 | 1.012968 | 18.25 | 1.034923 | 18.59 | 0.546945 | 1.2 | 18.58 | 0.53221 | 1.2 | 18.44 | 0.580168 | 1.1 | 18.5 | 0.608331 | 1.2 | 18.94 | 0.686726 | 1.6 | 18.88 | 0.635904 | 1.6 |
| 1067 | ELAV-like protein 1 OS=Homo sapiens GN=ELAVL1 PE=1 SV=2 | ELAV1_HUMAN | 36 kDa |  | 0.001 | 1.10 | 1.25 | 1.60 | 16.83 | 0.605498 | 16.87 | 0.636599 | 16.95 | 0.544475 | 1.1 | 16.95 | 0.533034 | 1.1 | 17.14 | 0.545813 | 1.3 | 17.15 | 0.536345 | 1.2 | 17.45 | 0.553755 | 1.6 | 17.51 | 0.551765 | 1.6 |
| 239 | 40S ribosomal protein S3 OS=Homo sapiens GN=RPS3 PE=1 SV=2 | RS3_HUMAN | 27 kDa |  | 0.0001 | 1.55 | 1.30 | 1.60 | 17.97 | 0.777859 | 17.87 | 0.784977 | 18.51 | 0.547828 | 1.5 | 18.52 | 0.544786 | 1.6 | 18.33 | 0.542428 | 1.3 | 18.26 | 0.539341 | 1.3 | 18.64 | 0.590971 | 1.6 | 18.49 | 0.567553 | 1.6 |
| 201 | Coatomer subunit alpha OS=Homo sapiens GN=COPA PE=1 SV=2 | COPA_HUMAN | 138 kDa |  | 0.0001 | 1.50 | 1.30 | 1.60 | 17.89 | 0.828378 | 17.92 | 0.73435 | 18.51 | 0.610189 | 1.5 | 18.49 | 0.63266 | 1.5 | 18.27 | 0.550988 | 1.3 | 18.31 | 0.54845 | 1.3 | 18.51 | 0.55506 | 1.6 | 18.53 | 0.632417 | 1.6 |
| 449 | Integrin-linked protein kinase OS=Homo sapiens GN=ILK PE=1 SV=2 | ILK_HUMAN | 51 kDa |  | 0.0001 | 1.45 | 1.30 | 1.60 | 18.19 | 0.781514 | 18.16 | 0.725347 | 18.63 | 0.554689 | 1.4 | 18.67 | 0.589866 | 1.5 | 18.61 | 0.587864 | 1.3 | 18.58 | 0.523186 | 1.3 | 18.78 | 0.535425 | 1.5 | 18.86 | 0.563037 | 1.7 |
| 204 | Ribosome-binding protein 1 OS=Homo sapiens GN=RRBP1 PE=1 SV=4 | RRBP1_HUMAN | 152 kDa | TRUE | 0.0001 | 1.20 | 1.30 | 1.60 | 18.22 | 0.819132 | 18.24 | 0.750517 | 18.47 | 0.556468 | 1.2 | 18.48 | 0.55072 | 1.2 | 18.65 | 0.530052 | 1.3 | 18.64 | 0.535561 | 1.3 | 18.91 | 0.550291 | 1.6 | 18.95 | 0.553128 | 1.6 |
| 429 | Peroxiredoxin-1 OS=Homo sapiens GN=PRDX1 PE=1 SV=1 | PRDX1_HUMAN | 22 kDa | TRUE | 0.0001 | 1.10 | 1.35 | 1.60 | 18.47 | 0.85487 | 18.45 | 0.716395 | 18.54 | 0.572239 | 1.1 | 18.58 | 0.551026 | 1.1 | 18.92 | 0.568759 | 1.4 | 18.77 | 0.536665 | 1.3 | 19.16 | 0.558218 | 1.6 | 19.14 | 0.557516 | 1.6 |
| 886 | UDP-glucose 6-dehydrogenase OS=Homo sapiens GN=UGDH PE=1 SV=1 | UGDH_HUMAN | 55 kDa |  | 0.0001 | 1.00 | 1.35 | 1.60 | 18.26 | 0.752301 | 18.21 | 0.598602 | 18.29 | 0.525598 | 1 | 18.26 | 0.521989 | 1 | 18.65 | 0.540672 | 1.3 | 18.66 | 0.559711 | 1.4 | 18.93 | 0.563693 | 1.6 | 18.88 | 0.55818 | 1.6 |
| 544 | Galectin-3 OS=Homo sapiens GN=LGALS3 PE=1 SV=5 | LEG3_HUMAN | 26 kDa |  | 0.0082 | 1.45 | 1.40 | 1.60 | 17.68 | 0.741464 | 17.76 | 0.842203 | 18.19 | 0.540812 | 1.5 | 18.2 | 0.553578 | 1.4 | 18.17 | 0.531448 | 1.4 | 18.12 | 0.656983 | 1.4 | 18.32 | 0.544283 | 1.6 | 18.36 | 0.553117 | 1.6 |
| 1434 | Ribosome maturation protein SBDS OS=Homo sapiens GN=SBDS PE=1 SV=4 | SBDS_HUMAN | 29 kDa | TRUE | 0.018 | 1.40 | 1.40 | 1.60 | 16.6 | 0.545086 | 16.37 | 0.830294 | 16.99 | 0.536487 | 1.3 | 16.96 | 0.509095 | 1.5 | 17.01 | 0.49349 | 1.3 | 16.94 | 0.517724 | 1.5 | 17.22 | 0.496845 | 1.5 | 17.11 | 0.508656 | 1.7 |
| 1018 | Choline-phosphate cytidylyltransferase A OS=Homo sapiens GN=PCYT1A PE=1 SV=2 | PCY1A_HUMAN | 42 kDa |  | 0.0003 | 1.30 | 1.40 | 1.60 | 18.22 | 0.85385 | 18.24 | 0.836659 | 18.5 | 0.529829 | 1.3 | 18.53 | 0.507691 | 1.3 | 18.67 | 0.519862 | 1.4 | 18.65 | 0.524586 | 1.4 | 18.93 | 0.533485 | 1.6 | 18.87 | 0.526097 | 1.6 |
| 698 | Eukaryotic translation initiation factor 4B OS=Homo sapiens GN=EIF4B PE=1 SV=2 | IF4B_HUMAN | 69 kDa | TRUE | 0.0001 | 1.20 | 1.40 | 1.60 | 17.84 | 0.582962 | 17.87 | 0.60174 | 18.08 | 0.543074 | 1.2 | 18.11 | 0.576654 | 1.2 | 18.29 | 0.528469 | 1.4 | 18.4 | 0.52519 | 1.4 | 18.49 | 0.524483 | 1.6 | 18.58 | 0.531681 | 1.6 |
| 392 | Fascin OS=Homo sapiens GN=FSCN1 PE=1 SV=3 | FSCN1_HUMAN | 55 kDa | TRUE | 0.0001 | 1.05 | 1.40 | 1.60 | 17.87 | 0.629588 | 17.87 | 0.662769 | 17.91 | 0.553281 | 1 | 17.89 | 0.574031 | 1.1 | 18.34 | 0.568799 | 1.4 | 18.33 | 0.535916 | 1.4 | 18.55 | 0.594218 | 1.6 | 18.53 | 0.579806 | 1.6 |
| 319 | AP-1 complex subunit beta-1 OS=Homo sapiens GN=AP1B1 PE=1 SV=2 | AP1B1_HUMAN | 105 kDa | TRUE | 0.0001 | 1.80 | 1.45 | 1.60 | 17.36 | 0.654024 | 17.36 | 0.677362 | 18.16 | 0.707358 | 1.8 | 18.15 | 0.735383 | 1.8 | 17.9 | 0.530352 | 1.4 | 17.97 | 0.534285 | 1.5 | 18 | 0.542264 | 1.6 | 17.96 | 0.531262 | 1.6 |
| 750 | Basic leucine zipper and W2 domain-containing protein 1 OS=Homo sapiens GN=BZW1 PE=1 SV=1 | BZW1_HUMAN | 48 kDa |  | 0.0035 | 1.35 | 1.45 | 1.60 | 18.84 | 0.777384 | 18.79 | 0.697838 | 19.18 | 0.655308 | 1.3 | 19.22 | 0.61901 | 1.4 | 19.3 | 0.582444 | 1.4 | 19.39 | 0.565494 | 1.5 | 19.48 | 0.601416 | 1.6 | 19.44 | 0.583642 | 1.6 |
| 741 | Splicing factor 3A subunit 1 OS=Homo sapiens GN=SF3A1 PE=1 SV=1 | SF3A1_HUMAN | 89 kDa | TRUE | 0.0001 | 1.25 | 1.45 | 1.60 | 18.05 | 0.771979 | 18.02 | 0.751657 | 18.35 | 0.557386 | 1.2 | 18.33 | 0.552638 | 1.3 | 18.57 | 0.527532 | 1.5 | 18.47 | 0.508541 | 1.4 | 18.68 | 0.516735 | 1.6 | 18.7 | 0.543026 | 1.6 |
| 759 | Eukaryotic translation initiation factor 4 gamma 1 OS=Homo sapiens GN=EIF4G1 PE=1 SV=4 | IF4G1_HUMAN | 175 kDa | TRUE | 0.00034 | 1.25 | 1.45 | 1.60 | 17.65 | 0.770488 | 17.58 | 0.832308 | 17.87 | 0.559751 | 1.2 | 17.93 | 0.553642 | 1.3 | 18.15 | 0.525776 | 1.4 | 18.18 | 0.568799 | 1.5 | 18.32 | 0.585442 | 1.6 | 18.2 | 0.556489 | 1.6 |
| 396 | Proliferation-associated protein 2G4 OS=Homo sapiens GN=PA2G4 PE=1 SV=3 | PA2G4_HUMAN | 44 kDa |  | 0.0001 | 1.15 | 1.45 | 1.60 | 17.98 | 0.703006 | 18.07 | 0.695125 | 18.15 | 0.537491 | 1.2 | 18.18 | 0.524211 | 1.1 | 18.55 | 0.545905 | 1.5 | 18.49 | 0.524016 | 1.4 | 18.68 | 0.539903 | 1.6 | 18.75 | 0.572128 | 1.6 |
| 1856 | Cleavage and polyadenylation specificity factor subunit 5 OS=Homo sapiens GN=NUDT21 PE=1 SV=1 | CPSF5_HUMAN | 26 kDa |  | 0.011 | 1.95 | 1.50 | 1.60 | 19.04 | 0.782525 | 18.96 | 0.621878 | 19.97 | 0.520295 | 1.9 | 19.92 | 0.519598 | 2 | 19.66 | 0.522515 | 1.5 | 19.58 | 0.522223 | 1.5 | 19.68 | 0.517951 | 1.6 | 19.66 | 0.523682 | 1.6 |
| 773 | Tryptophan--tRNA ligase, cytoplasmic OS=Homo sapiens GN=WARS PE=1 SV=2 | SYWC_HUMAN | 53 kDa |  | 0.0028 | 1.45 | 1.50 | 1.60 | 17.58 | 0.68213 | 17.77 | 0.642989 | 18.13 | 0.500685 | 1.5 | 18.22 | 0.505124 | 1.4 | 18.14 | 0.563454 | 1.5 | 18.3 | 0.495721 | 1.5 | 18.26 | 0.4958 | 1.6 | 18.34 | 0.825233 | 1.6 |
| 1264 | Methylosome protein 50 OS=Homo sapiens GN=WDR77 PE=1 SV=1 | MEP50_HUMAN | 37 kDa |  | 0.012 | 1.40 | 1.50 | 1.60 | 18.03 | 0.90595 | 18.04 | 0.926215 | 18.38 | 0.574078 | 1.4 | 18.39 | 0.567141 | 1.4 | 18.62 | 0.4956 | 1.5 | 18.62 | 0.535667 | 1.5 | 18.64 | 0.503445 | 1.6 | 18.75 | 0.508361 | 1.6 |
| 303 | Eukaryotic translation initiation factor 5A-1 OS=Homo sapiens GN=EIF5A PE=1 SV=2 | IF5A1_HUMAN | 17 kDa |  | 0.00055 | 1.35 | 1.50 | 1.60 | 17.34 | 0.742055 | 17.38 | 0.698378 | 17.79 | 0.73877 | 1.4 | 17.83 | 0.742362 | 1.3 | 17.88 | 0.652524 | 1.5 | 17.93 | 0.612068 | 1.5 | 18.09 | 0.661914 | 1.7 | 17.95 | 0.717979 | 1.5 |
| 260 | Heterogeneous nuclear ribonucleoprotein Q OS=Homo sapiens GN=SYNCRIP PE=1 SV=2 | HNRPQ_HUMAN | 70 kDa | TRUE | 0.0001 | 1.25 | 1.50 | 1.60 | 17.81 | 0.668412 | 17.85 | 0.688946 | 18.13 | 0.543743 | 1.3 | 18.09 | 0.557407 | 1.2 | 18.4 | 0.538684 | 1.5 | 18.37 | 0.531903 | 1.5 | 18.48 | 0.534558 | 1.6 | 18.47 | 0.590139 | 1.6 |
| 769 | Sorcin OS=Homo sapiens GN=SRI PE=1 SV=1 | SORCN_HUMAN | 22 kDa |  | 0.0028 | 1.25 | 1.50 | 1.60 | 17.91 | 0.815033 | 18.02 | 0.846772 | 18.29 | 0.638195 | 1.3 | 18.29 | 0.634659 | 1.2 | 18.62 | 0.561842 | 1.6 | 18.51 | 0.531353 | 1.4 | 18.67 | 0.526482 | 1.7 | 18.64 | 0.559776 | 1.5 |
| 1037 | Sulfotransferase family cytosolic 1B member 1 OS=Homo sapiens GN=SULT1B1 PE=1 SV=2 | ST1B1_HUMAN | 35 kDa |  | 0.00036 | 1.50 | 1.55 | 1.60 | 17.72 | 0.631929 | 17.88 | 0.618691 | 18.33 | 0.568336 | 1.6 | 18.39 | 0.573439 | 1.4 | 18.46 | 0.528849 | 1.7 | 18.33 | 0.508779 | 1.4 | 18.37 | 0.512507 | 1.6 | 18.54 | 0.527192 | 1.6 |
| 657 | Serine--tRNA ligase, cytoplasmic OS=Homo sapiens GN=SARS PE=1 SV=3 | SYSC_HUMAN | 59 kDa |  | 0.0088 | 1.45 | 1.55 | 1.60 | 17.36 | 1.238739 | 17.5 | 1.057695 | 17.86 | 0.578301 | 1.5 | 17.97 | 0.559333 | 1.4 | 18.03 | 0.545884 | 1.7 | 17.97 | 0.580735 | 1.4 | 18.04 | 0.574989 | 1.6 | 18.05 | 0.579143 | 1.6 |
| 674 | Phosphoribosyl pyrophosphate synthase-associated protein 2 OS=Homo sapiens GN=PRPSAP2 PE=1 SV=1 | KPRB_HUMAN | 41 kDa | TRUE | 0.00026 | 1.55 | 1.55 | 1.60 | 17.92 | 0.662192 | 17.84 | 0.597776 | 18.54 | 0.531681 | 1.6 | 18.41 | 0.506761 | 1.5 | 18.5 | 0.520733 | 1.5 | 18.52 | 0.529996 | 1.6 | 18.47 | 0.506277 | 1.5 | 18.62 | 0.516289 | 1.7 |
| 263 | Adenylosuccinate lyase OS=Homo sapiens GN=ADSL PE=1 SV=2 | PUR8_HUMAN | 55 kDa |  | 0.0001 | 1.45 | 1.55 | 1.60 | 17.74 | 0.86135 | 17.78 | 0.871937 | 18.31 | 0.519684 | 1.5 | 18.29 | 0.526194 | 1.4 | 18.38 | 0.544291 | 1.6 | 18.29 | 0.530673 | 1.5 | 18.36 | 0.517863 | 1.6 | 18.46 | 0.588924 | 1.6 |
| 137 | Catalase OS=Homo sapiens GN=CAT PE=1 SV=3 | CATA_HUMAN | 60 kDa |  | 0.0001 | 1.35 | 1.55 | 1.60 | 17.97 | 0.835984 | 18.09 | 0.788582 | 18.41 | 0.59569 | 1.4 | 18.44 | 0.593173 | 1.3 | 18.61 | 0.550398 | 1.6 | 18.57 | 0.598181 | 1.5 | 18.75 | 0.567553 | 1.7 | 18.66 | 0.596316 | 1.5 |
| 594 | Hepatoma-derived growth factor OS=Homo sapiens GN=HDGF PE=1 SV=1 | HDGF_HUMAN | 27 kDa | TRUE | 0.0001 | 1.30 | 1.55 | 1.60 | 18.1 | 0.752735 | 18.02 | 0.59481 | 18.43 | 0.540553 | 1.3 | 18.41 | 0.550869 | 1.3 | 18.76 | 0.508152 | 1.6 | 18.57 | 0.517362 | 1.5 | 18.74 | 0.523697 | 1.6 | 18.68 | 0.551959 | 1.6 |
| 1609 | Splicing factor 3A subunit 2 OS=Homo sapiens GN=SF3A2 PE=1 SV=2 | SF3A2_HUMAN | 49 kDa |  | 0.00094 | 1.30 | 1.55 | 1.60 | 17.6 | 0.581795 | 17.73 | 0.560679 | 18.03 | 0.487994 | 1.4 | 17.97 | 0.494039 | 1.2 | 18.18 | 0.494856 | 1.5 | 18.39 | 0.575462 | 1.6 | 18.25 | 0.520119 | 1.6 | 18.43 | 0.491669 | 1.6 |
| 360 | FMNL_HUMAN | FMNL_HUMAN | ? | TRUE | 0.0001 | 1.75 | 1.60 | 1.60 | 17.04 | 0.872709 | 17 | 0.811914 | 17.73 | 0.556396 | 1.7 | 17.82 | 0.53891 | 1.8 | 17.71 | 0.552181 | 1.6 | 17.68 | 0.516552 | 1.6 | 17.7 | 0.536039 | 1.6 | 17.63 | 0.523123 | 1.6 |
| 364 | Ubiquitin carboxyl-terminal hydrolase 15 OS=Homo sapiens GN=USP15 PE=1 SV=3 | UBP15_HUMAN | 112 kDa | TRUE | 0.0001 | 1.70 | 1.60 | 1.60 | 17.61 | 0.950001 | 17.59 | 0.848207 | 18.31 | 0.553521 | 1.6 | 18.4 | 0.59168 | 1.8 | 18.16 | 0.528495 | 1.6 | 18.14 | 0.53511 | 1.6 | 18.19 | 0.524331 | 1.6 | 18.18 | 0.55081 | 1.6 |
| 96 | Acylamino-acid-releasing enzyme OS=Homo sapiens GN=APEH PE=1 SV=4 | ACPH_HUMAN | 81 kDa |  | 0.0001 | 1.70 | 1.60 | 1.60 | 17.6 | 1.201686 | 17.57 | 1.070523 | 18.3 | 0.585699 | 1.7 | 18.27 | 0.596015 | 1.7 | 18.27 | 0.602357 | 1.6 | 18.23 | 0.550214 | 1.6 | 18.3 | 0.577931 | 1.6 | 18.22 | 0.60235 | 1.6 |
| 1480 | Catechol O-methyltransferase OS=Homo sapiens GN=COMT PE=1 SV=2 | COMT_HUMAN | 30 kDa |  | 0.0097 | 1.70 | 1.60 | 1.60 | 18.57 | 0.902508 | 18.67 | 0.808401 | 19.24 | 0.559622 | 1.7 | 19.36 | 0.55822 | 1.7 | 19.17 | 0.511885 | 1.6 | 19.32 | 0.583281 | 1.6 | 19.26 | 0.507655 | 1.7 | 19.21 | 0.509545 | 1.5 |
| 803 | Translin-associated protein X OS=Homo sapiens GN=TSNAX PE=1 SV=1 | TSNAX_HUMAN | 33 kDa |  | 0.00012 | 1.50 | 1.60 | 1.60 | 17.58 | 0.677713 | 17.63 | 0.670893 | 18.15 | 0.544752 | 1.5 | 18.22 | 0.544255 | 1.5 | 18.3 | 0.534456 | 1.7 | 18.2 | 0.505777 | 1.5 | 18.29 | 0.526396 | 1.7 | 18.15 | 0.604733 | 1.5 |
| 575 | Deoxyribose-phosphate aldolase OS=Homo sapiens GN=DERA PE=1 SV=2 | DEOC_HUMAN | 35 kDa | TRUE | 0.0095 | 1.50 | 1.60 | 1.60 | 17.31 | 0.966265 | 17.41 | 1.07753 | 17.95 | 0.595482 | 1.6 | 17.88 | 0.560642 | 1.4 | 18 | 0.510331 | 1.6 | 18.02 | 0.548946 | 1.6 | 17.95 | 0.512853 | 1.6 | 18.02 | 0.586607 | 1.6 |
| 618 | 60 kDa heat shock protein, mitochondrial OS=Homo sapiens GN=HSPD1 PE=1 SV=2 | CH60_HUMAN | 61 kDa |  | 0.00034 | 1.75 | 1.65 | 1.60 | 16.3 | 1.001785 | 16.43 | 0.897972 | 17.14 | 0.549801 | 1.8 | 17.18 | 0.577286 | 1.7 | 17.03 | 0.544843 | 1.8 | 17.03 | 0.55209 | 1.5 | 17.03 | 0.517371 | 1.6 | 17.09 | 0.599405 | 1.6 |
| 32 | Talin-1 OS=Homo sapiens GN=TLN1 PE=1 SV=3 | TLN1_HUMAN | 270 kDa | TRUE | 0.0001 | 1.70 | 1.65 | 1.60 | 17.69 | 0.979905 | 17.72 | 0.949143 | 18.47 | 0.587492 | 1.7 | 18.48 | 0.586993 | 1.7 | 18.37 | 0.542074 | 1.7 | 18.34 | 0.55736 | 1.6 | 18.35 | 0.538029 | 1.6 | 18.39 | 0.566572 | 1.6 |
| 976 | Spermine synthase OS=Homo sapiens GN=SMS PE=1 SV=2 | SPSY_HUMAN | 41 kDa |  | 0.0001 | 1.70 | 1.65 | 1.60 | 16.17 | 0.618919 | 16.15 | 0.611822 | 16.96 | 0.55245 | 1.7 | 16.91 | 0.568479 | 1.7 | 16.94 | 0.558481 | 1.7 | 16.84 | 0.520423 | 1.6 | 16.93 | 0.582122 | 1.7 | 16.75 | 0.607863 | 1.5 |
| 315 | Rho GTPase-activating protein 1 OS=Homo sapiens GN=ARHGAP1 PE=1 SV=1 | RHG01_HUMAN | 50 kDa |  | 0.0001 | 1.40 | 1.65 | 1.60 | 18.04 | 0.675771 | 18.11 | 0.685645 | 18.54 | 0.575834 | 1.4 | 18.56 | 0.559505 | 1.4 | 18.76 | 0.566548 | 1.7 | 18.73 | 0.56216 | 1.6 | 18.69 | 0.527094 | 1.6 | 18.8 | 0.572321 | 1.6 |
| 622 | Eukaryotic translation initiation factor 3 subunit J OS=Homo sapiens GN=EIF3J PE=1 SV=2 | EIF3J_HUMAN | 29 kDa |  | 0.0019 | 1.20 | 1.65 | 1.60 | 17.86 | 0.896361 | 17.86 | 0.845893 | 18.09 | 0.567653 | 1.2 | 18.15 | 0.561709 | 1.2 | 18.6 | 0.579243 | 1.6 | 18.56 | 0.565631 | 1.7 | 18.47 | 0.54219 | 1.5 | 18.61 | 0.612793 | 1.7 |
| 467 | Keratin, type II cytoskeletal 5 OS=Homo sapiens GN=KRT5 PE=1 SV=3 | K2C5_HUMAN | 62 kDa | TRUE | 0.0001 | -1.25 | 1.65 | 1.60 | 17.51 | 0.764213 | 17.62 | 0.772796 | 17.3 | 0.562768 | 0.8 | 17.38 | 0.591547 | 0.8 | 18.32 | 0.627645 | 1.8 | 18.23 | 0.557322 | 1.5 | 18.19 | 0.611302 | 1.7 | 18.2 | 0.58286 | 1.5 |
| 40 | Ras GTPase-activating-like protein IQGAP1 OS=Homo sapiens GN=IQGAP1 PE=1 SV=1 | IQGA1_HUMAN | 189 kDa | TRUE | 0.0001 | 1.75 | 1.70 | 1.60 | 17.66 | 0.985505 | 17.66 | 0.928179 | 18.41 | 0.637685 | 1.7 | 18.43 | 0.634591 | 1.8 | 18.33 | 0.567216 | 1.7 | 18.3 | 0.566617 | 1.7 | 18.29 | 0.538504 | 1.6 | 18.31 | 0.556451 | 1.6 |
| 411 | Putative neutrophil cytosol factor 1B OS=Homo sapiens GN=NCF1B PE=5 SV=2 | NCF1B_HUMAN | 45 kDa | TRUE | 0.0001 | 1.65 | 1.70 | 1.60 | 17.97 | 1.00611 | 18.08 | 0.948056 | 18.77 | 0.578747 | 1.7 | 18.75 | 0.584407 | 1.6 | 18.81 | 0.551483 | 1.9 | 18.6 | 0.544779 | 1.5 | 18.61 | 0.525038 | 1.6 | 18.69 | 0.60077 | 1.6 |
| 351 | Profilin-1 OS=Homo sapiens GN=PFN1 PE=1 SV=2 | PROF1_HUMAN | 15 kDa |  | 0.0045 | 1.40 | 1.70 | 1.60 | 18.43 | 1.101767 | 18.57 | 1.115735 | 19 | 0.674528 | 1.5 | 19.02 | 0.709083 | 1.3 | 19.3 | 0.649864 | 1.8 | 19.19 | 0.667434 | 1.6 | 19.19 | 0.624465 | 1.7 | 19.17 | 0.648161 | 1.5 |
| 27 | Alpha-actinin-1 OS=Homo sapiens GN=ACTN1 PE=1 SV=2 | ACTN1_HUMAN | 103 kDa | TRUE | 0.0001 | 1.65 | 1.75 | 1.60 | 17.74 | 1.044102 | 17.69 | 0.941642 | 18.38 | 0.652842 | 1.6 | 18.41 | 0.660961 | 1.7 | 18.52 | 0.593945 | 1.8 | 18.43 | 0.581624 | 1.7 | 18.33 | 0.570613 | 1.6 | 18.31 | 0.571099 | 1.6 |
| 118 | Actin-related protein 3 OS=Homo sapiens GN=ACTR3 PE=1 SV=3 | ARP3_HUMAN | 47 kDa | TRUE | 0.0001 | 1.60 | 1.75 | 1.60 | 18.07 | 0.97119 | 18.09 | 0.933608 | 18.71 | 0.598794 | 1.6 | 18.73 | 0.594212 | 1.6 | 18.85 | 0.569292 | 1.8 | 18.8 | 0.53648 | 1.7 | 18.69 | 0.547886 | 1.6 | 18.74 | 0.585215 | 1.6 |
| 513 | Actin-related protein 2/3 complex subunit 4 OS=Homo sapiens GN=ARPC4 PE=1 SV=3 | ARPC4_HUMAN | 20 kDa |  | 0.0001 | 2.10 | 1.80 | 1.60 | 18.06 | 0.916929 | 18.15 | 0.802399 | 19.15 | 0.579625 | 2.2 | 19.1 | 0.605905 | 2 | 18.9 | 0.54855 | 1.8 | 18.89 | 0.566321 | 1.8 | 18.7 | 0.528665 | 1.6 | 18.73 | 0.574099 | 1.6 |
| 428 | N-acetyl-D-glucosamine kinase OS=Homo sapiens GN=NAGK PE=1 SV=4 | NAGK_HUMAN | 37 kDa |  | 0.0001 | 1.65 | 1.80 | 1.60 | 17.71 | 0.877157 | 17.66 | 0.8701 | 18.31 | 0.613211 | 1.6 | 18.31 | 0.590379 | 1.7 | 18.48 | 0.53551 | 1.8 | 18.46 | 0.546565 | 1.8 | 18.33 | 0.510226 | 1.6 | 18.31 | 0.539049 | 1.6 |
| 816 | Collagen alpha-2(V) chain OS=Homo sapiens GN=COL5A2 PE=1 SV=3 | CO5A2_HUMAN | 145 kDa | TRUE | 0.0067 | 1.85 | 1.85 | 1.60 | 18.08 | 0.531118 | 18.26 | 0.521272 | 19.04 | 0.572838 | 1.9 | 19.07 | 0.549123 | 1.8 | 19.18 | 0.516059 | 2.1 | 18.97 | 0.5212 | 1.6 | 18.81 | 0.522274 | 1.7 | 18.79 | 0.5271 | 1.5 |
| 408 | Grancalcin OS=Homo sapiens GN=GCA PE=1 SV=2 | GRAN_HUMAN | 24 kDa |  | 0.0001 | 2.75 | 1.90 | 1.60 | 17.15 | 1.111962 | 17.22 | 0.960605 | 18.5 | 0.746982 | 2.9 | 18.48 | 0.73428 | 2.6 | 18.05 | 0.5793 | 1.9 | 18.11 | 0.556468 | 1.9 | 17.8 | 0.537631 | 1.6 | 17.78 | 0.566229 | 1.6 |
| 895 | Chloride intracellular channel protein 4 OS=Homo sapiens GN=CLIC4 PE=1 SV=4 | CLIC4_HUMAN | 29 kDa | TRUE | 0.0022 | 1.60 | 1.90 | 1.60 | 16.84 | 0.645026 | 16.98 | 0.78714 | 17.42 | 0.557787 | 1.5 | 17.73 | 0.538028 | 1.7 | 17.48 | 0.566726 | 1.5 | 17.88 | 1.058228 | 2.3 | 17.69 | 0.510929 | 1.8 | 17.45 | 0.519404 | 1.4 |
| 1537 | Band 4.1-like protein 3 OS=Homo sapiens GN=EPB41L3 PE=1 SV=2 | E41L3_HUMAN | 121 kDa | TRUE | 0.0097 | -1.05 | 1.15 | 1.65 | 17.6 | 0.650093 | 17.56 | 0.639708 | 17.45 | 0.615529 | 0.9 | 17.5 | 0.582779 | 1 | 17.8 | 0.495146 | 1.2 | 17.7 | 0.500157 | 1.1 | 18.24 | 0.513703 | 1.6 | 18.27 | 0.501606 | 1.7 |
| 250 | Histone H1.5 OS=Homo sapiens GN=HIST1H1B PE=1 SV=3 | H15_HUMAN | 23 kDa | TRUE | 0.00033 | 1.05 | 1.30 | 1.65 | 18.43 | 0.681382 | 18.53 | 0.724589 | 18.55 | 0.551485 | 1.1 | 18.48 | 0.578198 | 1 | 18.92 | 0.610518 | 1.4 | 18.79 | 0.556096 | 1.2 | 19.19 | 0.655209 | 1.7 | 19.23 | 0.642236 | 1.6 |
| 945 | RNA-binding protein FUS OS=Homo sapiens GN=FUS PE=1 SV=1 | FUS_HUMAN | 53 kDa | TRUE | 0.00094 | 1.05 | 1.30 | 1.65 | 18.58 | 0.547309 | 18.57 | 0.526194 | 18.65 | 0.51369 | 1.1 | 18.63 | 0.508141 | 1 | 19.09 | 0.510181 | 1.4 | 18.85 | 0.499047 | 1.2 | 19.34 | 0.514339 | 1.7 | 19.26 | 0.51866 | 1.6 |
| 707 | Reticulocalbin-3 OS=Homo sapiens GN=RCN3 PE=1 SV=1 | RCN3_HUMAN | 37 kDa |  | 0.0001 | 1.15 | 1.30 | 1.65 | 17.32 | 0.883138 | 17.09 | 0.848012 | 17.38 | 0.526164 | 1 | 17.42 | 0.547607 | 1.3 | 17.52 | 0.538897 | 1.1 | 17.6 | 0.565571 | 1.5 | 17.86 | 0.538527 | 1.4 | 17.91 | 0.556215 | 1.9 |
| 1346 | Protein SEC13 homolog OS=Homo sapiens GN=SEC13 PE=1 SV=3 | SEC13_HUMAN | 36 kDa |  | 0.0032 | 1.70 | 1.35 | 1.65 | 17.59 | 0.583479 | 17.39 | 0.573071 | 18.17 | 0.640306 | 1.5 | 18.3 | 0.598704 | 1.9 | 17.87 | 0.533091 | 1.2 | 17.99 | 0.5056 | 1.5 | 18.24 | 0.511869 | 1.6 | 18.18 | 0.50269 | 1.7 |
| 1045 | Clathrin light chain A OS=Homo sapiens GN=CLTA PE=1 SV=1 | CLCA_HUMAN | 27 kDa |  | 0.0064 | 1.20 | 1.35 | 1.65 | 18.46 | 1.133341 | 18.23 | 0.696423 | 18.53 | 0.523753 | 1 | 18.65 | 0.513047 | 1.4 | 18.84 | 0.531104 | 1.2 | 18.79 | 0.533403 | 1.5 | 18.95 | 0.602581 | 1.5 | 19.11 | 0.562699 | 1.8 |
| 798 | Protein S100-A11 OS=Homo sapiens GN=S100A11 PE=1 SV=2 | S10AB_HUMAN | 12 kDa |  | 0.021 | 1.65 | 1.40 | 1.65 | 18.53 | 1.847423 | 18.61 | 1.861588 | 19.33 | 0.576668 | 1.7 | 19.33 | 0.560359 | 1.6 | 19 | 0.554804 | 1.5 | 18.92 | 0.569474 | 1.3 | 19.16 | 0.559448 | 1.6 | 19.18 | 0.603443 | 1.7 |
| 286 | Eukaryotic translation initiation factor 3 subunit A OS=Homo sapiens GN=EIF3A PE=1 SV=1 | EIF3A_HUMAN | 167 kDa | TRUE | 0.0001 | 1.50 | 1.40 | 1.65 | 17.7 | 0.743269 | 17.71 | 0.717863 | 18.25 | 0.52582 | 1.5 | 18.21 | 0.526311 | 1.5 | 18.23 | 0.524022 | 1.4 | 18.2 | 0.525508 | 1.4 | 18.37 | 0.518474 | 1.6 | 18.45 | 0.576236 | 1.7 |
| 939 | ATP-dependent RNA helicase DDX3X OS=Homo sapiens GN=DDX3X PE=1 SV=3 | DDX3X_HUMAN | 73 kDa | TRUE | 0.02 | 1.35 | 1.40 | 1.65 | 18.36 | 0.600398 | 18.3 | 0.86912 | 18.66 | 0.561009 | 1.3 | 18.65 | 0.536992 | 1.4 | 18.87 | 0.534844 | 1.4 | 18.78 | 0.511575 | 1.4 | 18.94 | 0.542042 | 1.5 | 19.05 | 0.543282 | 1.8 |
| 1331 | Heterogeneous nuclear ribonucleoproteins C1/C2 OS=Homo sapiens GN=HNRNPC PE=1 SV=4 | HNRPC_HUMAN | 34 kDa |  | 0.0074 | 1.25 | 1.40 | 1.65 | 18.12 | 0.835726 | 18.09 | 0.951343 | 18.23 | 0.543816 | 1.1 | 18.41 | 0.538927 | 1.4 | 18.47 | 0.535655 | 1.3 | 18.62 | 0.515314 | 1.5 | 18.81 | 0.538395 | 1.6 | 18.86 | 0.517263 | 1.7 |
| 646 | Heterogeneous nuclear ribonucleoprotein H OS=Homo sapiens GN=HNRNPH1 PE=1 SV=4 | HNRH1_HUMAN | 49 kDa | TRUE | 0.00027 | -1.05 | 1.40 | 1.65 | 17.91 | 0.86873 | 18.02 | 0.853555 | 17.9 | 0.577355 | 1 | 17.92 | 0.577959 | 0.9 | 18.37 | 0.537451 | 1.4 | 18.42 | 0.53821 | 1.4 | 18.59 | 0.513977 | 1.7 | 18.63 | 0.515729 | 1.6 |
| 1453 | Histone H1.2 OS=Homo sapiens GN=HIST1H1C PE=1 SV=2 | H12_HUMAN | 21 kDa | TRUE | 0.0052 | -1.18 | 1.40 | 1.65 | 18.94 | 0.709211 | 19.1 | 0.631321 | 18.79 | 0.510166 | 0.9 | 18.73 | 0.511693 | 0.8 | 19.62 | 0.615611 | 1.7 | 19.23 | 0.567178 | 1.1 | 19.71 | 0.622746 | 1.8 | 19.65 | 0.584339 | 1.5 |
| 591 | Far upstream element-binding protein 1 OS=Homo sapiens GN=FUBP1 PE=1 SV=3 | FUBP1_HUMAN | 68 kDa | TRUE | 0.0001 | 1.45 | 1.45 | 1.65 | 17.5 | 0.822805 | 17.45 | 0.820925 | 17.97 | 0.522988 | 1.4 | 17.95 | 0.509913 | 1.5 | 18.01 | 0.512487 | 1.5 | 17.88 | 0.506672 | 1.4 | 18.19 | 0.585129 | 1.7 | 18.09 | 0.525714 | 1.6 |
| 240 | Cytosolic non-specific dipeptidase OS=Homo sapiens GN=CNDP2 PE=1 SV=2 | CNDP2_HUMAN | 53 kDa |  | 0.0001 | 1.30 | 1.45 | 1.65 | 17.88 | 0.688156 | 17.89 | 0.684369 | 18.19 | 0.562208 | 1.3 | 18.22 | 0.551635 | 1.3 | 18.45 | 0.558371 | 1.5 | 18.38 | 0.570087 | 1.4 | 18.62 | 0.563902 | 1.7 | 18.55 | 0.56332 | 1.6 |
| 821 | EH domain-containing protein 4 OS=Homo sapiens GN=EHD4 PE=1 SV=1 | EHD4_HUMAN | 61 kDa | TRUE | 0.0029 | 1.25 | 1.45 | 1.65 | 18.34 | 0.912752 | 18.18 | 0.927531 | 18.54 | 0.558514 | 1.2 | 18.62 | 0.533609 | 1.3 | 18.77 | 0.524443 | 1.4 | 18.78 | 0.561303 | 1.5 | 19 | 0.55117 | 1.6 | 18.97 | 0.566911 | 1.7 |
| 1448 | Heterogeneous nuclear ribonucleoprotein H2 OS=Homo sapiens GN=HNRNPH2 PE=1 SV=1 | HNRH2_HUMAN | 49 kDa | TRUE | 0.0067 | 1.25 | 1.45 | 1.65 | 17.37 | 0.603954 | 17.22 | 0.680659 | 17.64 | 0.512182 | 1.2 | 17.61 | 0.498235 | 1.3 | 17.81 | 0.498662 | 1.4 | 17.78 | 0.517487 | 1.5 | 17.97 | 0.492376 | 1.5 | 18.05 | 0.499997 | 1.8 |
| 441 | Poly(rC)-binding protein 1 OS=Homo sapiens GN=PCBP1 PE=1 SV=2 | PCBP1_HUMAN | 37 kDa | TRUE | 0.00041 | 1.15 | 1.45 | 1.65 | 18.88 | 0.952293 | 19.02 | 0.810772 | 19.16 | 0.585276 | 1.2 | 19.22 | 0.583568 | 1.1 | 19.49 | 0.527152 | 1.5 | 19.48 | 0.567273 | 1.4 | 19.61 | 0.533413 | 1.7 | 19.64 | 0.576159 | 1.6 |
| 382 | Heat shock protein beta-1 OS=Homo sapiens GN=HSPB1 PE=1 SV=2 | HSPB1_HUMAN | 23 kDa |  | 0.0001 | 1.05 | 1.45 | 1.65 | 18.46 | 0.675385 | 18.37 | 0.631552 | 18.45 | 0.628061 | 1 | 18.46 | 0.604634 | 1.1 | 18.91 | 0.584955 | 1.4 | 18.95 | 0.574685 | 1.5 | 19.13 | 0.610844 | 1.6 | 19.18 | 0.604905 | 1.7 |
| 1166 | Transcriptional activator protein Pur-beta OS=Homo sapiens GN=PURB PE=1 SV=3 | PURB_HUMAN | 33 kDa | TRUE | 0.011 | 1.05 | 1.45 | 1.65 | 18 | 0.533413 | 18.15 | 0.535501 | 18.15 | 0.511461 | 1.1 | 18.12 | 0.517436 | 1 | 18.59 | 0.525265 | 1.5 | 18.67 | 0.535217 | 1.4 | 18.85 | 0.499313 | 1.8 | 18.76 | 0.574378 | 1.5 |
| 1807 | Mitochondrial enolase superfamily member 1 OS=Homo sapiens GN=ENOSF1 PE=1 SV=1 | ENOF1_HUMAN | 50 kDa |  | 0.011 | 1.20 | 1.50 | 1.65 | 16.76 | 0.504526 | 16.5 | 0.529108 | 16.86 | 0.488937 | 1.1 | 16.87 | 0.486641 | 1.3 | 17.2 | 0.488734 | 1.4 | 17.18 | 0.493579 | 1.6 | 17.31 | 0.484802 | 1.5 | 17.4 | 0.485461 | 1.8 |
| 1214 | Tubulin-folding cofactor B OS=Homo sapiens GN=TBCB PE=1 SV=2 | TBCB_HUMAN | 27 kDa | TRUE | 0.00013 | 1.00 | 1.50 | 1.65 | 17.67 | 0.533534 | 17.51 | 0.635544 | 17.47 | 0.75671 | 0.9 | 17.54 | 0.752467 | 1.1 | 18.15 | 0.503906 | 1.4 | 18.2 | 0.574735 | 1.6 | 18.28 | 0.541054 | 1.5 | 18.31 | 0.536357 | 1.8 |
| 348 | Importin-5 OS=Homo sapiens GN=IPO5 PE=1 SV=4 | IPO5_HUMAN | 124 kDa | TRUE | 0.0001 | 1.75 | 1.55 | 1.65 | 17.59 | 0.91023 | 17.42 | 0.86957 | 18.21 | 0.590741 | 1.6 | 18.26 | 0.605703 | 1.9 | 18.07 | 0.538437 | 1.4 | 18.15 | 0.518977 | 1.7 | 18.11 | 0.525395 | 1.5 | 18.25 | 0.53064 | 1.8 |
| 1723 | Echinoderm microtubule-associated protein-like 4 OS=Homo sapiens GN=EML4 PE=1 SV=3 | EMAL4_HUMAN | 109 kDa |  | 0.019 | 1.15 | 1.55 | 1.65 | 18.2 | 0.564858 | 18.19 | 0.592905 | 18.39 | 0.505699 | 1.1 | 18.47 | 0.506976 | 1.2 | 18.68 | 0.504128 | 1.4 | 18.98 | 0.592986 | 1.7 | 18.87 | 0.50642 | 1.6 | 18.98 | 0.512863 | 1.7 |
| 541 | Calpastatin OS=Homo sapiens GN=CAST PE=1 SV=4 | ICAL_HUMAN | 77 kDa |  | 0.0002 | 1.60 | 1.55 | 1.65 | 17.71 | 0.80209 | 17.84 | 0.930687 | 18.34 | 0.583524 | 1.6 | 18.44 | 0.534427 | 1.6 | 18.34 | 0.570556 | 1.6 | 18.29 | 0.577425 | 1.5 | 18.42 | 0.534504 | 1.6 | 18.54 | 0.674526 | 1.7 |
| 269 | V-type proton ATPase subunit B, brain isoform OS=Homo sapiens GN=ATP6V1B2 PE=1 SV=3 | VATB2_HUMAN | 57 kDa |  | 0.0001 | 1.55 | 1.55 | 1.65 | 17.44 | 0.723782 | 17.51 | 0.738077 | 18.08 | 0.54635 | 1.6 | 18.08 | 0.539063 | 1.5 | 18.06 | 0.533151 | 1.5 | 18.15 | 0.53806 | 1.6 | 18.13 | 0.518918 | 1.6 | 18.28 | 0.589528 | 1.7 |
| 275 | AP-2 complex subunit alpha-1 OS=Homo sapiens GN=AP2A1 PE=1 SV=3 | AP2A1_HUMAN | 108 kDa | TRUE | 0.0001 | 1.55 | 1.55 | 1.65 | 17.71 | 0.803798 | 17.76 | 0.74357 | 18.35 | 0.545882 | 1.6 | 18.36 | 0.527721 | 1.5 | 18.39 | 0.537631 | 1.6 | 18.36 | 0.525795 | 1.5 | 18.4 | 0.513105 | 1.6 | 18.48 | 0.518365 | 1.7 |
| 383 | Leucine-rich repeat flightless-interacting protein 1 OS=Homo sapiens GN=LRRFIP1 PE=1 SV=2 | LRRF1_HUMAN | 89 kDa | TRUE | 0.0001 | 1.55 | 1.55 | 1.65 | 17.7 | 0.798966 | 17.83 | 0.736586 | 18.33 | 0.539914 | 1.6 | 18.37 | 0.559547 | 1.5 | 18.36 | 0.541744 | 1.6 | 18.38 | 0.564872 | 1.5 | 18.42 | 0.537276 | 1.7 | 18.49 | 0.560162 | 1.6 |
| 637 | Xaa-Pro aminopeptidase 1 OS=Homo sapiens GN=XPNPEP1 PE=1 SV=3 | XPP1_HUMAN | 70 kDa |  | 0.0001 | 1.50 | 1.55 | 1.65 | 17.59 | 0.90304 | 17.6 | 0.769524 | 18.16 | 0.548137 | 1.5 | 18.15 | 0.529086 | 1.5 | 18.26 | 0.519912 | 1.6 | 18.14 | 0.527681 | 1.5 | 18.28 | 0.530792 | 1.6 | 18.31 | 0.538586 | 1.7 |
| 1095 | Suppressor of G2 allele of SKP1 homolog OS=Homo sapiens GN=SUGT1 PE=1 SV=3 | SUGT1_HUMAN | ? | TRUE | 0.017 | 1.45 | 1.55 | 1.65 | 17.19 | 1.156338 | 17.1 | 1.015056 | 17.7 | 0.509569 | 1.4 | 17.72 | 0.501377 | 1.5 | 17.81 | 0.499083 | 1.5 | 17.77 | 0.513657 | 1.6 | 17.86 | 0.505203 | 1.6 | 17.82 | 0.507503 | 1.7 |
| 193 | C-1-tetrahydrofolate synthase, cytoplasmic OS=Homo sapiens GN=MTHFD1 PE=1 SV=3 | C1TC_HUMAN | 102 kDa |  | 0.0001 | 1.40 | 1.55 | 1.65 | 17.92 | 0.856476 | 17.91 | 0.726762 | 18.33 | 0.551574 | 1.4 | 18.35 | 0.548117 | 1.4 | 18.55 | 0.544212 | 1.6 | 18.47 | 0.533929 | 1.5 | 18.53 | 0.529998 | 1.6 | 18.62 | 0.539057 | 1.7 |
| 644 | Glutaredoxin-3 OS=Homo sapiens GN=GLRX3 PE=1 SV=2 | GLRX3_HUMAN | 37 kDa |  | 0.0001 | 1.35 | 1.55 | 1.65 | 18.03 | 0.969562 | 17.8 | 0.824026 | 18.31 | 0.535052 | 1.2 | 18.33 | 0.548027 | 1.5 | 18.53 | 0.55077 | 1.5 | 18.44 | 0.565165 | 1.6 | 18.66 | 0.531267 | 1.6 | 18.57 | 0.531098 | 1.7 |
| 175 | T-complex protein 1 subunit gamma OS=Homo sapiens GN=CCT3 PE=1 SV=4 | TCPG_HUMAN | 61 kDa |  | 0.0001 | 1.75 | 1.60 | 1.65 | 18.13 | 0.841295 | 18.14 | 0.776881 | 18.88 | 0.628213 | 1.8 | 18.86 | 0.648641 | 1.7 | 18.82 | 0.55843 | 1.6 | 18.79 | 0.536133 | 1.6 | 18.81 | 0.526095 | 1.6 | 18.84 | 0.540284 | 1.7 |
| 1200 | Optineurin OS=Homo sapiens GN=OPTN PE=1 SV=2 | OPTN_HUMAN | 66 kDa | TRUE | 0.0001 | 1.60 | 1.60 | 1.65 | 16.46 | 0.615863 | 16.47 | 0.705428 | 17.06 | 0.544817 | 1.5 | 17.2 | 0.502279 | 1.7 | 17.13 | 0.491549 | 1.6 | 17.13 | 0.498129 | 1.6 | 17.1 | 0.488129 | 1.6 | 17.21 | 0.520938 | 1.7 |
| 471 | Proteasome subunit beta type-8 OS=Homo sapiens GN=PSMB8 PE=1 SV=3 | PSB8_HUMAN | 30 kDa |  | 0.0017 | 1.55 | 1.60 | 1.65 | 18.28 | 0.861767 | 18.32 | 0.868798 | 18.92 | 0.568431 | 1.6 | 18.89 | 0.561517 | 1.5 | 18.98 | 0.554364 | 1.7 | 18.87 | 0.550422 | 1.5 | 19.02 | 0.560407 | 1.7 | 18.94 | 0.600204 | 1.6 |
| 685 | Endophilin-B2 OS=Homo sapiens GN=SH3GLB2 PE=1 SV=1 | SHLB2_HUMAN | 44 kDa | TRUE | 0.0001 | 1.55 | 1.60 | 1.65 | 18.12 | 0.79826 | 18.02 | 0.85166 | 18.57 | 0.514632 | 1.4 | 18.74 | 0.520313 | 1.7 | 18.65 | 0.533345 | 1.5 | 18.7 | 0.544866 | 1.7 | 18.8 | 0.516005 | 1.6 | 18.76 | 0.518202 | 1.7 |
| 370 | Protein disulfide-isomerase A6 OS=Homo sapiens GN=PDIA6 PE=1 SV=1 | PDIA6_HUMAN | 48 kDa |  | 0.0001 | 1.45 | 1.60 | 1.65 | 17.37 | 1.02398 | 17.36 | 0.999841 | 17.9 | 0.566085 | 1.4 | 17.99 | 0.562695 | 1.5 | 17.95 | 0.514995 | 1.6 | 18.02 | 0.529238 | 1.6 | 18.03 | 0.507908 | 1.6 | 18.05 | 0.541194 | 1.7 |
| 855 | Calumenin OS=Homo sapiens GN=CALU PE=1 SV=2 | CALU_HUMAN | 37 kDa |  | 0.004 | 1.45 | 1.60 | 1.65 | 17.52 | 1.039536 | 17.54 | 0.954562 | 18.08 | 0.506809 | 1.5 | 18.08 | 0.503816 | 1.4 | 18.32 | 0.51854 | 1.7 | 18.14 | 0.566886 | 1.5 | 18.27 | 0.516064 | 1.7 | 18.17 | 0.541474 | 1.6 |
| 598 | cAMP-dependent protein kinase type I-alpha regulatory subunit OS=Homo sapiens GN=PRKAR1A PE=1 SV=1 | KAP0_HUMAN | 43 kDa | TRUE | 0.011 | 1.40 | 1.60 | 1.65 | 17.34 | 1.164713 | 17.44 | 1.174102 | 17.77 | 0.53205 | 1.4 | 17.8 | 0.544923 | 1.4 | 18.04 | 0.562813 | 1.7 | 17.95 | 0.534471 | 1.5 | 17.95 | 0.542496 | 1.6 | 18.07 | 0.565479 | 1.7 |
| 905 | Histidine triad nucleotide-binding protein 1 OS=Homo sapiens GN=HINT1 PE=1 SV=2 | HINT1_HUMAN | 14 kDa |  | 0.011 | 1.35 | 1.60 | 1.65 | 18.2 | 0.878018 | 18.26 | 0.893804 | 18.6 | 0.566805 | 1.4 | 18.55 | 0.555703 | 1.3 | 18.92 | 0.550026 | 1.7 | 18.79 | 0.531674 | 1.5 | 18.87 | 0.550978 | 1.7 | 18.83 | 0.56853 | 1.6 |
| 431 | 26S proteasome non-ATPase regulatory subunit 11 OS=Homo sapiens GN=PSMD11 PE=1 SV=3 | PSD11_HUMAN | 47 kDa |  | 0.0001 | 1.70 | 1.65 | 1.65 | 17.74 | 0.897828 | 17.75 | 0.98449 | 18.5 | 0.600706 | 1.8 | 18.38 | 0.576603 | 1.6 | 18.5 | 0.577107 | 1.7 | 18.42 | 0.529929 | 1.6 | 18.53 | 0.544003 | 1.7 | 18.48 | 0.54377 | 1.6 |
| 166 | Ubiquitin carboxyl-terminal hydrolase 5 OS=Homo sapiens GN=USP5 PE=1 SV=2 | UBP5_HUMAN | 96 kDa |  | 0.0001 | 1.70 | 1.65 | 1.65 | 17.55 | 0.895719 | 17.59 | 0.829302 | 18.26 | 0.565709 | 1.7 | 18.27 | 0.575963 | 1.7 | 18.28 | 0.543194 | 1.7 | 18.21 | 0.529482 | 1.6 | 18.3 | 0.526428 | 1.7 | 18.28 | 0.536022 | 1.6 |
| 1467 | Cell division cycle and apoptosis regulator protein 1 OS=Homo sapiens GN=CCAR1 PE=1 SV=2 | CCAR1_HUMAN | 133 kDa |  | 0.0056 | 1.40 | 1.65 | 1.65 | 17.27 | 0.899177 | 17.2 | 0.833855 | 17.69 | 0.514863 | 1.4 | 17.69 | 0.496448 | 1.4 | 17.92 | 0.493159 | 1.6 | 17.88 | 0.489277 | 1.7 | 17.91 | 0.488083 | 1.6 | 17.91 | 0.49258 | 1.7 |
| 1679 | Selenide, water dikinase 1 OS=Homo sapiens GN=SEPHS1 PE=1 SV=2 | SPS1_HUMAN | 43 kDa |  | 0.019 | 1.35 | 1.65 | 1.65 | 16.78 | 0.495998 | 16.92 | 0.506205 | 17.29 | 0.497691 | 1.4 | 17.32 | 0.494016 | 1.3 | 17.59 | 0.490262 | 1.8 | 17.46 | 0.526712 | 1.5 | 17.53 | 0.490176 | 1.7 | 17.58 | 0.499761 | 1.6 |
| 609 | Threonine--tRNA ligase, cytoplasmic OS=Homo sapiens GN=TARS PE=1 SV=3 | SYTC_HUMAN | 83 kDa |  | 0.0001 | 1.30 | 1.65 | 1.65 | 17.39 | 0.715899 | 17.21 | 0.682535 | 17.64 | 0.528908 | 1.2 | 17.66 | 0.553495 | 1.4 | 17.97 | 0.546264 | 1.5 | 18.01 | 0.606227 | 1.8 | 17.97 | 0.537919 | 1.5 | 18.05 | 0.642666 | 1.8 |
| 400 | Syntaxin-binding protein 2 OS=Homo sapiens GN=STXBP2 PE=1 SV=2 | STXB2_HUMAN | 66 kDa | TRUE | 0.0001 | 1.80 | 1.70 | 1.65 | 17.6 | 0.934922 | 17.59 | 0.921958 | 18.48 | 0.566205 | 1.9 | 18.41 | 0.534601 | 1.7 | 18.36 | 0.521337 | 1.7 | 18.39 | 0.561909 | 1.7 | 18.25 | 0.511575 | 1.6 | 18.28 | 0.556045 | 1.7 |
| 1030 | CTP synthase 1 OS=Homo sapiens GN=CTPS1 PE=1 SV=2 | PYRG1_HUMAN | 67 kDa |  | 0.013 | 1.50 | 1.70 | 1.65 | 16.97 | 0.835883 | 17.02 | 0.860897 | 17.45 | 0.583982 | 1.5 | 17.53 | 0.558558 | 1.5 | 17.59 | 0.549345 | 1.5 | 17.75 | 0.790112 | 1.9 | 17.65 | 0.517655 | 1.6 | 17.78 | 0.521868 | 1.7 |
| 225 | Bifunctional purine biosynthesis protein PURH OS=Homo sapiens GN=ATIC PE=1 SV=3 | PUR9_HUMAN | 65 kDa |  | 0.0001 | 1.30 | 1.70 | 1.65 | 17.73 | 0.902323 | 17.73 | 0.837217 | 18.12 | 0.572552 | 1.3 | 18.09 | 0.569235 | 1.3 | 18.46 | 0.572731 | 1.7 | 18.39 | 0.538707 | 1.7 | 18.42 | 0.555376 | 1.7 | 18.4 | 0.574258 | 1.6 |
| 1675 | LanC-like protein 1 OS=Homo sapiens GN=LANCL1 PE=1 SV=1 | LANC1_HUMAN | 45 kDa |  | 0.011 | 1.45 | 1.70 | 1.65 | 18.18 | 0.520217 | 18.03 | 0.549239 | 18.65 | 0.500376 | 1.4 | 18.6 | 0.506232 | 1.5 | 18.88 | 0.505865 | 1.6 | 18.91 | 0.503698 | 1.8 | 18.81 | 0.50517 | 1.6 | 18.8 | 0.500138 | 1.7 |
| 425 | Rab GDP dissociation inhibitor beta OS=Homo sapiens GN=GDI2 PE=1 SV=2 | GDIB_HUMAN | 51 kDa | TRUE | 0.0001 | 1.55 | 1.75 | 1.65 | 17.49 | 0.725713 | 17.54 | 0.697835 | 18.12 | 0.645521 | 1.5 | 18.14 | 0.646331 | 1.6 | 18.28 | 0.534405 | 1.8 | 18.26 | 0.518638 | 1.7 | 18.17 | 0.570416 | 1.7 | 18.2 | 0.58327 | 1.6 |
| 91 | Actin, alpha cardiac muscle 1 OS=Homo sapiens GN=ACTC1 PE=1 SV=1 | ACTC_HUMAN | 42 kDa | TRUE | 0.00026 | 1.35 | 1.75 | 1.65 | 17.25 | 0.919254 | 17.27 | 0.964106 | 17.71 | 0.569702 | 1.4 | 17.61 | 0.562539 | 1.3 | 18.02 | 0.656503 | 1.9 | 17.89 | 0.545842 | 1.6 | 17.96 | 0.620076 | 1.7 | 17.94 | 0.581456 | 1.6 |
| 238 | Lymphocyte-specific protein 1 OS=Homo sapiens GN=LSP1 PE=1 SV=1 | LSP1_HUMAN | 37 kDa |  | 0.0001 | 1.80 | 1.80 | 1.65 | 18.04 | 0.909561 | 18.15 | 0.843987 | 18.92 | 0.59647 | 1.8 | 18.98 | 0.589516 | 1.8 | 18.94 | 0.567663 | 1.9 | 18.83 | 0.575492 | 1.7 | 18.77 | 0.589432 | 1.7 | 18.74 | 0.582499 | 1.6 |
| 664 | Ubiquitin-like modifier-activating enzyme ATG7 OS=Homo sapiens GN=ATG7 PE=1 SV=1 | ATG7_HUMAN | 78 kDa |  | 0.0001 | 2.10 | 1.80 | 1.65 | 17.68 | 0.833769 | 17.57 | 0.82929 | 18.67 | 0.568014 | 2 | 18.69 | 0.576793 | 2.2 | 18.5 | 0.505788 | 1.8 | 18.4 | 0.51193 | 1.8 | 18.36 | 0.509555 | 1.6 | 18.33 | 0.533761 | 1.7 |
| 1211 | Serine/arginine-rich splicing factor 7 OS=Homo sapiens GN=SRSF7 PE=1 SV=1 | SRSF7_HUMAN | 27 kDa | TRUE | 0.016 | 1.65 | 1.85 | 1.65 | 17.44 | 0.873895 | 17.51 | 0.772454 | 18.14 | 0.537874 | 1.7 | 18.12 | 0.563726 | 1.6 | 18.04 | 0.508955 | 1.6 | 18.42 | 0.682766 | 2.1 | 18.12 | 0.502449 | 1.6 | 18.28 | 0.66158 | 1.7 |
| 1282 | Septin-5 OS=Homo sapiens GN=SEPT5 PE=1 SV=1 | SEPT5_HUMAN | 43 kDa | TRUE | 0.0038 | 2.10 | 1.90 | 1.65 | 18.07 | 0.650841 | 17.92 | 0.615314 | 19.03 | 0.546784 | 2 | 19.06 | 0.539275 | 2.2 | 18.91 | 0.506152 | 1.8 | 18.91 | 0.50178 | 2 | 18.7 | 0.514147 | 1.6 | 18.7 | 0.500014 | 1.7 |
| 547 | Peroxiredoxin-5, mitochondrial OS=Homo sapiens GN=PRDX5 PE=1 SV=4 | PRDX5_HUMAN | 22 kDa |  | 0.0001 | 1.65 | 1.90 | 1.65 | 18.13 | 0.822629 | 18.07 | 0.801981 | 18.8 | 0.639324 | 1.6 | 18.81 | 0.634114 | 1.7 | 18.92 | 0.571633 | 1.8 | 19.01 | 0.581558 | 2 | 18.76 | 0.543635 | 1.6 | 18.81 | 0.584817 | 1.7 |
| 122 | Alpha-enolase OS=Homo sapiens GN=ENO1 PE=1 SV=2 | ENOA_HUMAN | 47 kDa | TRUE | 0.0001 | 1.30 | 1.90 | 1.65 | 17.8 | 0.859308 | 17.79 | 0.802749 | 18.11 | 0.535282 | 1.3 | 18.11 | 0.542062 | 1.3 | 18.71 | 0.637223 | 2 | 18.53 | 0.601519 | 1.8 | 18.51 | 0.555506 | 1.7 | 18.44 | 0.5523 | 1.6 |
| 105 | Plastin-2 OS=Homo sapiens GN=LCP1 PE=1 SV=6 | PLSL_HUMAN | 70 kDa | TRUE | 0.0001 | 2.00 | 1.95 | 1.65 | 17.61 | 0.936766 | 17.64 | 0.887477 | 18.57 | 0.655404 | 2 | 18.6 | 0.644784 | 2 | 18.58 | 0.57833 | 2 | 18.51 | 0.539877 | 1.9 | 18.35 | 0.536154 | 1.7 | 18.37 | 0.576001 | 1.6 |
| 379 | Coatomer subunit beta OS=Homo sapiens GN=COPB1 PE=1 SV=3 | COPB_HUMAN | 107 kDa | TRUE | 0.0001 | 1.55 | 1.45 | 1.70 | 18.04 | 0.740423 | 18.08 | 0.716173 | 18.69 | 0.618572 | 1.6 | 18.67 | 0.582684 | 1.5 | 18.53 | 0.590667 | 1.5 | 18.53 | 0.594024 | 1.4 | 18.75 | 0.557497 | 1.7 | 18.8 | 0.581706 | 1.7 |
| 317 | 14-3-3 protein theta OS=Homo sapiens GN=YWHAQ PE=1 SV=1 | 1433T_HUMAN | 28 kDa | TRUE | 0.0001 | 1.35 | 1.45 | 1.70 | 16.96 | 0.74845 | 16.97 | 0.69062 | 17.37 | 0.652596 | 1.3 | 17.41 | 0.650151 | 1.4 | 17.54 | 0.576767 | 1.5 | 17.45 | 0.589417 | 1.4 | 17.66 | 0.555402 | 1.7 | 17.67 | 0.584945 | 1.7 |
| 582 | Serpin H1 OS=Homo sapiens GN=SERPINH1 PE=1 SV=2 | SERPH_HUMAN | 46 kDa |  | 0.0001 | 1.30 | 1.45 | 1.70 | 17.78 | 0.761834 | 17.7 | 0.692369 | 18.06 | 0.617336 | 1.3 | 17.99 | 0.588134 | 1.3 | 18.27 | 0.606424 | 1.4 | 18.27 | 0.540566 | 1.5 | 18.38 | 0.559472 | 1.5 | 18.57 | 0.635513 | 1.9 |
| 1052 | Biliverdin reductase A OS=Homo sapiens GN=BLVRA PE=1 SV=2 | BIEA_HUMAN | 33 kDa |  | 0.0001 | 1.25 | 1.45 | 1.70 | 16.17 | 0.632751 | 16.47 | 0.528925 | 16.66 | 0.496968 | 1.4 | 16.61 | 0.515987 | 1.1 | 16.78 | 0.680167 | 1.5 | 16.95 | 0.545814 | 1.4 | 16.97 | 0.518694 | 1.7 | 17.26 | 0.563286 | 1.7 |
| 1008 | Eukaryotic translation initiation factor 2 subunit 2 OS=Homo sapiens GN=EIF2S2 PE=1 SV=2 | IF2B_HUMAN | 38 kDa |  | 0.0001 | 1.15 | 1.45 | 1.70 | 18.73 | 0.601157 | 18.8 | 0.544256 | 18.97 | 0.527149 | 1.2 | 18.93 | 0.516086 | 1.1 | 19.36 | 0.541574 | 1.5 | 19.31 | 0.531414 | 1.4 | 19.45 | 0.587756 | 1.7 | 19.56 | 0.604259 | 1.7 |
| 10 | Actin, cytoplasmic 1 OS=Homo sapiens GN=ACTB PE=1 SV=1 | ACTB_HUMAN | 42 kDa | TRUE | 0.0001 | 1.30 | 1.50 | 1.70 | 16.98 | 0.863948 | 17.17 | 0.834989 | 17.42 | 0.678376 | 1.4 | 17.42 | 0.669331 | 1.2 | 17.6 | 0.720168 | 1.5 | 17.71 | 0.599793 | 1.5 | 17.69 | 0.593111 | 1.7 | 17.81 | 0.772711 | 1.7 |
| 416 | Proteasome subunit beta type-1 OS=Homo sapiens GN=PSMB1 PE=1 SV=2 | PSB1_HUMAN | 26 kDa |  | 0.0001 | 1.70 | 1.55 | 1.70 | 17.38 | 0.932955 | 17.4 | 0.85923 | 18.14 | 0.59044 | 1.7 | 18.17 | 0.608166 | 1.7 | 17.98 | 0.572651 | 1.5 | 18.04 | 0.544914 | 1.6 | 18.09 | 0.527562 | 1.7 | 18.14 | 0.604917 | 1.7 |
| 752 | ATP-dependent RNA helicase DDX1 OS=Homo sapiens GN=DDX1 PE=1 SV=2 | DDX1_HUMAN | 82 kDa | TRUE | 0.0001 | 1.60 | 1.55 | 1.70 | 17.7 | 0.690445 | 17.66 | 0.656623 | 18.31 | 0.527759 | 1.6 | 18.34 | 0.527952 | 1.6 | 18.32 | 0.536395 | 1.5 | 18.31 | 0.55284 | 1.6 | 18.45 | 0.524715 | 1.7 | 18.42 | 0.542541 | 1.7 |
| 1251 | DnaJ homolog subfamily B member 2 OS=Homo sapiens GN=DNAJB2 PE=1 SV=3 | DNJB2_HUMAN | 36 kDa |  | 0.0003 | 1.60 | 1.60 | 1.70 | 17.51 | 0.565619 | 17.45 | 0.597139 | 18.08 | 0.50688 | 1.5 | 18.18 | 0.507307 | 1.7 | 18.19 | 0.492676 | 1.6 | 18.17 | 0.495561 | 1.6 | 18.28 | 0.49379 | 1.7 | 18.22 | 0.4978 | 1.7 |
| 463 | COP9 signalosome complex subunit 4 OS=Homo sapiens GN=COPS4 PE=1 SV=1 | CSN4_HUMAN | 46 kDa | TRUE | 0.00021 | 1.50 | 1.60 | 1.70 | 18.15 | 0.942468 | 18.22 | 0.849541 | 18.8 | 0.593372 | 1.5 | 18.77 | 0.532015 | 1.5 | 18.82 | 0.518844 | 1.6 | 18.83 | 0.533998 | 1.6 | 18.92 | 0.534951 | 1.7 | 18.93 | 0.542827 | 1.7 |
| 764 | Proteasome subunit beta type-10 OS=Homo sapiens GN=PSMB10 PE=1 SV=1 | PSB10_HUMAN | 29 kDa |  | 0.00098 | 1.50 | 1.60 | 1.70 | 17.74 | 0.759316 | 17.88 | 0.854352 | 18.29 | 0.640977 | 1.5 | 18.49 | 0.592667 | 1.5 | 18.53 | 0.530842 | 1.7 | 18.47 | 0.540703 | 1.5 | 18.51 | 0.542093 | 1.7 | 18.51 | 0.61507 | 1.7 |
| 1003 | Porphobilinogen deaminase OS=Homo sapiens GN=HMBS PE=1 SV=2 | HEM3_HUMAN | 39 kDa |  | 0.0001 | 1.45 | 1.60 | 1.70 | 17.89 | 0.990173 | 17.74 | 0.85748 | 18.25 | 0.553892 | 1.4 | 18.2 | 0.519765 | 1.5 | 18.57 | 0.530922 | 1.6 | 18.44 | 0.51686 | 1.6 | 18.44 | 0.552618 | 1.5 | 18.56 | 0.633958 | 1.9 |
| 761 | Platelet-activating factor acetylhydrolase IB subunit alpha OS=Homo sapiens GN=PAFAH1B1 PE=1 SV=2 | LIS1_HUMAN | 47 kDa |  | 0.0001 | 1.30 | 1.60 | 1.70 | 18.17 | 0.774533 | 18.14 | 0.816591 | 18.51 | 0.587199 | 1.3 | 18.42 | 0.545892 | 1.3 | 18.92 | 0.554879 | 1.7 | 18.67 | 0.573477 | 1.5 | 18.96 | 0.573773 | 1.7 | 18.89 | 0.593744 | 1.7 |
| 785 | Dynamin-1-like protein OS=Homo sapiens GN=DNM1L PE=1 SV=2 | DNM1L_HUMAN | 82 kDa |  | 0.0001 | 1.30 | 1.60 | 1.70 | 17.88 | 0.619215 | 17.87 | 0.653089 | 18.3 | 0.523255 | 1.3 | 18.25 | 0.520764 | 1.3 | 18.51 | 0.518242 | 1.6 | 18.55 | 0.517374 | 1.6 | 18.63 | 0.532355 | 1.7 | 18.65 | 0.547312 | 1.7 |
| 171 | Serine/threonine-protein phosphatase 2A 65 kDa regulatory subunit A alpha isoform OS=Homo sapiens GN=PPP2R1A PE=1 SV=4 | 2AAA_HUMAN | 65 kDa | TRUE | 0.0001 | 1.65 | 1.65 | 1.70 | 17.63 | 0.771945 | 17.66 | 0.704694 | 18.28 | 0.611852 | 1.7 | 18.3 | 0.618161 | 1.6 | 18.28 | 0.603843 | 1.6 | 18.33 | 0.575881 | 1.7 | 18.37 | 0.535455 | 1.7 | 18.38 | 0.567674 | 1.7 |
| 224 | 26S protease regulatory subunit 7 OS=Homo sapiens GN=PSMC2 PE=1 SV=3 | PRS7_HUMAN | 49 kDa |  | 0.0001 | 1.65 | 1.65 | 1.70 | 17.8 | 0.942086 | 17.77 | 0.855589 | 18.46 | 0.590837 | 1.6 | 18.47 | 0.563651 | 1.7 | 18.52 | 0.540378 | 1.7 | 18.45 | 0.527209 | 1.6 | 18.53 | 0.537935 | 1.7 | 18.55 | 0.550815 | 1.7 |
| 197 | ATP-dependent 6-phosphofructokinase, liver type OS=Homo sapiens GN=PFKL PE=1 SV=6 | PFKAL_HUMAN | 85 kDa | TRUE | 0.0001 | 1.55 | 1.65 | 1.70 | 17.88 | 0.71965 | 17.87 | 0.710721 | 18.53 | 0.60597 | 1.5 | 18.53 | 0.597178 | 1.6 | 18.62 | 0.549893 | 1.7 | 18.52 | 0.560751 | 1.6 | 18.62 | 0.529253 | 1.7 | 18.67 | 0.586754 | 1.7 |
| 760 | Eukaryotic translation initiation factor 5 OS=Homo sapiens GN=EIF5 PE=1 SV=2 | IF5_HUMAN | 49 kDa |  | 0.0026 | 1.45 | 1.65 | 1.70 | 17.61 | 0.843164 | 17.61 | 0.753994 | 18.14 | 0.515946 | 1.5 | 18.09 | 0.523196 | 1.4 | 18.37 | 0.515319 | 1.7 | 18.32 | 0.505172 | 1.6 | 18.34 | 0.5777 | 1.7 | 18.39 | 0.520317 | 1.7 |
| 840 | Sorting nexin-2 OS=Homo sapiens GN=SNX2 PE=1 SV=2 | SNX2_HUMAN | 58 kDa | TRUE | 0.0001 | 1.40 | 1.65 | 1.70 | 17.71 | 0.890301 | 17.72 | 0.836113 | 18.2 | 0.52586 | 1.4 | 18.24 | 0.530837 | 1.4 | 18.38 | 0.539521 | 1.7 | 18.3 | 0.54012 | 1.6 | 18.48 | 0.521433 | 1.7 | 18.47 | 0.529436 | 1.7 |
| 299 | Proteasome subunit alpha type-5 OS=Homo sapiens GN=PSMA5 PE=1 SV=3 | PSA5_HUMAN | 26 kDa |  | 0.00023 | 1.70 | 1.70 | 1.70 | 17.38 | 1.041846 | 17.39 | 1.048615 | 18.07 | 0.578209 | 1.7 | 18.07 | 0.589253 | 1.7 | 18.05 | 0.537052 | 1.7 | 17.99 | 0.571156 | 1.7 | 18.09 | 0.532877 | 1.7 | 18.14 | 0.576964 | 1.7 |
| 281 | Protein-glutamine gamma-glutamyltransferase 2 OS=Homo sapiens GN=TGM2 PE=1 SV=2 | TGM2_HUMAN | 77 kDa |  | 0.0001 | 1.60 | 1.70 | 1.70 | 17.88 | 0.81919 | 17.86 | 0.813167 | 18.53 | 0.576035 | 1.6 | 18.56 | 0.573491 | 1.6 | 18.62 | 0.555997 | 1.7 | 18.56 | 0.524717 | 1.7 | 18.59 | 0.528006 | 1.7 | 18.6 | 0.57131 | 1.7 |
| 380 | 55 kDa erythrocyte membrane protein OS=Homo sapiens GN=MPP1 PE=1 SV=2 | EM55_HUMAN | 52 kDa |  | 0.00018 | 1.60 | 1.70 | 1.70 | 17.92 | 0.821577 | 17.87 | 0.838733 | 18.48 | 0.568995 | 1.5 | 18.59 | 0.610131 | 1.7 | 18.62 | 0.523014 | 1.7 | 18.61 | 0.529331 | 1.7 | 18.68 | 0.520634 | 1.7 | 18.61 | 0.549401 | 1.7 |
| 76 | Heat shock protein HSP 90-alpha OS=Homo sapiens GN=HSP90AA1 PE=1 SV=5 | HS90A_HUMAN | 85 kDa | TRUE | 0.0001 | 1.80 | 1.70 | 1.70 | 17.96 | 0.83242 | 17.99 | 0.838092 | 18.67 | 0.770032 | 1.8 | 18.67 | 0.764927 | 1.8 | 18.73 | 0.606147 | 1.8 | 18.64 | 0.555786 | 1.6 | 18.68 | 0.613556 | 1.7 | 18.69 | 0.676758 | 1.7 |
| 92 | Nicotinamide phosphoribosyltransferase OS=Homo sapiens GN=NAMPT PE=1 SV=1 | NAMPT_HUMAN | 56 kDa | TRUE | 0.0001 | 2.05 | 1.75 | 1.70 | 17.73 | 1.201005 | 17.76 | 1.098395 | 18.61 | 0.754072 | 2.1 | 18.59 | 0.797477 | 2 | 18.53 | 0.624185 | 1.8 | 18.47 | 0.568866 | 1.7 | 18.47 | 0.559697 | 1.7 | 18.44 | 0.59356 | 1.7 |
| 215 | Myeloid cell nuclear differentiation antigen OS=Homo sapiens GN=MNDA PE=1 SV=1 | MNDA_HUMAN | 46 kDa |  | 0.0017 | 1.85 | 1.75 | 1.70 | 18.19 | 0.918031 | 18.09 | 0.913254 | 19.02 | 0.597798 | 1.8 | 19.02 | 0.583041 | 1.9 | 19 | 0.564594 | 1.8 | 18.89 | 0.539111 | 1.7 | 18.93 | 0.566488 | 1.7 | 18.88 | 0.630238 | 1.7 |
| 480 | Sorbitol dehydrogenase OS=Homo sapiens GN=SORD PE=1 SV=4 | DHSO_HUMAN | 38 kDa | TRUE | 0.0001 | 1.75 | 1.75 | 1.70 | 17.07 | 0.789502 | 17.05 | 0.794373 | 17.84 | 0.56164 | 1.8 | 17.81 | 0.53492 | 1.7 | 17.81 | 0.525113 | 1.7 | 17.75 | 0.557415 | 1.8 | 17.79 | 0.528826 | 1.7 | 17.78 | 0.572493 | 1.7 |
| 593 | Glutathione S-transferase omega-1 OS=Homo sapiens GN=GSTO1 PE=1 SV=2 | GSTO1_HUMAN | 28 kDa |  | 0.0001 | 1.65 | 1.75 | 1.70 | 17.79 | 0.884689 | 17.85 | 0.843771 | 18.49 | 0.559348 | 1.6 | 18.6 | 0.658396 | 1.7 | 18.59 | 0.534224 | 1.8 | 18.52 | 0.530599 | 1.7 | 18.53 | 0.50798 | 1.7 | 18.6 | 0.521449 | 1.7 |
| 677 | Pyridoxal kinase OS=Homo sapiens GN=PDXK PE=1 SV=1 | PDXK_HUMAN | 35 kDa | TRUE | 0.0001 | 1.60 | 1.75 | 1.70 | 17.09 | 0.786963 | 17.22 | 0.76521 | 17.89 | 0.67901 | 1.7 | 17.79 | 0.703027 | 1.5 | 17.92 | 0.587314 | 1.8 | 17.89 | 0.578867 | 1.7 | 17.89 | 0.530455 | 1.7 | 17.97 | 0.629221 | 1.7 |
| 212 | Fructose-bisphosphate aldolase A OS=Homo sapiens GN=ALDOA PE=1 SV=2 | ALDOA_HUMAN | 39 kDa | TRUE | 0.0001 | 1.45 | 1.75 | 1.70 | 17.63 | 1.051062 | 17.61 | 1.072125 | 18.02 | 0.551478 | 1.4 | 18.11 | 0.606142 | 1.5 | 18.41 | 0.563399 | 1.8 | 18.28 | 0.52334 | 1.7 | 18.3 | 0.536458 | 1.7 | 18.29 | 0.549851 | 1.7 |
| 93 | Moesin OS=Homo sapiens GN=MSN PE=1 SV=3 | MOES_HUMAN | 68 kDa | TRUE | 0.0001 | 1.35 | 1.75 | 1.70 | 17.69 | 0.815747 | 17.64 | 0.856865 | 18.08 | 0.572288 | 1.3 | 18.13 | 0.568103 | 1.4 | 18.47 | 0.553609 | 1.8 | 18.4 | 0.535274 | 1.7 | 18.41 | 0.537711 | 1.7 | 18.38 | 0.561233 | 1.7 |
| 229 | Leukocyte elastase inhibitor OS=Homo sapiens GN=SERPINB1 PE=1 SV=1 | ILEU_HUMAN | 43 kDa | TRUE | 0.0001 | 1.70 | 1.80 | 1.70 | 18.14 | 0.965469 | 18.17 | 0.854383 | 18.91 | 0.695358 | 1.7 | 18.91 | 0.685879 | 1.7 | 19.02 | 0.619259 | 1.9 | 18.88 | 0.601621 | 1.7 | 18.8 | 0.569655 | 1.7 | 18.84 | 0.615066 | 1.7 |
| 624 | Rho GDP-dissociation inhibitor 2 OS=Homo sapiens GN=ARHGDIB PE=1 SV=3 | GDIR2_HUMAN | 23 kDa | TRUE | 0.0001 | 1.40 | 1.80 | 1.70 | 17.89 | 0.816143 | 17.87 | 0.783997 | 18.29 | 0.552182 | 1.4 | 18.29 | 0.547129 | 1.4 | 18.85 | 0.582836 | 1.9 | 18.62 | 0.628254 | 1.7 | 18.65 | 0.560045 | 1.7 | 18.65 | 0.576 | 1.7 |
| 481 | Ezrin OS=Homo sapiens GN=EZR PE=1 SV=4 | EZRI_HUMAN | 69 kDa | TRUE | 0.0001 | 1.70 | 1.85 | 1.70 | 17.91 | 0.824345 | 17.91 | 0.842494 | 18.64 | 0.568014 | 1.7 | 18.62 | 0.540266 | 1.7 | 18.75 | 0.557738 | 1.9 | 18.71 | 0.542742 | 1.8 | 18.64 | 0.54145 | 1.7 | 18.64 | 0.528344 | 1.7 |
| 334 | Isocitrate dehydrogenase [NADP] cytoplasmic OS=Homo sapiens GN=IDH1 PE=1 SV=2 | IDHC_HUMAN | 47 kDa | TRUE | 0.0001 | 1.70 | 1.90 | 1.70 | 17.79 | 0.732104 | 17.81 | 0.748832 | 18.49 | 0.596303 | 1.7 | 18.55 | 0.630364 | 1.7 | 18.73 | 0.545423 | 1.9 | 18.69 | 0.565497 | 1.9 | 18.55 | 0.519 | 1.7 | 18.54 | 0.58256 | 1.7 |
| 20 | Myosin-9 OS=Homo sapiens GN=MYH9 PE=1 SV=4 | MYH9_HUMAN | 227 kDa | TRUE | 0.0001 | 1.75 | 1.95 | 1.70 | 17.55 | 0.905739 | 17.59 | 0.852459 | 18.35 | 0.642082 | 1.8 | 18.37 | 0.639749 | 1.7 | 18.52 | 0.598011 | 2 | 18.44 | 0.571461 | 1.9 | 18.3 | 0.5583 | 1.7 | 18.32 | 0.571981 | 1.7 |
| 23 | Glyceraldehyde-3-phosphate dehydrogenase OS=Homo sapiens GN=GAPDH PE=1 SV=3 | G3P_HUMAN | 36 kDa | TRUE | 0.0001 | 1.70 | 2.05 | 1.70 | 17.44 | 0.997587 | 17.52 | 0.888846 | 18.16 | 0.768748 | 1.8 | 18.14 | 0.740034 | 1.6 | 18.41 | 0.737987 | 2.1 | 18.42 | 0.655144 | 2 | 18.15 | 0.65071 | 1.7 | 18.21 | 0.70814 | 1.7 |
| 50 | Periostin OS=Homo sapiens GN=POSTN PE=1 SV=2 | POSTN_HUMAN | 93 kDa | TRUE | 0.0001 | 1.45 | 2.10 | 1.70 | 17.94 | 0.921613 | 18 | 0.838326 | 18.46 | 0.660819 | 1.5 | 18.47 | 0.660421 | 1.4 | 18.95 | 0.694809 | 2.1 | 18.93 | 0.637744 | 2.1 | 18.6 | 0.588671 | 1.7 | 18.66 | 0.633582 | 1.7 |
| 1079 | Microtubule-associated protein 1S OS=Homo sapiens GN=MAP1S PE=1 SV=2 | MAP1S_HUMAN | 112 kDa |  | 0.0001 | 1.45 | 1.30 | 1.70 | 17.69 | 0.795994 | 17.67 | 0.886291 | 18.17 | 0.503181 | 1.4 | 18.17 | 0.511502 | 1.5 | 18.09 | 0.584367 | 1.3 | 18.06 | 0.539117 | 1.3 | 18.35 | 0.518672 | 1.6 | 18.39 | 0.596368 | 1.8 |
| 941 | EH domain-containing protein 2 OS=Homo sapiens GN=EHD2 PE=1 SV=2 | EHD2_HUMAN | 61 kDa | TRUE | 0.00058 | 1.10 | 1.45 | 1.70 | 17.31 | 0.834937 | 17.08 | 0.74325 | 17.28 | 0.515961 | 1 | 17.35 | 0.50518 | 1.2 | 17.73 | 0.513983 | 1.4 | 17.69 | 0.492325 | 1.5 | 17.96 | 0.576004 | 1.6 | 17.92 | 0.541705 | 1.8 |
| 492 | Proteasome subunit beta type-5 OS=Homo sapiens GN=PSMB5 PE=1 SV=3 | PSB5_HUMAN | 28 kDa | TRUE | 0.0001 | 1.50 | 1.55 | 1.70 | 17.98 | 0.803675 | 18.18 | 0.800801 | 18.65 | 0.565479 | 1.6 | 18.63 | 0.579645 | 1.4 | 18.78 | 0.534786 | 1.7 | 18.67 | 0.536453 | 1.4 | 18.8 | 0.553285 | 1.8 | 18.84 | 0.552614 | 1.6 |
| 542 | Eukaryotic translation initiation factor 2 subunit 1 OS=Homo sapiens GN=EIF2S1 PE=1 SV=3 | IF2A_HUMAN | 36 kDa |  | 0.0001 | 1.35 | 1.55 | 1.70 | 17.88 | 0.879192 | 17.96 | 0.850012 | 18.3 | 0.546565 | 1.4 | 18.31 | 0.543958 | 1.3 | 18.54 | 0.535134 | 1.6 | 18.47 | 0.526765 | 1.5 | 18.67 | 0.534055 | 1.8 | 18.59 | 0.551569 | 1.6 |
| 1011 | Immunoglobulin-binding protein 1 OS=Homo sapiens GN=IGBP1 PE=1 SV=1 | IGBP1_HUMAN | 39 kDa |  | 0.0027 | 1.40 | 1.60 | 1.70 | 17.46 | 1.073109 | 17.36 | 1.478471 | 17.77 | 0.507821 | 1.3 | 17.84 | 0.50401 | 1.5 | 17.98 | 0.533896 | 1.5 | 17.96 | 0.505276 | 1.7 | 18.15 | 0.535887 | 1.6 | 18.27 | 0.541666 | 1.8 |
| 1449 | Nuclear factor NF-kappa-B p100 subunit OS=Homo sapiens GN=NFKB2 PE=1 SV=4 | NFKB2_HUMAN | 97 kDa |  | 0.0097 | 1.30 | 1.60 | 1.70 | 17.16 | 0.543351 | 17.14 | 0.505419 | 17.77 | 0.48801 | 1.5 | 17.36 | 1.052534 | 1.1 | 17.73 | 0.482391 | 1.5 | 17.88 | 0.577207 | 1.7 | 17.84 | 0.489305 | 1.6 | 17.98 | 0.49581 | 1.8 |
| 207 | Polyubiquitin-C OS=Homo sapiens GN=UBC PE=1 SV=3 | UBC_HUMAN | 77 kDa |  | 0.0001 | 1.25 | 1.60 | 1.70 | 17.94 | 0.829082 | 17.74 | 0.773329 | 18.17 | 0.606051 | 1.2 | 18.19 | 0.657844 | 1.3 | 18.47 | 0.558119 | 1.5 | 18.45 | 0.571864 | 1.7 | 18.58 | 0.539742 | 1.6 | 18.56 | 0.551106 | 1.8 |
| 648 | Involucrin OS=Homo sapiens GN=IVL PE=1 SV=2 | INVO_HUMAN | 68 kDa |  | 0.0001 | 1.00 | 1.60 | 1.70 | 17.46 | 0.652757 | 17.34 | 0.808883 | 17.29 | 0.546344 | 0.9 | 17.35 | 0.559348 | 1.1 | 18.13 | 0.607606 | 1.6 | 17.97 | 0.524754 | 1.6 | 18.09 | 0.561817 | 1.6 | 18.12 | 0.553985 | 1.8 |
| 170 | T-complex protein 1 subunit delta OS=Homo sapiens GN=CCT4 PE=1 SV=4 | TCPD_HUMAN | 58 kDa |  | 0.0001 | 1.60 | 1.65 | 1.70 | 17.61 | 0.935568 | 17.59 | 0.823592 | 18.29 | 0.599704 | 1.6 | 18.3 | 0.598505 | 1.6 | 18.31 | 0.552331 | 1.7 | 18.31 | 0.577536 | 1.6 | 18.26 | 0.541086 | 1.6 | 18.36 | 0.580641 | 1.8 |
| 574 | COP9 signalosome complex subunit 3 OS=Homo sapiens GN=COPS3 PE=1 SV=3 | CSN3_HUMAN | 48 kDa |  | 0.0001 | 1.60 | 1.65 | 1.70 | 18.46 | 0.790503 | 18.6 | 0.844345 | 19.21 | 0.54445 | 1.7 | 19.18 | 0.531032 | 1.5 | 19.29 | 0.532661 | 1.8 | 19.11 | 0.524712 | 1.5 | 19.26 | 0.527014 | 1.8 | 19.21 | 0.530135 | 1.6 |
| 478 | Calponin-2 OS=Homo sapiens GN=CNN2 PE=1 SV=4 | CNN2_HUMAN | 34 kDa |  | 0.0001 | 2.00 | 1.75 | 1.70 | 19.02 | 1.018103 | 19.25 | 0.926683 | 20.1 | 0.560635 | 2.2 | 20.09 | 0.568907 | 1.8 | 19.98 | 0.550494 | 1.9 | 19.82 | 0.534487 | 1.6 | 19.8 | 0.531541 | 1.8 | 19.9 | 0.551289 | 1.6 |
| 159 | Actin-related protein 2/3 complex subunit 2 OS=Homo sapiens GN=ARPC2 PE=1 SV=1 | ARPC2_HUMAN | 34 kDa |  | 0.0001 | 2.00 | 1.80 | 1.70 | 17.7 | 0.816313 | 17.79 | 0.781701 | 18.68 | 0.62676 | 2 | 18.7 | 0.616851 | 2 | 18.57 | 0.574707 | 1.9 | 18.47 | 0.54665 | 1.7 | 18.48 | 0.529468 | 1.8 | 18.37 | 0.583096 | 1.6 |
| 52 | Alpha-actinin-4 OS=Homo sapiens GN=ACTN4 PE=1 SV=2 | ACTN4_HUMAN | 105 kDa | TRUE | 0.0001 | 1.85 | 1.80 | 1.70 | 17.78 | 0.901912 | 17.88 | 0.873195 | 18.64 | 0.694442 | 1.9 | 18.66 | 0.691567 | 1.8 | 18.67 | 0.581466 | 1.9 | 18.58 | 0.574255 | 1.7 | 18.54 | 0.569196 | 1.8 | 18.46 | 0.61888 | 1.6 |
| 1461 | Actin-related protein 10 OS=Homo sapiens GN=ACTR10 PE=1 SV=1 | ARP10_HUMAN | 46 kDa |  | 0.0097 | 1.85 | 1.85 | 1.70 | 15.57 | 0.559668 | 15.41 | 0.652368 | 16.33 | 0.500763 | 1.7 | 16.42 | 0.556691 | 2 | 16.41 | 0.479142 | 1.8 | 16.29 | 0.514168 | 1.9 | 16.26 | 0.478231 | 1.6 | 16.28 | 0.488783 | 1.8 |
| 1248 | Copine-1 OS=Homo sapiens GN=CPNE1 PE=1 SV=1 | CPNE1_HUMAN | 59 kDa |  | 0.0001 | 1.90 | 1.95 | 1.70 | 17.96 | 0.735384 | 17.81 | 0.945185 | 18.76 | 0.601315 | 1.7 | 18.9 | 0.616725 | 2.1 | 18.78 | 0.529892 | 1.8 | 18.87 | 0.644244 | 2.1 | 18.54 | 0.519913 | 1.6 | 18.58 | 0.536324 | 1.8 |
| 1565 | U1 small nuclear ribonucleoprotein A OS=Homo sapiens GN=SNRPA PE=1 SV=3 | SNRPA_HUMAN | 31 kDa | TRUE | 0.012 | 1.55 | 1.20 | 1.75 | 17.5 | 0.699885 | 17.39 | 0.695129 | 17.93 | 0.528565 | 1.4 | 18.1 | 0.489665 | 1.7 | 17.51 | 0.769978 | 1 | 17.79 | 0.501896 | 1.4 | 18.08 | 0.571351 | 1.5 | 18.36 | 0.492793 | 2 |
| 129 | Prelamin-A/C OS=Homo sapiens GN=LMNA PE=1 SV=1 | LMNA_HUMAN | 74 kDa | TRUE | 0.0001 | 1.10 | 1.25 | 1.75 | 17.72 | 0.825574 | 17.69 | 0.743644 | 17.85 | 0.558443 | 1.1 | 17.86 | 0.567428 | 1.1 | 18 | 0.565763 | 1.2 | 18.02 | 0.550115 | 1.3 | 18.49 | 0.675973 | 1.7 | 18.57 | 0.632066 | 1.8 |
| 1278 | 60S ribosomal protein L24 OS=Homo sapiens GN=RPL24 PE=1 SV=1 | RL24_HUMAN | 18 kDa |  | 0.0001 | 1.25 | 1.30 | 1.75 | 17.71 | 0.687481 | 17.58 | 0.641206 | 17.94 | 0.524228 | 1.2 | 17.94 | 0.534721 | 1.3 | 17.98 | 0.559177 | 1.2 | 18.05 | 0.549106 | 1.4 | 18.48 | 0.582438 | 1.7 | 18.49 | 0.622918 | 1.8 |
| 1773 | Lambda-crystallin homolog OS=Homo sapiens GN=CRYL1 PE=1 SV=3 | CRYL1_HUMAN | 35 kDa |  | 0.019 | 1.00 | 1.30 | 1.75 | 18.1 | 0.55268 | 18.18 | 0.519687 | 18.04 | 0.508726 | 1 | 18.23 | 0.541673 | 1 | 18.38 | 0.498645 | 1.2 | 18.62 | 0.539268 | 1.4 | 19.01 | 0.68731 | 1.9 | 18.82 | 0.542878 | 1.6 |
| 631 | 60S ribosomal protein L13a OS=Homo sapiens GN=RPL13A PE=1 SV=2 | RL13A_HUMAN | 24 kDa |  | 0.012 | 1.50 | 1.35 | 1.75 | 17.77 | 0.84562 | 17.56 | 0.670963 | 18.33 | 0.634353 | 1.5 | 18.19 | 0.592822 | 1.5 | 18.21 | 0.592965 | 1.3 | 18.09 | 0.589857 | 1.4 | 18.47 | 0.580241 | 1.6 | 18.45 | 0.554076 | 1.9 |
| 1429 | Polymerase I and transcript release factor OS=Homo sapiens GN=PTRF PE=1 SV=1 | PTRF_HUMAN | 43 kDa |  | 0.00015 | 1.45 | 1.40 | 1.75 | 17.66 | 0.617827 | 17.41 | 0.550568 | 18.1 | 0.506194 | 1.4 | 18.01 | 0.500627 | 1.5 | 18 | 0.498081 | 1.3 | 18.04 | 0.509536 | 1.5 | 18.3 | 0.509382 | 1.6 | 18.33 | 0.512906 | 1.9 |
| 924 | 60S ribosomal protein L35a OS=Homo sapiens GN=RPL35A PE=1 SV=2 | RL35A_HUMAN | 13 kDa |  | 0.0085 | 1.25 | 1.40 | 1.75 | 17.67 | 0.803154 | 17.66 | 0.937024 | 17.99 | 0.545189 | 1.3 | 17.94 | 0.585068 | 1.2 | 18.28 | 0.59244 | 1.5 | 18.09 | 0.519842 | 1.3 | 18.47 | 0.59258 | 1.8 | 18.28 | 0.741078 | 1.7 |
| 951 | LIM and senescent cell antigen-like-containing domain protein 1 OS=Homo sapiens GN=LIMS1 PE=1 SV=4 | LIMS1_HUMAN | 37 kDa |  | 0.0001 | 1.35 | 1.45 | 1.75 | 17.82 | 0.667538 | 17.62 | 0.6708 | 18.12 | 0.503336 | 1.3 | 18.07 | 0.527954 | 1.4 | 18.28 | 0.526278 | 1.4 | 18.23 | 0.592607 | 1.5 | 18.5 | 0.513392 | 1.6 | 18.52 | 0.515562 | 1.9 |
| 710 | 60S ribosomal protein L14 OS=Homo sapiens GN=RPL14 PE=1 SV=4 | RL14_HUMAN | 23 kDa | TRUE | 0.00064 | 1.30 | 1.45 | 1.75 | 17.59 | 0.614384 | 17.67 | 0.646226 | 17.94 | 0.549325 | 1.3 | 17.98 | 0.565822 | 1.3 | 18.12 | 0.570017 | 1.5 | 18.08 | 0.52281 | 1.4 | 18.48 | 0.543946 | 1.9 | 18.33 | 0.65088 | 1.6 |
| 509 | Thrombospondin-2 OS=Homo sapiens GN=THBS2 PE=1 SV=2 | TSP2_HUMAN | 130 kDa | TRUE | 0.0001 | 1.50 | 1.50 | 1.75 | 17.75 | 0.685259 | 17.74 | 0.715103 | 18.35 | 0.502641 | 1.5 | 18.38 | 0.505128 | 1.5 | 18.42 | 0.526901 | 1.6 | 18.3 | 0.512209 | 1.4 | 18.59 | 0.533997 | 1.8 | 18.5 | 0.526742 | 1.7 |
| 1161 | Serine/arginine-rich splicing factor 2 OS=Homo sapiens GN=SRSF2 PE=1 SV=4 | SRSF2_HUMAN | 25 kDa |  | 0.0081 | 1.45 | 1.50 | 1.75 | 18.75 | 0.763402 | 18.41 | 0.668222 | 19.09 | 0.517101 | 1.3 | 19.12 | 0.518807 | 1.6 | 19.16 | 0.521812 | 1.3 | 19.22 | 0.524177 | 1.7 | 19.26 | 0.525142 | 1.4 | 19.46 | 0.514658 | 2.1 |
| 424 | Coatomer subunit delta OS=Homo sapiens GN=ARCN1 PE=1 SV=1 | COPD_HUMAN | 57 kDa |  | 0.0001 | 1.45 | 1.50 | 1.75 | 18.13 | 0.728621 | 18.19 | 0.699714 | 18.64 | 0.557613 | 1.5 | 18.66 | 0.566515 | 1.4 | 18.76 | 0.566051 | 1.6 | 18.63 | 0.5457 | 1.4 | 18.97 | 0.58961 | 1.8 | 18.96 | 0.594722 | 1.7 |
| 990 | AH receptor-interacting protein OS=Homo sapiens GN=AIP PE=1 SV=2 | AIP_HUMAN | 38 kDa |  | 0.007 | 1.35 | 1.50 | 1.75 | 17.65 | 0.634512 | 17.8 | 0.823553 | 18.12 | 0.517738 | 1.4 | 18.11 | 0.517649 | 1.3 | 18.35 | 0.537423 | 1.6 | 18.28 | 0.515466 | 1.4 | 18.55 | 0.542552 | 1.9 | 18.5 | 0.574596 | 1.6 |
| 1071 | Heterogeneous nuclear ribonucleoprotein D-like OS=Homo sapiens GN=HNRNPDL PE=1 SV=3 | HNRDL_HUMAN | 46 kDa | TRUE | 0.0001 | 1.30 | 1.50 | 1.75 | 17.73 | 0.589186 | 17.55 | 0.594458 | 18 | 0.504026 | 1.2 | 18.06 | 0.507228 | 1.4 | 18.23 | 0.52379 | 1.4 | 18.23 | 0.548799 | 1.6 | 18.51 | 0.52188 | 1.7 | 18.42 | 0.532381 | 1.8 |
| 688 | Serine-threonine kinase receptor-associated protein OS=Homo sapiens GN=STRAP PE=1 SV=1 | STRAP_HUMAN | 38 kDa |  | 0.0001 | 1.25 | 1.50 | 1.75 | 18.08 | 0.771785 | 18.12 | 0.735839 | 18.43 | 0.519854 | 1.3 | 18.39 | 0.548505 | 1.2 | 18.61 | 0.581463 | 1.5 | 18.61 | 0.545755 | 1.5 | 18.85 | 0.592828 | 1.8 | 18.87 | 0.572999 | 1.7 |
| 1629 | PDZ domain-containing protein GIPC1 OS=Homo sapiens GN=GIPC1 PE=1 SV=2 | GIPC1_HUMAN | 36 kDa |  | 0.02 | 1.15 | 1.55 | 1.75 | 17.11 | 0.677656 | 16.9 | 0.704625 | 17.3 | 0.553086 | 1.1 | 17.15 | 0.490223 | 1.2 | 17.56 | 0.505422 | 1.4 | 17.61 | 0.530304 | 1.7 | 17.7 | 0.5093 | 1.6 | 17.83 | 0.508897 | 1.9 |
| 202 | Elongation factor 1-gamma OS=Homo sapiens GN=EEF1G PE=1 SV=3 | EF1G_HUMAN | 50 kDa |  | 0.0001 | 1.80 | 1.55 | 1.75 | 18.22 | 0.959296 | 18.29 | 0.904416 | 18.99 | 0.62702 | 1.8 | 19.05 | 0.622521 | 1.8 | 18.87 | 0.564161 | 1.6 | 18.81 | 0.54841 | 1.5 | 19.05 | 0.563519 | 1.8 | 19.08 | 0.601577 | 1.7 |
| 955 | Platelet-activating factor acetylhydrolase IB subunit beta OS=Homo sapiens GN=PAFAH1B2 PE=1 SV=1 | PA1B2_HUMAN | 26 kDa |  | 0.0001 | 1.60 | 1.55 | 1.75 | 17.29 | 0.657022 | 17.36 | 0.735872 | 17.97 | 0.514011 | 1.6 | 17.98 | 0.503362 | 1.6 | 17.91 | 0.507752 | 1.5 | 18.02 | 0.515195 | 1.6 | 18.08 | 0.506535 | 1.7 | 18.22 | 0.59896 | 1.8 |
| 177 | 14-3-3 protein gamma OS=Homo sapiens GN=YWHAG PE=1 SV=2 | 1433G_HUMAN | 28 kDa | TRUE | 0.0001 | 1.45 | 1.55 | 1.75 | 17.78 | 0.845449 | 17.76 | 0.908639 | 18.33 | 0.611289 | 1.4 | 18.32 | 0.616496 | 1.5 | 18.37 | 0.604249 | 1.5 | 18.35 | 0.591352 | 1.6 | 18.54 | 0.608697 | 1.7 | 18.59 | 0.596816 | 1.8 |
| 321 | NSFL1 cofactor p47 OS=Homo sapiens GN=NSFL1C PE=1 SV=2 | NSF1C_HUMAN | 41 kDa |  | 0.0001 | 1.40 | 1.55 | 1.75 | 17.49 | 0.80552 | 17.42 | 0.811218 | 17.87 | 0.543411 | 1.3 | 17.95 | 0.557787 | 1.5 | 18.04 | 0.535296 | 1.5 | 18.09 | 0.553032 | 1.6 | 18.22 | 0.550182 | 1.7 | 18.23 | 0.573534 | 1.8 |
| 329 | Polyadenylate-binding protein 1 OS=Homo sapiens GN=PABPC1 PE=1 SV=2 | PABP1_HUMAN | 71 kDa | TRUE | 0.0001 | 1.40 | 1.55 | 1.75 | 18.16 | 0.897591 | 18.27 | 0.850885 | 18.67 | 0.528473 | 1.4 | 18.68 | 0.560124 | 1.4 | 18.84 | 0.54525 | 1.6 | 18.81 | 0.533748 | 1.5 | 18.99 | 0.56691 | 1.8 | 19.05 | 0.564106 | 1.7 |
| 1358 | Protein Hook homolog 3 OS=Homo sapiens GN=HOOK3 PE=1 SV=2 | HOOK3_HUMAN | 83 kDa | TRUE | 0.0097 | 1.70 | 1.60 | 1.75 | 16.03 | 0.710597 | 16.02 | 0.778034 | 16.75 | 0.493452 | 1.7 | 16.74 | 0.501828 | 1.7 | 16.69 | 0.490693 | 1.6 | 16.69 | 0.507119 | 1.6 | 16.79 | 0.489425 | 1.7 | 16.87 | 0.496143 | 1.8 |
| 221 | 26S proteasome non-ATPase regulatory subunit 3 OS=Homo sapiens GN=PSMD3 PE=1 SV=2 | PSMD3_HUMAN | 61 kDa |  | 0.0001 | 1.50 | 1.60 | 1.75 | 17.98 | 0.947869 | 17.99 | 0.873561 | 18.53 | 0.628384 | 1.5 | 18.59 | 0.627904 | 1.5 | 18.71 | 0.567604 | 1.7 | 18.56 | 0.544382 | 1.5 | 18.76 | 0.559386 | 1.8 | 18.71 | 0.601506 | 1.7 |
| 911 | Myristoylated alanine-rich C-kinase substrate OS=Homo sapiens GN=MARCKS PE=1 SV=4 | MARCS_HUMAN | 32 kDa |  | 0.0001 | 1.50 | 1.60 | 1.75 | 16.56 | 0.631997 | 16.55 | 0.690517 | 17.12 | 0.517948 | 1.5 | 17.14 | 0.562135 | 1.5 | 17.22 | 0.52141 | 1.6 | 17.2 | 0.56552 | 1.6 | 17.32 | 0.525378 | 1.7 | 17.32 | 0.59153 | 1.8 |
| 214 | Heat shock cognate 71 kDa protein OS=Homo sapiens GN=HSPA8 PE=1 SV=1 | HSP7C_HUMAN | 71 kDa | TRUE | 0.0001 | 1.40 | 1.60 | 1.75 | 17.5 | 0.863609 | 17.56 | 0.810046 | 17.98 | 0.539555 | 1.4 | 18.01 | 0.540355 | 1.4 | 18.21 | 0.554377 | 1.7 | 18.11 | 0.57048 | 1.5 | 18.32 | 0.561626 | 1.8 | 18.31 | 0.57998 | 1.7 |
| 1444 | Kynureninase OS=Homo sapiens GN=KYNU PE=1 SV=1 | KYNU_HUMAN | 52 kDa |  | 0.014 | 1.10 | 1.60 | 1.75 | 17.95 | 1.044555 | 17.96 | 1.11118 | 18.16 | 0.551886 | 1.1 | 18.1 | 0.607196 | 1.1 | 18.32 | 0.496596 | 1.4 | 18.5 | 0.673498 | 1.8 | 18.67 | 0.498162 | 1.7 | 18.83 | 0.549387 | 1.8 |
| 373 | Sorting nexin-6 OS=Homo sapiens GN=SNX6 PE=1 SV=1 | SNX6_HUMAN | 47 kDa | TRUE | 0.02 | 1.75 | 1.65 | 1.75 | 18.1 | 1.092098 | 18.01 | 1.039511 | 18.73 | 0.648032 | 1.7 | 18.74 | 0.641791 | 1.8 | 18.73 | 0.521579 | 1.6 | 18.68 | 0.547778 | 1.7 | 18.93 | 0.523483 | 1.8 | 18.73 | 0.662423 | 1.7 |
| 560 | Interleukin enhancer-binding factor 2 OS=Homo sapiens GN=ILF2 PE=1 SV=2 | ILF2_HUMAN | 43 kDa |  | 0.0001 | 1.60 | 1.65 | 1.75 | 17.25 | 0.627031 | 17.28 | 0.72879 | 17.93 | 0.526215 | 1.6 | 17.94 | 0.521359 | 1.6 | 18 | 0.517911 | 1.7 | 17.91 | 0.536444 | 1.6 | 18.01 | 0.526454 | 1.7 | 18.13 | 0.534814 | 1.8 |
| 440 | Serine/threonine-protein kinase OSR1 OS=Homo sapiens GN=OXSR1 PE=1 SV=1 | OXSR1_HUMAN | 58 kDa | TRUE | 0.0001 | 1.55 | 1.65 | 1.75 | 18.01 | 0.874367 | 18.2 | 0.866136 | 18.69 | 0.617298 | 1.6 | 18.7 | 0.614359 | 1.5 | 18.79 | 0.558146 | 1.8 | 18.75 | 0.555902 | 1.5 | 18.8 | 0.525653 | 1.8 | 18.98 | 0.567478 | 1.7 |
| 205 | UV excision repair protein RAD23 homolog A OS=Homo sapiens GN=RAD23A PE=1 SV=1 | RD23A_HUMAN | 40 kDa | TRUE | 0.00013 | 1.50 | 1.65 | 1.75 | 17.45 | 0.899135 | 17.54 | 0.803253 | 18.07 | 0.595616 | 1.5 | 18.1 | 0.567628 | 1.5 | 18.11 | 0.603247 | 1.7 | 18.19 | 0.607029 | 1.6 | 18.23 | 0.546088 | 1.8 | 18.25 | 0.67531 | 1.7 |
| 619 | Coronin-7 OS=Homo sapiens GN=CORO7 PE=1 SV=2 | CORO7_HUMAN | 101 kDa |  | 0.0001 | 1.45 | 1.65 | 1.75 | 18.14 | 0.708039 | 18.22 | 0.747956 | 18.65 | 0.577634 | 1.5 | 18.67 | 0.551924 | 1.4 | 18.89 | 0.524784 | 1.7 | 18.86 | 0.607654 | 1.6 | 18.97 | 0.520225 | 1.8 | 19.01 | 0.546248 | 1.7 |
| 643 | Eukaryotic translation initiation factor 3 subunit E OS=Homo sapiens GN=EIF3E PE=1 SV=1 | EIF3E_HUMAN | 52 kDa |  | 0.0001 | 1.45 | 1.65 | 1.75 | 16.91 | 0.664105 | 16.85 | 0.689832 | 17.4 | 0.519237 | 1.4 | 17.42 | 0.520812 | 1.5 | 17.52 | 0.527303 | 1.6 | 17.57 | 0.512658 | 1.7 | 17.63 | 0.514699 | 1.7 | 17.69 | 0.521898 | 1.8 |
| 771 | Phenylalanine--tRNA ligase beta subunit OS=Homo sapiens GN=FARSB PE=1 SV=3 | SYFB_HUMAN | 66 kDa |  | 0.0001 | 1.20 | 1.65 | 1.75 | 17.13 | 0.698754 | 17.16 | 0.582479 | 17.36 | 0.509159 | 1.2 | 17.47 | 0.510443 | 1.2 | 17.83 | 0.521683 | 1.7 | 17.85 | 0.526902 | 1.6 | 17.89 | 0.505167 | 1.7 | 17.99 | 0.518474 | 1.8 |
| 1381 | Lactoylglutathione lyase OS=Homo sapiens GN=GLO1 PE=1 SV=4 | LGUL_HUMAN | 21 kDa |  | 0.024 | 1.15 | 1.65 | 1.75 | 17.99 | 0.634323 | 17.85 | 0.786905 | 18.11 | 0.514962 | 1.1 | 18.1 | 0.534376 | 1.2 | 18.63 | 0.522522 | 1.6 | 18.57 | 0.542422 | 1.7 | 18.69 | 0.535316 | 1.6 | 18.77 | 0.567952 | 1.9 |
| 780 | Bifunctional coenzyme A synthase OS=Homo sapiens GN=COASY PE=1 SV=4 | COASY_HUMAN | 62 kDa |  | 0.00088 | 1.80 | 1.70 | 1.75 | 17.31 | 1.151842 | 16.99 | 0.968057 | 18.03 | 0.583321 | 1.6 | 18.02 | 0.562389 | 2 | 17.82 | 0.529205 | 1.5 | 17.82 | 0.516172 | 1.9 | 17.98 | 0.539423 | 1.6 | 17.87 | 0.574331 | 1.9 |
| 734 | Nuclear transport factor 2 OS=Homo sapiens GN=NUTF2 PE=1 SV=1 | NTF2_HUMAN | 14 kDa |  | 0.0017 | 1.65 | 1.70 | 1.75 | 18.61 | 0.758771 | 18.51 | 1.007582 | 19.21 | 0.541414 | 1.6 | 19.19 | 0.5399 | 1.7 | 19.36 | 0.557048 | 1.7 | 19.22 | 0.530045 | 1.7 | 19.32 | 0.536372 | 1.6 | 19.41 | 0.534916 | 1.9 |
| 176 | V-type proton ATPase catalytic subunit A OS=Homo sapiens GN=ATP6V1A PE=1 SV=2 | VATA_HUMAN | 68 kDa |  | 0.0001 | 1.60 | 1.70 | 1.75 | 17.54 | 1.089462 | 17.52 | 0.930756 | 18.14 | 0.584621 | 1.6 | 18.14 | 0.589061 | 1.6 | 18.29 | 0.579376 | 1.7 | 18.28 | 0.569043 | 1.7 | 18.33 | 0.564749 | 1.7 | 18.32 | 0.636711 | 1.8 |
| 545 | Protein deglycase DJ-1 OS=Homo sapiens GN=PARK7 PE=1 SV=2 | PARK7_HUMAN | 20 kDa |  | 0.0001 | 1.95 | 1.70 | 1.75 | 18.26 | 0.976896 | 18.28 | 0.913867 | 19.16 | 0.635075 | 2 | 19.17 | 0.607385 | 1.9 | 19.09 | 0.578805 | 1.8 | 18.94 | 0.554086 | 1.6 | 19.01 | 0.522318 | 1.7 | 19.04 | 0.570796 | 1.8 |
| 822 | Fumarate hydratase, mitochondrial OS=Homo sapiens GN=FH PE=1 SV=3 | FUMH_HUMAN | 55 kDa |  | 0.0001 | 1.55 | 1.70 | 1.75 | 16.92 | 0.647104 | 16.67 | 0.68307 | 17.39 | 0.509519 | 1.4 | 17.44 | 0.494558 | 1.7 | 17.6 | 0.502748 | 1.6 | 17.59 | 0.512295 | 1.8 | 17.53 | 0.506016 | 1.5 | 17.69 | 0.505712 | 2 |
| 90 | Ubiquitin-like modifier-activating enzyme 1 OS=Homo sapiens GN=UBA1 PE=1 SV=3 | UBA1_HUMAN | 118 kDa | TRUE | 0.0001 | 1.50 | 1.70 | 1.75 | 17.84 | 1.008068 | 17.97 | 0.937082 | 18.45 | 0.584506 | 1.6 | 18.45 | 0.580677 | 1.4 | 18.63 | 0.572367 | 1.8 | 18.57 | 0.55504 | 1.6 | 18.61 | 0.546002 | 1.8 | 18.63 | 0.592599 | 1.7 |
| 147 | T-complex protein 1 subunit eta OS=Homo sapiens GN=CCT7 PE=1 SV=2 | TCPH_HUMAN | 59 kDa |  | 0.0001 | 1.35 | 1.70 | 1.75 | 17.43 | 1.052373 | 17.56 | 0.916643 | 18.03 | 0.601595 | 1.4 | 18.01 | 0.606036 | 1.3 | 18.28 | 0.599332 | 1.8 | 18.15 | 0.561774 | 1.6 | 18.23 | 0.559632 | 1.8 | 18.23 | 0.572314 | 1.7 |
| 156 | Programmed cell death 6-interacting protein OS=Homo sapiens GN=PDCD6IP PE=1 SV=1 | PDC6I_HUMAN | 96 kDa | TRUE | 0.0001 | 1.65 | 1.75 | 1.75 | 17.69 | 0.853887 | 17.61 | 0.846119 | 18.35 | 0.620682 | 1.6 | 18.31 | 0.626833 | 1.7 | 18.51 | 0.549868 | 1.8 | 18.37 | 0.534907 | 1.7 | 18.42 | 0.524628 | 1.7 | 18.42 | 0.548173 | 1.8 |
| 35 | Myeloperoxidase OS=Homo sapiens GN=MPO PE=1 SV=1 | PERM_HUMAN | 84 kDa | TRUE | 0.0001 | 1.60 | 1.75 | 1.75 | 18.19 | 0.988001 | 18.18 | 0.97559 | 18.78 | 0.658435 | 1.6 | 18.81 | 0.649552 | 1.6 | 18.88 | 0.599525 | 1.7 | 18.97 | 0.598206 | 1.8 | 18.9 | 0.565979 | 1.7 | 18.97 | 0.634319 | 1.8 |
| 490 | Protein-L-isoaspartate(D-aspartate) O-methyltransferase OS=Homo sapiens GN=PCMT1 PE=1 SV=4 | PIMT_HUMAN | 25 kDa |  | 0.0001 | 1.60 | 1.75 | 1.75 | 18.11 | 0.993388 | 18.08 | 0.932633 | 18.72 | 0.607631 | 1.6 | 18.75 | 0.61274 | 1.6 | 18.83 | 0.555012 | 1.7 | 18.79 | 0.592616 | 1.8 | 18.83 | 0.546576 | 1.7 | 18.87 | 0.534261 | 1.8 |
| 327 | 4-trimethylaminobutyraldehyde dehydrogenase OS=Homo sapiens GN=ALDH9A1 PE=1 SV=3 | AL9A1_HUMAN | 54 kDa | TRUE | 0.0001 | 1.55 | 1.75 | 1.75 | 17.28 | 0.874919 | 17.37 | 0.869993 | 17.9 | 0.585863 | 1.6 | 17.95 | 0.588102 | 1.5 | 18.14 | 0.599431 | 1.8 | 18.06 | 0.549741 | 1.7 | 18.12 | 0.581618 | 1.8 | 18.07 | 0.55654 | 1.7 |
| 695 | DnaJ homolog subfamily B member 1 OS=Homo sapiens GN=DNAJB1 PE=1 SV=4 | DNJB1_HUMAN | 38 kDa | TRUE | 0.0001 | 1.40 | 1.80 | 1.75 | 17.09 | 0.930041 | 16.95 | 0.77091 | 17.5 | 0.545066 | 1.3 | 17.54 | 0.544112 | 1.5 | 17.78 | 0.553253 | 1.7 | 17.84 | 0.548601 | 1.9 | 17.81 | 0.557497 | 1.7 | 17.78 | 0.572541 | 1.8 |
| 491 | Pleckstrin OS=Homo sapiens GN=PLEK PE=1 SV=3 | PLEK_HUMAN | 40 kDa | TRUE | 0.0001 | 2.05 | 1.80 | 1.75 | 17.05 | 0.937482 | 17.05 | 0.778891 | 18.09 | 0.597602 | 2.1 | 18.04 | 0.583547 | 2 | 17.92 | 0.546697 | 1.8 | 17.84 | 0.537585 | 1.8 | 17.85 | 0.546596 | 1.8 | 17.78 | 0.604639 | 1.7 |
| 155 | T-complex protein 1 subunit alpha OS=Homo sapiens GN=TCP1 PE=1 SV=1 | TCPA_HUMAN | 60 kDa | TRUE | 0.0001 | 1.85 | 1.80 | 1.75 | 17.44 | 0.884371 | 17.37 | 0.909886 | 18.29 | 0.641364 | 1.8 | 18.27 | 0.636622 | 1.9 | 18.26 | 0.569233 | 1.8 | 18.19 | 0.528994 | 1.8 | 18.17 | 0.547846 | 1.7 | 18.13 | 0.610022 | 1.8 |
| 700 | Mitogen-activated protein kinase 14 OS=Homo sapiens GN=MAPK14 PE=1 SV=3 | MK14_HUMAN | 41 kDa |  | 0.0001 | 1.40 | 1.80 | 1.75 | 17.26 | 0.666425 | 17.23 | 0.694541 | 17.74 | 0.533983 | 1.4 | 17.7 | 0.527489 | 1.4 | 18.05 | 0.570573 | 1.8 | 18.03 | 0.530721 | 1.8 | 18.02 | 0.533286 | 1.7 | 18.07 | 0.578943 | 1.8 |
| 1403 | ATP-binding cassette sub-family E member 1 OS=Homo sapiens GN=ABCE1 PE=1 SV=1 | ABCE1_HUMAN | 67 kDa | TRUE | 0.00078 | 1.60 | 1.85 | 1.75 | 16.92 | 0.568677 | 16.8 | 0.582994 | 17.57 | 0.570265 | 1.6 | 17.5 | 0.509686 | 1.6 | 17.73 | 0.518281 | 1.8 | 17.71 | 0.497074 | 1.9 | 17.85 | 0.510222 | 1.9 | 17.6 | 0.781132 | 1.6 |
| 495 | Vasodilator-stimulated phosphoprotein OS=Homo sapiens GN=VASP PE=1 SV=3 | VASP_HUMAN | 40 kDa |  | 0.0001 | 1.85 | 1.95 | 1.75 | 17.56 | 0.902618 | 17.58 | 0.807746 | 18.47 | 0.59356 | 1.8 | 18.51 | 0.618117 | 1.9 | 18.46 | 0.578119 | 1.9 | 18.54 | 0.636718 | 2 | 18.43 | 0.560765 | 1.8 | 18.36 | 0.581978 | 1.7 |
| 249 | Transaldolase OS=Homo sapiens GN=TALDO1 PE=1 SV=2 | TALDO_HUMAN | 38 kDa |  | 0.0001 | 1.60 | 1.95 | 1.75 | 18.24 | 0.890377 | 18.18 | 0.815794 | 18.86 | 0.608564 | 1.6 | 18.85 | 0.615128 | 1.6 | 19.27 | 0.596611 | 2.1 | 19.06 | 0.536801 | 1.8 | 19.01 | 0.56717 | 1.7 | 19.02 | 0.57583 | 1.8 |
| 514 | Carbonic anhydrase 1 OS=Homo sapiens GN=CA1 PE=1 SV=2 | CAH1_HUMAN | 29 kDa |  | 0.0001 | 1.10 | 1.30 | 1.80 | 18.34 | 0.710167 | 18.32 | 0.639645 | 18.47 | 0.556577 | 1.1 | 18.41 | 0.562517 | 1.1 | 18.8 | 0.555755 | 1.4 | 18.63 | 0.574632 | 1.2 | 19.28 | 0.602077 | 1.9 | 19.06 | 0.686631 | 1.7 |
| 566 | Proteasome activator complex subunit 2 OS=Homo sapiens GN=PSME2 PE=1 SV=4 | PSME2_HUMAN | 27 kDa | TRUE | 0.00054 | 1.75 | 1.50 | 1.80 | 17.44 | 0.823102 | 17.48 | 0.9078 | 18.28 | 0.578747 | 1.8 | 18.3 | 0.604611 | 1.7 | 17.97 | 0.584261 | 1.5 | 18 | 0.545437 | 1.5 | 18.17 | 0.546904 | 1.7 | 18.28 | 0.662793 | 1.9 |
| 1225 | Enhancer of rudimentary homolog OS=Homo sapiens GN=ERH PE=1 SV=1 | ERH_HUMAN | 12 kDa |  | 0.0001 | 1.30 | 1.50 | 1.80 | 18.04 | 0.611484 | 18.14 | 0.583427 | 18.49 | 0.497206 | 1.4 | 18.43 | 0.511247 | 1.2 | 18.7 | 0.515203 | 1.6 | 18.58 | 0.503327 | 1.4 | 18.94 | 0.540604 | 1.9 | 18.89 | 0.546947 | 1.7 |
| 1227 | Nucleolar protein 58 OS=Homo sapiens GN=NOP58 PE=1 SV=1 | NOP58_HUMAN | 60 kDa |  | 0.0074 | 1.35 | 1.55 | 1.80 | 17.97 | 0.531725 | 17.89 | 0.515898 | 18.37 | 0.552305 | 1.3 | 18.34 | 0.503556 | 1.4 | 18.48 | 0.539555 | 1.4 | 18.64 | 0.516992 | 1.7 | 18.69 | 0.497262 | 1.7 | 18.8 | 0.604434 | 1.9 |
| 266 | Importin subunit beta-1 OS=Homo sapiens GN=KPNB1 PE=1 SV=2 | IMB1_HUMAN | 97 kDa |  | 0.0001 | 1.90 | 1.55 | 1.80 | 17.71 | 0.998809 | 17.84 | 0.881112 | 18.63 | 0.568828 | 2 | 18.65 | 0.584108 | 1.8 | 18.46 | 0.568923 | 1.6 | 18.45 | 0.512848 | 1.5 | 18.48 | 0.51398 | 1.7 | 18.7 | 0.582597 | 1.9 |
| 352 | 26S protease regulatory subunit 6B OS=Homo sapiens GN=PSMC4 PE=1 SV=2 | PRS6B_HUMAN | 47 kDa | TRUE | 0.0001 | 1.50 | 1.55 | 1.80 | 18.08 | 0.890541 | 18.14 | 0.729218 | 18.65 | 0.532938 | 1.5 | 18.67 | 0.530521 | 1.5 | 18.71 | 0.580885 | 1.5 | 18.8 | 0.601175 | 1.6 | 18.86 | 0.532792 | 1.7 | 18.99 | 0.634232 | 1.9 |
| 846 | Translationally-controlled tumor protein OS=Homo sapiens GN=TPT1 PE=1 SV=1 | TCTP_HUMAN | 20 kDa |  | 0.00027 | 1.45 | 1.60 | 1.80 | 17.48 | 0.696881 | 17.42 | 0.635636 | 17.91 | 0.50522 | 1.4 | 18.03 | 0.554932 | 1.5 | 18.21 | 0.524121 | 1.7 | 18.04 | 0.511998 | 1.5 | 18.34 | 0.553142 | 1.9 | 18.26 | 0.620161 | 1.7 |
| 1530 | Splicing factor 3B subunit 6 OS=Homo sapiens GN=SF3B6 PE=1 SV=1 | SF3B6_HUMAN | 15 kDa | TRUE | 0.0056 | 1.45 | 1.60 | 1.80 | 17.3 | 0.496372 | 17.26 | 0.588552 | 17.77 | 0.494248 | 1.4 | 17.84 | 0.484567 | 1.5 | 18.05 | 0.564727 | 1.7 | 17.86 | 0.498027 | 1.5 | 18.2 | 0.506106 | 1.9 | 18.05 | 0.518016 | 1.7 |
| 1352 | NEDD8-activating enzyme E1 regulatory subunit OS=Homo sapiens GN=NAE1 PE=1 SV=1 | ULA1_HUMAN | 60 kDa |  | 0.0012 | 1.45 | 1.65 | 1.80 | 17.5 | 0.535419 | 17.47 | 0.66505 | 17.98 | 0.505932 | 1.4 | 18.04 | 0.509948 | 1.5 | 18.22 | 0.514523 | 1.6 | 18.2 | 0.496606 | 1.7 | 18.26 | 0.510041 | 1.7 | 18.36 | 0.605083 | 1.9 |
| 163 | Lysozyme C OS=Homo sapiens GN=LYZ PE=1 SV=1 | LYSC_HUMAN | 17 kDa |  | 0.00023 | 1.30 | 1.65 | 1.80 | 18.69 | 0.957042 | 18.61 | 0.871343 | 19 | 0.737034 | 1.3 | 19 | 0.740328 | 1.3 | 19.28 | 0.695945 | 1.6 | 19.28 | 0.672015 | 1.7 | 19.37 | 0.601 | 1.7 | 19.44 | 0.657063 | 1.9 |
| 1027 | 26S proteasome non-ATPase regulatory subunit 8 OS=Homo sapiens GN=PSMD8 PE=1 SV=2 | PSMD8_HUMAN | 40 kDa |  | 0.00042 | 1.60 | 1.70 | 1.80 | 18.09 | 0.724626 | 18.11 | 0.843594 | 18.73 | 0.569057 | 1.6 | 18.73 | 0.538395 | 1.6 | 18.82 | 0.517791 | 1.7 | 18.89 | 0.526078 | 1.7 | 18.84 | 0.516571 | 1.7 | 19.04 | 0.652063 | 1.9 |
| 160 | Dynamin-2 OS=Homo sapiens GN=DNM2 PE=1 SV=2 | DYN2_HUMAN | 98 kDa | TRUE | 0.0001 | 1.55 | 1.70 | 1.80 | 17.74 | 0.860395 | 17.7 | 0.848571 | 18.36 | 0.595882 | 1.5 | 18.34 | 0.568895 | 1.6 | 18.44 | 0.574317 | 1.7 | 18.39 | 0.562156 | 1.7 | 18.45 | 0.548741 | 1.7 | 18.52 | 0.589894 | 1.9 |
| 304 | 26S protease regulatory subunit 8 OS=Homo sapiens GN=PSMC5 PE=1 SV=1 | PRS8_HUMAN | 46 kDa | TRUE | 0.0001 | 1.55 | 1.70 | 1.80 | 17.72 | 1.071643 | 17.79 | 1.057794 | 18.34 | 0.622651 | 1.6 | 18.36 | 0.626049 | 1.5 | 18.48 | 0.562685 | 1.8 | 18.36 | 0.553155 | 1.6 | 18.51 | 0.544817 | 1.9 | 18.44 | 0.563991 | 1.7 |
| 831 | Osteoclast-stimulating factor 1 OS=Homo sapiens GN=OSTF1 PE=1 SV=2 | OSTF1_HUMAN | 24 kDa |  | 0.0029 | 1.75 | 1.75 | 1.80 | 17.04 | 1.0781 | 17.29 | 1.114163 | 17.98 | 0.594123 | 1.9 | 17.97 | 0.591667 | 1.6 | 18.01 | 0.522143 | 1.9 | 17.92 | 0.544458 | 1.6 | 17.91 | 0.544444 | 1.9 | 17.92 | 0.558479 | 1.7 |
| 520 | Laminin subunit alpha-4 OS=Homo sapiens GN=LAMA4 PE=1 SV=4 | LAMA4_HUMAN | 203 kDa |  | 0.0014 | 1.90 | 1.80 | 1.80 | 16.42 | 0.99856 | 16.36 | 0.998559 | 17.26 | 0.529545 | 1.8 | 17.33 | 0.59161 | 2 | 17.24 | 0.511749 | 1.8 | 17.18 | 0.510888 | 1.8 | 17.18 | 0.493182 | 1.7 | 17.29 | 0.514105 | 1.9 |
| 1627 | Uridine 5'-monophosphate synthase OS=Homo sapiens GN=UMPS PE=1 SV=1 | UMPS_HUMAN | 52 kDa |  | 0.027 | 1.70 | 1.90 | 1.80 | 18.08 | 0.783756 | 17.96 | 0.841955 | 18.79 | 0.61466 | 1.6 | 18.82 | 0.59453 | 1.8 | 18.83 | 0.521703 | 1.7 | 19 | 0.572729 | 2.1 | 18.87 | 0.516392 | 1.7 | 18.95 | 0.51831 | 1.9 |
| 82 | Adenylyl cyclase-associated protein 1 OS=Homo sapiens GN=CAP1 PE=1 SV=5 | CAP1_HUMAN | 52 kDa |  | 0.0001 | 2.25 | 2.00 | 1.80 | 17.48 | 1.16473 | 17.54 | 1.106016 | 18.59 | 0.761832 | 2.3 | 18.61 | 0.765084 | 2.2 | 18.48 | 0.620803 | 2 | 18.44 | 0.569795 | 2 | 18.31 | 0.60209 | 1.9 | 18.22 | 0.598119 | 1.7 |
| 1292 | Nuclease-sensitive element-binding protein 1 OS=Homo sapiens GN=YBX1 PE=1 SV=3 | YBOX1_HUMAN | 36 kDa | TRUE | 0.0097 | 1.35 | 1.20 | 1.80 | 17.28 | 1.115682 | 17.42 | 1.225711 | 17.65 | 0.531709 | 1.4 | 17.59 | 0.540727 | 1.3 | 17.58 | 0.491349 | 1.3 | 17.41 | 0.503878 | 1.1 | 18.1 | 0.550357 | 1.8 | 18.22 | 0.676118 | 1.8 |
| 355 | Selenium-binding protein 1 OS=Homo sapiens GN=SELENBP1 PE=1 SV=2 | SBP1_HUMAN | 52 kDa |  | 0.0001 | 1.70 | 1.40 | 1.80 | 18.05 | 0.800408 | 18.09 | 0.674086 | 18.76 | 0.595506 | 1.7 | 18.79 | 0.582355 | 1.7 | 18.53 | 0.549322 | 1.4 | 18.59 | 0.550094 | 1.4 | 18.89 | 0.53219 | 1.8 | 18.97 | 0.551522 | 1.8 |
| 872 | ATP-dependent 6-phosphofructokinase, platelet type OS=Homo sapiens GN=PFKP PE=1 SV=2 | PFKAP_HUMAN | 86 kDa | TRUE | 0.00037 | 1.30 | 1.40 | 1.80 | 17.8 | 0.768845 | 17.72 | 0.695382 | 18.13 | 0.511807 | 1.3 | 18.08 | 0.534691 | 1.3 | 18.25 | 0.598531 | 1.3 | 18.29 | 0.509362 | 1.5 | 18.47 | 0.571976 | 1.6 | 18.72 | 0.638246 | 2 |
| 493 | 40S ribosomal protein S3a OS=Homo sapiens GN=RPS3A PE=1 SV=2 | RS3A_HUMAN | 30 kDa |  | 0.0012 | 1.45 | 1.45 | 1.80 | 17.4 | 0.910507 | 17.5 | 0.88899 | 17.96 | 0.556823 | 1.5 | 17.93 | 0.55355 | 1.4 | 18.07 | 0.602996 | 1.5 | 17.93 | 0.526199 | 1.4 | 18.38 | 0.593936 | 2 | 18.2 | 0.618139 | 1.6 |
| 930 | Protein SET OS=Homo sapiens GN=SET PE=1 SV=3 | SET_HUMAN | 33 kDa |  | 0.017 | 1.75 | 1.50 | 1.80 | 17.48 | 0.935313 | 17.66 | 0.923082 | 18.38 | 0.541073 | 1.9 | 18.35 | 0.553544 | 1.6 | 18.27 | 0.536952 | 1.7 | 18.11 | 0.532897 | 1.3 | 18.44 | 0.531405 | 2 | 18.33 | 0.551278 | 1.6 |
| 564 | Proliferating cell nuclear antigen OS=Homo sapiens GN=PCNA PE=1 SV=1 | PCNA_HUMAN | 29 kDa |  | 0.0001 | 1.35 | 1.50 | 1.80 | 18.01 | 0.760397 | 18 | 0.772432 | 18.37 | 0.510443 | 1.3 | 18.38 | 0.525808 | 1.4 | 18.64 | 0.530051 | 1.5 | 18.56 | 0.520923 | 1.5 | 18.84 | 0.532647 | 1.8 | 18.83 | 0.535604 | 1.8 |
| 802 | Activated RNA polymerase II transcriptional coactivator p15 OS=Homo sapiens GN=SUB1 PE=1 SV=3 | TCP4_HUMAN | 14 kDa |  | 0.0032 | 1.45 | 1.55 | 1.80 | 17.98 | 0.809434 | 18.17 | 0.88074 | 18.53 | 0.523032 | 1.5 | 18.72 | 0.789402 | 1.4 | 18.78 | 0.522096 | 1.8 | 18.6 | 0.524225 | 1.3 | 18.91 | 0.56795 | 2 | 18.86 | 0.54365 | 1.6 |
| 194 | Heterogeneous nuclear ribonucleoprotein K OS=Homo sapiens GN=HNRNPK PE=1 SV=1 | HNRPK_HUMAN | 51 kDa |  | 0.0001 | 1.40 | 1.55 | 1.80 | 17.22 | 1.021987 | 17.19 | 0.97299 | 17.67 | 0.642891 | 1.4 | 17.66 | 0.624592 | 1.4 | 17.8 | 0.579194 | 1.5 | 17.82 | 0.585492 | 1.6 | 18.03 | 0.560633 | 1.8 | 18.02 | 0.599638 | 1.8 |
| 1473 | 60S ribosomal protein L27a OS=Homo sapiens GN=RPL27A PE=1 SV=2 | RL27A_HUMAN | 17 kDa |  | 0.0081 | -1.05 | 1.55 | 1.80 | 17.56 | 0.731386 | 17.65 | 0.593085 | 17.53 | 0.751071 | 1 | 17.53 | 0.596815 | 0.9 | 17.95 | 0.548004 | 1.3 | 18.26 | 1.220987 | 1.8 | 18.32 | 0.640168 | 1.6 | 18.63 | 0.683811 | 2 |
| 789 | Filaggrin OS=Homo sapiens GN=FLG PE=1 SV=3 | FILA_HUMAN | 435 kDa |  | 0.0001 | -1.33 | 1.55 | 1.80 | 15.2 | 0.751013 | 15.26 | 0.596498 | 14.91 | 0.663788 | 0.8 | 14.84 | 0.594059 | 0.7 | 15.78 | 0.649115 | 1.5 | 15.83 | 0.681171 | 1.6 | 16.06 | 0.657536 | 1.8 | 16.06 | 0.746565 | 1.8 |
| 1390 | Replication protein A 32 kDa subunit OS=Homo sapiens GN=RPA2 PE=1 SV=1 | RFA2_HUMAN | 29 kDa |  | 0.019 | 1.40 | 1.60 | 1.80 | 18.28 | 0.705015 | 18.29 | 0.661806 | 18.82 | 0.519028 | 1.4 | 18.77 | 0.510173 | 1.4 | 19.04 | 0.506721 | 1.7 | 18.92 | 0.522051 | 1.5 | 19.08 | 0.510167 | 1.8 | 19.15 | 0.514109 | 1.8 |
| 770 | Signal transducer and activator of transcription 3 OS=Homo sapiens GN=STAT3 PE=1 SV=2 | STAT3_HUMAN | 88 kDa |  | 0.0001 | 1.75 | 1.65 | 1.80 | 17.6 | 0.687782 | 17.63 | 0.66251 | 18.39 | 0.52734 | 1.8 | 18.4 | 0.535494 | 1.7 | 18.31 | 0.511317 | 1.7 | 18.33 | 0.517703 | 1.6 | 18.46 | 0.509009 | 1.8 | 18.48 | 0.591503 | 1.8 |
| 127 | Lamin-B1 OS=Homo sapiens GN=LMNB1 PE=1 SV=2 | LMNB1_HUMAN | 66 kDa | TRUE | 0.0001 | 1.60 | 1.70 | 1.80 | 17.66 | 1.029632 | 17.67 | 0.903801 | 18.31 | 0.577627 | 1.6 | 18.31 | 0.574936 | 1.6 | 18.36 | 0.56287 | 1.7 | 18.32 | 0.579285 | 1.7 | 18.47 | 0.556332 | 1.8 | 18.48 | 0.559652 | 1.8 |
| 439 | Probable ATP-dependent RNA helicase DDX17 OS=Homo sapiens GN=DDX17 PE=1 SV=2 | DDX17_HUMAN | 80 kDa | TRUE | 0.0001 | 1.65 | 1.70 | 1.80 | 17.73 | 1.085458 | 17.67 | 1.169007 | 18.39 | 0.528466 | 1.6 | 18.4 | 0.529339 | 1.7 | 18.34 | 0.521779 | 1.6 | 18.42 | 0.538205 | 1.8 | 18.56 | 0.528753 | 1.8 | 18.51 | 0.527084 | 1.8 |
| 1711 | Caspase recruitment domain-containing protein 8 OS=Homo sapiens GN=CARD8 PE=1 SV=1 | CARD8_HUMAN | 49 kDa |  | 0.0056 | 1.50 | 1.70 | 1.80 | 17.13 | 0.804638 | 17.21 | 0.793884 | 17.71 | 0.502869 | 1.5 | 17.79 | 0.501213 | 1.5 | 17.99 | 0.496572 | 1.8 | 17.91 | 0.514628 | 1.6 | 18.01 | 0.499509 | 1.8 | 18.02 | 0.49157 | 1.8 |
| 165 | X-ray repair cross-complementing protein 5 OS=Homo sapiens GN=XRCC5 PE=1 SV=3 | XRCC5_HUMAN | 83 kDa | TRUE | 0.0001 | 1.80 | 1.75 | 1.80 | 17.25 | 0.802515 | 17.26 | 0.777487 | 18.05 | 0.631067 | 1.8 | 18.11 | 0.607727 | 1.8 | 18.02 | 0.568438 | 1.7 | 18.04 | 0.580889 | 1.8 | 18.1 | 0.543525 | 1.8 | 18.07 | 0.57613 | 1.8 |
| 559 | Beta-hexosaminidase subunit beta OS=Homo sapiens GN=HEXB PE=1 SV=3 | HEXB_HUMAN | 63 kDa |  | 0.001 | 1.75 | 1.75 | 1.80 | 17.56 | 0.831089 | 17.53 | 0.896048 | 18.37 | 0.519265 | 1.7 | 18.41 | 0.533724 | 1.8 | 18.42 | 0.516028 | 1.8 | 18.31 | 0.51442 | 1.7 | 18.42 | 0.534346 | 1.8 | 18.34 | 0.579391 | 1.8 |
| 322 | Proteasome subunit alpha type-3 OS=Homo sapiens GN=PSMA3 PE=1 SV=2 | PSA3_HUMAN | 28 kDa | TRUE | 0.001 | 2.10 | 1.80 | 1.80 | 17.76 | 1.400543 | 17.79 | 1.18315 | 18.69 | 0.667825 | 2.2 | 18.64 | 0.685585 | 2 | 18.68 | 0.547244 | 1.9 | 18.55 | 0.548561 | 1.7 | 18.59 | 0.533884 | 1.8 | 18.59 | 0.543853 | 1.8 |
| 100 | T-complex protein 1 subunit epsilon OS=Homo sapiens GN=CCT5 PE=1 SV=1 | TCPE_HUMAN | 60 kDa |  | 0.0001 | 1.75 | 1.80 | 1.80 | 17.93 | 0.913681 | 17.98 | 0.853932 | 18.75 | 0.609088 | 1.8 | 18.72 | 0.617393 | 1.7 | 18.77 | 0.579227 | 1.9 | 18.69 | 0.576439 | 1.7 | 18.75 | 0.569723 | 1.8 | 18.78 | 0.555573 | 1.8 |
| 488 | Neutrophil cytosol factor 4 OS=Homo sapiens GN=NCF4 PE=1 SV=2 | NCF4_HUMAN | 39 kDa | TRUE | 0.0001 | 1.70 | 1.85 | 1.80 | 18.25 | 0.894978 | 18.38 | 0.814615 | 19.01 | 0.589712 | 1.7 | 19.06 | 0.632561 | 1.7 | 19.12 | 0.581833 | 1.9 | 19.19 | 0.553733 | 1.8 | 19.02 | 0.542165 | 1.8 | 19.16 | 0.605151 | 1.8 |
| 1006 | Heterogeneous nuclear ribonucleoprotein R OS=Homo sapiens GN=HNRNPR PE=1 SV=1 | HNRPR_HUMAN | 71 kDa | TRUE | 0.0001 | 1.70 | 1.85 | 1.80 | 15.77 | 0.729137 | 15.93 | 0.874719 | 16.52 | 0.505299 | 1.7 | 16.64 | 0.495034 | 1.7 | 16.65 | 0.496254 | 1.8 | 16.81 | 0.498885 | 1.9 | 16.67 | 0.526113 | 1.8 | 16.73 | 0.505022 | 1.8 |
| 247 | Myosin light polypeptide 6 OS=Homo sapiens GN=MYL6 PE=1 SV=2 | MYL6_HUMAN | 17 kDa | TRUE | 0.0001 | 1.55 | 1.90 | 1.80 | 17.75 | 1.160335 | 17.71 | 1.057607 | 18.31 | 0.566019 | 1.5 | 18.35 | 0.553168 | 1.6 | 18.61 | 0.57787 | 1.9 | 18.54 | 0.554558 | 1.9 | 18.46 | 0.569776 | 1.8 | 18.43 | 0.576021 | 1.8 |
| 1558 | Haloacid dehalogenase-like hydrolase domain-containing protein 2 OS=Homo sapiens GN=HDHD2 PE=1 SV=1 | HDHD2_HUMAN | 29 kDa |  | 0.026 | 1.80 | 2.00 | 1.80 | 17.57 | 0.629513 | 17.33 | 0.797825 | 18.38 | 0.490122 | 1.8 | 18.17 | 0.484792 | 1.8 | 18.48 | 0.486216 | 1.9 | 18.38 | 0.509707 | 2.1 | 18.37 | 0.490237 | 1.8 | 18.21 | 0.500287 | 1.8 |
| 1575 | Gamma-enolase OS=Homo sapiens GN=ENO2 PE=1 SV=3 | ENOG_HUMAN | 47 kDa | TRUE | 0.0018 | 1.35 | 2.05 | 1.80 | 16.77 | 0.68607 | 16.37 | 0.625388 | 17.03 | 0.49201 | 1.2 | 16.99 | 0.492701 | 1.5 | 17.64 | 0.503105 | 1.8 | 17.57 | 0.516684 | 2.3 | 17.4 | 0.519041 | 1.6 | 17.35 | 0.496087 | 2 |
| 1527 | Probable aminopeptidase NPEPL1 OS=Homo sapiens GN=NPEPL1 PE=1 SV=3 | PEPL1_HUMAN | 56 kDa |  | 0.006 | 1.85 | 2.10 | 1.80 | 16.38 | 0.610284 | 16.46 | 0.531189 | 17.32 | 0.581071 | 1.9 | 17.32 | 0.50988 | 1.8 | 17.39 | 0.506125 | 2 | 17.49 | 0.755355 | 2.2 | 17.36 | 0.489292 | 2 | 17.17 | 0.59766 | 1.6 |
| 681 | 60S ribosomal protein L18 OS=Homo sapiens GN=RPL18 PE=1 SV=2 | RL18_HUMAN | 22 kDa |  | 0.021 | 1.00 | 1.20 | 1.85 | 17.59 | 1.001801 | 17.53 | 1.068616 | 17.65 | 0.605711 | 1 | 17.68 | 0.742401 | 1 | 17.72 | 0.551935 | 1.2 | 17.67 | 0.573249 | 1.2 | 18.19 | 0.625241 | 1.7 | 18.29 | 0.645312 | 2 |
| 689 | Triosephosphate isomerase OS=Homo sapiens GN=TPI1 PE=1 SV=3 | TPIS_HUMAN | 31 kDa |  | 0.0001 | -1.05 | 1.35 | 1.85 | 18.67 | 0.826637 | 18.81 | 0.849732 | 18.66 | 0.587503 | 1 | 18.63 | 0.553872 | 0.9 | 19.2 | 0.59578 | 1.5 | 18.98 | 0.555958 | 1.2 | 19.57 | 0.637685 | 2 | 19.55 | 0.671998 | 1.7 |
| 1279 | 40S ribosomal protein S17 OS=Homo sapiens GN=RPS17 PE=1 SV=2 | RS17_HUMAN | 16 kDa | TRUE | 0.00015 | 1.05 | 1.40 | 1.85 | 18.63 | 0.640062 | 18.82 | 0.591711 | 18.7 | 0.759637 | 1.1 | 18.88 | 0.517354 | 1 | 19.13 | 0.52168 | 1.4 | 19.28 | 0.530284 | 1.4 | 19.38 | 0.518622 | 1.7 | 19.78 | 0.535382 | 2 |
| 833 | 60S acidic ribosomal protein P2 OS=Homo sapiens GN=RPLP2 PE=1 SV=1 | RLA2_HUMAN | 12 kDa |  | 0.00019 | 1.20 | 1.45 | 1.85 | 16.96 | 0.887869 | 17.04 | 0.751568 | 17.28 | 0.5604 | 1.2 | 17.3 | 0.550548 | 1.2 | 17.49 | 0.538637 | 1.5 | 17.42 | 0.512392 | 1.4 | 17.72 | 0.540376 | 1.8 | 17.88 | 0.594123 | 1.9 |
| 1017 | Bifunctional 3'-phosphoadenosine 5'-phosphosulfate synthase 1 OS=Homo sapiens GN=PAPSS1 PE=1 SV=2 | PAPS1_HUMAN | 71 kDa | TRUE | 0.00078 | 1.35 | 1.50 | 1.85 | 17.75 | 0.706057 | 17.73 | 0.799656 | 18.08 | 0.510997 | 1.3 | 18.1 | 0.540796 | 1.4 | 18.31 | 0.509001 | 1.5 | 18.34 | 0.503855 | 1.5 | 18.62 | 0.522857 | 1.8 | 18.62 | 0.51896 | 1.9 |
| 970 | 40S ribosomal protein S25 OS=Homo sapiens GN=RPS25 PE=1 SV=1 | RS25_HUMAN | 14 kDa |  | 0.00011 | 1.20 | 1.50 | 1.85 | 17.24 | 0.583726 | 17.23 | 0.544257 | 17.52 | 0.510033 | 1.2 | 17.55 | 0.517417 | 1.2 | 17.73 | 0.528101 | 1.4 | 17.81 | 0.808416 | 1.6 | 18.09 | 0.521139 | 1.8 | 18.17 | 0.562317 | 1.9 |
| 1514 | Protein NOXP20 OS=Homo sapiens GN=FAM114A1 PE=1 SV=2 | NXP20_HUMAN | 61 kDa |  | 0.0034 | 1.50 | 1.55 | 1.85 | 15.91 | 0.715352 | 15.57 | 0.727213 | 16.29 | 0.497752 | 1.3 | 16.35 | 0.516705 | 1.7 | 16.38 | 0.496314 | 1.4 | 16.39 | 0.538919 | 1.7 | 16.61 | 0.508916 | 1.6 | 16.63 | 0.49546 | 2.1 |
| 909 | Lupus La protein OS=Homo sapiens GN=SSB PE=1 SV=2 | LA_HUMAN | 47 kDa | TRUE | 0.0001 | 1.30 | 1.55 | 1.85 | 17.21 | 0.657468 | 17.31 | 0.727834 | 17.62 | 0.510222 | 1.3 | 17.62 | 0.507967 | 1.3 | 17.95 | 0.5125 | 1.7 | 17.77 | 0.535517 | 1.4 | 18.12 | 0.51303 | 1.9 | 18.15 | 0.525277 | 1.8 |
| 499 | Heterogeneous nuclear ribonucleoprotein U-like protein 2 OS=Homo sapiens GN=HNRNPUL2 PE=1 SV=1 | HNRL2_HUMAN | 85 kDa | TRUE | 0.0001 | 1.40 | 1.55 | 1.85 | 18.09 | 0.660982 | 18.15 | 0.713966 | 18.56 | 0.52189 | 1.4 | 18.61 | 0.519569 | 1.4 | 18.73 | 0.528142 | 1.6 | 18.67 | 0.527397 | 1.5 | 18.99 | 0.524599 | 1.9 | 19.02 | 0.586301 | 1.8 |
| 1416 | Protein FAM98B OS=Homo sapiens GN=FAM98B PE=1 SV=1 | FA98B_HUMAN | 37 kDa |  | 0.0022 | 1.40 | 1.55 | 1.85 | 16.29 | 0.743009 | 16.5 | 0.701523 | 16.98 | 0.554006 | 1.5 | 16.97 | 0.552103 | 1.3 | 16.98 | 0.487118 | 1.6 | 16.97 | 0.551379 | 1.5 | 17.28 | 0.573423 | 2.1 | 17.1 | 0.587888 | 1.6 |
| 588 | Coronin-1B OS=Homo sapiens GN=CORO1B PE=1 SV=1 | COR1B_HUMAN | 54 kDa | TRUE | 0.0001 | 1.30 | 1.55 | 1.85 | 17.53 | 0.723005 | 17.57 | 0.741793 | 17.92 | 0.564046 | 1.3 | 17.94 | 0.552124 | 1.3 | 18.09 | 0.624468 | 1.5 | 18.17 | 0.57663 | 1.6 | 18.38 | 0.646767 | 1.8 | 18.46 | 0.715177 | 1.9 |
| 1330 | Peptidyl-prolyl cis-trans isomerase FKBP10 OS=Homo sapiens GN=FKBP10 PE=1 SV=1 | FKB10_HUMAN | 64 kDa |  | 0.0001 | 1.30 | 1.55 | 1.85 | 18.47 | 0.571317 | 18.48 | 0.616858 | 18.89 | 0.518187 | 1.3 | 18.81 | 0.513962 | 1.3 | 19.13 | 0.568612 | 1.6 | 19.03 | 0.525396 | 1.5 | 19.41 | 0.580004 | 1.9 | 19.32 | 0.545872 | 1.8 |
| 726 | GTPase IMAP family member 4 OS=Homo sapiens GN=GIMAP4 PE=1 SV=1 | GIMA4_HUMAN | 38 kDa |  | 0.00011 | 1.25 | 1.55 | 1.85 | 17.18 | 0.806363 | 17.16 | 0.782429 | 17.46 | 0.573773 | 1.2 | 17.52 | 0.59207 | 1.3 | 17.73 | 0.632753 | 1.5 | 17.79 | 0.515677 | 1.6 | 17.92 | 0.573466 | 1.8 | 18.13 | 0.629854 | 1.9 |
| 470 | Proteasome subunit alpha type-4 OS=Homo sapiens GN=PSMA4 PE=1 SV=1 | PSA4_HUMAN | 29 kDa |  | 0.0037 | 1.70 | 1.60 | 1.85 | 16.99 | 1.139683 | 17.28 | 1.040901 | 17.85 | 0.561708 | 1.8 | 17.89 | 0.583752 | 1.6 | 17.79 | 0.581896 | 1.7 | 17.74 | 0.549325 | 1.5 | 17.87 | 0.551818 | 1.9 | 17.92 | 0.683497 | 1.8 |
| 484 | Heterogeneous nuclear ribonucleoprotein F OS=Homo sapiens GN=HNRNPF PE=1 SV=3 | HNRPF_HUMAN | 46 kDa | TRUE | 0.0001 | 1.30 | 1.60 | 1.85 | 18.18 | 0.920301 | 18.21 | 0.891312 | 18.47 | 0.551589 | 1.2 | 18.6 | 0.525717 | 1.4 | 18.9 | 0.529402 | 1.7 | 18.77 | 0.503047 | 1.5 | 19.13 | 0.564461 | 1.9 | 19.03 | 0.622986 | 1.8 |
| 579 | Myeloblastin OS=Homo sapiens GN=PRTN3 PE=1 SV=3 | PRTN3_HUMAN | 28 kDa |  | 0.00056 | 2.20 | 1.65 | 1.85 | 17.64 | 0.87058 | 17.84 | 0.896344 | 18.78 | 0.752638 | 2.4 | 18.78 | 0.751706 | 2 | 18.37 | 0.570904 | 1.7 | 18.56 | 0.538946 | 1.6 | 18.56 | 0.562519 | 1.9 | 18.7 | 0.678295 | 1.8 |
| 744 | Vacuolar protein sorting-associated protein 29 OS=Homo sapiens GN=VPS29 PE=1 SV=1 | VPS29_HUMAN | 21 kDa |  | 0.0001 | 1.75 | 1.65 | 1.85 | 18.84 | 0.720706 | 18.91 | 0.762846 | 19.69 | 0.554511 | 1.8 | 19.7 | 0.547785 | 1.7 | 19.58 | 0.541698 | 1.7 | 19.57 | 0.522407 | 1.6 | 19.72 | 0.533124 | 1.9 | 19.73 | 0.545782 | 1.8 |
| 494 | Protein transport protein Sec23A OS=Homo sapiens GN=SEC23A PE=1 SV=2 | SC23A_HUMAN | 86 kDa | TRUE | 0.0008 | 1.55 | 1.65 | 1.85 | 17.67 | 0.908469 | 17.78 | 0.9107 | 18.27 | 0.591015 | 1.5 | 18.38 | 0.576783 | 1.6 | 18.42 | 0.541396 | 1.7 | 18.43 | 0.508694 | 1.6 | 18.51 | 0.532032 | 1.8 | 18.65 | 0.603165 | 1.9 |
| 526 | RuvB-like 1 OS=Homo sapiens GN=RUVBL1 PE=1 SV=1 | RUVB1_HUMAN | 50 kDa |  | 0.0001 | 1.50 | 1.65 | 1.85 | 18.02 | 0.755731 | 18.07 | 0.75941 | 18.64 | 0.554561 | 1.5 | 18.63 | 0.557237 | 1.5 | 18.77 | 0.563503 | 1.7 | 18.71 | 0.586409 | 1.6 | 18.85 | 0.602268 | 1.8 | 18.91 | 0.615227 | 1.9 |
| 290 | UV excision repair protein RAD23 homolog B OS=Homo sapiens GN=RAD23B PE=1 SV=1 | RD23B_HUMAN | 43 kDa | TRUE | 0.0001 | 1.45 | 1.65 | 1.85 | 17.9 | 0.800051 | 17.9 | 0.717399 | 18.42 | 0.522845 | 1.4 | 18.48 | 0.549031 | 1.5 | 18.59 | 0.562228 | 1.7 | 18.59 | 0.543488 | 1.6 | 18.69 | 0.554323 | 1.8 | 18.77 | 0.600955 | 1.9 |
| 1704 | RILP-like protein 2 OS=Homo sapiens GN=RILPL2 PE=1 SV=1 | RIPL2_HUMAN | 24 kDa | TRUE | 0.0038 | 1.35 | 1.65 | 1.85 | 18.03 | 0.663333 | 18.03 | 0.721184 | 18.4 | 0.522679 | 1.3 | 18.5 | 0.543043 | 1.4 | 18.75 | 0.561368 | 1.6 | 18.77 | 0.5289 | 1.7 | 18.9 | 0.5587 | 1.8 | 19.03 | 0.608689 | 1.9 |
| 148 | Beta-actin-like protein 2 OS=Homo sapiens GN=ACTBL2 PE=1 SV=2 | ACTBL_HUMAN | 42 kDa | TRUE | 0.011 | 1.00 | 1.65 | 1.85 | 15.74 | 0.661365 | 15.82 | 0.687343 | 15.74 | 0.758833 | 1 | 15.77 | 0.748393 | 1 | 16.47 | 0.892689 | 1.6 | 16.54 | 0.652059 | 1.7 | 16.39 | 0.595431 | 1.6 | 16.8 | 0.780243 | 2.1 |
| 1270 | Paxillin OS=Homo sapiens GN=PXN PE=1 SV=3 | PAXI_HUMAN | 65 kDa |  | 0.0019 | 1.80 | 1.70 | 1.85 | 16.43 | 0.709322 | 16.47 | 0.659817 | 17.27 | 0.590786 | 1.8 | 17.34 | 0.573397 | 1.8 | 17.23 | 0.5225 | 1.8 | 17.14 | 0.507284 | 1.6 | 17.33 | 0.491861 | 1.9 | 17.26 | 0.52539 | 1.8 |
| 532 | Cathepsin D OS=Homo sapiens GN=CTSD PE=1 SV=1 | CATD_HUMAN | 45 kDa | TRUE | 0.0001 | 1.75 | 1.70 | 1.85 | 18.41 | 0.878779 | 18.43 | 0.775875 | 19.25 | 0.648388 | 1.9 | 19.14 | 0.564953 | 1.6 | 19.21 | 0.620953 | 1.8 | 19.06 | 0.529393 | 1.6 | 19.26 | 0.58092 | 1.8 | 19.32 | 0.593412 | 1.9 |
| 106 | Delta-aminolevulinic acid dehydratase OS=Homo sapiens GN=ALAD PE=1 SV=1 | HEM2_HUMAN | 36 kDa |  | 0.0001 | 1.65 | 1.70 | 1.85 | 17.61 | 0.915137 | 17.64 | 0.772612 | 18.33 | 0.621236 | 1.7 | 18.33 | 0.619498 | 1.6 | 18.24 | 0.621551 | 1.6 | 18.45 | 0.603917 | 1.8 | 18.39 | 0.555511 | 1.7 | 18.61 | 0.647109 | 2 |
| 174 | 26S protease regulatory subunit 4 OS=Homo sapiens GN=PSMC1 PE=1 SV=1 | PRS4_HUMAN | 49 kDa | TRUE | 0.0001 | 1.65 | 1.70 | 1.85 | 17.77 | 0.826812 | 17.74 | 0.865005 | 18.42 | 0.561377 | 1.6 | 18.45 | 0.553782 | 1.7 | 18.58 | 0.541537 | 1.8 | 18.41 | 0.537621 | 1.6 | 18.61 | 0.520841 | 1.8 | 18.62 | 0.558105 | 1.9 |
| 581 | Heterogeneous nuclear ribonucleoprotein A1 OS=Homo sapiens GN=HNRNPA1 PE=1 SV=5 | ROA1_HUMAN | 39 kDa | TRUE | 0.00069 | 1.25 | 1.70 | 1.85 | 16.82 | 0.724886 | 16.64 | 0.793516 | 17.09 | 0.655011 | 1.2 | 17.03 | 0.614465 | 1.3 | 17.49 | 0.536603 | 1.6 | 17.5 | 0.534984 | 1.8 | 17.74 | 0.586581 | 1.9 | 17.54 | 0.795537 | 1.8 |
| 787 | Eukaryotic translation initiation factor 3 subunit H OS=Homo sapiens GN=EIF3H PE=1 SV=1 | EIF3H_HUMAN | 40 kDa |  | 0.0001 | 2.05 | 1.75 | 1.85 | 17.3 | 0.802763 | 17.28 | 0.755211 | 18.27 | 0.586896 | 2 | 18.3 | 0.595445 | 2.1 | 18.01 | 0.513724 | 1.7 | 18.09 | 0.559255 | 1.8 | 18.17 | 0.525297 | 1.8 | 18.18 | 0.546114 | 1.9 |
| 453 | 26S proteasome non-ATPase regulatory subunit 5 OS=Homo sapiens GN=PSMD5 PE=1 SV=3 | PSMD5_HUMAN | 56 kDa |  | 0.0001 | 1.85 | 1.75 | 1.85 | 17.53 | 0.800631 | 17.54 | 0.797508 | 18.38 | 0.572735 | 1.8 | 18.41 | 0.558292 | 1.9 | 18.29 | 0.501955 | 1.7 | 18.36 | 0.564024 | 1.8 | 18.42 | 0.531506 | 1.8 | 18.49 | 0.555043 | 1.9 |
| 693 | Copper chaperone for superoxide dismutase OS=Homo sapiens GN=CCS PE=1 SV=1 | CCS_HUMAN | 29 kDa |  | 0.001 | 1.70 | 1.75 | 1.85 | 17.74 | 0.880646 | 17.85 | 0.984998 | 18.54 | 0.522735 | 1.7 | 18.57 | 0.513481 | 1.7 | 18.62 | 0.551593 | 1.8 | 18.58 | 0.515353 | 1.7 | 18.64 | 0.513911 | 1.9 | 18.69 | 0.574786 | 1.8 |
| 366 | Chloride intracellular channel protein 1 OS=Homo sapiens GN=CLIC1 PE=1 SV=4 | CLIC1_HUMAN | 27 kDa | TRUE | 0.0001 | 1.95 | 1.80 | 1.85 | 18.02 | 1.03326 | 18.1 | 1.009545 | 18.95 | 0.659789 | 2 | 19.01 | 0.65811 | 1.9 | 18.91 | 0.572038 | 1.9 | 18.82 | 0.563683 | 1.7 | 18.92 | 0.549963 | 1.9 | 18.9 | 0.58338 | 1.8 |
| 274 | Proteasome activator complex subunit 1 OS=Homo sapiens GN=PSME1 PE=1 SV=1 | PSME1_HUMAN | 29 kDa | TRUE | 0.0001 | 1.90 | 1.80 | 1.85 | 18.13 | 0.928559 | 18.17 | 0.95974 | 19 | 0.587449 | 1.9 | 19.01 | 0.580025 | 1.9 | 19.06 | 0.550312 | 1.9 | 18.93 | 0.533542 | 1.7 | 19.02 | 0.545536 | 1.9 | 18.99 | 0.548827 | 1.8 |
| 1356 | U6 snRNA-associated Sm-like protein LSm8 OS=Homo sapiens GN=LSM8 PE=1 SV=3 | LSM8_HUMAN | 10 kDa |  | 0.017 | 1.80 | 1.80 | 1.85 | 17.24 | 0.824683 | 17.1 | 0.853166 | 18.03 | 0.513396 | 1.7 | 18.05 | 0.505184 | 1.9 | 17.96 | 0.543108 | 1.7 | 18.06 | 0.526228 | 1.9 | 18.09 | 0.515636 | 1.8 | 18.03 | 0.514212 | 1.9 |
| 121 | T-complex protein 1 subunit beta OS=Homo sapiens GN=CCT2 PE=1 SV=4 | TCPB_HUMAN | 57 kDa |  | 0.0001 | 1.75 | 1.80 | 1.85 | 17.94 | 0.896942 | 17.96 | 0.875525 | 18.71 | 0.607877 | 1.8 | 18.7 | 0.591099 | 1.7 | 18.82 | 0.585439 | 1.9 | 18.68 | 0.542957 | 1.7 | 18.77 | 0.542674 | 1.8 | 18.82 | 0.586267 | 1.9 |
| 102 | T-complex protein 1 subunit theta OS=Homo sapiens GN=CCT8 PE=1 SV=4 | TCPQ_HUMAN | 60 kDa |  | 0.0001 | 1.80 | 1.85 | 1.85 | 17.74 | 1.109244 | 17.88 | 1.111822 | 18.59 | 0.569104 | 1.9 | 18.62 | 0.559465 | 1.7 | 18.64 | 0.570375 | 1.9 | 18.62 | 0.555878 | 1.8 | 18.65 | 0.543556 | 1.9 | 18.65 | 0.596337 | 1.8 |
| 701 | Dual specificity mitogen-activated protein kinase kinase 1 OS=Homo sapiens GN=MAP2K1 PE=1 SV=2 | MP2K1_HUMAN | 43 kDa | TRUE | 0.02 | 1.60 | 1.85 | 1.85 | 18.06 | 0.788704 | 18 | 0.672485 | 18.63 | 0.511294 | 1.5 | 18.76 | 0.518253 | 1.7 | 19.02 | 0.524211 | 1.9 | 18.84 | 0.518539 | 1.8 | 19.04 | 0.580484 | 2.1 | 18.68 | 0.578052 | 1.6 |
| 483 | Growth factor receptor-bound protein 2 OS=Homo sapiens GN=GRB2 PE=1 SV=1 | GRB2_HUMAN | 25 kDa |  | 0.0001 | 1.95 | 1.90 | 1.85 | 17.71 | 0.836973 | 17.56 | 0.846041 | 18.57 | 0.731183 | 1.9 | 18.53 | 0.657709 | 2 | 18.55 | 0.625917 | 1.9 | 18.54 | 0.570163 | 1.9 | 18.55 | 0.56121 | 1.8 | 18.5 | 0.632789 | 1.9 |
| 236 | Heat shock 70 kDa protein 1A/1B OS=Homo sapiens GN=HSPA1A PE=1 SV=5 | HSP71_HUMAN | ? | TRUE | 0.0001 | 1.75 | 1.90 | 1.85 | 17.28 | 1.039943 | 17.42 | 1.04824 | 18.15 | 0.557852 | 1.8 | 18.18 | 0.559689 | 1.7 | 18.31 | 0.572803 | 2.1 | 18.2 | 0.512006 | 1.7 | 18.21 | 0.522618 | 1.9 | 18.22 | 0.56897 | 1.8 |
| 1421 | Immunity-related GTPase family Q protein OS=Homo sapiens GN=IRGQ PE=1 SV=1 | IRGQ_HUMAN | 63 kDa |  | 0.011 | 1.50 | 1.95 | 1.85 | 16.82 | 0.787909 | 16.87 | 0.703757 | 17.38 | 0.653436 | 1.6 | 17.31 | 0.642976 | 1.4 | 17.73 | 0.540887 | 1.9 | 17.86 | 0.513601 | 2 | 17.71 | 0.501377 | 1.8 | 17.77 | 0.501877 | 1.9 |
| 182 | Hexokinase-3 OS=Homo sapiens GN=HK3 PE=1 SV=2 | HXK3_HUMAN | 99 kDa | TRUE | 0.0001 | 2.40 | 2.05 | 1.85 | 17.93 | 1.018961 | 17.92 | 0.967242 | 19.09 | 0.60937 | 2.4 | 19.13 | 0.62274 | 2.4 | 18.87 | 0.560977 | 2 | 18.9 | 0.557953 | 2.1 | 18.73 | 0.538124 | 1.8 | 18.8 | 0.5641 | 1.9 |
| 438 | Cell division control protein 42 homolog OS=Homo sapiens GN=CDC42 PE=1 SV=2 | CDC42_HUMAN | 21 kDa | TRUE | 0.0001 | 2.05 | 2.05 | 1.85 | 18.04 | 1.484655 | 17.9 | 1.210639 | 18.93 | 0.552297 | 1.9 | 18.99 | 0.540583 | 2.2 | 18.88 | 0.559409 | 1.9 | 18.86 | 0.597571 | 2.2 | 18.72 | 0.535002 | 1.7 | 18.83 | 0.539626 | 2 |
| 130 | Transketolase OS=Homo sapiens GN=TKT PE=1 SV=3 | TKT_HUMAN | 68 kDa |  | 0.0001 | 1.85 | 2.15 | 1.85 | 18.29 | 1.034241 | 18.21 | 0.983468 | 19 | 0.701193 | 1.8 | 19.03 | 0.681943 | 1.9 | 19.39 | 0.627895 | 2.2 | 19.22 | 0.550648 | 2.1 | 19.13 | 0.559094 | 1.8 | 19.1 | 0.582496 | 1.9 |
| 79 | L-lactate dehydrogenase A chain OS=Homo sapiens GN=LDHA PE=1 SV=2 | LDHA_HUMAN | 37 kDa | TRUE | 0.0001 | 2.20 | 2.45 | 1.85 | 17.61 | 1.043516 | 17.66 | 1.031132 | 18.71 | 0.73025 | 2.2 | 18.76 | 0.73813 | 2.2 | 18.84 | 0.776114 | 2.6 | 18.79 | 0.604474 | 2.3 | 18.43 | 0.626241 | 1.9 | 18.46 | 0.655167 | 1.8 |
| 926 | 40S ribosomal protein S13 OS=Homo sapiens GN=RPS13 PE=1 SV=2 | RS13_HUMAN | 17 kDa |  | 0.0018 | 1.20 | 1.35 | 1.90 | 17.7 | 0.883244 | 17.83 | 0.811575 | 18.02 | 0.617 | 1.2 | 18.09 | 0.506945 | 1.2 | 18.16 | 0.514378 | 1.4 | 18.16 | 0.496521 | 1.3 | 18.61 | 0.575875 | 1.9 | 18.69 | 0.6006 | 1.9 |
| 983 | Tripeptidyl-peptidase 1 OS=Homo sapiens GN=TPP1 PE=1 SV=2 | TPP1_HUMAN | 61 kDa |  | 0.0001 | 1.40 | 1.50 | 1.90 | 18.17 | 0.631677 | 18.17 | 0.732445 | 18.68 | 0.563144 | 1.4 | 18.68 | 0.528365 | 1.4 | 18.72 | 0.57516 | 1.5 | 18.82 | 0.518764 | 1.5 | 19 | 0.570792 | 1.8 | 19.16 | 0.588491 | 2 |
| 443 | Polypyrimidine tract-binding protein 1 OS=Homo sapiens GN=PTBP1 PE=1 SV=1 | PTBP1_HUMAN | 57 kDa | TRUE | 0.0001 | 1.80 | 1.55 | 1.90 | 17.91 | 0.909291 | 17.8 | 0.804437 | 18.62 | 0.594293 | 1.7 | 18.61 | 0.594943 | 1.9 | 18.45 | 0.616612 | 1.4 | 18.57 | 0.53443 | 1.7 | 18.74 | 0.570255 | 1.8 | 18.77 | 0.626906 | 2 |
| 48 | Heat shock protein HSP 90-beta OS=Homo sapiens GN=HSP90AB1 PE=1 SV=4 | HS90B_HUMAN | 83 kDa | TRUE | 0.0001 | 1.50 | 1.55 | 1.90 | 17.8 | 0.84264 | 17.84 | 0.893117 | 18.31 | 0.760385 | 1.5 | 18.37 | 0.780434 | 1.5 | 18.4 | 0.621199 | 1.5 | 18.41 | 0.577247 | 1.6 | 18.66 | 0.598984 | 1.9 | 18.7 | 0.645015 | 1.9 |
| 1288 | Tubulin beta-2A chain OS=Homo sapiens GN=TUBB2A PE=1 SV=1 | TBB2A_HUMAN | 50 kDa | TRUE | 0.0022 | 1.35 | 1.55 | 1.90 | 17.36 | 1.072826 | 17.47 | 0.931314 | 17.81 | 0.509073 | 1.4 | 17.83 | 0.519055 | 1.3 | 18.04 | 0.516214 | 1.6 | 18.11 | 0.571783 | 1.5 | 18.21 | 0.546248 | 1.9 | 18.36 | 0.572364 | 1.9 |
| 404 | Cytosol aminopeptidase OS=Homo sapiens GN=LAP3 PE=1 SV=3 | AMPL_HUMAN | 56 kDa |  | 0.0001 | 1.00 | 1.55 | 1.90 | 17.96 | 0.782276 | 18 | 0.709207 | 18 | 0.558025 | 1 | 18.01 | 0.546438 | 1 | 18.63 | 0.559788 | 1.6 | 18.57 | 0.535904 | 1.5 | 18.91 | 0.612536 | 1.9 | 18.95 | 0.614797 | 1.9 |
| 309 | Azurocidin OS=Homo sapiens GN=AZU1 PE=1 SV=3 | CAP7_HUMAN | 27 kDa |  | 0.0001 | 1.60 | 1.60 | 1.90 | 17.56 | 1.147246 | 17.74 | 1.116171 | 18.23 | 0.556247 | 1.7 | 18.28 | 0.560068 | 1.5 | 18.27 | 0.57026 | 1.6 | 18.39 | 0.530492 | 1.6 | 18.43 | 0.552049 | 1.9 | 18.52 | 0.648781 | 1.9 |
| 1216 | Transportin-1 OS=Homo sapiens GN=TNPO1 PE=1 SV=2 | TNPO1_HUMAN | 102 kDa |  | 0.00049 | 1.45 | 1.60 | 1.90 | 18.07 | 0.720301 | 18.2 | 0.770744 | 18.65 | 0.520108 | 1.5 | 18.68 | 0.526841 | 1.4 | 18.86 | 0.531579 | 1.8 | 18.62 | 0.506277 | 1.4 | 19.01 | 0.588029 | 2 | 18.99 | 0.543353 | 1.8 |
| 966 | 40S ribosomal protein S12 OS=Homo sapiens GN=RPS12 PE=1 SV=3 | RS12_HUMAN | 15 kDa |  | 0.0052 | 1.40 | 1.60 | 1.90 | 17.08 | 0.814793 | 17.05 | 0.669406 | 17.49 | 0.525752 | 1.3 | 17.68 | 0.553472 | 1.5 | 17.75 | 0.517082 | 1.6 | 17.69 | 0.55353 | 1.6 | 17.95 | 0.566634 | 1.8 | 17.98 | 0.606268 | 2 |
| 1202 | Prefoldin subunit 2 OS=Homo sapiens GN=PFDN2 PE=1 SV=1 | PFD2_HUMAN | 17 kDa |  | 0.0001 | 1.20 | 1.60 | 1.90 | 17.48 | 0.656695 | 17.5 | 0.622897 | 17.75 | 0.505961 | 1.2 | 17.79 | 0.495289 | 1.2 | 18.15 | 0.521084 | 1.6 | 18.1 | 0.553188 | 1.6 | 18.38 | 0.532601 | 1.9 | 18.39 | 0.539932 | 1.9 |
| 1159 | Cystatin-B OS=Homo sapiens GN=CSTB PE=1 SV=2 | CYTB_HUMAN | 11 kDa |  | 0.00094 | 1.15 | 1.60 | 1.90 | 17.48 | 0.749345 | 17.63 | 0.613537 | 17.7 | 0.554862 | 1.2 | 17.76 | 0.526874 | 1.1 | 18.3 | 0.562135 | 1.8 | 18.06 | 0.497112 | 1.4 | 18.5 | 0.562078 | 2.1 | 18.42 | 0.568608 | 1.7 |
| 389 | Ribonuclease inhibitor OS=Homo sapiens GN=RNH1 PE=1 SV=2 | RINI_HUMAN | 50 kDa |  | 0.0001 | 1.70 | 1.65 | 1.90 | 17.43 | 0.908285 | 17.53 | 0.842989 | 18.19 | 0.573976 | 1.8 | 18.17 | 0.553068 | 1.6 | 18.16 | 0.533568 | 1.7 | 18.14 | 0.549274 | 1.6 | 18.32 | 0.551352 | 1.9 | 18.38 | 0.565811 | 1.9 |
| 1670 | Ras-related protein Rab-27A OS=Homo sapiens GN=RAB27A PE=1 SV=3 | RB27A_HUMAN | 25 kDa |  | 0.0056 | 1.35 | 1.65 | 1.90 | 17.92 | 1.254131 | 17.84 | 0.991437 | 18.19 | 0.564401 | 1.3 | 18.25 | 0.5685 | 1.4 | 18.6 | 0.567829 | 1.6 | 18.53 | 0.504953 | 1.7 | 18.67 | 0.537735 | 1.7 | 18.83 | 0.521006 | 2.1 |
| 51 | Transitional endoplasmic reticulum ATPase OS=Homo sapiens GN=VCP PE=1 SV=4 | TERA_HUMAN | 89 kDa | TRUE | 0.0001 | 1.50 | 1.70 | 1.90 | 17.86 | 1.086401 | 17.88 | 1.052964 | 18.37 | 0.575302 | 1.5 | 18.39 | 0.581876 | 1.5 | 18.57 | 0.578004 | 1.7 | 18.55 | 0.561076 | 1.7 | 18.73 | 0.581982 | 1.9 | 18.72 | 0.593911 | 1.9 |
| 963 | Reticulocalbin-1 OS=Homo sapiens GN=RCN1 PE=1 SV=1 | RCN1_HUMAN | 39 kDa |  | 0.0001 | 1.75 | 1.75 | 1.90 | 17.45 | 0.756195 | 17.41 | 0.725494 | 18.12 | 0.608802 | 1.7 | 18.16 | 0.594324 | 1.8 | 18.28 | 0.539874 | 1.8 | 18.13 | 0.520124 | 1.7 | 18.31 | 0.550262 | 1.8 | 18.37 | 0.619597 | 2 |
| 200 | Spliceosome RNA helicase DDX39B OS=Homo sapiens GN=DDX39B PE=1 SV=1 | DX39B_HUMAN | 49 kDa | TRUE | 0.0031 | 1.65 | 1.75 | 1.90 | 16.92 | 0.895642 | 16.86 | 0.851376 | 17.52 | 0.541878 | 1.5 | 17.65 | 0.571517 | 1.8 | 17.72 | 0.531753 | 1.8 | 17.64 | 0.530803 | 1.7 | 17.76 | 0.530769 | 1.9 | 17.73 | 0.620949 | 1.9 |
| 158 | T-complex protein 1 subunit zeta OS=Homo sapiens GN=CCT6A PE=1 SV=3 | TCPZ_HUMAN | 58 kDa | TRUE | 0.0001 | 1.85 | 1.90 | 1.90 | 18.22 | 1.057925 | 18.19 | 0.943993 | 19.03 | 0.613735 | 1.8 | 19.07 | 0.605033 | 1.9 | 19.1 | 0.594635 | 1.9 | 19.04 | 0.58349 | 1.9 | 19.12 | 0.555218 | 1.9 | 19.09 | 0.655243 | 1.9 |
| 1332 | Inositol monophosphatase 2 OS=Homo sapiens GN=IMPA2 PE=1 SV=1 | IMPA2_HUMAN | 31 kDa |  | 0.0045 | 1.80 | 1.90 | 1.90 | 17.44 | 0.843537 | 17.57 | 1.023962 | 18.31 | 0.518332 | 1.8 | 18.4 | 0.518341 | 1.8 | 18.42 | 0.581159 | 2.1 | 18.33 | 0.50301 | 1.7 | 18.45 | 0.558774 | 2.1 | 18.31 | 0.502166 | 1.7 |
| 25 | Pyruvate kinase PKM OS=Homo sapiens GN=PKM PE=1 SV=4 | KPYM_HUMAN | 58 kDa | TRUE | 0.0001 | 1.45 | 1.90 | 1.90 | 17.43 | 0.959474 | 17.41 | 0.92397 | 17.91 | 0.688933 | 1.4 | 17.93 | 0.681099 | 1.5 | 18.25 | 0.672816 | 1.9 | 18.22 | 0.629979 | 1.9 | 18.24 | 0.614378 | 1.9 | 18.23 | 0.667723 | 1.9 |
| 1068 | Galactokinase OS=Homo sapiens GN=GALK1 PE=1 SV=1 | GALK1_HUMAN | 42 kDa | TRUE | 0.016 | 1.40 | 1.90 | 1.90 | 18 | 1.165211 | 17.93 | 1.087416 | 18.39 | 0.523359 | 1.3 | 18.53 | 0.518194 | 1.5 | 18.71 | 0.539459 | 1.7 | 18.94 | 0.588667 | 2.1 | 18.84 | 0.52809 | 1.9 | 18.84 | 0.573561 | 1.9 |
| 602 | Proteasome subunit beta type-6 OS=Homo sapiens GN=PSMB6 PE=1 SV=4 | PSB6_HUMAN | 25 kDa |  | 0.0001 | 1.70 | 1.95 | 1.90 | 18.03 | 0.855522 | 17.93 | 0.792582 | 18.66 | 0.689762 | 1.6 | 18.73 | 0.718834 | 1.8 | 18.9 | 0.629804 | 1.9 | 18.93 | 0.575168 | 2 | 18.85 | 0.538798 | 1.8 | 18.88 | 0.539411 | 2 |
| 375 | Thymidine phosphorylase OS=Homo sapiens GN=TYMP PE=1 SV=2 | TYPH_HUMAN | 50 kDa |  | 0.0001 | 1.45 | 1.95 | 1.90 | 17.86 | 1.068831 | 17.79 | 0.905436 | 18.31 | 0.543768 | 1.4 | 18.32 | 0.549138 | 1.5 | 18.73 | 0.573733 | 1.9 | 18.74 | 0.541225 | 2 | 18.65 | 0.532221 | 1.7 | 18.78 | 0.609874 | 2.1 |
| 333 | Hemoglobin subunit gamma-2 OS=Homo sapiens GN=HBG2 PE=1 SV=2 | HBG2_HUMAN | 16 kDa | TRUE | 0.0001 | 1.55 | 1.95 | 1.90 | 17.81 | 0.861968 | 17.86 | 0.851154 | 18.5 | 0.741351 | 1.6 | 18.44 | 0.724778 | 1.5 | 18.83 | 0.67522 | 2.1 | 18.68 | 0.588961 | 1.8 | 18.79 | 0.617087 | 2 | 18.69 | 0.611904 | 1.8 |
| 1810 | Inositol polyphosphate 1-phosphatase OS=Homo sapiens GN=INPP1 PE=1 SV=1 | INPP_HUMAN | 44 kDa |  | 0.019 | 1.70 | 2.05 | 1.90 | 14.72 | 0.639759 | 14.46 | 0.478193 | 15.24 | 0.474005 | 1.4 | 15.48 | 0.470558 | 2 | 15.51 | 0.478103 | 1.7 | 15.72 | 0.490668 | 2.4 | 15.61 | 0.48469 | 1.9 | 15.41 | 0.47602 | 1.9 |
| 1394 | Ribulose-phosphate 3-epimerase OS=Homo sapiens GN=RPE PE=1 SV=1 | RPE_HUMAN | 25 kDa |  | 0.0097 | 2.00 | 2.10 | 1.90 | 17.25 | 1.367179 | 17.12 | 1.282714 | 18.16 | 0.522437 | 1.9 | 18.15 | 0.528894 | 2.1 | 18.38 | 0.505009 | 2.1 | 18.23 | 0.496068 | 2.1 | 18.2 | 0.498347 | 1.9 | 18.13 | 0.52079 | 1.9 |
| 415 | Nicotinate phosphoribosyltransferase OS=Homo sapiens GN=NAPRT PE=1 SV=2 | PNCB_HUMAN | 58 kDa |  | 0.0001 | 1.85 | 2.10 | 1.90 | 17.84 | 1.037468 | 18 | 1.06714 | 18.75 | 0.584158 | 1.9 | 18.82 | 0.589047 | 1.8 | 18.87 | 0.675989 | 2.2 | 18.93 | 0.542909 | 2 | 18.72 | 0.566047 | 2 | 18.85 | 0.730195 | 1.8 |
| 254 | GTP-binding nuclear protein Ran OS=Homo sapiens GN=RAN PE=1 SV=3 | RAN_HUMAN | 24 kDa |  | 0.0001 | 1.95 | 2.25 | 1.90 | 18.08 | 1.14417 | 18 | 1.005738 | 18.98 | 0.601173 | 2 | 18.95 | 0.574784 | 1.9 | 19.2 | 0.59175 | 2.4 | 19.03 | 0.573093 | 2.1 | 18.92 | 0.546517 | 1.9 | 18.85 | 0.579222 | 1.9 |
| 670 | Bifunctional ATP-dependent dihydroxyacetone kinase/FAD-AMP lyase (cyclizing) OS=Homo sapiens GN=DAK PE=1 SV=2 | DHAK_HUMAN | ? |  | 0.0001 | 1.90 | 2.25 | 1.90 | 16.76 | 0.717484 | 16.56 | 0.766849 | 17.63 | 0.525171 | 1.8 | 17.58 | 0.528593 | 2 | 17.88 | 0.566645 | 2.2 | 17.77 | 0.51405 | 2.3 | 17.5 | 0.531991 | 1.7 | 17.61 | 0.593939 | 2.1 |
| 1220 | U5 small nuclear ribonucleoprotein 200 kDa helicase OS=Homo sapiens GN=SNRNP200 PE=1 SV=2 | U520_HUMAN | 245 kDa |  | 0.0016 | 1.85 | 1.70 | 1.90 | 15.71 | 0.905037 | 15.66 | 0.916745 | 16.54 | 0.490702 | 1.9 | 16.53 | 0.495667 | 1.8 | 16.27 | 0.532167 | 1.5 | 16.52 | 0.500916 | 1.9 | 16.47 | 0.563296 | 1.6 | 16.8 | 0.555118 | 2.2 |
| 1535 | BRISC and BRCA1-A complex member 1 OS=Homo sapiens GN=BABAM1 PE=1 SV=1 | BABA1_HUMAN | 37 kDa |  | 0.00094 | 1.40 | 2.00 | 1.90 | 17.55 | 0.695017 | 17.78 | 0.601207 | 18.17 | 0.493002 | 1.5 | 18.17 | 0.500644 | 1.3 | 18.76 | 0.697158 | 2.3 | 18.57 | 0.554358 | 1.7 | 18.65 | 0.569716 | 2.2 | 18.5 | 0.504075 | 1.6 |
| 807 | 40S ribosomal protein S23 OS=Homo sapiens GN=RPS23 PE=1 SV=3 | RS23_HUMAN | 16 kDa |  | 0.0038 | -1.05 | 1.30 | 1.95 | 18.03 | 0.65866 | 18.26 | 0.703766 | 18.07 | 1.088986 | 1.1 | 17.91 | 1.059527 | 0.8 | 18.65 | 0.523637 | 1.5 | 18.43 | 0.522667 | 1.1 | 19.07 | 0.602943 | 2 | 19.17 | 0.568366 | 1.9 |
| 292 | Deoxynucleoside triphosphate triphosphohydrolase SAMHD1 OS=Homo sapiens GN=SAMHD1 PE=1 SV=2 | SAMH1_HUMAN | 72 kDa |  | 0.0001 | 1.25 | 1.45 | 1.95 | 17.02 | 0.848755 | 17.06 | 0.869101 | 17.3 | 0.521463 | 1.2 | 17.39 | 0.524405 | 1.3 | 17.54 | 0.519222 | 1.5 | 17.55 | 0.531334 | 1.4 | 17.93 | 0.52719 | 1.9 | 17.98 | 0.554238 | 2 |
| 704 | PITH domain-containing protein 1 OS=Homo sapiens GN=PITHD1 PE=1 SV=1 | PITH1_HUMAN | 24 kDa |  | 0.014 | 1.65 | 1.55 | 1.95 | 16.93 | 0.92143 | 16.83 | 0.958429 | 17.6 | 0.619583 | 1.6 | 17.6 | 0.642177 | 1.7 | 17.56 | 0.531476 | 1.5 | 17.6 | 0.542497 | 1.6 | 17.82 | 0.536648 | 1.9 | 17.83 | 0.56359 | 2 |
| 465 | Guanine nucleotide-binding protein subunit beta-2-like 1 OS=Homo sapiens GN=GNB2L1 PE=1 SV=3 | GBLP_HUMAN | 35 kDa |  | 0.0001 | 1.45 | 1.60 | 1.95 | 18.14 | 0.846765 | 18.22 | 0.76646 | 18.66 | 0.549899 | 1.5 | 18.67 | 0.563918 | 1.4 | 18.88 | 0.599969 | 1.6 | 18.87 | 0.564372 | 1.6 | 19.14 | 0.613908 | 2 | 19.14 | 0.604801 | 1.9 |
| 1155 | Tubulin beta-3 chain OS=Homo sapiens GN=TUBB3 PE=1 SV=2 | TBB3_HUMAN | 50 kDa | TRUE | 0.00051 | 1.35 | 1.60 | 1.95 | 17.27 | 0.696218 | 17.45 | 0.629084 | 17.73 | 0.568099 | 1.4 | 17.73 | 0.574955 | 1.3 | 17.82 | 0.534807 | 1.5 | 18.2 | 0.546757 | 1.7 | 18.24 | 0.534082 | 2 | 18.45 | 0.574956 | 1.9 |
| 863 | Ras GTPase-activating protein-binding protein 1 OS=Homo sapiens GN=G3BP1 PE=1 SV=1 | G3BP1_HUMAN | 52 kDa | TRUE | 0.0001 | 1.20 | 1.65 | 1.95 | 18.13 | 0.788541 | 18.16 | 0.71454 | 18.34 | 0.501636 | 1.2 | 18.42 | 0.518208 | 1.2 | 18.81 | 0.515722 | 1.6 | 18.91 | 0.536514 | 1.7 | 19.1 | 0.589777 | 1.9 | 19.2 | 0.525718 | 2 |
| 957 | Pre-mRNA-processing factor 19 OS=Homo sapiens GN=PRPF19 PE=1 SV=1 | PRP19_HUMAN | 55 kDa |  | 0.0015 | 1.70 | 1.70 | 1.95 | 18.04 | 0.806954 | 17.58 | 0.779079 | 18.52 | 0.511608 | 1.4 | 18.54 | 0.506252 | 2 | 18.55 | 0.502736 | 1.4 | 18.48 | 0.550145 | 2 | 18.7 | 0.513856 | 1.5 | 18.74 | 0.525706 | 2.4 |
| 232 | Proteasome subunit alpha type-6 OS=Homo sapiens GN=PSMA6 PE=1 SV=1 | PSA6_HUMAN | 27 kDa |  | 0.0001 | 1.90 | 1.75 | 1.95 | 17.49 | 0.80627 | 17.48 | 0.76178 | 18.42 | 0.555198 | 1.9 | 18.37 | 0.576176 | 1.9 | 18.36 | 0.548136 | 1.8 | 18.28 | 0.547262 | 1.7 | 18.44 | 0.547122 | 2 | 18.38 | 0.566122 | 1.9 |
| 1057 | Calcium-regulated heat stable protein 1 OS=Homo sapiens GN=CARHSP1 PE=1 SV=2 | CHSP1_HUMAN | 16 kDa |  | 0.0069 | 1.65 | 1.80 | 1.95 | 17.97 | 0.902208 | 18.06 | 0.896525 | 18.82 | 0.538111 | 1.8 | 18.67 | 0.509879 | 1.5 | 18.93 | 0.525003 | 1.9 | 18.8 | 0.516281 | 1.7 | 18.92 | 0.521096 | 1.9 | 18.96 | 0.755597 | 2 |
| 217 | 14-3-3 protein beta/alpha OS=Homo sapiens GN=YWHAB PE=1 SV=3 | 1433B_HUMAN | 28 kDa | TRUE | 0.0011 | 1.55 | 1.80 | 1.95 | 17.33 | 0.865837 | 17.29 | 0.873419 | 18 | 0.664659 | 1.5 | 18.05 | 0.630353 | 1.6 | 18.17 | 0.609454 | 1.9 | 18.07 | 0.560865 | 1.7 | 18.21 | 0.553376 | 1.9 | 18.22 | 0.564468 | 2 |
| 1187 | cAMP-dependent protein kinase catalytic subunit alpha OS=Homo sapiens GN=PRKACA PE=1 SV=2 | KAPCA_HUMAN | 41 kDa |  | 0.014 | 1.90 | 1.80 | 1.95 | 17.56 | 1.057723 | 17.14 | 0.775109 | 18.17 | 0.676641 | 1.8 | 18.18 | 0.668782 | 2 | 18.2 | 0.513722 | 1.6 | 18.19 | 0.528524 | 2 | 18.25 | 0.525634 | 1.6 | 18.23 | 0.713903 | 2.3 |
| 183 | 26S protease regulatory subunit 6A OS=Homo sapiens GN=PSMC3 PE=1 SV=3 | PRS6A_HUMAN | 49 kDa | TRUE | 0.0001 | 1.70 | 1.80 | 1.95 | 17.2 | 0.816738 | 17.19 | 0.762237 | 17.94 | 0.528292 | 1.7 | 17.96 | 0.521104 | 1.7 | 18.09 | 0.541044 | 1.8 | 18 | 0.542301 | 1.8 | 18.13 | 0.539445 | 1.9 | 18.17 | 0.539332 | 2 |
| 219 | Flavin reductase (NADPH) OS=Homo sapiens GN=BLVRB PE=1 SV=3 | BLVRB_HUMAN | 22 kDa |  | 0.0001 | 1.50 | 1.80 | 1.95 | 18.07 | 0.897594 | 18.18 | 0.776779 | 18.62 | 0.584028 | 1.5 | 18.69 | 0.562671 | 1.5 | 18.96 | 0.547666 | 1.8 | 18.98 | 0.548488 | 1.8 | 19.06 | 0.548663 | 2 | 19.12 | 0.546275 | 1.9 |
| 256 | Retinal dehydrogenase 1 OS=Homo sapiens GN=ALDH1A1 PE=1 SV=2 | AL1A1_HUMAN | 55 kDa | TRUE | 0.0001 | 1.55 | 1.85 | 1.95 | 18.11 | 1.207693 | 18.19 | 1.099187 | 18.74 | 0.567379 | 1.6 | 18.77 | 0.571604 | 1.5 | 19.01 | 0.577357 | 1.9 | 18.9 | 0.604798 | 1.8 | 18.94 | 0.57262 | 1.9 | 18.97 | 0.661413 | 2 |
| 673 | Keratin, type I cytoskeletal 17 OS=Homo sapiens GN=KRT17 PE=1 SV=2 | K1C17_HUMAN | 48 kDa | TRUE | 0.0001 | 1.00 | 1.95 | 1.95 | 17.1 | 0.609744 | 17.09 | 0.591135 | 17.16 | 0.737392 | 1 | 17.1 | 0.649789 | 1 | 18.12 | 0.7398 | 2 | 18.01 | 0.545964 | 1.9 | 18 | 0.613109 | 1.9 | 18.06 | 0.662963 | 2 |
| 340 | Transforming protein RhoA OS=Homo sapiens GN=RHOA PE=1 SV=1 | RHOA_HUMAN | 22 kDa | TRUE | 0.0001 | 1.75 | 2.05 | 1.95 | 17.49 | 1.210352 | 17.49 | 1.101719 | 18.35 | 0.710417 | 1.7 | 18.39 | 0.666396 | 1.8 | 18.54 | 0.563007 | 2.2 | 18.33 | 0.53988 | 1.9 | 18.43 | 0.526406 | 2 | 18.4 | 0.581945 | 1.9 |
| 83 | Glucose-6-phosphate 1-dehydrogenase OS=Homo sapiens GN=G6PD PE=1 SV=4 | G6PD_HUMAN | 59 kDa |  | 0.0001 | 2.30 | 2.10 | 1.95 | 17.91 | 1.205222 | 18.03 | 1.053197 | 19.14 | 0.724145 | 2.3 | 19.13 | 0.725169 | 2.3 | 19.08 | 0.630293 | 2.2 | 19 | 0.586143 | 2 | 18.89 | 0.563997 | 2 | 18.92 | 0.642769 | 1.9 |
| 1230 | Collagen alpha-1(I) chain OS=Homo sapiens GN=COL1A1 PE=1 SV=5 | CO1A1_HUMAN | 139 kDa |  | 0.013 | 1.05 | 2.15 | 1.95 | 18.92 | 0.599302 | 19.01 | 0.569309 | 18.94 | 0.522571 | 1 | 19.09 | 0.523947 | 1.1 | 20.06 | 0.537034 | 2.2 | 20.08 | 0.537086 | 2.1 | 19.96 | 0.525297 | 2 | 19.96 | 0.537895 | 1.9 |
| 369 | L-lactate dehydrogenase B chain OS=Homo sapiens GN=LDHB PE=1 SV=2 | LDHB_HUMAN | 37 kDa | TRUE | 0.0001 | 2.25 | 2.30 | 1.95 | 17.49 | 1.037208 | 17.58 | 0.925951 | 18.69 | 0.707134 | 2.4 | 18.73 | 0.683883 | 2.1 | 18.83 | 0.645518 | 2.5 | 18.66 | 0.568989 | 2.1 | 18.51 | 0.572441 | 2 | 18.48 | 0.629913 | 1.9 |
| 452 | Nucleolin OS=Homo sapiens GN=NCL PE=1 SV=3 | NUCL_HUMAN | 77 kDa | TRUE | 0.0001 | 1.55 | 1.65 | 1.95 | 17.89 | 0.701183 | 17.96 | 0.680765 | 18.53 | 0.523083 | 1.6 | 18.56 | 0.527163 | 1.5 | 18.67 | 0.567669 | 1.8 | 18.51 | 0.557435 | 1.5 | 18.95 | 0.54511 | 2.1 | 18.79 | 0.573225 | 1.8 |
| 1406 | Ataxin-3 OS=Homo sapiens GN=ATXN3 PE=1 SV=4 | ATX3_HUMAN | 42 kDa |  | 0.0017 | 1.30 | 1.65 | 1.95 | 16.79 | 0.770804 | 17.13 | 1.029469 | 17.25 | 0.508063 | 1.4 | 17.3 | 0.503064 | 1.2 | 17.51 | 0.502221 | 1.7 | 17.65 | 0.546849 | 1.6 | 17.8 | 0.602027 | 2.2 | 17.76 | 0.531255 | 1.7 |
| 1824 | Signal transducer and activator of transcription 5A OS=Homo sapiens GN=STAT5A PE=1 SV=1 | STA5A_HUMAN | 91 kDa | TRUE | 0.013 | 1.35 | 1.70 | 1.95 | 17.1 | 0.741816 | 17.04 | 0.94175 | 17.52 | 0.531957 | 1.3 | 17.54 | 0.495065 | 1.4 | 17.83 | 0.495144 | 1.7 | 17.83 | 0.50098 | 1.7 | 17.88 | 0.49873 | 1.7 | 18.17 | 0.503451 | 2.2 |
| 1172 | Long-chain-fatty-acid--CoA ligase 1 OS=Homo sapiens GN=ACSL1 PE=1 SV=1 | ACSL1_HUMAN | 78 kDa | TRUE | 0.0001 | 1.85 | 1.70 | 1.95 | 17.15 | 0.783134 | 17.01 | 1.022015 | 17.93 | 0.526768 | 1.8 | 17.87 | 0.529675 | 1.9 | 17.77 | 0.502932 | 1.6 | 17.78 | 0.534451 | 1.8 | 17.92 | 0.504043 | 1.8 | 17.99 | 0.51631 | 2.1 |
| 1427 | Peptidyl-prolyl cis-trans isomerase NIMA-interacting 1 OS=Homo sapiens GN=PIN1 PE=1 SV=1 | PIN1_HUMAN | 18 kDa |  | 0.00013 | 1.50 | 1.75 | 1.95 | 18.06 | 0.757948 | 17.83 | 0.75644 | 18.55 | 0.523012 | 1.4 | 18.53 | 0.508294 | 1.6 | 18.72 | 0.541601 | 1.7 | 18.68 | 0.557282 | 1.8 | 18.84 | 0.51552 | 1.8 | 18.86 | 0.517611 | 2.1 |
| 110 | Calmodulin OS=Homo sapiens GN=CALM1 PE=1 SV=2 | CALM_HUMAN | 17 kDa | TRUE | 0.0001 | 1.35 | 1.75 | 1.95 | 17.12 | 0.87618 | 17.26 | 0.830131 | 17.52 | 0.607203 | 1.4 | 17.57 | 0.593211 | 1.3 | 17.86 | 0.572589 | 1.7 | 17.96 | 0.644169 | 1.8 | 18.09 | 0.644986 | 2.1 | 18.06 | 0.625069 | 1.8 |
| 184 | Calreticulin OS=Homo sapiens GN=CALR PE=1 SV=1 | CALR_HUMAN | 48 kDa |  | 0.0001 | 1.35 | 1.75 | 1.95 | 18.17 | 0.817547 | 18.31 | 0.835181 | 18.64 | 0.583531 | 1.4 | 18.69 | 0.561279 | 1.3 | 19.07 | 0.613779 | 1.9 | 18.92 | 0.544416 | 1.6 | 19.24 | 0.617144 | 2.2 | 19.07 | 0.587043 | 1.7 |
| 1336 | N-acetylneuraminate lyase OS=Homo sapiens GN=NPL PE=1 SV=1 | NPL_HUMAN | 35 kDa |  | 0.013 | 2.00 | 1.80 | 1.95 | 17.89 | 0.734745 | 18.15 | 0.597226 | 18.9 | 0.68816 | 2 | 19.11 | 0.615935 | 2 | 18.7 | 0.652606 | 1.9 | 18.9 | 0.530956 | 1.7 | 19.07 | 0.572055 | 2.2 | 18.95 | 0.515078 | 1.7 |
| 103 | Stress-induced-phosphoprotein 1 OS=Homo sapiens GN=STIP1 PE=1 SV=1 | STIP1_HUMAN | 63 kDa |  | 0.0001 | 1.90 | 1.90 | 1.95 | 17.98 | 1.062945 | 18.06 | 0.933116 | 18.88 | 0.579178 | 1.9 | 18.9 | 0.582088 | 1.9 | 18.96 | 0.554122 | 2 | 18.86 | 0.558264 | 1.8 | 18.99 | 0.537826 | 2.1 | 18.9 | 0.586717 | 1.8 |
| 713 | Heterogeneous nuclear ribonucleoprotein A/B OS=Homo sapiens GN=HNRNPAB PE=1 SV=2 | ROAA_HUMAN | 36 kDa | TRUE | 0.00026 | 1.90 | 1.95 | 1.95 | 16.97 | 0.777979 | 16.98 | 0.715387 | 17.88 | 0.600123 | 1.9 | 17.92 | 0.594377 | 1.9 | 17.8 | 0.508993 | 1.8 | 18.03 | 0.566242 | 2.1 | 18.01 | 0.515603 | 2.1 | 17.83 | 0.6232 | 1.8 |
| 927 | 40S ribosomal protein S18 OS=Homo sapiens GN=RPS18 PE=1 SV=3 | RS18_HUMAN | 18 kDa |  | 0.0045 | 1.20 | 1.40 | 2.00 | 18.11 | 0.827409 | 18.23 | 0.717153 | 18.4 | 0.530895 | 1.2 | 18.4 | 0.522281 | 1.2 | 18.62 | 0.57427 | 1.4 | 18.65 | 0.565667 | 1.4 | 19.03 | 0.682629 | 1.9 | 19.28 | 0.549322 | 2.1 |
| 684 | 40S ribosomal protein S5 OS=Homo sapiens GN=RPS5 PE=1 SV=4 | RS5_HUMAN | 23 kDa | TRUE | 0.0001 | 1.60 | 1.45 | 2.00 | 17.95 | 0.668116 | 18.16 | 0.655274 | 18.68 | 0.527711 | 1.7 | 18.68 | 0.505661 | 1.5 | 18.5 | 0.575479 | 1.4 | 18.7 | 0.562956 | 1.5 | 18.92 | 0.52819 | 2 | 19.08 | 0.668859 | 2 |
| 975 | SPARC OS=Homo sapiens GN=SPARC PE=1 SV=1 | SPRC_HUMAN | 35 kDa |  | 0.0005 | 1.60 | 1.50 | 2.00 | 17.54 | 1.087194 | 17.7 | 1.114394 | 18.21 | 0.663118 | 1.7 | 18.26 | 0.651923 | 1.5 | 18.2 | 0.571034 | 1.6 | 18.16 | 0.587602 | 1.4 | 18.57 | 0.582515 | 2.1 | 18.53 | 0.640958 | 1.9 |
| 37 | Vimentin OS=Homo sapiens GN=VIM PE=1 SV=4 | VIME_HUMAN | 54 kDa | TRUE | 0.0001 | 1.35 | 1.55 | 2.00 | 17.91 | 0.921275 | 17.87 | 0.900739 | 18.32 | 0.62411 | 1.3 | 18.36 | 0.629934 | 1.4 | 18.44 | 0.603636 | 1.5 | 18.48 | 0.609137 | 1.6 | 18.82 | 0.630132 | 2 | 18.82 | 0.630214 | 2 |
| 1286 | Signal recognition particle 54 kDa protein OS=Homo sapiens GN=SRP54 PE=1 SV=1 | SRP54_HUMAN | 56 kDa |  | 0.0022 | 1.85 | 1.60 | 2.00 | 16.4 | 1.268094 | 16.32 | 0.797205 | 17.31 | 0.524719 | 1.9 | 17.21 | 0.524946 | 1.8 | 16.95 | 0.510146 | 1.6 | 17.02 | 0.560042 | 1.6 | 17.4 | 0.537327 | 1.9 | 17.41 | 0.506897 | 2.1 |
| 584 | Asparagine--tRNA ligase, cytoplasmic OS=Homo sapiens GN=NARS PE=1 SV=1 | SYNC_HUMAN | 63 kDa | TRUE | 0.0001 | 1.70 | 1.60 | 2.00 | 17.39 | 0.69503 | 17.39 | 0.723099 | 18.15 | 0.525962 | 1.7 | 18.17 | 0.521911 | 1.7 | 18.11 | 0.556783 | 1.6 | 18.07 | 0.52217 | 1.6 | 18.39 | 0.513163 | 2 | 18.4 | 0.514771 | 2 |
| 466 | Heterogeneous nuclear ribonucleoprotein D0 OS=Homo sapiens GN=HNRNPD PE=1 SV=1 | HNRPD_HUMAN | 38 kDa | TRUE | 0.0061 | 1.55 | 1.65 | 2.00 | 17.11 | 1.057996 | 17.16 | 0.848513 | 17.71 | 0.568647 | 1.6 | 17.69 | 0.627044 | 1.5 | 17.99 | 0.546971 | 1.8 | 17.75 | 0.505908 | 1.5 | 18.13 | 0.563704 | 2.1 | 18 | 0.625909 | 1.9 |
| 919 | Polyadenylate-binding protein 4 OS=Homo sapiens GN=PABPC4 PE=1 SV=1 | PABP4_HUMAN | 71 kDa | TRUE | 0.0001 | 1.45 | 1.65 | 2.00 | 16.98 | 0.528719 | 16.97 | 0.513938 | 17.48 | 0.508168 | 1.4 | 17.51 | 0.498951 | 1.5 | 17.7 | 0.529386 | 1.7 | 17.66 | 0.604907 | 1.6 | 18.04 | 0.571253 | 2.1 | 17.93 | 0.554621 | 1.9 |
| 1426 | Poly(rC)-binding protein 2 OS=Homo sapiens GN=PCBP2 PE=1 SV=1 | PCBP2_HUMAN | 39 kDa | TRUE | 0.00036 | 1.40 | 1.65 | 2.00 | 18.3 | 0.697917 | 18.49 | 0.755147 | 18.83 | 0.547461 | 1.5 | 18.83 | 0.535062 | 1.3 | 19.08 | 0.513495 | 1.8 | 19.05 | 0.527477 | 1.5 | 19.21 | 0.522647 | 2 | 19.42 | 0.571786 | 2 |
| 1004 | High mobility group protein B1 OS=Homo sapiens GN=HMGB1 PE=1 SV=3 | HMGB1_HUMAN | 25 kDa | TRUE | 0.00049 | 1.70 | 1.70 | 2.00 | 17.85 | 0.827352 | 17.99 | 0.817817 | 18.61 | 0.569303 | 1.8 | 18.64 | 0.572393 | 1.6 | 18.68 | 0.644026 | 2 | 18.39 | 0.615375 | 1.4 | 18.86 | 0.641098 | 2.2 | 18.7 | 0.696136 | 1.8 |
| 1523 | Protein unc-45 homolog A OS=Homo sapiens GN=UNC45A PE=1 SV=1 | UN45A_HUMAN | 103 kDa |  | 0.001 | 1.70 | 1.70 | 2.00 | 15.34 | 0.69858 | 15.59 | 0.762602 | 16.21 | 0.497064 | 1.8 | 16.24 | 0.491793 | 1.6 | 16.07 | 0.605164 | 1.6 | 16.44 | 0.611399 | 1.8 | 16.29 | 0.54723 | 1.9 | 16.59 | 0.576726 | 2.1 |
| 1029 | Transcriptional activator protein Pur-alpha OS=Homo sapiens GN=PURA PE=1 SV=2 | PURA_HUMAN | 35 kDa | TRUE | 0.0014 | 1.70 | 1.80 | 2.00 | 17.16 | 0.795629 | 17.08 | 0.767266 | 17.86 | 0.655069 | 1.7 | 17.83 | 0.653507 | 1.7 | 18.05 | 0.563327 | 1.9 | 17.89 | 0.542388 | 1.7 | 18.15 | 0.551282 | 2 | 18.05 | 0.54995 | 2 |
| 690 | Vacuolar protein sorting-associated protein 26A OS=Homo sapiens GN=VPS26A PE=1 SV=2 | VP26A_HUMAN | 38 kDa | TRUE | 0.002 | 1.65 | 1.85 | 2.00 | 17.08 | 0.843322 | 17.15 | 0.802136 | 17.76 | 0.626357 | 1.7 | 17.8 | 0.667268 | 1.6 | 18.04 | 0.529716 | 1.9 | 17.97 | 0.505375 | 1.8 | 18.11 | 0.514307 | 2 | 18.18 | 0.575216 | 2 |
| 376 | Ubiquitin-associated domain-containing protein 1 OS=Homo sapiens GN=UBAC1 PE=1 SV=1 | UBAC1_HUMAN | 45 kDa | TRUE | 0.0001 | 1.65 | 1.90 | 2.00 | 17.01 | 0.891025 | 17.1 | 0.884668 | 17.86 | 0.570326 | 1.7 | 17.83 | 0.583424 | 1.6 | 17.96 | 0.57005 | 1.9 | 18.03 | 0.615705 | 1.9 | 18.06 | 0.534797 | 2.1 | 18.03 | 0.53932 | 1.9 |
| 71 | WD repeat-containing protein 1 OS=Homo sapiens GN=WDR1 PE=1 SV=4 | WDR1_HUMAN | 66 kDa |  | 0.0001 | 1.55 | 1.90 | 2.00 | 17.52 | 1.185042 | 17.58 | 1.070071 | 18.13 | 0.618122 | 1.6 | 18.15 | 0.599385 | 1.5 | 18.47 | 0.604129 | 2.1 | 18.29 | 0.586148 | 1.7 | 18.49 | 0.583813 | 2.1 | 18.42 | 0.6016 | 1.9 |
| 157 | Proteasome subunit alpha type-1 OS=Homo sapiens GN=PSMA1 PE=1 SV=1 | PSA1_HUMAN | 30 kDa |  | 0.0001 | 1.45 | 1.90 | 2.00 | 17.48 | 0.959757 | 17.53 | 0.912148 | 18.07 | 0.672792 | 1.5 | 18.04 | 0.668726 | 1.4 | 18.41 | 0.593413 | 2 | 18.33 | 0.593034 | 1.8 | 18.43 | 0.608885 | 2 | 18.49 | 0.643901 | 2 |
| 248 | Protein S100-P OS=Homo sapiens GN=S100P PE=1 SV=2 | S100P_HUMAN | 10 kDa | TRUE | 0.00038 | 2.40 | 1.95 | 2.00 | 16.94 | 1.179542 | 16.99 | 1.024798 | 18.24 | 0.621476 | 2.5 | 18.22 | 0.660416 | 2.3 | 17.87 | 0.571031 | 1.9 | 17.9 | 0.599172 | 2 | 17.83 | 0.60113 | 2 | 17.89 | 0.570533 | 2 |
| 19 | Lactotransferrin OS=Homo sapiens GN=LTF PE=1 SV=6 | TRFL_HUMAN | 78 kDa | TRUE | 0.0001 | 1.50 | 2.10 | 2.00 | 17.54 | 1.299593 | 17.65 | 1.214348 | 18.16 | 0.655898 | 1.5 | 18.18 | 0.650153 | 1.5 | 18.58 | 0.627957 | 2.2 | 18.53 | 0.6112 | 2 | 18.54 | 0.64081 | 2.1 | 18.49 | 0.650822 | 1.9 |
| 1746 | Interleukin-1 beta OS=Homo sapiens GN=IL1B PE=1 SV=2 | IL1B_HUMAN | 31 kDa |  | 0.017 | 1.15 | 1.40 | 2.05 | 17.05 | 0.492643 | 17.28 | 0.50177 | 17.34 | 0.487933 | 1.2 | 17.36 | 0.486189 | 1.1 | 17.61 | 0.506436 | 1.5 | 17.62 | 0.494481 | 1.3 | 18.25 | 0.517642 | 2.3 | 18.1 | 0.674124 | 1.8 |
| 1865 | PHD finger-like domain-containing protein 5A OS=Homo sapiens GN=PHF5A PE=1 SV=1 | PHF5A_HUMAN | 12 kDa |  | 0.0074 | 1.45 | 1.70 | 2.05 | 17.7 | 0.500896 | 17.84 | 0.580131 | 18.26 | 0.494309 | 1.5 | 18.28 | 0.495382 | 1.4 | 18.62 | 0.572222 | 1.9 | 18.38 | 0.502584 | 1.5 | 18.86 | 0.558527 | 2.2 | 18.74 | 0.509835 | 1.9 |
| 1594 | Thiopurine S-methyltransferase OS=Homo sapiens GN=TPMT PE=1 SV=1 | TPMT_HUMAN | 28 kDa |  | 0.011 | 1.20 | 1.70 | 2.05 | 16.99 | 0.644072 | 16.64 | 0.79057 | 17.09 | 0.490033 | 1.1 | 17.06 | 0.543456 | 1.3 | 17.61 | 0.534889 | 1.5 | 17.59 | 0.508091 | 1.9 | 17.84 | 0.640493 | 1.8 | 17.85 | 0.665109 | 2.3 |
| 1087 | Serine/threonine-protein phosphatase PP1-alpha catalytic subunit OS=Homo sapiens GN=PPP1CA PE=1 SV=1 | PP1A_HUMAN | 38 kDa | TRUE | 0.0018 | 1.30 | 1.70 | 2.05 | 18.71 | 0.664435 | 18.78 | 0.680615 | 19.07 | 0.523728 | 1.3 | 19.17 | 0.523565 | 1.3 | 19.53 | 0.549288 | 1.8 | 19.41 | 0.517194 | 1.6 | 19.69 | 0.537655 | 2 | 19.78 | 0.538379 | 2.1 |
| 60 | Elongation factor 2 OS=Homo sapiens GN=EEF2 PE=1 SV=4 | EF2_HUMAN | 95 kDa | TRUE | 0.0001 | 1.40 | 1.75 | 2.05 | 17.81 | 0.905736 | 17.84 | 0.882524 | 18.28 | 0.605809 | 1.4 | 18.3 | 0.612354 | 1.4 | 18.66 | 0.594697 | 1.8 | 18.55 | 0.574432 | 1.7 | 18.82 | 0.58616 | 2.1 | 18.82 | 0.609833 | 2 |
| 839 | Alpha-soluble NSF attachment protein OS=Homo sapiens GN=NAPA PE=1 SV=3 | SNAA_HUMAN | 33 kDa |  | 0.0001 | 1.85 | 1.80 | 2.05 | 17.78 | 0.819548 | 17.9 | 0.766751 | 18.66 | 0.544806 | 1.9 | 18.73 | 0.542151 | 1.8 | 18.64 | 0.527834 | 1.8 | 18.69 | 0.509938 | 1.8 | 18.81 | 0.518448 | 2.1 | 18.88 | 0.603173 | 2 |
| 111 | Peroxiredoxin-2 OS=Homo sapiens GN=PRDX2 PE=1 SV=5 | PRDX2_HUMAN | 22 kDa | TRUE | 0.0001 | 1.55 | 1.90 | 2.05 | 18.36 | 0.880743 | 18.42 | 0.840196 | 19.01 | 0.648781 | 1.6 | 19.03 | 0.616123 | 1.5 | 19.22 | 0.840739 | 2 | 19.15 | 0.636978 | 1.8 | 19.21 | 0.739986 | 2 | 19.26 | 0.775954 | 2.1 |
| 362 | Mitogen-activated protein kinase 1 OS=Homo sapiens GN=MAPK1 PE=1 SV=3 | MK01_HUMAN | 41 kDa | TRUE | 0.0001 | 1.60 | 2.00 | 2.05 | 18.12 | 0.906303 | 18.22 | 0.924496 | 18.9 | 0.628662 | 1.6 | 18.92 | 0.619658 | 1.6 | 19.09 | 0.631468 | 2.1 | 19.11 | 0.599377 | 1.9 | 19.09 | 0.60982 | 2.1 | 19.12 | 0.626722 | 2 |
| 1297 | BRO1 domain-containing protein BROX OS=Homo sapiens GN=BROX PE=1 SV=1 | BROX_HUMAN | 46 kDa |  | 0.0018 | 1.35 | 2.05 | 2.05 | 17.43 | 0.684823 | 17.52 | 0.704852 | 17.92 | 0.549102 | 1.4 | 17.94 | 0.511085 | 1.3 | 18.48 | 0.562829 | 2.1 | 18.49 | 0.509603 | 2 | 18.51 | 0.535244 | 2.1 | 18.55 | 0.519654 | 2 |
| 460 | ADP-ribosylation factor 3 OS=Homo sapiens GN=ARF3 PE=1 SV=2 | ARF3_HUMAN | 21 kDa | TRUE | 0.00097 | 1.85 | 2.30 | 2.05 | 17.3 | 0.862631 | 17.19 | 0.816967 | 17.95 | 0.665967 | 1.7 | 18.14 | 0.67725 | 2 | 18.21 | 0.768718 | 2.2 | 18.26 | 0.752559 | 2.4 | 18.19 | 0.666568 | 2.1 | 18.13 | 0.522693 | 2 |
| 259 | Ferritin light chain OS=Homo sapiens GN=FTL PE=1 SV=2 | FRIL_HUMAN | 20 kDa |  | 0.00085 | 1.95 | 1.70 | 2.10 | 18.16 | 1.274871 | 18.15 | 1.103194 | 19.02 | 0.877341 | 2 | 19.01 | 0.861246 | 1.9 | 18.71 | 0.680354 | 1.7 | 18.81 | 0.653642 | 1.7 | 19.15 | 0.710659 | 2.3 | 18.91 | 0.638333 | 1.9 |
| 867 | UMP-CMP kinase OS=Homo sapiens GN=CMPK1 PE=1 SV=3 | KCY_HUMAN | 22 kDa |  | 0.0001 | 1.65 | 1.90 | 2.10 | 17.7 | 0.891249 | 17.38 | 1.095952 | 18.17 | 0.589794 | 1.5 | 18.21 | 0.550286 | 1.8 | 18.45 | 0.58324 | 1.7 | 18.44 | 0.530031 | 2.1 | 18.6 | 0.580409 | 1.9 | 18.52 | 0.550542 | 2.3 |
| 565 | Low molecular weight phosphotyrosine protein phosphatase OS=Homo sapiens GN=ACP1 PE=1 SV=3 | PPAC_HUMAN | 18 kDa |  | 0.0001 | 1.35 | 1.90 | 2.10 | 16.8 | 1.211803 | 16.94 | 1.033902 | 17.25 | 0.59045 | 1.4 | 17.35 | 0.581618 | 1.3 | 17.72 | 0.573718 | 2 | 17.74 | 0.572476 | 1.8 | 17.91 | 0.595144 | 2.3 | 17.86 | 0.611268 | 1.9 |
| 1419 | Hemoglobin subunit mu OS=Homo sapiens GN=HBM PE=2 SV=1 | HBM_HUMAN | 16 kDa |  | 0.024 | 1.50 | 1.50 | 2.10 | 17.63 | 0.831067 | 17.99 | 0.732201 | 18.35 | 0.515012 | 1.7 | 18.34 | 0.513186 | 1.3 | 18.22 | 0.555244 | 1.5 | 18.54 | 0.636155 | 1.5 | 18.59 | 0.536232 | 2 | 18.99 | 0.786964 | 2.2 |
| 1167 | 60S ribosomal protein L34 OS=Homo sapiens GN=RPL34 PE=1 SV=3 | RL34_HUMAN | 13 kDa |  | 0.0018 | 1.40 | 1.50 | 2.10 | 18.09 | 0.806522 | 17.88 | 0.69792 | 18.42 | 0.502498 | 1.3 | 18.44 | 0.518359 | 1.5 | 18.58 | 0.519609 | 1.5 | 18.42 | 0.519851 | 1.5 | 19.03 | 0.542094 | 2 | 19.02 | 0.568567 | 2.2 |
| 711 | 60S ribosomal protein L26 OS=Homo sapiens GN=RPL26 PE=1 SV=1 | RL26_HUMAN | 17 kDa |  | 0.0001 | 1.05 | 1.50 | 2.10 | 17.55 | 0.675751 | 17.58 | 0.625028 | 17.54 | 0.625753 | 1 | 17.75 | 0.555361 | 1.1 | 18.02 | 0.52464 | 1.4 | 18.14 | 0.705397 | 1.6 | 18.65 | 0.585034 | 2.1 | 18.65 | 0.531524 | 2.1 |
| 1386 | 40S ribosomal protein S15a OS=Homo sapiens GN=RPS15A PE=1 SV=2 | RS15A_HUMAN | 15 kDa |  | 0.00094 | 1.85 | 1.55 | 2.10 | 17.86 | 0.633077 | 17.91 | 0.717884 | 18.74 | 0.516503 | 1.8 | 18.8 | 0.521719 | 1.9 | 18.58 | 0.507978 | 1.6 | 18.54 | 0.53041 | 1.5 | 18.92 | 0.518626 | 2.1 | 19 | 0.515134 | 2.1 |
| 1144 | RNA-binding protein 8A OS=Homo sapiens GN=RBM8A PE=1 SV=1 | RBM8A_HUMAN | 20 kDa |  | 0.00012 | 1.45 | 1.70 | 2.10 | 18.04 | 0.969059 | 18.1 | 0.977911 | 18.54 | 0.573199 | 1.5 | 18.52 | 0.562843 | 1.4 | 18.87 | 0.560227 | 1.9 | 18.7 | 0.55081 | 1.5 | 19.17 | 0.571563 | 2.2 | 19.12 | 0.533378 | 2 |
| 477 | Acid ceramidase OS=Homo sapiens GN=ASAH1 PE=1 SV=5 | ASAH1_HUMAN | 45 kDa |  | 0.0001 | 1.60 | 1.75 | 2.10 | 17.33 | 0.729638 | 17.48 | 0.762643 | 18.08 | 0.597856 | 1.7 | 18.07 | 0.584064 | 1.5 | 18.21 | 0.535172 | 1.9 | 18.15 | 0.538571 | 1.6 | 18.49 | 0.552042 | 2.2 | 18.47 | 0.570816 | 2 |
| 562 | Niban-like protein 1 OS=Homo sapiens GN=FAM129B PE=1 SV=3 | NIBL1_HUMAN | 84 kDa |  | 0.0001 | 1.25 | 1.80 | 2.10 | 17.48 | 1.155585 | 17.45 | 1.029148 | 17.68 | 0.534762 | 1.2 | 17.74 | 0.525834 | 1.3 | 18.29 | 0.536996 | 1.9 | 18.15 | 0.530445 | 1.7 | 18.51 | 0.583705 | 2.1 | 18.52 | 0.585973 | 2.1 |
| 1325 | Probable ATP-dependent RNA helicase DDX58 OS=Homo sapiens GN=DDX58 PE=1 SV=2 | DDX58_HUMAN | 107 kDa | TRUE | 0.00094 | 1.45 | 1.90 | 2.10 | 17.6 | 0.533355 | 17.54 | 0.564182 | 18.12 | 0.499659 | 1.4 | 18.12 | 0.49929 | 1.5 | 18.48 | 0.499775 | 1.8 | 18.5 | 0.510901 | 2 | 18.62 | 0.551138 | 2 | 18.69 | 0.528102 | 2.2 |
| 54 | Coronin-1A OS=Homo sapiens GN=CORO1A PE=1 SV=4 | COR1A_HUMAN | 51 kDa | TRUE | 0.0001 | 2.00 | 2.15 | 2.10 | 17.88 | 1.253723 | 17.97 | 1.040127 | 18.82 | 0.700407 | 2 | 18.85 | 0.694134 | 2 | 18.94 | 0.630511 | 2.2 | 18.92 | 0.620492 | 2.1 | 18.9 | 0.603976 | 2.1 | 18.88 | 0.66045 | 2.1 |
| 1662 | Dickkopf-related protein 3 OS=Homo sapiens GN=DKK3 PE=1 SV=2 | DKK3_HUMAN | 38 kDa |  | 0.0018 | 1.85 | 2.25 | 2.10 | 17.23 | 0.741762 | 17.42 | 0.781541 | 18.18 | 0.496307 | 2 | 18.1 | 0.500079 | 1.7 | 18.47 | 0.512193 | 2.4 | 18.41 | 0.521432 | 2.1 | 18.3 | 0.497159 | 2.2 | 18.39 | 0.510969 | 2 |
| 70 | Phosphoglycerate kinase 1 OS=Homo sapiens GN=PGK1 PE=1 SV=3 | PGK1_HUMAN | 45 kDa | TRUE | 0.0001 | 1.50 | 2.30 | 2.10 | 17.47 | 1.009897 | 17.52 | 0.944604 | 18.03 | 0.659408 | 1.5 | 18.08 | 0.670046 | 1.5 | 18.65 | 0.674746 | 2.5 | 18.49 | 0.617328 | 2.1 | 18.5 | 0.587961 | 2.2 | 18.42 | 0.590417 | 2 |
| 1093 | 40S ribosomal protein S10 OS=Homo sapiens GN=RPS10 PE=1 SV=1 | RS10_HUMAN | 19 kDa |  | 0.00096 | 1.40 | 1.25 | 2.15 | 18.81 | 0.747893 | 18.64 | 0.67521 | 19.23 | 0.517659 | 1.4 | 19.14 | 0.525955 | 1.4 | 19.08 | 0.601467 | 1.2 | 19.07 | 0.524859 | 1.3 | 19.65 | 0.570466 | 1.8 | 19.96 | 0.597725 | 2.5 |
| 1615 | Golgi phosphoprotein 3 OS=Homo sapiens GN=GOLPH3 PE=1 SV=1 | GOLP3_HUMAN | 34 kDa |  | 0.00072 | 1.50 | 1.65 | 2.15 | 15.3 | 0.63035 | 15.78 | 0.650512 | 16.06 | 0.586515 | 1.7 | 16.16 | 0.584293 | 1.3 | 16.25 | 0.562194 | 1.9 | 16.24 | 0.497568 | 1.4 | 16.55 | 0.521306 | 2.4 | 16.74 | 0.519992 | 1.9 |
| 1554 | Developmentally-regulated GTP-binding protein 1 OS=Homo sapiens GN=DRG1 PE=1 SV=1 | DRG1_HUMAN | 41 kDa |  | 0.0022 | 1.65 | 1.75 | 2.15 | 18.54 | 0.570118 | 18.33 | 0.559139 | 19.12 | 0.505686 | 1.5 | 19.15 | 0.54758 | 1.8 | 19.08 | 0.538987 | 1.4 | 19.38 | 0.558317 | 2.1 | 19.45 | 0.516411 | 1.9 | 19.57 | 0.537826 | 2.4 |
| 21 | Vitamin D-binding protein OS=Homo sapiens GN=GC PE=1 SV=1 | VTDB_HUMAN | 53 kDa | TRUE | 0.0001 | 1.80 | 1.90 | 2.15 | 17.93 | 1.131656 | 18.03 | 1.003961 | 18.75 | 0.769959 | 1.9 | 18.75 | 0.760697 | 1.7 | 18.87 | 0.690192 | 2 | 18.79 | 0.656749 | 1.8 | 18.99 | 0.700568 | 2.3 | 18.95 | 0.675493 | 2 |
| 1653 | Complement component 1 Q subcomponent-binding protein, mitochondrial OS=Homo sapiens GN=C1QBP PE=1 SV=1 | C1QBP_HUMAN | 31 kDa |  | 0.0074 | 1.55 | 2.00 | 2.15 | 16.96 | 1.042596 | 17.02 | 0.800952 | 17.56 | 0.516514 | 1.5 | 17.69 | 0.503687 | 1.6 | 18.04 | 0.581242 | 2.1 | 17.94 | 0.49338 | 1.9 | 18.14 | 0.593745 | 2.3 | 18 | 0.504335 | 2 |
| 1464 | Myotrophin OS=Homo sapiens GN=MTPN PE=1 SV=2 | MTPN_HUMAN | 13 kDa |  | 0.0056 | 1.85 | 2.10 | 2.15 | 17.75 | 0.98465 | 17.86 | 0.822547 | 18.74 | 0.52126 | 1.9 | 18.75 | 0.529116 | 1.8 | 18.98 | 0.570658 | 2.4 | 18.74 | 0.544382 | 1.8 | 18.96 | 0.517987 | 2.3 | 18.85 | 0.510197 | 2 |
| 1307 | Histone H2A.V OS=Homo sapiens GN=H2AFV PE=1 SV=3 | H2AV_HUMAN | 14 kDa | TRUE | 0.026 | 1.20 | 1.60 | 2.15 | 17.63 | 1.018627 | 17.62 | 0.916815 | 17.85 | 0.699044 | 1.2 | 17.88 | 0.625931 | 1.2 | 18.42 | 0.595596 | 1.7 | 18.19 | 0.55286 | 1.5 | 18.76 | 0.622286 | 2.2 | 18.71 | 0.616198 | 2.1 |
| 1026 | Proteasome subunit beta type-2 OS=Homo sapiens GN=PSMB2 PE=1 SV=1 | PSB2_HUMAN | 23 kDa |  | 0.0015 | 1.95 | 1.65 | 2.15 | 18.07 | 1.242049 | 18.19 | 1.228576 | 19.08 | 0.584087 | 2 | 19.12 | 0.583396 | 1.9 | 18.76 | 0.517823 | 1.7 | 18.74 | 0.547283 | 1.6 | 19.01 | 0.529487 | 2.1 | 19.17 | 0.56862 | 2.2 |
| 300 | Heterogeneous nuclear ribonucleoproteins A2/B1 OS=Homo sapiens GN=HNRNPA2B1 PE=1 SV=2 | ROA2_HUMAN | 37 kDa | TRUE | 0.0001 | 1.55 | 1.70 | 2.15 | 18.1 | 0.959723 | 18.17 | 1.017605 | 18.73 | 0.540972 | 1.6 | 18.72 | 0.555874 | 1.5 | 18.98 | 0.547123 | 1.8 | 18.82 | 0.540321 | 1.6 | 19.26 | 0.594981 | 2.2 | 19.2 | 0.581416 | 2.1 |
| 1285 | Signal recognition particle 14 kDa protein OS=Homo sapiens GN=SRP14 PE=1 SV=2 | SRP14_HUMAN | 15 kDa |  | 0.0018 | 1.65 | 1.75 | 2.15 | 16.83 | 0.777742 | 16.89 | 0.676859 | 17.6 | 0.500056 | 1.7 | 17.61 | 0.492662 | 1.6 | 17.66 | 0.48376 | 1.8 | 17.67 | 0.523248 | 1.7 | 17.94 | 0.488885 | 2.2 | 17.96 | 0.488865 | 2.1 |
| 1348 | Serine/arginine-rich splicing factor 6 OS=Homo sapiens GN=SRSF6 PE=1 SV=2 | SRSF6_HUMAN | 40 kDa | TRUE | 0.0052 | 2.00 | 1.80 | 2.15 | 17.97 | 1.054692 | 17.98 | 1.017783 | 18.99 | 0.527712 | 2 | 18.98 | 0.524976 | 2 | 18.92 | 0.516862 | 1.9 | 18.83 | 0.524008 | 1.7 | 19.07 | 0.511261 | 2.1 | 19.13 | 0.513121 | 2.2 |
| 823 | Hydroxyacylglutathione hydrolase, mitochondrial OS=Homo sapiens GN=HAGH PE=1 SV=2 | GLO2_HUMAN | 34 kDa |  | 0.00027 | 1.65 | 1.85 | 2.15 | 15.76 | 0.589948 | 15.85 | 0.627884 | 16.5 | 0.625585 | 1.7 | 16.52 | 0.510224 | 1.6 | 16.66 | 0.52323 | 1.9 | 16.69 | 0.503611 | 1.8 | 16.85 | 0.506511 | 2.1 | 16.95 | 0.522051 | 2.2 |
| 720 | Calmodulin-like protein 3 OS=Homo sapiens GN=CALML3 PE=1 SV=2 | CALL3_HUMAN | 17 kDa | TRUE | 0.00019 | -1.05 | 2.00 | 2.15 | 16.77 | 0.660937 | 16.94 | 0.642119 | 16.72 | 0.557531 | 1 | 16.83 | 0.552775 | 0.9 | 17.91 | 0.569535 | 2.2 | 17.8 | 0.536966 | 1.8 | 17.9 | 0.592635 | 2.2 | 18.02 | 0.631253 | 2.1 |
| 1229 | SEC23-interacting protein OS=Homo sapiens GN=SEC23IP PE=1 SV=1 | S23IP_HUMAN | 111 kDa | TRUE | 0.0038 | 1.55 | 2.05 | 2.15 | 17.41 | 0.570024 | 17.38 | 0.665342 | 18.02 | 0.522189 | 1.5 | 18.09 | 0.510479 | 1.6 | 18.49 | 0.552249 | 2.1 | 18.43 | 0.493554 | 2 | 18.55 | 0.509864 | 2.2 | 18.49 | 0.526604 | 2.1 |
| 330 | Peroxiredoxin-6 OS=Homo sapiens GN=PRDX6 PE=1 SV=3 | PRDX6_HUMAN | 25 kDa | TRUE | 0.0001 | 2.10 | 2.35 | 2.15 | 17.7 | 1.056815 | 17.7 | 0.935472 | 18.74 | 0.604753 | 2.1 | 18.72 | 0.621838 | 2.1 | 18.95 | 0.578429 | 2.5 | 18.85 | 0.559703 | 2.2 | 18.78 | 0.53813 | 2.2 | 18.72 | 0.57626 | 2.1 |
| 938 | Uroporphyrinogen decarboxylase OS=Homo sapiens GN=UROD PE=1 SV=2 | DCUP_HUMAN | 41 kDa |  | 0.00048 | 1.55 | 1.45 | 2.20 | 16.72 | 0.558135 | 17.18 | 0.62774 | 17.57 | 0.52694 | 1.8 | 17.56 | 0.521959 | 1.3 | 17.31 | 0.654573 | 1.5 | 17.67 | 0.517952 | 1.4 | 17.72 | 0.503672 | 2 | 18.47 | 1.013454 | 2.4 |
| 277 | Histone H4 OS=Homo sapiens GN=HIST1H4A PE=1 SV=2 | H4_HUMAN | 11 kDa |  | 0.0001 | 1.40 | 1.60 | 2.20 | 17.95 | 1.34011 | 17.97 | 1.249472 | 18.4 | 0.648682 | 1.4 | 18.42 | 0.678995 | 1.4 | 18.52 | 0.610984 | 1.5 | 18.63 | 0.60884 | 1.7 | 18.96 | 0.69971 | 2.2 | 18.94 | 0.815938 | 2.2 |
| 859 | Eukaryotic translation initiation factor 3 subunit I OS=Homo sapiens GN=EIF3I PE=1 SV=1 | EIF3I_HUMAN | 37 kDa |  | 0.0001 | 1.75 | 1.65 | 2.20 | 18.36 | 1.144252 | 18.46 | 1.087665 | 19.19 | 0.541527 | 1.8 | 19.21 | 0.545274 | 1.7 | 19.2 | 0.568331 | 1.8 | 19.07 | 0.535944 | 1.5 | 19.52 | 0.576302 | 2.3 | 19.47 | 0.562001 | 2.1 |
| 398 | 40S ribosomal protein S2 OS=Homo sapiens GN=RPS2 PE=1 SV=2 | RS2_HUMAN | 31 kDa |  | 0.0001 | 1.80 | 1.70 | 2.20 | 18.04 | 0.951672 | 18.18 | 0.892158 | 18.91 | 0.54708 | 1.9 | 18.9 | 0.565887 | 1.7 | 18.93 | 0.561123 | 1.9 | 18.75 | 0.529328 | 1.5 | 19.3 | 0.636899 | 2.4 | 19.21 | 0.578492 | 2 |
| 454 | 60S acidic ribosomal protein P0 OS=Homo sapiens GN=RPLP0 PE=1 SV=1 | RLA0_HUMAN | 34 kDa |  | 0.0001 | 1.50 | 1.70 | 2.20 | 18.41 | 1.41741 | 18.58 | 1.239491 | 19.05 | 0.556216 | 1.6 | 19.02 | 0.535571 | 1.4 | 19.17 | 0.558422 | 1.9 | 19.03 | 0.556192 | 1.5 | 19.48 | 0.628331 | 2.4 | 19.5 | 0.590305 | 2 |
| 1715 | Peptidyl-prolyl cis-trans isomerase FKBP9 OS=Homo sapiens GN=FKBP9 PE=1 SV=2 | FKBP9_HUMAN | 63 kDa |  | 0.013 | 1.25 | 1.70 | 2.20 | 16.69 | 0.877313 | 16.71 | 0.873489 | 17.06 | 0.507189 | 1.3 | 16.95 | 0.509874 | 1.2 | 17.52 | 0.518621 | 1.8 | 17.35 | 0.524406 | 1.6 | 17.77 | 0.603964 | 2.3 | 17.66 | 0.624358 | 2.1 |
| 296 | Heterogeneous nuclear ribonucleoprotein U OS=Homo sapiens GN=HNRNPU PE=1 SV=6 | HNRPU_HUMAN | 91 kDa | TRUE | 0.0001 | 1.50 | 1.75 | 2.20 | 17.97 | 0.787534 | 17.97 | 0.771813 | 18.52 | 0.544149 | 1.5 | 18.52 | 0.552831 | 1.5 | 18.84 | 0.58159 | 1.9 | 18.64 | 0.523824 | 1.6 | 19.07 | 0.622124 | 2.2 | 19.08 | 0.633691 | 2.2 |
| 457 | V-type proton ATPase subunit E 1 OS=Homo sapiens GN=ATP6V1E1 PE=1 SV=1 | VATE1_HUMAN | 26 kDa |  | 0.0001 | 2.00 | 1.85 | 2.20 | 17.2 | 1.035692 | 17.44 | 1.083193 | 18.22 | 0.574456 | 2.1 | 18.3 | 0.576993 | 1.9 | 18.18 | 0.568564 | 2 | 18.17 | 0.546909 | 1.7 | 18.35 | 0.526786 | 2.3 | 18.38 | 0.573405 | 2.1 |
| 654 | 26S proteasome non-ATPase regulatory subunit 7 OS=Homo sapiens GN=PSMD7 PE=1 SV=2 | PSMD7_HUMAN | 37 kDa |  | 0.0001 | 1.75 | 1.85 | 2.20 | 17.82 | 0.890097 | 18.04 | 0.892009 | 18.74 | 0.535754 | 1.9 | 18.73 | 0.528616 | 1.6 | 18.87 | 0.562148 | 2 | 18.78 | 0.516924 | 1.7 | 18.98 | 0.54262 | 2.2 | 19.09 | 0.66863 | 2.2 |
| 173 | Hemoglobin subunit delta OS=Homo sapiens GN=HBD PE=1 SV=2 | HBD_HUMAN | 16 kDa | TRUE | 0.0001 | 1.50 | 1.90 | 2.20 | 18.29 | 1.154851 | 18.44 | 1.081939 | 18.89 | 0.563764 | 1.6 | 18.92 | 0.550394 | 1.4 | 19.14 | 0.575638 | 1.9 | 19.27 | 0.59593 | 1.9 | 19.43 | 0.584209 | 2.3 | 19.45 | 0.789679 | 2.1 |
| 359 | Elongation factor 1-delta OS=Homo sapiens GN=EEF1D PE=1 SV=5 | EF1D_HUMAN | 31 kDa | TRUE | 0.0001 | 2.10 | 1.95 | 2.20 | 17.18 | 0.958169 | 17.19 | 0.885345 | 18.21 | 0.775583 | 2 | 18.28 | 0.751787 | 2.2 | 18.07 | 0.554921 | 2 | 18.08 | 0.555786 | 1.9 | 18.3 | 0.569374 | 2.3 | 18.16 | 0.609974 | 2.1 |
| 1212 | Serine/threonine-protein kinase 24 OS=Homo sapiens GN=STK24 PE=1 SV=1 | STK24_HUMAN | 49 kDa | TRUE | 0.011 | 1.55 | 1.95 | 2.20 | 17.69 | 0.624281 | 17.56 | 0.506685 | 18.22 | 0.498586 | 1.5 | 18.23 | 0.504675 | 1.6 | 18.58 | 0.568748 | 1.9 | 18.52 | 0.493651 | 2 | 18.67 | 0.528696 | 2 | 18.84 | 0.500856 | 2.4 |
| 577 | LIM and SH3 domain protein 1 OS=Homo sapiens GN=LASP1 PE=1 SV=2 | LASP1_HUMAN | 30 kDa | TRUE | 0.0001 | 1.40 | 1.95 | 2.20 | 18.23 | 0.782116 | 18.31 | 0.8597 | 18.75 | 0.709098 | 1.4 | 18.75 | 0.5706 | 1.4 | 19.28 | 0.627007 | 2.1 | 19.09 | 0.582351 | 1.8 | 19.35 | 0.5952 | 2.2 | 19.42 | 0.623317 | 2.2 |
| 1366 | Protein LZIC OS=Homo sapiens GN=LZIC PE=1 SV=1 | LZIC_HUMAN | 21 kDa |  | 0.012 | 1.55 | 2.00 | 2.20 | 16.91 | 1.311381 | 16.95 | 0.963252 | 17.55 | 0.514693 | 1.6 | 17.53 | 0.537751 | 1.5 | 17.77 | 0.596651 | 2 | 17.89 | 0.514181 | 2 | 18.06 | 0.506031 | 2.2 | 17.98 | 0.537946 | 2.2 |
| 22 | Antithrombin-III OS=Homo sapiens GN=SERPINC1 PE=1 SV=1 | ANT3_HUMAN | 53 kDa | TRUE | 0.0001 | 2.20 | 2.05 | 2.20 | 16.43 | 1.00046 | 16.54 | 0.927427 | 17.57 | 0.745232 | 2.2 | 17.67 | 0.749244 | 2.2 | 17.48 | 0.67669 | 2.1 | 17.48 | 0.654157 | 2 | 17.59 | 0.604888 | 2.3 | 17.61 | 0.645126 | 2.1 |
| 687 | Sulfotransferase 1A1 OS=Homo sapiens GN=SULT1A1 PE=1 SV=3 | ST1A1_HUMAN | 34 kDa | TRUE | 0.0001 | 2.05 | 2.05 | 2.20 | 17.77 | 1.008812 | 17.84 | 1.010035 | 18.73 | 0.537105 | 2.1 | 18.75 | 0.535607 | 2 | 18.8 | 0.532853 | 2.1 | 18.76 | 0.538794 | 2 | 18.77 | 0.518228 | 2.1 | 18.87 | 0.601577 | 2.3 |
| 810 | Alcohol dehydrogenase class-3 OS=Homo sapiens GN=ADH5 PE=1 SV=4 | ADHX_HUMAN | 40 kDa |  | 0.0001 | 1.80 | 2.15 | 2.20 | 17.87 | 1.054063 | 17.95 | 1.211001 | 18.71 | 0.523043 | 1.8 | 18.77 | 0.522387 | 1.8 | 19.04 | 0.540352 | 2.3 | 18.88 | 0.519254 | 2 | 19.01 | 0.537516 | 2.3 | 18.97 | 0.523268 | 2.1 |
| 246 | High mobility group protein B2 OS=Homo sapiens GN=HMGB2 PE=1 SV=2 | HMGB2_HUMAN | 24 kDa | TRUE | 0.0012 | 1.75 | 2.45 | 2.20 | 17.51 | 1.004872 | 17.6 | 0.899255 | 18.4 | 0.724153 | 1.8 | 18.38 | 0.746067 | 1.7 | 18.91 | 0.659527 | 2.9 | 18.53 | 0.559505 | 2 | 18.8 | 0.611829 | 2.6 | 18.42 | 0.698935 | 1.8 |
| 964 | 60S ribosomal protein L23a OS=Homo sapiens GN=RPL23A PE=1 SV=1 | RL23A_HUMAN | 18 kDa |  | 0.0001 | 1.20 | 1.40 | 2.25 | 17.66 | 0.606118 | 17.73 | 0.646372 | 17.95 | 0.527483 | 1.2 | 17.98 | 0.528567 | 1.2 | 18.24 | 0.52933 | 1.5 | 18.05 | 0.517147 | 1.3 | 18.83 | 0.572178 | 2.3 | 18.81 | 0.567527 | 2.2 |
| 1080 | S-adenosylmethionine synthase isoform type-2 OS=Homo sapiens GN=MAT2A PE=1 SV=1 | METK2_HUMAN | 44 kDa | TRUE | 0.0097 | 1.60 | 1.75 | 2.25 | 18.09 | 1.513056 | 18.16 | 1.264407 | 18.85 | 0.504267 | 1.6 | 18.81 | 0.519551 | 1.6 | 19 | 0.507632 | 1.8 | 18.86 | 0.528905 | 1.7 | 19.33 | 0.50795 | 2.2 | 19.27 | 0.563819 | 2.3 |
| 680 | 60S ribosomal protein L15 OS=Homo sapiens GN=RPL15 PE=1 SV=2 | RL15_HUMAN | 24 kDa |  | 0.0001 | 1.55 | 1.85 | 2.25 | 17.82 | 0.817994 | 17.97 | 0.794038 | 18.42 | 0.598544 | 1.6 | 18.51 | 0.620269 | 1.5 | 18.81 | 0.651311 | 2 | 18.68 | 0.587137 | 1.7 | 19.14 | 0.664125 | 2.4 | 19.07 | 0.677778 | 2.1 |
| 1329 | Eukaryotic translation initiation factor 3 subunit F OS=Homo sapiens GN=EIF3F PE=1 SV=1 | EIF3F_HUMAN | 38 kDa |  | 0.00017 | 1.95 | 1.90 | 2.25 | 17.12 | 1.024485 | 17.24 | 0.855957 | 18.12 | 0.516743 | 2.1 | 18.04 | 0.497545 | 1.8 | 18.04 | 0.49793 | 1.9 | 18.09 | 0.549356 | 1.9 | 18.3 | 0.501716 | 2.2 | 18.46 | 0.523715 | 2.3 |
| 961 | DNA repair protein RAD50 OS=Homo sapiens GN=RAD50 PE=1 SV=1 | RAD50_HUMAN | 154 kDa | TRUE | 0.0022 | 1.75 | 1.90 | 2.25 | 16.61 | 0.880688 | 16.65 | 0.652524 | 17.45 | 0.49402 | 1.7 | 17.5 | 0.507318 | 1.8 | 17.53 | 0.488844 | 1.9 | 17.54 | 0.525723 | 1.9 | 17.79 | 0.537526 | 2.2 | 17.84 | 0.552592 | 2.3 |
| 385 | Phosphatidylinositol 5-phosphate 4-kinase type-2 alpha OS=Homo sapiens GN=PIP4K2A PE=1 SV=2 | PI42A_HUMAN | 46 kDa | TRUE | 0.0001 | 2.10 | 2.00 | 2.25 | 17.3 | 1.072269 | 17.43 | 0.963795 | 18.41 | 0.511102 | 2.2 | 18.41 | 0.520988 | 2 | 18.35 | 0.560957 | 2.1 | 18.28 | 0.553618 | 1.9 | 18.53 | 0.52863 | 2.3 | 18.52 | 0.574385 | 2.2 |
| 192 | Proteasome subunit alpha type-7 OS=Homo sapiens GN=PSMA7 PE=1 SV=1 | PSA7_HUMAN | 28 kDa | TRUE | 0.0001 | 1.85 | 2.05 | 2.25 | 17.94 | 0.907652 | 18.02 | 0.95918 | 18.85 | 0.656281 | 1.9 | 18.88 | 0.628481 | 1.8 | 19.07 | 0.598342 | 2.2 | 18.94 | 0.540094 | 1.9 | 19.1 | 0.572876 | 2.3 | 19.12 | 0.615296 | 2.2 |
| 410 | Microtubule-associated protein RP/EB family member 1 OS=Homo sapiens GN=MAPRE1 PE=1 SV=3 | MARE1_HUMAN | 30 kDa | TRUE | 0.0001 | 1.60 | 2.30 | 2.25 | 17.91 | 1.000733 | 18.1 | 0.90618 | 18.71 | 0.640644 | 1.7 | 18.71 | 0.668679 | 1.5 | 19.22 | 0.641626 | 2.6 | 19.03 | 0.574894 | 2 | 19.13 | 0.585507 | 2.4 | 19.16 | 0.601314 | 2.1 |
| 1486 | V-type proton ATPase subunit G 1 OS=Homo sapiens GN=ATP6V1G1 PE=1 SV=3 | VATG1_HUMAN | 14 kDa |  | 0.011 | 1.25 | 1.55 | 2.30 | 17.74 | 0.634078 | 18.13 | 0.659651 | 18.23 | 0.523104 | 1.4 | 18.22 | 0.529545 | 1.1 | 18.49 | 0.52188 | 1.7 | 18.6 | 0.509142 | 1.4 | 18.94 | 0.617386 | 2.4 | 19.24 | 0.619395 | 2.2 |
| 1408 | UPF0568 protein C14orf166 OS=Homo sapiens GN=C14orf166 PE=1 SV=1 | CN166_HUMAN | 28 kDa |  | 0.0022 | 1.50 | 1.70 | 2.30 | 17.23 | 0.752494 | 17.23 | 0.638089 | 17.8 | 0.496519 | 1.4 | 17.9 | 0.485705 | 1.6 | 17.98 | 0.506416 | 1.7 | 17.95 | 0.498645 | 1.7 | 18.58 | 0.691239 | 2.7 | 18.23 | 0.662493 | 1.9 |
| 81 | Tubulin beta chain OS=Homo sapiens GN=TUBB PE=1 SV=2 | TBB5_HUMAN | 50 kDa | TRUE | 0.00031 | 1.55 | 1.75 | 2.30 | 18.08 | 1.160423 | 18.07 | 0.849939 | 18.69 | 0.579359 | 1.6 | 18.67 | 0.55946 | 1.5 | 18.79 | 0.562191 | 1.7 | 18.87 | 0.550985 | 1.8 | 19.25 | 0.610534 | 2.4 | 19.21 | 0.609266 | 2.2 |
| 63 | 14-3-3 protein zeta/delta OS=Homo sapiens GN=YWHAZ PE=1 SV=1 | 1433Z_HUMAN | 28 kDa | TRUE | 0.0001 | 1.70 | 2.10 | 2.30 | 17.06 | 1.050832 | 17.16 | 0.940746 | 17.87 | 0.596331 | 1.7 | 17.94 | 0.597279 | 1.7 | 18.15 | 0.616101 | 2.3 | 18.03 | 0.611254 | 1.9 | 18.26 | 0.603998 | 2.4 | 18.22 | 0.600058 | 2.2 |
| 1405 | ADP-ribosylation factor-like protein 8B OS=Homo sapiens GN=ARL8B PE=1 SV=1 | ARL8B_HUMAN | 22 kDa | TRUE | 0.0001 | 1.75 | 2.10 | 2.30 | 17.87 | 0.607702 | 17.98 | 0.618105 | 18.7 | 0.534137 | 1.8 | 18.78 | 0.515469 | 1.7 | 18.99 | 0.513918 | 2.2 | 18.96 | 0.53481 | 2 | 19.11 | 0.516418 | 2.4 | 19.16 | 0.515103 | 2.2 |
| 1031 | Ras-related protein Rab-10 OS=Homo sapiens GN=RAB10 PE=1 SV=1 | RAB10_HUMAN | 23 kDa | TRUE | 0.00015 | 1.95 | 2.30 | 2.30 | 17.9 | 0.984072 | 17.86 | 0.769069 | 18.87 | 0.525996 | 2.1 | 18.72 | 0.51638 | 1.8 | 19.18 | 0.5765 | 2.7 | 18.8 | 0.51534 | 1.9 | 19.03 | 0.516797 | 2.3 | 19.05 | 0.52355 | 2.3 |
| 1349 | Signal transducing adapter molecule 1 OS=Homo sapiens GN=STAM PE=1 SV=3 | STAM1_HUMAN | 59 kDa |  | 0.013 | 2.50 | 2.35 | 2.30 | 16.81 | 0.865687 | 16.79 | 0.871117 | 18.2 | 0.567667 | 2.5 | 18.09 | 0.521337 | 2.5 | 18.17 | 0.550401 | 2.5 | 17.98 | 0.49813 | 2.2 | 18.03 | 0.492384 | 2.3 | 17.98 | 0.540499 | 2.3 |
| 1813 | Tropomodulin-1 OS=Homo sapiens GN=TMOD1 PE=1 SV=1 | TMOD1_HUMAN | 41 kDa | TRUE | 0.011 | 4.25 | 3.00 | 2.30 | 14.14 | 0.526834 | 14.13 | 0.536289 | 16.25 | 0.738185 | 4.3 | 16.19 | 0.784703 | 4.2 | 15.7 | 0.478026 | 2.9 | 15.76 | 0.482389 | 3.1 | 15.29 | 0.492703 | 2.2 | 15.42 | 0.482586 | 2.4 |
| 603 | 60S ribosomal protein L6 OS=Homo sapiens GN=RPL6 PE=1 SV=3 | RL6_HUMAN | 33 kDa | TRUE | 0.0001 | 1.95 | 1.80 | 2.35 | 17.64 | 0.813499 | 17.69 | 0.692711 | 18.63 | 0.617447 | 1.9 | 18.68 | 0.651779 | 2 | 18.47 | 0.589051 | 1.8 | 18.5 | 0.536785 | 1.8 | 18.91 | 0.58696 | 2.4 | 18.9 | 0.651865 | 2.3 |
| 354 | 40S ribosomal protein S19 OS=Homo sapiens GN=RPS19 PE=1 SV=2 | RS19_HUMAN | 16 kDa |  | 0.0001 | 1.35 | 1.80 | 2.35 | 17.8 | 0.824161 | 17.7 | 0.747005 | 18.17 | 0.614819 | 1.3 | 18.14 | 0.5991 | 1.4 | 18.64 | 0.59808 | 1.8 | 18.5 | 0.575698 | 1.8 | 18.97 | 0.569662 | 2.3 | 18.93 | 0.600259 | 2.4 |
| 406 | Coronin-1C OS=Homo sapiens GN=CORO1C PE=1 SV=1 | COR1C_HUMAN | 53 kDa | TRUE | 0.0001 | 1.55 | 1.95 | 2.35 | 17.75 | 0.848707 | 17.85 | 0.754937 | 18.39 | 0.54027 | 1.6 | 18.42 | 0.539255 | 1.5 | 18.77 | 0.528459 | 2.1 | 18.65 | 0.548542 | 1.8 | 18.95 | 0.563631 | 2.4 | 18.99 | 0.601601 | 2.3 |
| 663 | ATPase ASNA1 OS=Homo sapiens GN=ASNA1 PE=1 SV=2 | ASNA_HUMAN | 39 kDa |  | 0.0001 | 2.20 | 2.10 | 2.35 | 17.87 | 1.185016 | 18 | 1.058873 | 18.99 | 0.593672 | 2.3 | 19 | 0.588897 | 2.1 | 19.06 | 0.533252 | 2.2 | 18.97 | 0.535456 | 2 | 19.16 | 0.542217 | 2.4 | 19.19 | 0.559908 | 2.3 |
| 462 | Calpain small subunit 1 OS=Homo sapiens GN=CAPNS1 PE=1 SV=1 | CPNS1_HUMAN | 28 kDa |  | 0.0001 | 1.75 | 2.10 | 2.35 | 16.97 | 0.814732 | 17.13 | 0.867729 | 17.9 | 0.604931 | 1.9 | 17.87 | 0.640866 | 1.6 | 18.04 | 0.576748 | 2.2 | 18.04 | 0.574897 | 2 | 18.12 | 0.531141 | 2.3 | 18.17 | 0.698726 | 2.4 |
| 371 | Ras-related protein Rab-1B OS=Homo sapiens GN=RAB1B PE=1 SV=1 | RAB1B_HUMAN | 22 kDa | TRUE | 0.0013 | 2.10 | 2.35 | 2.35 | 17.69 | 0.809474 | 17.66 | 0.709913 | 18.67 | 0.550903 | 2.1 | 18.7 | 0.567871 | 2.1 | 19.03 | 0.56155 | 2.6 | 18.71 | 0.513567 | 2.1 | 18.92 | 0.571024 | 2.4 | 18.84 | 0.572784 | 2.3 |
| 97 | Protein disulfide-isomerase OS=Homo sapiens GN=P4HB PE=1 SV=3 | PDIA1_HUMAN | 57 kDa |  | 0.0001 | 1.80 | 2.50 | 2.35 | 17.52 | 0.956388 | 17.52 | 0.913876 | 18.3 | 0.589863 | 1.8 | 18.36 | 0.574406 | 1.8 | 18.87 | 0.635915 | 2.7 | 18.69 | 0.611973 | 2.3 | 18.71 | 0.572781 | 2.4 | 18.69 | 0.599778 | 2.3 |
| 740 | 40S ribosomal protein S11 OS=Homo sapiens GN=RPS11 PE=1 SV=3 | RS11_HUMAN | 18 kDa |  | 0.0001 | 1.55 | 1.55 | 2.35 | 18.16 | 0.807183 | 18.23 | 0.739665 | 18.87 | 0.667607 | 1.6 | 18.9 | 0.634032 | 1.5 | 18.83 | 0.558703 | 1.6 | 18.78 | 0.548199 | 1.5 | 19.44 | 0.581726 | 2.5 | 19.33 | 0.612642 | 2.2 |
| 1360 | Small nuclear ribonucleoprotein-associated proteins B and B' OS=Homo sapiens GN=SNRPB PE=1 SV=2 | RSMB_HUMAN | 25 kDa |  | 0.0022 | 2.30 | 1.90 | 2.35 | 16.56 | 1.122801 | 16.24 | 0.78284 | 17.55 | 0.488655 | 2.1 | 17.55 | 0.497641 | 2.5 | 17.43 | 0.496471 | 1.9 | 17.24 | 0.492208 | 1.9 | 17.65 | 0.52306 | 2.2 | 17.58 | 0.546361 | 2.5 |
| 585 | Carbonic anhydrase 2 OS=Homo sapiens GN=CA2 PE=1 SV=2 | CAH2_HUMAN | 29 kDa |  | 0.0001 | 1.75 | 1.95 | 2.35 | 17.13 | 0.954687 | 17.28 | 0.958441 | 17.9 | 0.571286 | 1.8 | 17.93 | 0.563566 | 1.7 | 18.29 | 0.587022 | 2.2 | 18.08 | 0.554695 | 1.7 | 18.45 | 0.573196 | 2.5 | 18.45 | 0.594127 | 2.2 |
| 548 | Proteasome subunit beta type-3 OS=Homo sapiens GN=PSMB3 PE=1 SV=2 | PSB3_HUMAN | 23 kDa |  | 0.0001 | 2.05 | 2.15 | 2.35 | 17.78 | 1.081682 | 17.65 | 1.224949 | 18.88 | 0.608176 | 2 | 18.83 | 0.618699 | 2.1 | 18.85 | 0.544077 | 2.1 | 18.72 | 0.551058 | 2.2 | 18.87 | 0.548782 | 2.2 | 18.86 | 0.64329 | 2.5 |
| 1335 | Nucleosome assembly protein 1-like 1 OS=Homo sapiens GN=NAP1L1 PE=1 SV=1 | NP1L1_HUMAN | 45 kDa | TRUE | 0.0067 | 2.20 | 2.20 | 2.35 | 17.4 | 0.6339 | 17.23 | 0.654575 | 18.44 | 0.501343 | 2.1 | 18.42 | 0.501873 | 2.3 | 18.46 | 0.544238 | 2.1 | 18.45 | 0.525967 | 2.3 | 18.42 | 0.506205 | 2 | 18.64 | 0.514242 | 2.7 |
| 1740 | Alcohol dehydrogenase [NADP(+)] OS=Homo sapiens GN=AKR1A1 PE=1 SV=3 | AK1A1_HUMAN | 37 kDa |  | 0.011 | 1.40 | 2.45 | 2.35 | 15.58 | 0.556906 | 15.61 | 0.501676 | 16.02 | 0.488489 | 1.4 | 16.05 | 0.488976 | 1.4 | 16.88 | 0.486672 | 2.4 | 16.94 | 0.519277 | 2.5 | 16.9 | 0.495388 | 2.5 | 16.77 | 0.500595 | 2.2 |
| 1626 | 60S ribosomal protein L23 OS=Homo sapiens GN=RPL23 PE=1 SV=1 | RL23_HUMAN | 15 kDa |  | 0.0056 | 1.65 | 1.70 | 2.40 | 17.5 | 0.893123 | 17.71 | 1.059336 | 18.32 | 0.543141 | 1.8 | 18.33 | 0.530005 | 1.5 | 18.35 | 0.498871 | 1.8 | 18.37 | 0.538523 | 1.6 | 18.83 | 0.539941 | 2.5 | 18.92 | 0.523354 | 2.3 |
| 1788 | Zinc finger protein ZPR1 OS=Homo sapiens GN=ZPR1 PE=1 SV=1 | ZPR1_HUMAN | 51 kDa |  | 0.013 | 1.50 | 1.90 | 2.40 | 16.94 | 1.09478 | 16.92 | 1.167266 | 17.49 | 0.494172 | 1.4 | 17.58 | 0.491047 | 1.6 | 17.89 | 0.521126 | 1.9 | 17.85 | 0.495294 | 1.9 | 18.28 | 0.549396 | 2.5 | 18.12 | 0.492997 | 2.3 |
| 314 | Nucleophosmin OS=Homo sapiens GN=NPM1 PE=1 SV=2 | NPM_HUMAN | 33 kDa |  | 0.0001 | 1.55 | 1.95 | 2.40 | 18.01 | 0.963345 | 18.05 | 0.850106 | 18.63 | 0.550392 | 1.5 | 18.71 | 0.575451 | 1.6 | 19.02 | 0.591479 | 2 | 18.91 | 0.560526 | 1.9 | 19.24 | 0.618385 | 2.4 | 19.29 | 0.605971 | 2.4 |
| 152 | Hsc70-interacting protein OS=Homo sapiens GN=ST13 PE=1 SV=2 | F10A1_HUMAN | 41 kDa |  | 0.0001 | 2.00 | 2.15 | 2.40 | 17.79 | 1.05138 | 17.82 | 1.02185 | 18.7 | 0.537577 | 2 | 18.75 | 0.544621 | 2 | 18.98 | 0.554241 | 2.3 | 18.88 | 0.598845 | 2 | 19.01 | 0.538578 | 2.4 | 19.04 | 0.583626 | 2.4 |
| 1077 | U6 snRNA-associated Sm-like protein LSm4 OS=Homo sapiens GN=LSM4 PE=1 SV=1 | LSM4_HUMAN | 15 kDa |  | 0.014 | 1.60 | 2.25 | 2.40 | 18.14 | 0.740591 | 18.11 | 0.77153 | 18.71 | 0.799248 | 1.5 | 18.84 | 0.776605 | 1.7 | 19.31 | 0.670669 | 2.5 | 19.04 | 0.581053 | 2 | 19.33 | 0.585156 | 2.4 | 19.3 | 0.593501 | 2.4 |
| 1488 | Macrophage migration inhibitory factor OS=Homo sapiens GN=MIF PE=1 SV=4 | MIF_HUMAN | 12 kDa |  | 0.00094 | 1.55 | 2.35 | 2.40 | 17.81 | 0.755921 | 18.05 | 0.840755 | 18.48 | 0.504151 | 1.6 | 18.53 | 0.506775 | 1.5 | 19.15 | 0.507722 | 2.6 | 19.08 | 0.506045 | 2.1 | 19.03 | 0.524008 | 2.4 | 19.22 | 0.5509 | 2.4 |
| 639 | Adenine phosphoribosyltransferase OS=Homo sapiens GN=APRT PE=1 SV=2 | APT_HUMAN | 20 kDa |  | 0.0001 | 1.70 | 2.45 | 2.40 | 18.37 | 1.010953 | 18.41 | 0.939501 | 19.23 | 0.611042 | 1.7 | 19.24 | 0.622814 | 1.7 | 19.6 | 0.63087 | 2.5 | 19.64 | 0.560073 | 2.4 | 19.52 | 0.562259 | 2.3 | 19.64 | 0.664905 | 2.5 |
| 289 | Peptidyl-prolyl cis-trans isomerase A OS=Homo sapiens GN=PPIA PE=1 SV=2 | PPIA_HUMAN | 18 kDa | TRUE | 0.0001 | 1.10 | 2.45 | 2.40 | 18.18 | 0.89516 | 18.1 | 0.841139 | 18.36 | 0.678089 | 1.1 | 18.37 | 0.721539 | 1.1 | 19.47 | 0.754548 | 2.6 | 19.23 | 0.64963 | 2.3 | 19.4 | 0.62934 | 2.5 | 19.3 | 0.609792 | 2.3 |
| 64 | 6-phosphogluconate dehydrogenase, decarboxylating OS=Homo sapiens GN=PGD PE=1 SV=3 | 6PGD_HUMAN | 53 kDa |  | 0.0001 | 2.25 | 2.75 | 2.40 | 17.48 | 1.206875 | 17.55 | 1.156388 | 18.67 | 0.706408 | 2.2 | 18.7 | 0.670229 | 2.3 | 18.95 | 0.638579 | 2.9 | 18.85 | 0.575185 | 2.6 | 18.75 | 0.567089 | 2.5 | 18.71 | 0.62371 | 2.3 |
| 836 | 40S ribosomal protein S7 OS=Homo sapiens GN=RPS7 PE=1 SV=1 | RS7_HUMAN | 22 kDa |  | 0.0001 | 1.75 | 1.65 | 2.40 | 18.48 | 0.941344 | 18.12 | 1.020422 | 19.05 | 0.51306 | 1.5 | 19.05 | 0.519714 | 2 | 19.03 | 0.5729 | 1.5 | 18.98 | 0.547149 | 1.8 | 19.51 | 0.555812 | 2.1 | 19.48 | 0.575472 | 2.7 |
| 1268 | Plasminogen activator inhibitor 1 OS=Homo sapiens GN=SERPINE1 PE=1 SV=1 | PAI1_HUMAN | 45 kDa |  | 0.029 | 1.55 | 1.65 | 2.40 | 17.11 | 0.864559 | 17.13 | 0.913084 | 17.78 | 0.494107 | 1.6 | 17.68 | 0.498337 | 1.5 | 17.82 | 0.516377 | 1.6 | 17.89 | 0.499044 | 1.7 | 18.14 | 0.563298 | 2.1 | 18.35 | 0.666099 | 2.7 |
| 1697 | BUB3-interacting and GLEBS motif-containing protein ZNF207 OS=Homo sapiens GN=ZNF207 PE=1 SV=1 | ZN207_HUMAN | 51 kDa |  | 0.019 | 1.20 | 2.10 | 2.40 | 17.58 | 0.495875 | 17.64 | 0.501913 | 17.79 | 0.52779 | 1.2 | 17.89 | 0.531156 | 1.2 | 18.72 | 0.666452 | 2.2 | 18.62 | 0.499245 | 2 | 18.69 | 0.526847 | 2.2 | 19 | 0.725036 | 2.6 |
| 682 | 60S ribosomal protein L7a OS=Homo sapiens GN=RPL7A PE=1 SV=2 | RL7A_HUMAN | 30 kDa |  | 0.0001 | 1.60 | 1.65 | 2.45 | 17.69 | 0.909414 | 17.77 | 0.803771 | 18.32 | 0.553917 | 1.6 | 18.38 | 0.516749 | 1.6 | 18.41 | 0.565614 | 1.6 | 18.51 | 0.551614 | 1.7 | 18.91 | 0.604256 | 2.3 | 19.07 | 0.649493 | 2.6 |
| 940 | Probable ATP-dependent RNA helicase DDX5 OS=Homo sapiens GN=DDX5 PE=1 SV=1 | DDX5_HUMAN | 69 kDa | TRUE | 0.0001 | 1.55 | 1.70 | 2.45 | 16.71 | 0.814991 | 16.67 | 0.824547 | 17.28 | 0.516337 | 1.5 | 17.35 | 0.50943 | 1.6 | 17.44 | 0.506184 | 1.6 | 17.49 | 0.50373 | 1.8 | 17.89 | 0.521208 | 2.3 | 17.98 | 0.549062 | 2.6 |
| 923 | 60S ribosomal protein L30 OS=Homo sapiens GN=RPL30 PE=1 SV=2 | RL30_HUMAN | 13 kDa |  | 0.001 | 1.55 | 1.80 | 2.45 | 17.45 | 0.763335 | 17.31 | 0.830145 | 18.01 | 0.521461 | 1.5 | 18.01 | 0.500876 | 1.6 | 18.21 | 0.513525 | 1.7 | 18.19 | 0.558476 | 1.9 | 18.6 | 0.53203 | 2.3 | 18.69 | 0.573918 | 2.6 |
| 696 | Elongation factor 1-beta OS=Homo sapiens GN=EEF1B2 PE=1 SV=3 | EF1B_HUMAN | 25 kDa | TRUE | 0.0001 | 2.40 | 2.20 | 2.45 | 18 | 1.219139 | 18.05 | 1.150267 | 19.3 | 0.655438 | 2.5 | 19.14 | 0.590499 | 2.3 | 19.36 | 0.686135 | 2.5 | 18.95 | 0.542361 | 1.9 | 19.24 | 0.567222 | 2.5 | 19.24 | 0.56729 | 2.4 |
| 948 | Core histone macro-H2A.1 OS=Homo sapiens GN=H2AFY PE=1 SV=4 | H2AY_HUMAN | 40 kDa |  | 0.0004 | 1.90 | 2.20 | 2.45 | 17.03 | 1.031118 | 17.27 | 1.085406 | 18.03 | 0.519739 | 2 | 18.12 | 0.522274 | 1.8 | 18.21 | 0.647947 | 2.3 | 18.27 | 0.571814 | 2.1 | 18.38 | 0.580012 | 2.5 | 18.64 | 0.727736 | 2.4 |
| 123 | Ferritin heavy chain OS=Homo sapiens GN=FTH1 PE=1 SV=2 | FRIH_HUMAN | 21 kDa |  | 0.0001 | 1.95 | 1.65 | 2.50 | 17.38 | 1.117335 | 17.4 | 0.894325 | 18.19 | 0.683969 | 2 | 18.22 | 0.661781 | 1.9 | 18.08 | 0.658119 | 1.7 | 18.03 | 0.598755 | 1.6 | 18.54 | 0.678919 | 2.4 | 18.67 | 0.703766 | 2.6 |
| 1639 | Branched-chain-amino-acid aminotransferase, cytosolic OS=Homo sapiens GN=BCAT1 PE=1 SV=3 | BCAT1_HUMAN | 43 kDa |  | 0.00044 | 1.35 | 1.80 | 2.50 | 16.53 | 0.540279 | 16.28 | 0.591533 | 16.8 | 0.493716 | 1.2 | 16.86 | 0.489623 | 1.5 | 17.16 | 0.490312 | 1.6 | 17.3 | 0.50631 | 2 | 17.69 | 0.496937 | 2.2 | 17.79 | 0.55446 | 2.8 |
| 1592 | UDP-N-acetylhexosamine pyrophosphorylase-like protein 1 OS=Homo sapiens GN=UAP1L1 PE=1 SV=2 | UAP1L_HUMAN | 57 kDa |  | 0.011 | 1.60 | 1.85 | 2.50 | 16.2 | 0.597996 | 16.4 | 0.497288 | 17.01 | 0.484192 | 1.8 | 16.88 | 0.480018 | 1.4 | 17.12 | 0.497042 | 1.9 | 17.24 | 0.506447 | 1.8 | 17.58 | 0.567127 | 2.6 | 17.67 | 0.490727 | 2.4 |
| 922 | 60S ribosomal protein L17 OS=Homo sapiens GN=RPL17 PE=1 SV=3 | RL17_HUMAN | 21 kDa |  | 0.00011 | 1.75 | 1.95 | 2.50 | 17.18 | 0.736422 | 17.11 | 0.748488 | 17.94 | 0.52271 | 1.7 | 17.94 | 0.532384 | 1.8 | 18.13 | 0.553503 | 2 | 18.06 | 0.505084 | 1.9 | 18.48 | 0.515355 | 2.5 | 18.45 | 0.531954 | 2.5 |
| 101 | Elongation factor 1-alpha 1 OS=Homo sapiens GN=EEF1A1 PE=1 SV=1 | EF1A1_HUMAN | 50 kDa |  | 0.0001 | 1.75 | 1.95 | 2.50 | 18.17 | 0.995226 | 18.34 | 0.940456 | 18.99 | 0.682028 | 1.8 | 19.05 | 0.704708 | 1.7 | 19.21 | 0.594615 | 2.1 | 19.13 | 0.585118 | 1.8 | 19.52 | 0.637983 | 2.7 | 19.51 | 0.689326 | 2.3 |
| 1362 | U6 snRNA-associated Sm-like protein LSm6 OS=Homo sapiens GN=LSM6 PE=1 SV=1 | LSM6_HUMAN | 9 kDa |  | 0.022 | 1.65 | 2.15 | 2.50 | 16.4 | 0.92213 | 16.6 | 0.868418 | 17.3 | 0.552786 | 1.8 | 17.25 | 0.587427 | 1.5 | 17.61 | 0.751525 | 2.6 | 17.27 | 0.59123 | 1.7 | 17.65 | 0.707444 | 2.7 | 17.67 | 0.670273 | 2.3 |
| 167 | X-ray repair cross-complementing protein 6 OS=Homo sapiens GN=XRCC6 PE=1 SV=2 | XRCC6_HUMAN | 70 kDa | TRUE | 0.0001 | 1.95 | 2.25 | 2.50 | 17.23 | 1.031546 | 17.17 | 0.984232 | 18.19 | 0.578118 | 1.9 | 18.21 | 0.583567 | 2 | 18.42 | 0.582211 | 2.4 | 18.18 | 0.573664 | 2.1 | 18.51 | 0.584716 | 2.5 | 18.44 | 0.588986 | 2.5 |
| 86 | 14-3-3 protein sigma OS=Homo sapiens GN=SFN PE=1 SV=1 | 1433S_HUMAN | 28 kDa | TRUE | 0.0001 | 1.15 | 2.30 | 2.50 | 17.67 | 0.875329 | 17.87 | 0.803219 | 17.87 | 0.671696 | 1.2 | 17.94 | 0.637563 | 1.1 | 18.98 | 0.693551 | 2.6 | 18.83 | 0.604458 | 2 | 19.02 | 0.604672 | 2.6 | 19.05 | 0.606589 | 2.4 |
| 527 | Protein S100-A12 OS=Homo sapiens GN=S100A12 PE=1 SV=2 | S10AC_HUMAN | 11 kDa |  | 0.0001 | 1.85 | 2.35 | 2.50 | 17.94 | 0.898018 | 18.09 | 0.913685 | 18.88 | 0.580764 | 2 | 18.89 | 0.591743 | 1.7 | 19.22 | 0.573278 | 2.5 | 19.16 | 0.64397 | 2.2 | 19.25 | 0.568678 | 2.5 | 19.46 | 0.63696 | 2.5 |
| 14 | Hemoglobin subunit alpha OS=Homo sapiens GN=HBA1 PE=1 SV=2 | HBA_HUMAN | 15 kDa | TRUE | 0.0001 | 2.45 | 2.25 | 2.55 | 17.62 | 1.208224 | 17.76 | 1.116157 | 18.8 | 0.916934 | 2.7 | 18.73 | 0.839371 | 2.2 | 18.9 | 0.839126 | 2.4 | 18.79 | 0.664231 | 2.1 | 18.97 | 0.696783 | 2.7 | 18.94 | 0.72793 | 2.4 |
| 339 | Ras-related C3 botulinum toxin substrate 2 OS=Homo sapiens GN=RAC2 PE=1 SV=1 | RAC2_HUMAN | 21 kDa | TRUE | 0.0001 | 1.95 | 2.70 | 2.55 | 17.96 | 1.051083 | 17.93 | 0.865719 | 18.85 | 0.589129 | 1.9 | 18.9 | 0.579319 | 2 | 19.42 | 0.623808 | 2.8 | 19.29 | 0.610825 | 2.6 | 19.24 | 0.602343 | 2.5 | 19.3 | 0.583283 | 2.6 |
| 679 | 60S ribosomal protein L13 OS=Homo sapiens GN=RPL13 PE=1 SV=4 | RL13_HUMAN | 24 kDa |  | 0.0001 | 1.40 | 1.80 | 2.60 | 18.04 | 0.861348 | 18 | 0.831326 | 18.5 | 0.533239 | 1.4 | 18.49 | 0.540265 | 1.4 | 18.85 | 0.537262 | 1.9 | 18.72 | 0.539545 | 1.7 | 19.32 | 0.533438 | 2.5 | 19.38 | 0.533937 | 2.7 |
| 1689 | Tubulin alpha-1A chain OS=Homo sapiens GN=TUBA1A PE=1 SV=1 | TBA1A_HUMAN | 50 kDa | TRUE | 0.013 | 1.95 | 1.80 | 2.60 | 18.3 | 0.618118 | 18.29 | 0.653723 | 19.25 | 0.49772 | 1.9 | 19.28 | 0.500691 | 2 | 19.23 | 0.577095 | 1.8 | 19.16 | 0.492922 | 1.8 | 19.74 | 0.497472 | 2.7 | 19.65 | 0.535368 | 2.5 |
| 11 | Hemoglobin subunit beta OS=Homo sapiens GN=HBB PE=1 SV=2 | HBB_HUMAN | 16 kDa | TRUE | 0.0001 | 1.85 | 1.85 | 2.60 | 17.48 | 1.030159 | 17.7 | 0.918439 | 18.26 | 0.755466 | 2 | 18.28 | 0.73634 | 1.7 | 18.35 | 0.703605 | 1.9 | 18.43 | 0.619289 | 1.8 | 18.74 | 0.737341 | 2.6 | 18.94 | 0.745789 | 2.6 |
| 139 | Cathepsin G OS=Homo sapiens GN=CTSG PE=1 SV=2 | CATG_HUMAN | 29 kDa |  | 0.0001 | 2.10 | 2.10 | 2.60 | 17.66 | 0.950926 | 17.76 | 0.802858 | 18.72 | 0.672283 | 2.2 | 18.74 | 0.668483 | 2 | 18.71 | 0.59084 | 2.2 | 18.72 | 0.606347 | 2 | 18.96 | 0.576336 | 2.6 | 19.06 | 0.654546 | 2.6 |
| 145 | Protein S100-A9 OS=Homo sapiens GN=S100A9 PE=1 SV=1 | S10A9_HUMAN | 13 kDa |  | 0.0001 | 1.70 | 2.10 | 2.60 | 17.82 | 1.206108 | 17.92 | 0.980799 | 18.69 | 0.674528 | 1.7 | 18.69 | 0.662146 | 1.7 | 18.95 | 0.655965 | 2.2 | 18.83 | 0.600645 | 2 | 19.15 | 0.647751 | 2.7 | 19.11 | 0.710894 | 2.5 |
| 915 | Nuclear autoantigenic sperm protein OS=Homo sapiens GN=NASP PE=1 SV=2 | NASP_HUMAN | 85 kDa | TRUE | 0.0001 | 2.30 | 2.25 | 2.60 | 16.92 | 1.098339 | 16.84 | 1.035777 | 18.03 | 0.512894 | 2.2 | 18.02 | 0.531551 | 2.4 | 18.18 | 0.550436 | 2.3 | 18.01 | 0.537117 | 2.2 | 18.27 | 0.523346 | 2.5 | 18.29 | 0.545431 | 2.7 |
| 901 | F-box only protein 7 OS=Homo sapiens GN=FBXO7 PE=1 SV=1 | FBX7_HUMAN | 59 kDa |  | 0.0001 | 2.80 | 2.45 | 2.65 | 18.31 | 1.032956 | 18.42 | 0.994724 | 19.71 | 0.609615 | 2.8 | 19.79 | 0.596951 | 2.8 | 19.62 | 0.553196 | 2.5 | 19.6 | 0.555118 | 2.4 | 19.74 | 0.538186 | 2.8 | 19.74 | 0.663383 | 2.5 |
| 671 | Neutrophil elastase OS=Homo sapiens GN=ELANE PE=1 SV=1 | ELNE_HUMAN | 29 kDa |  | 0.0001 | 2.20 | 1.85 | 2.70 | 17.83 | 1.198007 | 18.19 | 0.916865 | 19.08 | 0.62278 | 2.5 | 19.11 | 0.62206 | 1.9 | 18.73 | 0.629499 | 1.9 | 19.01 | 0.569971 | 1.8 | 19.31 | 0.578696 | 2.8 | 19.55 | 0.700608 | 2.6 |
| 965 | 60S ribosomal protein L28 OS=Homo sapiens GN=RPL28 PE=1 SV=3 | RL28_HUMAN | 16 kDa | TRUE | 0.0046 | 1.95 | 1.85 | 2.70 | 16.31 | 1.090919 | 16.5 | 0.994904 | 17.21 | 0.501623 | 1.9 | 17.35 | 0.613035 | 2 | 17.4 | 0.557558 | 2 | 17.21 | 0.524389 | 1.7 | 17.92 | 0.652955 | 2.8 | 17.99 | 0.681982 | 2.6 |
| 1759 | TATA-binding protein-associated factor 2N OS=Homo sapiens GN=TAF15 PE=1 SV=1 | RBP56_HUMAN | 62 kDa | TRUE | 0.026 | 1.65 | 1.85 | 2.70 | 16.53 | 0.74733 | 16.78 | 1.050731 | 17.43 | 0.520264 | 1.9 | 17.28 | 0.587059 | 1.4 | 17.66 | 0.59237 | 2.2 | 17.37 | 0.568657 | 1.5 | 18.08 | 0.608777 | 2.9 | 18.09 | 0.624787 | 2.5 |
| 1271 | Astrocytic phosphoprotein PEA-15 OS=Homo sapiens GN=PEA15 PE=1 SV=2 | PEA15_HUMAN | 15 kDa |  | 0.0022 | 1.45 | 1.85 | 2.70 | 16.65 | 0.810087 | 16.55 | 0.950263 | 17.01 | 0.508607 | 1.4 | 17.08 | 0.5616 | 1.5 | 17.55 | 0.494775 | 1.9 | 17.39 | 0.4901 | 1.8 | 18.01 | 0.493207 | 2.6 | 18.07 | 0.492589 | 2.8 |
| 1758 | Destrin OS=Homo sapiens GN=DSTN PE=1 SV=3 | DEST_HUMAN | 19 kDa | TRUE | 0.0074 | 1.90 | 2.25 | 2.70 | 16.47 | 0.489732 | 16.53 | 0.525949 | 17.37 | 0.48864 | 1.9 | 17.45 | 0.492519 | 1.9 | 17.67 | 0.491762 | 2.3 | 17.64 | 0.495095 | 2.2 | 17.89 | 0.50257 | 2.7 | 17.96 | 0.49166 | 2.7 |
| 1019 | Prefoldin subunit 3 OS=Homo sapiens GN=VBP1 PE=1 SV=3 | PFD3_HUMAN | 23 kDa |  | 0.0001 | 1.55 | 2.25 | 2.75 | 16.78 | 0.858096 | 16.7 | 1.055027 | 17.36 | 0.593803 | 1.4 | 17.41 | 0.569186 | 1.7 | 18.02 | 0.620704 | 2.3 | 17.89 | 0.622843 | 2.2 | 18.18 | 0.582864 | 2.6 | 18.22 | 0.560937 | 2.9 |
| 77 | Cofilin-1 OS=Homo sapiens GN=CFL1 PE=1 SV=3 | COF1_HUMAN | 19 kDa | TRUE | 0.0001 | 1.95 | 2.30 | 2.75 | 17.62 | 1.243359 | 17.68 | 1.063929 | 18.53 | 0.586785 | 2 | 18.58 | 0.57403 | 1.9 | 18.85 | 0.673208 | 2.5 | 18.66 | 0.604877 | 2.1 | 19.03 | 0.658772 | 2.9 | 18.95 | 0.61062 | 2.6 |
| 981 | Thioredoxin OS=Homo sapiens GN=TXN PE=1 SV=3 | THIO_HUMAN | 12 kDa |  | 0.00096 | 2.25 | 2.40 | 2.75 | 17.89 | 1.81828 | 17.97 | 1.606843 | 19 | 0.537795 | 2.3 | 19.04 | 0.537993 | 2.2 | 19.13 | 0.562405 | 2.6 | 19 | 0.533263 | 2.2 | 19.26 | 0.555099 | 2.9 | 19.24 | 0.563953 | 2.6 |
| 1383 | 14 kDa phosphohistidine phosphatase OS=Homo sapiens GN=PHPT1 PE=1 SV=1 | PHP14_HUMAN | 14 kDa |  | 0.011 | 1.35 | 2.55 | 2.75 | 18.13 | 0.685953 | 18.18 | 0.576323 | 18.51 | 0.582472 | 1.2 | 18.75 | 0.601667 | 1.5 | 19.61 | 0.688624 | 2.9 | 19.31 | 0.528821 | 2.2 | 19.55 | 0.616761 | 2.8 | 19.58 | 0.604388 | 2.7 |
| 337 | Proteasome subunit alpha type-2 OS=Homo sapiens GN=PSMA2 PE=1 SV=2 | PSA2_HUMAN | 26 kDa | TRUE | 0.0001 | 1.95 | 2.50 | 2.80 | 17.77 | 1.315434 | 18.07 | 1.113624 | 18.87 | 0.6135 | 2.1 | 18.89 | 0.578154 | 1.8 | 19.27 | 0.605964 | 2.9 | 19.14 | 0.603534 | 2.1 | 19.28 | 0.60001 | 2.9 | 19.46 | 0.609742 | 2.7 |
| 61 | Histone H2B type 1-C/E/F/G/I OS=Homo sapiens GN=HIST1H2BC PE=1 SV=4 | H2B1C_HUMAN | 14 kDa | TRUE | 0.011 | 2.10 | 2.10 | 2.85 | 18.05 | 1.622317 | 18.09 | 1.491954 | 18.91 | 0.690822 | 2.1 | 18.96 | 0.636702 | 2.1 | 19.14 | 0.617926 | 2.4 | 18.82 | 0.603472 | 1.8 | 19.46 | 0.596641 | 3 | 19.39 | 0.608508 | 2.7 |
| 968 | 40S ribosomal protein S15 OS=Homo sapiens GN=RPS15 PE=1 SV=2 | RS15_HUMAN | 17 kDa |  | 0.0001 | 1.30 | 2.10 | 2.90 | 16.37 | 0.615046 | 16.57 | 0.73245 | 16.97 | 0.811985 | 1.4 | 16.88 | 0.744051 | 1.2 | 17.53 | 0.67783 | 2.2 | 17.62 | 0.663656 | 2 | 17.98 | 0.788474 | 3 | 18.12 | 0.672002 | 2.8 |
| 472 | 60S ribosomal protein L10a OS=Homo sapiens GN=RPL10A PE=1 SV=2 | RL10A_HUMAN | 25 kDa |  | 0.0001 | 1.90 | 2.25 | 2.90 | 17.35 | 1.040852 | 17.32 | 0.856665 | 18.22 | 0.587301 | 1.9 | 18.22 | 0.597458 | 1.9 | 18.5 | 0.586812 | 2.2 | 18.42 | 0.555885 | 2.3 | 18.84 | 0.575804 | 2.8 | 18.92 | 0.560093 | 3 |
| 1801 | rRNA 2'-O-methyltransferase fibrillarin OS=Homo sapiens GN=FBL PE=1 SV=2 | FBRL_HUMAN | 34 kDa |  | 0.017 | 1.55 | 2.25 | 2.90 | 16.71 | 0.634572 | 16.95 | 0.61446 | 17.5 | 0.662673 | 1.8 | 17.33 | 0.505839 | 1.3 | 17.92 | 0.68762 | 2.4 | 18.07 | 0.93204 | 2.1 | 18.1 | 0.56105 | 2.7 | 18.55 | 1.218958 | 3.1 |
| 576 | KH domain-containing, RNA-binding, signal transduction-associated protein 1 OS=Homo sapiens GN=KHDRBS1 PE=1 SV=1 | KHDR1_HUMAN | 48 kDa |  | 0.0001 | 2.10 | 2.60 | 2.90 | 16.97 | 0.872968 | 17.27 | 0.874893 | 18.18 | 0.533929 | 2.3 | 18.18 | 0.526236 | 1.9 | 18.49 | 0.565667 | 3 | 18.37 | 0.534145 | 2.2 | 18.61 | 0.531165 | 3.2 | 18.58 | 0.579376 | 2.6 |
| 401 | Tropomyosin alpha-3 chain OS=Homo sapiens GN=TPM3 PE=1 SV=2 | TPM3_HUMAN | 33 kDa | TRUE | 0.00071 | 1.70 | 1.90 | 2.95 | 17.57 | 1.116113 | 17.49 | 0.957725 | 18.22 | 0.543043 | 1.6 | 18.28 | 0.56664 | 1.8 | 18.45 | 0.551754 | 1.9 | 18.37 | 0.53227 | 1.9 | 19.13 | 0.729276 | 2.8 | 19.18 | 0.730923 | 3.1 |
| 1680 | Barrier-to-autointegration factor OS=Homo sapiens GN=BANF1 PE=1 SV=1 | BAF_HUMAN | 10 kDa |  | 0.019 | 2.05 | 2.45 | 2.95 | 15.15 | 0.528431 | 14.73 | 0.501137 | 15.88 | 0.485455 | 1.7 | 16 | 0.49429 | 2.4 | 15.93 | 0.610536 | 1.7 | 16.42 | 0.494658 | 3.2 | 16.61 | 0.488343 | 2.7 | 16.41 | 0.516098 | 3.2 |
| 1163 | Sialic acid synthase OS=Homo sapiens GN=NANS PE=1 SV=2 | SIAS_HUMAN | 40 kDa | TRUE | 0.013 | 2.35 | 2.30 | 3.00 | 15.29 | 0.658561 | 15.34 | 1.31051 | 16.55 | 0.486044 | 2.4 | 16.53 | 0.484676 | 2.3 | 16.56 | 0.482982 | 2.4 | 16.5 | 0.501583 | 2.2 | 16.92 | 0.556881 | 3.1 | 16.9 | 0.527853 | 2.9 |
| 1243 | ADP-ribosylation factor 4 OS=Homo sapiens GN=ARF4 PE=1 SV=3 | ARF4_HUMAN | 21 kDa | TRUE | 0.00036 | 2.35 | 2.40 | 3.00 | 17.15 | 0.863629 | 17.26 | 0.747272 | 18.4 | 0.554497 | 2.4 | 18.43 | 0.527926 | 2.3 | 18.39 | 0.510355 | 2.3 | 18.64 | 0.715355 | 2.5 | 18.73 | 0.522221 | 3 | 18.83 | 0.533737 | 3 |
| 1231 | V-type proton ATPase subunit F OS=Homo sapiens GN=ATP6V1F PE=1 SV=2 | VATF_HUMAN | 13 kDa | TRUE | 0.0056 | 2.30 | 2.55 | 3.00 | 15.73 | 0.58789 | 15.94 | 0.632473 | 16.98 | 0.568992 | 2.4 | 17.07 | 0.552433 | 2.2 | 17.16 | 0.516941 | 2.7 | 17.24 | 0.520452 | 2.4 | 17.26 | 0.562614 | 2.9 | 17.62 | 0.802435 | 3.1 |
| 456 | Protein S100-A4 OS=Homo sapiens GN=S100A4 PE=1 SV=1 | S10A4_HUMAN | 12 kDa | TRUE | 0.0001 | 2.40 | 2.45 | 3.10 | 17.69 | 1.33529 | 17.72 | 1.145054 | 18.97 | 0.602017 | 2.4 | 18.94 | 0.569019 | 2.4 | 19.14 | 0.626237 | 2.7 | 18.89 | 0.579558 | 2.2 | 19.44 | 0.590441 | 3.3 | 19.23 | 0.592315 | 2.9 |
| 633 | S-phase kinase-associated protein 1 OS=Homo sapiens GN=SKP1 PE=1 SV=2 | SKP1_HUMAN | 19 kDa |  | 0.0001 | 2.80 | 2.70 | 3.10 | 16.4 | 1.350044 | 16.48 | 1.34917 | 17.95 | 0.521005 | 2.9 | 17.96 | 0.538458 | 2.7 | 18.1 | 0.57475 | 3 | 17.88 | 0.539986 | 2.4 | 18.19 | 0.537038 | 3.3 | 18.06 | 0.540067 | 2.9 |
| 571 | Actin-related protein 2/3 complex subunit 3 OS=Homo sapiens GN=ARPC3 PE=1 SV=3 | ARPC3_HUMAN | 21 kDa |  | 0.0001 | 2.70 | 3.05 | 3.10 | 17.52 | 1.225066 | 17.6 | 0.885312 | 19.06 | 0.785924 | 2.8 | 18.99 | 0.763252 | 2.6 | 19.2 | 0.720585 | 3.3 | 19.06 | 0.629541 | 2.8 | 19.13 | 0.616526 | 3.2 | 19.18 | 0.597269 | 3 |
| 1459 | 40S ribosomal protein S26 OS=Homo sapiens GN=RPS26 PE=1 SV=3 | RS26_HUMAN | 13 kDa |  | 0.026 | 2.05 | 2.20 | 3.15 | 17.06 | 1.216329 | 17.12 | 0.95878 | 18.12 | 0.544894 | 2 | 18.22 | 0.52891 | 2.1 | 18.17 | 0.596377 | 2.2 | 18.21 | 0.562042 | 2.2 | 18.78 | 0.693429 | 3.3 | 18.73 | 0.773337 | 3 |
| 181 | Histone H3.2 OS=Homo sapiens GN=HIST2H3A PE=1 SV=3 | H32_HUMAN | 15 kDa | TRUE | 0.0001 | 2.55 | 2.45 | 3.15 | 17.35 | 1.113489 | 17.48 | 1.014209 | 18.65 | 0.641986 | 2.6 | 18.74 | 0.687024 | 2.5 | 18.69 | 0.631786 | 2.6 | 18.64 | 0.665185 | 2.3 | 18.99 | 0.676825 | 3.3 | 18.99 | 0.710081 | 3 |
| 1048 | Histone H2B type 2-E OS=Homo sapiens GN=HIST2H2BE PE=1 SV=3 | H2B2E_HUMAN | 14 kDa | TRUE | 0.013 | 2.45 | 2.50 | 3.15 | 17.79 | 1.563217 | 18.12 | 1.328808 | 19.31 | 0.577702 | 2.7 | 19.27 | 0.570146 | 2.2 | 19.38 | 0.605844 | 3 | 19.02 | 0.626409 | 2 | 19.58 | 0.564712 | 3.4 | 19.59 | 0.604461 | 2.9 |
| 1775 | Interleukin-1 receptor-associated kinase 4 OS=Homo sapiens GN=IRAK4 PE=1 SV=1 | IRAK4_HUMAN | 52 kDa |  | 0.019 | 4.40 | 3.75 | 3.15 | 13.56 | 1.873886 | 13.28 | 1.784302 | 15.34 | 0.482481 | 4.2 | 15.19 | 0.521115 | 4.6 | 15.05 | 0.495859 | 3.2 | 15.11 | 0.488602 | 4.3 | 14.85 | 0.489356 | 3 | 14.81 | 0.487039 | 3.3 |
| 361 | Histone H1.4 OS=Homo sapiens GN=HIST1H1E PE=1 SV=2 | H14_HUMAN | 22 kDa | TRUE | 0.00044 | 1.60 | 2.55 | 3.20 | 17.6 | 0.865133 | 17.77 | 0.678815 | 18.35 | 0.512589 | 1.7 | 18.3 | 0.515352 | 1.5 | 19.25 | 0.584145 | 3.1 | 18.74 | 0.513444 | 2 | 19.37 | 0.60182 | 3.4 | 19.33 | 0.551013 | 3 |
| 962 | Ran-specific GTPase-activating protein OS=Homo sapiens GN=RANBP1 PE=1 SV=1 | RANG_HUMAN | 23 kDa |  | 0.0001 | 2.25 | 2.50 | 3.30 | 17.58 | 0.853887 | 17.66 | 0.867226 | 18.77 | 0.54029 | 2.3 | 18.78 | 0.526574 | 2.2 | 19.06 | 0.541598 | 2.8 | 18.79 | 0.576362 | 2.2 | 19.3 | 0.524893 | 3.3 | 19.33 | 0.539548 | 3.3 |
| 797 | Protein S100-A2 OS=Homo sapiens GN=S100A2 PE=1 SV=3 | S10A2_HUMAN | 11 kDa |  | 0.0001 | 1.65 | 3.95 | 3.30 | 17.79 | 0.98918 | 18.02 | 0.871975 | 18.53 | 0.643912 | 1.7 | 18.6 | 0.666783 | 1.6 | 19.99 | 0.625873 | 4.9 | 19.55 | 0.57055 | 3 | 19.71 | 0.543347 | 3.9 | 19.45 | 0.539529 | 2.7 |
| 55 | Protein S100-A8 OS=Homo sapiens GN=S100A8 PE=1 SV=1 | S10A8_HUMAN | 11 kDa | TRUE | 0.0001 | 1.75 | 4.95 | 3.30 | 15.41 | 1.039195 | 15.58 | 0.952567 | 16.47 | 1.07187 | 1.9 | 16.48 | 0.999734 | 1.6 | 17.42 | 1.326682 | 5.8 | 17.27 | 1.098039 | 4.1 | 17.1 | 0.83071 | 3.7 | 17.01 | 0.861036 | 2.9 |
| 1481 | Tropomyosin alpha-1 chain OS=Homo sapiens GN=TPM1 PE=1 SV=2 | TPM1_HUMAN | 33 kDa | TRUE | 0.0081 | 1.35 | 1.55 | 3.40 | 17.75 | 0.705859 | 17.74 | 0.575721 | 18.03 | 0.602018 | 1.3 | 18.27 | 0.522551 | 1.4 | 18.12 | 0.602025 | 1.3 | 18.62 | 0.75257 | 1.8 | 19.48 | 0.934187 | 3.3 | 19.58 | 0.798894 | 3.5 |
| 199 | Tropomyosin alpha-4 chain OS=Homo sapiens GN=TPM4 PE=1 SV=3 | TPM4_HUMAN | 29 kDa | TRUE | 0.0001 | 1.10 | 1.85 | 3.45 | 18.14 | 0.810226 | 18.37 | 0.677946 | 18.32 | 0.541703 | 1.1 | 18.42 | 0.537558 | 1.1 | 19.19 | 0.602582 | 2.1 | 19 | 0.557562 | 1.6 | 20.02 | 0.83322 | 3.8 | 19.99 | 0.792708 | 3.1 |
| 1164 | 60S ribosomal protein L19 OS=Homo sapiens GN=RPL19 PE=1 SV=1 | RL19_HUMAN | 23 kDa |  | 0.00044 | 1.45 | 2.15 | 3.45 | 16.94 | 0.627538 | 16.99 | 0.658919 | 17.45 | 0.618592 | 1.5 | 17.43 | 0.616806 | 1.4 | 18.14 | 0.581556 | 2.3 | 17.98 | 0.552764 | 2 | 18.7 | 0.603547 | 3.4 | 18.79 | 0.57061 | 3.5 |
| 1491 | Matrin-3 OS=Homo sapiens GN=MATR3 PE=1 SV=2 | MATR3_HUMAN | 95 kDa |  | 0.011 | 1.25 | 2.55 | 3.55 | 15.53 | 0.482962 | 15.71 | 0.482838 | 16.06 | 0.500536 | 1.4 | 15.82 | 0.484089 | 1.1 | 17.17 | 0.550957 | 3.1 | 16.72 | 0.499369 | 2 | 17.46 | 0.869474 | 3.8 | 17.42 | 0.920132 | 3.3 |
| 1764 | Histone H2A type 2-B OS=Homo sapiens GN=HIST2H2AB PE=1 SV=3 | H2A2B_HUMAN | 14 kDa | TRUE | 0.013 | 2.55 | 2.75 | 3.85 | 15.91 | 0.57853 | 15.87 | 0.55361 | 17.14 | 0.575991 | 2.3 | 17.37 | 0.485487 | 2.8 | 17.35 | 0.488123 | 2.7 | 17.35 | 0.485393 | 2.8 | 17.68 | 0.521284 | 3.4 | 17.97 | 0.51356 | 4.3 |
| 808 | Protein S100-A6 OS=Homo sapiens GN=S100A6 PE=1 SV=1 | S10A6_HUMAN | 10 kDa |  | 0.00017 | 2.10 | 4.20 | 6.60 | 16.4 | 1.518713 | 16.99 | 1.175987 | 17.87 | 0.648015 | 2.3 | 17.96 | 0.678623 | 1.9 | 18.46 | 0.979718 | 5.6 | 18.32 | 0.687811 | 2.8 | 18.92 | 1.095353 | 8 | 18.91 | 0.986871 | 5.2 |
|  |  |  |  |  |  |  |  |  |  |  |  |  |  |  |  |  |  |  |  |  |  |  |  |  |  |  |  |  |  |  |
