# Supplementary material for: Proteomic Analysis of Exudates from Chronic Ulcer of Diabetic Foot Treated with Scorpion Antimicrobial Peptide
Source: Mediators Inflamm. 2022 Oct 3;2022:5852786. doi: 10.1155/2022/5852786 (PMC9550419; doi:10.1155/2022/5852786)
Supplement: Supplementary Materials — Bacteriological identification of diabetic foot ulcer wounds is available on Supplementary Table 1–3. Identification results by mass spectrometry is available on Supplementary Table 4; analysis of proteins in diabetic wound exudate by iTRAQ is available on Supplementary Table 5; IPA technology for the annotation of differential proteins is available on Supplementary Table 6; classical signal pathway analysis of differential proteins is available on Supplementary Table 7; analysis of upstream regulatory factors is available on Supplementary Table 8; analysis of possible interaction networks in differential proteins is available on Supplementary Table 9. [file 5852786.f1.zip › Supplementary Table 6.docx]

Supplementary Table 6 IPA technology for the annotation of differential proteins

| **C-B** |  |  |  |  |  |  |
| --- | --- | --- | --- | --- | --- | --- |
| Functional classification | Name of the function | Subfunction comments | *P* value | Predict activity | Z-score | Number of related molecules |
| Cellular Movement | cell movement | cell movement | 4.16E-08 | Increased | 2.45 | 42 |
| Cellular Movement | migration | migration of cells | 5.57E-07 | Increased | 2.293 | 37 |
| Cancer, Organismal Injury and Abnormalities, Reproductive System Disease | mammary tumor | mammary tumor | 1.10E-06 | Increased | 2.191 | 38 |
| Cellular Development | differentiation | differentiation of cells | 1.64E-03 | Increased | 2.531 | 32 |
| Organismal Survival | survival | survival of organism | 1.86E-03 | Increased | 2.56 | 14 |
| Protein Synthesis | synthesis | synthesis of protein | 1.95E-03 | Increased | 2 | 10 |
| Cellular Movement | invasion | invasion of cells | 4.93E-03 | Increased | 2.735 | 15 |
| Cell Death and Survival | necrosis | necrosis | 2.12E-06 | Decreased | -2.258 | 44 |
| Cancer, Cell Death and Survival, Organismal Injury and Abnormalities, Tumor Morphology | cell death | cell death of osteosarcoma cells | 1.20E-03 | Decreased | -2.236 | 5 |
| Cell-To-Cell Signaling and Interaction, Hematological System Development and Function | binding | binding of leukocytes | 1.41E-03 | Decreased | -2.2 | 6 |
| Cancer, Cell Death and Survival, Organismal Injury and Abnormalities, Tumor Morphology | cell death | cell death of cancer cells | 2.59E-03 | Decreased | -2.343 | 9 |
| Cell Death and Survival, Neurological Disease | cell death | cell death of brain cells | 3.31E-03 | Decreased | -2.571 | 7 |
| Cell Death and Survival, Neurological Disease | cell death | cell death of cerebral cortex cells | 5.19E-03 | Decreased | -2.369 | 6 |
| Dermatological Diseases and Conditions | psoriasis | psoriasis | 9.54E-21 |  |  | 34 |
| Inflammatory Response | inflammation | inflammation of organ | 2.87E-14 |  | -1.33 | 40 |
| Immunological Disease | immediate hypersensitivity | immediate hypersensitivity | 1.48E-13 |  |  | 20 |
| Dermatological Diseases and Conditions, Immunological Disease, Inflammatory Disease, Inflammatory Response | atopic dermatitis | atopic dermatitis | 3.52E-13 |  |  | 18 |
|  |  |  |  |  |  |  |
| **D-B** |  |  |  |  |  |  |
|  |  |  |  |  |  |  |
| Infectious Diseases | infection | Viral Infection | 2.11E-16 | Increased | 4.28 | 79 |
| Protein Synthesis | synthesis | synthesis of protein | 6.04E-15 | Increased | 2.95 | 36 |
| Cellular Growth and Proliferation | proliferation | proliferation of cells | 2.12E-11 | Increased | 2.166 | 121 |
| Cellular Movement | cell movement | cell movement | 1.81E-10 | Increased | 2.657 | 81 |
| Cellular Movement | migration | migration of cells | 2.39E-10 | Increased | 2.346 | 75 |
| Infectious Diseases | replication | replication of RNA virus | 1.62E-07 | Increased | 2.054 | 27 |
| Infectious Diseases | infection | infection by RNA virus | 4.22E-07 | Increased | 4.051 | 38 |
| Cell Death and Survival | survival | cell survival | 1.72E-06 | Increased | 3.393 | 51 |
| Infectious Diseases | infection | infection by Hepatitis C virus | 3.47E-06 | Increased | 2.195 | 6 |
| Cellular Development, Cellular Growth and Proliferation | cell proliferation | cell proliferation of tumor cell lines | 6.19E-06 | Increased | 2.442 | 54 |
| Cell Death and Survival | cell viability | cell viability | 1.53E-05 | Increased | 3.184 | 46 |
| Infectious Diseases | infection | infection of cells | 1.67E-05 | Increased | 4.162 | 32 |
| Cellular Development | differentiation | differentiation of cells | 4.20E-05 | Increased | 2.389 | 68 |
| Infectious Diseases, Organismal Injury and Abnormalities | infection | infection of embryonic cell lines | 1.31E-04 | Increased | 3.036 | 14 |
| Infectious Diseases | infection | infection of epithelial cell lines | 1.31E-04 | Increased | 3.036 | 14 |
| Infectious Diseases | infection | infection of kidney cell lines | 1.85E-04 | Increased | 3.036 | 14 |
| Cell Death and Survival | cell viability | cell viability of tumor cell lines | 2.41E-04 | Increased | 3.344 | 29 |
| Organismal Survival | survival | survival of organism | 2.57E-04 | Increased | 3.114 | 27 |
| Infectious Diseases | infection | infection of hepatoma cell lines | 3.05E-04 | Increased | 2.408 | 6 |
| Cellular Movement | invasion | invasion of cells | 3.18E-04 | Increased | 3.304 | 31 |
| Infectious Diseases | HIV infection | HIV infection | 4.54E-04 | Increased | 3.678 | 26 |
| Cellular Assembly and Organization, Tissue Development | formation | formation of filaments | 4.90E-04 | Increased | 2.008 | 17 |
| Infectious Diseases | infection | infection by HIV-1 | 9.42E-04 | Increased | 3.678 | 22 |
| Cell Death and Survival | cell death | cell death | 2.83E-21 | Decreased | -3.811 | 136 |
| Cell Death and Survival | necrosis | necrosis | 1.01E-16 | Decreased | -4.467 | 108 |
| Cancer, Cell Death and Survival, Organismal Injury and Abnormalities, Tumor Morphology | cell death | cell death of osteosarcoma cells | 1.60E-14 | Decreased | -4.359 | 19 |
| Cell Death and Survival | cell death | cell death of tumor cell lines | 6.38E-12 | Decreased | -2.688 | 68 |
| Cell Death and Survival | apoptosis | apoptosis of tumor cell lines | 4.42E-11 | Decreased | -2.809 | 57 |
| Cell Death and Survival | apoptosis | apoptosis | 1.04E-10 | Decreased | -3.561 | 94 |
| Connective Tissue Disorders, Inflammatory Disease, Skeletal and Muscular Disorders | rheumatic disease | Rheumatic Disease | 4.85E-10 | Decreased | -2.381 | 51 |
| Connective Tissue Disorders, Inflammatory Disease, Inflammatory Response, Skeletal and Muscular Disorders | inflammation | inflammation of joint | 7.54E-10 | Decreased | -2.345 | 46 |
| Cancer, Cell Death and Survival, Organismal Injury and Abnormalities, Tumor Morphology | cell death | cell death of tumor cells | 6.44E-09 | Decreased | -4.577 | 28 |
| Cancer, Cell Death and Survival, Organismal Injury and Abnormalities, Tumor Morphology | cell death | cell death of cancer cells | 1.07E-08 | Decreased | -4.753 | 25 |
| Cancer, Organismal Injury and Abnormalities | cancer | cancer | 1.86E-07 | Decreased | -3.017 | 252 |
| Organismal Survival | morbidity or mortality | morbidity or mortality | 4.81E-06 | Decreased | -5.336 | 75 |
| Organismal Survival | organismal death | organismal death | 5.66E-06 | Decreased | -5.145 | 74 |
| Cancer, Organismal Injury and Abnormalities | urogenital cancer | urogenital cancer | 1.29E-05 | Decreased | -2.449 | 134 |
| Cell Death and Survival | cell death | cell death of central nervous system cells | 2.96E-04 | Decreased | -2.615 | 14 |
| Cardiovascular System Development and Function, Cellular Development, Cellular Function and Maintenance, Cellular Growth and Proliferation, Organismal Development, Tissue Development | proliferation | proliferation of endothelial cells | 5.82E-04 | Decreased | -2.513 | 14 |
| Tissue Development | development | development of epithelial tissue | 7.38E-04 | Decreased | -2.467 | 20 |
| Cell Death and Survival | cell death | cell death of brain | 8.88E-04 | Decreased | -2.909 | 13 |
| Dermatological Diseases and Conditions | psoriasis | psoriasis | 1.14E-18 |  |  | 47 |
| RNA Post-Transcriptional Modification | processing | processing of RNA | 1.48E-16 |  | -0.391 | 31 |
| Protein Synthesis | translation | translation | 2.35E-16 |  | 1.339 | 29 |
| Protein Synthesis | translation | translation of protein | 1.19E-15 |  | 1.214 | 28 |
| Immunological Disease | immediate hypersensitivity | immediate hypersensitivity | 1.28E-15 |  | -0.655 | 31 |
| Dermatological Diseases and Conditions, Immunological Disease, Inflammatory Disease, Inflammatory Response | atopic dermatitis | atopic dermatitis | 1.14E-14 |  |  | 27 |
