# Supplementary material for: Proteomic Analysis of Exudates from Chronic Ulcer of Diabetic Foot Treated with Scorpion Antimicrobial Peptide
Source: Mediators Inflamm. 2022 Oct 3;2022:5852786. doi: 10.1155/2022/5852786 (PMC9550419; doi:10.1155/2022/5852786)
Supplement: Supplementary Materials — Bacteriological identification of diabetic foot ulcer wounds is available on Supplementary Table 1–3. Identification results by mass spectrometry is available on Supplementary Table 4; analysis of proteins in diabetic wound exudate by iTRAQ is available on Supplementary Table 5; IPA technology for the annotation of differential proteins is available on Supplementary Table 6; classical signal pathway analysis of differential proteins is available on Supplementary Table 7; analysis of upstream regulatory factors is available on Supplementary Table 8; analysis of possible interaction networks in differential proteins is available on Supplementary Table 9. [file 5852786.f1.zip › Supplementary Table 7.docx]

Supplementary Table 7 Classical signal pathway analysis of differential proteins

| **C-B** |  |  |  |  |
| --- | --- | --- | --- | --- |
| Name of pathway | -log (*P* value) | Z-value | Identification rate | Identification of molecular |
| EIF2 Signaling | 9.02 | 2.24 | 0.07 | RPL27A,RPS19,RPS15,MAPK3,RPS23,RPS9,RPL19,RPL10,EIF3J,RPL26,RPS17,EIF2S2 |
| Cardiac Hypertrophy Signaling | 1.98 | 2.24 | 0.02 | ADSS,CALML5,MAPK3,MYL12A,HSPB1 |
| Phospholipase C Signaling | 1.88 | 2.24 | 0.02 | CALML5,MAPK3,IGHG4,IGHG1,MYL12A |
| Dendritic Cell Maturation | 1.68 | 2.00 | 0.02 | MAPK3,FSCN1,IGHG4,IGHG1 |
| Acute Phase Response Signaling | 8.30 | 0.00 | 0.07 | HP,APOA1,MBL2,APOH,TF,C4BPA,MAPK3,C9,HRG,LBP,C5 |
| Complement System | 7.13 | NaN | 0.16 | MBL2,C4BPA,C9,C7,C6,C5 |
| Glycolysis I | 6.50 | NaN | 0.20 | PGK1,ENO1,TPI1,ENO2,FBP1 |
| LXR/RXR Activation | 6.22 | 0.00 | 0.07 | HPR,APOA1,APOH,TF,C9,S100A8,PLTP,LBP |
| Regulation of eIF4 and p70S6K Signaling | 5.60 | NaN | 0.05 | RPS19,RPS15,MAPK3,RPS23,RPS9,EIF3J,RPS17,EIF2S2 |
| FXR/RXR Activation | 5.00 | NaN | 0.06 | HPR,APOA1,APOH,TF,C9,FBP1,PLTP |
| Gluconeogenesis I | 4.88 | NaN | 0.16 | PGK1,ENO1,ENO2,FBP1 |
| Adenine and Adenosine Salvage I | 4.46 | NaN | 1.00 | PNP,APRT |
| Systemic Lupus Erythematosus Signaling | 4.39 | NaN | 0.04 | MAPK3,C9,C7,IGHG4,C6,IGHG1,LSM2,C5 |
| mTOR Signaling | 3.91 | NaN | 0.04 | RPS19,RPS15,MAPK3,RPS23,RPS9,EIF3J,RPS17 |
| Role of IL-17A in Psoriasis | 2.59 | NaN | 0.15 | S100A7,S100A8 |
|  |  |  |  |  |
| **D-B** |  |  |  |  |
| EIF2 Signaling | 33.70 | 4.58 | 0.21 | RPL24,RPS23,RPS18,RPLP2,etc |
| Regulation of eIF4 and p70S6K Signaling | 9.97 | NaN | 0.11 | PABPC1,RPS19,RPS23,RPS18,etc |
| LXR/RXR Activation | 9.10 | -0.53 | 0.12 | APOE,LYZ,ALB,APOA1,etc |
| Acute Phase Response Signaling | 9.02 | -1.41 | 0.09 | APOH,C9,HNRNPK,FGG,etc |
| Complement System | 9.00 | NaN | 0.24 | MBL2,C4BPA,C9,C7,ect |
| mTOR Signaling | 7.51 | NaN | 0.08 | RPS19,RPS23,RPS18,RPS10,etc |
| FXR/RXR Activation | 6.93 | NaN | 0.10 | APOE,ALB,APOA1,APOH,etc |
| Glycogen Degradation II | 5.03 | NaN | 0.36 | PGM3,TYMP,PYGL,AGL |
| Glycogen Degradation III | 4.71 | NaN | 0.31 | PGM3,TYMP,PYGL,AGL |
| Clathrin-mediated Endocytosis Signaling | 4.42 | NaN | 0.06 | APOE,LYZ,ALB,APOA1,etc |
| Systemic Lupus Erythematosus Signaling | 3.85 | NaN | 0.05 | C9,HNRNPA2B1,C7,IGHG4,etc |
| Remodeling of Epithelial Adherens Junctions | 3.55 | NaN | 0.09 | TUBB1,TUBB3,TUBB2A,TUBB,etc |
| Glycolysis I | 3.51 | NaN | 0.16 | TPI1,ENO2,FBP1,PFKP |
| Heme Degradation | 2.99 | NaN | 0.50 | BLVRA,BLVRB |
| phagosome maturation | 2.96 | NaN | 0.06 | TUBB1,CALR,DYNC1I2,TUBB3,etc |
| Atherosclerosis Signaling | 2.87 | NaN | 0.06 | APOE,LYZ,ALB,APOA1,S100A8,etc |
| IL-12 Signaling and Production in Macrophages | 2.70 | NaN | 0.05 | APOE,LYZ,ALB,APOA1,etc |
| Granzyme A Signaling | 2.65 | NaN | 0.15 | HIST1H1B,HIST1H1C,HIST1H1E |
| Glycogen Biosynthesis II (from UDP-D-Glucose) | 2.60 | NaN | 0.33 | GYS1,GYG1 |
| Ephrin B Signaling | 2.55 | 0.00 | 0.07 | CFL1,GNB2L1,ACP1,CAP1,etc |
| Production of Nitric Oxide and Reactive Oxygen Species in Macrophages | 2.55 | 1.41 | 0.04 | APOE,LYZ,ALB,APOA1,S100A8,etc |
| 14-3-3-mediated Signaling | 2.33 | NaN | 0.05 | TUBB1,TUBB3,TUBB2A,VIM,etc |
| Protein Kinase A Signaling | 2.25 | 0.90 | 0.03 | HIST1H1B,HIST1H1C,CALML5,HIST1H1E,etc |
