# Supplementary material for: Proteomic Analysis of Exudates from Chronic Ulcer of Diabetic Foot Treated with Scorpion Antimicrobial Peptide
Source: Mediators Inflamm. 2022 Oct 3;2022:5852786. doi: 10.1155/2022/5852786 (PMC9550419; doi:10.1155/2022/5852786)
Supplement: Supplementary Materials — Bacteriological identification of diabetic foot ulcer wounds is available on Supplementary Table 1–3. Identification results by mass spectrometry is available on Supplementary Table 4; analysis of proteins in diabetic wound exudate by iTRAQ is available on Supplementary Table 5; IPA technology for the annotation of differential proteins is available on Supplementary Table 6; classical signal pathway analysis of differential proteins is available on Supplementary Table 7; analysis of upstream regulatory factors is available on Supplementary Table 8; analysis of possible interaction networks in differential proteins is available on Supplementary Table 9. [file 5852786.f1.zip › Supplementary Table 8.docx]

Supplementary Table 8 Analysis of upstream regulatory factors

| **C-B** |  |  |  |  |  |
| --- | --- | --- | --- | --- | --- |
| Upstream regulator | Type | Activity | Z-value | *P* value | Identification of molecular |
| IgG | complex | Inhibited | -2.828 | 2.90E-06 | C5,DSP,FABP5,HSPB1,etc |
| RICTOR | other | Inhibited | -2.813 | 8.38E-05 | FABP5,PSMD4,RPL10,RPL26,etc |
| MAPK1 | kinase | Inhibited | -2.236 | 3.35E-02 | DDX58,GBP1,KRT17,LAP3,etc |
| ROCK2 | kinase | Activated | 2.813 | 4.80E-12 | DSG1,DSP,FABP5,IVL,etc |
| MYC | transcription regulator | Activated | 3.915 | 1.86E-11 | BCAT1,CSTB,DSP,EIF2S2,etc |
| MYCN | transcription regulator | Activated | 2.111 | 6.72E-10 | FKBP9,MYL12A,RBBP7,RPL10,etc |
| EGFR | kinase | Activated | 2.916 | 6.82E-07 | GBP1,HNRNPA1,HNRNPH1,HP,etc |
| HIF1A | transcription regulator | Activated | 2.336 | 6.87E-07 | CARS,ENO1,ENO2,ERO1A,etc |
| IFNG | cytokine | Activated | 2.277 | 1.09E-03 | ALDH1A3,DDX58,FABP5,FBP1,etc |
| ERK1/2 | group | Activated | 2.2 | 3.69E-03 | ENO1,KRT17,MAPK3,POSTN,etc |
| IL5 | cytokine | Activated | 2.412 | 3.89E-03 | ENO1,ERO1A,IGHG1,TPI1,etc |
| ARNT2 | transcription regulator | Activated | 2 | 2.44E-02 | KRT5,LTF,PGK1,POSTN |
| TP73 | transcription regulator | Activated | 2.177 | 3.07E-02 | IVL,KRT10,LTBP1,S100A2,etc |
| ESR1 | ligand-dependent nuclear receptor | Activated | 2.132 | 3.95E-02 | ANXA3,APOA1,BCAT1,C5,etc |
| IL22 | cytokine |  | -0.789 | 1.78E-12 | CALML3,CALML5,FLG,HP,etc |
| HRAS | enzyme |  | 1.254 | 9.08E-09 | ALDH1A3,ANXA3,GSN,HSPB1,etc |
| KRT14 | other |  |  | 4.80E-08 | DSG1,IVL,KRT1,KRT16,etc |
| TGFB1 | growth factor |  | 1.982 | 9.01E-08 | C5,CALML3,CARS,DSP,etc |
|  |  |  |  |  |  |
|  |  |  |  |  |  |
| **D-B** |  |  |  |  |  |
| RICTOR | other | Inhibited | -4.888 | 1.32E-14 | FABP5,PSMA2,PSMD4,RPL10,etc |
| IgG | complex | Inhibited | -3.207 | 1.23E-08 | APOE,ASAH1,C5,CALR,etc |
| CD3 | complex | Inhibited | -2.000 | 2.85E-05 | ARHGDIB,EIF4E,GBP1,GYS1,etc |
| FAAH | enzyme | Inhibited | -2.000 | 3.24E-04 | RPL17,RPL5,RPL7,RPS18 |
| FMR1 | other | Inhibited | -2.000 | 2.01E-03 | CFL1,EEF2,PFKP,PPIA |
| MAPK1 | kinase | Inhibited | -2.058 | 7.25E-03 | APOE,DDX58,GBP1,HBB,etc |
| ACOX1 | enzyme | Inhibited | -2.425 | 7.75E-03 | ACOX1,C9,CBR1,CSTB,etc |
| AMPK | complex | Inhibited | -2.180 | 9.60E-03 | FSCN1,SERPINE1,STAT1,VIM,etc |
| PRKAA2 | kinase | Inhibited | -2.213 | 9.96E-03 | ALB,ARHGDIB,CA2,VIM,etc |
| HDAC1 | transcription regulator | Inhibited | -2.000 | 1.62E-01 | APOA1,MYO1F,PCYT1A,RBBP7,etc |
| MYCN | transcription regulator | Activated | 3.807 | 4.10E-31 | DPYSL3,EEF1A1,EEF2,FKBP9,etc |
| MYC | transcription regulator | Activated | 4.408 | 2.31E-25 | ADD1,ALB,BCAT1,CSDE1,etc |
| TGFB1 | growth factor | Activated | 2.483 | 3.42E-14 | ABCF1,AHNAK,ALB,APOE,etc |
| ROCK2 | kinase | Activated | 2.138 | 3.05E-09 | DSP,FABP5,IVL,KRT1,etc |
| KITLG | growth factor | Activated | 2.000 | 1.06E-07 | BLVRB,CA2,EEF1A1,FABP5,etc |
| PPARA | ligand-dependent nuclear receptor | Activated | 2.008 | 1.40E-07 | ACAT2,ACOX1,APOA1,APOE,etc |
| HRAS | enzyme | Activated | 2.043 | 3.90E-07 | CALD1,CRP,GSN,HNRNPA2B1,etc |
| EGFR | kinase | Activated | 3.776 | 8.29E-07 | EIF6,GBP1,HNRNPA1,HNRNPA2B1,etc |
| IFNG | cytokine | Activated | 3.129 | 3.22E-06 | DDX5,DDX58,EEF1A1,ELAVL1,etc |
| HIF1A | transcription regulator | Activated | 2.847 | 1.07E-05 | APOE,EIF4E,ENO2,FSCN1,etc |
| NFE2L2 | transcription regulator | Activated | 3.025 | 2.46E-05 | C5,CBR1,FTH1,GNB2L1,etc |
| FN1 | enzyme | Activated | 2.144 | 3.48E-05 | ADD1,APOE,BCAT1,CRP,etc |
| ADORA2A | g-protein coupled receptor | Activated | 2.121 | 1.72E-04 | EEF2,EIF2S2,HNRNPK,KLC1,etc |
| EP300 | transcription regulator | Activated | 2.216 | 4.78E-04 | CA2,CRISPLD2,FTH1,GIMAP4,etc |
| IL5 | cytokine | Activated | 3.278 | 5.78E-04 | IGHG1,MAT2A,NDRG1,P4HA1,etc |
| CD38 | enzyme | Activated | 2.699 | 5.88E-04 | IGHG1,NDRG1,P4HA1,PFKP,etc |
| PRL | cytokine | Activated | 2.813 | 1.50E-03 | DDX58,HNRNPH2,KRT14,KRT5,etc |
| ERBB2 | kinase | Activated | 2.556 | 1.84E-03 | AHNAK,EIF6,FSCN1,G3BP1,etc |
| CD40LG | cytokine | Activated | 2.412 | 2.16E-03 | CA2,FSCN1,IGHG1,MIF,etc |
| IGF1 | growth factor | Activated | 2.192 | 3.15E-03 | CAMP,DDX5,EIF4E,HBB,etc |
| RAF1 | kinase | Activated | 2.828 | 3.96E-03 | ACAT2,CA2,CRP,HNRNPA2B1,etc |
| SP1 | transcription regulator | Activated | 2.397 | 4.91E-03 | ANK1,APOA1,APOE,CAMP,etc |
| STAT4 | transcription regulator | Activated | 2.060 | 4.94E-03 | AHNAK,ENO2,FSCN1,NDRG1,etc |
| SYVN1 | transporter | Activated | 2.449 | 5.70E-03 | BCAT1,HSPB1,PCBP1,RPL10,etc |
| ESR1 | ligand-dependent nuclear receptor | Activated | 2.126 | 5.74E-03 | ADAMTS13,AHNAK,APOA1,APOE,etc |
| XBP1 | transcription regulator | Activated | 2.433 | 1.12E-02 | APOA1,CALR,FKBP10,GOLPH3,etc |
| SPP1 | cytokine | Activated | 2.196 | 1.93E-02 | CA2,DSP,S100A6,SERPINE1,etc |
| Nfat (family) | group | Activated | 2.000 | 2.20E-02 | CALD1,EHD4,SERPINE1,TUBB3 |
| ANGPT2 | growth factor | Activated | 2.213 | 2.62E-02 | CALR,FKBP10,MIF,SERPINE1,etc |
| GLI1 | transcription regulator | Activated | 2.207 | 2.99E-02 | HNRNPU,LMNA,PCDH18,RPL30,etc |
| EGF | growth factor | Activated | 2.568 | 3.16E-02 | APOA1,DDX5,EEF1A1,HNRNPK,etc |
| F2 | peptidase | Activated | 2.534 | 3.21E-02 | CALD1,CALR,CORO1C,EHD4,etc |
| POLG | enzyme |  |  | 7.44E-12 | GNB2L1,RPL13,RPL23,RPL27A,etc |
| IL22 | cytokine |  | -1.583 | 7.86E-10 | CALML3,CALML5,FGA,FLG,etc |
| EPO | cytokine |  | 0.388 | 1.57E-09 | ANK1,BLVRB,CA1,CA2,etc |
| HNF1A | transcription regulator |  | -0.847 | 1.77E-08 | ACAT2,ALB,ANGPTL3,APOH,etc |
| OSM | cytokine |  | 0.378 | 2.04E-08 | ALB,ASAH1,C4BPA,CRP,etc |
