# Supplementary material for: Proteomic Analysis of Exudates from Chronic Ulcer of Diabetic Foot Treated with Scorpion Antimicrobial Peptide
Source: Mediators Inflamm. 2022 Oct 3;2022:5852786. doi: 10.1155/2022/5852786 (PMC9550419; doi:10.1155/2022/5852786)
Supplement: Supplementary Materials — Bacteriological identification of diabetic foot ulcer wounds is available on Supplementary Table 1–3. Identification results by mass spectrometry is available on Supplementary Table 4; analysis of proteins in diabetic wound exudate by iTRAQ is available on Supplementary Table 5; IPA technology for the annotation of differential proteins is available on Supplementary Table 6; classical signal pathway analysis of differential proteins is available on Supplementary Table 7; analysis of upstream regulatory factors is available on Supplementary Table 8; analysis of possible interaction networks in differential proteins is available on Supplementary Table 9. [file 5852786.f1.zip › Supplementary Table 9.docx]

Supplementary Table 9 Analysis of possible interaction networks in differential proteins

| **C-B** |  |  |  |  |
| --- | --- | --- | --- | --- |
| No. | Identification of proteins | Score | Number of molecules | Major biological functions |
| 1 | 60S ribosomal subunit,A2ML1,CALML3,CALML5,etc | 72 | 31 | Dermatological Diseases and Conditions, Immunological Disease, Inflammatory Disease |
| 2 | 26s Proteasome,ALDH1A3,Alpha catenin,BCAT,etc | 36 | 19 | Carbohydrate Metabolism, Post-Translational Modification, Protein Folding |
| 3 | AMPK,APOA1,APOH,APRT,etc | 34 | 19 | Developmental Disorder, Hereditary Disorder, Immunological Disease |
| 4 | ACAT2,AGT,ANXA3,AP3B2,etc | 29 | 16 | Cellular Movement, Hematological Disease, Immunological Disease |
| 5 | Actin,Calmodulin,EIF2S2,F Actin,etc | 27 | 15 | RNA Post-Transcriptional Modification, Cardiovascular Disease, Developmental Disorder |
| 6 | ADSS,APP,BABAM1,CALML3,etc | 22 | 14 | Energy Production, Molecular Transport, Nucleic Acid Metabolism |
| 7 | ACAT2,ACP1,Ap1,CD3,etc | 20 | 12 | Hematological System Development and Function, Immune Cell Trafficking, Inflammatory Response |
|  |  |  |  |  |
| **D-B** |  |  |  |  |
| 1 | 60S ribosomal subunit,ABCF1,CBR1,EHD4,etc | 63 | 32 | Gene Expression, Protein Synthesis, Cancer |
| 2 | CALD1,CNDP1,CNDP2,CORO1C,etc | 52 | 28 | RNA Post-Transcriptional Modification, Infectious Diseases, Protein Synthesis |
| 3 | ASAH1,Akt,CAP1,CFL1,etc | 46 | 26 | Dermatological Diseases and Conditions, Immunological Disease, Inflammatory Disease |
| 4 | 26s Proteasome,CALML5,CALR,Cyclin E,etc | 44 | 25 | Cancer, Cell Death and Survival, Organismal Injury and Abnormalities |
| 5 | Beta Tubulin,C5,C5-C6-C7,C5-C6-C7-C8,etc | 33 | 21 | Developmental Disorder, Hereditary Disorder, Immunological Disease |
| 6 | ACOX1,APP,BTF3,CALML3,etc | 30 | 20 | Organismal Injury and Abnormalities, Cellular Assembly and Organization, Nervous System Development and Function |
| 7 | AHNAK,ARHGDIB,BAG6,BCAT1,etc | 30 | 19 | Infectious Diseases, Antimicrobial Response, Cell-To-Cell Signaling and Interaction |
| 8 | ARL8B,ATXN3,Actin,CLTA,etc | 29 | 19 | RNA Post-Transcriptional Modification, Connective Tissue Disorders, Metabolic Disease |
| 9 | A2ML1,ANGPTL3,APMAP,APOH,etc | 29 | 20 | Cell Death and Survival, Cardiovascular Disease, Respiratory Disease |
| 10 | ADAMTS13,ALB,ALT,AP1G1,etc | 26 | 17 | Cardiovascular System Development and Function, Lymphoid Tissue Structure and Development, Organismal Development |
| 11 | APOA1,ARPC4,Alpha catenin,Alpha tubulin,etc | 26 | 17 | Hereditary Disorder, Metabolic Disease, Organismal Injury and Abnormalities |
| 12 | ACAT2,ACP1,AGL,Alp,etc | 24 | 16 | Cancer, Cell Cycle, Small Molecule Biochemistry |
| 13 | 14-3-3,ADD1,AMPK,ANK1,etc | 21 | 16 | Developmental Disorder, Hereditary Disorder, Metabolic Disease |
